# Irreversible *endo*-Selective Diels–Alder Reactions of Substituted Alkoxyfurans: A General Synthesis of *endo*-Cantharimides

**DOI:** 10.1002/chem.201406286

**Published:** 2015-03-10

**Authors:** Robert W Foster, Laure Benhamou, Michael J Porter, Dejan-Krešimir Bučar, Helen C Hailes, Christopher J Tame, Tom D Sheppard

**Affiliations:** [a] Department of Chemistry, University College London, Christopher Ingold Laboratories20 Gordon Street, London, WC1H 0AJ (UK); [b] GlaxoSmithKline, Medicines Research CentreGunnels Wood Road, Stevenage, Herts, SG1 2NY (UK)

**Keywords:** cantharimides, cycloadditions, dienes, furans, phthalimide

## Abstract

The [4+2] cycloaddition of 3-alkoxyfurans with N-substituted maleimides provides the first general route for preparing *endo*-cantharimides. Unlike the corresponding reaction with 3*H* furans, the reaction can tolerate a broad range of 2-substitued furans including alkyl, aromatic, and heteroaromatic groups. The cycloaddition products were converted into a range of cantharimide products with promising lead-like properties for medicinal chemistry programs. Furthermore, the electron-rich furans are shown to react with a variety of alternative dienophiles to generate 7-oxabicyclo[2.2.1]heptane derivatives under mild conditions. DFT calculations have been performed to rationalize the activation effect of the 3-alkoxy group on a furan Diels–Alder reaction.

## Introduction

To access new areas of chemical space, medicinal chemistry programs are increasingly focusing on fragments and scaffolds with rigid 3D structures that contain a significant proportion of sp^3^ carbon atoms.[1] This in turn presents a considerable synthetic challenge as these molecules are generally not straightforward to synthesize, and late-stage derivatization is often far from trivial. Further challenges reside in the control of relative and absolute stereochemistry due to the presence of numerous chiral centres. Current structural scaffolds of interest include strained small-ring molecules (cyclopropanes, oxetanes, azetidines),[2] as well as fused (dihydrobenzofurans, indolines, tetrahydroquinolines)[3] and bridged bicylic and polycyclic compounds (bicyclopentanes, cubanes, etc).[4] Natural products have also traditionally provided chemists with inspiration, as they include bioactive molecules with complex 3D architectures.[5] Many of these compounds, however, have high molecular weights or are too structurally complex to be suitable for use as scaffolds for medicinal chemistry applications. Nevertheless, smaller natural products contain ring systems that are potentially ideal scaffolds for use in medicinal chemistry, provided that efficient synthetic routes can be developed with appropriate functional groups at positions on the central core.

**Figure 1:**
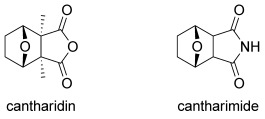
Natural product cantharidin and cantharimide.

The *exo*-cantharimide skeleton (Figure 1, derived from cantharidin, a natural product secreted by many species of blister beetle with well-established cytotoxic activity)[6] has been exploited in a wide range of molecules with useful biological properties. The motif is present in several cytotoxic compounds,[7] antiplasmodial agents,[8] androgen receptor antagonists[9] and in a positive allosteric modulator of the metabotropic glutamate receptor 4 (mGlu4).[10] More generally, the 7-oxabicyclo[2.2.1]heptyl skeleton is found in a number of other important natural products[11]–[13] and it has proved to be a valuable intermediate for synthetic chemists.[14]–[17] The properties of the *exo*-cantharimide skeleton have been extensively explored with a range of N-substituted derivatives showing useful biological properties. However, there are few methods for the introduction of substituents around the 7-oxabicyclo[2.2.1]heptyl ring system.[18] Furthermore, the corresponding *endo*-cantharimide scaffold has rarely been reported at all.[19]

**Scheme 1:**
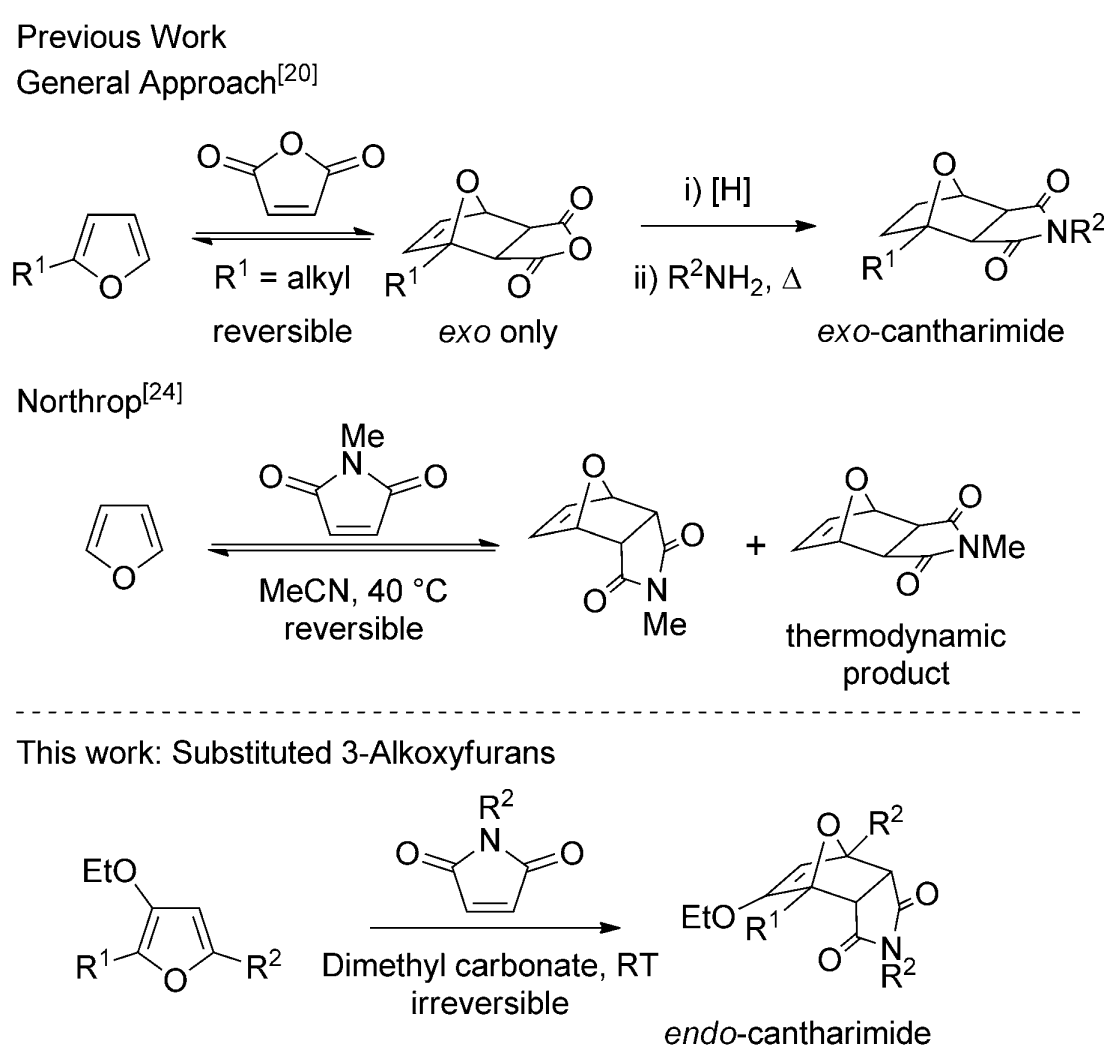
Synthetic approaches to cantharimides.

The *exo*-cantharimide skeleton is typically prepared by the [4+2] cycloaddition of furans and maleic anhydride, followed by alkene reduction and condensation with an amine (Scheme 1).[20] A curious feature of the cycloaddition reaction is the high stereoselectivity for the *exo* diastereomer observed, believed to be the result of a highly reversible cycloaddition process which is operating under thermodynamic control.[21], [22] It is possible to access the corresponding *endo*-cantharimide by a Diels–Alder reaction of furan with maleimide;[23] however, experimental and computational studies have shown that this reaction is under thermodynamic control with the *exo*-cantharimide being the thermodynamic product.[24] As a consequence, the *endo*-adduct of maleimide and furan is known to rapidly isomerize either in hot solvent or when exposed to visible light, which impedes both the isolation and application of these compounds.[25] Another serious limitation of furan Diels–Alder reactions is that any deactivating substituents on the furan have a profound effect on the equilibrium position of the cyclization. For example, there are no reported examples of the [4+2] cycloaddition of 2-aryl or 2-heteroaryl furans with dienophiles of any type.

There is a long tradition of activating dienes for Diels–Alder reactions through the use of electron-donating substituents, which are known to reduce the activation energy for the cycloaddition reaction.[26] However, this is generally a kinetic effect and reducing the kinetic barriers to a thermodynamically controlled reaction would only increase the rate at which isomerization occurs. To access stable *endo*-cantharimides it is therefore necessary to develop reactions with a significantly improved thermodynamic driving force.[24]

**Scheme 2:**
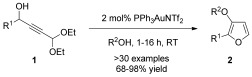
Gold-catalysed synthesis of 3-alkoxyfurans 2 from propargylic alcohols 1.[27]

We have recently developed a straightforward approach to 2-substituted-3-alkoxyfurans by gold-catalysed solvolytic cyclisation of suitably functionalised propargylic alcohols (Scheme 2).[27] Preliminary studies indicated that 3-alkoxyfurans underwent rapid and *endo*-selective reactions with *N*-methylmaleimide to generate kinetically stable cantharimide products. The distinct 3D structure of the *endo*-cantharimide motif, coupled with its physical properties, should make it a valuable new scaffold for medicinal chemistry applications. Such an approach should enable control of substituents at a variety of positions on the tricyclic ring system.

## Results and Discussion

The reaction of 3-ethoxyfuran **2 a** with 1.2 equivalents of *N*-methylmaleimide proceed in a variety of solvents at room temperature to give cantharimide **3 a** in near quantitative yield (Table 1, entries 1 to 4). Crucially the cantharimide was formed with a clear preference for the *endo* diastereomer and the two isomers could be readily separated by flash column chromatography. The identity of the solvent had little impact on yield or diastereoselectivity, so dimethyl carbonate (DMC) was selected on the grounds of its excellent environmental profile.[28] The reaction could also be scaled up to use 1 g of furan **2 a**, giving cantharimide **3 a** in 95 % yield (entry 5, *endo*/*exo* ratio of 75:25). A purified sample of *endo*-**3 a** was treated under the same reaction conditions and no isomerization was observed, suggesting the reaction proceeds under kinetic control. However, it was possible to increase the proportion of *exo*-**3 a** by heating the reaction at 80 °C for 16 h (entry 6). The cyclization was equally effective when 3-methoxyfuran **2 b** was used as a diene, giving the corresponding adduct in excellent yield as an 80:20 mixture of *endo* and *exo* diastereomers (entry 7).

**Table 1:** [4+2] cycloaddition of 3-alkoxyfurans 2 with *N*-methylmaleimide.

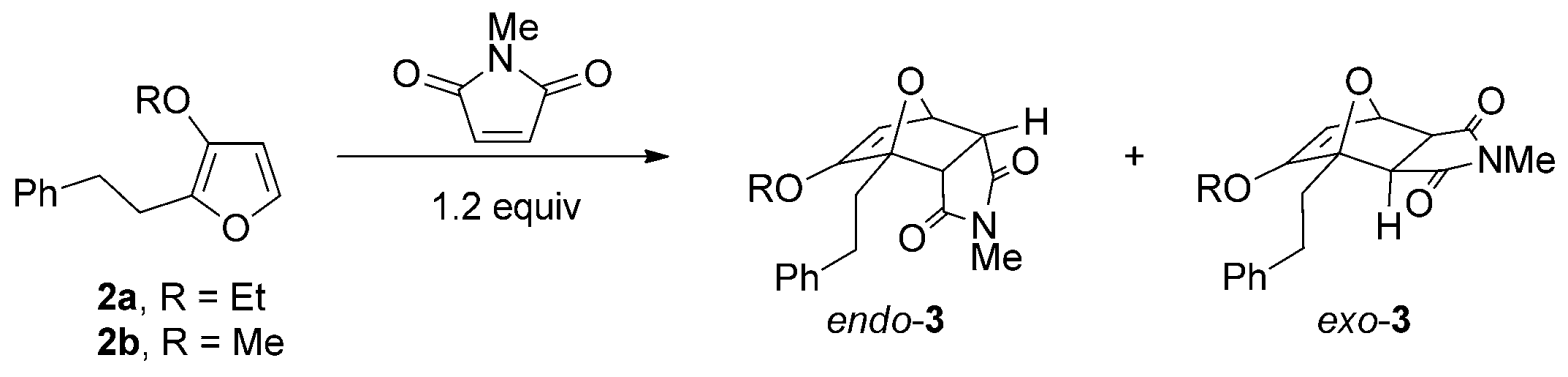							
Entry	R	Solvent[a]	*T* [°C]	Reaction *t* [h]	Product	Yield [%]	*endo*/*exo*[b]
1	Et	Et_2_O	25	4	**3 a**	98[c]	65:35
2	Et	PhMe	25	4	**3 a**	100[c]	65:35
3	Et	EtOH	25	4	**3 a**	100[c]	70:30
4	Et	DMC	25	4	**3 a**	93[d]	70:30
5[e]	Et	DMC	25	4	**3 a**	95[d]	75:25
6	Et	DMC	80	16	**3 a**	93[d]	55:45
7	Me	DMC	25	4	**3 b**	89[d]	80:20

[a] DMC refers to dimethyl carbonate.

[b] Determined by analysis of the crude ^1^H NMR spectrum.

[c] Yield determined by ^1^H NMR spectroscopy using pentachlorobenzene as an internal standard.

[d] Isolated yield.

[e] Reaction conducted with 1.0 g of furan **2 a**.

**Figure 2:**
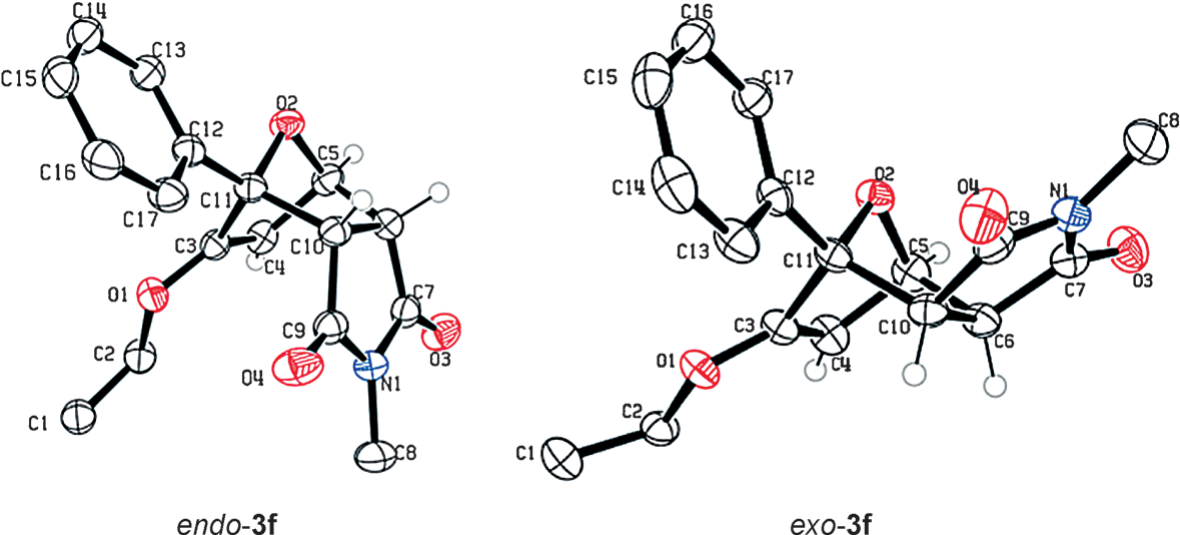
Crystal structures of cantharimides 3 f. Ellipsoids are shown at the 50 % probability level. Only hydrogen atoms belonging to the cyclic core are shown for clarity.[29]

These reaction conditions were applied to a wide range of 3-ethoxyfurans with different substituents at the 2-position, with the results summarized in Table 2. The reaction tolerated furans with primary and secondary aliphatic substituents (Table 2, entries 2 and 3). It was also possible to incorporate a *tert*-butoxycarbonyl (*N-*Boc) piperidine, as shown in entry 4. The reaction was very effective with an aromatic group at the 2-position, giving the first reported examples of 4-arylcantharimides (entries 5 to 10). The reaction of 2-phenylfuran **2 f** gave an 80:20 mixture of *endo* and *exo* diastereomers in good yield. This reaction could also be conducted on a 1.0 g scale, giving the two diastereomers **3 f** in a combined yield of 86 %, and with complete isomeric separation following chromatography on silica gel. The relative stereochemistry of the two diastereomers was confirmed by X-ray crystallography (Figure 2).

**Table 2:** [4+2] cycloaddition of 3-alkoxyfurans 2 with *N*-methylmaleimide.

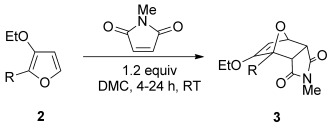			
Entry	R	Isolated yield [%][a]	*endo*/*exo*[b]
1	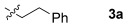	93	70:30
2	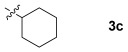	95	85:15
3	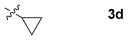	90	80:20
4	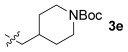	85	75:25
5	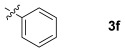	86 (86)[c]	80:20
6	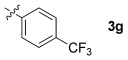	75	80:20
7	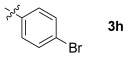	78	80:20
8	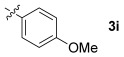	95	80:20
9	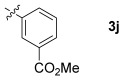	84	75:25
10	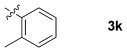	86	80:20
11	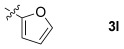	85	90:10
12	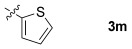	96	70:30
13	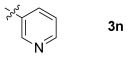	92	70:30

[a] Combined isolated yield of *endo*-**3** and *exo*-**3**.

[b] Determined by analysis of the ^1^H NMR spectrum of the crude product.

[c] Reaction conducted with 1.0 g of furan **2 f**.

**Scheme 3:**
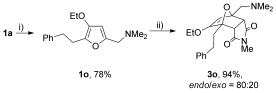
Synthesis of a 7-substituted *endo*-cantharimide: i) (H_2_C—NMe_2_)I (2 equiv), MeCN, 16 h, RT; ii) *N*-methylmaleimide (1.2 equiv), DMC, 24 h, RT.

The reaction was tolerant of electron-poor aromatic substituents (Table 2, entries 6 and 9), an electron-rich aromatic substituent (entry 8) and an aryl bromide substituent (entry 7). It was also possible to use a sterically encumbered 2-tolyl substituent to give cantharimide **3 k** in 86 % yield. Furthermore, the reaction was effective when the 3-alkoxyfuran possessed a heteroaromatic substituent, as can be seen in entries 11 to 13 (85–96 % yields). The chemoselective reaction of bis-furan **2 l** with *N-*methylmaleimide to give exclusively the enol ether adduct is an interesting demonstration of the high reactivity of the 3-alkoxyfuran unit in a [4+2] cycloaddition reaction. It was also possible to functionalize a 3-alkoxyfuran at the 5-position prior to the cycloaddition reaction, in order to introduce a substituent at the 7-position of the *endo*-cantharimide scaffold (Scheme 3).

The cycloaddition of 3-alkoxyfuran **2 a** was effective with a number of alternative N*-*substituted maleimides, as illustrated in Table 3.[30] Sterically more challenging N*-*substituents could be incorporated in high yield and without an extended reaction time.

**Table 3:** [4+2] cycloaddition of 3-alkoxyfuran 2 a with maleimides 4.

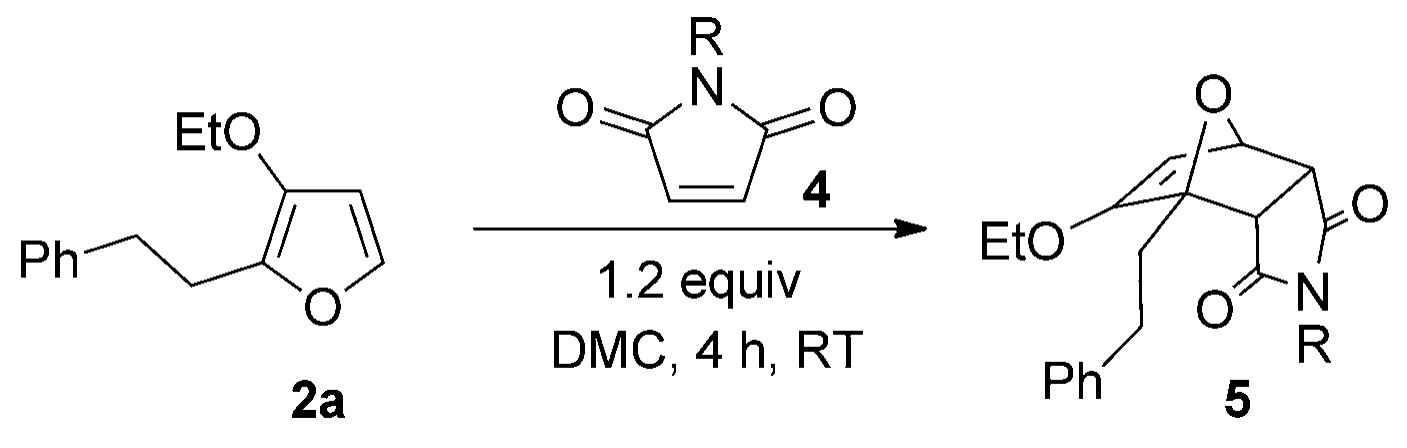			
Entry	R	Yield5[%]	*endo*/*exo*[a]
1	Ph **4 a**	94	65:35
2	4-MeC_6_H_4_ **4 b**	83	55:35
3	c-Pr **4 c**	87	60:40

[a] Determined by analysis of the ^1^H NMR spectrum of the crude product.

**Scheme 4:**
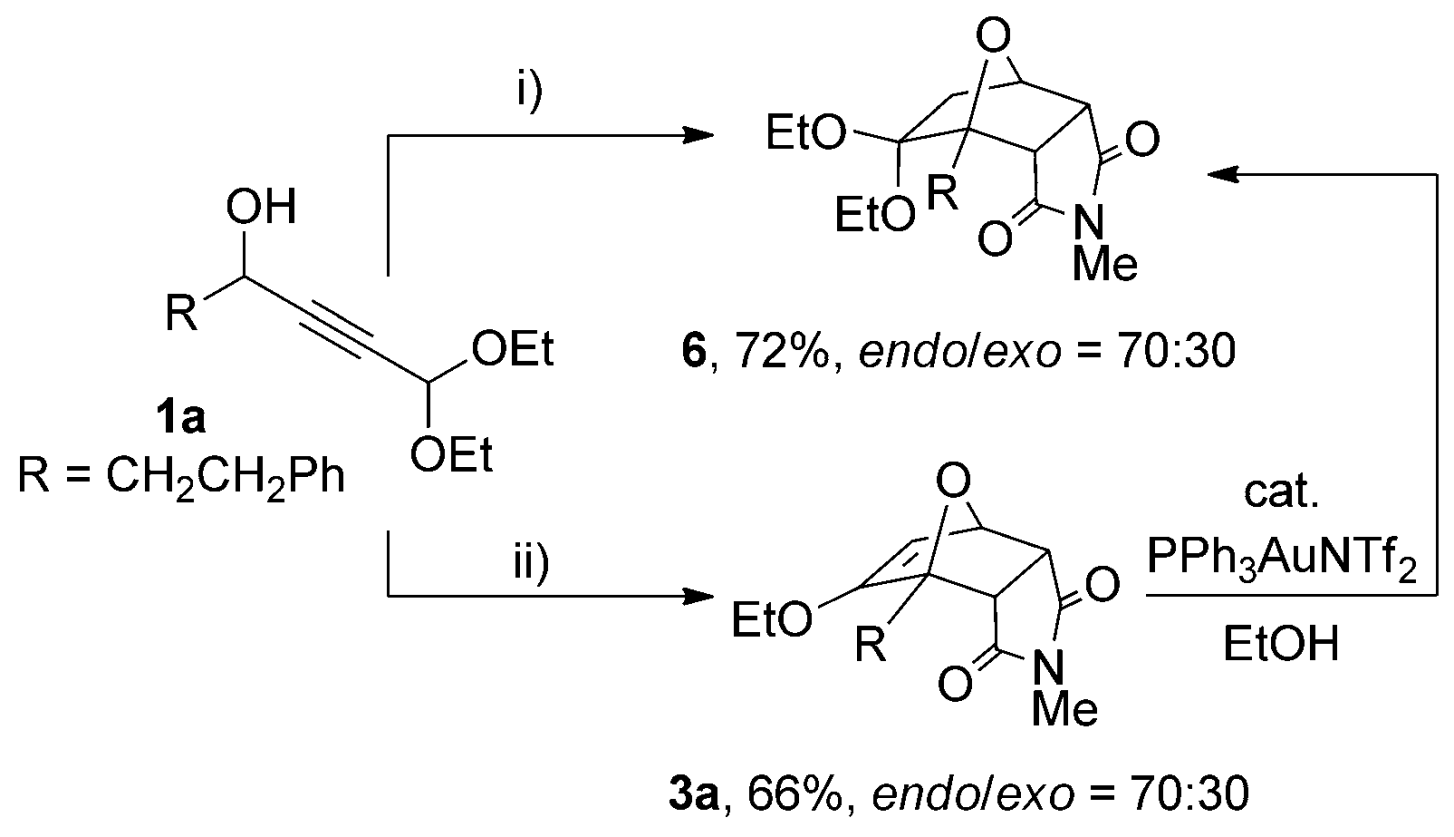
One-pot cantharimide synthesis from propargylic alcohol 1 a. i) *N*-methylmaleimide, 2 mol % PPh_3_AuNTf_2_, EtOH; ii) 2 mol % PPh_3_AuNTf_2_, EtOH then 2.5 mol % PPh_3_ then *N*-methylmaleimide.

Additionally, it was possible to combine the gold-mediated furan synthesis with the cycloaddition reaction in a single step (Scheme 4, conditions i). Treating propargylic alcohol **1 a** with gold catalyst and *N-*methylmaleimide gave diethyl acetal **6** in good yield. It appeared that the gold catalyst was responsible for the in situ conversion of enol ether **3 a** into the corresponding diethyl acetal, as the interconversion can be avoided by poisoning the catalyst with 2.5 mol % PPh_3_ prior to addition of the *N-*methylmaleimide, to give enol ether **3 a** (*endo*/*exo* ratio of 70:30). Treatment of a sample of enol ether **3 a** with catalytic PPh_3_AuNTf_2_ in ethanol was also observed to result in formation of acetal **6**.

### Transformation of cycloaddition products

**Scheme 5:**
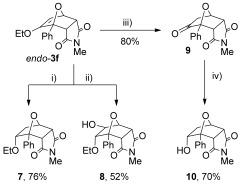
Functional-group interconversion of enol ether *endo*-3 f: i) H_2_, 10 % Pd/C; ii) 9-BBN then H_2_O_2_/NaOH; iii) SCX-2 cartridge; iv) NaBH_4_, MeOH. 9-BBN=9-borabicyclo[3.3.1]nonane.

The *endo*-cantharimides contain an enol ether moiety, which can be readily transformed into a variety of functional groups (Scheme 5). For example, enol ether *endo*-**3 f** can by hydrogenated to generate ether **7** with complete diastereocontrol.[31] The enol ether also underwent hydroboration and oxidation to give alcohol **8**, with complete regio- and stereocontrol. Enol ether *endo*-**3 f** could be hydrolysed to give ketone **9** in good yield by passing it through a strong cation exchange (SCX-2) cartridge.[32] Treating ketone **9** with NaBH_4_ afforded alcohol **10**, again with high stereocontrol.

**Scheme 6:**
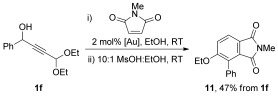
Synthesis of substituted phthalimide 11 by acid-catalysed aromatisation of cantharimide intermediates.

The acid-mediated aromatization of 7-oxabicyclo[2.2.1]heptane derivatives has been previously applied to the synthesis of aromatic rings, and this approach could be used to prepare substituted phthalimide **11**.[33] The one-pot cantharimide synthesis described in Scheme 4 was used to convert alcohol **1 f** into the crude cantharimide, which could be converted into phthalimide **11** by acid-mediated ring-opening and aromatization (Scheme 6).

### Physicochemical properties

**Figure 3:**
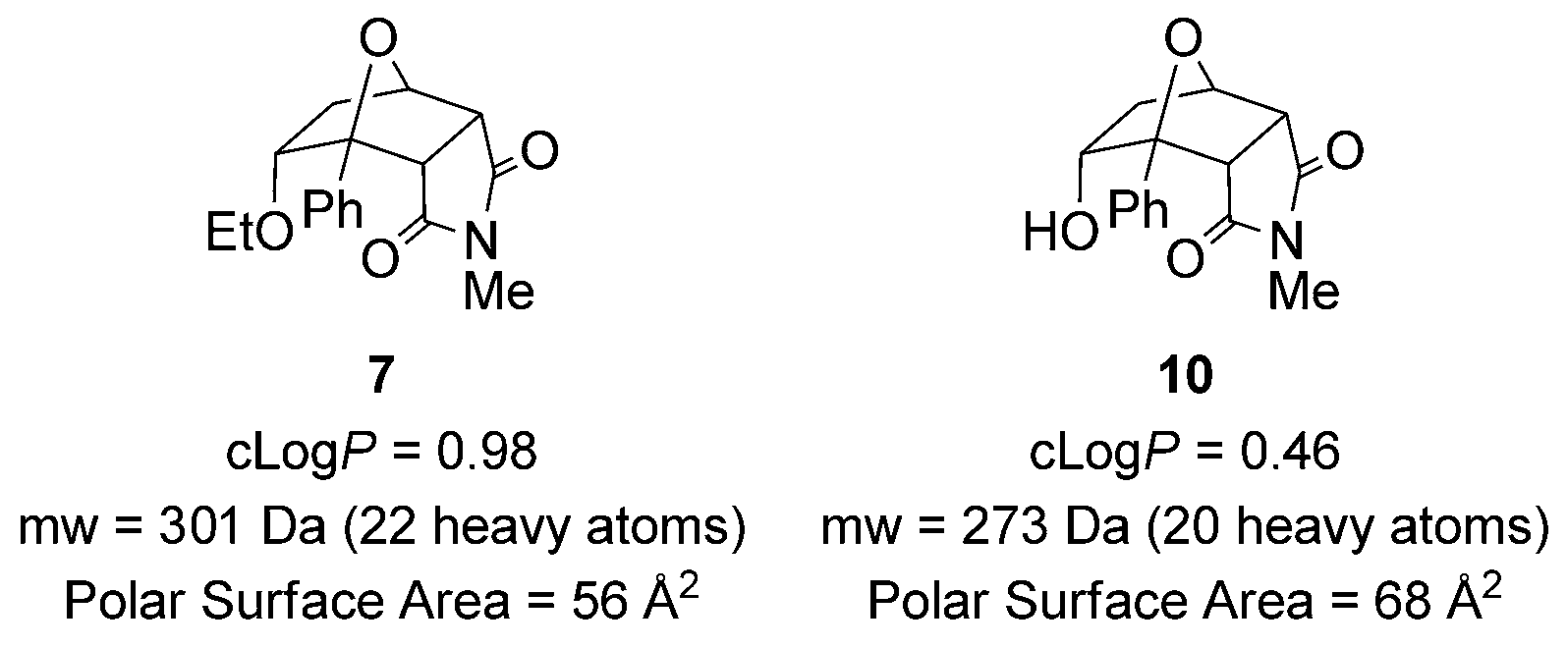
Physicochemical properties of *endo-*cantharimides.[39]

An important challenge for drug development is the generation of novel heterocyclic building blocks with suitable properties for use in screening and medicinal chemistry programs.[34] The cantharimides accessed using this methodology have appropriate physicochemical properties for lead-like compounds, including lipophilicity,[35] molecular weight and polar surface area[36] (Figure 3). Another attractive feature of these scaffolds is the high proportion of sp^3^-hybridized carbon atoms, which is typically associated with improved protein binding selectivity and frequency, better solubility and a reduced chance of off-target effects.[37] Indeed, cantharimides **7**, **10**, *endo*-**3 f** and *exo*-**3 f** were screened against the hERG receptor (IC_50_>50 μm) and the aryl hydrocarbon receptor (EC_50_>100 μm), which are responsible for common off target effects, and no affinity was observed. In addition the in vitro clearance of alcohol **10** in the presence of human microsomes was determined and only a low level of turnover was observed (<0.53 mL min^−1^ g^−1^).[38]

### [4+2] cycloadditions with other dienophiles

The [4+2] cycloaddition of furans with maleate esters is known but was reported to require either forcing pressure[40] or high catalyst loadings of a Lewis acid.[41] In contrast, the catalyst-free reaction of dimethyl maleate **12 a** and furan **2 a** proceeded at room temperature to give adduct **13 a** in a good yield and with excellent *endo* selectivity (Table 4, entry 1). The reactions of dimethyl and diethyl fumarate (**12 b** and **12 c**) with furan **2 a** proceeded more rapidly, giving the corresponding adducts in 77–89 % yield after 4 h (entries 2 and 3). There is a clear selectivity in both examples for the product which possessed *exo* stereochemistry with respect to the 3-position (3-*exo*-**13**).[42] Heating furan **2 a** with ethyl vinyl ketone at 80 °C for 16 h, followed by hydrolysis of the enol ether on an SCX-2 cartridge, gave diketone **13 d** with high regiocontrol (95:5), although as a 60:40 mixture of *endo*/*exo* isomers.

**Table 4:** [4+2] cycloaddition of 3-alkoxyfuran 2 a with different dienophiles.

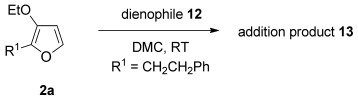					
Entry	Dienophile12	Reaction *t* [h]	Product13	Isolated yield [%]	Product ratio[a]
1	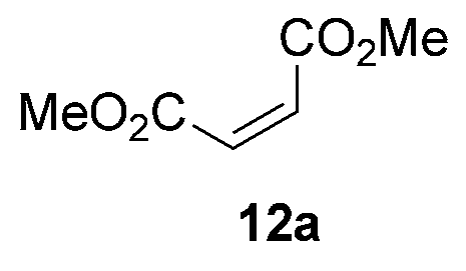	72	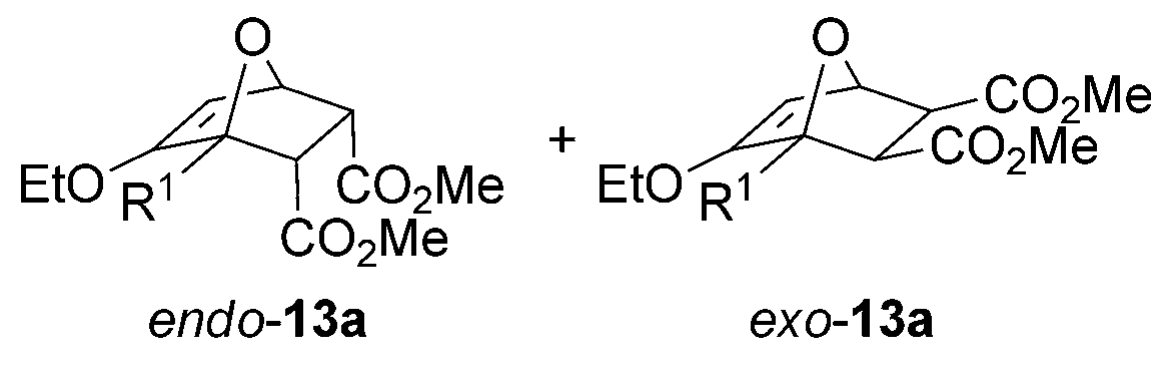	70	12:1
2	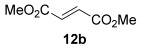	4	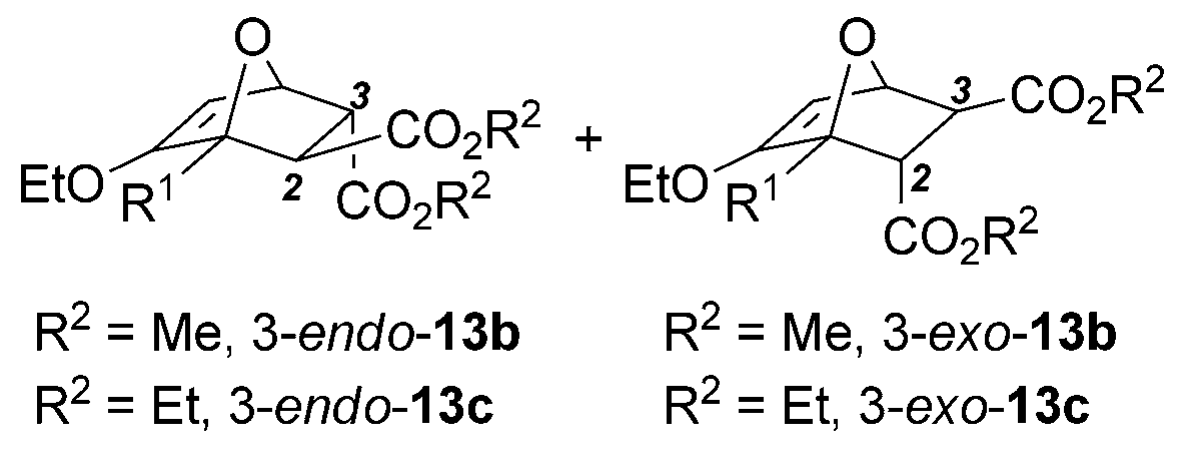	77	15:85
3	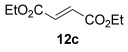	4		89	15:85
4[b,c]	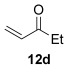	16	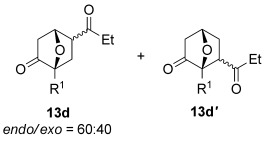	60	95:5
5[c,d]	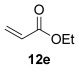	6	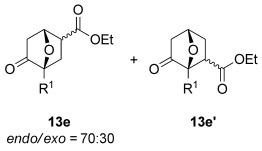	89	95: 5

[a] Determined by analysis of the ^1^H NMR spectrum of the crude product.

[b] Reaction conducted at 80 °C.

[c] Crude product flushed through a SCX-2 cartridge.

[d] Reaction conducted with 2 mol % HfCl_4_.[41]

The catalyst-free reaction of furan **2 a** with ethyl acrylate **12 e** was relatively slow at room temperature, with <100 % conversion after 24 h. However, it was possible to accelerate the reaction through the use of 2 mol % HfCl_4_, giving the ketone **13 e** in 89 % yield and with good regiocontrol (95:5) after 6 h at room temperature (Table 4, entry 5). The catalyst loading for this reaction is much lower than the high (sometimes stoichiometric) loading reported for the Lewis acid-catalyzed reactions of 3*H* furans and acrylates.[41], [43]

### Computational study

The reactions of five 3-alkoxyfurans and *N*-methylmaleimide were explored with the M06-2X exchange–correlation function of Truhlar et al.,[44] a density functional that has been successfully used to model the reaction and activation energies of different cycloaddition processes.[45] 2-Substituted-3-methoxyfurans were chosen as suitable models for our 3-alkoxyfurans and these were compared to the corresponding 3*H* furans.

**Figure 4:**
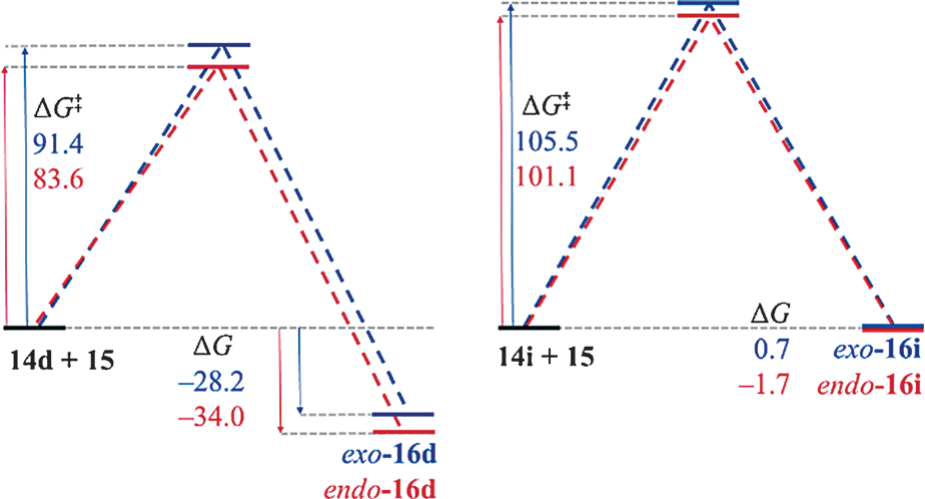
Calculated Δ*G* and Δ*G*^≠^ values for the reactions of 2-phenylfurans 14 d and 14 i and *N*-methylmaleimide 15 in kJ mol^−1^.

**Figure 5:**
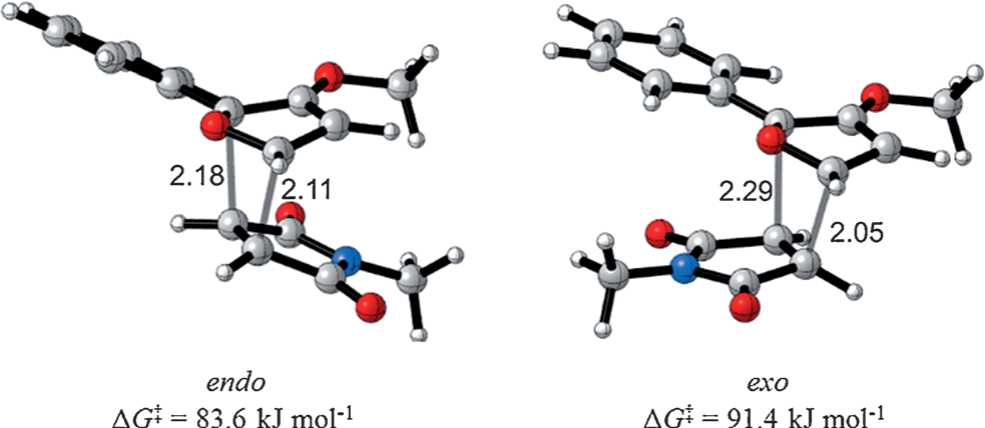
M06-2X/6-31G(d)-optimized *endo* and *exo* transition states for the reaction of furan 14 d and *N*-methylmaleimide 15.[46] Distances in Å.

The 3-alkoxy group has a dramatic effect on the thermodynamics of the cycloaddition reaction, as is evident in Table 5. All five reactions of 3-alkoxyfurans have a clear thermodynamic driving force for the formation of both *endo*- and *exo*-addition products (Figure 5) and the data is consistent with a reaction that is likely to be kinetically controlled. In contrast, the values of Δ*G* for the corresponding reactions of 3*H* furans are all greater by 24–34 kJ mol^−1^ (Figure 4). This effect is most significant when the 2-substituent is aromatic, as this results in a value of Δ*G* close to zero for the furans **14 h**–**j**. As expected, the 3-alkoxy group also has a significant effect on the free energy of activation for the cycloaddition reaction, with the kinetic barrier reduced by 11–23 kJ mol^−1^. The effect of solvation on these reactions was also considered but was found to have little effect (see Supporting Information).

**Table 5:** Calculated Δ*G* and Δ*G*^≠^ for the reactions of furans 14 and *N*-methylmaleimide 15.[a]

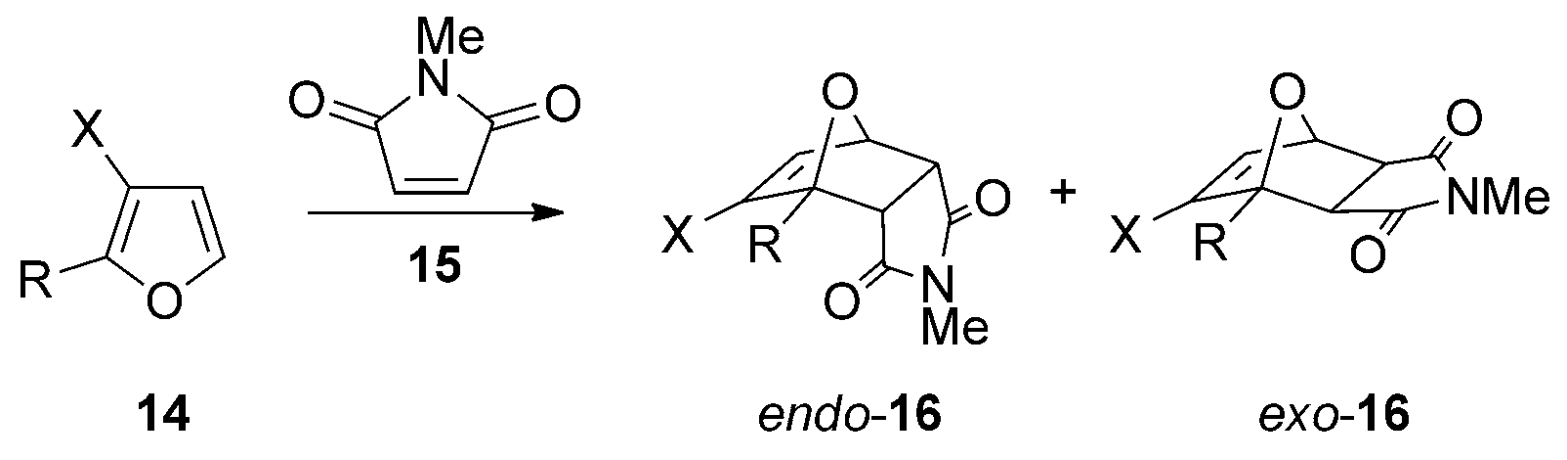						
14	R	X	*endo*		*exo*	
			Δ*G*	Δ*G*^≠^	Δ*G*	Δ*G*^≠^
**14 a**	Me	OMe	−41.5	81.1	−47.7	82.1
**14 b**	^*c*^Pr	OMe	−44.1	75.0	−53.2	78.6
**14 c**	4-MeOC_6_H_4_	OMe	−32.5	76.8	−30.1	85.3
**14 d**	Ph	OMe	−34.0	83.6	−28.2	91.4
**14 e**	4-F_3_CC_6_H_4_	OMe	−25.3	85.9	−23.8	96.8
						
**14 f**	Me	H	−11.8	97.9	−16.8	96.2
**14 g**	^*c*^Pr	H	−12.5	92.2	−10.3	92.8
**14 h**	4-MeOC_6_H_4_	H	−6.1	95.4	−3.6	98.8
**14 i**	Ph	H	−1.7	101.1	0.7	105.5
**14 j**	4-F_3_CC_6_H_4_	H	−0.9	101.6	0.9	108.3

[a] All values in kJ mol^−1^. All data is calculated for species in the gas phase.

**Scheme 7:**
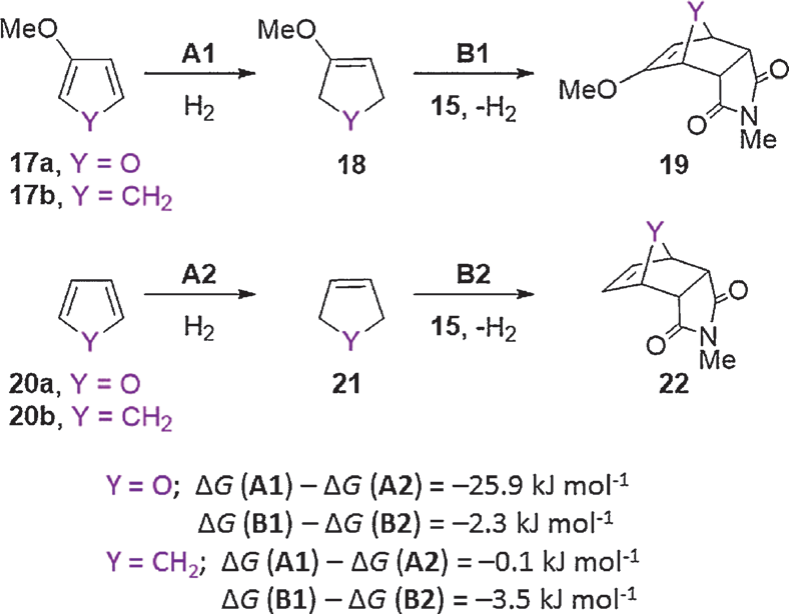
Thermodynamic cycle involving the hydrogenation of dienes 17 and 20.

The reversibility of most furan Diels–Alder reactions has been attributed to the loss of aromatic stabilization upon formation of an adduct, which results in a facile retro-cycloaddition.[22] In order to examine the effect of a 3-methoxy group on this phenomenon, thermodynamic cycles involving the partial hydrogenation of 3-methoxyfuran **17 a** and furan **20 a** to the corresponding 2,5-dihydrofurans **18 a** and **21 a** were considered (Scheme 7). It is notable that the free energy of hydrogenation for furan **20 a** was 25.9 kJ mol^−1^ greater than for 3-methoxyfuran **17 a**. The corresponding reaction free energies for cyclopentadienes **17 b** and **20 b** were also calculated but no significant difference was observed. The implications of these calculations are that 1) the difference in behaviour between 3*H* and 3-methoxy furans in cycloaddition reactions can be attributed to differences associated with loss of aromaticity rather than with C—C bond formation and 2) a 3-methoxy group can reduce the energetic penalty associated with the loss of aromaticity upon the Diels–Alder reaction of a furan, increasing the thermodynamic stability of the cycloaddition product.

## Conclusions

We have demonstrated that 3-alkoxyfurans are excellent dienes for [4+2] cycloadditions with a wide variety of maleimides and other dienophiles. This methodology significantly expands the nature of cantharimides that can be readily prepared with high *endo* selectivity. The reaction tolerates alkyl, aryl and heteroaryl substituents and the enol ether cycloaddition product can be transformed into a diverse collection of drug-like compounds. Finally, DFT calculations have confirmed that a 3-alkoxy group has a significant effect on both the thermodynamic driving-force and the activation energy of the Diels–Alder reaction of 2-substituted furans with *N*-methylmaleimides. The former effect can potentially be attributed to the 3-alkoxygroup leading to a reduced energetic penalty associated with the loss of furan aromaticity that occurs during the cycloaddition reaction.

## Experimental Section

### General cycloaddition procedure

A solution of the maleimide (1.2 equiv) in dimethyl carbonate (3.6 m) was added to a stirring solution of 3-alkoxyfuran (1.0 equiv) in dimethyl carbonate (1.5 m) at room temperature and the reaction stirred at room temperature for 4–24 h. The reaction was then diluted with ethyl acetate and loaded onto an aminopropyl cartridge. After 5 min the cartridge was then flushed with ethyl acetate and the solvent removed in vacuo to give the crude cycloaddition product.

Experimental procedures, ^1^H and ^13^C NMR spectra, characterization data of all compounds, compound screening data, details of computational studies including energy minimized geometries and XRD crystallography files are available in the Supporting Information.
