# Supplementary material for: Irreversible endo-Selective Diels–Alder Reactions of Substituted Alkoxyfurans: A General Synthesis of endo-Cantharimides
Source: Chemistry. 2015 Mar 10;21(16):6107–14. doi: 10.1002/chem.201406286 (PMC4406157; doi:10.1002/chem.201406286)

CHEMISTRY
A European Journal

Supporting Information

Irreversible *endo*-Selective Diels–Alder Reactions of Substituted Alkoxyfurans: A General Synthesis of *endo*-Cantharimides

Robert W. Foster,[a] Laure Benhamou,[a] Michael J. Porter,[a] Dejan-Krešimir Bučar,[a] Helen C. Hailes,[a] Christopher J. Tame,*[b] and Tom D. Sheppard*[a]

chem_201406286_sm_miscellaneous_information.pdf

Table of Contents

1 General Experimental Details 2
2 General Experimental Procedures 3
3 Compound Synthesis: Experimental Details & Compound Characterisation 4
4 Biological Testing 36
5 Crystallography Data 37
6 DFT Study 39
7 References 103
8 NMR Spectra 104

1 General Experimental Details

All solvents and chemicals were used as obtained from commercial suppliers. Column chromatography was carried out using BDH (40-63 μm) silica gel and analytical thin layer chromatography was carried out using Merck Kieselgel aluminium-backed plates coated with silica gel. Components were visualised using combinations of UV (254 nm) and potassium permanganate. Infrared (IR) spectra were recorded on a Perkin-Elmer spectrum 100 FT-IR spectrometer as thin films. ^{1}H and ^{13}C NMR spectra were recorded respectively at 400 MHz and 100 MHz on a Bruker Avance 400 spectrometer, 500 MHz and 125 MHz in a Bruker Avance 500 or at 600 MHz and 150 MHz on a Bruker Avance 600 spectrometer in the stated solvent. Mass spectra were obtained using either a VG70-SE or MAT 900XP spectrometer at the Department of Chemistry, University College London.

2 General Experimental Procedures

General Alkynylation Procedure: Addition of 3,3-diethoxyprop-1-yne to Aldehydes

According to the procedure of Sheppard *et al.*[1]: A solution of *n*-butyl lithium (1.1 equivalents, 1.6 M in hexanes) was added dropwise to a stirring solution of 3,3-diethoxyprop-1-yne (1.2 equivalents) in dry THF (2.0 mL/mmol of aldehyde) at -78 °C. The reaction mixture was stirred at -78 °C for 1 h before the aldehyde (1.0 equivalents; neat unless otherwise indicated) was added dropwise. The reaction was stirred for 16 h, with the reaction allowed to reach room temperature. The reaction was then cooled to 0 °C and was quenched by dropwise addition of aq. sat. NH_4Cl (20 mL). The reaction mixture was diluted with ethyl acetate (20 mL) and the aqueous extract washed with ethyl acetate (3 × 20 mL). The combined organic extracts were dried (phase separator) and the solvent removed *in vacuo* to give the crude product.

General Furan Procedure: Gold(I)-Catalysed Cyclization of Propargylic Alcohols

According to the modified Procedure of Sheppard *et al.*[1]: A solution of [Bis(trifluoromethanesulfonyl)imidate](triphenylphosphine)gold(I) (2:1) toluene adduct (1 mol%, 2mol% [Au]) in ethanol (50% of total volume) was added dropwise to a stirring solution of propargylic alcohol **1** in ethanol (50% of total volume) at room temperature to give a solution of the stated concentration. The resulting solution was stirred for 16 h before being purified by flash column chromatography (aminopropyl column; 0 to 10% petrol 30–40 °C: TBME) to give the indicated 3-alkoxy furan.

General Cycloaddition Procedure: Catalyst-Free [4+2]-Cycloaddition

A solution of dienophile (1.2 equivalents) and 3-alkoxy furan (1.0 equivalents) in dimethylcarbonate (1.0 M with respect to the 3-alkoxy furan) were stirred at room temperature (unless otherwise indicated) until the reaction was judged to be complete by TLC or LC-MS analysis. The reaction was then diluted with ethyl acetate and loaded onto an aminopropyl cartridge. After 5 minutes the cartridge was then flushed with ethyl acetate and the solvent removed *in vacuo* to give the indicated product.

3 Compound Synthesis: Experimental Details & Compound Characterisation

6,6-Diethoxy-1-phenylhex-4-yn-3-ol 1a

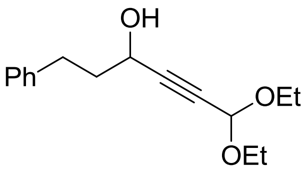

Prepared from 3-phenylpropanal (8.8 ml, 9.0 g, 90% purity by weight, 60 mmol) according to the General Alkynylation Procedure and purified by flash column chromatography (4:1 petrol 60–80 °C:ethyl acetate) to give 6,6-diethoxy-1-phenylhex-4-yn-3-ol **1a** as a colourless oil (12.8 g, 48.8 mmol, 81%); R_f = 0.33 (4:1 petrol 60–80 °C:ethyl acetate); ν_{max} (film/cm^{-1}) 3426s (O-H), 2976s (C-H), 1454s; ^{1}H NMR (500 MHz, $CDCl_3$); 7.30–7.25 (2H, m, Ar*H*), 7.21–7.18 (3H, m, Ar*H*), 5.31 (1H, s, C*H*(OEt)$_2$), 4.45–4.40 (1H, m, C*H*OH), 3.78–3.71 (2H, m, C(OC*H*H')$_2$), 3.68–3.57 (2H, m, C(OCH*H*')$_2$), 2.80 (2H, t, *J* = 7.8, PhC*H*$_2$), 2.08–2.01 (2H, m, C*H*$_2$COH), 1.89 (1H, d, *J* = 5.1, O*H*), 1.24 (6H, t, *J* = 7.1, C*H*$_3$); ^{13}C NMR (125 MHz, $CDCl_3$) 141.3 (*Ar*), 128.6 (*Ar*), 128.5 (*Ar*), 126.1 (*Ar*), 91.4 (*C*(OEt)$_2$), 86.7 (*C*≡C), 80.3 (*C*≡C), 61.3 (*C*OH), 61.1 (O*C*H$_2$), 61.0 (O*C*H$_2$), 39.1 (Ph*C*H$_2$), 31.5 (*C*H$_2$COH), 15.2 (CH$_2$*C*H$_3$); data in accordance with the literature.[1]

1-Cyclohexyl-4,4-diethoxybut-2-yn-1-ol 1c

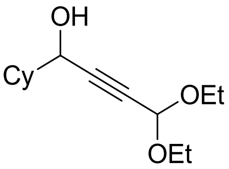

Prepared from cyclohexanecarbaldehyde (2.0 mL, 1.3 g, 14.0 mmol) according to the General Alkynylation Procedure and purified by flash column chromatography (0 to 50% cyclohexane:ethyl acetate) to give 1-cyclohexyl-4,4-diethoxybut-2-yn-1-ol **1c** as a colourless oil (2.39 g, 9.94 mmol, 86%); R_f = 0.29 (10:1 petrol 60–80 °C:ethyl acetate); ν_{max} (film/cm^{-1}) 3416s br. (O-H), 2925s (C-H), 1450s; ^{1}H NMR (600 MHz, $CDCl_3$) 5.28 (1H, s, C*H*(OEt)$_2$), 4.18 (1H, d, *J* = 6.0, C*H*OH), 3.74–3.68 (2H, m, OC*H*H'), 3.59–3.53 (2H, m, OCH*H*'), 2.20 (br. s, CHO*H*), 1.89–1.79 (2H, m, CHC*H*H'), 1.76–1.72 (2H, m, CHCH$_2$C*H*H'), 1.67–1.62 (1H, m, CHCH$_2$CH$_2$C*H*H'), 1.57–1.51 (1H, m, C*H*CH$_2$), 1.26–1.18 (8H, m, C*H*$_3$; CHCH$_2$CH*H*'), 1.16–0.99 (3H, m, CHCH*H*'; CHCH$_2$CH$_2$CH*H*'); ^{13}C NMR (150 MHz, $CDCl_3$) 91.3 (*C*H(OEt)$_2$), 85.7 (*C*≡C), 80.9 (*C*≡C), 67.0 (*C*HOH), 61.0 (O*C*H$_2$), 60.9 (O*C*H$_2$), 43.9 (*C*HCH$_2$), 28.6 (CH*C*H$_2$), 28.2 (CH*C*H$_2$), 26.4 (CHCH$_2$CH$_2$*C*H$_2$), 25.9 (CHCH$_2$*C*H$_2$), 25.9 (CHCH$_2$*C*H$_2$), 15.2 (CH$_2$*C*H$_3$); data in accordance with the literature.[1]

1-Cyclopropyl-4,4-diethoxybut-2-yn-1-ol 1d

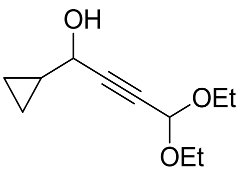

Prepared from cyclopropanecarbaldehyde (0.90 mL, 0.84 g, 12 mmol) according to the General Alkynylation Procedure and purified by flash column chromatography (0 to 50% cyclohexane:ethyl acetate) to give 1-cyclopropyl-4,4-diethoxybut-2-yn-1-ol **1d** as a colourless oil (1.97 g, 9.94 mmol, 83%); R_f = 0.29 (3:1

cyclohexane:ethyl acetate); ν_{max} (film/cm^{-1}) 3403s br. (O-H), 2977s (C-H), 1444m; ^{1}H NMR (400 MHz, $CDCl_3$) 5.32–5.29 (1H, m, C*H*(OEt)$_2$), 4.27–4.21 (1H, m, C*H*OH), 3.78–3.69 (2H, m, OC*H*H’), 3.64–3.54 (2H, m, OCH*H*’), 2.40–1.93 (1H, m, O*H*); 1.31–1.21 (7H, m, CH_2C*H*$_3$, C*H*(CH_2)$_2$), 0.63–0.42 (4H, m, CH(C*H*$_2$)$_2$); ^{13}C NMR (100 MHz, $CDCl_3$) 89.6 (*C*H(OEt)$_2$), 82.7 (*C*≡C), 78.7 (*C*≡C), 63.9 (*C*HOH), 59.3 (O*C*H$_2$), 59.2 (O*C*H$_2$), 15.3 (CH_2*C*H$_3$), 13.4 (*C*H(CH_2)$_2$), 1.6 (CH(*C*H$_2$)$_2$); data in accordance with the literature.[1]

***tert*-Butyl 4-(5,5-diethoxy-2-hydroxypent-3-yn-1-yl)piperidine-1-carboxylate 1e**

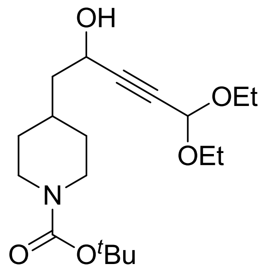

Prepared from a solution of *tert*-butyl 4-(2-oxoethyl)piperidine-1-carboxylate (1.50 g, 6.60 mmol) in dry THF (4.0 mL) according to the General Alkynylation Procedure and purified by flash column chromatography (0 to 100% heptane:TBME) to give *tert*-butyl 4-(5,5-diethoxy-2-hydroxypent-3-yn-1-yl)piperidine-1-carboxylate **1e** as a colourless oil (1.67 g, 4.70 mmol, 71%); R_f = 0.47 (1:1 petrol 60–80 °C:ethyl acetate); ν_{max} (film/cm^{-1}) 3417s br. (O-H), 2929s (C-H), 1693s (C=O), 1669s (C=O), 1424s; ^{1}H NMR (400 MHz, $CDCl_3$) 5.31 (1H, s, (*C*H(OEt)$_2$), 4.54 (1H q, *J* = 5.9 C*H*OH), 4.10 (2H, br. s, NC*H*H’), 3.79–3.71 (2H, m, OC*H*H’), 3.64–3.57 (2H, m, OCH*H*’), 2.71 (2H, br. t, NCH*H*’), 1.89 (1H, m, O*H*), 1.76–1.62 (5H, m, OCHC*H*$_2$C*H*(C*H*H’)$_2$), 1.47 (9H, s, C(C*H*$_3$)$_3$), 1.26 (6H, t, CH_2C*H*$_3$), 1.20–1.12 (2H, m, OCHCH$_2$CH(CH*H*’)$_2$); ^{13}C NMR (100 MHz, $CDCl_3$) 154.8 (*C*(O)), 91.3 (*C*H(OEt)$_2$), 86.3 (*C*≡C), 80.4 (*C*≡C), 79.3 (*C*(CH$_3$)$_3$), 61.0 (O*C*H$_2$), 60.9 (O*C*H$_2$), 60.0 (*C*HOH), 43.05 (N*C*H$_2$; OCH*C*H$_2$), 32.5 (OCHCH$_2$*C*H), 32.3 (OCHCH$_2$CH(*C*H$_2$)$_2$), 31.8 (OCHCH$_2$CH(*C*H$_2$)$_2$), 28.5 (C(*C*H$_3$)$_3$), 15.1 (CH_2*C*H$_3$); HRMS (ESI$^+$) found [M+Na]$^+$ 378.2202; $C_{19}H_{33}NO_5Na$ requires 378.2256.

4,4-Diethoxy-1-phenylbut-2-yn-1-ol 1f

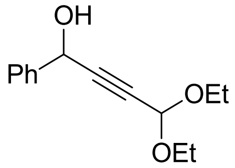

Prepared from benzaldehyde (4.9 mL, 5.0 g, 47 mmol) according to the General Alkynylation Procedure and purified by flash column chromatography (7:1 petrol 60–80 °C:ethyl acetate) to give 4,4-diethoxy-1-phenylbut-2-yn-1-ol **1f** as a colourless oil (10.5 g, 44.6 mmol, 95%); R_f = 0.33 (5:1 petrol 60–80 °C:ethyl acetate); ν_{max} (film/cm^{-1}) 3317s br. (O-H), 2977s (C-H), 1493m, 1455s; ^{1}H NMR (400 MHz, $CDCl_3$) 7.57–7.54 (2H, m, Ar*H*), 7.43–7.33 (3H, m, Ar*H*), 5.83 (1H, s, C*H*(OEt)$_2$), 5.55 (1H, d, *J* = 6.2, C*H*OH), 3.81–3.73 (2H, m, OC*H*H’), 3.67–3.58 (2H, m, OCH*H*’), 2.43 (1H, d, *J* = 6.2, O*H*), 1.25 (6H, t, *J* = 7.1, CH_2C*H*$_3$); ^{13}C NMR (125 MHz, $CDCl_3$) 140.1 (*Ar*), 128.7 (*Ar*), 128.5 (*Ar*), 126.7 (*Ar*), 91.4 (*C*H(OEt)$_2$), 85.2 (*C*≡C), 82.0 (*C*≡C), 64.5 (*C*HOH), 61.1 (O*C*H$_2$), 61.0 (O*C*H$_2$), 15.1 (CH_2*C*H$_3$); data in accordance with the literature.[1]

4,4-Diethoxy-1-(4-(trifluoromethyl)phenyl)but-2-yn-1-ol 1g

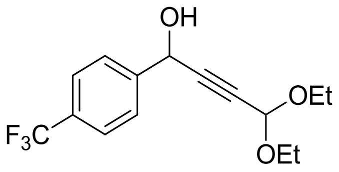

Prepared from 4-(trifluoromethyl)benzaldehyde (1.6 mL, 2.0 g, 12 mmol) according to the General Alkynylation Procedure and purified by flash column chromatography (0 to 50% cyclohexane:ethyl acetate) to give 4,4-diethoxy-1-(4-(trifluoromethyl)phenyl)but-2-yn-1-ol **1g** as a colourless oil (2.88 g, 9.94 mmol, 79%); R_f = 0.22 (3:1 cyclohexane:ethyl acetate); ν_{max} (film/cm^{-1}) 3408s br. (O-H), 2981s (C-H), 1620s, 1416s; ^{1}H NMR (400 MHz, $CDCl_3$) 7.69–7.64 (4H, m, Ar*H*), 5.60 (1H, d, *J* = 5.9, C*H*OH), 5.37 (1H, s, C*H*(OEt)$_2$), 4.27–4.21 (1H, m, C*H*OH), 3.80–3.72 (2H, m, OC*H*H’), 3.66–3.58 (2H, m, OCH*H*’), 2.66–1.63 (1H, m, CO*H*), 1,26 (6H, t, *J* = 7.0, CH_2CH_3); ^{13}C NMR (100 MHz, $CDCl_3$) 143.7 (*Ar*), 130.6 (q, *J* = 30.0, *Ar*), 126.9 (*Ar*), 125.6 (q, *J* = 3.5, *Ar*), 124.0 (q, *J* = 272.2, *C*F_3), 91.2 (*C*H(OEt)$_2$), 84.2 (*C*≡C), 82.6 (*C*≡C), 63.7 (*C*HOH), 61.1 (O*C*H$_2$), 61.1 (O*C*H$_2$), 15.0 (CH_2CH_3); data in accordance with the literature.[1]

1-(4-Bromophenyl)-4,4-diethoxybut-2-yn-1-ol 1h

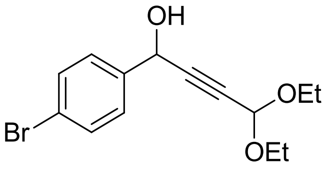

Prepared from a solution of 4-bromobenzaldehyde (2.15 g, 11.9 mmol) in dry THF (12 mL) according to the General Alkynylation Procedure and purified by flash column chromatography (0 to 100% cyclohexane:ethyl acetate) to give 1-(4-bromophenyl)-4,4-diethoxybut-2-yn-1-ol **1h** as a colourless oil (3.01 g, 9.61 mmol, 83%); R_f = 0.69 (1:1 cyclohexane:ethyl acetate); ν_{max} (film/cm^{-1}) 3410s br. (O-H), 2978s (C-H), 1486s; ^{1}H NMR (400 MHz, $CDCl_3$) 7.55–7.51 (2H, m, Ar*H*), 7.45–7.41 (2H, m, Ar*H*), 5.51 (1H, d, *J* = 6.0, C*H*OH), 5.36 (1H, s, C*H*(OEt)$_2$), 3.80–3.71 (2H, m, OC*H*H’), 3.66–3.58 (2H, m, OCH*H*’), 2.40 (1H, d, *J* = 6.0, CO*H*), 1.26 (3H, t, *J* = 7.1, CH_2CH_3), 1.26 (3H, t, *J* = 7.1, CH_2CH_3); ^{13}C NMR (100 MHz, $CDCl_3$) 138.4 (*Ar*), 131.7 (*Ar*), 128.3 (*Ar*), 122.5 (*Ar*), 91.3 (*C*H(OEt)$_2$), 84.4 (*C*≡C), 82.4 (*C*≡C), 63.8 (*C*HOH), 61.1 (O*C*H$_2$), 61.0 (O*C*H$_2$), 15.1 (CH_2CH_3); data in accordance with the literature.[1]

4,4-Diethoxy-1-(4-methoxyphenyl)but-2-yn-1-ol 1i

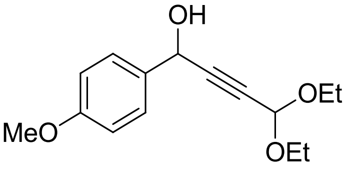

Prepared from 4-methoxybenzaldehyde (1.4 mL, 1.6 g, 11 mmol) according to the General Alkynylation Procedure and purified by flash column chromatography (0 to 50% cyclohexane:ethyl acetate) to give 4,4-diethoxy-1-(4-methoxyphenyl)but-2-yn-1-ol **1i** as a yellow oil (2.92 g, 11.1 mmol, 96%); R_f = 0.18 (3:1 cyclohexane:ethyl acetate); ν_{max} (film/cm^{-1}) 3423s br. (O-H), 2976s (C-H), 1611s, 1511s; ^{1}H NMR (400 MHz, DMSO-d$_6$) 7.43–733 (2H, m, Ar*H*), 6.96–9.88 (2H, m, Ar*H*), 5.97 (1H, br. s, CHO*H*), 5.37 (1H, br. s, C*H*OH), 5.37 (1H, s, C*H*(OEt)$_2$), 3.75 (3H, s, OC*H*$_3$), 3.67–3.56 (2H, m, OC*H*H’), 3.55–3.45 (2H, m, OCH*H*’), 1.13 (6H, t, *J* = 7.1, CH_2CH_3); ^{13}C NMR (100 MHz, DMSO-d$_6$) 159.2 (*Ar*), 134.3 (*Ar*), 128.1 (*Ar*), 114.1 (*Ar*), 91.2 (*C*H(OEt)$_2$), 87.2 (*C*≡C), 80.7 (*C*≡C), 62.3 (*C*HOH), 60.6 (O*C*H$_2$), 55.6 (O*C*H$_3$), 15.4 (CH_2CH_3); data in accordance with the literature.[1]

Methyl 3-(4,4-diethoxy-1-hydroxybut-2-yn-1-yl)benzoate 1j

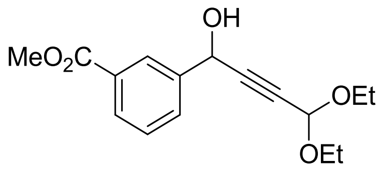

Prepared from a solution of methyl 3-formylbenzoate (1.31 g, 7.98 mmol) in dry THF (8 mL) according to the General Alkynylation Procedure and purified by flash column chromatography (0 to 100% TBME:cyclohexane) to give methyl 3-(4,4-diethoxy-1-hydroxybut-2-yn-1-yl)benzoate **1j** as a colourless oil (1.83 g, 6.26 mmol, 78%); R_f = 0.71 (3:1 cyclohexane:ethyl acetate); ν_{max} (film/cm^{-1}) 3433s br. (O-H), 2887s (C-H), 1721s (C=O), 1481s; ^{1}H NMR (400 MHz, MeOH-d_4) 8.22 (1H, s, Ar*H*), 7.98 (1H, d, *J* = 7.8, Ar*H*), 7.77 (1H, d, *J* = 7.8, Ar*H*), 7.51 (1H, t, *J* = 7.8, Ar*H*), 5.56 (1H, s, C*H*OH), 5.37 (1H, s, C*H*(OEt)$_2$), 3.93 (3H, s, OC*H*$_3$), 3.80–3.70 (2H, m, OC*H*H’), 3.67–3.56 (2H, m, OCH*H*’), 1.21 (6H, t, *J* = 7.1 CH$_2$C*H*$_3$); ^{13}C NMR (100 MHz, MeOH-d_4) 166.8 (*C*(O)), 141.8 (*Ar*), 131.0 (*Ar*), 130.2 (*Ar*), 128.8 (*Ar*), 128.4 (*Ar*), 127.3 (*Ar*), 91.3 (*C*H(OEt)$_2$), 85.0 (*C*≡C), 81.1 (*C*≡C), 62.8 (*C*HOH), 60.7 (O*C*H$_2$), 60.7 (O*C*H$_2$), 51.3 (O*C*H$_3$), 14.0 (CH$_2$*C*H$_3$); data in accordance with the literature.[1]

4,4-Diethoxy-1-(*o*-tolyl)but-2-yn-1-ol 1k

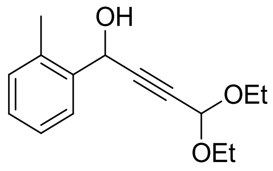

Prepared from 2-methylbenzaldehyde (1.3 mL, 1.4 g, 11 mmol) according to the General Alkynylation Procedure and purified by flash column chromatography (0 to 100% cyclohexane:ethyl acetate) to give 4,4-diethoxy-1-(*o*-tolyl)but-2-yn-1-ol **1k** as a colourless oil (2.58 g, 10.4 mmol, 92%); R_f = 0.24 (3:1 cyclohexane:ethyl acetate); ν_{max} (film/cm^{-1}) 3422s br. (O-H), 2977s (C-H), 1487s, 1460s; ^{1}H NMR (400 MHz, $CDCl_3$) 7.68–7.63 (1H, m, Ar*H*), 7.28–7.18 (3H, m, Ar*H*), 5.70 (1H, dd, *J* = 6.0, 1.2, C*H*OH), 5.37 (1H, d, *J* = 1.2, C*H*(OEt)$_2$), 3.81–3.72 (2H, m, OC*H*H’), 3.67–3.58 (2H, m, OCH*H*’), 2.46 (3H, s, ArC*H*$_3$), 2.20 (1H, d, *J* = 6.0, O*H*), 1.26 (3H, t, *J* = 7.1, CH$_2$C*H*$_3$), 1.25 (3H, t, *J* = 7.1, CH$_2$C*H*$_3$); ^{13}C NMR (100 MHz, $CDCl_3$) 137.0 (*Ar*), 136.0 (*Ar*), 130.8 (*Ar*), 128.6 (*Ar*), 126.6 (*Ar*), 126.2 (*Ar*), 91.4 (*C*H(OEt)$_2$), 84.7 (*C*≡C), 82.0 (*C*≡C), 62.3 (*C*HOH), 61.0 (O*C*H$_2$), 61.0 (O*C*H$_2$), 18.9 (ArCH$_3$), 15.1 (CH$_2$*C*H$_3$); HRMS (CI$^+$) found $[M+H]^+$ 248.1409; $C_{15}H_{20}O_3$ requires 248.1412.

4,4-Diethoxy-1-(furan-2-yl)but-2-yn-1-ol 1l

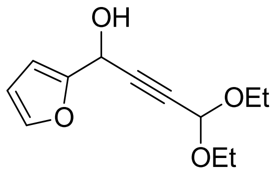

Prepared from furfural (0.96 mL, 1.1 g, 12 mmol) according to the General Alkynylation Procedure and purified by flash column chromatography (0 to 50% cyclohexane:ethyl acetate) to give 4,4-diethoxy-1-(furan-2-yl)but-2-yn-1-ol **1l** as a yellow oil (2.23 g, 9.96 mmol, 86%); R_f = 0.30 (3:1 cyclohexane:ethyl acetate); ν_{max} (film/cm^{-1}) 3411s br. (O-H), 2978s (C-H); ^{1}H NMR (400 MHz, $CDCl_3$) 7.43–7.42 (1H, m, Ar*H*), 6.47 (1H, d, *J* = 3.2, Ar*H*), 6.38–6.36 (1H, m, Ar*H*), 5.53 (1H, d, *J* = 6.9, C*H*OH), 5.37–5.36 (1H, m, C*H*(OEt)$_2$), 3.82–3.73 (2H, m, OC*H*H’), 3.68–3.60 (2H, m, OCH*H*’), 2.69–1.50 (1H, m, O*H*), 1.25 (6H, t, *J* = 6.8, C*H*$_3$); ^{13}C NMR (100 MHz, $CDCl_3$) 152.4 (*Ar*), 143.1 (*Ar*), 110.4 (*Ar*), 108.0 (*Ar*), 91.2 (*C*H(OEt)$_2$), 82.6 (*C*≡C), 81.2 (*C*≡C), 61.1 (O*C*H$_2$), 61.0 (O*C*H$_2$), 58.0 (*C*HOH), 15.0 (CH$_2$*C*H$_3$); data in accordance with the literature.[1]

4,4-Diethoxy-1-(thiophen-2-yl)but-2-yn-1-ol 1m

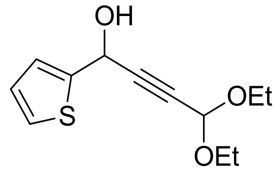

Prepared from thiophene-2-carbaldehyde (1.6 ml, 1.9 g, 17 mmol) according to the General Alkynylation Procedure and purified by flash column chromatography (0 to 100% cyclohexane:TBME) to give 4,4-diethoxy-1-(thiophen-2-yl)but-2-yn-1-ol **1m** as a yellow oil (2.00 g, 8.32 mmol, 49%); R_f = 0.61 (1:1 cyclohexane:ethyl acetate); ν_{max} (film/cm^{-1}) 3403s br. (O-H), 2926s (C-H); ^{1}H NMR (400 MHz, $CDCl_3$) 7.33–7.31 (1H, m Ar*H*), 7.21–7.17 (1H, m, Ar*H*), 7.00–6.98 (1H, m Ar*H*), 5.74 (1H, d, *J* = 6.9, C*H*OH), 5.37 (1H, s, C*H*(OEt)$_2$), 3.83–3.74 (2H, m, OC*H*H'), 3.67–3.58 (2H, m, OCH*H*'), 2.69 (1H, d, *J* = 6.9, O*H*), 1.26 (6H, t, *J* = 7.1, CH$_2$C*H*$_3$); ^{13}C NMR (100 MHz, $CDCl_3$) 143.9 (*Ar*), 126.8 (*Ar*), 126.2 (*Ar*), 125.7 (Ar), 91.2 (*C*H(OEt)$_2$), 84.3 (*C*≡C), 81.4 (*C*≡C), 61.1 (O*C*H$_2$), 61.0 (O*C*H$_2$), 60.1 (*C*HOH), 15.1 (CH$_2$*C*H$_3$); data in accordance with the literature.[1]

4,4-Diethoxy-1-(pyridin-3-yl)but-2-yn-1-ol 1n

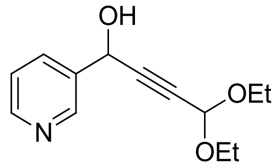

Prepared from nicotinaldehyde (1.1 mL, 1.3 g, 12 mmol) according to the General Alkynylation Procedure and purified by flash column chromatography (0 to 100% cyclohexane: ethyl acetate) to give 4,4-diethoxy-1-(pyridin-3-yl)but-2-yn-1-ol **1n** as a yellow oil (1.93 g, 8.20 mmol, 70%); R_f = 0.40 (40:1 cyclohexane:ethyl acetate); ν_{max} (film/cm^{-1}) 3160s br. (O-H), 2977s (C-H), 1427s; ^{1}H NMR (400 MHz, DMSO-d$_6$) 8.65 (1H, d, *J* = 1.8, Ar*H*), 8.53 (1H, dd, *J* = 4.9, 1.8, Ar*H*), 7.84 (1H, dt, 7.8, 1.8, Ar*H*), 7.42 (1H, dd, *J* = 7.8, 4.9, Ar*H*), 6.31 (1H, d, *J* = 6.1, CHO*H*), 5.55 (1H, d, *J* = 6.1, C*H*OH), 5.37 (1H, s, C*H*(OEt)$_2$), 3.66–3.58 (2H, m, OC*H*H'), 3.55–3.47 (2H, m, OCH*H*'), 1.13 (6H, t, *J* = 7.1, CH$_2$C*H*$_3$); ^{13}C NMR (100 MHz, DMSO-d$_6$) 148.9 (*Ar*), 147.8 (*Ar*), 137.1 (*Ar*), 134.0 (*Ar*), 123.5 (*Ar*), 90.7 (*C*H(OEt)$_2$), 85.6 (*C*≡C), 81.0 (*C*≡C), 60.8 (*C*HOH), 60.3 (O*C*H$_2$), 60.2 (O*C*H$_2$), 14.9 (CH$_2$*C*H$_3$); HRMS (CI$^+$) found [M+H]$^+$ 236.1281; $C_{15}H_{20}O_3$ requires 236.1287.

3-Ethoxy-2-phenethylfuran 2a

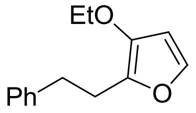

Prepared according to the General Furan Procedure from 6,6-diethoxy-1-phenylhex-4-yn-3-ol **1a** (500 mg, 1.91 mmol) in ethanol (0.5 M) to give 3-ethoxy-2-phenethylfuran **2a** as a colourless oil (737 mg, 3.41 mmol, 45%); R_f = 0.72 (10:1 cyclohexane:ethyl acetate); ν_{max} (film/cm^{-1}) 2927s (C-H), 1635s, 1495s, 1453s, 1420s; ^{1}H NMR (400 MHz, MeOH-d$_4$) 7.27–7.09 (6H, m, Ar*H*), 6.28 (1H, d, *J* = 2.0, Ar*H*), 3.74 (2H, q, *J* = 7.1, OC*H*$_2$), 2.93–2.81 (4H, m, C*H*$_2$C*H*$_2$Ph), 1.19 (3H, t, *J* = 7.1, CH$_2$C*H*$_3$); ^{13}C NMR (125 MHz, $CDCl_3$) 142.4 (*Ar*), 141.3 (*Ar*), 139.3 (*Ar*), 139.0 (*Ar*), 128.1 (*Ar*), 127.8 (*Ar*), 125.5 (*Ar*), 103.6 (*Ar*), 67.4 (O*C*H$_2$), 33.9 (*C*H$_2$), 26.6 (*C*H$_2$), 14.0 (CH$_2$*C*H$_3$);. data in accordance with the literature.[1]

3-Methoxy-2-phenethylfuran 2b

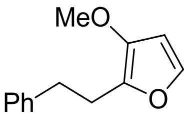

A solution of [Bis(trifluoromethanesulfonyl)imidate](triphenylphosphine)gold(I) (2:1) toluene adduct (30 mg, 0.019 mmol, 1 mol%, 2mol% [Au]) in methanol (4.8 mL) was added dropwise to a stirring solution of 6,6-diethoxy-1-phenylhex-4-yn-3-ol **1a** (500 mg, 1.91 mmol) in methanol (4.8 mL) at room temperature. The resulting solution was stirred for 16 h before being filtered through a silica plug, eluting with TBME. The eluent was concentrated under vacuum to give the crude product, which was purified by flash column chromatography (aminopropyl column; 0 to 10% petrol 30–40 °C: TBME) to give 3-methoxy-2-phenethylfuran **2b** as a colourless oil (266 mg, 1.32 mmol, 69%); R_f = 0.29 (40:1 petrol 60–80 °C:ethyl acetate); ν_{max} (film/cm^{-1}) 2935s (C-H), 1637s, 1496s, 1454s 1410s; ^{1}H NMR (600 MHz, $CDCl_3$) 7.28–7.26 (2H, m, Ar*H*), 7.20–7.16 (3H, m, Ar*H*), 7.14 (1H, d, *J* = 2.1, *Ar*), 6.26 (1H, d, *J* = 2.1, *Ar*), 3.59 (3H, s, OCH_3), 2.94–2.86 (4H, m, PhCH_2CH_2); ^{13}C NMR (150 MHz, $CDCl_3$) 143.7 (*Ar*), 141.6 (*Ar*), 139.2 (*Ar*), 139.2 (*Ar*), 128.6 (*Ar*), 128.4 (*Ar*), 126.0 (*Ar*), 103.3 (*Ar*), 59.6 (O*C*H_3), 34.4 (*C*H_2) 27.2 (*C*H_2); data in accordance with the literature.[1]

2-Cyclohexyl-3-ethoxyfuran 2c

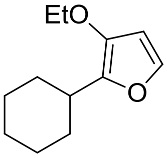

Prepared according to the General Furan Procedure from 1-cyclohexyl-4,4-diethoxybut-2-yn-1-ol **1c** (0.500 g, 2.08 mmol) in ethanol (2.0 M) to give 3-cyclohexyl-2-phenylfuran **2c** as a colourless oil (273 mg, 1.41 mmol, 68%); R_f = 0.33 (40:1 petrol 60–80 °C:ethyl acetate); ν_{max} (film/cm^{-1}) 2927s (C-H), 1627s; ^{1}H NMR (600 MHz, $CDCl_3$) 7.09 (1H, s, Ar*H*), 7.08 (1H, s, ArC*H*), 3.91 (2H, q, *J* = 7.0, OCH_2), 2.70 (1H, tt, *J* = 11.7, 3.2, ArC*H*), 1.84–1.76 (4H, m, CHC*H*H'; CHCH_2C*H*H'), 1.71–1.66 (1H, m, C*H*H'CH_2CH_2CH), 1.58–1.51 (2H, m, CHCH*H'*), 1.37–1.29 (5H, m, CH_3; CHCH_2CH*H'*), 1.28–1.23 (1H, m, CH*H'*CH_2CH_2CH); ^{13}C NMR (150 MHz, $CDCl_3$) 145.0 (*Ar*), 140.6 (*Ar*), 138.6 (Ar), 140.2 (Ar), 68.1 (O*C*H_2), 35.3 (*C*HCH_2), 31.3 (CH*C*H_2), 26.5 (CHCH_2*C*H_2), 26.1 (CHCH_2CH_2*C*H_2), 15.4 (CH_2*C*H_3); HRMS (CI^+) found $[M+H]^+$ 195.1389; $C_{12}H_{19}O_2$ requires 195.1385.

2-Cyclopropyl-3-ethoxyfuran 2d

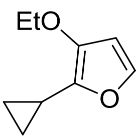

Prepared according to the General Furan Procedure from 1-cyclopropyl-4,4-diethoxybut-2-yn-1-ol **1d** (250 mg, 1.26 mmol) in ethanol (2.0 M) to give 2-cyclopropyl-3-ethoxyfuran **2d** as a colourless oil (56 mg, 0.368 mmol, 29%); R_f = 0.78 (10:1 cyclohexane:ethyl acetate); ν_{max} (film/cm^{-1}) 2991s (C-H), 1666s, 1600s, 1432s; ^{1}H NMR (400 MHz, DMSO-d_6) 7.24 (1H, d, *J* = 2.0, Ar*H*), 6.41 (1H, d, *J* = 2.0, Ar*H*), 3.91 (2H, q, *J* = 7.1, CH_2CH_3), 1.86–1.79 (1H, m, C*H*(CH_2)), 1.24 (3H, t, *J* = 7.1, CH_3), 0.84–0.80 (2H, m, CH(C*H*H')), 0.73–0.69 (2H, m, CH(CH*H'*)); ^{13}C NMR (100 MHz, DMSO-d_6) 142.0 (*Ar*), 139.1 (*Ar*), 128.6 (*Ar*), 104.5 (*Ar*), 66.8 (O*C*H_2), 14.9 (CH_2*C*H_3), 6.3 (*C*H(CH_2)$_2$), 5.3 (CH(*C*H_2)$_2$); data in accordance with the literature.[1]

tert-Butyl 4-((3-ethoxyfuran-2-yl)methyl)piperidine-1-carboxylate 2e

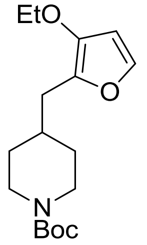

Prepared according to the General Furan Procedure from (*tert*-butyl 4-(5,5-diethoxy-2-hydroxypent-3-yn-1-yl)piperidine-1-carboxylate **1e** (500 mg, 1.41 mmol) in ethanol (1.0 M) to give *tert*-butyl 4-((3-ethoxyfuran-2-yl)methyl)piperidine-1-carboxylate **2e** as a colourless oil (335 mg, 1.08 mmol, 77%); R_f = 0.34 (10:1 cyclohexane:ethyl acetate); ν_{max} (film/cm^{-1}) 2930s (C-H), 1693s (C=O), 1422m; ^{1}H NMR (400 MHz, DMSO-d$_6$) 7.20 (1H, d, *J* = 2.0, Ar*H*), 6.35 (1H, d, *J* = 2.0, Ar*H*), 4.07–4.01 (2H, m, NC*H*H'), 3.95 (2H, q, *J* = 7.1, C*H*$_2$CH$_3$), 2.83–2.68 (2H, m, NCH*H*'), 2.53 (2H, d, *J* = 6.9, ArC*H*$_2$), 1.86–1.75 (1H, m, ArCH$_2$C*H*), 1.67–1.61 (2H, m, CH(C*H*H')$_2$), 1.47 (9H, s, C(C*H*$_3$)$_3$), 1.31 (3H, t, *J* = 7.1, CH$_2$C*H*$_3$), 1.19–1.07 (2H, m, CH(CH*H*')$_2$); ^{13}C NMR (100 MHz, DMSO-d$_6$) 156.6 (*C*(O)), 144.4 (*Ar*), 140.6 (*Ar*), 139.7 (*Ar*), 104.6 (*Ar*), 80.9 (*C*Me$_3$), 68.6 (*C*H$_2$CH$_3$), 36.9 (ArCH$_2$*C*H), 33.0 (CH(*C*H$_2$)$_2$), 32.5 (Ar*C*H$_2$), 28.7 (C(*C*H$_3$)$_3$), 15.5 (CH$_2$*C*H$_3$); HRMS (CI$^+$) found [M+H]$^+$ 310.2012; $C_{17}H_{27}NO_4$ requires 310.2018.

3-Ethoxy-2-phenylfuran 2f

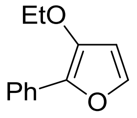

Prepared according to the General Furan Procedure from 4,4-diethoxy-1-phenylbut-2-yn-1-ol **1f** (2.00 g, 8.54 mmol) in ethanol (2.0 M) to give 3-ethoxy-2-phenylfuran **2f** as a colourless oil (910 mg, 4.83 mmol, 57%); R_f = 0.33 (40:1 petrol 60–80 °C:ethyl acetate); ν_{max} (film/cm^{-1}) 2980s (C-H), 1612s, 1510s, 1427s; ^{1}H NMR (600 MHz, CDCl$_3$) 7.81 (2H, d, *J* = 7.7, Ar*H*), 7.37 (2H, t, *J* = 7.7, Ar*H*), 7.27 (1H, d, *J* = 1.6, Ar*H*), 7.17 (1H, t, *J* = 7.7, Ar*H*), 6.40 (1H, d, *J* = 1.6, Ar*H*), 4.10 (2H, q, *J* = 7.0, OC*H*$_2$), 1.44 (3H, t, *J* = 7.0, C*H*$_3$); ^{13}C NMR (150 MHz, CDCl$_3$) 144.4 (*Ar*), 140.2 (Ar), 136.9 (*Ar*), 131.1 (*Ar*), 128.5 (*Ar*), 125.8 (*Ar*), 123.1 (*Ar*), 104.1 (*Ar*), 67.3 (O*C*H$_2$), 15.4 (CH$_2$*C*H$_3$); data in accordance with the literature.[1]

3-Ethoxy-2-(4-(trifluoromethyl)phenyl)furan 2g

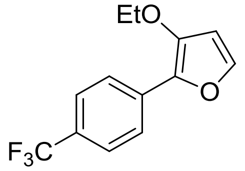

Prepared according to the General Furan Procedure from 4,4-diethoxy-1-(4-(trifluoromethyl)phenyl)but-2-yn-1-ol **1g** (500 mg, 1.65 mmol) in ethanol (2.0 M) to give 3-ethoxy-2-(4-(trifluoromethyl)phenyl)furan **2g** as a colourless oil (357 mg, 1.39 mmol, 84%); R_f = 0.50 (10:1 cyclohexane:ethyl acetate); ν_{max} (film/cm^{-1}) 2985s (C-H), 1614s; ^{1}H NMR (600 MHz, MeOH-d$_4$) 7.92 (2H, d, *J* = 8.3, Ar*H*), 7.34 (2H, d, *J* = 8.3, Ar*H*), 7.47 (1H, d, *J* = 2.1, Ar*H*), 6.62 (1H, d, *J* = 2.1, Ar*H*), 4.19 (2H, q, *J* = 7.1, C*H*$_2$CH$_3$), 1.46 (3H, t, *J* = 7.1, CH$_2$C*H*$_3$); ^{13}C NMR (150 MHz, MeOH-d$_4$) 146.5 (*Ar*), 141.7 (*Ar*), 134.8 (*Ar*), 134.4 (q, *J* = 1.3, *Ar*), 126.5 (q, *J* = 32.3, *Ar*), 124.9 (q, *J* = 3.9, *Ar*), 124.5 (q, *J* = 271.0, *C*F$_3$), 122.3 (*Ar*), 103.6 (*Ar*), 66.9 (O*C*H$_2$), 14.0 (CH$_2$*C*H$_3$); data in accordance with the literature.[1]

2-(4-Bromophenyl)-3-ethoxyfuran 2h

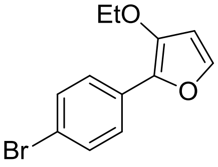

Prepared according to the General Furan Procedure from 4,4-diethoxy-1-(4-bromophenyl)but-2-yn-1-ol **1h** (500 mg, 1.60 mmol) in ethanol (2.0 M) to give 3-ethoxy-2-(4-methoxyphenyl)furan **2h** as a colourless oil (334 mg, 1.25 mmol, 78%); R$_f$ = 0.57 (10:1 cyclohexane:ethyl acetate); ν_{max} (film/cm^{-1}) 2980s (C-H), 1669m, 1612s, 1504s, 1431s; ^{1}H NMR (400 MHz, DMSO-d$_6$) 7.67–7.65 (5H, m, Ar*H*), 6.75 (1H, d, *J* = 2.0, Ar*H*), 4.10 (2H, q, *J* = 6.8, OC*H*$_2$), 1.35 (3H, t, *J* = 6.8, CH$_2$C*H*$_3$); ^{13}C NMR (100 MHz, DMSO-d$_6$) 145.1 (*Ar*), 141.9 (*Ar*), 134.2 (*Ar*), 131.6 (*Ar*), 129.6 (*Ar*), 124.0 (*Ar*), 118.1 (*Ar*), 104.8 (*Ar*), 66.8 (O*C*H$_2$), 14.9 (CH$_2$*C*H$_3$); HRMS (CI$^+$) found [M+H]$^+$ 265.9942; $C_{12}H_{12}BrO_2$ requires 265.9945.

3-Ethoxy-2-(4-methoxyphenyl)furan 2i

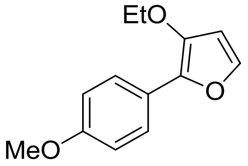

Prepared according to the General Furan Procedure from 4,4-diethoxy-1-(4-methoxyphenyl)but-2-yn-1-ol **1i** (500 mg, 1.89 mmol) in ethanol (0.5 M) to give 3-ethoxy-2-(4-methoxyphenyl)furan **2i** as a colourless oil (334 mg, 1.53 mmol, 81%); R$_f$ = 0.48 (10:1 cyclohexane:ethyl acetate); ν_{max} (film/cm^{-1}) 2980s (C-H), 1606s, 1520s, 1431s; ^{1}H NMR (400 MHz, MeOH-d$_4$) 7.72–7.69 (2H, m, Ar*H*), 7.30 (1H, d, *J* = 2.2, Ar*H*), 7.95–7.91 (2H, m, Ar*H*), 6.51 (1H, d, *J* = 2.2, Ar*H*), 4.08 (2H, q, *J* = 7.0, OC*H*$_2$), 3.90 (3H, s, OC*H*$_3$), 1.40 (3H, t, *J* = 7.0, CH$_2$C*H*$_3$); ^{13}C NMR (100 MHz, MeOH-d$_4$) 157.9 (*Ar*), 142.7 (*Ar*), 139.3 (*Ar*), 136.9 (*Ar*), 128.5 (*Ar*), 124.0 (*Ar*), 113.5 (*Ar*), 103.8 (*Ar*), 66.8 (O*C*H$_2$), 54.3 (O*C*H$_3$), 14.2 (CH$_2$*C*H$_3$); data in accordance with the literature.[1]

Methyl 3-(3-ethoxyfuran-2-yl)benzoate 2j

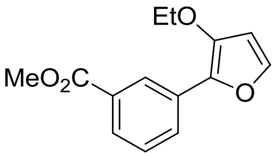

Prepared according to the General Furan Procedure from methyl 3-(4,4-diethoxy-1-hydroxybut-2-yn-1-yl)benzoate **1j** (500 mg, 1.71 mmol) in ethanol (0.5 M) to give methyl 3-(3-ethoxyfuran-2-yl)benzoate **2j** as a waxy solid (302 mg, 1.23 mmol, 72%); R$_f$ = 0.47 (10:1 cyclohexane:ethyl acetate); ν_{max} (film/cm^{-1}) 2981s (C-H), 1720s (c=O), 1667s, 1431s; ^{1}H NMR (400 MHz, d$_4$-MeOH) 8.43 (1H, t, *J* = 1.4, Ar*H*), 8.00 (1H, dt, *J* = 7.8, 1.4, Ar*H*), 7.80 (1H, dt, *J* = 7.8, 1.4, Ar*H*), 7.47 (1H, t, *J* = 7.8, Ar*H*), 7.43 (1H, d, *J* = 2.1, Ar*H*), 6.61 (1H, d, *J* = 2.1, Ar*H*), 4.17 (2H, q, *J* = 7.0, OC*H*$_2$), 3.94 (3H, s, OC*H*$_3$), 1.46 (3H, t, *J* = 7.0, CH$_2$C*H*$_3$); ^{13}C NMR (100 MHz, MeOH-d$_4$) 167.2 (*C*(O)), 145.3 (*Ar*), 140.9 (*Ar*), 135.4 (*Ar*), 131.4 (*Ar*), 130.2 (*Ar*), 128.3 (*Ar*), 126.7 (*Ar*), 125.9 (*Ar*), 123.3 (*Ar*), 103.7 (*Ar*), 66.9 (*C*H$_2$CH$_3$), 51.2 (O*C*H$_3$), 14.1 (CH$_2$*C*H$_3$); HRMS (CI$^+$) found [M+H]$^+$ 247.0971; $C_{14}H_{15}O_4$ requires 247.0970.

3-Ethoxy-2-(*o*-tolyl)furan 2k

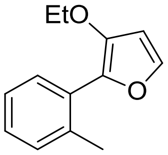

Prepared according to the General Furan Procedure from 4,4-diethoxy-1-(*o*-tolyl)but-2-yn-1-ol **1k** (500 mg, 2.01 mmol) in ethanol (2.0 M) to give 3-ethoxy-2-(*o*-tolyl)furan **2k** as a colourless oil (177 mg, 0.875 mmol, 44%); R_f = 0.64 (10:1 cyclohexane:ethyl acetate); ν_{max} (film/cm^{-1}) 2979s (C-H), 1618s; ^{1}H NMR (400 MHz, d_4-MeOH) 7.52–7.48 (1H, m, Ar*H*), 7.41 (1H, d, *J* = 2.0, Ar*H*), 7.24–7.17 (3H, m, Ar*H*), 6.53 (1H, d, *J* = 2.0, Ar*H*), 4.02 (2H, q, *J* = 7.1, CH_2CH_3), 2.39 (3H, s, ArCH_3), 1.32 (3H, t, *J* = 7.1, CH_2CH_3); ^{13}C NMR (100 MHz, d_4-MeOH) 145.1 (*Ar*), 141.7 (*Ar*), 139.2 (*Ar*), 137.0 (*Ar*), 131.6 (*Ar*), 131.3 (*Ar*), 129.5 (*Ar*), 128.4 (*Ar*), 126.4 (*Ar*), 104.9 (*Ar*), 68.2 (CH_2CH_3), 21.1 (ArCH_3), 15.5 (CH_2CH_3); HRMS (CI$^+$) found [M+H]$^+$ 203.1075; $C_{13}H_{15}O_2$ requires 203.1072.

3-Ethoxy-2,2'-bifuran 2l

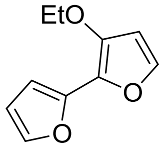

Prepared according to the General Furan Procedure from 4,4-diethoxy-1-(furan-2-yl)but-2-yn-1-ol **1l** (500 mg, 2.23 mmol) in ethanol (0.5 M) to give 3-ethoxy-2,2'-bifuran **2l** as a colourless oil (102 mg, 0.572 mmol, 26%); R_f = 0.50 (10:1 cyclohexane:ethyl acetate); ν_{max} (film/cm^{-1}); 3150m, 2981s (C-H), 1629s, 1575s, 1415s; ^{1}H NMR (400 MHz, MeOH-d_4) 7.47 (1H, d, *J* = 2.0, Ar*H*), 7.35 (1H, d, *J* = 2.2, Ar*H*), 6.53 (1H, d, *J* = 2.2, Ar*H*), 6.50 (1H, dd, *J* = 3.4, 2.0, Ar*H*), 6.45 (1H, d, *J* = 3.4, Ar*H*), 4.10 (2H, q, *J* = 6.9, OCH_2), 1.39 (3H, t, *J* = 6.9, CH_2CH_3); ^{13}C NMR (100 MHz, MeOH-d_4) 145.5 (*Ar*), 143.2 (*Ar*), 140.6 (*Ar*), 140.5 (*Ar*), 112.0 (*Ar*), 110.7 (*Ar*), 103.9 (*Ar*), 103.6 (*Ar*), 67.01 (OCH_2), 14.0 (CH_2CH_3); HRMS (CI$^+$) found [M+H]$^+$ 179.070; $C_{10}H_{10}O_3$ requires 179.0708.

3-Ethoxy-2-(thiophen-2-yl)furan 2m

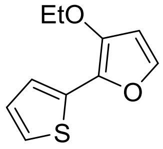

Prepared according to the General Furan Procedure from 4,4-diethoxy-1-(furan-2-yl)but-2-yn-1-ol **1m** (500 mg, 2.23 mmol) in ethanol (0.5 M) to give 3-ethoxy-2,2'-bifuran **2m** as a colourless oil (328 mg, 1.69 mmol, 78%); R_f = 0.52 (10:1 cyclohexane:ethyl acetate); ν_{max} (film/cm^{-1}) 2980s (C-H), 1620s; ^{1}H NMR (400 MHz, MeOH-d_4) 7.32 (1H, d, *J* = 2.2, Ar*H*), 7.27–7.22 (2H, m, Ar*H*), 7.05–7.02 (1H, m, Ar*H*), 6.52 (1H, d, *J* = 2.2, Ar*H*), 4.12 (2H, q, *J* = 7.1, OCH_2), 1.42 (3H, t, *J* = 7.1, CH_2CH_3); ^{13}C NMR (100 MHz, MeOH-d_4) 142.6 (*Ar*), 139.9 (*Ar*), 134.6 (*Ar*), 132.3 (*Ar*), 126.6 (*Ar*), 122.1 (*Ar*), 120.0 (*Ar*), 103.7 (*Ar*), 67.1 (OCH_2), 14.1 (CH_2CH_3); HRMS (CI$^+$) found [M+H]$^+$ 195.0473; $C_{10}H_{11}SO_2$ requires 195.0473.

3-(3-Ethoxyfuran-2-yl)pyridine 2n

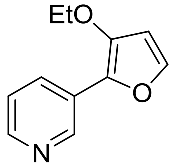

Subjecting 3-(3-ethoxyfuran-2-yl)pyridine **1n** to the General Furan Procedure in ethanol (0.5 M) resulted in no reaction.

A stirring solution of 3-(3-ethoxyfuran-2-yl)pyridine **1n** (200 mg, 0.851 mmol) and methanesulfonic acid (0.28 mL, 0.48 g, 4.3 mmol) in ethanol (4.2 mL) was treated with [Bis(trifluoromethanesulfonyl)imidate](triphenylphosphine)gold(I) (2:1) toluene adduct (33 mg, 0.021 mmol, 2.5 mol%, 5 mol% [Au]) at room temperature. The resulting solution was stirred for 2 h at room temperature before it was treated with water (5 mL) and stirred for a further 1 h. The reaction was then cooled to 0 °C, diluted with diethyl ether (20 mL) and quenched with aq. sat. $NaHCO_3$ (20 mL). The aqueous extract was washed with diethyl ether (3 × 20 mL) and the combined organic extracts were dried (phase separator) and concentrated to give the crude product, which was purified by flash column chromatography (aminopropyl column; 0 to 10% petrol 30–40 °C: TBME) to give 3-(3-ethoxyfuran-2-yl)pyridine **2n** as a colourless oil (117 mg, 0.619 mmol, 73%); R_f = 0.23 (10:1 cyclohexane:ethyl acetate); ν_{max} (film/cm^{-1}) 2959w (C-H), 1659s, 1678s, 1440s; ^{1}H NMR (400 MHz, MeOH-d_4) 8.94 (1H, d, *J* = 1.7, Ar*H*), 8.31 (1H, dd, *J* = 4.9, 1.7, Ar*H*), 8.14 (1H, d, *J* = 8.1, Ar*H*), 7.49 (1H, d, *J* = 2.2, Ar*H*), 7.44 (1H, dd, *J* = 8.1, 4.9, Ar*H*), 6.64 (1H, d, *J* = 2.2, Ar*H*), 4.19 (2H, q, *J* = 7.0, OCH_2), 1.45 (3H, t, *J* = 7.0, CH_2CH_3); ^{13}C NMR (100 MHz, MeOH-d_4) 146.5 (*Ar*), 145.0 (*Ar*), 142.9 (*Ar*), 142.1 (*Ar*), 132.9 (*Ar*), 129.9 (*Ar*), 127.8 (*Ar*), 123.8 (*Ar*), 103.6 (*Ar*), 67.0 (OCH_2), 14.0 (CH_2CH_3); HRMS (CI^+) found $[M+H]^+$ 190.0863; $C_{11}H_{12}NO_2$ requires 190.0868.

1-(4-Ethoxy-5-phenethylfuran-2-yl)-*N*,*N*-dimethylmethanamine 2o

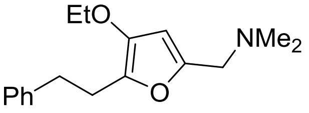

Prepared according to the modified procedure of Sheppard *et al.*[1]; A stirring solution of 3-ethoxy-2-phenethylfuran **2a** (50 mg, 0.23 mmol) in acetonitrile (2.3 mL) was treated with dimethylmethylideneammonium iodide (85 mg, 0.46 mmol) and the resulting mixture was stirred at room temperature for 16 h before it was concentrated *in vacuo* to give the crude product. This was purified by flash column chromatography (silica; 1:1 petrol 40–60 °C: diethyl ether with 1% NEt_3) to give 1-(4-ethoxy-5-phenethylfuran-2-yl)-*N*,*N*-dimethylmethanamine **2o** as a colourless oil (49 mg, 0.18 mmol, 78%); R_f = 0.30 (1:1 petrol 40–60 °C: ethyl acetate); ν_{max} (film/cm^{-1}) 2927s (C-H), 1635s, 1495s, 1453s, 1420s; ^{1}H NMR (400 MHz, MeOH-d_4) 7.27–7.09 (5H, m, Ar*H*), 6.28 (1H, d, *J* = 2.0, Ar*H*), 3.74 (2H, q, *J* = 7.1, OCH_2), 2.93–2.81 (4H, m, CH_2CH_2Ph), 1.19 (3H, t, *J* = 7.1, CH_2CH_3); ^{13}C NMR (125 MHz, $CDCl_3$) 142.4 (*Ar*), 141.3 (*Ar*), 139.3 (*Ar*), 139.0 (*Ar*), 128.1 (*Ar*), 127.8 (*Ar*), 125.5 (*Ar*), 103.6 (*Ar*), 67.4 (OCH_2), 33.9 (CH_2), 26.6 (CH_2), 14.0 (CH_2CH_3);. HRMS (ESI^+) found $[M+H]^+$ 274.1818; $C_{17}H_{24}NO_2$ requires 274.1807.

(3a*S*,4*R*,7*R*,7a*R*)-5-Ethoxy-2-methyl-4-phenethyl-3a,4,7,7a-tetrahydro-1*H*-4,7-epoxyisoindole-1,3(2*H*)-dione e*ndo*-3a and (3a*R*,4*R*,7*R*,7a*S*)-5-Ethoxy-2-methyl-4-phenethyl-3a,4,7,7a-tetrahydro-1*H*-4,7-epoxyisoindole-1,3(2*H*)-dione e*xo*-3a

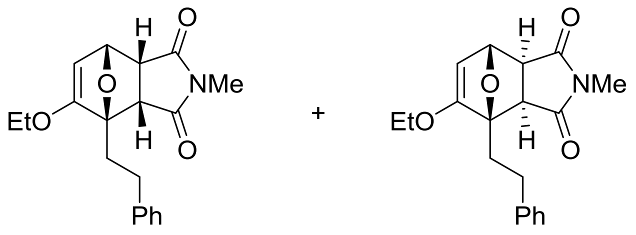

endo-**3a** *exo*-**3a**

Method A: Prepared from 3-ethoxy-2-phenethylfuran **2a** (108 mg, 0.500 mmol) and *N*-methylmaleimide according to the General Cycloaddition Procedure to give the title compounds as a colourless oil (153 mg, 0.467 mmol, 93%; *endo*-**3a**:*exo*-**3a** = 70:30).

Method B:Prepared from 3-ethoxy-2-phenethylfuran **2a** (1.00 g, 4.62 mmol) and *N*-methylmaleimide according to the General Cycloaddition Procedure to give the crude product (*endo*-**3a**:*exo*-**3a** = 75:25). Purification by flash column chromatography (0 to 100% cyclohexane:TBME) gave (3a*S*,4*R*,7*R*,7a*R*)-5-ethoxy-2-methyl-4-phenethyl-3a,4,7,7a-tetrahydro-1*H*-4,7-epoxyisoindole-1,3(2*H*)-dione *endo*-**3a** (906 mg, 2.77 mmol, 60%). Further elution of the column gave a mixture of (3a*S*,4*R*,7*R*,7a*R*)-5-ethoxy-2-methyl-4-phenethyl-3a,4,7,7a-tetrahydro-1*H*-4,7-epoxyisoindole-1,3(2*H*)-dione *endo*-**3a** and (3a*R*,4*R*,7*R*,7a*S*)-5-ethoxy-2-methyl-4-phenethyl-3a,4,7,7a-tetrahydro-1*H*-4,7-epoxyisoindole-1,3(2*H*)-dione *exo*-**3a** (202 mg, 0.618 mmol, 13%). Further elution of the column gave (3a*R*,4*R*,7*R*,7a*S*)-5-ethoxy-2-methyl-4-phenethyl-3a,4,7,7a-tetrahydro-1*H*-4,7-epoxyisoindole-1,3(2*H*)-dione *exo*-**3a** (310 mg, 0.948 mmol, 21%).

Method C:A solution of *N*-methylmaleimide (67 mg, 0.60 mmol) and 3-ethoxy-2-phenethylfuran **2a** (108 mg, 0.500 mmol) in dimethylcarbonate (0.50 mL) was stirred at 80 °C for 16 h. The reaction was then allowed to cool to room temperature before it was diluted with ethyl acetate and loaded onto an aminopropyl cartridge. After 5 minutes the cartridge was then flushed with ethyl acetate and the solvent removed *in vacuo* to give the title compounds as a colourless oil (153 mg, 0.467 mmol, 93%; *endo*-**3a**:*exo*-**3a** = 55:45).

Method D: A solution of [Bis(trifluoromethanesulfonyl)imidate](triphenylphosphine)gold(I) (2:1) toluene adduct (60 mg, 1 mol%, 2 mol% [Au]) in ethanol (1.9 mL) was added dropwise to a stirring solution of 6,6-diethoxy-1-phenylhex-4-yn-3-ol **1a** (1.00 g, 3.81 mmol) in ethanol (1.9 mL) at room temperature. The resulting solution was stirred for 3 h before it was treated with triphenyl phosphine (25 mg, 2.5 mol%). After a further 1 h the reaction was treated with *N*-methylmaleimide (508 mg, 4.57 mmol) and stirred for 16 h at room temperature. The reaction mixture was then loaded onto an aminopropyl cartridge and, after 5 minutes, eluted with ethyl acetate. The eluent was concentrated to give the crude product (*endo*-**3a**:*exo*-**3a** = 70:30), which was purified by flash column chromatography (KP-NH; 0 to 100% cyclohexane:TBME) to give the title compounds as a colourless oil (828 mg, 2.53 mmol, 66%; *endo*-**3a**:*exo*-**3a** = 70:30).

(3a*S*,4*R*,7*R*,7a*R*)-5-Ethoxy-2-methyl-4-phenethyl-3a,4,7,7a-tetrahydro-1*H*-4,7-epoxyisoindole-1,3(2*H*)-dione e*ndo*-3a: Isolated a white crystalline solid. m.p. 90–92 °C; R_f = 0.62 (2:1 petrol 60–80 °C:ethyl acetate); ν_{max} (film/cm^{-1}) 2981s (C-H), 1774m (C=O), 1710s (C=O), 1623s, 1432s; ^{1}H NMR (600 MHz, $CDCl_3$) 7.29 (2H, t, *J* = 7.3, Ar*H*), 7.23 (2H, d, *J* = 7.3, Ar*H*), 7.19 (1H, t, *J* = 7.3, Ar*H*), 5.18 (1H, dd, *J* = 5.3, 1.4, COC*H*), 4.96 (1H, d, *J* = 1.4, C=C*H*), 3.85–3.80 (1H, m, OC*H*H'), 3.67 (1H, dd, *J* = 7.6, 5.3, OCHC*H*CH), 3.57–3.51 (1H, m, OCH*H*'), 3.19 (1H, d, *J* = 7.6, OCHCHC*H*), 2.83–2.79 (5H, m, NC*H*$_3$; PhC*H*$_2$), 2.62–2.55 (1H, m, PhCH$_2$C*H*H'), 2.28–2.22 (1H, m, PhCH$_2$CH*H*'), 1.27 (3H, t, *J* = 7.0, CH$_2$C*H*$_3$); ^{13}C NMR (150 MHz, $CDCl_3$) 175.7 (*C*(O)), 174.2 (*C*(O)), 164.1 (EtO*C*), 141.7 (*Ar*), 128.5 (*Ar*), 128.5 (*Ar*), 126.1 (*Ar*), 96.6 (C=*C*H), 89.6 (PhCH$_2$CH$_2$*C*), 78.2 (COC*H*),

66.9 (O*C*H_2), 51.4 (OCH*C*HCH), 49.8 (OCHCH*C*H), 31.9 (PhCH$_2$*C*H_2), 30.5 (Ph*C*H_2), 24.5 (N*C*H_3), 14.2 (CH$_2$*C*H_3); HRMS (CI$^+$) found [M+H]$^+$ 328.1544; $C_{19}H_{22}NO_4$ requires 328.1543.

(3a*R*,4*R*,7*R*,7a*S*)-5-Ethoxy-2-methyl-4-phenethyl-3a,4,7,7a-tetrahydro-1*H*-4,7-epoxyisoindole-1,3(2*H*)-dione *exo*-3a: Isolated as a waxy solid. R_f = 0.50 (1:1 cyclohexane:ethyl acetate); ^{1}H NMR (400 MHz, MeOH-d$_4$) 7.36–7.16 (5H, m, Ar*H*), 5.20 (1H, d, *J* = 2.0, C=C*H*), 5.13 (1H, d, *J* = 2.0, COC*H*), 3.94–3.76 (2H, m, OC*H*$_2$), 3.15 (1H, d, *J* = 6.4, C*H*C(O)), 2.95 (1H, d, *J* = 6.4, C*H*C(O)), 2.90–2.74 (2H, m, PhC*H*$_2$), 2.44–2.36 (1H, m, PhCH$_2$C*H*H'), 2.31–2.22 (1H, m, PhCH$_2$CH*H*'), 1.39 (3H, t, *J* = 7.1, CH$_2$C*H*$_3$); ^{13}C NMR (150 MHz, MeOH-d$_4$) 177.1 (*C*(O)), 175.6 (*C*(O)), 166.6 (*C*OEt), 142.1 (*Ar*), 128.0 (*Ar*), 127.9 (*Ar*), 125.5 (*Ar*), 99.5 (C=*C*H), 88.8 (*C*OCH), 79.9 (CO*C*H), 66.5 (O*C*H$_2$), 54.3 (*C*HC(O)), 49.1 (*C*HC(O)), 30.4 (*C*H$_2$), 29.7 (*C*H$_2$), 23.4 (N*C*H$_3$), 13.2 (CH$_2$*C*H$_3$).

(3a*S*,4*R*,7*R*,7a*R*)-5-Methoxy-2-methyl-4-phenethyl-3a,4,7,7a-tetrahydro-1*H*-4,7-epoxyisoindole-1,3(2*H*)-dione *endo*-3b and (3a*R*,4*R*,7*R*,7a*S*)-5-Methoxy-2-methyl-4-phenethyl-3a,4,7,7a-tetrahydro-1*H*-4,7-epoxyisoindole-1,3(2*H*)-dione *exo*-3b

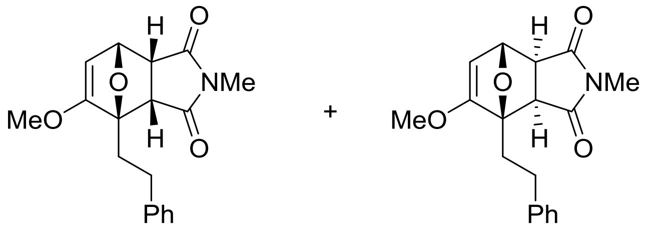

endo-**3b** *exo*-**3b**

Prepared from 3-methoxy-2-phenethylfuran **2a** (101 mg, 0.500 mmol) and *N*-methylmaleimide according to the General Cycloaddition Procedure over 4 h to give the crude product (*endo*-**3b**:*exo*-**3b** = 80:20). This was purified by flash column chromatography (0 to 100% cyclohexane: TBME) to give (3a*S*,4*R*,7*R*,7a*R*)-5-methoxy-2-methyl-4-phenethyl-3a,4,7,7a-tetrahydro-1*H*-4,7-epoxyisoindole-1,3(2*H*)-dione *endo*-**3b** as a white crystalline solid (105 mg, 0.335 mmol, 67%). Further elution of the column gave (3a*R*,4*R*,7*R*,7a*S*)-5-methoxy-2-methyl-4-phenethyl-3a,4,7,7a-tetrahydro-1*H*-4,7-epoxyisoindole-1,3(2*H*)-dione *exo*-**3b** as a white crystalline solid (34 mg, 0.11 mmol, 22%).

(3a*S*,4*R*,7*R*,7a*R*)-5-Methoxy-2-methyl-4-phenethyl-3a,4,7,7a-tetrahydro-1*H*-4,7-epoxyisoindole-1,3(2*H*)-dione *endo*-3b: m.p. 94–96 °C; R_f = 0.40 (1:1 petrol 60–80 °C:ethyl acetate); ν_{max} (film/cm^{-1}) 2931s (C-H), 1774s, 1699s (C=O), 1625s, 1433s; ^{1}H NMR (600 MHz, $CDCl_3$) 7.27 (2H, t, *J* = 7.5, Ar*H*), 7.24 (2H, d, *J* = 7.5, Ar*H*), 7.19 (1H, t, *J* = 7.5, Ar*H*), 5.20 (1H, dd, *J* = 5.2, 1.3, COC*H*), 5.02 (1H, d, *J* = 1.3, C=C*H*), 3.70 (1H, dd, *J* = 7.5, 5.2, OCHC*H*CH), 3.53 (3H, s, OC*H*$_3$), 3.21 (1H, d, *J* = 7.5, OCHCHC*H*), 2.85 (3H, s, NC*H*$_3$), 2.83–2.78 (2H, m, PhC*H*$_2$), 2.62–2.56 (1H, m, PhCH$_2$C*H*H'), 2.28–2.22 (1H, m, PhCH$_2$CH*H*'); ^{13}C NMR (150 MHz, $CDCl_3$) 175.6 (*C*(O)), 174.3 (*C*(O)), 165.4 (MeO*C*), 141.6 (*Ar*), 128.5 (*Ar*), 128.5 (*Ar*), 126.1 (*Ar*), 96.9 (C=*C*H), 89.7 (*C*OCH), 78.2 (CO*C*H), 58.2 (O*C*H$_3$), 51.3 (OCH*C*HCH), 49.8 (OCHCH*C*H), 31.9 (PhCH$_2$*C*H$_2$), 30.5 (Ph*C*H$_2$), 24.6 (N*C*H$_3$); data in accordance with the literature.[1]

(3a*R*,4*R*,7*R*,7a*S*)-5-Methoxy-2-methyl-4-phenethyl-3a,4,7,7a-tetrahydro-1*H*-4,7-epoxyisoindole-1,3(2*H*)-dione *exo*-3b: m.p. 149–151 °C; R_f = 0.50 (1:2 petrol 60–80 °C:ethyl acetate); ν_{max} (film/cm^{-1}) 2922s (C-H), 1764s, 1698s (C=O), 1633s, 1440s; ^{1}H NMR (600 MHz, $CDCl_3$) 7.29–7.25 (2H, m, Ar*H*), 7.24–7.21 (2H, m, Ar*H*), 7.17 (1H, t, *J* = 7.2, Ar*H*), 5.18 (1H, s, C=C*H*), 5.16 (1H, s, COC*H*), 3.66 (3H, s, OC*H*$_3$), 3.14 (1H, d, *J* = 6.4, C*H*C(O)), 2.96 (3H, s, NC*H*$_3$), 2.93 (1H, d, *J* = 6.4, C*H*C(O)), 2.85–2.73 (2H, m, PhC*H*$_2$), 2.39–2.33 (1H, m, PhCH$_2$C*H*H'), 2.30–2.20 (1H, m, PhCH$_2$CH*H*'); ^{13}C NMR (150 MHz, $CDCl_3$) 176.4 (*C*(O)), 174.9 (*C*(O)), 168.4 (MeO*C*), 141.9 (*Ar*), 128.5 (*Ar*), 128.4 (*Ar*), 126.0 (*Ar*), 99.6 (C=*C*H), 89500 M.3 (*C*OCH), 79.9 (CO*C*H), 58.3 (O*C*H$_3$), 54.4 (*C*HC(O)), 49.2 (*C*HC(O)), 30.8 (Ph*C*H$_2$), 25.0 (PhCH$_2$*C*H$_2$), 25.0 (N*C*H$_3$); data in accordance with the literature.[1]

(3a*S*,4*R*,7*R*,7a*R*)-4-Cyclohexyl-5-ethoxy-2-methyl-3a,4,7,7a-tetrahydro-1*H*-4,7-epoxyisoindole-1,3(2*H*)-dione e*ndo*-3c and (3a*R*,4*R*,7*R*,7a*S*)-4-Cyclohexyl-5-ethoxy-2-methyl-3a,4,7,7a-tetrahydro-1*H*-4,7-epoxyisoindole-1,3(2*H*)-dione e*xo*-3c

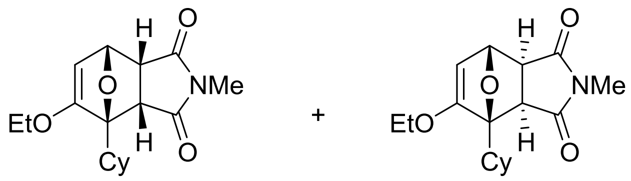

endo-**3c** *exo*-**3c**

Prepared from 2-cyclohexyl-3-ethoxyfuran **2c** (97 mg, 0.50 mmol) and *N*-methylmaleimide according to the General Cycloaddition Procedure over 4 h to give the title compounds **3c** as a colourless oil (145 mg, 0.475 mmol, 95%, *endo*-**3c**:*exo*-**3c** = 85:15); R_f = 0.51 (1:1 cyclohexane:ethyl acetate); ν_{max} (film/cm^{-1}) 2983s (C-H), 1773m, 1702s (C=O), 1624s, 1433s; ^{1}H NMR (400 MHz, MeOH-d_4) 5.21 (1H dd, *J* = 2.1, 1.0, C=C*H* *exo*-**3c**), 5.06 (1H, dd, *J* = 5.0, 2.1, COC*H* *endo*-**3c**), 5.00 (1H, d, *J* = 2.0, C=C*H* *endo*-**3c**), 4.96 (1H, d, *J* = 2.1, COC*H* *exo*-**3c**), 3.88–3.75 (1H, m, OC*H*H' *endo*-**3c**; 2H, m, OCH_2 *exo*-**3c**), 3.63 (1H, dd, *J* = 7.4, 5.0, OCHC*H*CH *endo*-**3c**), 3.58–3.51 (1H, m, OCH*H'* *endo*-**3c**), 3.51 (1H, d, *J* = 7.4, CyCC*H* *endo*-**3c**), 3.18–3.13 (2H, m, 2 × C*H*C(O) *exo*-**3c**), 2.91 (3H, s, NCH_3 *exo*-**3c**), 2.79 (3H, s, NCH_3 *endo*-**3c**), 2.36–2.29 (1H, m, C*H*H' *exo*-**3c**), 2.14–1.91 (3H, m, 3 × C*H*H' *endo*-**3c**), 1.91–1.66 (4H, m, 4 × C*H*H' *endo*-**3c**), 1.44–1.14 (3H, m, CH$_2$CH_3 *endo*-**3c**; 3H, m, CH$_2$CH_3 *exo*-**3c**; 3H, 3 × C*H*H' *endo*-**3c**) [9 × C*H*H' protons of *exo*-**3c** cannot be assigned with confidence]; ^{13}C NMR (100 MHz, MeOH-d_4) 177.1 (*C*(O)), 176.3 (*C*(O)), 175.3 (*C*(O)), 175.1 (*C*(O)), 167.4 (*C*OEt), 164.3 (*C*OEt), 100.6 (C=*C*H *exo*-**3c**), 96.6 (C=*C*H *endo*-**3c**), 92.3 (Cy*C*), 91.8 (Cy*C*), 79.3 (CO*C*H *exo*-**3c**), 77.3 (CO*C*H *endo*-**3c**), 66.4 (O*C*H$_2$), 66.2 (O*C*H$_2$), 54.3 (C*H*C(O) *exo*-**3c**), 51.3 (OCHC*H*CH *endo*-**3c**), 47.9 (C*H*C(O) *exo*-**3c**), 46.2 (CyCC*H* *endo*-**3c**), 39.2 (C*H*(CH$_2$)$_2$ *exo*-**3c**), 37.0 (C*H*(CH$_2$)$_2$ *endo*-**3c**), 27.8 (*C*H$_2$), 27.5 (*C*H$_2$), 26.7 (*C*H$_2$), 26.4 (*C*H$_2$), 26.3 (*C*H$_2$), 26.3 (*C*H$_2$), 26.2 (*C*H$_2$), 26.1 (*C*H$_2$), 26.1 (*C*H$_2$), 26.0 (*C*H$_2$), 23.5 (N*C*H$_3$), 23.2 (N*C*H$_3$), 13.3 (CH$_2$*C*H$_3$), 13.2 (CH$_2$*C*H$_3$); HRMS (CI$^+$) found [M+H]$^+$ 306.1700; $C_{17}H_{24}NO_4$ requires 306.1705.

(3a*S*,4*R*,7*R*,7a*R*)-4-Cyclopropyl-5-ethoxy-2-methyl-3a,4,7,7a-tetrahydro-1*H*-4,7-epoxyisoindole-1,3(2*H*)-dione e*ndo*-3d and (3a*R*,4*R*,7*R*,7a*S*)-4-Cyclopropyl-5-ethoxy-2-methyl-3a,4,7,7a-tetrahydro-1*H*-4,7-epoxyisoindole-1,3(2*H*)-dione e*xo*-3d

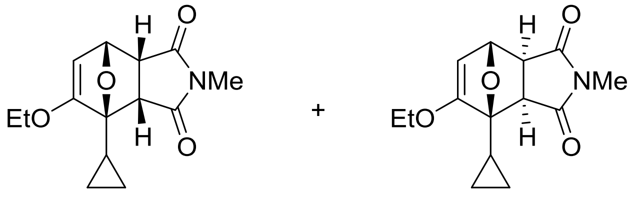

endo-**3d** *exo*-**3d**

Prepared from 2-cyclopropyl-3-ethoxyfuran **2d** (76 mg, 0.50 mmol) and *N*-methylmaleimide according to the General Cycloaddition Procedure over 4 h to give the title compounds **3d** as a colourless oil (118 mg, 0.448 mmol, 90%, *endo*-**3d**:*exo*-**3d** = 80:20); R_f = 0.48 (1:1 cyclohexane:ethyl acetate); ν_{max} (film/cm^{-1}) 2980s (C-H), 1702s (C=O), 1624m; ^{1}H NMR (400 MHz, MeOH-d_4) 5.15 (1H, d, *J* = 2.0, C=C*H* *exo*-**3d**), 5.04 (1H, dd, *J* = 5.1, 2.0, COC*H* *endo*-**3d**), 5.00 (1H, d, *J* = 2.0, C=C*H* *endo*-**3d**), 4.96 (1H, d, *J* = 2.0, COC*H* *exo*-**3d**), 3.93–3.76 (1H, m, OC*H*H' *endo*-**3d**; 2H, m, OCH_2 *exo*-**3d**), 3.67 (1H, dd, *J* = 7.6, 5.1, OCHC*H*CH *endo*-**3d**), 3.62–3.54 (1H, m, OCH*H'* *endo*-**3d**), 3.24–3.20 (1H, d, *J* = 7.6, OCHCHC*H* *endo*-**3d**; 1H, m, C*H*C(O) *exo*-**3d**), 3.00 (1H, d, *J* = 6.4, C*H*C(O) *exo*-**3d**), 2.92 (3H, s, NCH_3 *exo*-**3d**), 2.81 (3H, s, NCH_3 *endo*-**3d**), 1.57–1.47 (1H, m, C*H*(CH$_2$)$_2$ *endo*-**3d**), 1.44–1.38 (1H, m, C*H*(CH$_2$)$_2$ *exo*-**3d**), 1.34 (3H, t, *J* = 7.0, CH$_2$CH_3 *exo*-**3d**), 1.26 (3H, t, *J* = 7.1, CH$_2$CH_3 *endo*-**3d**), 0.79–0.49 (4H, m, CH(CH_2)$_2$ *endo*-**3d**, 3H, m, CH(CH_2)(C*H*H') *exo*-**3d**), 0.34–0.30 (1H, m, CH(CH_2)(CH*H'*)

exo-**3d**); ^{13}C NMR (100 MHz, MeOH-d$_4$) 177.2 (*C*(O)), 176.2 (*C*(O)), 175.6 (*C*(O)), 174.9 (*C*(O)), 167.5 (*C*OEt), 164.7 (*C*OEt), 98.6 (C=*C*H *exo*-**3d**), 95.9 (C=*C*H *endo*-**3d**), 89.1 (^{c}Pr*C*), 88.4 (^{c}Pr*C*), 79.1 (CO*C*H *exo*-**3d**), 77.3 (CO*C*H *endo*-**3d**), 66.4 (O*C*H$_2$), 66.4 (O*C*H$_2$), 54.6 (*C*HC(O) *exo*-**3d**), 51.2 (OCH*C*HCH *endo*-**3d**), 49.5 (*C*HC(O) *exo*-**3d**), 48.8 (OCHCH*C*H *endo*-**3d**), 23.4 (N*C*H$_3$), 23.3 (N*C*H$_3$), 13.2 (CH$_2$*C*H$_3$), 13.1 (CH$_2$*C*H$_3$), 9.5 (*C*H(CH$_2$)$_2$), 7.9 (*C*H(CH$_2$)$_2$), 0.9 (CH(*C*H$_2$)$_2$), 0.5 (CH(*C*H$_2$)$_2$), 0.3 (CH(*C*H$_2$)$_2$); HRMS (CI$^+$) found [M+H]$^+$ 264.1239; $C_{14}H_{18}NO_5$ requires 264.1236.

tert-Butyl 4-(((3a*S*,4*R*,7*R*,7a*R*)-5-ethoxy-2-methyl-1,3-dioxo-2,3,3a,4,7,7a-hexahydro-1*H*-4,7-epoxyisoindol-4-yl)methyl)piperidine-1-carboxylate e*ndo*-3e and *tert*-Butyl 4-(((3a*R*,4*R*,7*R*,7a*S*)-5-ethoxy-2-methyl-1,3-dioxo-2,3,3a,4,7,7a-hexahydro-1*H*-4,7-epoxyisoindol-4-yl)methyl)piperidine-1-carboxylate e*xo*-3e

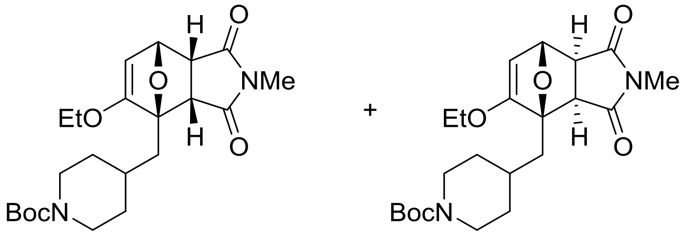

endo-**3e** *exo*-**3e**

Prepared from *tert*-butyl 4-((3-ethoxyfuran-2-yl)methyl)piperidine-1-carboxylate **1e** (44 mg, 0.14 mmol) and *N*-methylmaleimide according to the General Cycloaddition Procedure over 4 h to give the title compounds **3e** as a colourless oil (51 mg, 0.12 mmol, 85%, *endo*-**3e**:*exo*-**3e** = 75:25); R$_f$ = 0.50 (1:1 cyclohexane:ethyl acetate); ν_{max} (film/cm^{-1}) 2929s (C-H), 1701s (C=O), 1428m; ^{1}H NMR (600 MHz, CDCl$_3$) 5.19–5.16 (1H, m, COC*H* *endo*-**3e**; 1H, m, COC*H* *exo*-**3e**), 5.05 (1H, s, C=C*H* *exo*-**3e**), 4.94 (1H, s, C=C*H* *endo*-**3e**), 4.04 (2H, br. s, NC*H*H' *endo*-**3e**; 2H, br. s, NC*H*H' *exo*-**3e**), 3.89–3.75 (1H, m, OC*H*H' *endo*-**3e**; 2H, m, OC*H$_2$* *exo*-**3e**), 3.64 (1H, dd, *J* = 7.7, OCHC*H*CH *endo*-**3e**), 3.58–3.52 (1H, m, OCH*H*' *endo*-**3e**), 3.17 (1H, d, *J* = 7.7, OCHCHC*H* *endo*-**3e**), 3.08 (1H, d, *J* = 6.2, C*H*C(O) *exo*-**3e**), 2.98 (3H, s, NC*H$_3$*, *exo*-**3e**), 2.84 (3H, s, NC*H$_3$* *endo*-**3e**), 2.83 (1H, d, *J* = 6.2, C*H*C(O) *exo*-**3e**), 2.76–2.63 (2H, m, NCH*H*' *endo*-**3e**; 2H, br. s, NCH*H*' *exo*-**3e**), 2.30 (1H, dd, *J* = 15.0, 5.5, COCC*H*H' *endo*-**3e**), 2.09 (1H, dd, *J* = 15.4, 5.5, CHOCC*H*H' *exo*-**3e**), 1.91–1.65 (1H, m, COCCH*H*' *endo*-**3e**; 1H, m, N(CH$_2$C*H*H')$_2$ *endo*-**3e**; 1H, m, N(CH$_2$CH$_2$)$_2$C*H* *endo*-**3e**; 1H, m, COCCH*H*' *exo*-**3e**; 1H, m, N(CH$_2$C*H*H')$_2$ *exo*-**3e**; 1H, m, N(CH$_2$CH$_2$)$_2$C*H* *exo*-**3e**), 1.59 (9H, s, C(C*H$_3$*)$_3$ *exo*-**3e**), 1.46 (9H, s, C(C*H$_3$*)$_3$ *endo*-**3e**), 1.35 (3H, t, *J* = 7.0, CH$_2$C*H$_3$* *exo*-**3e**), 1.28 (3H, t, *J* = 7.0, CH$_2$C*H$_3$* *endo*-**3e**), 1.25–1.11 (1H, m, N(CH$_2$C*H*H')$_2$ *endo*-**3e**; 1H, m, N(CH$_2$C*H*H')$_2$ *exo*-**3e**); ^{13}C NMR (150 MHz, CDCl$_3$) 176.4 (*C*(O)N), 175.5 (*C*(O)N), 175.0 (*C*(O)N), 174.1 (*C*(O)N), 166.9 (EtO*C*), 164.1 (EtO*C*), 154.9 (*C*O^{t}Bu), 98.8 (C=*C*H *exo*-**3e**), 96.1 (C=*C*H *endo*-**3e**), 89.6 (*C*OCH), 89.2 (*C*OCH), 79.9 (CO*C*H), 79.2 (*C*Me$_3$), 79.1 (*C*Me$_3$), 78.1 (CO*C*H), 66.8 (O*C*H$_2$), 66.6 (O*C*H$_2$), 54.2 (*C*H), 51.0 (*C*H), 50.8 (*C*H), 49.8 (*C*H), 43.9 (br. N*C*H$_2$ *endo*-**3e**; N*C*H$_2$ *exo*-**3e**), 36.1 (*C*H$_2$), 33.1 (br. NCH$_2$*C*H$_2$), 32.9 (N(CH$_2$CH$_2$)$_2$*C*H), 32.6 (N(CH$_2$CH$_2$)$_2$*C*H), 32.5 (br. NCH$_2$*C*H$_2$), 28.5 (C(*C*H$_3$)$_3$ *endo*-**3e**; C(*C*H$_3$)$_3$ *exo*-**3e**) 24.8 (N*C*H$_3$), 24.4 (N*C*H$_3$), 14.2 (CH$_2$*C*H$_3$), 14.1 (CH$_2$*C*H$_3$); HRMS (CI$^+$) found [M+H]$^+$ 421.2333; $C_{22}H_{33}N_2O_6$ requires 421.2339; Strong ROE between OCHC*H*CH *endo*-**3e** and COC*H* *endo*-**3e**; Weak ROE between OCHC*H*CH *exo*-**3e** and COC*H* *exo*-**3e**.

(3a*S*,4*S*,7*R*,7a*R*)-5-Ethoxy-2-methyl-4-phenyl-3a,4,7,7a-tetrahydro-1*H*-4,7-epoxyisoindole-1,3(2*H*)-dione e*ndo*-3f and (3a*R*,4*S*,7*R*,7a*S*)-5-Ethoxy-2-methyl-4-phenyl-3a,4,7,7a-tetrahydro-1*H*-4,7-epoxyisoindole-1,3(2*H*)-dione e*xo*-3f

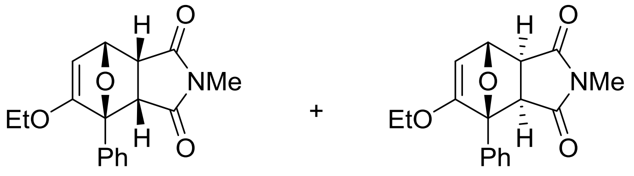

endo-**3f** *exo*-**3f**

Prepared from 3-ethoxy-2-phenylfuran **2f** (94 mg, 0.50 mmol) and *N*-methylmaleimide according to the General Cycloaddition Procedure over 6 h to give the title compounds **3f** as a white crystalline solid (128 mg, 0.428 mmol, 86%, *endo*-**3f**:*exo*-**3f** = 80:20).

Prepared from 3-ethoxy-2-phenylfuran **2f** (1.00 g, 5.32 mmol) and *N*-methylmaleimide according to the General Cycloaddition Procedure over 24 h to give the crude product, which was purified by column chromatography (KP-NH_2; 0 to 70% cyclohexane:TBME) to give (3a*S*,4*S*,7*R*,7a*R*)-5-ethoxy-2-methyl-4-phenyl-3a,4,7,7a-tetrahydro-1*H*-4,7-epoxyisoindole-1,3(2*H*)-dione *endo*-**3f** as a white crystalline solid (1.04 g, 3.47 mmol, 65%). Further elution of the column gave (3a*R*,4*S*,7*R*,7a*S*)-5-ethoxy-2-methyl-4-phenyl-3a,4,7,7a-tetrahydro-1*H*-4,7-epoxyisoindole-1,3(2*H*)-dione *exo*-**3f** as a white crystalline solid (334 mg, 1.12 mmol, 21%).

(3a*S*,4*S*,7*R*,7a*R*)-5-Ethoxy-2-methyl-4-phenyl-3a,4,7,7a-tetrahydro-1*H*-4,7-epoxyisoindole-1,3(2*H*)-dione e*ndo*-3f: m.p. 96–98 °C; R_f = 0.45 (3:1 petrol 60–80 °C:ethyl acetate); ν_{max} (film/cm^{-1}) 2982 (C-H), 1774s (C=O), 1700s (C=O), 1623s, 1500s, 1432s; ^{1}H NMR (600 MHz, $CDCl_3$) 7.89–7.79 (2H, m, Ar*H*), 7.47–7.38 (3H, m, Ar*H*), 5.32 (1H, dd, *J* = 5.2, 2.1, COC*H*), 5.02 (1H, d, *J* = 2.1, C=C*H*), 3.84–3.78 (2H, m, OC*H*H'; OCHC*H*CH), 3.58–3.52 (2H, m, OCH*H*'; PhCC*H*), 2.91 (3H, s, NC*H*$_3$), 1.18 (3H, t, *J* = 7.1, CH_2C*H*$_3$); ^{13}C NMR (150 MHz, $CDCl_3$) 175.4 (*C*(O)), 174.3 (*C*(O)), 164.9 (*C*OEt), 134.7 (*Ar*), 128.7 (*Ar*), 128.4 (*Ar*), 127.2 (*Ar*), 95.5 (C=*C*H), 90.3 (*C*Ph), 77.9 (CO*C*H), 67.1 (O*C*H$_2$), 51.8 (OCH*C*HCH), 51.0 (PhC*C*H), 24.6 (N*C*H$_3$), 13.9 (CH_2*C*H$_3$); HRMS (CI$^+$) found [M+H]$^+$ 300.1239; $C_{17}H_{18}NO_4$ requires 300.1230.

(3a*R*,4*S*,7*R*,7a*S*)-5-Ethoxy-2-methyl-4-phenyl-3a,4,7,7a-tetrahydro-1*H*-4,7-epoxyisoindole-1,3(2*H*)-dione e*xo*-3f: m.p. 119–121 °C; R_f = 0.38 (3:1 petrol 60–80 °C:ethyl acetate); ^{1}H NMR (400 MHz, MeOH-d_4) 7.51–7.45 (2H, m, Ar*H*), 7.39–7.29 (3H, m, Ar*H*), 5.28 (1H, d, *J* = 2.2, C=C*H*), 5.22 (1H, d, *J* = 2.2, COC*H*), 3.83 (2H, q, *J* = 7.1, OC*H*$_2$), 3.46 (1H, d, *J* = 6.4, C*H*C(O)), 3.38–3.34 (1H, m, C*H*C(O)), 2.80 (3H, s, NC*H*$_3$), 1.28 (3H, t, *J* = 7.1, CH_2C*H*$_3$); ^{13}C NMR (400 MHz, MeOH-d_4) 177.0 (*C*(O)), 174.5 (*C*(O)), 167.1 (*C*OEt), 132.6 (*Ar*), 127.5 (*Ar*), 127.1 (*Ar*), 126.4 (*Ar*), 98.1 (C=*C*H), 90.2 (*C*Ph), 79.8 (CO*C*H), 66.6 (O*C*H$_2$), 54.6 (*C*HC(O)), 49.9 (*C*HC(O)), 23.3 (N*C*H$_3$), 13.0 (CH_2*C*H$_3$).

(3a*S*,4*S*,7*R*,7a*R*)-5-Ethoxy-2-methyl-4-(4-(trifluoromethyl)phenyl)-3a,4,7,7a-tetrahydro-1*H*-4,7-epoxyisoindole-1,3(2*H*)-dione e*ndo*-3g and (3a*R*,4*S*,7*R*,7a*S*)-5-Ethoxy-2-methyl-4-(4-(trifluoromethyl)phenyl)-3a,4,7,7a-tetrahydro-1*H*-4,7-epoxyisoindole-1,3(2*H*)-dione e*xo*-3g

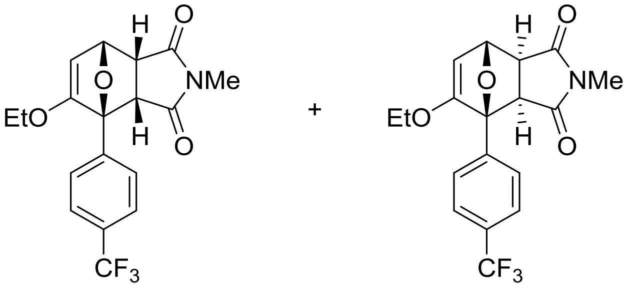

endo-**3g** *exo*-**3g**

Prepared from 3-ethoxy-2-(4-(trifluoromethyl)phenyl)furan **2g** (64 mg, 0.25 mmol) and *N*-methylmaleimide according to the General Cycloaddition Procedure over 24 h to give the title compounds **3g** as a white crystalline solid (69 mg, 0.18 mmol, 75%, *endo*-**3g**:*exo*-**3g** = 80:20); m.p. = 243–245 °C; R_f = 0.55 and 0.75 (1:1 cyclohexane:ethyl acetate); ν_{max} (film/cm^{-1}) 2986m (C-H), 1776m, 1702s (C=O), 1625s; ^{1}H NMR (400 MHz, MeOH-d$_4$) 7.98 (2H, d, *J* = 7.7, Ar*H* *endo*-**3g**), 7.71 (2H, d, *J* = 7.7, Ar*H* *endo*-**3g**), 7.66–7.65 (4H, m, Ar*H* *exo*-**3g**), 5.31–5.30 (1H, dd, *J* = 5.1, 2.2, COC*H* *endo*-**3g**; 1H, m, COC*H* *exo*-**3g**), 5.24 (1H, d, *J* = 2.2, C=C*H* *exo*-**3g**), 5.14 (1H, d, *J* = 2.2, C=C*H* *endo*-**3g**), 3.87 (1H, dd, *J* = 5.1, 7.6, OCHC*H*CH *endo*-**3g**), 3.86–3.77 (1H, m, OC*H*H' *endo*-**3g**; 2H, m, OCH_2 *exo*-**3g**), 3.60 (1H, d, *J* = 7.6, OCHCHC*H* *endo*-**3g**), 3.60–3.55 (1H, m, OCH*H*' *endo*-**3g**), 3.51 (1H, d, *J* = 6.4, C*H*C(O) *exo*-**3g**), 3.36 (1H, d, *J* = 6.4, C*H*C(O) *exo*-**3g**), 2.87 (3H, s, NCH_3 *endo*-**3g**), 2.78 (3H, s, NCH_3 *exo*-**3g**), 1.26 (3H, t, *J* = 7.1, CH$_2$CH_3 *exo*-**3g**), 1.15 (3H, t, *J* = 7.1, CH$_2$CH_3 *endo*-**3g**); ^{13}C NMR (150 MHz, MeOH-d$_4$) 176.7 (*C*(O)), 175.6 (*C*(O)), 174.5 (*C*(O)), 174.3 (*C*(O)), 166.4 (*C*OEt), 164.3 (*C*OEt), 139.6 (*Ar*), 137.1 (*Ar*), 130.1 (q, *J* = 33.2, *Ar*), 129.9 (q, *J* = 32.4, Ar), 127.7 (*Ar*), 127.2 (*Ar*), 124.4 (q, *J* = 4.4, *Ar*), 124.0 (q, *J* = 4.4, *Ar*), 124.4 (q, *J* = 270.9, *C*F$_3$), 124.3 (q, *J* = 270.9, *C*F$_3$), 98.4 (C=C*H* *exo*-**3g**), 95.6 (C=C*H* *endo*-**3g**), 89.6 (*C*OCH), 89.3 (*C*OCH), 80.1 (COC*H* *exo*-**3g**), 77.9 (COC*H* *endo*-**3g**), 66.9 (O*C*H$_2$), 66.9 (O*C*H$_2$), 54.4 (*C*HC(O) *exo*-**3g**), 51.5 (OCH*C*HCH *endo*-**3g**), 50.8 (OCHCH*C*H *endo*-**3g**), 49.9 (*C*HC(O) *exo*-**3g**), 23.4 (N*C*H$_3$), 23.3 (N*C*H$_3$), 13.0 (CH$_2$*C*H$_3$), 12.9 (CH$_2$*C*H$_3$); HRMS (CI$^+$) found [M+H]$^+$ 368.1111; $C_{18}H_{17}F_3NO_4$ requires 368.1110.

(3a*S*,4*S*,7*R*,7a*R*)-4-(4-Bromophenyl)-5-ethoxy-2-methyl-3a,4,7,7a-tetrahydro-1*H*-4,7-epoxyisoindole-1,3(2*H*)-dione e*ndo*-3h and (3a*R*,4*S*,7*R*,7a*S*)-4-(4-Bromophenyl)-5-ethoxy-2-methyl-3a,4,7,7a-tetrahydro-1*H*-4,7-epoxyisoindole-1,3(2*H*)-dione e*xo*-3h

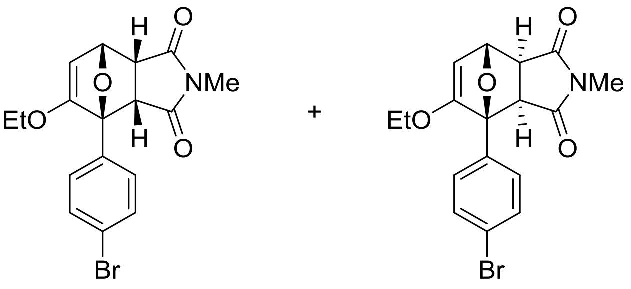

endo-**3h** *exo*-**3h**

Prepared from 3-ethoxy-2-(4-bromophenyl)furan **2h** (134 mg, 0.500 mmol) and *N*-methylmaleimide according to the General Cycloaddition Procedure to give the crude product (*endo*-**3h**:*exo*-**3h** = 80:20), which was purified by flash column chromatography (0 to 100% cyclohexane:TBME) to give title compounds **3h** as a colourless oil (149 mg, 0.392 mmol, 78%, *endo*-**3h**:*exo*-**3h** = 20:1); R_f = 0.28 and 0.49 (1:1 cyclohexane:ethyl acetate); ν_{max}

(film/cm^{-1}) 2981s (C-H), 1775w, 1702s (C=O), 1624s, 1491s; ^{1}H NMR (400 MHz, MeOH-d$_4$, *endo*-**3h**) 7.75–7.61 (2H, m, Ar*H*), 7.61–7.55 (2H, m, Ar*H*), 5.29 (1H, dd, *J* = 5.1, 2.1, COC*H*), 5.13 (1H, d, *J* = 2.1, C=C*H*), 3.87 (1H, dd, *J* = 5.1, 7.8, OCHC*H*CH), 3.84–3.77 (1H, m, OC*H*H'), 3.60 (1H, d, *J* = 7.8, OCHCHC*H*), 3.60–3.53 (1H, m, OCH*H*'), 2.88 (3H, s, NC*H*$_3$), 1.17 (3H, t, *J* = 7.1, CH$_2$C*H*$_3$); ^{13}C NMR (100 MHz, MeOH-d$_4$, *endo*-**3h**), 175.7 (*C*(O)), 174.6 (*C*(O)), 164.4 (*C*OEt), 134.5 (*Ar*), 130.7 (*Ar*), 129.0 (*Ar*), 122.1 (*Ar*), 95.5 (C=*C*H), 89.4 (*C*OCH), 77.7 (CO*C*H), 66.8 (O*C*H$_2$), 51.5 (*C*HC(O)), 50.7 (*C*HC(O)), 23.4 (N*C*H$_3$), 12.9 (CH$_2$*C*H$_3$); HRMS (CI$^+$) found [M+H]$^+$ 378.0340; $C_{17}H_{17}BrNO_4$ requires 378.0341.

(3a*S*,4*S*,7*R*,7a*R*)-5-Ethoxy-4-(4-methoxyphenyl)-2-methyl-3a,4,7,7a-tetrahydro-1*H*-4,7-epoxyisoindole-1,3(2*H*)-dione e*ndo*-3i and (3a*R*,4*S*,7*R*,7a*S*)-5-Ethoxy-4-(4-methoxyphenyl)-2-Methyl-3a,4,7,7a-tetrahydro-1*H*-4,7-epoxyisoindole-1,3(2*H*)-dione e*xo*-3i

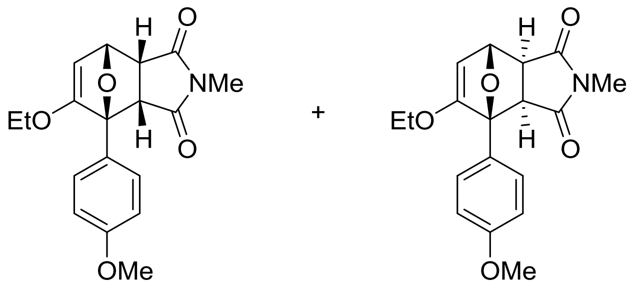

endo-**3i** *exo*-**3i**

Prepared from 3-ethoxy-2-(4-methoxyphenyl)furan **2i** (55 mg, 0.25 mmol) and *N*-methylmaleimide according to the General Cycloaddition Procedure to give the title compounds **3i** as a colourless oil (74 mg, 0.23 mmol, 90%, *endo*-**3i**:*exo*-**3i** = 80:20); R$_f$ = 0.15 and 0.08 (3:1 cyclohexane:ethyl acetate); ν_{max} (film/cm^{-1}) 2981s (C-H), 1773m, 1702s (C=O), 1622s, 1519s; ^{1}H NMR (400 MHz, CDCl$_3$) 7.76–7.71 (2H, m, Ar*H* *endo*-**3i**), 7.46–7.43 (2H, m, Ar*H* *exo*-**3i**), 7.01–6.94 (2H, m, Ar*H* *endo*-**3i**; 2H, m, Ar*H* *exo*-**3i**), 5.33–5.30 (1H, dd, *J* = 2.0, 5.1, COC*H* *endo*-**3i**; 1H, m, COC*H* *exo*-**3i**), 5.15 (1H, d, J = 2.2, C=C*H* *exo*-**3i**), 5.04 (1H, d, *J* = 2.0, C=C*H* *endo*-**3i**), 3.87–3.76 (3H, m, OC*H*$_3$ *endo*-**3i**, 1H, m, OCHC*H*CH *endo*-**3i**; 1H, m, OC*H*H' *endo*-**3i**; 3H, m, OC*H*$_3$ *exo*-**3i**; 1H, m, OC*H*$_2$ *exo*-**3i**), 3.60–3.52 (1H, m, OCHCHC*H* *endo*-**3i**; OCH*H*' *endo*-**3i**), 3.30 (1H, d, *J* = 6.3, C(O)C*H* *exo*-**3i**), 2.26 (1H, d, *J* = 6.3, C(O)C*H* *exo*-**3i**), 2.92 (3H, m, NC*H*$_3$ *endo*-**3i**), 2.88 (3H, m, NC*H*$_3$ *exo*-**3i**), 1.30 (3H, t, *J* = 7.1, CH$_2$C*H*$_3$ *exo*-**3i**), 1.20 (3H, t, *J* = 7.1, CH$_2$C*H*$_3$ *endo*-**3i**); ^{13}C NMR (100 MHz, CDCl$_3$) 176.2 (*C*(O)), 175.3 (*C*(O)), 174.2 (*C*(O)), 173.9 (*C*(O)), 167.5 (EtO*C*), 164.9 (EtO*C*), 159.8 (*Ar*), 159.3 (*Ar*), 128.5 (*Ar*), 127.7 (*Ar*), 126.8 (*Ar*), 124.1 (*Ar*), 113.7 (*Ar*), 113.2 (*Ar*), 97.9 (C=C*H* *exo*-**3i**), 95.4 (C=C*H* *endo*-**3i**), 90.2 (*C*OCH), 90.1 (*C*OCH), 79.8 (COCH *exo*-**3i**), 77.7 (CO*C*H *endo*-**3i**), 66.9 (O*C*H$_2$), 55.3 (O*C*H$_3$), 55.1 (O*C*H$_3$), 54.7 (C*H*), 51.8 (C*H*), 50.9 (*C*H), 50.0 (*C*H), 24.8 (N*C*H$_3$), 24.6 (N*C*H$_3$), 14.8 (CH$_2$*C*H$_3$), 13.8 (CH$_2$*C*H$_3$); HRMS (CI$^+$) found [M+H]$^+$ 330.1347; $C_{18}H_{20}NO_5$ requires 330.1342.

Methyl 3-((3a*S*,4*S*,7*R*,7a*R*)-5-ethoxy-2-methyl-1,3-dioxo-2,3,3a,4,7,7a-hexahydro-1*H*-4,7-epoxyisoindol-4-yl)benzoate e*ndo*-3j and Methyl 3-((3a*R*,4*S*,7*R*,7a*S*)-5-ethoxy-2-methyl-1,3-dioxo-2,3,3a,4,7,7a-hexahydro-1*H*-4,7-epoxyisoindol-4-yl)benzoate e*xo*-3j

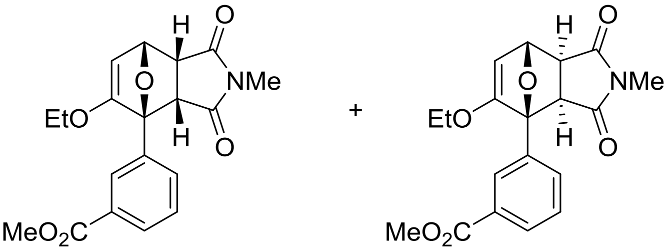

endo-**3j** *exo*-**3j**

Prepared from methyl 3-(3-ethoxyfuran-2-yl)benzoate **2j** (123 mg, 0.500 mmol) and *N*-methylmaleimide according to the General Cycloaddition Procedure to give the crude product (*endo*-**3j**:*exo*-**3j** = 75:25). This was purified by flash column chromatography (0 to 100% cyclohexane: TBME) to give methyl 3-((3a*S*,4*S*,7*R*,7a*R*)-5-ethoxy-2-methyl-1,3-dioxo-2,3,3a,4,7,7a-hexahydro-1*H*-4,7-epoxyisoindol-4-yl)benzoate *endo*-**3j** (107 mg, 0.300 mmol, 60%). Further elution of the column gave methyl 3-((3a*R*,4*S*,7*R*,7a*S*)-5-ethoxy-2-methyl-1,3-dioxo-2,3,3a,4,7,7a-hexahydro-1*H*-4,7-epoxyisoindol-4-yl)benzoate *exo*-**3j** (41 mg, 0.12 mmol, 23%).

Methyl 3-((3a*S*,4*S*,7*R*,7a*R*)-5-ethoxy-2-methyl-1,3-dioxo-2,3,3a,4,7,7a-hexahydro-1*H*-4,7-epoxyisoindol-4-yl)benzoate e*ndo*-3j: Isolated as a white crystalline solid; m.p. = 126–128 °C; R_f = 0.44 (1:1 cyclohexane:ethyl acetate); ν_{max} (film/cm^{-1}) 2980s (C-H), 1775m, 1720s (C=O ester and imide), 1624s, 1433s; ^{1}H NMR (400 MHz, DMSO-d_6) 8.30 (1H, t, *J* = 1.6, Ar*H*), 8.06–7.96 (2H, m, Ar*H*), 7.63 (1H, t, *J* = 7.7, Ar*H*), 5.36 (1H, dd, *J* = 5.1, 2.0, COC*H*), 5.22 (1H, d, *J* = 2.0, C=C*H*), 3.92–3.85 (4H, m, CO_2CH_3; OCHC*H*CH), 3.83–3.74 (1H, m, OC*H*H’), 3.68 (1H, d, *J* = 7.6, OCHCHC*H*), 3.59–3.50 (1H, m, OCH*H*’), 2.78 (3H, s, NCH_3), 1.05 (3H, t, *J* = 7.0, CH_2CH_3); ^{13}C NMR (100 MHz, DMSO-d_6) 175.3 (*C*(O)N), 174.5 (*C*(O)N), 166.5 (*C*(O)OMe or *C*OEt), 164.0 (*C*(O)OMe or *C*OEt), 136.4 (*Ar*), 132.5 (*Ar*), 130.0 (*Ar*), 129.6 (*Ar*), 129.2 (*Ar*), 128.2 (*Ar*), 97.0 (C=*C*H), 89.3 (*C*OCH), 77.7 (CO*C*H), 67.1 (O*C*H_2), 52.7 (O*C*H_3 or OCH*C*HCH), 51.7 (O*C*H_3 or OCH*C*HCH), 50.9 (OCHCH*C*H), 24.8 (N*C*H_3), 14.1 (CH_2*C*H_3) HRMS (CI$^+$) found [M+H]$^+$ 358.1294; $C_{19}H_{20}NO_6$ requires 358.1291.

Methyl 3-((3a*R*,4*S*,7*R*,7a*S*)-5-ethoxy-2-methyl-1,3-dioxo-2,3,3a,4,7,7a-hexahydro-1*H*-4,7-epoxyisoindol-4-yl)benzoate e*xo*-3j: Isolated as a white crystalline solid; m.p. = 118–120 °C; R_f = 0.27 (1:1 cyclohexane:ethyl acetate); ^{1}H NMR (400 MHz, DMSO-d_6) 7.99 (1H, t, *J* = 1.4, Ar*H*), 7.93 (1H, dd, *J* = 7.7, 1.4, Ar*H*), 7.75–7.69 (1H, m, Ar*H*), 7.58–7.52 (1H, m, Ar*H*), 5.37 (1H, d, *J* = 2.2, C=C*H*), 5.24 (1H, d, *J* = 2.2, COC*H*), 3.87 (3H, s, OCH_3), 3.80 (2H, q, *J* = 7.0, OCH_2), 3.56 (1H, d, *J* = 6.4, C*H*C(O)), 3.37 (1H, d, *J* = 6.4, C*H*C(O)), 2.69 (3H, s, NCH_3), 1.18 (3H, t, *J* = 7.0, CH_2CH_3); ^{13}C NMR (100 MHz, DMSO-d_6) 176.4 (*C*(O)N), 174.2 (*C*(O)N), 166.6 (*C*(O)OMe or *C*OEt), 165.9 (*C*(O)OMe or *C*OEt), 133.9 (*Ar*), 132.2 (*Ar*), 129.5 (*Ar*), 129.1 (*Ar*), 128.5 (*Ar*), 127.4 (*Ar*), 99.7 (C=*C*H), 89.7 (*C*OCH), 80.0 (CO*C*H), 67.1 (O*C*H_2), 54.5 (*C*HC(O)), 52.7 (O*C*H_3), 50.0 (*C*HC(O)), 24.7 (N*C*H_3), 14.3 (CH_2*C*H_3).

(3a*S*,4*S*,7*R*,7a*R*)-4-(*o*-Tolyl)-5-ethoxy-2-methyl-3a,4,7,7a-tetrahydro-1*H*-4,7-epoxyisoindole-1,3(2*H*)-dione e*ndo*-3k and (3a*R*,4*S*,7*R*,7a*S*)-4-(*o*-Tolyl)-5-ethoxy-2-methyl-3a,4,7,7a-tetrahydro-1*H*-4,7-epoxyisoindole-1,3(2*H*)-dione e*xo*-3k

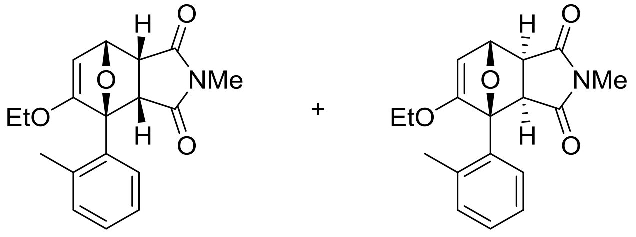

endo-**3k** *exo*-**3k**

Prepared from 3-ethoxy-2-(*o*-tolyl)furan **2k** (101 mg, 0.500 mmol) and *N*-methylmaleimide according to the General Cycloaddition Procedure over 24 h to give the title compounds **3k** as a colourless oil (135 mg, 0.431 mmol, 86%, *endo*:*exo* = 80:20); R_f = 0.63 and 0.59 (1:1 cyclohexane:ethyl acetate); ν_{max} (film/cm^{-1}) 2981s (C-H), 1773m, 1700s (C=O), 1625s, 1433s; ^{1}H NMR (400 MHz, DMSO-d_6) 8.01–7.97 (1H, m, Ar*H endo*-**3k**), 7.36–7.28 (2H, m, Ar*H endo*-**3k**; 1H, m, Ar*H exo*-**3k**), 7.23–7.17 (1H, m, Ar*H endo*-**3k**; 2H, m, Ar*H exo*-**3k**), 7.13–7.08 (1H, m, Ar*H exo*-**3k**), 5.36 (1H, d, *J* = 2.2, C=C*H exo*-**3k**), 5.31 (1H, dd, *J* = 5.1, 2.0, COC*H endo*-**3k**), 5.19–5.16 (1H, m, C=C*H endo*-**3k**; 1H, m, COC*H exo*-**3k**). 4.07 (1H, d, *J* = 7.8, OCHCHC*H endo*-**3k**), 3.83–3.73 (1H, m, OC*H*H' *endo*-**3k**; 1H, m, OCHC*H*CH *endo*-**3k**; 1H, m, OCH_2 *exo*-**3k**; 1H, m, C*H*C(O) *exo*-**3k**), 3.60–3.52 (1H, m, OC*H*H' *endo*-**3k**), 3.34–3.31 (1H, m, C*H*C(O) *exo*-**3k**; *H*OD), 2.76 (3H, s, NCH_3 *endo*-**3k**), 2.69 (3H, s, NCH_3 *exo*-**3k**), 2.59 (3H, s, ArCH_3 *exo*-**3k**), 2.31 (3H, s, ArCH_3 *endo*-**3k**), 1.16 (3H, t, *J* = 7.0, CH_2CH_3 *exo*-**3k**), 1.02 (3H, t, *J* = 7.0, CH_2CH_3 *endo*-**3k**); ^{13}C NMR (100 MHz, $CDCl_3$) 176.7 (*C*(O)), 175.5 (*C*(O)), 174.5 (*C*(O)), 174.3 (*C*(O)), 166.9 (*C*OEt), 164.3 (*C*OEt), 139.3 *(Ar)*, 137.3 *(Ar)*, 132.4 *(Ar)*, 132.4 *(Ar)*, 131.4 *(Ar)*, 131.2 *(Ar)*, 130.4 *(Ar)*, 129.4 *(Ar)*, 128.2 *(Ar)*, 125.9 *(Ar)*, 125.5 *(Ar)*, 100.0 (C=*C*H *exo*-**3k**), 96.0 (C=*C*H *endo*-**3k**), 91.3 (*C*OCH), 90.8 (*C*OCH), 78.8 (CO*C*H *exo*-**3k**), 77.9 (CO*C*H *endo*-**3k**), 67.0 (O*C*H_2), 66.8 (O*C*H_2), 53.7 (C*H*C(O) *exo*-**3k**), 51.0 (OCHCHCH *endo*-**3k**), 49.3 (C*H*C(O) *exo*-**3k**), 49.0 (OCHCHC*H* *endo*-**3k**), 24.7 (N*C*H_3), 24.7 (N*C*H_3), 22.3 (Ar*C*H_3), 20.8 (Ar*C*H_3), 14.4 (CH_2CH_3), 14.2 (CH_2CH_3); HRMS (CI$^+$) found $[M+H]^+$ 314.1399; $C_{18}H_{20}NO_4$ requires 314.1392.

(3a*S*,4*S*,7*R*,7a*R*)-4-(Furan-2-yl)-5-ethoxy-2-methyl-3a,4,7,7a-tetrahydro-1*H*-4,7-epoxyisoindole-1,3(2*H*)-dione e*ndo*-3l and (3a*R*,4*S*,7*R*,7a*S*)-4-(Furan-2-yl)-5-ethoxy-2-methyl-3a,4,7,7a-tetrahydro-1*H*-4,7-epoxyisoindole-1,3(2*H*)-dione e*xo*-3l

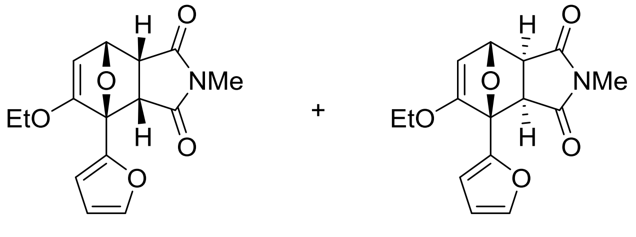

endo-**3l** *exo*-**3l**

Prepared from 3-ethoxy-2,2'-bifuran **3l** (104 mg, 0.584 mmol) and *N*-methylmaleimide according to the General Cycloaddition Procedure over 24 h to give the crude product (*endo*-**3l**:*exo*-**3l** = 90:10). This was purified by flash column chromatography (0 to 100% cyclohexane: ethyl acetate) to give the title compound **3l** as a white crystalline solid (143 mg, 0.494 mmol, 85%, *endo*:*exo* = 95:5); 122–124 °C; R_f = 0.16 (2:1 cyclohexane:ethyl acetate); ν_{max} (film/cm^{-1}) 2982s (C-H), 1775m, 1701s (C=O), 1631s 1434s; ^{1}H NMR (400 MHz, DMSO-d_6, *endo*-**3l**) 7.83–7.73 (1H, m, Ar*H*), 6.84 (1H, d, *J* = 3.4, Ar*H*), 6.57 (1H, dd, *J* = 3.4, 2.0, Ar*H*), 5.31–5.21 (2H, m, C=C*H*; COC*H*), 3.92 (1H, d, *J* = 7.6, OCHCHC*H*), 3.88–3.81 (2H, m, OC*H*H'; OCHC*H*CH), 3.59–3.51 (1H, m, OCH*H*'), 2.72 (3H, s,

NCH_3), 1.10 (3H, t, *J* = 7.1, CH_2CH_3); ^{1}H NMR (400 MHz, DMSO-d_6, *exo*-**3l**) 7.72–7.71 (1H, m, Ar*H*), 6.70 (1H, d, *J* = 3.2, Ar*H*), 6.46 (1H, dd, *J* = 2.0, 3.4, Ar*H*), 5.36 (1H, d, *J* = 2.0, C=C*H* or COC*H*), 5.16 (1H, d, *J* = 2.0, C=C*H* or COC*H*), 2.78 (3H, s, NC*H*3), 1.21 (3H, t, *J* = 6.9, CH_2CH_3), remaining resonances obscured by the major diastereomer; ^{13}C NMR (100 MHz, DMSO-d_6, *endo*-**3l**), 175.4 (*C*(O)), 173.6 (*C*(O)), 162.5 (*C*OEt), 147.6 (*Ar*), 144.9 (*Ar*), 112.8 (*Ar*), 111.2 (*Ar*), 97.9 (C=*C*H), 84.4 (*C*OCH), 76.0 (CO*C*H), 67.2 (O*C*H2), 51.0 (*C*HC(O)), 48.9 (*C*HC(O)), 24.6 (N*C*H3)), 14.3 (CH_2CH_3); HRMS (CI$^+$) found [M+H]$^+$ 290.1028; $C_{15}H_{16}NO_5$ requires 290.1029.

(3a*S*,4*R*,7*R*,7a*R*)-5-Ethoxy-2-methyl-4-(thiophen-2-yl)-3a,4,7,7a-tetrahydro-1*H*-4,7-epoxyisoindole-1,3(2*H*)-dione e*ndo*-3m and (3a*R*,4*R*,7*R*,7a*S*)-5-Ethoxy-2-methyl-4-(thiophen-2-yl)-3a,4,7,7a-tetrahydro-1*H*-4,7-epoxyisoindole-1,3(2*H*)-dione e*xo*-3m

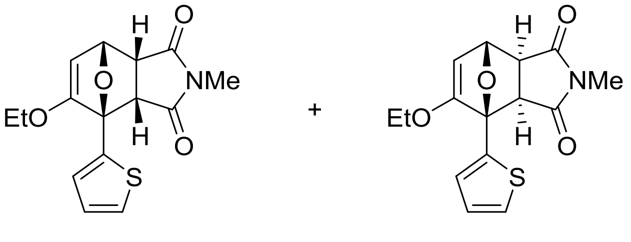

endo-**3m** *exo*-**3m**

Prepared from 3-ethoxy-2-(thiophen-3-yl)furan **2m** (97 mg, 0.50 mmol) and *N*-methylmaleimide according to the General Cycloaddition Procedure over 24 h to give the title compounds **3l** as a colourless oil (147 mg, 0.481 mmol, 96%, *endo*: *exo* = 70:30); R_f = 0.42 and 0.63 (1:1 cyclohexane:ethyl acetate); ν_{max} (film/cm^{-1}) 2982s (C-H), 1774m, 1700s C=O), 1626s, 1433s; ^{1}H NMR (400 MHz, DMSO-d_6) 7.63 (1H, dd, *J* = 5.0, 1.1, Ar*H* *endo*-**3m**), 7.57–7.48 (1H, m, Ar*H* *endo*-**3m**; 1H, m, Ar*H* *exo*-**3m**), 7.25 (1H, dd, *J* = 3.5, 1.1, Ar*H* *exo*-**3m**), 7.14 (1H, dd, *J* = 5.0, 3.7, Ar*H* *endo*-**3m**), 7.07 (1H, dd, *J* = 5.1, 3.5, Ar*H* *exo*-**3m**), 5.34 (1H, d, *J* = 2.0, C=C*H* *exo*-**3m**), 5.27 (2H, dd, *J* = 5.1, 2.2, COC*H* *endo*-**3m**), 5.21 (1H, d, *J* = 2.2, C=C*H* *endo*-**3m**), 5.18 (1H, d, *J* = 2.0, COC*H* *exo*-**3m**), 3.90–3.77 (1H, m, OCHC*H*CH *endo*-**3m**; 1H, m, OC*H*H' *endo*-**3m**; 1H, m, OC*H*2 *exo*-**3m**), 3.74 (1H, d, *J* = 7.8, OCHCHC*H* *endo*-**3m**), 3.59–3.51 (1H, m, OCH*H'* *endo*-**3m**), 3.37 (1H, d, *J* = 6.6, C*H*C(O) *exo*-**3m**), 3.33–3.32 (1H, m, C*H*C(O) *exo*-**3m**; *H*OD), 2.75 (3H, s, NC*H*3 *endo*-**3m**; 3H, s, NC*H*3 *exo*-**3m**), 1.22 (3H, t, *J* = 7.1, CH_2CH_3 *exo*-**3m**), 1.09 (3H, t, *J* = 7.0, CH_2CH_3 *endo*-**3m**); ^{13}C NMR (100 MHz, DMSO-d_6) 176.3 (*C*(O)), 175.4 (*C*(O)), 173.9 (*C*(O)), 173.7 (*C*(O)), 165.7 (EtO*C*), 163.4 (EtO*C*), 137.2 (*Ar*), 134.5 (*Ar*), 128.4 (*Ar*), 127.7 (*Ar*), 127.4 (*Ar*), 127.2 (*Ar*), 127.0 (*Ar*), 126.4 (*Ar*), 99.4 (C=*C*H *exo*-**3l**), 97.2 (C=*C*H *endo*-**3l**), 88.2 (*C*OCH), 87.2 (*C*OCH), 80.2 (CO*C*H *exo*-**3l**), 77.9 (CO*C*H *endo*-**3l**), 67.2 (O*C*H2), 67.2 (O*C*H2), 55.0 (*C*H), 51.7 (*C*H), 51.6 (*C*H), 51.0 (*C*H), 24.8 (N*C*H3), 24.7 (N*C*H3), 14.4 (CH_2CH_3), 14.2 (CH_2CH_3); HRMS (CI$^+$) found [M+H]$^+$ 306.0795; $C_{15}H_{16}NO_4S$ requires 306.0800.

(3a*S*,4*S*,7*R*,7a*R*)-5-Ethoxy-2-methyl-4-(pyridin-3-yl)-3a,4,7,7a-tetrahydro-1*H*-4,7-epoxyisoindole-1,3(2*H*)-dione e*ndo*-3n and (3a*R*,4*S*,7*R*,7a*S*)-5-Ethoxy-2-methyl-4-(pyridin-3-yl)-3a,4,7,7a-tetrahydro-1*H*-4,7-epoxyisoindole-1,3(2*H*)-dione e*xo*-3n

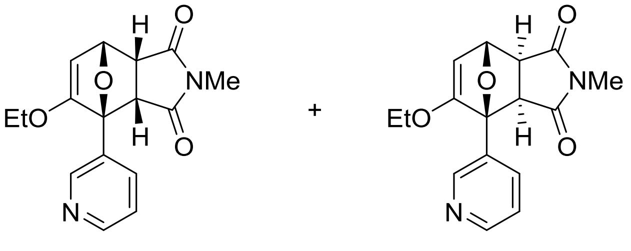

endo-**3n** *exo*-**3n**

Prepared from 3-(3-ethoxyfuran-2-yl)pyridine **2n** (25 mg, 0.015 mmol) and *N*-methylmaleimide according to the General Cycloaddition Procedure over 24 h to give the title compounds **3n** as a colourless oil (37 mg, 0.12 mmol, 92%, *endo*-**3n**: *exo*-**3n** = 70:30); R_f = 0.28 and 0.16 (1:1 cyclohexane:ethyl acetate); ν_{max} (film/cm^{-1}) 2980s (C-H),

1774s, 1695s (C=O), 1623s, 1480s; ^{1}H NMR (400 MHz, DMSO-d$_6$) 8.93 (1H, m, Ar*H* *endo*-**3n**), 8.66 (1H, m, Ar*H* *exo*-**3n**), 8.58 (1H, d, *J* = 5.0, Ar*H* *endo*-**3n**), 8.52 (1H, d, *J* = 5.0, ArH *exo*-**3n**), 8.26 (1H, d, *J* = 8.2, Ar*H* *endo*-**3n**), 7.95 (1H, d, *J* = 8.3, Ar*H* *exo*-**3n**), 7.52 (1H, dd, *J* = 8.2, 5.0, Ar*H* *endo*-**3n**), 7.47 (1H, dd, *J* = 8.3, 5.0, Ar*H* *exo*-**3n**), 5.36–5.35 (1H, m, COC*H* *endo*-**3n**; 1H, m, C=C*H* *exo*-**3n**), 5.29 (1H, d, *J* = 2.0, COC*H* *exo*-**3n**), 5.19 (1H, d, *J* = 2.2, C=C*H* *endo*-**3n**), 3.92–3.79 (1H, m, OCHC*H*CH *endo*-**3n**; 1H, m, OC*H*H' *endo*-**3n**; 2H, m, OC*H*$_2$ *exo*-**3n**), 3.67 (1H, d, *J* = 7.6, OCHCHC*H* *endo*-**3n**), 3.64–3.56 (1H, m, OCH*H*' *endo*-**3n**; 1H, m, C*H*C(O) *exo*-**3n**), 3.39 (1H, d, *J* = 6.4, C*H*C(O) *exo*-**3n**), 2.89 (3H, s, NC*H*$_3$ *endo*-**3n**), 2.81 (3H, s, NC*H*$_3$ *endo*-**3n**), 1.28 (3H, t, *J* = 7.1, CH$_2$C*H*$_3$ *exo*-**3n**), 1.18 (3H, t, *J* = 7.1, CH$_2$C*H*$_3$ *endo*-**3n**); ^{13}C NMR (100 MHz, MeOH-d$_4$) 176.6 (*C*(O)), 175.5 (*C*(O)), 174.4 (*C*(O)), 174.3 (*C*(O)), 166.2 (*C*OEt), 163.9 (*C*OEt), 148.6 (*Ar*), 148.0 (*Ar*), 147.5 (*Ar*), 147.0 (*Ar*), 135.8 (*Ar*), 135.3 (*Ar*), 132.0 (*Ar*), 129.7 (*Ar*), 123.3 (*Ar*), 123.0 (*Ar*), 98.5 (C=*C*H *exo*-**3n**), 95.8 (C=*C*H *endo*-**3n**), 88.3 (*C*OCH), 87.9 (*C*OCH), 80.4 (CO*C*H *exo*-**3n**), 78.2 (CO*C*H *endo*-**3n**), 67.0 (O*C*H$_2$), 67.0 (O*C*H$_2$), 54.3 (C*H*C(O) *exo*-**3n**), 51.3 (OCH*C*HCH *endo*-**3n**), 50.8 (OCHCH*C*H *endo*-**3n**), 49.7 (OCHCH*C*H *endo*-**3n**), 23.4 (N*C*H$_3$), 23.4 (N*C*H$_3$), 13.0 (CH$_2$*C*H$_3$), 12.9 (CH$_2$*C*H$_3$); HRMS (CI$^+$) found [M+H]$^+$ 301.1189; $C_{16}H_{17}N_2O_4$ requires 301.1188.

(3a*S*,4*R*,7*R*,7a*R*)-5-Ethoxy-2-methyl-4-phenethyl-3a,4,7,7a-tetrahydro-1*H*-4,7-epoxyisoindole-1,3(2*H*)-dione e*ndo*-3o and (3a*R*,4*R*,7*R*,7a*S*)-5-Ethoxy-2-methyl-4-phenethyl-3a,4,7,7a-tetrahydro-1*H*-4,7-epoxyisoindole-1,3(2*H*)-dione e*xo*-3o

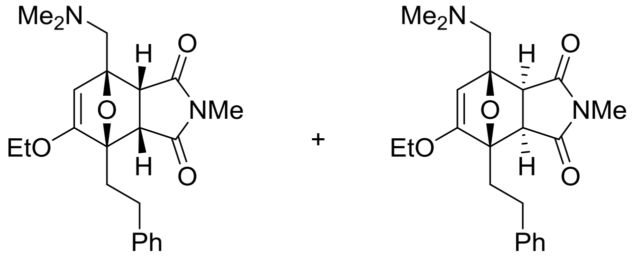

endo-**3l** *exo*-**3l**

Prepared from 1-(4-ethoxy-5-phenethylfuran-2-yl)-*N*,*N*-dimethylmethanamine **2o** (88 mg, 0.32 mmol) and *N*-methylmaleimide according to the General Cycloaddition Procedure to give the title compounds as a colourless oil (116 mg, 0.302 mmol, 94%; *endo*-**3o**:*exo*-**3o** = 20:80); R$_f$ = 0.35 (diethyl ether); ν_{max} (film/cm^{-1}) 2938s (C-H), 1772m, 1698s (C=O), 1626s, 1454s; ^{1}H NMR (600 MHz, MeOH-d$_4$) 7.29–7.12 (5H, m, Ar*H* *endo*-**3o**; 5H, m, Ar*H* *exo*-**3o**), 5.28 (1H, s, C=C*H* *exo*-**3o**), 5.01 (1H, s, C=C*H* *endo*-**3o**), 3.97–3.91 (1H, m, OC*H*H' *exo*-**3o**), 3.88–3.82 (1H, m, OC*H*H' *endo*-**3o**; 1H, m, OCH*H*' *exo*-**3o**), 3.59–3.51 (1H, m, OCH*H*' *endo*-**3o**), 3.47 (1H, d, *J* = 7.5, Me$_2$NCH$_2$CC*H*C(O) *endo*-**3o**), 3.37 (1H, d, *J* = 14.7, Me$_2$NCH*H*' *exo*-**3o**), 3.33 (1H, d, *J* = 7.5, EtOCCC*H*C(O) *endo*-**3o**), 3.22 (1H, d, *J* = 14.3, Me$_2$NCH*H*' *endo*-**3o**), 3.15 (1H, d, *J* = 6.4, Me$_2$NCH$_2$CC*H*C(O) *exo*-**3o**), 3.02 (1H, d, *J* = 6.4, EtOCCC*H*C(O) *exo*-**3o**), 2.89 (3H, s, C(O)NC*H*$_3$ *exo*-**3o**), 2.82–2.68 (3H, m, C(O)NC*H*$_3$ *endo*-**3o**; 2H, m, C*H*$_2$CH$_2$ *endo*-**3o**; 3H, m, C(O)NC*H*$_3$ *exo*-**3o**; 2H, m, C*H*$_2$CH$_2$ *exo*-**3o**), 2.74 (1H, d, *J* = 14.3, Me$_2$NCH*H*' *endo*-**3o**), 2.55 (1H, d, *J* = 14.7, Me$_2$NCH*H*' *exo*-**3o**), 2.48–2.43 (1H, m, CH$_2$C*H*H' *endo*-**3o**), 2.34 (6H, s, N(C*H*$_3$)$_2$ *endo*-**3o**; 6H, s, N(C*H*$_3$)$_2$ *exo*-**3o**), 2.33–2.06 (1H, m, CH$_2$C*H*H' *endo*-**3o**; 1H, m, CH$_2$C*H*$_2$ *exo*-**3o**), 1.37 (3H, t, *J* = 7.1, CH$_2$C*H*$_3$ *exo*-**3o**), 1.25 (3H, t, *J* = 7.1, CH$_2$C*H*$_3$ *endo*-**3o**); ^{13}C NMR (150 MHz, MeOH-d$_4$) 177.4 (*C*(O)), 176.6 (*C*(O)), 176.5 (*C*(O)), 176.3 (*C*(O)), 167.3 (*C*OEt), 165.0 (*C*OEt), 143.4 (*Ar*), 143.1 (*Ar*), 129.5 (*Ar*), 129.4 (*Ar*), 129.3 (*Ar*), 127.0 (*Ar*), 127.0 (*Ar*), 102.6 (C=*C*H *exo*-**3o**), 99.1 (C=*C*H *endo*-**3o**), 91.2 (*C*OC), 90.9 (*C*OC), 90.8 (*C*OC), 90.2 (*C*OC), 68.0 (O*C*H$_2$ *endo*-**3o**), 67.9 (O*C*H$_2$ *exo*-**3o**), 61.7 (N*C*H$_2$ *endo*-**3o**), 60.3 (N*C*H$_2$ *exo*-**3o**), 57.7 (Me$_2$NCH$_2$CC*H*C(O) *exo*-**3o**), 54.6 (Me$_2$NCH$_2$CC*H*C(O) *endo*-**3o**), 52.7 (EtOCC*C*HC(O) *endo*-**3o**), 52.6 (EtOCC*C*HC(O) *exo*-**3o**), 47.3 (N(*C*H$_3$)$_2$ *endo*-**3o**), 47.2 (N(*C*H$_3$)$_2$ *exo*-**3o**), 33.2 (*C*H$_2$CH$_2$ *endo*-**3o**), 32.1 (*C*H$_2$CH$_2$ *exo*-**3o**), 31.6 (*C*H$_2$CH$_2$ *endo*-**3o**), 31.1 (*C*H$_2$CH$_2$ *exo*-**3o**), 24.9 (N*C*H$_3$ imide *exo*-**3o**), 24.7 (N*C*H$_3$ imide *endo*-**3o**), 14.7 (CH$_2$C*H*$_3$ *exo*-**3o**), 14.6 (CH$_2$C*H*$_3$ *endo*-**3o**); Significant NOE between Me$_2$NCH$_2$CC*H*C(O) and Me$_2$NC*H*$_2$ for *endo*-**3o**; No Significant NOE between Me$_2$NCH$_2$CC*H*C(O) and Me$_2$NC*H*$_2$ for *exo*-**3o**; HRMS (CI$^+$) found [M+H]$^+$ 385.2127; $C_{22}H_{29}N_2O_4$ requires 385.2127.

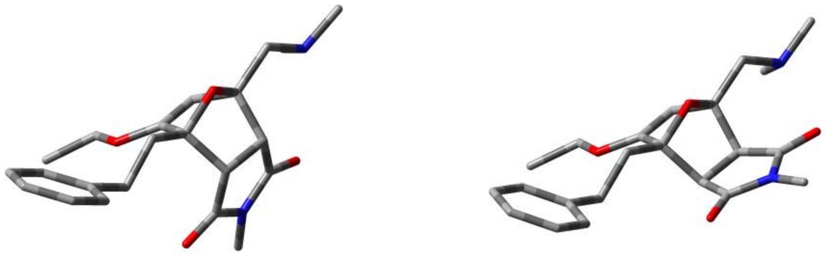

1-(4-Methylbenzyl)-1*H*-pyrrole-2,5-dione 4b

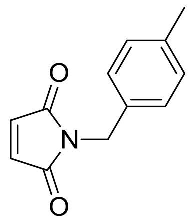

According to the modified procedure of Ordónez *et al*.[2]: *p*-Tolylmethanamine (2.6 ml, 2.4 g, 20 mmol) was added to a stirring solution of maleic anhydride (2.00 g, 20.4 mmol) in acetic acid (40 ml) at room temperature and the resulting solution was heated at reflux for 3 h. The reaction was then allowed to cool and concentrated under vacuum before being redissolved in ethyl acetate (50 mL) and washed with aq. sat. NH_4Cl. The aqueous extract was washed with ethyl acetate (3 × 50 mL) and the combined organic extracts were dried (phase separator) and concentrated to give the crude product. This was purified by flash column chromatography (0 to 100% cyclohexane: ethyl acetate) to give 1-(4-methylbenzyl)-1*H*-pyrrole-2,5-dione **4b** as a white crystalline solid (1.23 g, 6.11 mmol, 30%); m.p. = 100–102 °C; R_f = 0.62 (1:1 cyclohexane:ethyl acetate); ν_{max} (film/cm^{-1}) 3097m (C-H), 2941m (C-H), 1696s (C=O), 1515s, 1443s; ^{1}H NMR (400 MHz, MeOH-d_4) 7.15–7.10 (4H, m, Ar*H*), 7.06 (2H, s, *H*C=C*H*), 4.55 (2H, s, NC*H*$_2$), 2.27 (3H, s, ArC*H*$_3$); ^{13}C NMR (100 MHz, MeOH-d_4) 170.8 (*C*(O)), 136.6 (*Ar*), 134.6 (*C*H=*C*H), 133.7 (*Ar*), 129.1 (*Ar*), 127.2 (*Ar*), 40.7 (N*C*H$_2$), 20.6 (Ar*C*H$_3$); HRMS (CI$^+$) found [M+H$^+$ 202.0860; $C_{12}H_{12}NO_2$ requires 200.0868.

1-Cyclopropyl-1*H*-pyrrole-2,5-dione 4c

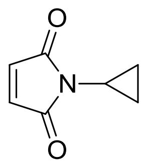

According to the modified procedure of. Ordónez *et al*.[2]: Cyclopropanamine (1.4 mL, 1.1 g, 20 mmol) was added to a stirring solution of maleic anhydride (2.00 g, 20.4 mmol) in acetic acid (40 ml) at room temperature and the resulting solution was heated at reflux for 2 h. The reaction was then allowed to cool and concentrated under vacuum before being dissolved in ethyl acetate (50 mL) and washed with aq. sat. $NaHCO_3$. The aqueous extract was washed with ethyl acetate (3 × 50 mL) and the combined organic extracts were dried (phase separator) and concentrated to give the crude product. This was purified by flash column chromatography (0 to 100% cyclohexane: TBME) to give 1-cyclopropyl-1*H*-pyrrole-2,5-dione **4c** as a white crystalline solid (1.30 g, 9.48 mmol, 47%); mp = 57–59 °C; R_f = 0.55 (1:1 cyclohexane:ethyl acetate); ν_{max} (film/cm^{-1}) 2977s (C-H), 1775m, 1757s (C=O), 1400s; ^{1}H NMR (400 MHz, DMSO-d_6) 6.93 (2H, s, *H*C=C*H*), 2.52–2.45 (1H, m, NC*H*), 0.85–0.79 (2H, m, CHC*H*H') 0.78–0.72 (2H, m, CHCH*H*'); ^{13}C NMR (100 MHz, DMSO-d_6) 171.4 (*C*(O)), 134.2 (*C*H=*C*H), 19.7 (N*C*H), 4.4 (NCH*C*H$_2$); HRMS (CI$^+$) found [M+H]$^+$ 138.0555; $C_7H_8NO_2$ requires 138.0555.

(3a*S*,4*R*,7*R*,7a*R*)-5-Ethoxy-2-phenyl-4-phenethyl-3a,4,7,7a-tetrahydro-1*H*-4,7-epoxyisoindole-1,3(2*H*)-dione e*ndo*-5a and (3a*R*,4*R*,7*R*,7a*S*)-5-Ethoxy-2-phenyl-4-phenethyl-3a,4,7,7a-tetrahydro-1*H*-4,7-epoxyisoindole-1,3(2*H*)-dione e*xo*-5a

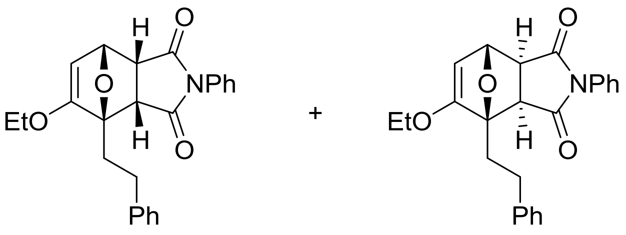

endo-**5a** *exo*-**5a**

Prepared from 3-ethoxy-2-phenethylfuran **2a** (108 mg, 0.500 mmmol) and 1-phenyl-1*H*-pyrrole-2,5-dione **4a** according to the General Cycloaddition Procedure over 4 h to give the title compounds as a waxy solid (183 mg, 0.470 mmol, 94%, *endo*-**5a**:*exo*-**5a** = 65:35); R_f = 0.64 (1:1 cyclohexane:ethyl acetate); ν_{max} (film/cm^{-1}) 2977s (C-H), 1713s (C=O), 1624s, 1498s; ^{1}H NMR (400 MHz, $CDCl_3$) 7.51–7.17 (10H, m, Ar*H*, *endo*-**5a**; 10H, m, Ar*H*, *exo*-**5a**), 5.32 (1H, t, *J* = 2.0, COC*H* *endo*-**5a**), 5.31 (1H, d, *J* = 2.0, COC*H* *exo*-**5a**), 5.19 (1H, d, *J* = 2.0, C=C*H* *exo*-**5a**), 5.15 (1H, d, *J* = 2.0, C=C*H* *exo*-**5a**), 3.96–3.80 (1H, m, OCHC*H*CH *endo*-**5a**; 1H, m, OC*H*H' *endo*-**5a**; 2H, m, OCH_2 *exo*-**5a**), 3.76–3.68 (1H, m, OCH*H*' *endo*-**5a**), 3.38 (1H, d, *J* = 7.8, OCHCHC*H* *endo*-**5a**), 3.32 (1H, d, *J* = 6.6, C(O)C*H* *exo*-**5a**), 3.10 (1H, d, *J* = 6.6, C(O)C*H* *exo*-**5a**), 2.95–2.82 (2H, m, PhCH_2 *endo*-**5a**; 2H, m, PhCH_2 *exo*-**5a**), 2.73–2.63 (1H, m, PhCH$_2$C*H*H' *endo*-**5a**), 2.50–2.42 (1H, m, PhCH$_2$C*H*H' *exo*-**5a**), 2.41–2.28 (1H, m, PhCH$_2$CH*H*' *endo*-**5a**; 1H, m, PhCH$_2$CH*H*' *exo*-**5a**), 1.40 (3H, t, *J* = 7.1, CH$_2$CH_3 *exo*-**5a**), 1.33 (3H, t, *J* = 7.1, CH$_2$CH_3 *endo*-**5a**); ^{13}C NMR (100 MHz, $CDCl_3$) 175.3 (*C*(O)), 174.3 (*C*(O)), 173.8 (*C*(O)), 172.8 (*C*(O)), 167.2 (*C*OEt), 164.4 (*C*OEt), 142.0 (*Ar*), 141.6 (*Ar*), 131.8 (*Ar*), 131.7 (*Ar*), 129.1 (*Ar*), 129.1 (*Ar*), 128.7 (*Ar*), 128.4 (*Ar*), 128.4 (*Ar*), 128.3 (*Ar*), 126.6 (*Ar*), 126.0 (*Ar*), 125.9 (*Ar*), 125.8 (*Ar*), 99.6 (C=*C*H *exo*-**5a**), 97.0 (C=*C*H *endo*-**5a**), 90.0 (*C*OCH), 89.7 (*C*OCH), 80.5 (CO*C*H), 78.5 (CO*C*H), 66.9 (O*C*H$_2$), 66.8 (O*C*H$_2$), 54.3 (*C*HC(O) *exo*-**5a**), 51.2 (OCH*C*HCH *endo*-**5a**), 49.5 (OCHCH*C*H *endo*-**5a**), 49.2 (*C*HC(O) *exo*-**5a**), 31.9 (*C*H$_2$), 30.7 (*C*H$_2$), 30.4 (*C*H$_2$), 29.5 (*C*H$_2$), 14.3 (CH$_2$*C*H$_3$), 14.3 (CH$_2$*C*H$_3$); HRMS (CI$^+$) found [M+H]$^+$ 390.1708; $C_{24}H_{24}NO_4$ requires 390.1705.

(3a*S*,4*R*,7*R*,7a*R*)-5-Ethoxy-2-(4-methylbenzyl)-4-phenethyl-3a,4,7,7a-tetrahydro-1*H*-4,7-epoxyisoindole-1,3(2*H*)-dione e*ndo*-5b and (3a*R*,4*R*,7*R*,7a*S*)-5-Ethoxy-2-(4-methylbenzyl)-4-phenethyl-3a,4,7,7a-tetrahydro-1*H*-4,7-epoxyisoindole-1,3(2*H*)-dione e*xo*-5b

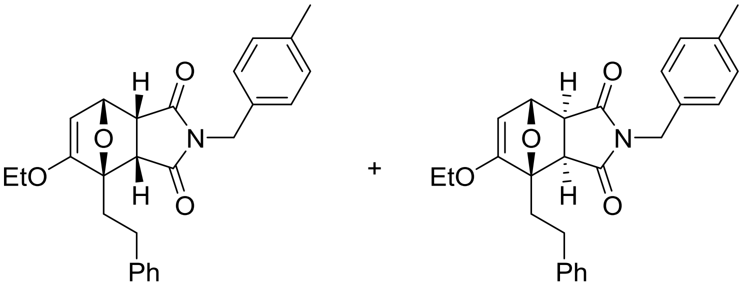

endo-**5b** *exo*-**5b**

Prepared from 3-ethoxy-2-phenethylfuran **2a** (108 mg, 0.500 mmol) and 1-(4-methylbenzyl)-1*H*-pyrrole-2,5-dione **4b** according to the General Cycloaddition Procedure over 4 h to give the title compounds **5b** as a waxy solid (173 mg, 0.414 mmol, 83%, *endo*-**5b**:*exo*-**5b** = 55:45); R_f = 0.51 (1:1 cyclohexane:ethyl acetate); ν_{max} (film/cm^{-1}) 2977s (C-H), 1771m, 1701s (C=O), 1623m, 1513m, 1430m; ^{1}H NMR (600 MHz, $CDCl_3$) 7.32–7.11 (9H, m, Ar*H* *endo*-**5b**; 9H, m, Ar*H* *exo*-**5b**), 5.22 (1H, d, *J* = 1.8, COC*H* *exo*-**5b**), 5.18 (1H, dd, *J* = 5.1, 1.5, COC*H* *endo*-**5b**), 5.12 (1H, d, *J* = 1.8, C=C*H* *exo*-**5b**), 4.75 (1H, d, *J* = 1.5, C=C*H* *endo*-**5b**), 4.62 (2H, AB system, *J* = 14.7, NCH_2

exo-**5b**), 4.53 (1H, d, *J* = 14.0, NC*H*H' *endo*-**5b**), 4.39 (1H, d, *J* = 14.0, NCH*H*' *endo*-**5b**), 3.91–3.89 (1H, m, OC*H*H' *exo*-**5b**), 3.83–3.78 (1H, m, OC*H*H' *exo*-**5b**), 3.69 (1H, dd, *J* = 7.7, 5.1, OCHC*H*CH *endo*-**5b**), 3.33–3.28 (1H, m, OC*H*H' *endo*-**5b**), 3.21 (1H, d, *J* = 7.7, OCHCHC*H* *endo*-**5b**), 3.14 (1H, d, *J* = 6.4, C(O)C*H* *exo*-**5b**), 2.92 (1H, d, *J* = 6.4, C(O)C*H* *exo*-**5b**), 2.86–2.75 (2H, m, CH_2Ph *endo*-**5b**; 2H, m, CH_2Ph *exo*-**5b**), 2.58–2.51 (1H, m, C*H*H'CH_2Ph *endo*-**5b**), 2.51–2.46 (1H, m, OCH*H*' *endo*-**5b**), 2.40–2.32 (1H, m, C*H*H'CH_2Ph *exo*-**5b**; 3H, m, ArCH_3 *endo*-**5b**; 3H, m, ArCH_3 *endo*-**5b**), 2.17–2.09 (1H, m, CH*H*'CH_2Ph *endo*-**5b**; 1H, m, CH*H*'CH_2Ph *exo*-**5b**), 1.38 (3H, t, *J* = 7.0, *exo*-**5b** CH_2CH_3), 1.06 (3H, t, *J* = 7.0, CH_2CH_3 *endo*-**5b**); ^{13}C NMR (150 MHz, $CDCl_3$) 175.8 (*C*(O)), 175.0 (*C*(O)), 174.5 (*C*(O)), 173.6 (*C*(O)), 167.1 (*C*OEt), 164.2 (*C*OEt), 142.0 (*Ar*), 141.6 (*Ar*), 137.7 (*Ar*), 137.4 (*Ar*), 133.1 (*Ar*), 132.7 (*Ar*), 129.6 (*Ar*), 129.2 (*Ar*), 129.2 (*Ar*), 128.4 (*Ar*), 128.4 (*Ar*), 128.3 (*Ar*), 128.2 (*Ar*), 125.9 (*Ar*), 125.8 (*Ar*), 99.3 (C=*C*H *exo*-**5b**), 95.9 (C=*C*H *endo*-**5b**), 89.3 (*C*OCH), 89.3 (*C*OCH), 79.9 (COCH *exo*-**5b**), 78.2 (CO*C*H *endo*-**5b**), 66.7 (OCH_2 *exo*-**5b**), 65.8 (OCH_2 *endo*-**5b**), 54.3 (C(O)*C*H *exo*-**5b**), 51.0 (OCH*C*HCH *endo*-**5b**), 49.5 (OCHCH*C*H *endo*-**5b**), 49.1 (C(O)*C*H *exo*-**5b**), 42.1 (NCH_2), 41.9 (NCH_2), 31.9 (CH_2), 30.6 (CH_2), 30.3 (CH_2), 29.3 (CH_2), 21.1 (ArCH_3), 21.1 (ArCH_3), 14.3 (CH_2CH_3), 14.2 (CH_2CH_3); HRMS (CI$^+$) found [M+H]$^+$ 418.2020; $C_{26}H_{28}NO_4$ requires 418.2018.

(3a*S*,4*R*,7*R*,7a*R*)-2-Cyclopropyl-5-ethoxy-4-phenethyl-3a,4,7,7a-tetrahydro-1*H*-4,7-epoxyisoindole-1,3(2*H*)-dione e*ndo*-5c and (3a*R*,4*R*,7*R*,7a*S*)-2-Cyclopropyl-5-ethoxy -4-phenethyl-3a,4,7,7a-tetrahydro-1*H*-4,7-epoxyisoindole-1,3(2*H*)-dione e*xo*-5c

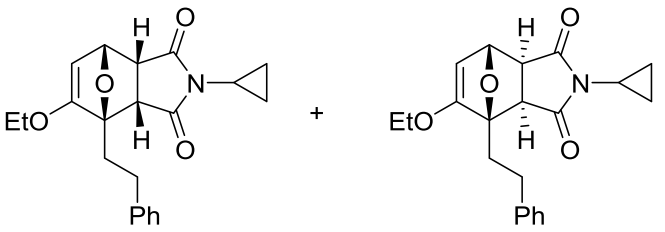

endo-**5c** *exo*-**5c**

Prepared from 3-ethoxy-2-phenethylfuran **2a** (108 mg, 0.500 mmol) and 1-cyclopropyl-1*H*-pyrrole-2,5-dione **5c** according to the General Cycloaddition Procedure over 4 h to give the title compounds as a waxy solid (153 mg, 0.433 mmol, 87%, *endo*:*exo*= 60:40); R_f = 0.69 (1:1 cyclohexane:ethyl acetate); ν_{max} (film/cm^{-1}) 2977s (C-H), 1775m, 1707s (C=O), 1401s; ^{1}H NMR (600 MHz, $CDCl_3$) 7.34–7.18 (5H, m, Ar*H*, *endo*-**5c**; 5H, m, Ar*H*, *exo*-**5c**), 5.19 (1H, dd, *J* = 5.4, 2.0, COC*H* *endo*-**5c**), 5.17 (1H, d, *J* = 2.0, COC*H* *exo*-**5c**), 5.11 (1H, d, *J* = 2.0, C=C*H* *exo*-**5c**), 5.00 (1H, d, *J* = 2.0, C=C*H* *endo*-**5c**), 3.92–3.75 (1H, m, OCH*H*' *endo*-**5c**; 1H, m, OCH_2 *exo*-**5c**), 3.65–3.58 (1H, m, OCH*H*' *endo*-**5c**; 1H, m, OCHC*H*CH *endo*-**5c**), 3.15 (1H, d, *J* = 7.8, OCHCHC*H* *endo*-**5c**), 3.07 (1H, d, *J* = 7.8, C*H*C(O) *exo*-**5c**), 2.89–2.74 (2H, m, CH_2Ph *endo*-**5c**; 1H, m, OCH*H*' *endo*-**5c**; 2H, m, CH_2Ph *endo*-**5c**; 1H, m, C*H*C(O) *exo*-**5c**), 2.63–2.55 (1H, m, NC*H* *exo*-**5c**; 1H, m, C*H*H'CH_2Ph *endo*-**5c**), 2.48 (1H, septet, *J* = 3.9, NC*H* *endo*-**5c**), 2.42–2.34 (1H, m, C*H*H'CH_2Ph *exo*-**5c**), 2.31–2.17 (1H, m, CH*H*'CH_2Ph *endo*-**5c**; 1H, m, CH*H*'CH_2Ph *exo*-**5c**), 1.37 (3H, t, *J* = 7.1, CH_2CH_3 *exo*-**5c**), 1.34 (3H, t, *J* = 7.1, CH_2CH_3 *endo*-**5c**), 0.98–0.85 (2H, m, CH(CH_2)$_2$ *endo*-**5c**; 4H, m, CH(C*H*H')$_2$ *exo*-**5c**), 0.82–0.78 (2H, m, CH(CH*H*')$_2$ *endo*-**5c**); ^{13}C NMR (150 MHz, $CDCl_3$) 176.7 (*C*(O)), 175.9 (*C*(O)), 175.4 (*C*(O)), 174.4 (*C*(O)), 167.0 (*C*OEt), 164.1 (*C*OEt), 142.0 (*Ar*), 141.6 (*Ar*), 128.4 (*Ar*), 128.4 (*Ar*), 128.4 (*Ar*), 128.3 (*Ar*), 126.0 (*Ar*), 125.8 (*Ar*), 99.4 (C=*C*H *exo*-**5c**), 96.5 (C=*C*H *endo*-**5c**), 89.6 (*C*OCH), 89.3 (*C*OCH), 80.1 (CO*C*H), 78.2 (CO*C*H), 66.7 (OCH_2), 66.6 (OCH_2), 53.8 (*C*HC(O) *exo*-**5c**), 50.6 (OCH*C*HCH *endo*-**5c**), 49.0 (OCHCH*C*H *endo*-**5c**), 48.5 (*C*HC(O) *exo*-**5c**), 31.8 (CH_2), 30.7 (CH_2), 30.3 (CH_2), 29.3 (CH_2), 22.2 (N*C*H), 21.9 (N*C*H), 14.3 (CH_2CH_3), 14.3 (CH_2CH_3), 5.0 (NCH*C*H), 5.0 (NCH*C*H), 4.9 (NCH*C*H), 4.6 (NCH*C*H); HRMS (CI$^+$) found [M+H]$^+$ 354.1708; $C_{21}H_{24}NO_4$ requires 354.1705.

(3a*S*,4*R*,7*R*,7a*R*)-5,5-Diethoxy-2-methyl-4-phenethylhexahydro-1*H*-4,7-epoxyisoindole-1,3(2*H*)-dione e*ndo*-6 and (3a*R*,4*R*,7*R*,7a*S*)-5,5-Diethoxy-2-methyl-4-phenethylhexahydro-1*H*-4,7-epoxyisoindole-1,3(2*H*)-dione e*xo*-6

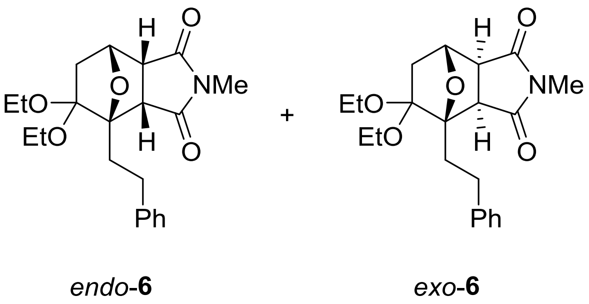

A solution of [Bis(trifluoromethanesulfonyl)imidate](triphenylphosphine)gold(I) (2:1) toluene adduct (60 mg, 1 mol%, 2 mol% [Au]) in ethanol (1.9 mL) was added dropwise to a stirring solution of 6,6-diethoxy-1-phenylhex-4-yn-3-ol **1a** (1.00 g, 3.81 mmol) and *N*-methylmaleimide (0.508 g, 4.57 mmol, 1.2 equivalents) in ethanol (5.7 mL) at room temperature. The resulting solution was stirred for 16 h before being filtered through an aminopropyl cartridge, eluting with ethyl acetate. The eluent was concentrated to give the crude product (*endo*-**6**:*exo*-**6** = 70:30), which was purified by flash column chromatography (0 to 100% cyclohexane:TBME) to give (3a*S*,4*R*,7*R*,7a*R*)-5,5-diethoxy-2-methyl-4-phenethylhexahydro-1*H*-4,7-epoxyisoindole-1,3(2*H*)-dione *endo*-**6** (276 mg, 0.740 mmol, 19%). Further elution of the column gave a mixture of *endo*-**6** and *exo*-**6** (528mg, 1.41 mmol, 37%). Further elution of the column gave (3a*R*,4*R*,7*R*,7a*S*)-5,5-diethoxy-2-methyl-4-phenethylhexahydro-1*H*-4,7-epoxyisoindole-1,3(2*H*)-dione e*xo*-**6** (228 mg 0.605 mmol, 16%).

(3a*S*,4*R*,7*R*,7a*R*)-5,5-Diethoxy-2-methyl-4-phenethylhexahydro-1*H*-4,7-epoxyisoindole-1,3(2*H*)-dione e*ndo*-6:Isolated as a white crystalline solid. m.p. = 123–125 °C; R_f = 0.61 (1:1 cyclohexane:ethyl acetate); ν_{max} (film/cm^{-1}) 2973s (C-H), 1772w, 1697s (C=O), 1431s; ^{1}H NMR (400 MHz, $CDCl_3$) 7.34–7.28 (2H, m, Ar*H*), 7.26–7.18 (3H, m, Ar*H*), 4.75 (1H, t, *J* = 6.3, COC*H*), 3.67–3.62 (1H, m, OCHC*H*CH), 3.50–3.42 (2H, m, OCHCHC*H*; OC*H*H’), 3.27–3.21 (3H, m, OCH*H*’; OC*H*$_2$), 2.87–2.79 (4H, m, NC*H*$_3$; PhC*H*H’), 2.75–2.66 (1H, m, PhCH*H*’), 2.48–2.40 (1H, m, PhCH$_2$C*H*H’), 2.37–2.31 (1H, m, CH*H*’C(O)), 2.08–1.99 (1H, m, PhCH$_2$CH*H*’), 1.41 (1H, d, *J* = 13.7, CH*H*’C(O)), 1.12 (3H, t, *J* = 7.1, CH$_2$C*H*$_3$), 0.95 (3H, t, *J* = 7.1, CH$_2$C*H*$_3$); ^{13}C NMR (100 MHz, $CDCl_3$) 175.8 (*C*(O)), 174.8 (*C*(O)), 142.4 (*Ar*), 128.9 (*Ar*), 128.6 (*Ar*), 126.3 (*Ar*), 107.6 (*C*(OEt)$_2$), 92.9 (*C*OCH), 75.4 (COC*H*), 58.3 (O*C*H$_2$), 55.9 (O*C*H$_2$), 52.4 (OCH*C*HCH), 49.8 (OCHCH*C*H), 38.9 (*C*H$_2$C(OEt)$_2$), 31.9 (*C*H$_2$), 30.1 (*C*H$_2$), 24.8 (N*C*H$_3$), 15.3 (CH$_2$*C*H$_3$), 15.2 (CH$_2$*C*H$_3$); HRMS (CI$^+$) found [M+H]$^+$ 374.1971; $C_{21}H_{28}NO_5$ requires 374.1968.

(3a*R*,4*R*,7*R*,7a*S*)-5,5-Diethoxy-2-methyl-4-phenethylhexahydro-1*H*-4,7-epoxyisoindole-1,3(2*H*)-dione e*xo*-6: Isolated as a colourless oil. R_f = 0.48 (1:1 cyclohexane:ethyl acetate); ^{1}H NMR (400 MHz, $CDCl_3$); 7.38–7.16 (5H, m, Ar*H*), 4.82 (1H, d, *J* = 5.9, COC*H*), 3.29 (1H, d, *J* = 7.1, C*H*C(O)), 3.10 (1H, d, *J* = 7.1, C*H*C(O)), 3.03 (3H, s, NC*H*$_3$), 2.89–2.76 (1H, m, C*H*H’CH$_2$Ph), 2.63–2.44 (1H, m, CH*H*’CH$_2$Ph; 1H, m, C*H*H’Ph), 2.44–2.31 (1H, m, CH*H*’Ph; 1H, m, C*H*H’C(O)), 1.71 (1H, d, *J* = 13.0, CH*H*’C(O)), 1.27 (3H, t, *J* = 7.0, CH$_2$C*H*$_3$), 1.18 (3H, t, *J* = 7.1, CH$_2$C*H*$_3$); ^{13}C NMR (100 MHz, $CDCl_3$) 177.1 (*C*(O)), 177.0 (*C*(O)), 142.7 (*Ar*), 128.5 (*Ar*), 128.3 (*Ar*), 125.8 (*Ar*), 108.0 (*C*(OEt)$_2$), 91.4 (*C*OCH), 77.2 (CO*C*H), 58.5 (O*C*H$_2$), 57.5 (O*C*H$_2$), 51.2 (*C*HC(O)), 46.8 (*C*HC(O)), 41.4 (*C*H$_2$CHO), 30.6 (*C*H$_2$), 29.4 (*C*H$_2$), 25.1 (N*C*H$_3$), 15.3 (CH$_2$*C*H$_3$), 15.1 (CH$_2$*C*H$_3$).

(3a*S*,4*S*,5*R*,7*R*,7a*R*)-5-Ethoxy-2-methyl-4-phenylhexahydro-1*H*-4,7-epoxyisoindole-1,3(2*H*)-dione 7

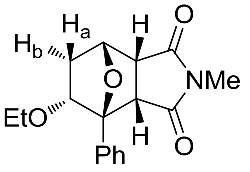

A solution of (3a*S*,4*S*,7*R*,7a*R*)-5-ethoxy-2-methyl-4-phenyl-3a,4,7,7a-tetrahydro-1*H*-4,7-epoxyisoindole-1,3(2*H*)-dione *endo*-**3f** (52 mg, 0.17 mmol) in ethanol (3.5 mL) was added to a flask primed with 10% Pd/C (30 mg, 0.28 mmol) at room temperature. The flask was placed under an atmosphere of hydrogen and stirred at room temperature for 16 h, before the reaction mixture was filtered through celite and concentrated to give (3a*S*,4*S*,5*R*,7*R*,7a*R*)-5-ethoxy-2-methyl-4-phenylhexahydro-1*H*-4,7-epoxyisoindole-1,3(2*H*)-dione **7** as a white crystalline solid (39 mg, 0.13 mmol, 76%); m.p. = 152–154 °C; R_f = 0.65 (1:1 cyclohexane:ethyl acetate); ν_{max} (film/cm^{-1}) 2977s (C-H), 1775m, 1699s (C=O), 1433s; ^{1}H NMR (400 MHz, $CDCl_3$) 7.83 (2H, d, *J* = 7.3, Ar*H*), 7.47–7.40 (2H, m, Ar*H*), 7.40–7.34 (1H, m, Ar*H*), 4.96 (1H, t, *J* = 6.1, COC*H*), 3.95 (1H, dd, *J* = 9.8, 3.2, C*H*OEt), 3.80–3.72 (1H, m, COCHC*H*CH), 3.52 (1H, d, *J* = 9.8, PhCC*H*C(O)), 3.38–3.29 (1H, m, OC*H*H'), 3.21–3.11 (1H, m, OCH*H*'), 3.04 (3H, s, NCH_3), 2.55–2.46 (1H, m, H_a), 1.66 (1H, dd, *J* = 13.7, 3.2, H_b), 1.04 (3H, t, *J* = 7.0, CH_2CH_3); ^{13}C NMR (100 MHz, $CDCl_3$) 175.3 (*C*(O)), 174.5 (*C*(O)), 139.3 (*Ar*), 128.5 (*Ar*), 128.1 (*Ar*), 126.0 (*Ar*), 91.4 (Ph*C*), 85.9 (*C*HOEt), 77.4 (CO*C*H), 66.8 (OCH_2), 53.0 (*C*HC(O)), 52.9 (*C*HC(O)), 35.6 (CH_a), 24.9 (NCH_3), 15.1 (CH_2CH_3); HRMS (CI$^+$) found [M+H]$^+$ 302.1399; $C_{17}H_{20}NO_4$ requires 302.1392.

(3a*S*,4*S*,5*R*,6*S*,7*S*,7a*R*)-5-Ethoxy-6-hydroxy-2-methyl-4-phenylhexahydro-1*H*-4,7-epoxyisoindole-1,3(2*H*)-dione 8

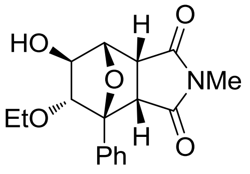

A solution of 9-borabicyclo[3.3.1]nonane (0.5 M in THF, 1.7 mL, 0.85 mmol) was added dropwise to a stirring solution of (3a*S*,4*S*,7*R*,7a*R*)-5-ethoxy-2-methyl-4-phenyl-3a,4,7,7a-tetrahydro-1*H*-4,7-epoxyisoindole-1,3(2*H*)-dione *endo*-**3f** (50 mg, 0.17 mmol) in dry THF (0.85 mL) at 0 °C. The resulting reaction mixture was stirred at 0 °C for 5 h before the reaction was treated with 2 M aqueous NaOH (2 mL) and hydrogen peroxide (30% in water, 1 mL) at 0 °C. The resulting mixture was stirred at room temperature for 16 h before the reaction was quenched with 10% aqueous $Na_2S_2O_3$ (10 mL) and extracted with ethyl acetate (3 × 20mL). The combined organic fractions were dried (phase separator) and concentrated to give the crude product, which was purified by flash column chromatography (0 to 100% cyclohexane:TBME) to give (3a*S*,4*S*,5*R*,6*S*,7*S*,7a*R*)-5-ethoxy-6-hydroxy-2-methyl-4-phenylhexahydro-1*H*-4,7-epoxyisoindole-1,3(2*H*)-dione **8** as a white crystalline solid (28 mg 0.088 mmol, 52%); m.p. = 216–218 °C; R_f = 0.65 (1:1 petrol 60–80 °C:ethyl acetate); ν_{max} (film/cm^{-1}) 3447m br. (O-H), 2973w (C-H), 1774w, 1695s (C=O), 1434; ^{1}H NMR (400 MHz, DMSO-d_6) 7.77–7.70 (2H, m, Ar*H*), 7.47–7.41 (2H, m, Ar*H*), 7.40–7.34 (1H, m, Ar*H*), 5.65 (1H, d, *J* = 4.6, CO*H*), 4.61 (1H, dd, *J* = 7.0, 1.5, COC*H*), 3.78 (1H, dd, *J* = 9.7, 7.0, OCHC*H*C(O)), 3.67 (1H, dd, *J* = 4.6, 1.5, C*H*OH), 3.59–3.56 (1H, m, C*H*OEt), 3.39 (1H, d, *J* = 9.7, PhCC*H*C(O)), 3.27–3.19 (2H, m, OCH_2), 2.84 (3H, s, NCH_3), 0.95 (3H, t, *J* = 7.0, CH_2CH_3); ^{13}C NMR (100 MHz DMSO-d_6) 175.2 (*C*(O)), 174.3 (*C*(O)), 139.6 (*Ar*), 128.7 (*Ar*), 128.5 (*Ar*), 126.4 (*Ar*), 95.8 (*C*OEt), 90.3 (Ph*C*), 83.4 (PhCO*C*H), 76.1 (*C*HOH), 66.4 (OCH_2), 52.1 (PhC*C*HC(O)), 50.1 (OCH*C*HC(O)), 24.9 (NCH_3), 15.3 (CH_2CH_3); HRMS (CI$^+$) found [M+H]$^+$ 318.1342; $C_{17}H_{20}NO_5$ requires 318.1342; Weak (but real) ROE between C*H*OH and NCH_3; No significant ROE between C*H*OEt and NCH_3

(3a*S*,4*S*,7*R*,7a*R*)-2-Methyl-4-phenyltetrahydro-1*H*-4,7-epoxyisoindole-1,3,5(2*H*,6*H*)-trione 9

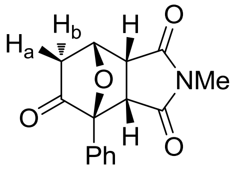

A solution of (3a*S*,4*S*,7*R*,7a*R*)-5-ethoxy-2-methyl-4-phenyl-3a,4,7,7a-tetrahydro-1*H*-4,7-epoxyisoindole-1,3(2*H*)-dione *endo*-**3f** (290 mg, 0.970 mmol) in ethyl acetate (5.0 mL) was loaded onto a strong cationic exchange (SCX) cartridge (20 g), which was then flushed with ethyl acetate after 10 minutes. The eluent was concentrated under reduced pressure to give the crude product, which was treated with cyclohexane (5.0 mL), sonicated and filtered to give (3a*S*,4*S*,7*R*,7a*R*)-2-methyl-4-phenyltetrahydro-1*H*-4,7-epoxyisoindole-1,3,5(2*H*,6*H*)-trione **9** as a white crystalline solid (210 mg, 0.775 mmol, 80%); m.p. 171–173 °C; R_f = 0.45 (2:1 petrol 60–80 °C:ethyl acetate); ν_{max} (film/cm^{-1}) 2988s (C-H), 1765s (C=O), 1694s (C=O), 1500s;. ^1H NMR (400 MHz, $CDCl_3$) 7.76–7.72 (2H, m, Ar*H*), 7.47–7.38 (3H, m, Ar*H*), 5.31 (1H, t, *J* = 6.0, OC*H*), 3.95 (1H, dd, *J* = 9.1, 6.0, OCHC*H*CH), 3.66 (1H, d, *J* = 9.1, PhCC*H*), 3.00 (3H, s, NCH_3), 2.85 (1H, dd, *J* = 18.3, 6.0, H_a), 4.32 (1H, d, *J* = 18.3, H_b); ^{13}C NMR (100 MHz, $CDCl_3$) 204.7 (*C*(O) ketone), 173.8 (*C*(O) imide), 172.3 (*C*(O) imide), 132.3 (*Ar*), 128.9 (*Ar*), 128.5 (*Ar*), 126.5 (*Ar*), 90.6 (Ph*C*O), 74.7 (CO*C*H), 53.2 (PhC*C*H), 51.5 (OCH*C*H), 41.7 (*C*H_a), 25.2 (NCH_3); HRMS (CI^+) found $[M+H]^+$ 272.0920; $C_{15}H_{14}NO_4$ requires 272.0923.

(3a*S*,4*S*,5*R*,7*R*,7a*R*)-5-Hydroxy-2-methyl-4-phenylhexahydro-1*H*-4,7-epoxyisoindole-1,3(2*H*)-dione 10

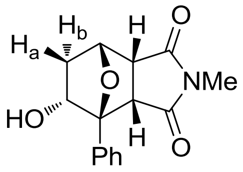

Sodium borohydride (35 mg, 0.92 mmol) was added to a stirring solution of (3a*S*,4*S*,7*R*,7a*R*)-2-methyl-4-phenyltetrahydro-1*H*-4,7-epoxyisoindole-1,3,5(2*H*,6*H*)-trione **9** (50 mg, 0.18 mmol) in methanol (1.8 mL) at 0 °C. The resulting solution was stirred at 0 °C for 1 h, before being filtered through a silica plug, eluting with ethyl acetate. The solvent was removed *in vacuo* to give the crude product, which was purified by flash column chromatography (0 to 100% cyclohexane:TBME) to give (3a*S*,4*S*,5*R*,7*R*,7a*R*)-5-hydroxy-2-methyl-4-phenylhexahydro-1*H*-4,7-epoxyisoindole-1,3(2*H*)-dione **10** as a white crystalline solid (35 mg, 0.13 mmol, 70%); m.p. = 185–187 °C; R_f = 0.43 (1:1 cyclohexane:ethyl acetate); ν_{max} (film/cm^{-1}) 3388s br. (O-H), 2943w (C-H), 1763m, 1685s (C=O); 1492s; ^1H NMR (400 MHz, $CDCl_3$) 7.88–7.80 (2H, m, Ar*H*), 7.51–7.42 (2H, m, Ar*H*), 7.42–7.35 (1H, m, Ar*H*), 4.98 (1H, t, *J* = 6.1, COC*H*), 4.28 (1H, dd, *J* = 9.7, 3.0, C*H*OH), 3.77 (1H, dd, *J* = 10.0, 6.1, 1.8, COCHC*H*), 3.50 (1H, d, *J* = 10.0, PhCC*H*C(O)), 3.06 (3H, s, NCH_3), 2.57 (1H, dddd, *J* = 13.8, 9.7, 6.1, 1.8, H_a), 1.96 (1H, br. s, O*H*), 1.63 (1H, dd, *J* = 13.8, 3.0 H_b); ^{13}C NMR (100 MHz, $CDCl_3$) 175.4 (*C*(O)), 175.1 (*C*(O)), 138.3 (*Ar*), 128.8 (*Ar*), 128.4 (*Ar*), 125.7 (*Ar*), 91.8 (Ph*C*), 79.5 (*C*OH), 77.7 (CO*C*H), 53.0 (COCH*C*H), 52.5 (PhC*C*HC(O)), 36.1 (*C*H_a), 25.0 (NCH_3); HRMS (CI^+) found $[M+H]^+$ 274.1075; $C_{15}H_{16}NO_4$ requires 274.1079.

5-Ethoxy-2-methyl-4-phenylisoindoline-1,3-dione 11

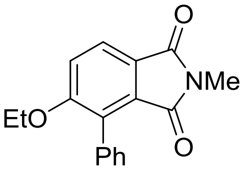

A solution of 4,4-diethoxy-1-phenylbut-2-yn-1-ol **1f** (100 mg, 0.427 mmol) in ethanol (1.0 mL) was treated with *N*-methylmaleimide (57 mg, 0.51 mmol) and [Bis(trifluoromethanesulfonyl)imidate](triphenylphosphine)gold(I) (2:1) toluene adduct (7 mg, 0.043 mmol, 2 mol% [Au]) at room temperature. The resulting solution was stirred at room temperature for 9 h before it was filtered through a silica plug, eluting with ethyl acetate, and the eluent concentrated to give the crude intermediate. This was then treated with ethanol (0.2 mL) and methanesulfonic acid (2.0 mL) at room temperature and stirred for 16 h. The reaction was then diluted with water (30 mL) and ethyl acetate (30 mL) and the aquous extract washed ethyl aceteate (3 × 20 mL). The combined organic extracts were then washed with 10% aqueous K_2CO_3 (50 mL) and brine, dried ($MgSO_4$) and concentrated. The concentrated material was then treated with ethanol (0.2 mL) and methanesulfonic acid (2.0 mL) at room temperature and stirred for 16 h. The reaction was then diluted with water (30 mL) and ethyl acetate (30 mL) and the aquous extract washed ethyl aceteate (3 × 20 mL). The combined organic extracts were then washed with 10% aqueous K_2CO_3 (50 mL) and brine, dried ($MgSO_4$) and concentrated to give the crude product. This was purified by flash column chromatography (7:1 petrol 60–80 °C: ethyl acetate) to give 5-ethoxy-2-methyl-4-phenylisoindoline-1,3-dione **11** as a white crystalline solid (55 mg, 0.20 mmol, 47%); m.p. 93–95 °C; R_f = 0.31 (7:1 petrol 60–80 °C:ethyl acetate); ν_{max} (film/cm^{-1}) 2922s (C-H), 1764m (C=O), 1709s (C=O), 1466s; ^{1}H NMR (600 MHz, $CDCl_3$) 7.80 (1H, d, *J* = 8.2, Ar*H*), 7.46–7.40 (3H, m, Ar*H*), 7.38–7.36 (2H, m, Ar*H*), 7.15 (1H, d, *J* = 8.2, Ar*H*), 4.09 (2H, q, *J* = 6.8, OCH_2), 3.07 (3H, s, NCH_3), 1.32 (2H, t, *J* = 6.8, CH_2CH_3); ^{13}C NMR (150 MHz, $CDCl_3$) 168.2 (*C*(O)), 167.8 (*C*(O)), 161.2 (*Ar*), 132.0 (*Ar*), 130.5 (*Ar*), 130.3 (*Ar*), 130.2 (*Ar*), 128.3 (*Ar*), 127.7 (*Ar*), 124.3 (*Ar*), 124.0 (*Ar*), 115.5 (*Ar*), 65.1 (OCH_2), 23.9 (NCH_3), 14.5 (CH_2CH_3); HRMS (CI$^+$) found [M]$^+$ 281.1047; $C_{17}H_{15}NO_3$ requires 281.1052.

(1*R*,2*S*,3*R*,4*R*)-Dimethyl 6-ethoxy-1-phenethyl-7-oxabicyclo[2.2.1]hept-5-ene-2,3-dicarboxylate *endo*-13a and Dimethyl (1*R*,2*R*,3*S*,4*R*)-6-ethoxy-1-phenethyl-7-oxabicyclo[2.2.1]hept-5-ene-2,3-dicarboxylate *exo*-13a

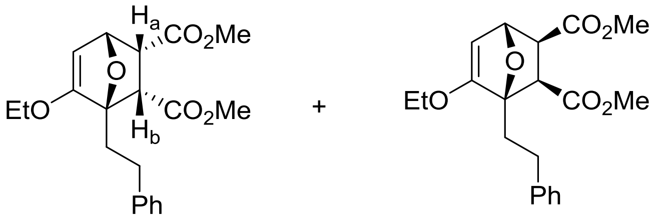

endo-**13a** *exo*-**13a**

Prepared from 3-ethoxy-2-phenethylfuran **2a** (108 mg, 0.500 mmol) and dimethyl maleate according to the General Cycloaddition Procedure over 3 days to give the crude product (3-*endo*-**13b**: 3-*exo*-**13b** = 12:1), which was purified by flash column chromatography (0 to 100% cyclohexane: TBME)to give the title compounds **13a** as a colourless oil (125 mg, 0.347 mmol, 69%, *endo*-**13a**:*exo*-**13a** = 12:1); R_f = 0.32 (3:1 cyclohexane:ethyl acetate); ν_{max} (film/cm^{-1}) 2951s (C-H), 1739s (C=O), 1630s, 1435s; ^{1}H NMR (400 MHz, $CDCl_3$, *endo*-**13a**) 7.35–7.19 (5H, m, Ar*H*), 5.34 (1H, d, *J* = 2.0, C=C*H*), 5.03 (1H, dd, *J* = 2.0, 3.9, COC*H*), 3.91–3.84 (1H, m, OC*H*H'), 3.78–3.72 (1H, m, OCH*H*'), 3.65 (3H, s, OCH_3), 3.64 (3H, s, OCH_3), 3.49 (1H, dd, *J* = 9.8, 3.9, CH_a), 3.31 (1H, dd, *J* = 9.8, CH_b), 2.88–2.74 (2H, m, CH_2Ph), 2.46–2.38 (1H, m, C*H*H'CH_2Ph), 2.20–2.12 (1H, m, CH*H*'CH_2Ph), 1.30 (3H, t, *J* = 7.1, CH_2CH_3); ^{1}H NMR (400 MHz, $CDCl_3$, *exo*-**13a**) the following peaks in the ^{1}H NMR spectra indicate the presence of *exo*-**13a**; 5.19 (1H, d, *J* = 2.0, COC*H*), 5.11 ((1H, d, *J* = 2.0, C=C*H*). The remaining resonances were obscured by the major diastereomers and could not be full assigned; ^{13}C NMR (100 MHz, $CDCl_3$, *endo*-**13a**) 171.2 (*C*(O)), 170.1 (*C*(O), 163.0 (*C*OEt), 142.1 (*Ar*), 128.4 (*Ar*), 125.9 (*Ar*), 99.2 (C=*C*H), 90.4 (*C*OCH), 78.3 (CO*C*H), 66.0 (OCH_2), 51.8 (*C*H or CH_3), 51.6 (*C*H or CH_3), 51.5 (*C*H or CH_3), 50.9 (*C*H or CH_3), 31.4 (CH_2), 30.6 (CH_2), 14.4 (CH_2CH_3); HRMS (CI$^+$) found [M+H]$^+$ 361.1654; $C_{20}H_{25}O_6$ requires 361.1651.

(1*R*,2*R*,3*R*,4*R*)-Dimethyl 6-oxo-1-phenethyl-7-oxabicyclo[2.2.1]heptane-2,3-dicarboxylate 3-*endo*-13b and (1*R*,2*S*,3*S*,4*R*)-Dimethyl 6-oxo-1-phenethyl-7-oxabicyclo[2.2.1]heptane-2,3-dicarboxylate 3-*exo*-13b

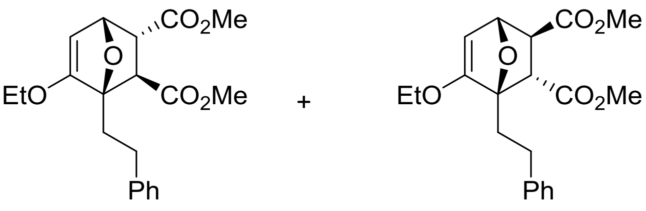

3-*endo*-**13b** 3-*exo*-**13b**

Prepared from 3-ethoxy-2-phenethylfuran **2a** (108 mg, 0.500 mmol) and dimethyl fumarate according to the General Cycloaddition Procedure over 4 h to give the crude product (3-*endo*-**13b**: 3-*exo*-**13b** = 20:80), which was purified by flash column chromatography (0 to 100% cyclohexane: TBME) to give to give the title compounds **13b** as a colourless oil (139 mg, 0.386 mmol, 77%; 3-*endo*-**13b**: 3-*exo*-**13b** = 20:80); R_f = 0.39 (2:1 cyclohexane:ethyl acetate); ν_{max} (film/cm^{-1}) 2952s (C-H), 1736s (C=O), 1630s, 1436s; ^{1}H NMR (400 MHz, DMSO-d$_6$) 7.35–7.18 (5H, m, Ar*H* 3-*endo*-**13b**; 5H, m, Ar*H* 3-*exo*-**13b**), 5.20 (1H, dd, *J* = 4.5, 2.0, COC*H* 3-*endo*-**13b**), 5.18 (1H, d, *J* = 2.0, COC*H* 3-*exo*-**13b**), 5.11 (1H, d, *J* = 2.0, C=C*H* 3-*endo*-**13b**), 5.01 (1H, br. s, C=C*H* 3-*exo*-**13b**), 3.94–3.55 (6H, m, 2 × CO_2CH_3 3-*endo*-**13b**; 6H, m, 2 × CO_2CH_3 3-*exo*-**13b**; 2H, m, OCHC*H*C*H* 3-*endo*-**13b**; 2H, m, OC*H*$_2$ 3-*endo*-**13b**; 2H, m, OC*H*$_2$ 3-*exo*-**13b**), 3.20 (1H, d, *J* = 3.9, C*H*CO_2Me 3-*exo*-**13b**), 3.06 (1H, d, *J* = 3.9, C*H*CO_2Me 3-*exo*-**13b**), 2.78–2.69 (1H, m, PhC*H*H' 3-*exo*-**13b**), 2.66–2.54 (2H, m, PhC*H*$_2$ 3-*endo*-**13b**; 1H, m, PhCH*H*' 3-*exo*-**13b**), 2.35–2.26 (1H, m, C*H*H'CH_2Ph 3-*exo*-**13b**), 2.20–2.08 (1H, m, C*H*H'CH_2Ph 3-*endo*-**13b**; 1H, m, CH*H*'CH_2Ph 3-*exo*-**13b**), 1.78–1.69 (1H, m, CH*H*'CH_2Ph 3-*endo*-**13b**), 1.25 (3H, t, *J* = 7.0, CH_2CH_3 3-*endo*-**13b**), 1.19 (3H, t, *J* = 7.1, CH_2CH_3 3-*exo*-**13b**);^{13}C NMR (100 MHz, DMSO-d$_6$) 172.7 (*C*(O)), 172.1 (*C*(O)), 171.3 (*C*(O)), 170.8 (*C*(O)), 165.6 (*C*OEt), 163.4 (*C*OEt), 142.1 (*Ar*), 142.0 (*Ar*), 128.9 (*Ar*), 128.8 (*Ar*), 128.6 (*Ar*), 128.5 (*Ar*), 126.4 (*Ar*), 126.3 (*Ar*), 100.3 (C=*C*H), 99.3 (C=*C*H), 90.7 (*C*OCH), 89.4 (*C*OCH), 80.6 (CO*C*H), 78.4 (CO*C*H), 66.9 (O*C*H$_2$), 66.4 (O*C*H$_2$), 54.2 (*C*H or *C*H$_3$), 52.6 (*C*H or *C*H$_3$), 52.5 (*C*H or *C*H$_3$), 52.4 (*C*H or *C*H$_3$), 50.6 (*C*H), 50.4 (*C*H), 31.2 (CH$_2$), 31.0 (CH$_2$), 30.6 (CH$_2$), 30.0 (CH$_2$), 14.6 (CH_2CH_3), 14.3 (CH_2CH_3); HRMS (CI$^+$) found [M+H]$^+$ 361.1658; $C_{20}H_{25}O_6$ requires 361.1651.

(1*R*,2*R*,3*R*,4*R*)-Diethyl 6-oxo-1-phenethyl-7-oxabicyclo[2.2.1]heptane-2,3-dicarboxylate 3-*endo*-13c and (1*R*,2*S*,3*S*,4*R*)-Diethyl 6-oxo-1-phenethyl-7-oxabicyclo[2.2.1]heptane-2,3-dicarboxylate 3-*exo*-13c

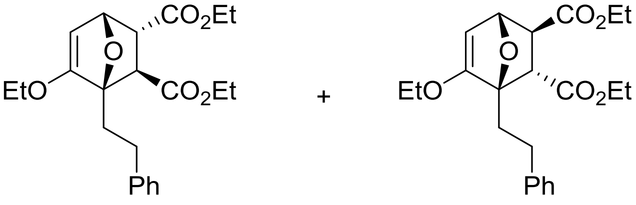

3-*endo*-**13c** 3-*exo*-**13c**

Prepared from 3-ethoxy-2-phenethylfuran **2a** (108 mg, 0.500 mmol) and diethyl fumarate according to the General Cycloaddition Procedure over 4 h to give the crude product (3-*endo*-**13c**: 3-*exo*-**13c** = 15:85), which was purified by flash column chromatography (0 to 100% cyclohexane: TBME) to give the title compounds **13c** as a colourless oil (172 mg, 0.443 mmol, 89%, 3-*endo*-**13c**: 3-*exo*-**13c** = 15:85); R_f = 0.83 (1:1 cyclohexane:ethyl acetate); ν_{max}

(film/cm^{-1}) 2981s (C-H), 1731s (C=O), 1629s; ^{1}H NMR (400 MHz, MeOH-d$_4$) 7.31–7.14 (5H, m, Ar*H* 3-*endo*-**13c**; 5H, m, Ar*H* 3-*exo*-**13c**), 5.18 (1H, d, *J* = 2.0, C=C*H* 3-*exo*-**13c**), 5.14 (1H, dd, *J* = 4.4, 2.0, COC*H* 3-*endo*-**13c**), 5.11–5.08 (1H, m, C=C*H* 3-*endo*-**13c**; 1H, m, COC*H* 3-*exo*-**13c**), 4.25–4.03 (4H, m, CO_2CH_2 3-*endo*-**13c**; 4H, m, CO_2CH_2 3-*exo*-**13c**), 3.87–3.76 (1H, m, OCHC*H*CH 3-*endo*-**13c**; 2H, m, C=COCH_2 3-*endo*-**13c**; 2H, m, C=COCH_2 3-*exo*-**13c**), 3.29 (1H, d, *J* = 3.7, C*H*C(O) 3-*exo*-**13c**), 3.14 (1H, d, *J* = 3.7, C*H*C(O) 3-*exo*-**13c**), 2.92 (1H, d, *J* = 4.4, OCHCHC*H* 3-*endo*-**13c**), 2.86–2.65 (2H, m, CH_2Ph 3-*endo*-**13c**; 2H, m, CH_2Ph 3-*exo*-**13c**), 2.46–2.39 (1H, m, C*H*H'CH_2Ph 3-*exo*-**13c**), 2.29–2.17 (1H, m, C*H*H'CH_2Ph 3-*endo*-**13c**; CH*H'*CH_2Ph 3-*exo*-**13c**), 1.86–1.78 (1H, m, CH*H'*CH_2Ph 3-*endo*-**13c**), 1.38–1.20 (9H, m, CH_2CH_3 3-*endo*-**13c**; 9H, m, CH_2CH_3 3-*exo*-**13c**); ^{13}C NMR (100 MHz, $CDCl_3$) 172.6 (*C*(O)), 172.2 (*C*(O)), 171.0 (*C*(O)), 170.5 (*C*(O)), 165.9 (*C*OEt), 162.8 (*C*OEt), 142.0 (*Ar*), 141.9 (*Ar*), 128.1 (*Ar*), 128.0 (*Ar*), 127.9 (*Ar*), 127.8 (*Ar*), 125.6 (*Ar*), 125.5 (*Ar*), 99.2 (C=*C*H), 98.2 (C=*C*H), 90.9 (*C*OCH), 89.4 (*C*OCH), 80.9 (CO*C*H), 78.5 (CO*C*H), 66.4 (OCH_2), 65.9 (OCH_2), 60.8 (OCH_2), 60.8 (OCH_2), 60.7 (OCH_2), 60.6 (OCH_2), 54.3 (*C*HC(O)), 52.6 (*C*HC(O)), 50.2 (*C*HC(O)), 50.1 (*C*HC(O)), 31.2 (*C*H_2), 30.6 (*C*H_2), 30.2 (*C*H_2), 30.0 (*C*H_2), 13.3 (CH_2CH_3), 13.2 (CH_2CH_3), 13.2 (CH_2CH_3), 13.2 (CH_2CH_3); HRMS (CI$^+$) found $[M+H]^+$ 389.1967; $C_{22}H_{29}O_6$ requires 389.1964.

(1*R*,4*R*,5*R*)-1-Phenethyl-5-propionyl-7-oxabicyclo[2.2.1]heptan-2-one *endo*-13d, (1*R*,4*R*,5*S*)-1-Phenethyl-5-propionyl-7-oxabicyclo[2.2.1]heptan-2-one *exo*-13d and (1R,4S)-1-phenethyl-6-propionyl-7-oxabicyclo[2.2.1]heptan-2-one 13d'

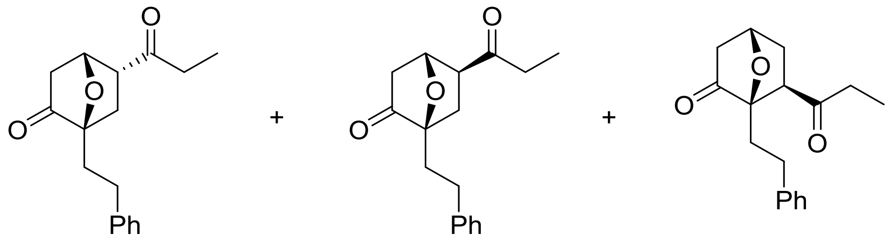

endo-**13d** *exo*-**13d** *exo*-**13d'**

Pent-1-en-3-one (49 μl, 0.50 mmol) was added to a stirring solution of 3-ethoxy-2-phenethylfuran **2a** (54 mg, 0.25 mmol) in dimethylcarbonate (0.25 mL) in a sealed tube at room temperature. The resulting reaction mixture was stirred at 80 °C for 16 h before the reaction was allowed to cool to room temperature. The reaction mixture was concentrated to give the crude product (*endo*-**13d**: *exo*-**13d**: *exo*-**13d'** = 60:40:5), which was purified by flash column chromatography (aminopropyl cartridge; 0 to 100% cyclohexane: TBME) to give a mixture of the title compounds as a colourless oil (22 mg, 0.081 mmol, *endo*-**13d**: *exo*-**13d**: *exo*-**13d'** = 10:3:1, fraction A). Further elution of the column gave a second fraction of the title compounds as a colourless oil (19 mg, 0.070 mmol, 28%, *endo*-**13d**: *exo*-**13d**: *exo*-**13d'** = 1:12:1, fraction B); R_f = 0.47 (4:1 hexane:ethyl acetate); ν_{max} (film/cm^{-1}) 2939s (C-H), 1756s (C=O), 1715s (C=O), 1456s; HRMS (CI$^+$) found $[M+H]^+$ 273.1493; $C_{17}H_{21}O_3$ requires 273.1491.

(1*R*,4*R*,5*R*)-1-Phenethyl-5-propionyl-7-oxabicyclo[2.2.1]heptan-2-one *endo*-13d

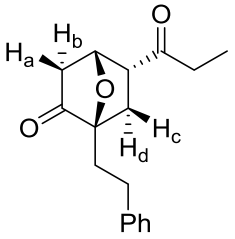

^{1}H NMR (600 MHz, DMSO-d$_6$) 7.30–7.26 (2H, m, Ar*H*), 7.22–7.16 (3H, m, Ar*H*), 5.13 (1H, t, *J* = 5.1, COC*H*), 3.60–3.57 (1H, m, C*H*C(O)), 2.76–2.70 (1H, m, PhC*H*H'), 2.60–2.45 (3H, m, CH_2CH_3; PhCH*H'*), 2.48 (1H, dd, *J* = 17.7, 5.1, *H*$_a$), 2.09–1.96 (2H, m, PhCH_2C*H*$_2$), 1.96 (1H, d, *J* = 17.7, *H*$_b$), 1.93 (1H, dd, *J* = 13.2, 4.9, *H*$_d$), 1.86

(1H, dd, *J* = 13.2, 10.5, H_c), 0.93 (3H, t, *J* = 7.2, CH_2CH_3); ^{13}C NMR (150 MHz, DMSO-d_6) 211.0 (*C*(O)), 208.7 (*C*(O)), 141.6 (*Ar*), 128.4 (*Ar*), 128.1 (*Ar*), 125.9 (*Ar*), 88.5 (*C*OCH), 74.9 (CO*C*H), 53.9 (*C*HC(O)Et), 40.9 (*C*H_a), 35.5 (*C*H_2CH_3), 30.8 (*C*H_c), 30.5 ($PhCH_2CH_2$), 29.8 ($PhCH_2$), 7.4 (CH_2CH_3).

(1*R*,4*R*,5*S*)-1-Phenethyl-5-propionyl-7-oxabicyclo[2.2.1]heptan-2-one *exo*-13d

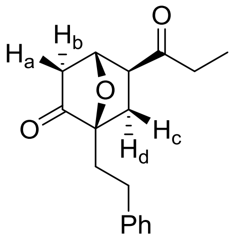

^{1}H NMR (600 MHz, DMSO-d_6) 7.30–7.26 (2H, m, Ar*H*), 7.22–7.16 (3H, m, Ar*H*), (1H, d, *J* = 5.0, COC*H*), 3.16 (1H, dd, *J* = 9.0, 4.9, C*H*C(O)), 2.76–2.70 (1H, m, PhC*H*H'), 2.60–2.45 (3H, m, CH_2CH$_3$; PhCH*H*'), 2.52 (1H, dd, *J* = 17.3, 5.0, H_a), 2.09–1.96 (2H, m, PhCH$_2$CH_2), 2.34 (1H, d, *J* = 17.3, H_b), 2.10 (1H, dd, *J* = 13.2, 4.9, H_c), 1.68 (1H, dd, *J* = 13.2, 9.0, H_d), 0.96 (3H, t, *J* = 7.2, CH$_2$CH_3); ^{1}H NMR (400 MHz, MeOH-d_4) 7.34–7.11 (5H, m, Ar*H*), 5.02 (1H, d, *J* = 6.1, COC*H*), 3.15 (1H, dd, *J* = 9.0, 4.9, C*H*C(O)), 2.81 (1H, ddd, *J* = 13.6, 11.7, 5.5, PhC*H*H'), 2.74–2.49 (4H, m, CH_2CH$_3$; PhCH*H*'; H_a), 2.30 (1H, d, *J* = 17.6, H_b), 2.20–2.02 (3H, m, PhCH$_2$CH_2; H_c), 1.80 (1H, dd, *J* = 13.2, 9.0, H_d), 1.08 (3H, t, *J* = 7.2, CH$_2$CH_3); ^{13}C NMR (150 MHz, MeOH-d_4) 212.0 (*C*(O)), 209.6 (*C*(O)), 141.8 (*Ar*), 128.0 (*Ar*), 127.9 (*Ar*), 125.6 (*Ar*), 87.7 (*C*OCH), 76.3 (CO*C*H), 54.2 (*C*HC(O)), 43.7 (*C*H_a), 33.5 (*C*H$_2$CH$_3$), 30.8 (*C*H_c), 30.5 (PhCH$_2$*C*H$_2$), 30.0 (Ph*C*H$_2$), 6.7 (CH$_2$*C*H$_3$).

(1*R*,4*S*)-1-Phenethyl-6-propionyl-7-oxabicyclo[2.2.1]heptan-2-one *exo*-13d'

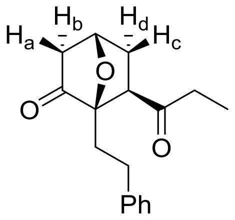

^{1}H NMR (600 MHz, DMSO-d_6) 4.94 (1H, t, *J* = 5.7, COC*H*), 2.90 (1H, dd, *J* = 8.7, 5.3, C*H*C(O)), 2.50 (1H, dd, *J* = 17.7, 5.7, H_a), 2.29 (1H, d, *J* = 17.7, H_b), 2.21–2.17 (1H, m, H_c), 1.96 (1H, dd, *J* = 12.4, 8.7, H_d), 0.87 (3H, t, *J* = 7.2, CH$_2$CH_3); remaining peaks obscured by the major products.

(1*R*,2*R*,4*R*)-Ethyl 5-oxo-4-phenethyl-7-oxabicyclo[2.2.1]heptane-2-carboxylate 13e

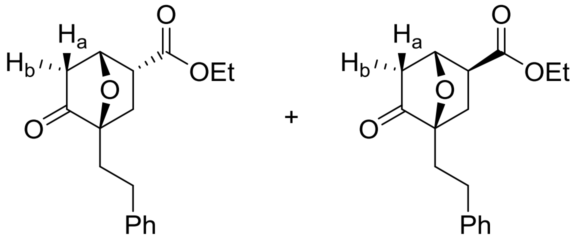

endo-**13e** *exo*-**13e**

A solution of HfCl$_4$ (1.6 mg, 2 mol%) in dimethyl carbonate (60 mL) was added dropwise to a stirring solution of ethyl acrylate (41 µL, 38 mg, 0.38 mmol) and 3-ethoxy-2-phenethylfuran **2a** (54 mg, 0.25 mmol) in dimethylcarbonate (0.18 mL). The resulting solution was stirred at room temperature for 6 h before it was loaded onto a strong cationic exchange (SCX) cartridge (10 g), which was then flushed with ethyl acetate. The eluent was concentrated to give the title compounds **13e** as a colourless oil (64 mg, 0.22 mmol, 89%, *endo*-**13e**:*exo*-**13e** = 70:30; no evidence for a minor regioisomer); R_f = 0.73 (1:1 cyclohexane:ethyl acetate); ν_{max} (film/cm^{-1}) 2938s (C-

H), 1762s (C=O), 1730s (C=O), 1604m, 1493m, 1454s; HRMS (ESI^+) found $[M+H]^+$ 289.1437; $C_{17}H_{21}O_4$ requires 289.1434. In order to aid characterisation a sample of the mixed product was separated by Mass Directed Automated Purification to give the two diastereoisomers.

Ethyl (1*R*,2*R*,4*R*)-5-oxo-4-phenethyl-7-oxabicyclo[2.2.1]heptane-2-carboxylate *endo*-13e: ^{1}H NMR (600 MHz, $CDCl_3$) 7.35–7.21 (2H, m, Ar*H*), 7.26 (2H, d, *J* = 7.0, Ar*H*), 7.25–7.22 (1H, m, Ar*H*), 5.01 (1H, t, *J* = 5.4, COC*H*), 4.22 (2H, q, *J* = 7.2, OCH_2), 3.40 (1H, dt, *J* = 11.2, 5.4, C*H*CO_2Et), 2.85 (1H, td, *J* = 12.9, 5.0, PhC*H*H'), 2.71 (1H, td, *J* = 12.9, 5.0, PhCH*H*'), 2.57 (1H, dd, *J* = 17.8, 5.4, C(O)C*H*H'), 2.38 (1H, d, *J* = 17.8, C(O)CH*H*'), 2.29 (1H, ddd, *J* = 14.4, 12.9, 5.0, PhCH_2C*H*H'), 2.18–2.12 (2H, m, PhCH_2CH*H*'; C*H*H'CHCO_2Et), 2.08–2.02 (1H, m, CH*H*'CHCO_2Et), 1.32 (3H, t, *J* = 7.2, CH_2CH_3); ^{13}C NMR (150 MHz, $CDCl_3$) 210.2 (*C*O_2Et), 171.3 (*C*(O)), 141.6 (*Ar*), 128.4 (*Ar*), 128.3 (*Ar*), 126.0 (*Ar*), 88.9 (*C*OCH), 75.6 (COC*H*), 61.2 (OCH_2), 47.3 (*C*HCO_2Et), 41.4 (C(O)CH_2), 32.8 (CH_2CHCO_2Et), 30.9 (CH_2CH_2Ph), 30.2 (CH_2Ph), 14.2 (CH_2CH_3); No ROE between H_b and C*H*CO_2Et.

Ethyl (1*R*,2*S*,4*R*)-5-oxo-4-phenethyl-7-oxabicyclo[2.2.1]heptane-2-carboxylate *exo*-13e: ^{1}H NMR (600 MHz, $CDCl_3$) 7.31–7.27 (2H, m, Ar*H*), 7.24–7.21 (2H, m, Ar*H*), 7.21–7.17 (1H, m, Ar*H*), 5.10 (1H, d, *J* = 6.1, COC*H*), 4.23 (2H, q, *J* = 7.2, OCH_2), 2.88 (1H, dd, *J* = 9.2, 4.8, C*H*CO_2Et), 2.83 (1H, td, *J* = 12.9, 5.0, PhC*H*H'), 2.67 (1H, td, *J* = 12.9, 5.0, PhCH*H*'), 2.59 (1H, dd, *J* = 17.2, 6.1, C(O)C*H*H'), 2.28–2.14 (4H, m, PhCH_2CH_2'; C(O)CH*H*'; C*H*H'CHCO_2Et), 1.90 (1H, dd, *J* = 13.6, 9.2, CH*H*'CHCO_2Et), 1.31 (3H, t, *J* = 7.2, CH_2CH_3); (150 MHz, $CDCl_3$) 211.2 (*C*(O)), 172.3 (*C*(O)), 141.6 (*Ar*), 128.4 (*Ar*), 128.4 (*Ar*), 126.0 (*Ar*), 88.0 (*C*OCH), 77.3 (COC*H*), 61.4 (OCH_2), 47.9 (*C*HCO_2Et), 44.2 (C(O)CH_2), 32.4 (CH_2CHCO_2Et), 30.6 (PhCH_2CH_2), 30.3 (PhCH_2), 14.2 (CH_2CH_3); ROE between H_b and C*H*CO_2Et.

4 Biological Testing

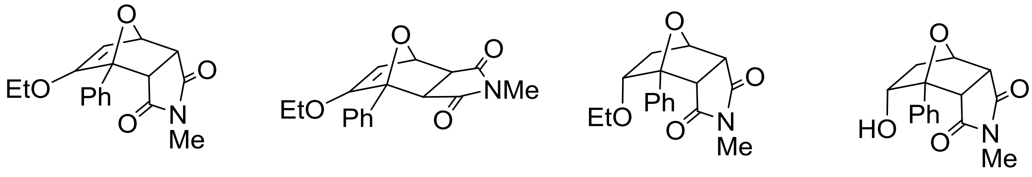

endo-**3f** *exo*-**3f** **7** **10**

Compound	Assay	Parameter	Value/µM
endo-**3f**	KCNH2 (human Ether-à-go-go-Related Gene) Human Antagonist Barracuda Electrophysiology Population Patch Clamp	IC50	>50
exo-**3f**	KCNH2 (human Ether-à-go-go-Related Gene) Human Antagonist Barracuda Electrophysiology Population Patch Clamp	IC50	>50
**7**	KCNH2 (human Ether-à-go-go-Related Gene) Human Antagonist Barracuda Electrophysiology Population Patch Clamp	IC50	>50
**10**	KCNH2 (human Ether-à-go-go-Related Gene) Human Antagonist Barracuda Electrophysiology Population Patch Clamp	IC50	>50
endo-**3f**	Aryl Hydrocarbon Receptor (AhR) Human LS180 CYP1A1 FRET - pEC50 (Abse5)	EC50	>100
exo-**3f**	Aryl Hydrocarbon Receptor (AhR) Human LS180 CYP1A1 FRET - pEC50 (Abse5)	EC50	>100
**7**	Aryl Hydrocarbon Receptor (AhR) Human LS180 CYP1A1 FRET - pEC50 (Abse5)	EC50	>100
**10**	Aryl Hydrocarbon Receptor (AhR) Human LS180 CYP1A1 FRET - pEC50 (Abse5)	EC50	>100

5 Crystallography Data

Structure ccdc_deposit_xstr0272-final: *endo*-3f

Table 1. General and crystallographic data for ***endo*-3f**.

**Empirical formula**	$C_{17}H_{17}NO_4$
**Formula weight / g mol^{-1}**	299.32
**Temperature / K**	150.00(10)
**Crystal system**	triclinic
**Space group**	$P\bar{1}$
a **/ Å**	7.2630(2)
b **/ Å**	10.8264(3)
c **/ Å**	19.9792(4)
α **/ °**	78.833(2)
β **/ °**	89.832(2)
γ **/ °**	71.899(2)
**Volume / Å^3**	1462.22(6)
Z	4
**ρ_{calc}/ g cm^{-3}**	1.36
**μ / mm^{-1}**	0.801
***F*(000)**	632.0
**Crystal size / mm^3**	0.41 × 0.17 × 0.07
**Radiation**	CuK_α (λ = 1.5418 Å)
**2θ range for data collection / °**	8.78 to 133.2
**Index ranges**	$-8 \leq h \leq 8, -12 \leq k \leq 12, -23 \leq l \leq 23$
**Reflections collected**	22496
**Independent reflections**	5146 [R_{int} = 0.0363, R_{sigma} = 0.0235]
**Data / restraints / parameters**	5146 / 0 / 400
**Goodness-of-fit on F^2**	0.998
**Final *R* indexes [I ≥ 2$\sigma(I)$]**	R_1 = 0.0393, wR_2 = 0.0975
**Final *R* indexes [all data]**	R_1 = 0.0453, wR_2 = 0.1016
**Largest diff. peak/hole / e Å^{-3}**	0.23 / -0.20
**CCDC deposition number**	1035038

Structure ccdc_deposit_xstr0273-final: *exo*-3f

Table 2. General and crystallographic data for ***exo*-3f**.

**Empirical formula**	$C_{17}H_{17}NO_4$
**Formula weight / g mol^{-1}**	299.32
**Temperature / K**	150.00(10)
**Crystal system**	monoclinic
**Space group**	$P2_1/c$
***a* / Å**	6.64710(10)
***b* /Å**	14.1775(2)
***c* /Å**	15.4276(2)
***α* /°**	90
***β* /°**	97.1400(10)
***γ* /°**	90
**Volume / Å^3**	1442.61(4)
Z	4
**ρ_{calc} / g cm^{-3}**	1.378
**μ / mm^{-1}**	0.812
***F*(000)**	632.0
**Crystal size / mm^3**	0.26 × 0.15 × 0.09
**Radiation**	CuK_α (λ = 1.5418 Å)
**2θ range for data collection / °**	8.5 to 133.18
**Index ranges**	$-7 \le h \le 7$, $-16 \le k \le 16$, $-18 \le l \le 18$
**Reflections collected**	20945
**Independent reflections**	2532 [R_{int} = 0.0455, R_{sigma} = 0.0194]
**Data / restraints / parameters**	2532 / 0 / 201
**Goodness-of-fit on F^2**	1.063
**Final *R* indexes [$I \ge 2\sigma(I)$]**	R_1 = 0.0372, wR_2 = 0.0976
**Final *R* indexes [all data]**	R_1 = 0.0402, wR_2 = 0.1007
**Largest diff. peak/hole / e Å^{-3}**	0.24 / -0.19
**CCDC deposition number**	1035039

6 DFT Study

Cartesian Coordinates of M06-2X/6-31G(d) stationary points and M06-2X/6-31G(d) energetics.

All calculations were conducted using Gaussian09.[3] Global minima for starting materials and products were located by geometry optimisation of each potential rotamer at the M06-2X/6-31G(d) level. Transition states were located using the QST2[4] method. Solvation energies were obtained by single-point calculations on the gas-phase optimised structures using the IEFPCM method. Reported energies are zero-point exclusive, while enthalpies and free energies are computed at 298 K and include harmonic unscaled vibrational zero point energies. All stationary points were identified as minima or transition states by the presence of respectively zero or one imaginary frequency and transition states were confirmed using IRC (intrinsic reaction coordinate) calculations.[5] Unless otherwise stated, all values for energy are in hartrees and all distances are in Angstroms.

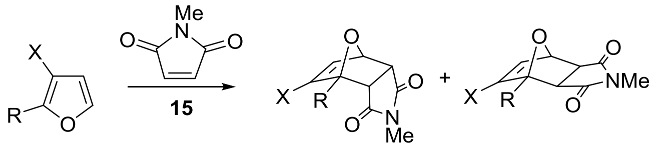

14 *endo*-**16** *exo*-**16**

16	R	X	*endo*				*exo*			
			ΔG	$\Delta G^{\ddagger}$	$\Delta G_{(ether)}$	$\Delta G^{\ddagger}_{(ether)}$	ΔG	$\Delta G^{\ddagger}$	$\Delta G_{(ether)}$	$\Delta G^{\ddagger}_{(ether)}$
**16a**	Me	OMe	–41.5	81.1	–40.9	81.1	–47.7	82.1	–46.7	82.7
**16b**	^{c}Pr	OMe	–44.1	75.0	–42.7	77.0	–53.2	78.6	–52.1	78.6
**16c**	Ph	OMe	–34.0	83.6	–31.2	86.9	–28.2	91.4	–27.9	90.4
**16d**	4-MeOPh	OMe	–32.5	76.8	–30.0	80.3	–30.1	85.3	–30.0	84.2
**16e**	4-F_3CPh	OMe	–25.3	85.9	–22.1	89.2	–23.8	96.8	–23.3	95.8
**16f**	Me	H	–11.8	97.9	–10.5	98.5	–16.8	96.2	–16.2	97.0
**16g**	^{c}Pr	H	–12.5	92.2	–9.8	93.0	–10.3	92.8	–18.4	99.4
**16h**	Ph	H	–1.7	101.1	1.9	103.9	0.7	105.5	1.3	104.1
**16i**	4-MeOPh	H	–6.1	95.4	–2.5	98.4	–3.6	98.8	–2.4	97.3
**16j**	4-F_3CPh	H	–0.9	101.6	2.7	104.3	0.9	108.3	1.6	107.1

All values in kJ mol^{-1}. All data is calculated in the gas phase or in diethyl ether (ether) as indicated.

***N*-Methylmaleimide 15**

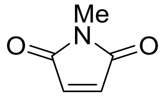

sum of electronic and thermal energies	-398.476048
sum of electronic and thermal enthalpies	-398.475104
sum of electronic and thermal free energies	-398.513021
$E_{solvation}(Et_2O)$	-0.005684

1	N	-0.001197	0.588822	0.000000
2	C	-0.003985	2.035367	0.000000
3	H	0.514005	2.380476	0.896025
4	H	0.514005	2.380476	-0.896025
5	H	-1.024484	2.427919	0.000000
6	C	-0.001574	-0.199850	-1.145733
7	O	0.004050	0.207875	-2.280402
8	C	-0.001574	-0.199850	1.145733
9	O	0.004050	0.207875	2.280402
10	C	-0.001574	-1.627516	0.665459
11	H	0.000871	-2.460217	1.356105
12	C	-0.001574	-1.627516	-0.665459
13	H	0.000871	-2.460217	-1.356105

3-Ethoxy-2-methylfuran 14a

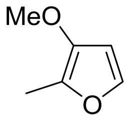

sum of electronic and thermal energies	-383.552490
sum of electronic and thermal enthalpies	-383.551545
sum of electronic and thermal free energies	-383.593955
$E_{solvation}$ (Et_2O)	-0.0031542

1	C	-0.900194	0.512778	0.008208
2	C	0.350750	-0.013764	-0.081114
3	C	0.220144	-1.439499	-0.073449
4	C	-1.112513	-1.672306	0.014996
5	O	-1.798140	-0.509963	0.060025
6	H	0.995954	-2.188736	-0.122677
7	H	-1.701840	-2.574403	0.057668
8	C	-1.404501	1.907738	0.030221
9	H	-2.008599	2.133865	-0.854650
10	H	-2.017577	2.096100	0.917352
11	H	-0.549124	2.586747	0.046709
12	C	2.669484	0.080101	0.148254
13	H	3.462517	0.826706	0.098403
14	H	2.616082	-0.338753	1.160099
15	H	2.885521	-0.722541	-0.566890
16	O	1.470396	0.751303	-0.187364

(3a*S*,4*R*,7*R*,7a*R*)-5-Methoxy-2,4-dimethyl-3a,4,7,7a-tetrahydro-1*H*-4,7-epoxyisoindole-1,3(2*H*)-dione *endo*-16a

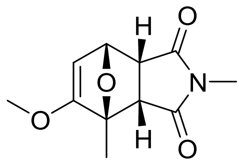

sum of electronic and thermal energies	-782.068107
sum of electronic and thermal enthalpies	-782.067163
sum of electronic and thermal free energies	-782.122796
$E_{solvation}$ (Et_2O)	-0.008594

1	C	1.234390	0.409464	-0.689426
2	C	1.521771	-0.491558	0.506370
3	C	0.596407	-1.721868	-0.984948
4	C	0.670037	-0.351283	-1.635801
5	H	0.226599	-0.074227	-2.581920
6	C	0.128426	-0.799434	1.173576
7	H	0.289224	-1.291790	2.135170
8	C	-0.537292	-1.678784	0.105892
9	H	-0.799527	-2.682107	0.446520
10	H	0.574815	-2.591269	-1.638707
11	O	1.752231	-1.739938	-0.143874
12	C	-0.776319	0.404756	1.336072
13	C	-1.775620	-0.910167	-0.313455
14	N	-1.834432	0.256850	0.442590
15	C	-2.872891	1.247917	0.256861
16	H	-2.807714	1.679890	-0.745390
17	H	-3.853287	0.781774	0.373582
18	H	-2.725453	2.021682	1.010622
19	O	-0.624227	1.347606	2.075228
20	O	-2.582043	-1.201125	-1.164120
21	O	1.459873	1.722660	-0.579620
22	C	0.967852	2.490368	-1.664488
23	H	-0.116389	2.355309	-1.760567
24	H	1.199561	3.531195	-1.443268
25	H	1.453620	2.186229	-2.598909
26	C	2.623262	-0.073676	1.439923
27	H	2.790860	-0.851091	2.189969
28	H	3.549685	0.096312	0.886461
29	H	2.332216	0.852099	1.943931

Transition state: *endo*-16a

sum of electronic and thermal energies	-782.020433
sum of electronic and thermal enthalpies	-782.019489
sum of electronic and thermal free energies	-782.076098
$E_{solvation}$ (Et_2O)	-0.008812

1	C	1.201468	0.506402	-0.765599
2	C	1.851489	-0.295312	0.213214
3	C	0.892953	-1.641538	-1.174963
4	C	0.593743	-0.352565	-1.661074
5	H	-0.099658	-0.126264	-2.458244
6	C	0.102347	-0.834022	1.341695
7	H	0.696455	-1.060200	2.218261
8	C	-0.501633	-1.768509	0.499308
9	H	-0.433416	-2.844433	0.588443
10	H	0.783234	-2.590719	-1.680272
11	O	1.918197	-1.562180	-0.282539
12	C	-0.727703	0.407573	1.325116
13	C	-1.731575	-1.147382	-0.061677
14	N	-1.753328	0.170944	0.406684
15	C	-2.822496	1.096863	0.115668
16	H	-2.542343	2.065787	0.532949
17	H	-2.968750	1.173250	-0.965054
18	H	-3.760045	0.760006	0.567202
19	O	-0.565740	1.443220	1.930232
20	O	-2.568649	-1.613378	-0.801706
21	O	1.159175	1.838855	-0.635018
22	C	0.227320	2.494199	-1.476813
23	H	-0.773629	2.070629	-1.339700
24	H	0.231378	3.543246	-1.184495
25	H	0.523754	2.398819	-2.528306
26	C	2.867711	0.156088	1.203067
27	H	3.085558	-0.642217	1.916032
28	H	3.798813	0.452974	0.709919
29	H	2.466337	1.019595	1.741070

(3a*R*,4*R*,7*R*,7a*S*)-5-Methoxy-2,4-dimethyl-3a,4,7,7a-tetrahydro-1*H*-4,7-epoxyisoindole-1,3(2*H*)-dione *exo*-16a

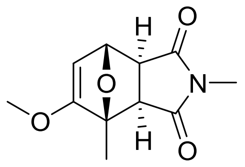

sum of electronic and thermal energies	-782.070568
sum of electronic and thermal enthalpies	-782.069624
sum of electronic and thermal free energies	-782.125139
$E_{solvation}$ (Et_2O)	-0.008444

1	C	2.118162	-0.133880	0.113096
2	C	0.838216	0.604577	0.492282
3	C	0.413745	-1.492471	0.646107
4	C	1.872230	-1.444455	0.222283
5	H	2.487580	-2.294792	-0.035606
6	C	-0.123295	0.389026	-0.737530
7	H	0.329809	0.710377	-1.677380
8	C	-0.449426	-1.111854	-0.600309
9	H	-0.238034	-1.733663	-1.471318
10	H	0.063800	-2.367811	1.192002
11	O	0.264592	-0.306048	1.429147
12	C	-1.453669	1.094238	-0.529992
13	O	-1.667889	2.283106	-0.574144
14	C	-1.928548	-1.161897	-0.258610
15	O	-2.585207	-2.144250	-0.008644
16	N	-2.418036	0.136420	-0.254583
17	C	-3.788107	0.468580	0.076262
18	H	-4.080460	1.346448	-0.500985
19	H	-4.413951	-0.388961	-0.172149
20	H	-3.883010	0.690296	1.142666
21	C	0.991922	1.997681	1.040961
22	H	1.664856	1.976810	1.901481
23	H	1.416804	2.651689	0.275427
24	H	0.024400	2.401265	1.343876
25	C	4.259519	-0.210900	-0.763703
26	H	5.036322	0.482236	-1.084327
27	H	4.630667	-0.840569	0.053039
28	H	3.953378	-0.848725	-1.601120
29	O	3.167203	0.575766	-0.319936

Transition state: *exo*-16a

sum of electronic and thermal energies	-782.018762
sum of electronic and thermal enthalpies	-782.017818
sum of electronic and thermal free energies	-782.075698
$E_{solvation}$ (Et_2O)	-0.008608

1	C	2.095208	-0.076980	0.283140
2	C	0.956039	0.642576	0.736342
3	C	0.507637	-1.464632	0.941467
4	C	1.828325	-1.421197	0.441954
5	H	2.402473	-2.272887	0.107735
6	C	-0.211255	0.230263	-1.120100
7	H	0.489076	0.634013	-1.839830
8	C	-0.522975	-1.113427	-0.910875
9	H	-0.136160	-1.959476	-1.461635
10	H	-0.029960	-2.294280	1.381265
11	O	0.169681	-0.234653	1.412683
12	C	-1.407109	1.032875	-0.732634
13	O	-1.577331	2.231980	-0.793611
14	C	-1.909510	-1.182152	-0.356126
15	O	-2.548763	-2.158591	-0.032754
16	N	-2.346876	0.134743	-0.233404
17	C	-3.601627	0.530521	0.361587
18	H	-4.229068	-0.359130	0.429601
19	H	-3.446520	0.947119	1.361242
20	H	-4.078629	1.285272	-0.266704
21	C	0.887060	2.088258	1.081098
22	H	1.359964	2.271182	2.051727
23	H	1.425661	2.657097	0.319620
24	H	-0.144854	2.441440	1.108226
25	C	4.101832	-0.278577	-0.875844
26	H	4.867292	0.375041	-1.292112
27	H	4.549238	-0.941626	-0.126067
28	H	3.653182	-0.882925	-1.673355
29	O	3.131999	0.565113	-0.282060

2-Cyclopropyl-3-ethoxyfuran 14b

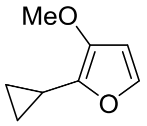

sum of electronic and thermal energies	-460.869780
sum of electronic and thermal enthalpies	-460.868836
sum of electronic and thermal free energies	-460.914854
$E_{solvation}$ (Et_2O)	-0.003280

1	C	-0.283316	-0.744092	0.000005
2	C	0.736251	0.163515	-0.000164
3	C	1.964592	-0.575233	-0.000266
4	C	1.593525	-1.881812	0.000167
5	O	0.247693	-1.997825	0.000073
6	H	2.974274	-0.192728	-0.000454
7	H	2.149271	-2.805985	0.000363
8	C	1.719651	2.282968	0.000288
9	H	1.409845	3.328184	0.000297
10	H	2.322781	2.080604	0.894434
11	H	2.323571	2.080874	-0.893387
12	O	0.538078	1.512437	-0.000349
13	C	-1.749169	-0.617766	0.000096
14	C	-2.409950	0.514286	0.748690
15	C	-2.410051	0.514102	-0.748685
16	H	-2.260268	-1.575109	0.000252
17	H	-1.750712	1.218502	1.244478
18	H	-3.336544	0.297533	1.269078
19	H	-3.336714	0.297227	-1.268899
20	H	-1.750872	1.218189	-1.244739

(3a*S*,4*R*,7*R*,7a*R*)-4-Cyclopropyl-5-methoxy-2-methyl-3a,4,7,7a-tetrahydro-1*H*-4,7-epoxyisoindole-1,3(2*H*)-dione *endo*-16b

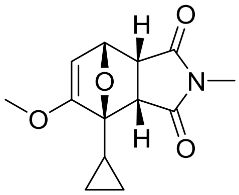

sum of electronic and thermal energies	-859.386565
sum of electronic and thermal enthalpies	-859.385620
sum of electronic and thermal free energies	-859.444660
$E_{\text{solvation}}$ (Et_2O)	-0.008442

1	C	0.543316	0.814050	-0.856033
2	C	1.205310	-0.251314	0.011354
3	C	0.136999	-1.288505	-1.531746
4	C	-0.115637	0.178111	-1.833422
5	H	-0.795178	0.561935	-2.581418
6	C	0.049819	-0.935489	0.834234
7	H	0.491253	-1.574429	1.602073
8	C	-0.714167	-1.682768	-0.268812
9	H	-0.751017	-2.765860	-0.138147
10	H	0.085226	-1.991396	-2.360832
11	O	1.445767	-1.274839	-0.953449
12	C	-0.947865	0.029302	1.441753
13	C	-2.109343	-1.088338	-0.247059
14	N	-2.151376	-0.128003	0.758619
15	C	-3.329083	0.673731	1.013991
16	H	-3.593993	1.245633	0.120988
17	H	-4.169967	0.028704	1.277756
18	H	-3.093099	1.346281	1.839110
19	O	-0.760620	0.822328	2.333186
20	O	-3.041962	-1.347023	-0.969767
21	O	0.605506	2.091752	-0.471308
22	C	-0.237363	2.964102	-1.203600
23	H	-1.281506	2.639354	-1.116365
24	H	-0.113904	3.955682	-0.770631
25	H	0.050368	2.976123	-2.261136
26	C	2.419160	0.143135	0.788679
27	C	3.458044	-0.899632	1.079055
28	C	3.742005	0.195047	0.086959
29	H	2.210191	0.887754	1.551849
30	H	3.285005	-1.893110	0.678476
31	H	3.955891	-0.866173	2.041638
32	H	4.434708	0.980480	0.366937
33	H	3.748962	-0.081284	-0.962052

Transition state: *endo*-16b

sum of electronic and thermal energies	-859.340459
sum of electronic and thermal enthalpies	-859.339515
sum of electronic and thermal free energies	-859.399307
$E_{solvation}$ (Et_2O)	-0.008202

1	C	0.756688	-0.036654	1.078401
2	C	1.372613	0.833123	0.129651
3	C	-0.218281	1.950487	1.077698
4	C	-0.240556	0.682847	1.704319
5	H	-1.000922	0.331185	2.386730
6	C	-0.249054	0.684447	-1.337783
7	H	0.446989	0.907447	-2.135688
8	C	-1.176735	1.562825	-0.768881
9	H	-1.312785	2.600149	-1.046120
10	H	-0.671475	2.867546	1.428132
11	O	0.974538	2.096754	0.424566
12	C	-0.791040	-0.698561	-1.203958
13	C	-2.354261	0.764609	-0.328770
14	N	-2.019042	-0.572990	-0.546609
15	C	-2.901894	-1.671984	-0.235333
16	H	-2.393756	-2.592411	-0.528405
17	H	-3.130770	-1.689188	0.834359
18	H	-3.840713	-1.574308	-0.786777
19	O	-0.302533	-1.757575	-1.537033
20	O	-3.398736	1.130032	0.161892
21	O	1.109365	-1.329356	1.158580
22	C	0.190488	-2.166427	1.840200
23	H	-0.802274	-2.088488	1.385127
24	H	0.567268	-3.182899	1.737449
25	H	0.134963	-1.894487	2.900786
26	C	2.639404	0.674058	-0.605813
27	C	3.732671	-0.227306	-0.081986
28	C	2.943220	-0.638973	-1.283351
29	H	2.941006	1.582536	-1.118378
30	H	3.538564	-0.750724	0.847314
31	H	4.753509	0.111897	-0.219478
32	H	3.417782	-0.584431	-2.256986
33	H	2.205256	-1.426681	-1.172204

(3a*R*,4*R*,7*R*,7a*S*)-4-Cyclopropyl-5-methoxy-2-methyl-3a,4,7,7a-tetrahydro-1*H*-4,7-epoxyisoindole-1,3(2*H*)-dione *exo*-16b

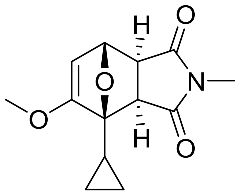

sum of electronic and thermal energies	-859.389262
sum of electronic and thermal enthalpies	-859.388318
sum of electronic and thermal free energies	-859.448121
$E_{solvation}$ (Et_2O)	-0.008580

1	C	2.022104	-0.537623	-0.045007
2	C	0.788001	0.353138	0.063390
3	C	0.288262	-1.511467	0.999047
4	C	1.725562	-1.701979	0.545314
5	H	2.295010	-2.618636	0.606796
6	C	-0.228447	-0.255327	-0.978841
7	H	0.214412	-0.355427	-1.971231
8	C	-0.630601	-1.559617	-0.262375
9	H	-0.525151	-2.480525	-0.837956
10	H	-0.068894	-2.098823	1.844092
11	O	0.225829	-0.111196	1.288910
12	C	-1.506634	0.561402	-1.051894
13	O	-1.674576	1.625806	-1.596910
14	C	-2.081274	-1.347126	0.142821
15	O	-2.765251	-2.093264	0.802015
16	N	-2.494700	-0.119083	-0.353554
17	C	-3.816304	0.433096	-0.142974
18	H	-4.302946	0.611123	-1.104431
19	H	-4.383747	-0.290345	0.442704
20	H	-3.740693	1.381346	0.394570
21	C	4.126611	-1.040442	-0.866544
22	H	4.922055	-0.551171	-1.427098
23	H	4.493457	-1.340666	0.121659
24	H	3.774454	-1.927667	-1.405943
25	O	3.078584	-0.095498	-0.735027
26	C	0.994158	1.834592	0.043234
27	C	0.077657	2.703160	0.850065
28	C	1.502525	2.506232	1.285565
29	H	1.330870	2.215293	-0.916220
30	H	-0.691118	2.202249	1.429818
31	H	-0.214762	3.653792	0.419467
32	H	2.200856	3.327090	1.167425
33	H	1.672685	1.870927	2.148512

Transition state: *exo*-16b

sum of electronic and thermal energies	-859.337758
sum of electronic and thermal enthalpies	-859.336813
sum of electronic and thermal free energies	-859.397943
$E_{solvation}$ (Et_2O)	-0.0089742

1	C	-2.070993	-0.316821	-0.151891
2	C	-0.926251	0.508189	-0.326435
3	C	-0.523487	-1.365684	-1.335198
4	C	-1.836996	-1.496714	-0.828723
5	H	-2.425109	-2.402682	-0.832211
6	C	0.223644	-0.678944	1.230063
7	H	-0.496551	-0.670460	2.037977
8	C	0.576717	-1.763568	0.425465
9	H	0.263928	-2.790366	0.558338
10	H	-0.022941	-1.953975	-2.092876
11	O	-0.171289	-0.046684	-1.302435
12	C	1.374072	0.261162	1.272191
13	O	1.510056	1.275385	1.919572
14	C	1.945686	-1.485196	-0.119823
15	O	2.612793	-2.157404	-0.874900
16	N	2.323687	-0.243847	0.376620
17	C	3.553051	0.424715	0.023479
18	H	3.572084	0.652046	-1.046234
19	H	3.596988	1.348146	0.603216
20	H	4.411819	-0.207123	0.262864
21	C	-4.049634	-0.932837	0.901734
22	H	-4.785569	-0.488302	1.570532
23	H	-4.537458	-1.246598	-0.028726
24	H	-3.588321	-1.804306	1.381648
25	O	-3.081624	0.067931	0.645542
26	C	-0.787051	1.950693	-0.075941
27	C	0.442204	2.666214	-0.554543
28	C	-0.862234	2.906946	-1.247535
29	H	-1.222666	2.270191	0.864906
30	H	1.185273	2.068465	-1.074103
31	H	0.845136	3.432270	0.096809
32	H	-1.372935	3.850088	-1.088231
33	H	-1.001335	2.466791	-2.229548

3-Methoxy-2-(4-methoxyphenyl)furan 14c

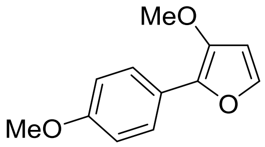

sum of electronic and thermal energies	-689.604980
sum of electronic and thermal enthalpies	-689.604036
sum of electronic and thermal free energies	-689.658369
$E_{solvation}$ (Et_2O)	-0.005577

1	C	-3.013389	-1.985124	-0.000275
2	C	-3.575705	-0.748528	0.000117
3	C	-2.472919	0.165115	0.000071
4	C	-1.321669	-0.581014	-0.000210
5	O	-1.669050	-1.901961	-0.000466
6	H	-3.427012	-2.981420	-0.000495
7	H	-4.630722	-0.520041	0.000359
8	C	0.095819	-0.251133	-0.000069
9	C	1.062194	-1.259267	0.000480
10	C	0.526105	1.087027	-0.000493
11	C	2.421930	-0.958202	0.000526
12	H	0.747856	-2.297784	0.000894
13	C	1.874822	1.392637	-0.000417
14	H	-0.207668	1.885242	-0.000859
15	C	2.833993	0.374333	0.000009
16	H	3.142371	-1.767939	0.001097
17	H	2.218353	2.421880	-0.000681
18	O	-2.499341	1.521742	0.000307
19	C	-3.787950	2.098095	0.000297
20	H	-4.351421	1.805606	0.894761
21	H	-3.641981	3.178231	0.000413
22	H	-4.351340	1.805752	-0.894267
23	O	4.132563	0.780372	-0.000128
24	C	5.123412	-0.221070	0.000090
25	H	6.081423	0.299295	-0.000504
26	H	5.053653	-0.853190	0.894151
27	H	5.053253	-0.854066	-0.893338

(3a*S*,4*S*,7*R*,7a*R*)-5-Methoxy-4-(4-methoxyphenyl)-2-methyl-3a,4,7,7a-tetrahydro-1*H*-4,7-epoxyisoindole-1,3(2*H*)-dione *endo*-16c

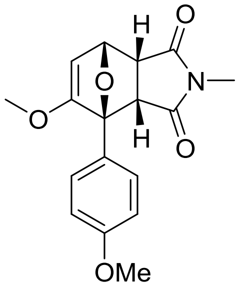

sum of electronic and thermal energies	-1088.114455
sum of electronic and thermal enthalpies	-1088.113510
sum of electronic and thermal free energies	-1088.183779
$E_{solvation}$ (Et_2O)	-0.010285

1	C	0.856190	-0.714314	1.155471
2	C	0.132023	-0.783574	-0.189492
3	C	1.862015	-2.045841	-0.350766
4	C	1.932079	-1.507480	1.067021
5	H	2.751066	-1.649002	1.758298
6	C	1.034778	0.041914	-1.193955
7	H	0.480119	0.211973	-2.119206
8	C	2.250948	-0.889689	-1.340008
9	H	2.401743	-1.274885	-2.350023
10	H	2.339573	-3.002824	-0.551824
11	O	0.458926	-2.109188	-0.602701
12	C	1.603728	1.352154	-0.669767
13	C	3.439967	-0.069223	-0.889687
14	N	2.976161	1.189944	-0.524239
15	C	3.856598	2.212230	0.001313
16	H	4.306685	1.872546	0.937689
17	H	4.654135	2.416716	-0.716011
18	H	3.257858	3.107117	0.171521
19	O	1.010308	2.373355	-0.409711
20	O	4.596376	-0.408348	-0.813455
21	C	-1.342611	-0.524074	-0.203267
22	C	-1.843001	0.768539	-0.317350
23	C	-2.233296	-1.593347	-0.061107
24	C	-3.217572	1.004482	-0.298229
25	H	-1.157398	1.606643	-0.416115
26	C	-3.598940	-1.370146	-0.047556
27	H	-1.841443	-2.601208	0.027625
28	C	-4.099026	-0.068327	-0.165518
29	H	-3.581925	2.020886	-0.389290
30	H	-4.307117	-2.185799	0.053447
31	O	0.424156	0.148527	2.076844
32	C	1.286730	0.293104	3.191463
33	H	2.270580	0.650717	2.863791
34	H	0.822977	1.022835	3.853590
35	H	1.404160	-0.665223	3.710237
36	O	-5.452920	0.046942	-0.141605
37	C	-5.997130	1.344328	-0.233571
38	H	-5.726431	1.825136	-1.181749
39	H	-7.079374	1.223120	-0.185595
40	H	-5.663981	1.976926	0.598354

Transition state: *endo*-16c

sum of electronic and thermal energies	-1088.071609
sum of electronic and thermal enthalpies	-1088.070664
sum of electronic and thermal free energies	-1088.142127
$E_{solvation}$ (Et_2O)	-0.009946

1	C	0.874648	-0.179619	1.383011
2	C	-0.020658	-0.873011	0.509169
3	C	1.771707	-2.071505	0.666816
4	C	2.005295	-0.957917	1.510077
5	H	2.945190	-0.715005	1.984465
6	C	1.066107	-0.363184	-1.326819
7	H	0.157211	-0.475814	-1.902467
8	C	2.119272	-1.282181	-1.242623
9	H	2.161305	-2.231551	-1.761172
10	H	2.299338	-3.015723	0.667946
11	O	0.431417	-2.150466	0.404107
12	C	1.626791	0.997170	-1.086910
13	C	3.378040	-0.513523	-1.029181
14	N	2.996660	0.819283	-0.866185
15	C	3.936411	1.887172	-0.619315
16	H	4.654108	1.960833	-1.440622
17	H	3.365793	2.814395	-0.541935
18	H	4.489689	1.702982	0.306310
19	O	1.061203	2.068866	-1.037049
20	O	4.520736	-0.905354	-0.955969
21	C	-1.444744	-0.621505	0.272205
22	C	-1.905681	0.683987	0.094301
23	C	-2.351089	-1.687031	0.190982
24	C	-3.255163	0.931286	-0.140460
25	H	-1.199114	1.508456	0.112090
26	C	-3.693085	-1.446861	-0.042340
27	H	-1.994408	-2.703938	0.319563
28	C	-4.154719	-0.135326	-0.204344
29	H	-3.588026	1.952726	-0.280333
30	H	-4.412055	-2.256976	-0.102371
31	O	0.586084	1.046281	1.836411
32	C	1.680171	1.753441	2.393440
33	H	2.488683	1.840668	1.659476
34	H	1.306229	2.742482	2.653979
35	H	2.049795	1.246918	3.292745
36	O	-5.487295	-0.003309	-0.423311
37	C	-5.994379	1.302547	-0.592056
38	H	-5.551534	1.791851	-1.467977
39	H	-7.068223	1.192540	-0.742432
40	H	-5.811305	1.918366	0.296815

(3a*R*,4*S*,7*R*,7a*S*)-5-Methoxy-4-(4-methoxyphenyl)-2-methyl-3a,4,7,7a-tetrahydro-1*H*-4,7-epoxyisoindole-1,3(2*H*)-dione *exo*-16c

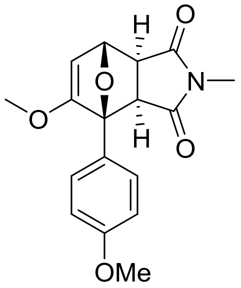

sum of electronic and thermal energies	-1088.113862
sum of electronic and thermal enthalpies	-1088.112918
sum of electronic and thermal free energies	-1088.182858
$E_{solvation}$ (Et_2O)	-0.011207

1	C	-0.779014	2.194730	-0.121776
2	C	-0.472943	0.694383	-0.157184
3	C	-2.350786	0.971834	-1.158663
4	C	-1.939816	2.377730	-0.761718
5	H	-2.535227	3.271900	-0.881162
6	C	-1.483042	0.072744	0.890868
7	H	-1.416106	0.560210	1.865021
8	C	-2.815955	0.227682	0.131958
9	H	-3.614948	0.747731	0.662361
10	H	-3.009437	0.846847	-2.017264
11	O	-1.097201	0.318869	-1.379882
12	C	-1.288688	-1.427848	1.041425
13	O	-0.411700	-1.995963	1.644145
14	C	-3.235932	-1.193769	-0.203867
15	O	-4.192584	-1.534401	-0.859016
16	N	-2.315757	-2.064962	0.352383
17	C	-2.409576	-3.502096	0.203385
18	H	-1.684406	-3.950337	0.882806
19	H	-3.422023	-3.827011	0.450326
20	H	-2.185437	-3.790761	-0.826467
21	C	0.964220	0.284839	-0.078570
22	C	1.665255	0.398142	1.127080
23	C	1.625962	-0.187925	-1.203967
24	C	3.000765	0.050030	1.198349
25	H	1.157156	0.756060	2.017959
26	C	2.972557	-0.549265	-1.143861
27	H	1.083898	-0.276656	-2.139501
28	C	3.662939	-0.427160	0.060713
29	H	3.558590	0.126162	2.125476
30	H	3.462375	-0.919568	-2.036641
31	O	0.029604	3.005664	0.566490
32	C	-0.403973	4.353793	0.621148
33	H	-1.379411	4.421775	1.117248
34	H	0.346536	4.900752	1.190207
35	H	-0.483733	4.767687	-0.390476
36	C	5.672693	-1.245299	-0.883741
37	H	5.695844	-0.514118	-1.701352
38	H	6.689809	-1.438707	-0.542643
39	H	5.227206	-2.178910	-1.248627
40	O	4.973333	-0.744619	0.232833

Transition state: *exo*-16c

sum of electronic and thermal energies	-1088.067650
sum of electronic and thermal enthalpies	-1088.066706
sum of electronic and thermal free energies	-1088.138897
$E_{solvation}$ (Et_2O)	-0.011668

1	C	1.201032	2.114554	0.296190
2	C	0.548812	0.863865	0.529759
3	C	2.617011	0.641834	1.153151
4	C	2.497958	1.994470	0.743250
5	H	3.313918	2.693803	0.636985
6	C	1.627610	-0.074401	-1.286783
7	H	1.402231	0.674797	-2.034081
8	C	2.853818	-0.299172	-0.651470
9	H	3.804154	0.133478	-0.937413
10	H	3.378448	0.196719	1.781290
11	O	1.360083	0.139874	1.337955
12	C	0.839230	-1.327188	-1.238718
13	O	-0.242407	-1.574621	-1.720823
14	C	2.834261	-1.716858	-0.148141
15	O	3.693826	-2.307099	0.468907
16	N	1.590295	-2.232776	-0.471838
17	C	1.126046	-3.540593	-0.074840
18	H	0.071594	-3.605901	-0.348496
19	H	1.688854	-4.326390	-0.585933
20	H	1.247057	-3.666778	1.004038
21	C	-0.861598	0.493320	0.486278
22	C	-1.783841	1.224266	-0.263162
23	C	-1.296160	-0.659482	1.159944
24	C	-3.117680	0.833858	-0.329407
25	H	-1.460835	2.105669	-0.805273
26	C	-2.619172	-1.050072	1.102449
27	H	-0.582578	-1.244716	1.731917
28	C	-3.540745	-0.305716	0.356613
29	H	-3.810263	1.420362	-0.920943
30	H	-2.971371	-1.935181	1.621338
31	O	0.587775	3.127658	-0.341680
32	C	1.409711	4.252360	-0.596905
33	H	2.253456	3.977212	-1.240578
34	H	0.781835	4.985241	-1.101981
35	H	1.789253	4.672641	0.341485
36	C	-5.771230	-0.063221	-0.395947
37	H	-5.505256	-0.053540	-1.459664
38	H	-6.714644	-0.592378	-0.262348
39	H	-5.879998	0.968668	-0.040182
40	O	-4.815314	-0.768965	0.364283

3-Methoxy-2-phenylfuran16d

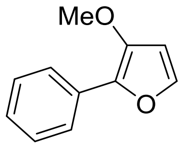

sum of electronic and thermal energies	-575.163174
sum of electronic and thermal enthalpies	-575.162230
sum of electronic and thermal free energies	-575.211203
$E_{\mathrm{solvation}}$ (Et_2O)	-0.004497

1	C	2.103283	2.036461	-0.000092
2	C	2.715953	0.823676	0.000089
3	C	1.651387	-0.133745	-0.000022
4	C	0.470036	0.565588	-0.000004
5	O	0.764819	1.899655	-0.000015
6	H	2.476354	3.048720	-0.000150
7	H	3.779493	0.639356	0.000177
8	C	-0.933686	0.179995	0.000000
9	C	-1.934410	1.161381	0.000041
10	C	-1.306181	-1.172174	-0.000039
11	C	-3.275207	0.797746	0.000043
12	H	-1.652096	2.208868	0.000070
13	C	-2.650256	-1.525210	-0.000033
14	H	-0.538773	-1.937938	-0.000072
15	C	-3.641052	-0.546260	0.000008
16	H	-4.038982	1.569930	0.000076
17	H	-2.925257	-2.575871	-0.000062
18	H	-4.689386	-0.828272	0.000012
19	O	1.733589	-1.486321	-0.000026
20	C	3.044967	-2.011212	0.000032
21	H	3.595665	-1.696482	0.894719
22	H	2.940968	-3.095946	0.000037
23	H	3.595740	-1.696498	-0.894614

(3a*S*,4*S*,7*R*,7a*R*)-5-Methoxy-2-methyl-4-phenyl-3a,4,7,7a-tetrahydro-1*H*-4,7-epoxyisoindole-1,3(2*H*)-dione *endo*-16d

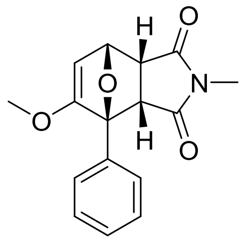

sum of electronic and thermal energies	-973.671932
sum of electronic and thermal enthalpies	-973.670988
sum of electronic and thermal free energies	-973.737160
$E_{solvation}$ (Et_2O)	-0.009146

1	C	0.115238	-0.440784	1.235552
2	C	-0.575703	-0.651117	-0.112443
3	C	0.968597	-2.135272	0.027801
4	C	1.074620	-1.370646	1.335001
5	H	1.848032	-1.494579	2.080080
6	C	0.458736	-0.134798	-1.194315
7	H	-0.040689	-0.049029	-2.161689
8	C	1.536181	-1.231644	-1.124730
9	H	1.652752	-1.803899	-2.046830
10	H	1.315011	-3.166696	0.015622
11	O	-0.423246	-2.057725	-0.277913
12	C	1.192943	1.156472	-0.861621
13	C	2.818940	-0.513026	-0.767663
14	N	2.527347	0.840156	-0.635968
15	C	3.527165	1.812320	-0.246448
16	H	3.866313	1.616251	0.774246
17	H	4.384542	1.746223	-0.919085
18	H	3.067562	2.798882	-0.307361
19	O	0.742360	2.276286	-0.790366
20	O	3.915180	-0.986794	-0.588413
21	C	-2.001581	-0.206359	-0.241451
22	C	-3.028268	-1.130109	-0.043771
23	C	-2.305646	1.125429	-0.526619
24	C	-4.355596	-0.724661	-0.142518
25	H	-2.777655	-2.162142	0.177289
26	C	-3.635902	1.525336	-0.618291
27	H	-1.503212	1.843470	-0.675449
28	C	-4.662131	0.603220	-0.429031
29	H	-5.151223	-1.448747	0.004347
30	H	-3.869534	2.561847	-0.841840
31	H	-5.698436	0.918450	-0.505655
32	O	-0.217536	0.619994	1.972384
33	C	0.631775	0.849053	3.083473
34	H	1.663582	1.002797	2.743998
35	H	0.261661	1.745028	3.579674
36	H	0.599582	-0.003540	3.771345

Transition state: *endo*-16d

sum of electronic and thermal energies	-973.628642
sum of electronic and thermal enthalpies	-973.627697
sum of electronic and thermal free energies	-973.692378
$E_{solvation}$ (Et_2O)	-0.0089313

1	C	0.151616	0.055252	1.349238
2	C	-0.758950	-0.642614	0.494796
3	C	0.841782	-2.012867	0.971928
4	C	1.159434	-0.827228	1.676564
5	H	2.087542	-0.633935	2.194767
6	C	0.493031	-0.533242	-1.287606
7	H	-0.377609	-0.629123	-1.922926
8	C	1.424127	-1.536750	-0.995095
9	H	1.391029	-2.549256	-1.376286
10	H	1.244892	-3.002908	1.136167
11	O	-0.472799	-1.968276	0.602791
12	C	1.192233	0.781771	-1.190216
13	C	2.745095	-0.881614	-0.776438
14	N	2.510226	0.494083	-0.824361
15	C	3.550718	1.480752	-0.653876
16	H	3.087081	2.464444	-0.745639
17	H	4.022384	1.371944	0.326952
18	H	4.319725	1.359920	-1.421308
19	O	0.754325	1.901732	-1.343578
20	O	3.827231	-1.376540	-0.556392
21	C	-2.120714	-0.258036	0.103360
22	C	-2.385046	1.060951	-0.283972
23	C	-3.143145	-1.210679	0.089587
24	C	-3.673869	1.418900	-0.662011
25	H	-1.575035	1.783535	-0.313260
26	C	-4.428433	-0.841438	-0.292131
27	H	-2.925942	-2.232585	0.383417
28	C	-4.697601	0.473332	-0.663795
29	H	-3.877535	2.441380	-0.964538
30	H	-5.221708	-1.582739	-0.297362
31	H	-5.702080	0.759615	-0.959929
32	O	-0.002482	1.358569	1.607975
33	C	1.129642	1.998648	2.171100
34	H	2.000179	1.872934	1.517864
35	H	0.875870	3.054315	2.253802
36	H	1.349908	1.589160	3.163835

(3a*R*,4*S*,7*R*,7a*S*)-5-Methoxy-2-methyl-4-phenyl-3a,4,7,7a-tetrahydro-1*H*-4,7-epoxyisoindole-1,3(2*H*)-dione *exo*-16d

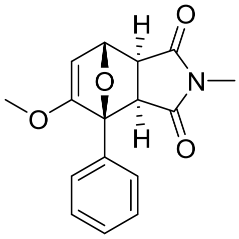

sum of electronic and thermal energies	-973.671516
sum of electronic and thermal enthalpies	-973.670572
sum of electronic and thermal free energies	-973.734983
$E_{\text{solvation}}$ (Et_2O)	-0.010037

1	C	-0.794971	1.945536	0.054071
2	C	-0.355229	0.484300	0.184671
3	C	1.224392	1.684325	1.002945
4	C	0.178306	2.698862	0.579330
5	H	0.289060	3.773702	0.604893
6	C	0.766149	0.321785	-0.919692
7	H	0.423229	0.646082	-1.903616
8	C	1.911684	1.136059	-0.285767
9	H	2.345863	1.920222	-0.907815
10	H	1.910539	1.951242	1.805748
11	O	0.439087	0.544256	1.363608
12	C	1.289047	-1.104167	-0.991479
13	O	0.748889	-2.056560	-1.497467
14	C	2.962480	0.100056	0.079572
15	O	3.998952	0.290915	0.670271
16	N	2.528568	-1.138324	-0.360665
17	C	3.291429	-2.351072	-0.151005
18	H	2.762409	-3.161269	-0.652859
19	H	4.293795	-2.233038	-0.567797
20	H	3.375084	-2.558992	0.918253
21	C	-1.432610	-0.554941	0.253667
22	C	-2.159672	-0.885911	-0.890891
23	C	-1.724326	-1.177670	1.465339
24	C	-3.173066	-1.833748	-0.821592
25	H	-1.926172	-0.407176	-1.837624
26	C	-2.737634	-2.130719	1.530102
27	H	-1.152265	-0.912545	2.347786
28	C	-3.463588	-2.458998	0.389445
29	H	-3.731928	-2.091430	-1.715755
30	H	-2.958222	-2.617408	2.475268
31	H	-4.252276	-3.203506	0.441682
32	O	-1.926750	2.227867	-0.597021
33	C	-2.181846	3.613891	-0.749446
34	H	-1.376236	4.089773	-1.320891
35	H	-3.125876	3.700881	-1.285626
36	H	-2.261684	4.094375	0.232261

Transition state: *exo*-16d

sum of electronic and thermal energies	-973.624795
sum of electronic and thermal enthalpies	-973.623851
sum of electronic and thermal free energies	-973.689441
$E_{solvation}$ (Et_2O)	-0.010548

1	C	-0.619008	2.079079	0.298761
2	C	-0.486430	0.670900	0.512093
3	C	1.351375	1.602725	1.191212
4	C	0.524754	2.678148	0.777572
5	H	0.832067	3.709909	0.692879
6	C	0.951036	0.476173	-1.262721
7	H	0.364253	0.976928	-2.021563
8	C	2.092446	0.961382	-0.615010
9	H	2.647654	1.849365	-0.888353
10	H	2.223901	1.634362	1.831386
11	O	0.569088	0.495409	1.345331
12	C	0.988528	-1.006672	-1.224756
13	O	0.229719	-1.806433	-1.721860
14	C	2.852616	-0.233351	-0.108291
15	O	3.886221	-0.254749	0.523187
16	N	2.106904	-1.349567	-0.449698
17	C	2.444954	-2.696959	-0.057448
18	H	1.678734	-3.354930	-0.470451
19	H	3.426606	-2.974412	-0.449674
20	H	2.465967	-2.781668	1.032550
21	C	-1.470706	-0.408321	0.434685
22	C	-2.636956	-0.269992	-0.325864
23	C	-1.211309	-1.618043	1.090292
24	C	-3.535056	-1.326908	-0.412079
25	H	-2.833716	0.659689	-0.847003
26	C	-2.115941	-2.667443	0.999763
27	H	-0.297615	-1.726373	1.666865
28	C	-3.280711	-2.525283	0.249940
29	H	-4.438194	-1.214641	-1.004018
30	H	-1.908294	-3.601428	1.512523
31	H	-3.985277	-3.347848	0.176107
32	O	-1.667760	2.604001	-0.358007
33	C	-1.576947	3.995527	-0.608251
34	H	-0.699406	4.221119	-1.225340
35	H	-2.485698	4.273186	-1.140398
36	H	-1.513317	4.552122	0.333770

3-Methoxy-2-(4-(trifluoromethyl)phenyl)furan 14e

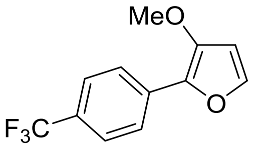

sum of electronic and thermal energies	-912.094821
sum of electronic and thermal enthalpies	-912.093877
sum of electronic and thermal free energies	-912.152171
$E_{solvation}$ (Et_2O)	-0.004644

1	C	-3.785813	-1.831164	0.013684
2	C	-4.237666	-0.549474	0.016197
3	C	-3.058871	0.262801	0.003493
4	C	-1.978295	-0.585023	-0.005800
5	O	-2.442501	-1.870373	0.000501
6	H	-4.288366	-2.785920	0.020017
7	H	-5.268276	-0.228974	0.025773
8	C	-0.539442	-0.384273	-0.017656
9	C	0.324453	-1.489186	-0.015418
10	C	0.005867	0.908988	-0.034862
11	C	1.699494	-1.306883	-0.026252
12	H	-0.091327	-2.490767	-0.007475
13	C	1.381571	1.085486	-0.046316
14	H	-0.653987	1.768571	-0.042346
15	C	2.229834	-0.019569	-0.041375
16	H	2.364223	-2.164462	-0.029656
17	H	1.801927	2.085916	-0.066849
18	O	-2.965510	1.612058	0.001433
19	C	-4.200112	2.301734	0.014961
20	H	-4.775215	2.058109	0.915994
21	H	-3.958109	3.364043	0.012880
22	H	-4.794269	2.058856	-0.873799
23	C	3.713689	0.185817	0.008227
24	F	4.381000	-0.849923	-0.522987
25	F	4.156056	0.327480	1.269462
26	F	4.085082	1.291959	-0.655731

(3a*S*,4*S*,7*R*,7a*R*)-5-Methoxy-2-methyl-4-(4-(trifluoromethyl)phenyl)-3a,4,7,7a-tetrahydro-1*H*-4,7-epoxyisoindole-1,3(2*H*)-dione *endo*-16e

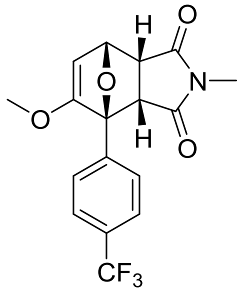

sum of electronic and thermal energies	-1310.603913
sum of electronic and thermal enthalpies	-1310.602968
sum of electronic and thermal free energies	-1310.674839
$E_{solvation}$ (Et_2O)	-0.009117

1	C	1.393451	-0.544282	1.248677
2	C	0.657468	-0.799651	-0.067693
3	C	2.371746	-2.087928	-0.063375
4	C	2.461412	-1.352504	1.261459
5	H	3.287945	-1.400593	1.956546
6	C	1.559878	-0.130120	-1.184384
7	H	1.001110	-0.073155	-2.120926
8	C	2.761009	-1.092159	-1.213278
9	H	2.890151	-1.619254	-2.160074
10	H	2.836063	-3.069654	-0.129914
11	O	0.964132	-2.171114	-0.289876
12	C	2.153647	1.228349	-0.837451
13	C	3.968777	-0.234110	-0.901164
14	N	3.526447	1.069769	-0.702799
15	C	4.426030	2.142268	-0.330503
16	H	4.894891	1.920827	0.631703
17	H	5.208134	2.247444	-1.085054
18	H	3.836989	3.056995	-0.263277
19	O	1.574145	2.279335	-0.688099
20	O	5.121294	-0.577322	-0.798578
21	C	-0.814508	-0.519841	-0.101409
22	C	-1.273599	0.793547	-0.224270
23	C	-1.721015	-1.571404	0.025691
24	C	-2.638800	1.048704	-0.223333
25	H	-0.560680	1.608043	-0.319989
26	C	-3.088281	-1.314804	0.019668
27	H	-1.347998	-2.584668	0.125330
28	C	-3.541312	-0.005786	-0.103892
29	H	-3.007362	2.065920	-0.310417
30	H	-3.801036	-2.126626	0.117837
31	O	0.978348	0.446671	2.038395
32	C	1.866002	0.761262	3.098847
33	H	2.843261	1.053794	2.695788
34	H	1.418384	1.590927	3.643649
35	H	1.989508	-0.102402	3.762028
36	C	-5.012567	0.286264	-0.164689
37	F	-5.745581	-0.700209	0.370861
38	F	-5.432003	0.445216	-1.430561
39	F	-5.317290	1.419062	0.487280

Transition state: *endo*-16e

sum of electronic and thermal energies	-1310.559194
sum of electronic and thermal enthalpies	-1310.558250
sum of electronic and thermal free energies	-1310.632469
$E_{solvation}$ (Et_2O)	-0.009083

1	C	1.447357	-0.163879	1.395506
2	C	0.532203	-0.854121	0.540063
3	C	2.297139	-2.085265	0.700911
4	C	2.565841	-0.960985	1.517753
5	H	3.516906	-0.729572	1.975279
6	C	1.571132	-0.399917	-1.316681
7	H	0.657204	-0.517138	-1.884289
8	C	2.623162	-1.319552	-1.240138
9	H	2.652341	-2.278833	-1.740741
10	H	2.812288	-3.036260	0.700168
11	O	0.954851	-2.144538	0.456877
12	C	2.142854	0.965936	-1.113073
13	C	3.886957	-0.551823	-1.057261
14	N	3.512616	0.786436	-0.910527
15	C	4.461879	1.855358	-0.702727
16	H	5.047029	1.672644	0.203057
17	H	5.149527	1.924281	-1.549648
18	H	3.896037	2.783916	-0.609242
19	O	1.578292	2.037914	-1.075773
20	O	5.028751	-0.945921	-0.995359
21	C	-0.894647	-0.580654	0.328958
22	C	-1.323396	0.737813	0.123363
23	C	-1.815270	-1.630354	0.304856
24	C	-2.670269	0.994122	-0.083484
25	H	-0.594170	1.541688	0.096934
26	C	-3.164823	-1.366839	0.097425
27	H	-1.471868	-2.648418	0.454919
28	C	-3.587947	-0.056253	-0.090876
29	H	-3.013831	2.010135	-0.252469
30	H	-3.885924	-2.176602	0.081256
31	O	1.188253	1.073993	1.828741
32	C	2.298752	1.766479	2.374663
33	H	3.113003	1.812170	1.643106
34	H	1.949573	2.771193	2.607141
35	H	2.649159	1.272646	3.288276
36	C	-5.041969	0.264083	-0.284142
37	F	-5.781618	-0.836184	-0.478584
38	F	-5.229175	1.070655	-1.339774
39	F	-5.549261	0.902931	0.782065

(3a*R*,4*S*,7*R*,7a*S*)-5-Methoxy-2-methyl-4-(4-(trifluoromethyl)phenyl)-3a,4,7,7a-tetrahydro-1*H*-4,7-epoxyisoindole-1,3(2*H*)-dione *exo*-16e

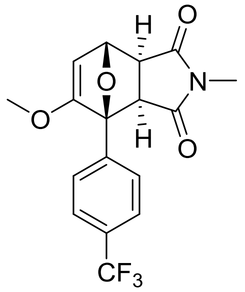

sum of electronic and thermal energies	-1310.603408
sum of electronic and thermal enthalpies	-1310.602464
sum of electronic and thermal free energies	-1310.674245
$E_{solvation}$ (Et_2O)	-0.010152

1	C	1.429552	2.196447	0.113869
2	C	1.023181	0.721392	0.175702
3	C	2.959086	0.866255	1.084829
4	C	2.630664	2.295479	0.694836
5	H	3.294003	3.144705	0.777781
6	C	1.932064	0.015696	-0.909199
7	H	1.849564	0.490881	-1.888173
8	C	3.307693	0.082348	-0.219590
9	H	4.113137	0.543334	-0.792854
10	H	3.646230	0.699802	1.913270
11	O	1.672064	0.308403	1.371348
12	C	1.614931	-1.468652	-1.015024
13	O	0.652230	-1.972090	-1.539127
14	C	3.644336	-1.363207	0.110163
15	O	4.607922	-1.764615	0.717556
16	N	2.634474	-2.171671	-0.384237
17	C	2.628046	-3.609781	-0.211586
18	H	1.854973	-4.016666	-0.863677
19	H	3.608277	-4.008792	-0.478174
20	H	2.410743	-3.865541	0.828407
21	C	-0.440471	0.402256	0.157440
22	C	-1.196210	0.676361	-0.982741
23	C	-1.045757	-0.172708	1.273695
24	C	-2.553884	0.386762	-1.006131
25	H	-0.721908	1.116428	-1.854477
26	C	-2.403469	-0.469385	1.249638
27	H	-0.447460	-0.387226	2.152082
28	C	-3.152950	-0.186375	0.111779
29	H	-3.145334	0.594351	-1.890990
30	H	-2.881226	-0.926176	2.110761
31	O	0.649119	3.062894	-0.538252
32	C	1.177182	4.376843	-0.621572
33	H	2.129482	4.370359	-1.164539
34	H	0.441984	4.974939	-1.158036
35	H	1.334593	4.785744	0.382627
36	C	-4.627261	-0.467973	0.125405
37	F	-4.898265	-1.649827	0.699968
38	F	-5.301398	0.460969	0.823889
39	F	-5.149166	-0.486889	-1.108928

Transition state: *exo*-16e

sum of electronic and thermal energies	-1310.555077
sum of electronic and thermal enthalpies	-1310.554133
sum of electronic and thermal free energies	-1310.628341
$E_{solvation}$ (Et_2O)	-0.010678

1	C	1.861941	2.059837	0.309261
2	C	1.088417	0.871911	0.499340
3	C	3.088168	0.457142	1.222570
4	C	3.120194	1.814182	0.811101
5	H	4.004332	2.431113	0.749125
6	C	2.108543	-0.177165	-1.246967
7	H	1.963845	0.576690	-2.009734
8	C	3.300509	-0.500244	-0.589197
9	H	4.286836	-0.139351	-0.850468
10	H	3.779968	-0.063754	1.872684
11	O	1.784408	0.077007	1.350271
12	C	1.222774	-1.369822	-1.217853
13	O	0.135538	-1.527330	-1.723645
14	C	3.159449	-1.911298	-0.089378
15	O	3.955989	-2.568250	0.542926
16	N	1.885458	-2.329762	-0.440886
17	C	1.324797	-3.606922	-0.066848
18	H	1.955956	-4.420086	-0.433308
19	H	1.246858	-3.686154	1.020970
20	H	0.333785	-3.670130	-0.518885
21	C	-0.352620	0.644000	0.393523
22	C	-1.156978	1.473361	-0.395641
23	C	-0.920340	-0.458912	1.044164
24	C	-2.515100	1.210736	-0.515454
25	H	-0.717382	2.316205	-0.915762
26	C	-2.276646	-0.717067	0.921929
27	H	-0.290210	-1.110780	1.640691
28	C	-3.072525	0.119944	0.143893
29	H	-3.142313	1.846920	-1.130431
30	H	-2.719889	-1.573252	1.419933
31	O	1.377786	3.123936	-0.351792
32	C	2.314183	4.164701	-0.576155
33	H	3.153028	3.804167	-1.182669
34	H	1.780442	4.949653	-1.109633
35	H	2.691600	4.553425	0.376201
36	C	-4.549264	-0.137153	0.066586
37	F	-4.822477	-1.449891	0.055884
38	F	-5.195366	0.383622	1.123377
39	F	-5.096362	0.402644	-1.031910

2-Methylfuran 14f

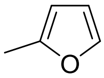

sum of electronic and thermal energies	-269.119085
sum of electronic and thermal enthalpies	-269.118141
sum of electronic and thermal free energies	-269.153260
$E_{solvation}$ (Et_2O)	-0.002199

1	C	-1.388068	-0.734199	0.000272
2	C	-1.547143	0.611461	-0.000251
3	C	-0.222333	1.162126	0.000190
4	C	0.633799	0.106605	-0.000022
5	O	-0.069956	-1.056272	-0.000156
6	H	-2.074281	-1.566540	0.000399
7	H	-2.483425	1.149510	-0.000485
8	H	0.056041	2.206069	0.000307
9	C	2.116585	-0.014206	-0.000010
10	H	2.468482	-0.554286	0.884683
11	H	2.468349	-0.555376	-0.884086
12	H	2.567442	0.980078	-0.000649

(3a*S*,4*S*,7*R*,7a*R*)-2,4-Dimethyl-3a,4,7,7a-tetrahydro-1*H*-4,7-epoxyisoindole-1,3(2*H*)-dione *endo*-16f

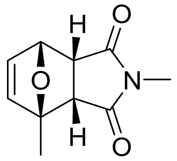

sum of electronic and thermal energies	-667.622040
sum of electronic and thermal enthalpies	-667.621096
sum of electronic and thermal free energies	-667.670769
$E_{solvation}$ (Et_2O)	-0.007375

1	C	-1.162089	-0.000264	1.431173
2	C	-1.712778	0.295347	0.038921
3	C	-0.972653	-1.715454	-0.038615
4	C	-0.710750	-1.252797	1.386460
5	H	-0.167402	-1.810061	2.139293
6	C	-0.464776	0.453823	-0.911729
7	H	-0.784021	0.855860	-1.876061
8	C	0.069904	-0.982923	-0.963544
9	H	0.077342	-1.427297	-1.960324
10	H	-1.085909	-2.781915	-0.221811
11	O	-2.155640	-1.000315	-0.376692
12	C	0.659036	1.283612	-0.328493
13	C	1.476909	-0.897788	-0.401799
14	N	1.734785	0.433726	-0.093198
15	C	2.966222	0.855629	0.541022
16	H	3.051269	0.407831	1.534482
17	H	3.818157	0.537477	-0.062795
18	H	2.938164	1.942505	0.621129
19	O	0.641770	2.464010	-0.068465
20	O	2.258288	-1.798384	-0.209516
21	H	-1.082101	0.726601	2.230910
22	C	-2.775476	1.351132	-0.102460
23	H	-3.153511	1.367967	-1.128319
24	H	-3.606475	1.150400	0.578181
25	H	-2.345662	2.330155	0.129474

Transition state: *endo*-16f

sum of electronic and thermal energies	-667.578751
sum of electronic and thermal enthalpies	-667.577807
sum of electronic and thermal free energies	-667.629001
$E_{solvation}$ (Et_2O)	-0.007649

1	C	-1.093362	0.037209	1.405348
2	C	-1.879273	0.204788	0.233144
3	C	-1.092903	-1.801356	0.157536
4	C	-0.602439	-1.244122	1.364895
5	H	0.136240	-1.701097	2.009132
6	C	-0.291014	0.435177	-1.175332
7	H	-0.934922	0.877459	-1.924979
8	C	0.220075	-0.867804	-1.205361
9	H	0.055961	-1.587460	-1.997151
10	H	-1.120275	-2.840897	-0.138892
11	O	-2.131897	-1.035327	-0.280551
12	C	0.675043	1.295343	-0.438387
13	C	1.536230	-0.849584	-0.495278
14	N	1.702895	0.449756	-0.016914
15	C	2.798422	0.855009	0.831847
16	H	2.689753	0.432058	1.835435
17	H	3.741693	0.508169	0.405225
18	H	2.784699	1.944223	0.888540
19	O	0.605441	2.479131	-0.189522
20	O	2.320169	-1.751746	-0.307355
21	H	-0.837207	0.832550	2.092746
22	C	-2.854443	1.291827	-0.073957
23	H	-3.231783	1.185316	-1.093330
24	H	-3.703032	1.268660	0.616756
25	H	-2.349110	2.257346	0.017606

(3a*S*,4*S*,7*R*,7a*R*)-2,4-Dimethyl-3a,4,7,7a-tetrahydro-1*H*-4,7-epoxyisoindole-1,3(2*H*)-dione *endo*-16f

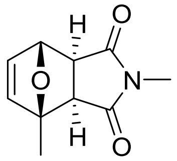

sum of electronic and thermal energies	-667.625517
sum of electronic and thermal enthalpies	-667.624573
sum of electronic and thermal free energies	-667.672667
$E_{\text{solvation}}$ (Et_2O)	-0.007683

1	C	2.707361	-0.046523	-0.398045
2	C	1.477321	0.656911	0.171615
3	C	1.117581	-1.445671	0.405347
4	C	2.490614	-1.350266	-0.241643
5	H	3.087496	-2.188129	-0.579378
6	C	0.341343	0.411622	-0.897523
7	H	0.635240	0.733389	-1.897862
8	C	0.078272	-1.094911	-0.709061
9	H	0.169949	-1.718679	-1.599648
10	H	0.872029	-2.325644	0.998107
11	O	1.063985	-0.259011	1.189401
12	C	-0.958656	1.087423	-0.495265
13	O	-1.200477	2.271563	-0.514056
14	C	-1.331357	-1.176545	-0.147115
15	O	-1.921382	-2.170129	0.203550
16	N	-1.846390	0.107895	-0.071640
17	C	-3.161487	0.392606	0.463753
18	H	-3.397409	1.429902	0.225668
19	H	-3.888876	-0.283171	0.010785
20	H	-3.169137	0.247790	1.547014
21	H	3.528687	0.455851	-0.895266
22	C	1.649850	2.055225	0.706338
23	H	2.463964	2.067840	1.435385
24	H	1.888192	2.743531	-0.109259
25	H	0.732533	2.403451	1.184368

Transition state: *exo*-16f

sum of electronic and thermal energies	-667.578452
sum of electronic and thermal enthalpies	-667.577508
sum of electronic and thermal free energies	-667.629626
$E_{solvation}$ (Et_2O)	-0.007589

1	C	2.685431	-0.062826	-0.310955
2	C	1.651638	0.655662	0.344570
3	C	1.191198	-1.442415	0.585843
4	C	2.414714	-1.395821	-0.130247
5	H	2.914350	-2.246734	-0.571674
6	C	0.146397	0.265974	-1.267315
7	H	0.676882	0.670552	-2.117704
8	C	-0.087039	-1.088983	-0.994600
9	H	0.168094	-1.918060	-1.641447
10	H	0.740734	-2.273532	1.113301
11	O	0.977188	-0.214803	1.141919
12	C	-0.968226	1.046896	-0.668636
13	O	-1.155600	2.245433	-0.683234
14	C	-1.365710	-1.174274	-0.209642
15	O	-1.918725	-2.159605	0.224200
16	N	-1.795211	0.130924	-0.013621
17	C	-2.932367	0.492906	0.801075
18	H	-2.856203	1.555223	1.037061
19	H	-3.867284	0.307191	0.265338
20	H	-2.922981	-0.103113	1.715536
21	H	3.461012	0.388544	-0.914692
22	C	1.623322	2.094783	0.729766
23	H	2.184464	2.244983	1.657948
24	H	2.087819	2.690213	-0.059261
25	H	0.600544	2.448656	0.868701

2-Cyclopropylfuran 14g

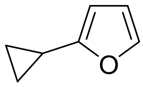

sum of electronic and thermal energies	-346.438231
sum of electronic and thermal enthalpies	-346.437287
sum of electronic and thermal free energies	-346.476065
$E_{solvation}$ (Et_2O)	-0.002405

1	C	-0.161426	0.389484	0.000305
2	C	-1.270497	1.176259	0.000011
3	C	-2.396782	0.286551	-0.000186
4	C	-1.883216	-0.966653	0.000065
5	O	-0.524002	-0.920201	-0.000247
6	H	-1.284603	2.256202	0.000124
7	H	-3.443673	0.552117	-0.000062
8	H	-2.318370	-1.953554	0.000231
9	C	1.280693	0.674916	0.000401
10	C	2.221012	-0.238080	0.748017
11	C	2.221106	-0.237171	-0.748349
12	H	1.513353	1.734581	0.001189
13	H	1.772974	-1.089161	1.249396
14	H	3.056902	0.218161	1.266631
15	H	3.057241	0.219076	-1.266582
16	H	1.772845	-1.087651	-1.250544

(3a*S*,4*S*,7*R*,7a*R*)-4-Cyclopropyl-2-methyl-3a,4,7,7a-tetrahydro-1*H*-4,7-epoxyisoindole-1,3(2*H*)-dione *endo*-16g

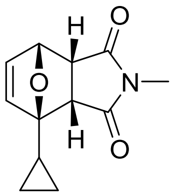

sum of electronic and thermal energies	-744.940890
sum of electronic and thermal enthalpies	-744.939946
sum of electronic and thermal free energies	-744.993859
$E_{solvation}$ (Et_2O)	-0.007067

1	C	-0.561339	-0.298159	1.513260
2	C	-1.215152	-0.034787	0.159365
3	C	-0.169343	-1.896144	-0.045354
4	C	0.081288	-1.458408	1.388892
5	H	0.738424	-1.955507	2.091566
6	C	-0.057708	0.360304	-0.835250
7	H	-0.492602	0.742545	-1.761317
8	C	0.695578	-0.969071	-0.976847
9	H	0.725004	-1.365683	-1.993176
10	H	-0.123568	-2.958382	-0.275918
11	O	-1.467140	-1.366366	-0.301312
12	C	0.948190	1.336432	-0.263860
13	C	2.097170	-0.681210	-0.472333
14	N	2.156609	0.662211	-0.117604
15	C	3.335433	1.252016	0.481727
16	H	3.490486	0.851766	1.487248
17	H	4.208383	1.017259	-0.129618
18	H	3.177114	2.329453	0.530976
19	O	0.758587	2.487535	0.052047
20	O	3.018868	-1.452994	-0.355965
21	H	-0.569917	0.394072	2.346621
22	C	-2.427989	0.840241	0.119454
23	C	-3.479968	0.583231	-0.919070
24	C	-3.750547	0.264199	0.526077
25	H	-2.213200	1.874341	0.377827
26	H	-3.318868	-0.255891	-1.587478
27	H	-3.978008	1.439722	-1.359445
28	H	-4.437599	0.897698	1.075866
29	H	-3.758095	-0.784130	0.805545

Transition state: *endo*-16g

sum of electronic and thermal energies	-744.898043
sum of electronic and thermal enthalpies	-744.897099
sum of electronic and thermal free energies	-744.953953
$E_{solvation}$ (Et_2O)	-0.007797

1	C	-0.479972	-0.239229	1.517916
2	C	-1.363945	-0.150149	0.408414
3	C	-0.254847	-1.984081	0.157927
4	C	0.210596	-1.416220	1.371229
5	H	1.057033	-1.772507	1.942354
6	C	0.094122	0.408845	-1.079519
7	H	-0.669559	0.786652	-1.746825
8	C	0.778770	-0.806030	-1.215012
9	H	0.665702	-1.496108	-2.041741
10	H	-0.140076	-3.001879	-0.189133
11	O	-1.441326	-1.384422	-0.167515
12	C	0.977470	1.358204	-0.352139
13	C	2.129802	-0.632717	-0.593568
14	N	2.142345	0.648616	-0.046250
15	C	3.227777	1.173931	0.747755
16	H	3.227874	2.260425	0.649442
17	H	3.109394	0.908242	1.803579
18	H	4.164675	0.754108	0.378106
19	O	0.769485	2.507514	-0.030323
20	O	3.043104	-1.422914	-0.518066
21	H	-0.316153	0.550606	2.238669
22	C	-2.508664	0.763278	0.224598
23	C	-3.422574	0.580674	-0.952861
24	C	-3.909509	0.239569	0.423683
25	H	-2.292870	1.777982	0.545993
26	H	-3.201697	-0.245195	-1.621448
27	H	-3.808463	1.473986	-1.430699
28	H	-4.629268	0.896985	0.898417
29	H	-4.007261	-0.811468	0.673728

(3a*R*,4*S*,7*R*,7a*S*)-4-Cyclopropyl-2-methyl-3a,4,7,7a-tetrahydro-1*H*-4,7-epoxyisoindole-1,3(2*H*)-dione *exo*-16g

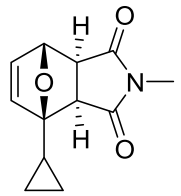

sum of electronic and thermal energies	-744.943553
sum of electronic and thermal enthalpies	-744.942608
sum of electronic and thermal free energies	-744.996511
$E_{solvation}$ (Et_2O)	-0.007664

1	C	2.077979	1.427579	0.747093
2	C	1.289505	0.199630	0.296070
3	C	0.276737	1.893770	-0.546965
4	C	1.452290	2.477912	0.220240
5	H	1.650486	3.532967	0.361319
6	C	-0.035747	0.234464	1.158277
7	H	0.169386	0.312379	2.226778
8	C	-0.771459	1.422871	0.509821
9	H	-1.099723	2.216230	1.183309
10	H	-0.138310	2.452841	-1.384587
11	O	0.785245	0.631617	-0.970202
12	C	-0.915424	-0.972865	0.883777
13	O	-0.753159	-2.101864	1.279266
14	C	-1.962106	0.795881	-0.199212
15	O	-2.769417	1.355764	-0.901688
16	N	-1.966859	-0.562452	0.071039
17	C	-2.958138	-1.463797	-0.477983
18	H	-2.847835	-2.422472	0.029168
19	H	-3.955210	-1.051637	-0.312464
20	H	-2.801157	-1.589163	-1.552421
21	H	2.921495	1.400990	1.426253
22	C	2.004465	-1.113047	0.200909
23	C	1.627423	-2.076597	-0.882904
24	C	2.939033	-1.345837	-0.951053
25	H	2.269482	-1.538055	1.165284
26	H	0.849383	-1.763725	-1.571619
27	H	1.626664	-3.131380	-0.633463
28	H	3.852161	-1.899531	-0.762550
29	H	3.022495	-0.546201	-1.679709

Transition state: *exo*-16g

sum of electronic and thermal energies	-744.896933
sum of electronic and thermal enthalpies	-744.895989
sum of electronic and thermal free energies	-744.951321
$E_{solvation}$ (Et_2O)	-0.007985

1	C	1.775707	1.604341	0.426985
2	C	1.288688	0.445579	-0.236781
3	C	-0.170632	2.004224	-0.576939
4	C	0.870151	2.604923	0.178507
5	H	0.842496	3.599136	0.602069
6	C	-0.279165	0.048272	1.316573
7	H	0.341206	0.015006	2.200973
8	C	-1.170356	1.066899	0.953113
9	H	-1.438111	1.920040	1.562822
10	H	-0.951012	2.478717	-1.158034
11	O	0.307509	0.832537	-1.092571
12	C	-0.768546	-1.221627	0.722391
13	O	-0.308084	-2.339740	0.811349
14	C	-2.243545	0.438770	0.106944
15	O	-3.202638	0.969324	-0.406985
16	N	-1.906143	-0.900228	-0.025906
17	C	-2.614113	-1.834866	-0.868990
18	H	-2.229254	-1.811529	-1.893337
19	H	-2.479881	-2.835363	-0.455439
20	H	-3.670523	-1.562264	-0.878279
21	H	2.636996	1.633545	1.080976
22	C	1.975822	-0.820408	-0.534203
23	C	3.445567	-0.809252	-0.888285
24	C	2.998240	-1.325798	0.442480
25	H	1.348038	-1.564768	-1.011418
26	H	3.935326	0.158598	-0.924682
27	H	3.782891	-1.520586	-1.634037
28	H	3.008996	-2.393888	0.623571
29	H	3.194630	-0.718358	1.321052

2-(4-Methoxyphenyl)furan 14h

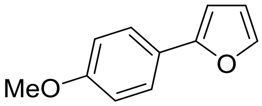

sum of electronic and thermal energies	-575.171025
sum of electronic and thermal enthalpies	-575.170081
sum of electronic and thermal free energies	-575.216791
$E_{solvation}$ (Et_2O)	-0.004475

1	C	0.000005615	0.000013963	-0.000007040
2	C	-0.000004145	-0.000003872	-0.000004677
3	C	-0.000003343	-0.000004960	-0.000002653
4	C	-0.000001773	0.000015699	-0.000011136
5	O	-0.000003037	-0.000019688	-0.000003484
6	H	-0.000000252	0.000002131	-0.000005612
7	H	-0.000002909	-0.000002170	-0.000006905
8	H	-0.000001601	-0.000001542	-0.000003737
9	C	0.000007474	0.000001693	0.000047030
10	C	0.000036099	-0.000001036	-0.000035697
11	C	-0.000034152	0.000000393	-0.000021623
12	C	-0.000055054	-0.000000573	-0.000008892
13	H	-0.000002151	-0.000000108	0.000002500
14	C	0.000012108	-0.000002227	-0.000025549
15	H	0.000004010	-0.000000072	0.000003315
16	C	0.000019656	0.000000776	0.000059064
17	H	0.000009146	0.000000955	0.000003964
18	H	-0.000004818	-0.000000092	0.000008239
19	O	0.000007775	0.000000511	-0.000015534
20	C	0.000004815	0.000003326	0.000010126
21	H	-0.000002997	-0.000001134	0.000003536
22	H	0.000004110	0.000003591	0.000007040
23	H	0.000005425	-0.000005563	0.000007724

(3a*S*,4*S*,7*R*,7a*R*)-4-(4-Methoxyphenyl)-2-methyl-3a,4,7,7a-tetrahydro-1*H*-4,7-epoxyisoindole-1,3(2H)-dione *endo*-16h

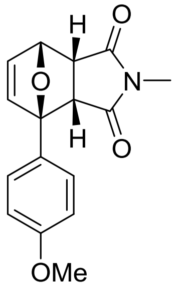

sum of electronic and thermal energies	-973.668910
sum of electronic and thermal enthalpies	-973.667965
sum of electronic and thermal free energies	-973.732126
$E_{solvation}$ (Et_2O)	-0.008788

1	C	0.909823	-0.788228	1.451285
2	C	0.235226	-0.757524	0.078726
3	C	1.961778	-2.017188	-0.137786
4	C	1.977635	-1.572885	1.316294
5	H	2.764347	-1.784568	2.029806
6	C	1.164899	0.132927	-0.843050
7	H	0.639861	0.366658	-1.771812
8	C	2.385787	-0.785230	-1.015514
9	H	2.567798	-1.098959	-2.044801
10	H	2.447033	-2.956549	-0.394843
11	O	0.571453	-2.052114	-0.431618
12	C	1.716330	1.398991	-0.209432
13	C	3.561755	0.002709	-0.477544
14	N	3.087351	1.240774	-0.057931
15	C	3.940313	2.226736	0.573078
16	H	4.161793	1.936710	1.603789
17	H	4.875454	2.295913	0.015388
18	H	3.411492	3.179894	0.564603
19	O	1.106956	2.388715	0.128192
20	O	4.713294	-0.348254	-0.384883
21	H	0.592549	-0.193141	2.298869
22	C	-1.240123	-0.499254	0.021405
23	C	-1.743489	0.793322	-0.083151
24	C	-2.132335	-1.573084	0.117921
25	C	-3.119460	1.025362	-0.096096
26	H	-1.059878	1.636911	-0.147020
27	C	-3.498478	-1.354503	0.097253
28	H	-1.739769	-2.581603	0.197129
29	C	-4.000626	-0.052099	-0.009245
30	H	-3.484403	2.042357	-0.178121
31	H	-4.205856	-2.174573	0.162237
32	O	-5.354712	0.058202	-0.021452
33	C	-5.901335	1.355721	-0.099668
34	H	-6.983912	1.229762	-0.085496
35	H	-5.594468	1.970073	0.755616
36	H	-5.605621	1.858672	-1.028604

Transition state: *endo*-16h

sum of electronic and thermal energies	-973.629380
sum of electronic and thermal enthalpies	-973.628436
sum of electronic and thermal free energies	-973.693490
$E_{solvation}$ (Et_2O)	-0.009004

1	C	0.989617	-0.558632	1.545688
2	C	0.144334	-0.949567	0.467302
3	C	1.965751	-2.086984	0.256197
4	C	2.132331	-1.305797	1.434850
5	H	3.048670	-1.220212	2.002585
6	C	1.243678	0.126044	-1.101067
7	H	0.380831	0.115989	-1.752785
8	C	2.361060	-0.720059	-1.189821
9	H	2.524979	-1.435919	-1.986263
10	H	2.517451	-2.974743	-0.023173
11	O	0.629300	-2.113289	-0.046230
12	C	1.678636	1.389732	-0.454259
13	C	3.540629	0.033026	-0.651147
14	N	3.038091	1.239517	-0.169417
15	C	3.815320	2.222137	0.548364
16	H	3.504785	2.269989	1.596556
17	H	4.861946	1.921418	0.486246
18	H	3.675990	3.206074	0.095909
19	O	1.036554	2.378409	-0.165442
20	O	4.696921	-0.315740	-0.583618
21	H	0.775508	0.246983	2.235315
22	C	-1.278679	-0.653862	0.264401
23	C	-1.757199	0.633216	0.508042
24	C	-2.163433	-1.639211	-0.191973
25	C	-3.102675	0.938210	0.325733
26	H	-1.063973	1.419223	0.797213
27	C	-3.502424	-1.343292	-0.375967
28	H	-1.793613	-2.639786	-0.392094
29	C	-3.981951	-0.054702	-0.113416
30	H	-3.446267	1.948335	0.513392
31	H	-4.205811	-2.093147	-0.722027
32	O	-5.308891	0.132754	-0.321730
33	C	-5.836110	1.415712	-0.061531
34	H	-6.903047	1.352419	-0.274547
35	H	-5.689144	1.700714	0.987225
36	H	-5.379321	2.173148	-0.709868

(3a*R*,4*S*,7*R*,7a*S*)-4-(4-Methoxyphenyl)-2-methyl-3a,4,7,7a-tetrahydro-1*H*-4,7-epoxyisoindole-1,3(2H)-dione *exo*-16h

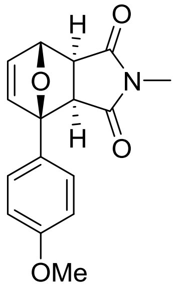

sum of electronic and thermal energies	-973.668491
sum of electronic and thermal enthalpies	-973.667547
sum of electronic and thermal free energies	-973.731193
$E_{solvation}$ (Et_2O)	-0.009709

1	C	-0.625950	2.478588	0.768953
2	C	-0.431325	1.042589	0.272498
3	C	-2.255273	1.742973	-0.619268
4	C	-1.748267	2.915394	0.203869
5	H	-2.266868	3.854385	0.350894
6	C	-1.511581	0.199677	1.067907
7	H	-1.447069	0.353157	2.146014
8	C	-2.808026	0.690731	0.390991
9	H	-3.577899	1.086745	1.055131
10	H	-2.899643	1.936636	-1.475705
11	O	-1.048891	1.097785	-1.015420
12	C	-1.447767	-1.287391	0.751316
13	O	-0.648179	-2.091484	1.164712
14	C	-3.330035	-0.517787	-0.369176
15	O	-4.292262	-0.557905	-1.098462
16	N	-2.497472	-1.591197	-0.108528
17	C	-2.704514	-2.898715	-0.695573
18	H	-1.972144	-3.575366	-0.255185
19	H	-3.719220	-3.241395	-0.482579
20	H	-2.568098	-2.847205	-1.778266
21	H	0.019265	2.960182	1.493447
22	C	0.979626	0.540489	0.204886
23	C	1.600702	-0.040846	1.315524
24	C	1.718829	0.726722	-0.958064
25	C	2.930024	-0.421302	1.259690
26	H	1.034098	-0.225497	2.223392
27	C	3.056357	0.340358	-1.030566
28	H	1.239166	1.175961	-1.821480
29	C	3.666780	-0.232152	0.085474
30	H	3.422567	-0.882994	2.108634
31	H	3.603337	0.492324	-1.953513
32	O	4.962049	-0.639547	0.130113
33	C	5.738220	-0.467406	-1.034055
34	H	6.730552	-0.850657	-0.796171
35	H	5.322765	-1.031709	-1.877975
36	H	5.812956	0.591483	-1.310910

Transition state: *exo*-16h

sum of electronic and thermal energies	-973.627498
sum of electronic and thermal enthalpies	-973.626553
sum of electronic and thermal free energies	-973.692195
$E_{solvation}$ (Et_2O)	-0.010700

1	C	-1.241560	2.641469	0.066547
2	C	-0.600364	1.422360	-0.290258
3	C	-2.650724	1.263007	-0.973730
4	C	-2.522234	2.566989	-0.414129
5	H	-3.332334	3.265324	-0.258140
6	C	-1.749290	0.265281	1.370967
7	H	-1.542170	0.896539	2.223233
8	C	-2.955105	0.174795	0.656191
9	H	-3.905989	0.574237	0.988639
10	H	-3.392011	0.908611	-1.679511
11	O	-1.383078	0.784527	-1.193474
12	C	-0.999178	-0.992807	1.188119
13	O	0.061828	-1.337083	1.658265
14	C	-2.962080	-1.182055	-0.009758
15	O	-3.816815	-1.660172	-0.722292
16	N	-1.753694	-1.776989	0.294946
17	C	-1.315951	-3.040699	-0.247619
18	H	-0.325184	-3.239823	0.164108
19	H	-2.005642	-3.839334	0.036833
20	H	-1.268516	-2.990108	-1.339074
21	H	-0.801988	3.425022	0.667770
22	C	0.804054	1.032228	-0.220369
23	C	1.698488	1.724939	0.607383
24	C	1.266676	-0.074672	-0.931769
25	C	3.017227	1.328489	0.706194
26	H	1.355919	2.577281	1.187353
27	C	2.594150	-0.480030	-0.844558
28	H	0.578011	-0.627600	-1.563765
29	C	3.475032	0.221064	-0.019948
30	H	3.720674	1.851711	1.344745
31	H	2.922420	-1.342985	-1.411148
32	O	4.783508	-0.089342	0.145565
33	C	5.279627	-1.217604	-0.542102
34	H	6.330361	-1.301724	-0.265395
35	H	4.748460	-2.129100	-0.243504
36	H	5.197697	-1.089078	-1.628230

2-Phenylfuran 14i

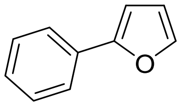

sum of electronic and thermal energies	-460.728067
sum of electronic and thermal enthalpies	-460.727123
sum of electronic and thermal free energies	-460.770349
$E_{solvation}$ (Et_2O)	-0.003318

1	C	0.000059476	-0.000056737	0.000031626
2	C	-0.000039760	0.000069865	0.000021795
3	C	-0.000029963	-0.000066889	0.000034486
4	C	0.000006303	0.000045311	0.000004959
5	O	0.000000553	0.000011982	-0.000073212
6	H	-0.000000225	-0.000005466	0.000018579
7	H	0.000001296	0.000009392	0.000009745
8	H	0.000007300	-0.000008091	0.000003328
9	C	-0.000019282	0.000004259	-0.000013367
10	C	-0.000057981	-0.000001076	-0.000055161
11	C	-0.000028836	-0.000002930	0.000058917
12	C	0.000037571	-0.000000889	-0.000018737
13	H	0.000024661	0.000000494	0.000019927
14	C	0.000039198	0.000001249	0.000003150
15	H	0.000014410	-0.000000436	-0.000028925
16	C	0.000003093	0.000000225	-0.000008829
17	H	-0.000004712	-0.000000008	0.000010994
18	H	0.000000340	-0.000000083	-0.000018354
19	H	-0.000013443	-0.000000171	-0.000000920

(3a*S*,4*S*,7*R*,7a*R*)-2-Methyl-4-phenyl-3a,4,7,7a-tetrahydro-1*H*-4,7-epoxyisoindole-1,3(2*H*)-dione *endo*-16i

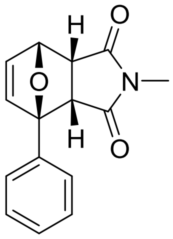

sum of electronic and thermal energies	-859.226615
sum of electronic and thermal enthalpies	-859.225670
sum of electronic and thermal free energies	-859.284001
$E_{solvation}$ (Et_2O)	-0.007665

1	C	0.107892	-0.655818	1.457721
2	C	-0.537872	-0.581682	0.072720
3	C	0.999063	-2.073406	-0.072957
4	C	1.058365	-1.584375	1.365850
5	H	1.798306	-1.879952	2.099348
6	C	0.520866	0.141477	-0.858505
7	H	0.048492	0.423117	-1.802223
8	C	1.600993	-0.945923	-0.985615
9	H	1.744521	-1.318350	-2.001143
10	H	1.351185	-3.079965	-0.289360
11	O	-0.378728	-1.925988	-0.388758
12	C	1.239871	1.333010	-0.246786
13	C	2.871933	-0.316235	-0.455377
14	N	2.574482	0.988158	-0.078383
15	C	3.555039	1.857360	0.539109
16	H	3.807420	1.492697	1.538240
17	H	4.459817	1.871983	-0.070838
18	H	3.118220	2.853716	0.605915
19	O	0.772864	2.404934	0.064332
20	O	3.960862	-0.824040	-0.334214
21	H	-0.136768	0.005262	2.280059
22	C	-1.964719	-0.124526	-0.007937
23	C	-2.275728	1.234020	-0.083877
24	C	-2.989095	-1.070984	0.044017
25	C	-3.608062	1.638886	-0.107589
26	H	-1.476558	1.970744	-0.120533
27	C	-4.317799	-0.660412	0.013014
28	H	-2.734208	-2.123956	0.100456
29	C	-4.630274	0.694985	-0.060751
30	H	-3.845583	2.696755	-0.166070
31	H	-5.111196	-1.400991	0.048118
32	H	-5.667853	1.014310	-0.082383

Transition state: *endo*-16i

sum of electronic and thermal energies	-859.186767
sum of electronic and thermal enthalpies	-859.185823
sum of electronic and thermal free energies	-859.244876
$E_{solvation}$ (Et_2O)	-0.007922

1	C	0.216647	-0.451064	1.532684
2	C	-0.651957	-0.730867	0.438759
3	C	0.971027	-2.144692	0.302164
4	C	1.231568	-1.368986	1.467227
5	H	2.137619	-1.410011	2.056075
6	C	0.620385	0.123432	-1.116732
7	H	-0.214577	0.230951	-1.796003
8	C	1.603017	-0.877937	-1.165635
9	H	1.677223	-1.628283	-1.943434
10	H	1.387100	-3.111102	0.049567
11	O	-0.344045	-1.970319	-0.035617
12	C	1.223520	1.326195	-0.482638
13	C	2.864897	-0.297302	-0.603273
14	N	2.537059	0.979399	-0.155763
15	C	3.450500	1.836234	0.563508
16	H	3.087668	2.861050	0.475235
17	H	3.499628	1.557430	1.621075
18	H	4.445824	1.741804	0.126039
19	O	0.723312	2.404101	-0.239726
20	O	3.956435	-0.806915	-0.492672
21	H	0.115223	0.394391	2.199845
22	C	-2.015167	-0.233187	0.201912
23	C	-2.303533	1.118702	0.417151
24	C	-3.016140	-1.098842	-0.245525
25	C	-3.593339	1.590314	0.204438
26	H	-1.507039	1.799550	0.705995
27	C	-4.304027	-0.617679	-0.456201
28	H	-2.781422	-2.144424	-0.416849
29	C	-4.596650	0.724552	-0.227878
30	H	-3.813018	2.640738	0.367417
31	H	-5.081180	-1.294262	-0.797996
32	H	-5.602557	1.098199	-0.392268

(3a*R*,4*S*,7*R*,7a*S*)-2-Methyl-4-phenyl-3a,4,7,7a-tetrahydro-1*H*-4,7-epoxyisoindole-1,3(2*H*)-dione *endo*-16i

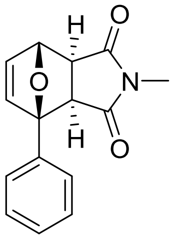

sum of electronic and thermal energies	-859.226019
sum of electronic and thermal enthalpies	-859.225075
sum of electronic and thermal free energies	-859.283115
$E_{\text{solvation}}$ (Et_2O)	-0.008753

1	C	0.392068	2.354976	0.790804
2	C	0.356670	0.935647	0.216163
3	C	-1.446085	1.892239	-0.447696
4	C	-0.719946	2.949707	0.366787
5	H	-1.103989	3.932819	0.608396
6	C	-0.733640	0.183397	1.085528
7	H	-0.544572	0.271509	2.156232
8	C	-2.021004	0.857147	0.568411
9	H	-2.669176	1.302769	1.324603
10	H	-2.143290	2.207071	-1.222978
11	O	-0.372738	1.133166	-0.995731
12	C	-0.880502	-1.282040	0.702283
13	O	-0.145301	-2.193386	0.994274
14	C	-2.756704	-0.240024	-0.184274
15	O	-3.783253	-0.129413	-0.811005
16	N	-2.038993	-1.415426	-0.055267
17	C	-2.457426	-2.657773	-0.670853
18	H	-1.785712	-3.440174	-0.317446
19	H	-3.487998	-2.879522	-0.386756
20	H	-2.399481	-2.574683	-1.758669
21	H	1.159428	2.717641	1.463518
22	C	1.683560	0.274851	-0.015125
23	C	2.336574	-0.398053	1.018334
24	C	2.304529	0.401214	-1.257890
25	C	3.601447	-0.937643	0.809949
26	H	1.847136	-0.526637	1.979347
27	C	3.567134	-0.146402	-1.465503
28	H	1.788294	0.925485	-2.055063
29	C	4.219420	-0.813116	-0.431925
30	H	4.099787	-1.466213	1.616482
31	H	4.041990	-0.049392	-2.437081
32	H	5.204297	-1.240375	-0.593970

Transition state: *exo*-16i

sum of electronic and thermal energies	-859.184570
sum of electronic and thermal enthalpies	-859.183626
sum of electronic and thermal free energies	-859.243170
$E_{solvation}$ (Et_2O)	-0.009543

1	C	0.016388	2.618125	-0.191695
2	C	-0.298478	1.300919	0.244704
3	C	1.744270	1.674719	0.851218
4	C	1.294086	2.874838	0.231056
5	H	1.909929	3.734111	0.005874
6	C	0.996106	0.353885	-1.392624
7	H	0.609725	0.864079	-2.263708
8	C	2.226570	0.582961	-0.756720
9	H	3.036777	1.177947	-1.160551
10	H	2.577562	1.540627	1.530078
11	O	0.638501	0.923867	1.149268
12	C	0.576424	-1.038315	-1.115469
13	O	-0.391399	-1.643821	-1.515473
14	C	2.584057	-0.693605	-0.033411
15	O	3.562631	-0.921409	0.642403
16	N	1.535526	-1.571236	-0.237811
17	C	1.441733	-2.874589	0.376232
18	H	0.538179	-3.347868	-0.011228
19	H	2.318250	-3.475873	0.123778
20	H	1.379500	-2.781585	1.464086
21	H	-0.618932	3.240649	-0.806102
22	C	-1.579696	0.592379	0.271942
23	C	-2.603593	0.953904	-0.607851
24	C	-1.767681	-0.480823	1.147796
25	C	-3.807530	0.262099	-0.597744
26	H	-2.453040	1.768049	-1.311494
27	C	-2.975711	-1.167447	1.155769
28	H	-0.964908	-0.767304	1.820026
29	C	-3.997884	-0.798136	0.286179
30	H	-4.597876	0.547021	-1.284974
31	H	-3.117311	-1.996493	1.841920
32	H	-4.938778	-1.339283	0.291086

2-(4-(Trifluoromethyl)phenyl)furan 14j

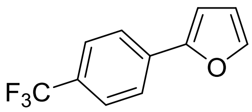

sum of electronic and thermal energies -797.658880

sum of electronic and thermal enthalpies -797.657936

sum of electronic and thermal free energies -797.710674

$E_{solvation}$ (Et_2O) -0.003538

1	C	-0.000017441	0.000005121	0.000010726
2	C	-0.000003679	-0.000002793	-0.000011500
3	C	0.000007385	0.000012840	-0.000003961
4	C	-0.000003547	-0.000005817	0.000007112
5	O	0.000009292	0.000000901	-0.000005119
6	H	0.000001703	0.000001850	-0.000003280
7	H	-0.000000232	0.000004218	-0.000001144
8	H	-0.000000213	0.000007049	-0.000001251
9	C	0.000008099	0.000000065	0.000013684
10	C	-0.000003855	-0.000002637	-0.000002578
11	C	-0.000025628	-0.000001715	-0.000008107
12	C	0.000010735	-0.000002106	-0.000007355
13	H	0.000002550	-0.000002550	-0.000001100
14	C	0.000027977	0.000001196	-0.000015663
15	H	0.000002485	0.000005102	0.000001894
16	C	-0.000012522	-0.000003108	0.000015723
17	H	-0.000007686	-0.000004488	0.000001629
18	H	-0.000009254	0.000001558	0.000003602
19	C	0.000013987	0.000035951	0.000025201
20	F	0.000008295	-0.000014573	0.000014208
21	F	-0.000010159	-0.000023880	-0.000009276
22	F	0.000001707	-0.000012185	-0.000023443

(3a*S*,4*S*,7*R*,7a*R*)-2-Methyl-4-(4-(trifluoromethyl)phenyl)-3a,4,7,7a-tetrahydro-1*H*-4,7-epoxyisoindole-1,3(2*H*)-dione *endo*-16j

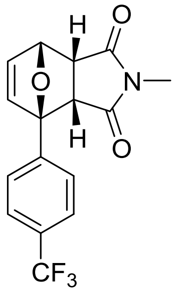

sum of electronic and thermal energies	-1196.157068
sum of electronic and thermal enthalpies	-1196.156124
sum of electronic and thermal free energies	-1196.224019
$E_{\text{solvation}}$ (Et_2O)	-0.007863

1	C	1.485339	-0.754160	1.475755
2	C	0.806378	-0.747484	0.105328
3	C	2.512867	-2.032176	-0.093540
4	C	2.542972	-1.554059	1.349520
5	H	3.331547	-1.758148	2.063065
6	C	1.741040	0.113160	-0.839728
7	H	1.215534	0.341158	-1.769730
8	C	2.945557	-0.827564	-1.003801
9	H	3.110644	-1.168572	-2.027132
10	H	2.982720	-2.984267	-0.330832
11	O	1.118622	-2.055525	-0.376778
12	C	2.318133	1.380670	-0.229285
13	C	4.139866	-0.045309	-0.497438
14	N	3.687981	1.207780	-0.096932
15	C	4.563207	2.195323	0.501754
16	H	4.789767	1.927487	1.537196
17	H	5.493212	2.234452	-0.067055
18	H	4.050539	3.156890	0.474274
19	O	1.724650	2.380458	0.106848
20	O	5.286557	-0.411817	-0.412424
21	H	1.181369	-0.136889	2.312211
22	C	-0.666740	-0.466595	0.054093
23	C	-1.134537	0.848650	0.017292
24	C	-1.571002	-1.528801	0.083956
25	C	-2.502249	1.096911	0.007301
26	H	-0.428757	1.675632	-0.000810
27	C	-2.938741	-1.279665	0.066764
28	H	-1.194062	-2.545256	0.113971
29	C	-3.399568	0.032904	0.029118
30	H	-2.874554	2.116063	-0.014696
31	H	-3.647611	-2.100769	0.089586
32	C	-4.872676	0.312481	-0.047475
33	F	-5.599729	-0.678836	0.487569
34	F	-5.282407	0.457208	-1.317947
35	F	-5.193855	1.445754	0.594782

Transition state: *endo*-16j

sum of electronic and thermal energies	-1196.116568
sum of electronic and thermal enthalpies	-1196.115623
sum of electronic and thermal free energies	-1196.185015
$E_{solvation}$ (Et_2O)	-0.008172

1	C	-1.607783	0.598261	1.552917
2	C	-0.734228	0.951016	0.485277
3	C	-2.527543	2.111689	0.205133
4	C	-2.737751	1.356635	1.392752
5	H	-3.669556	1.296201	1.938023
6	C	-1.784985	-0.141840	-1.087879
7	H	-0.909796	-0.156156	-1.723618
8	C	-2.895209	0.704357	-1.229558
9	H	-3.031717	1.406827	-2.042649
10	H	-3.063646	2.994932	-0.115732
11	O	-1.185849	2.113079	-0.063397
12	C	-2.245279	-1.390609	-0.421615
13	C	-4.092125	-0.030142	-0.705028
14	N	-3.609616	-1.228202	-0.180861
15	C	-4.417363	-2.189131	0.534450
16	H	-4.269263	-2.097449	1.615162
17	H	-5.462532	-1.992318	0.292573
18	H	-4.134724	-3.196101	0.222964
19	O	-1.610519	-2.370456	-0.092316
20	O	-5.247940	0.323217	-0.678795
21	H	-1.423940	-0.193407	2.266903
22	C	0.694002	0.638581	0.330209
23	C	1.147182	-0.655203	0.615413
24	C	1.590163	1.613711	-0.111732
25	C	2.493072	-0.958875	0.477075
26	H	0.436063	-1.426418	0.899090
27	C	2.938635	1.304215	-0.252069
28	H	1.229343	2.612138	-0.334875
29	C	3.386005	0.022498	0.047275
30	H	2.854396	-1.959023	0.695259
31	H	3.642960	2.058109	-0.586373
32	C	4.830119	-0.345494	-0.136590
33	F	5.287978	-1.065063	0.898868
34	F	5.613300	0.734783	-0.258607
35	F	5.010134	-1.093571	-1.235906

(3a*R*,4*S*,7*R*,7a*S*)-2-Methyl-4-(4-(trifluoromethyl)phenyl)-3a,4,7,7a-tetrahydro-1*H*-4,7-epoxyisoindole-1,3(2*H*)-dione *exo*-16j

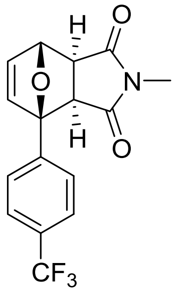

sum of electronic and thermal energies	-1196.156944
sum of electronic and thermal enthalpies	-1196.156000
sum of electronic and thermal free energies	-1196.223335
$E_{solvation}$ (Et_2O)	-0.008968

1	C	1.283847	-2.512668	0.745634
2	C	1.023117	-1.087459	0.249450
3	C	2.906090	-1.673542	-0.594225
4	C	2.445826	-2.877655	0.210007
5	H	3.015435	-3.784281	0.370635
6	C	2.026069	-0.184433	1.076055
7	H	1.940054	-0.342780	2.151955
8	C	3.367666	-0.596128	0.436817
9	H	4.138310	-0.952285	1.122171
10	H	3.583755	-1.823239	-1.433410
11	O	1.673706	-1.098493	-1.020689
12	C	1.879834	1.296348	0.753920
13	O	1.010549	2.043219	1.132666
14	C	3.842776	0.645933	-0.300620
15	O	4.823841	0.745283	-0.997258
16	N	2.939755	1.667556	-0.063629
17	C	3.086433	2.988658	-0.640031
18	H	2.333915	3.631783	-0.183565
19	H	4.090130	3.366172	-0.435686
20	H	2.937004	2.944571	-1.721394
21	H	0.650550	-3.036202	1.451147
22	C	-0.414973	-0.671853	0.145482
23	C	-1.092541	-0.155856	1.248737
24	C	-1.098363	-0.876463	-1.055392
25	C	-2.445186	0.154630	1.157690
26	H	-0.560733	0.035546	2.175414
27	C	-2.447466	-0.563271	-1.150042
28	H	-0.561443	-1.277786	-1.907969
29	C	-3.117577	-0.051128	-0.041200
30	H	-2.973674	0.563584	2.011495
31	H	-2.984070	-0.719685	-2.081031
32	C	-4.569265	0.304726	-0.180403
33	F	-5.128848	0.615793	0.996685
34	F	-5.274537	-0.709087	-0.707414
35	F	-4.742171	1.355805	-0.996313

Transition state: *exo*-16j

sum of electronic and thermal energies	-1196.114222
sum of electronic and thermal enthalpies	-1196.113278
sum of electronic and thermal free energies	-1196.182433
$E_{solvation}$ (Et_2O)	-0.009708

1	C	-1.737079	2.654386	0.134803
2	C	-1.129865	1.429595	-0.258429
3	C	-3.187925	1.323174	-0.907743
4	C	-3.031098	2.607263	-0.312635
5	H	-3.827778	3.313054	-0.124747
6	C	-2.192778	0.241481	1.369947
7	H	-1.934446	0.833041	2.237096
8	C	-3.439860	0.185922	0.729309
9	H	-4.361205	0.602095	1.117908
10	H	-3.959022	0.988087	-1.590553
11	O	-1.936719	0.833266	-1.171410
12	C	-1.477417	-1.031820	1.112723
13	O	-0.397513	-1.398612	1.516041
14	C	-3.509239	-1.149921	0.028832
15	O	-4.410575	-1.594404	-0.645385
16	N	-2.295714	-1.775744	0.249280
17	C	-1.920918	-3.036722	-0.347425
18	H	-1.950302	-2.962313	-1.437680
19	H	-0.908256	-3.265067	-0.011896
20	H	-2.605763	-3.826841	-0.030194
21	H	-1.267951	3.418567	0.738793
22	C	0.275787	1.020055	-0.235395
23	C	1.149462	1.569300	0.707658
24	C	0.731799	0.027980	-1.108343
25	C	2.467874	1.144443	0.767590
26	H	0.790645	2.313731	1.412444
27	C	2.052095	-0.396092	-1.049075
28	H	0.045341	-0.408201	-1.826657
29	C	2.916414	0.162940	-0.113119
30	H	3.146973	1.558650	1.505160
31	H	2.411107	-1.169179	-1.720125
32	C	4.356922	-0.259544	-0.085738
33	F	4.883436	-0.153158	1.142529
34	F	5.107922	0.499871	-0.900442
35	F	4.509093	-1.529163	-0.487224

Furan 20a

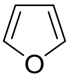

sum of electronic and thermal energies	-229.847734
sum of electronic and thermal enthalpies	-229.846790
sum of electronic and thermal free energies	-229.877642

1	C	1.088806	-0.348196	-0.000173
2	C	0.717346	0.956245	0.000099
3	C	-0.717122	0.956400	-0.000022
4	C	-1.088886	-0.347956	-0.000091
5	O	-0.000136	-1.153045	0.000175
6	H	2.043112	-0.851421	-0.000277
7	H	1.373476	1.813719	0.000181
8	H	-1.373044	1.814030	-0.000033
9	H	-2.043323	-0.850931	-0.000139

2,5-Dihydrofuran 21a

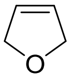

sum of electronic and thermal energies	-231.010467
sum of electronic and thermal enthalpies	-231.009523
sum of electronic and thermal free energies	-231.042625

1	C	-0.001748	-0.373400	1.167129
2	C	-0.001748	1.043513	0.663388
3	C	-0.001748	1.043513	-0.663388
4	C	-0.001748	-0.373400	-1.167129
5	O	0.008792	-1.182455	0.000000
6	H	0.881246	-0.599058	1.781861
7	H	-0.002969	1.909591	1.314651
8	H	-0.002969	1.909591	-1.314651
9	H	0.881246	-0.599058	-1.781861
10	H	-0.892468	-0.601394	1.770232
11	H	-0.892468	-0.601394	-1.770232

(3a*R*,4*R*,7*S*,7a*S*)-2-Methyl-3a,4,7,7a-tetrahydro-1*H*-4,7-epoxyisoindole-1,3(2*H*)-dione 22a

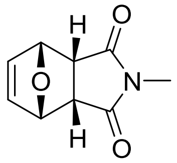

sum of electronic and thermal energies	-628.348604
sum of electronic and thermal enthalpies	-628.347660
sum of electronic and thermal free energies	-628.394227

1	C	-1.312489	0.656002	1.388029
2	C	-1.698684	1.057981	-0.027598
3	C	-1.689124	-1.072984	-0.034483
4	C	-1.306774	-0.676961	1.383782
5	H	-0.993264	-1.359724	2.163385
6	C	-0.456490	0.769430	-0.949397
7	H	-0.597126	1.222521	-1.932035
8	C	-0.449419	-0.767614	-0.954232
9	H	-0.586347	-1.215457	-1.939718
10	H	-2.156030	-2.042853	-0.191203
11	O	-2.553594	-0.010067	-0.423556
12	C	0.880925	1.174142	-0.359422
13	C	0.892716	-1.157633	-0.366716
14	N	1.590548	0.009753	-0.080711
15	C	2.885790	-0.004250	0.567031
16	H	2.798472	-0.423128	1.572812
17	H	3.577977	-0.618603	-0.011829
18	H	3.238461	1.025938	0.618533
19	O	1.277806	2.292149	-0.134058
20	O	1.311260	-2.267978	-0.141278
21	H	-1.004825	1.336612	2.171857
22	H	-2.173626	2.024919	-0.177649

3-Methoxyfuran 17a

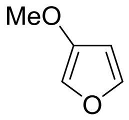

sum of electronic and thermal energies	-344.283145
sum of electronic and thermal enthalpies	-344.282201
sum of electronic and thermal free energies	-344.319070

1	C	-0.273590	-0.993967	0.000249
2	C	0.213632	0.279327	0.000145
3	C	-0.918204	1.158387	-0.000015
4	C	-2.001871	0.344334	-0.000122
5	O	-1.636433	-0.954688	-0.000108
6	H	0.177761	-1.972169	0.000566
7	H	-0.896021	2.237232	-0.000055
8	H	-3.064085	0.533843	-0.000324
9	O	1.491524	0.717909	0.000249
10	C	2.450487	-0.316989	-0.000265
11	H	3.430596	0.159553	-0.001121
12	H	2.344830	-0.945220	0.893949
13	H	2.343470	-0.945555	-0.894087

3-Methoxy-2,5-dihydrofuran 18a

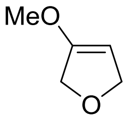

sum of electronic and thermal energies	-345.454717
sum of electronic and thermal enthalpies	-345.453772
sum of electronic and thermal free energies	-345.493903

1	C	1.700183	0.963704	0.000596
2	C	0.198678	1.051444	-0.000047
3	C	-0.289992	-0.188657	0.000077
4	C	0.821379	-1.200610	0.000755
5	O	2.005838	-0.429753	-0.001588
6	H	2.151685	1.432434	-0.885187
7	H	-0.350600	1.983343	-0.000121
8	H	0.777912	-1.847200	-0.886651
9	H	2.150456	1.429901	0.888416
10	H	0.778984	-1.844378	0.890332
11	O	-1.549239	-0.658674	0.000013
12	C	-2.550430	0.339435	-0.000250
13	H	-2.465145	0.969474	-0.893797
14	H	-3.509557	-0.177746	-0.000346
15	H	-2.465430	0.969687	0.893172

(3a*S*,4*R*,7*R*,7a*R*)-5-Methoxy-2-methyl-3a,4,7,7a-tetrahydro-1*H*-4,7-epoxyisoindole-1,3(2*H*)-dione 19a

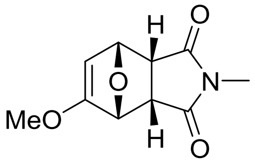

sum of electronic and thermal energies	-742.794755
sum of electronic and thermal enthalpies	-742.793811
sum of electronic and thermal free energies	-742.844623

1	C	1.559470	0.367021	-0.185387
2	C	1.485595	-0.526044	1.041856
3	C	0.946107	-1.754597	-0.612241
4	C	1.229940	-0.397397	-1.235341
5	H	1.055141	-0.127765	-2.267277
6	C	-0.036548	-0.771857	1.343257
7	H	-0.153528	-1.245957	2.318817
8	C	-0.425466	-1.663968	0.151249
9	H	-0.787918	-2.654758	0.430991
10	H	1.068133	-2.634636	-1.240416
11	O	1.845018	-1.788324	0.500929
12	C	-0.925458	0.454497	1.237711
13	C	-1.495988	-0.885802	-0.587733
14	N	-1.715619	0.301676	0.100616
15	C	-2.652915	1.302288	-0.363580
16	H	-2.316342	1.723290	-1.314736
17	H	-3.634174	0.846755	-0.510468
18	H	-2.703192	2.082582	0.396190
19	O	-0.958683	1.409662	1.974216
20	O	-2.065661	-1.188247	-1.608905
21	H	2.099887	-0.247679	1.895571
22	O	1.789092	1.674141	-0.033899
23	C	1.613692	2.438159	-1.214526
24	H	0.586409	2.332098	-1.583592
25	H	1.813003	3.475276	-0.948848
26	H	2.313214	2.107406	-1.990880

Cyclopenta-1,3-diene 20b

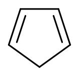

sum of electronic and thermal energies	-193.906541
sum of electronic and thermal enthalpies	-193.905597
sum of electronic and thermal free energies	-193.937243

1	C	-1.177322	0.280732	-0.000065
2	C	-0.735378	-0.988281	0.000075
3	C	0.735337	-0.988312	-0.000068
4	C	1.177334	0.280685	0.000022
5	H	-2.209368	0.609610	-0.000122
6	H	-1.349333	-1.881735	0.000129
7	H	1.349257	-1.881790	-0.000083
8	H	2.209394	0.609520	0.000045
9	C	0.000025	1.214731	0.000012
10	H	0.000072	1.873628	-0.879211
11	H	0.000003	1.873440	0.879384

Cyclopentene 21b

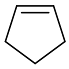

sum of electronic and thermal energies	-195.100997
sum of electronic and thermal enthalpies	-195.100053
sum of electronic and thermal free energies	-195.132903

1	C	1.228575	-0.320380	0.114167
2	C	0.665853	1.072036	-0.051241
3	C	-0.665853	1.072036	-0.051241
4	C	-1.228575	-0.320380	0.114167
5	H	2.057050	-0.533599	-0.569464
6	H	1.290816	1.957569	-0.114046
7	H	-1.290816	1.957569	-0.114046
8	H	-2.057050	-0.533599	-0.569464
9	H	1.618542	-0.453767	1.132668
10	H	-1.618542	-0.453767	1.132668
11	C	0.000000	-1.218518	-0.150761
12	H	0.000000	-2.128653	0.453948
13	H	0.000000	-1.520517	-1.202808

(3a*R*,4*S*,7*R*,7a*S*)-2-Methyl-3a,4,7,7a-tetrahydro-1*H*-4,7-methanoisoindole-1,3(2*H*)-dione 22b

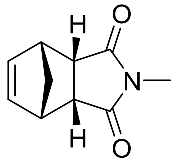

sum of electronic and thermal energies	-592.437502
sum of electronic and thermal enthalpies	-592.436558
sum of electronic and thermal free energies	-592.483435

1	C	-1.301583	0.658184	1.394275
2	C	-1.669416	1.121249	-0.005033
3	C	-1.659830	-1.134976	-0.013171
4	C	-1.295350	-0.678980	1.389301
5	H	-0.977652	-1.340239	2.187581
6	C	-0.451247	0.773056	-0.929458
7	H	-0.574837	1.221867	-1.918454
8	C	-0.444743	-0.770045	-0.934934
9	H	-0.564358	-1.212458	-1.927277
10	H	-1.995596	-2.166129	-0.119393
11	C	0.894732	1.174316	-0.354645
12	C	0.905267	-1.157990	-0.362211
13	N	1.606963	0.009245	-0.086620
14	C	2.913689	-0.005456	0.536795
15	H	2.844695	-0.424415	1.543953
16	H	3.594754	-0.620753	-0.054261
17	H	3.267799	1.024559	0.582006
18	O	1.298260	2.291391	-0.134951
19	O	1.328758	-2.267820	-0.141891
20	H	-2.013606	2.150322	-0.104090
21	C	-2.631482	-0.009457	-0.425589
22	H	-3.554768	-0.015251	0.158597
23	H	-2.862265	-0.006617	-1.496692
24	H	-0.989284	1.316424	2.197128

2-Methoxycyclopenta-1,3-diene 17b

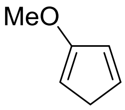

sum of electronic and thermal energies	-308.351390
sum of electronic and thermal enthalpies	-308.350445
sum of electronic and thermal free energies	-308.387915

1	C	0.234825	-1.032358	-0.000028
2	C	-0.244123	0.229135	-0.000010
3	C	0.860833	1.198774	-0.000010
4	C	2.017717	0.522076	0.000009
5	H	-0.328859	-1.954834	-0.000074
6	H	0.712004	2.271868	-0.000003
7	H	3.015159	0.944451	0.000034
8	O	-1.513175	0.687718	-0.000015
9	C	-2.499986	-0.320444	0.000023
10	H	-3.466027	0.184494	0.000079
11	H	-2.409683	-0.951736	-0.892845
12	H	-2.409592	-0.951759	0.892865
13	C	1.736578	-0.956535	0.000010
14	H	2.178633	-1.444029	0.879891
15	H	2.178709	-1.444087	-0.879797

1-Methoxycyclopent-1-ene 18b

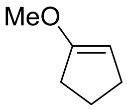

sum of electronic and thermal energies -309.547157

sum of electronic and thermal enthalpies -309.546213

sum of electronic and thermal free energies -309.583537

1	C	1.656922	1.084160	0.000984
2	C	0.150852	1.048770	-0.000086
3	C	-0.305339	-0.208014	0.000135
4	C	0.777607	-1.249365	0.001267
5	H	2.050506	1.614075	-0.875010
6	H	-0.458739	1.944574	-0.000360
7	H	0.684560	-1.899314	-0.876151
8	H	2.049052	1.610377	0.879888
9	H	0.686262	-1.895251	0.881892
10	O	-1.575023	-0.669383	-0.000058
11	C	-2.572742	0.328214	-0.000408
12	H	-2.489962	0.960624	-0.893113
13	H	-3.533312	-0.187318	-0.000528
14	H	-2.490318	0.960897	0.892137
15	C	2.079998	-0.414213	-0.001854
16	H	2.692237	-0.646493	0.872493
17	H	2.686118	-0.644422	-0.881014

(3a*R*,4*R*,7*R*,7a*S*)-5-Methoxy-2-methyl-3a,4,7,7a-tetrahydro-1*H*-4,7-methanoisoindole-1,3(2*H*)-dione 19b

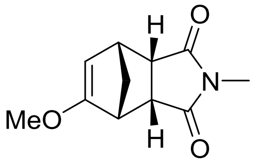

sum of electronic and thermal energies	-706.884899
sum of electronic and thermal enthalpies	-706.883954
sum of electronic and thermal free energies	-706.935027

1	C	1.524182	0.462681	-0.163872
2	C	1.501331	-0.398572	1.081054
3	C	1.010347	-1.703003	-0.689511
4	C	1.241724	-0.301856	-1.232309
5	H	1.045174	0.008428	-2.250339
6	C	0.001680	-0.781298	1.328015
7	H	-0.109956	-1.278495	2.294759
8	C	-0.336055	-1.675382	0.113562
9	H	-0.665197	-2.682961	0.381708
10	H	1.069496	-2.512649	-1.416665
11	C	-0.963925	0.387929	1.246724
12	C	-1.452571	-0.944589	-0.607827
13	N	-1.745658	0.209928	0.107814
14	C	-2.745147	1.159384	-0.332456
15	H	-2.451150	1.598630	-1.289356
16	H	-3.704825	0.654346	-0.460376
17	H	-2.820048	1.934145	0.430896
18	O	-1.056911	1.323333	2.003730
19	O	-2.004633	-1.256606	-1.636140
20	H	2.005960	0.024843	1.949024
21	O	1.678584	1.787860	-0.026031
22	C	1.489548	2.527835	-1.217102
23	H	0.471914	2.380589	-1.599554
24	H	1.646577	3.575510	-0.963627
25	H	2.209261	2.215160	-1.983175
26	C	2.007659	-1.727835	0.489531
27	H	3.047988	-1.676095	0.160529
28	H	1.865462	-2.579421	1.164135

7 References

1. M. N. Pennell, R. W. Foster, P. G. Turner, H. C. Hailes, C. J. Tame, T. D. Sheppard, *Chem. Commun.* **2014**, *50*, 1302–1304.
2. S. P. Borikar, V. Paul, V. G. Puranik, V. T Sathe, S. Lagunas-Rivera, M. Ordónez, *Synthesis* **2011**, 1595–1598.
3. **Gaussian09 Reference**: Revision A.02, M. J. Frisch, G. W. Trucks, H. B. Schlegel, G. E. Scuseria, M. A. Robb, J. R. Cheeseman, G. Scalmani, V. Barone, B. Mennucci, G. A. Petersson, H. Nakatsuji, M. Caricato, X. Li, H. P. Hratchian, A. F. Izmaylov, J. Bloino, G. Zheng, J. L. Sonnenberg, M. Hada, M. Ehara, K. Toyota, R. Fukuda, J. Hasegawa, M. Ishida, T. Nakajima, Y. Honda, O. Kitao, H. Nakai, T. Vreven, J. A. Montgomery, Jr., J. E. Peralta, F. Ogliaro, M. Bearpark, J. J. Heyd, E. Brothers, K. N. Kudin, V. N. Staroverov, R. Kobayashi, J. Normand, K. Raghavachari, A. Rendell, J. C. Burant, S. S. Iyengar, J. Tomasi, M. Cossi, N. Rega, J. M. Millam, M. Klene, J. E. Knox, J. B. Cross, V. Bakken, C. Adamo, J. Jaramillo, R. Gomperts, R. E. Stratmann, O. Yazyev, A. J. Austin, R. Cammi, C. Pomelli, J. W. Ochterski, R. L. Martin, K. Morokuma, V. G. Zakrzewski, G. A. Voth, P. Salvador, J. J. Dannenberg, S. Dapprich, A. D. Daniels, O. Farkas, J. B. Foresman, J. V. Ortiz, J. Cioslowski, and D. J. Fox, Gaussian, Inc., Wallingford CT, 2009
4. C. Peng, P. Y. Ayala, H. B. Schlegel, M. J. Frisch, *J. Comput. Chem*. **1996**, *17*, 49–56.
5. K. Fukui, S. Kato, H. Fujimoto, *J. Am, Chem. Soc.* **1975**, *97*, 1–7.

8 NMR Spectra

6,6-Diethoxy-1-phenylhex-4-yn-3-ol 1a

^{1}H NMR (500 MHz, $CDCl_3$)

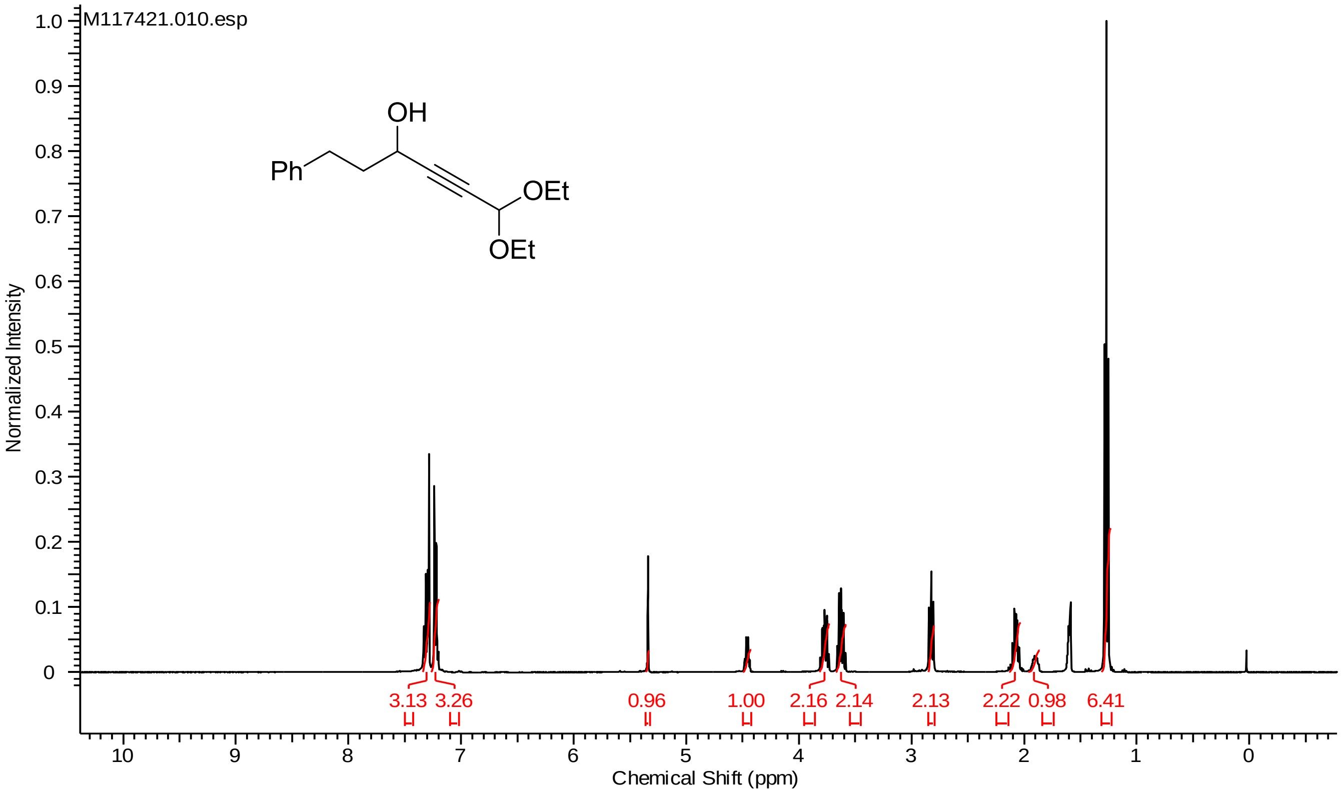

^{13}C NMR (125 MHz, $CDCl_3$)

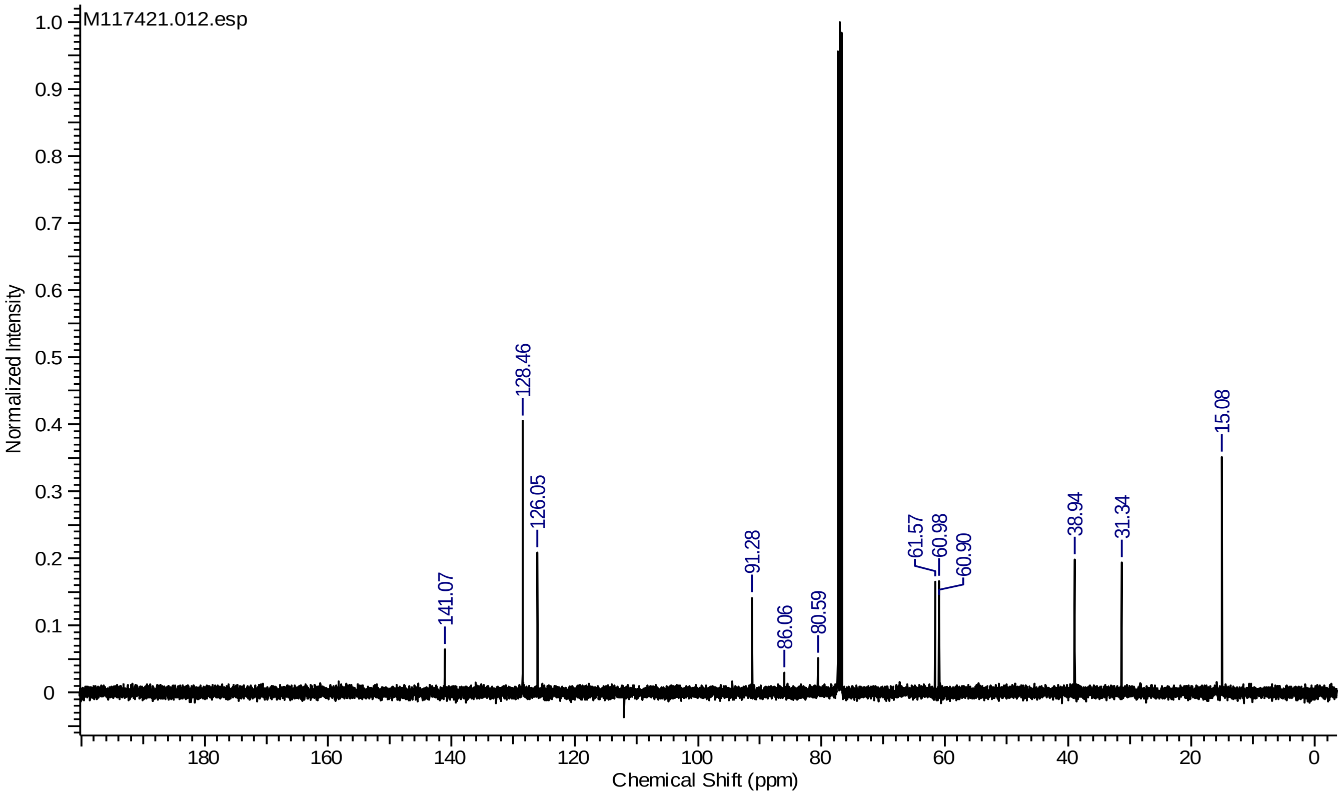

1-Cyclohexyl-4,4-diethoxybut-2-yn-1-ol 1c

^{1}H NMR (500 MHz, $CDCl_3$)

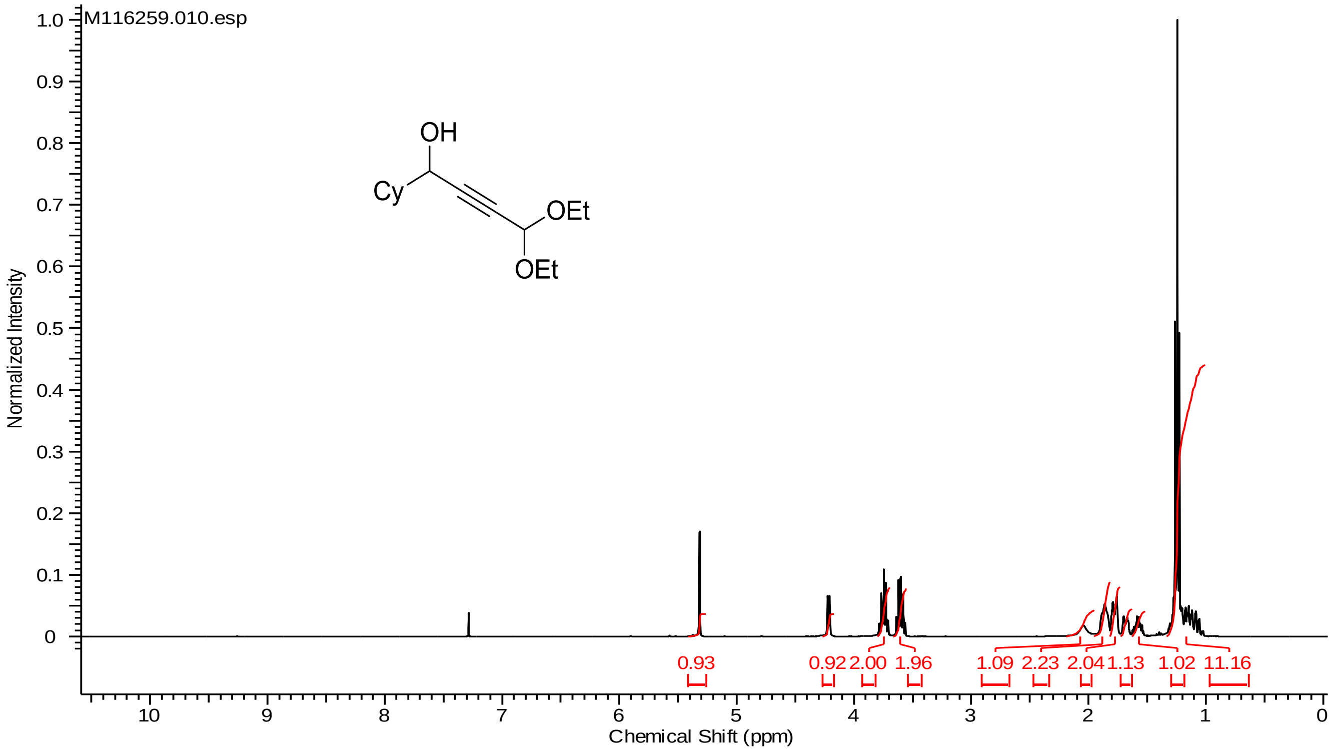

^{13}C NMR (125 MHz, $CDCl_3$)

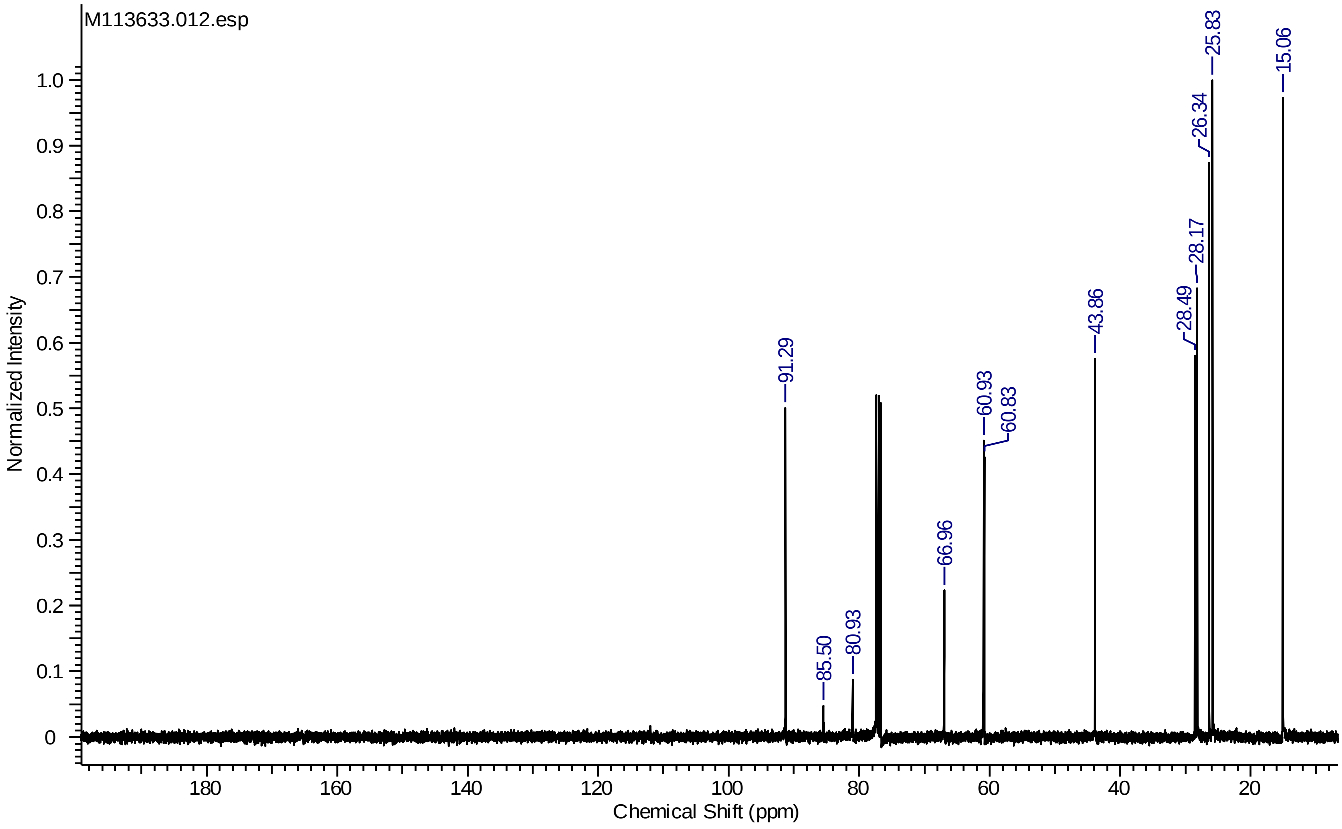

1-Cyclopropyl-4,4-diethoxybut-2-yn-1-ol 1d

^{1}H NMR (500 MHz, $CDCl_3$)

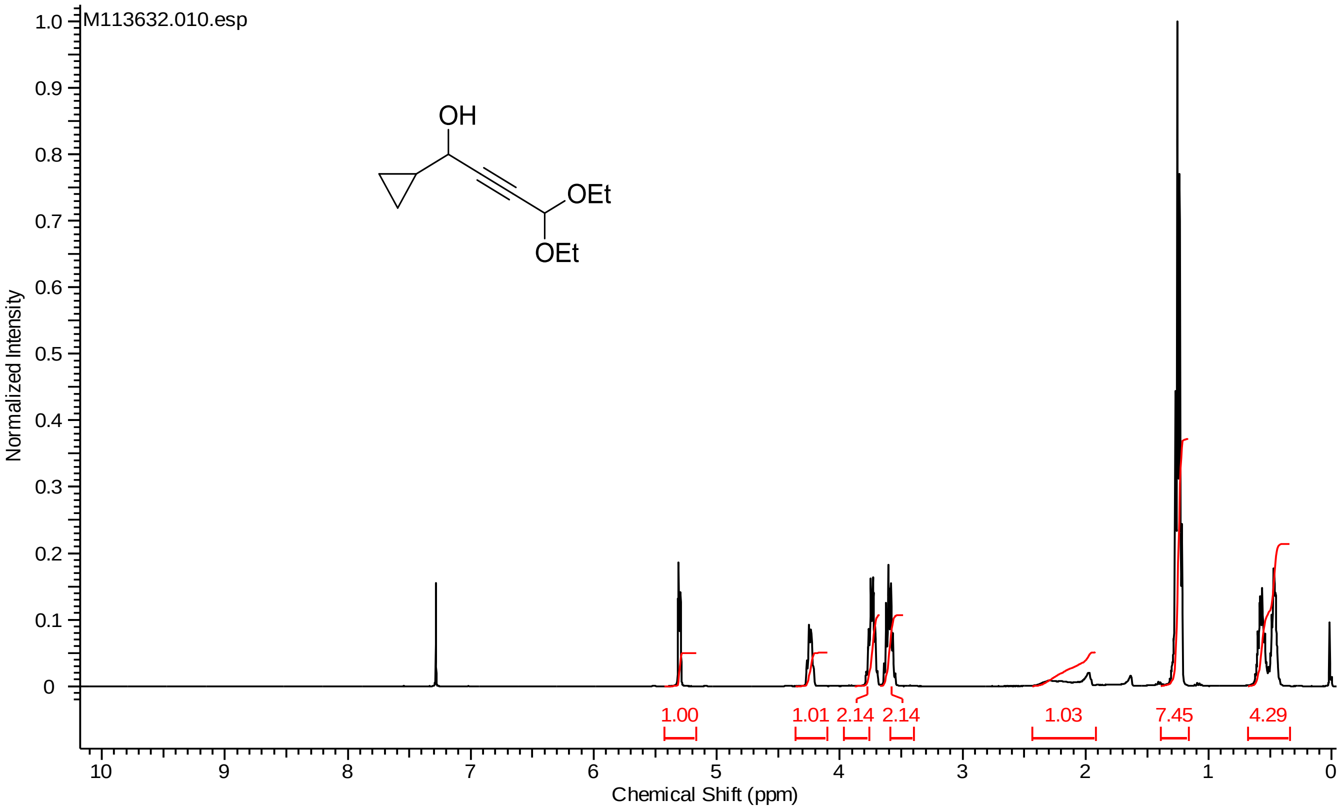

^{13}C NMR (125 MHz, $CDCl_3$)

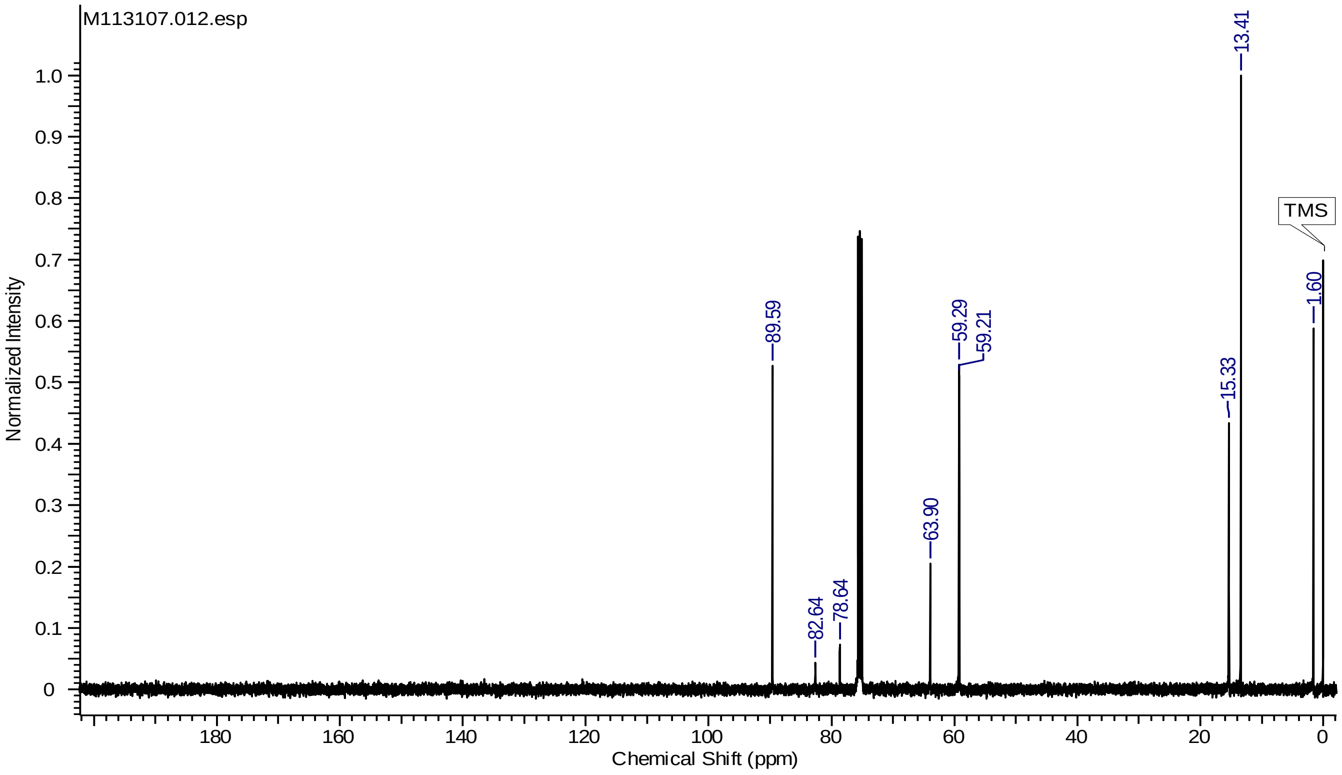

tert-Butyl 4-(5,5-diethoxy-2-hydroxypent-3-yn-1-yl)piperidine-1-carboxylate 1e

^{1}H NMR (500 MHz, $CDCl_3$)

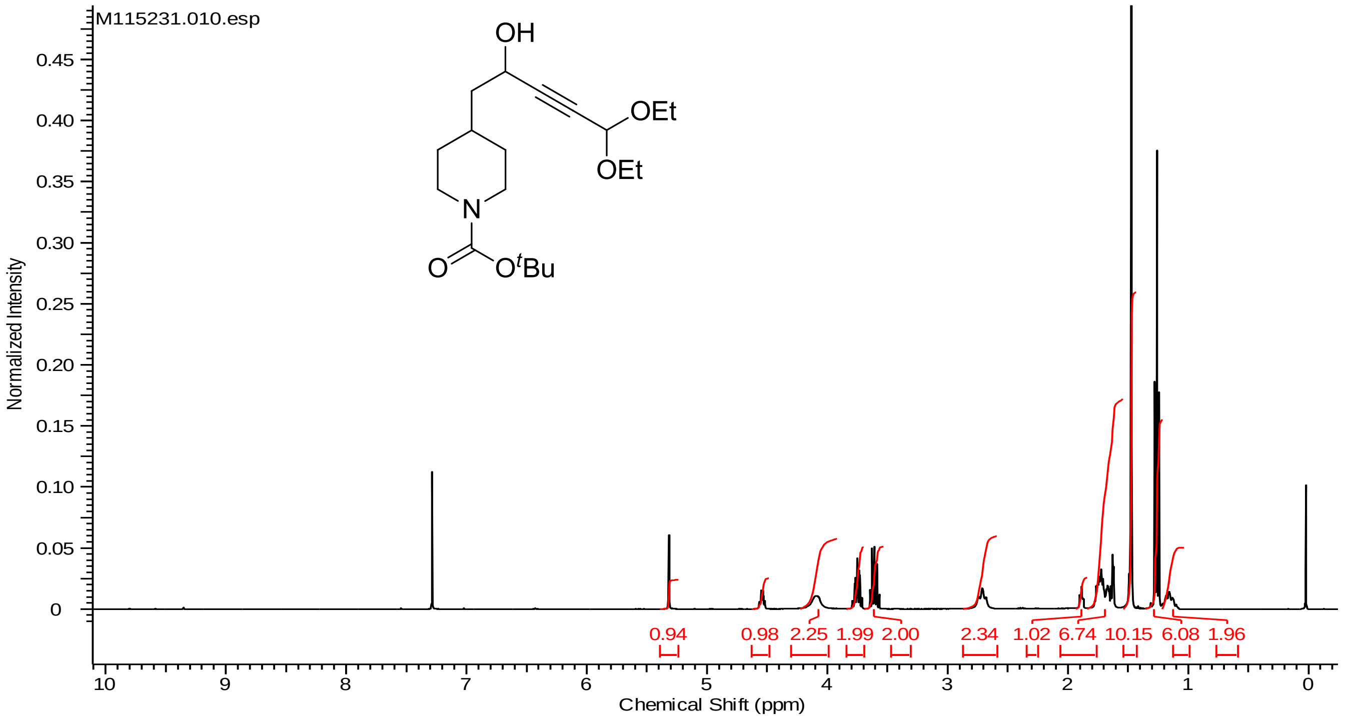

^{13}C NMR (125 MHz, $CDCl_3$)

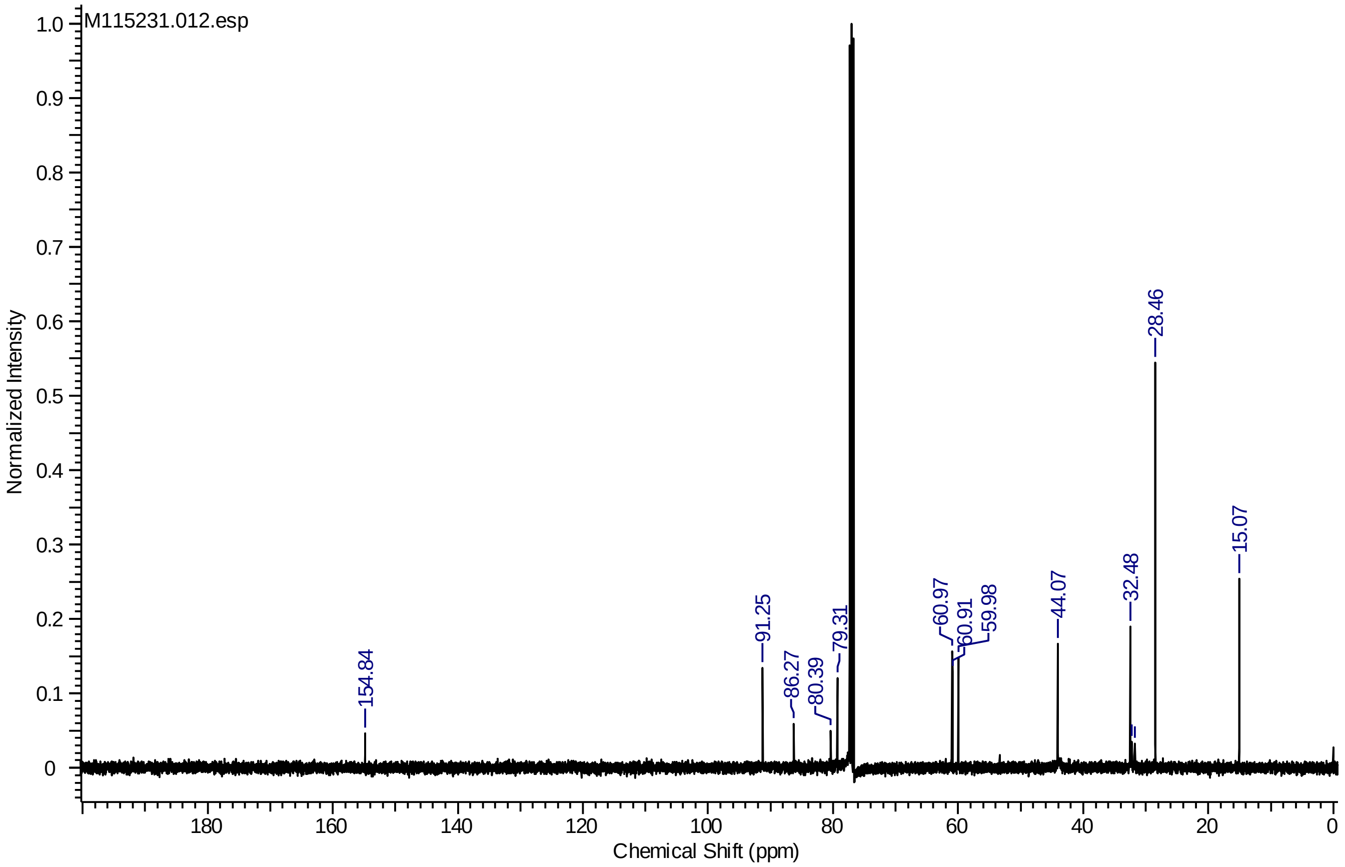

4,4-Diethoxy-1-phenylbut-2-yn-1-ol 1f

^{1}H NMR (500 MHz, $CDCl_3$)

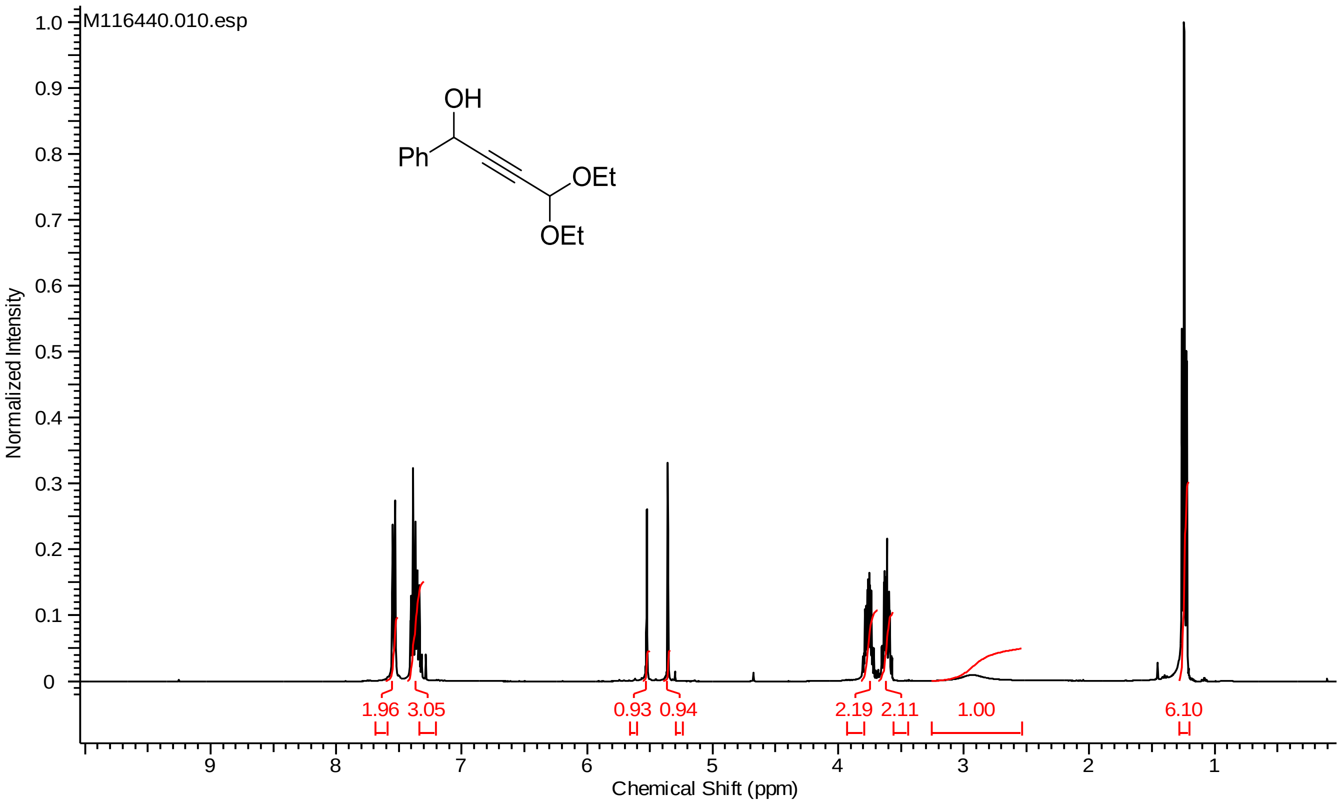

^{13}C NMR (125 MHz, $CDCl_3$)

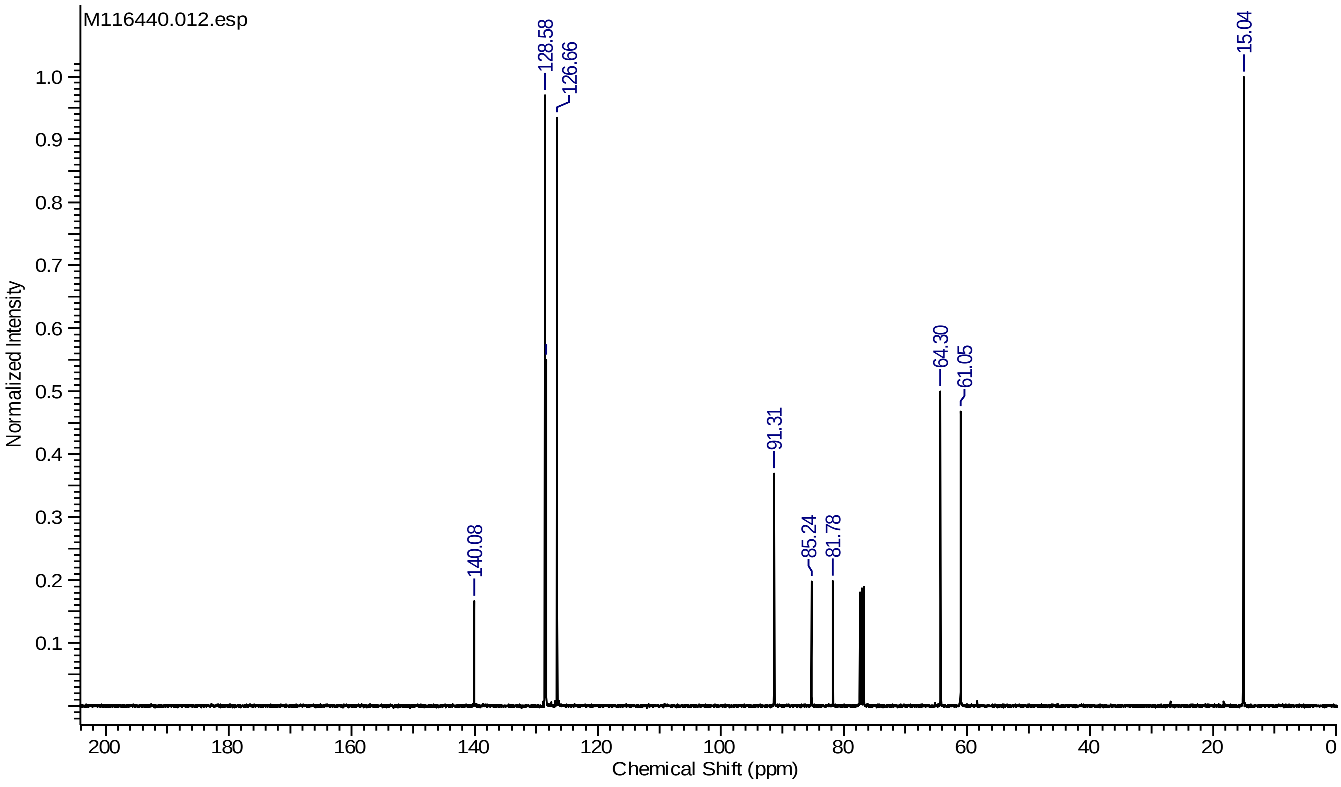

4,4-Diethoxy-1-(4-(trifluoromethyl)phenyl)but-2-yn-1-ol 1g

^{1}H NMR (500 MHz, $CDCl_3$)

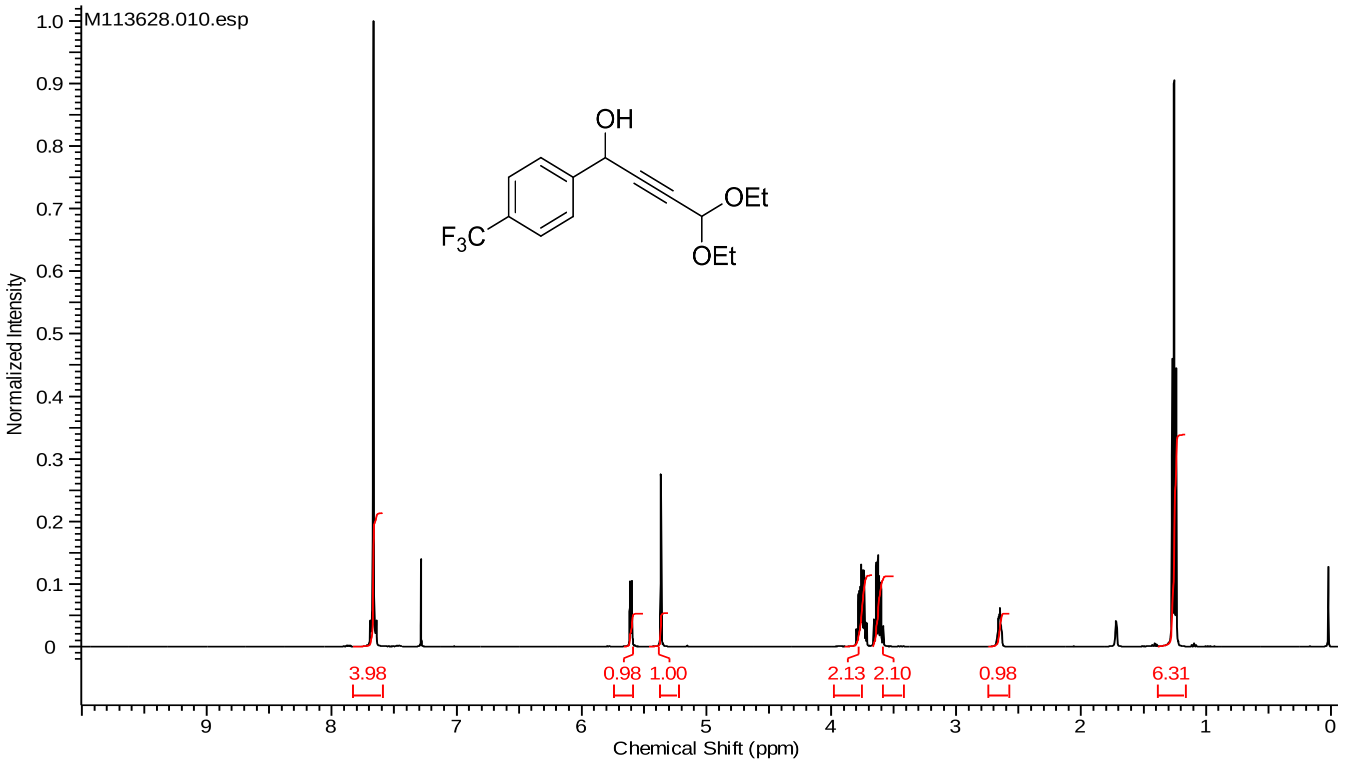

^{13}C NMR (125 MHz, $CDCl_3$)

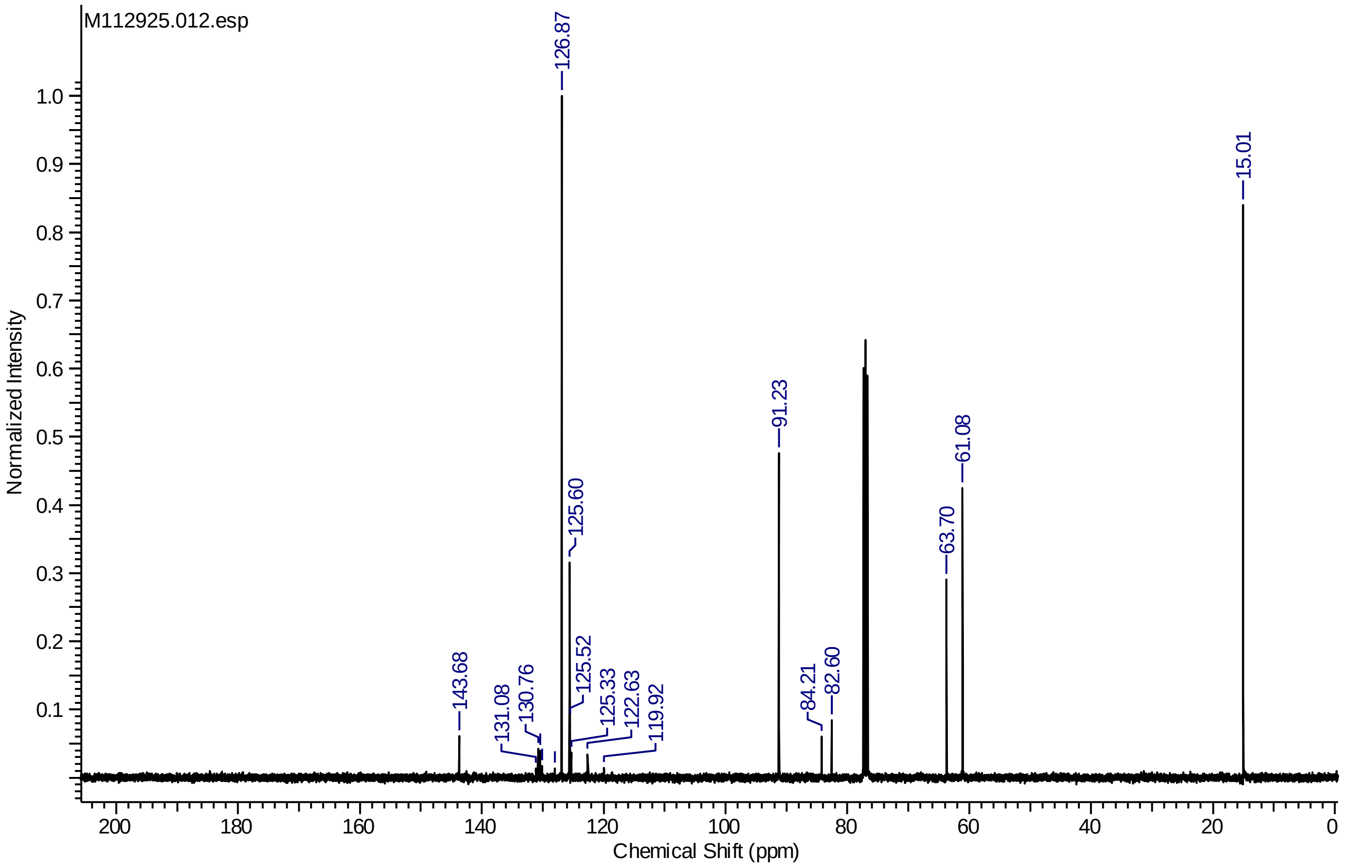

1-(4-Bromophenyl)-4,4-diethoxybut-2-yn-1-ol 1h

^{1}H NMR (500 MHz, $CDCl_3$)

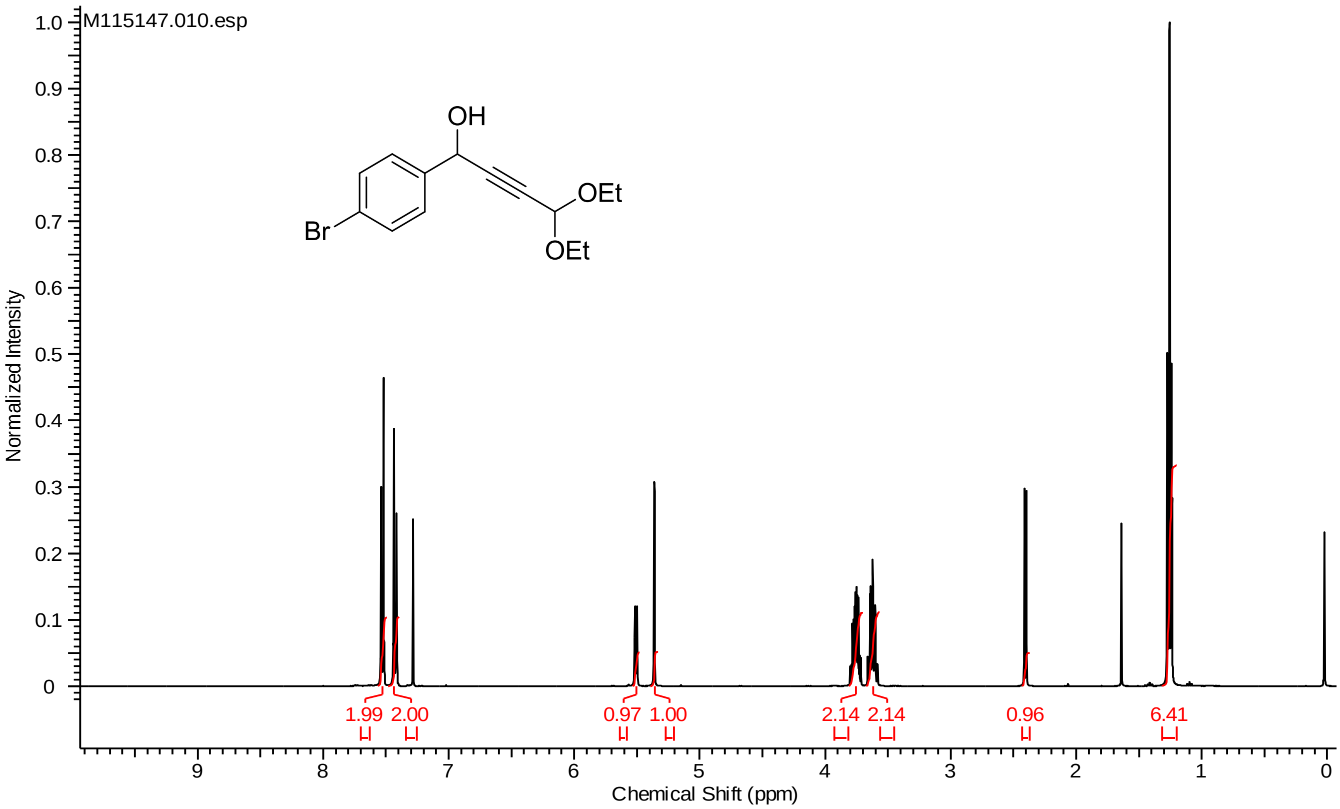

^{13}C NMR (125 MHz, $CDCl_3$)

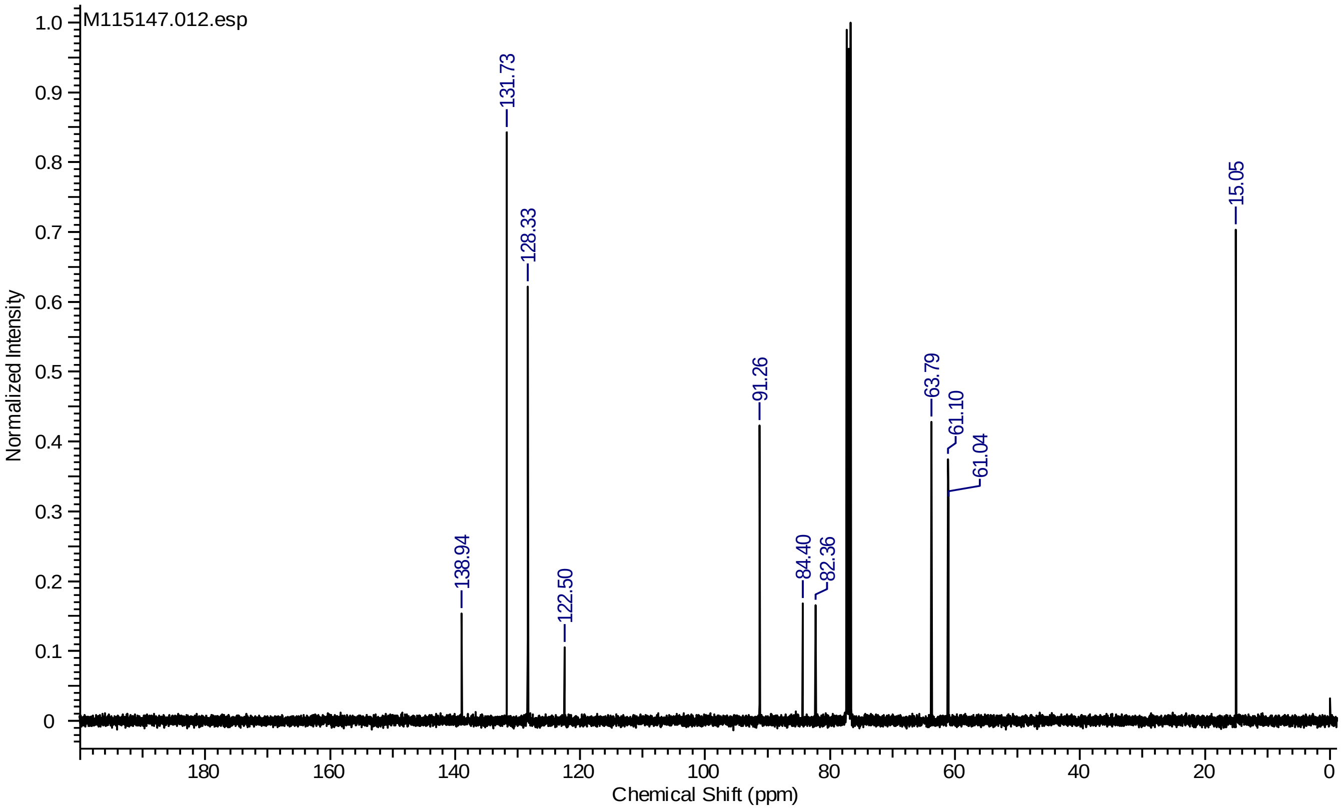

4,4-Diethoxy-1-(4-methoxyphenyl)but-2-yn-1-ol 1i

^{1}H NMR (500 MHz, DMSO-d$_6$)

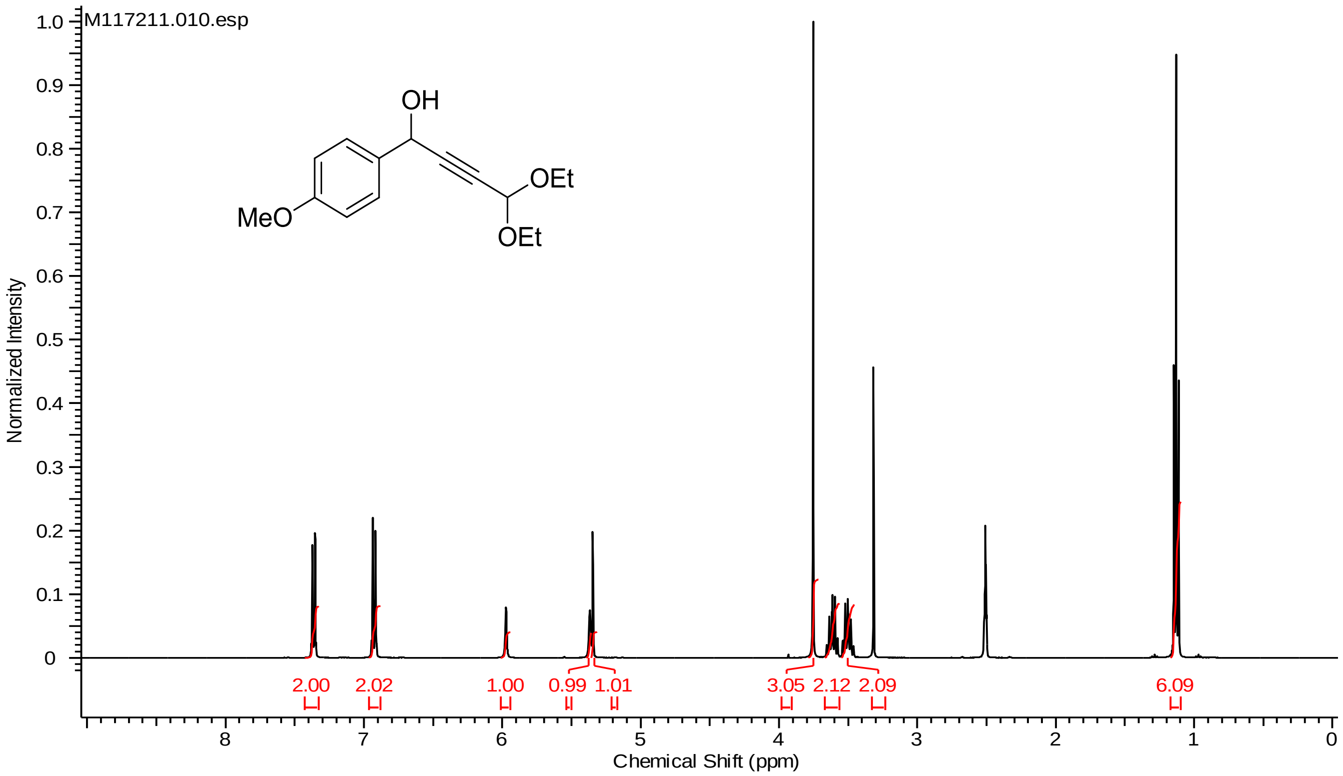

^{13}C NMR (125 MHz, DMSO-d$_6$)

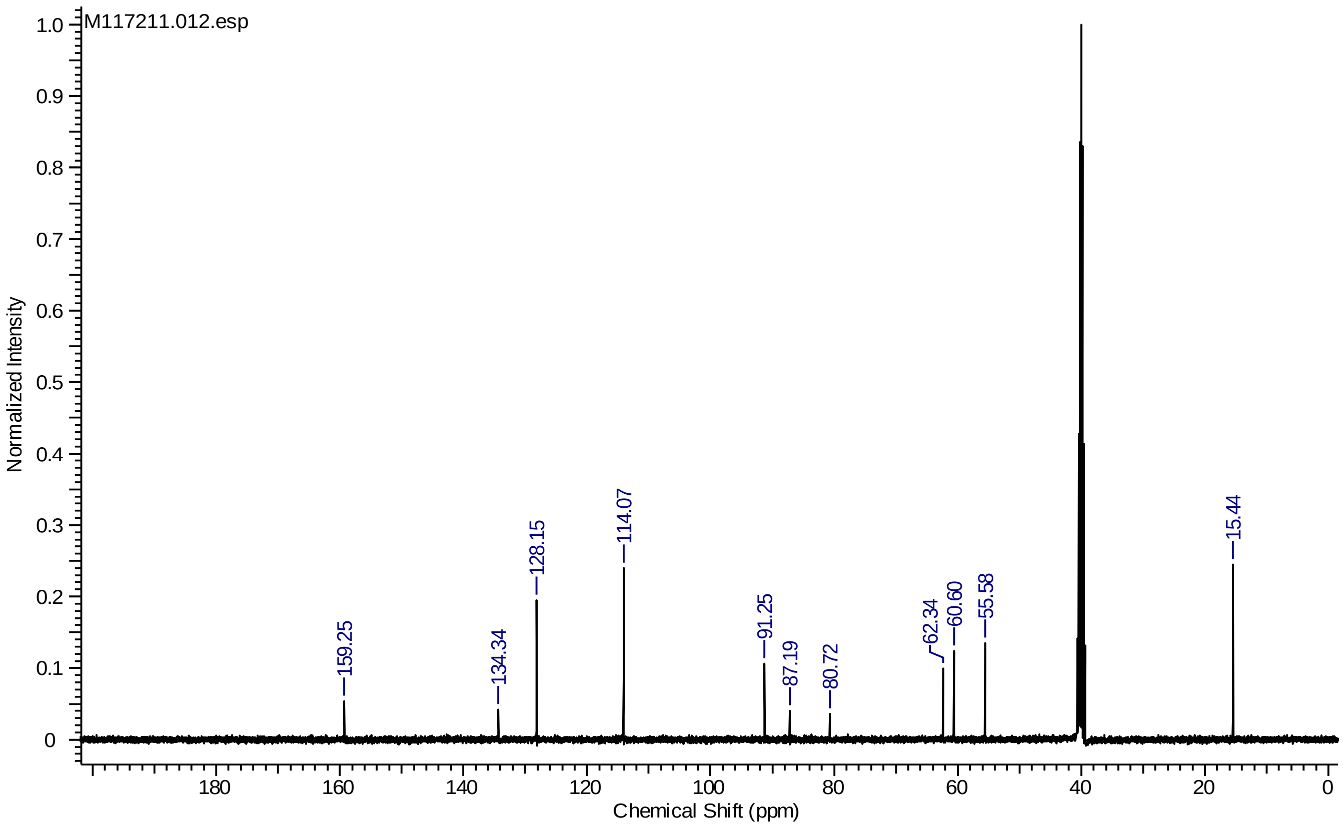

Methyl 3-(4,4-diethoxy-1-hydroxybut-2-yn-1-yl)benzoate 1j

^{1}H NMR (500 MHz, MeOH-d$_4$)

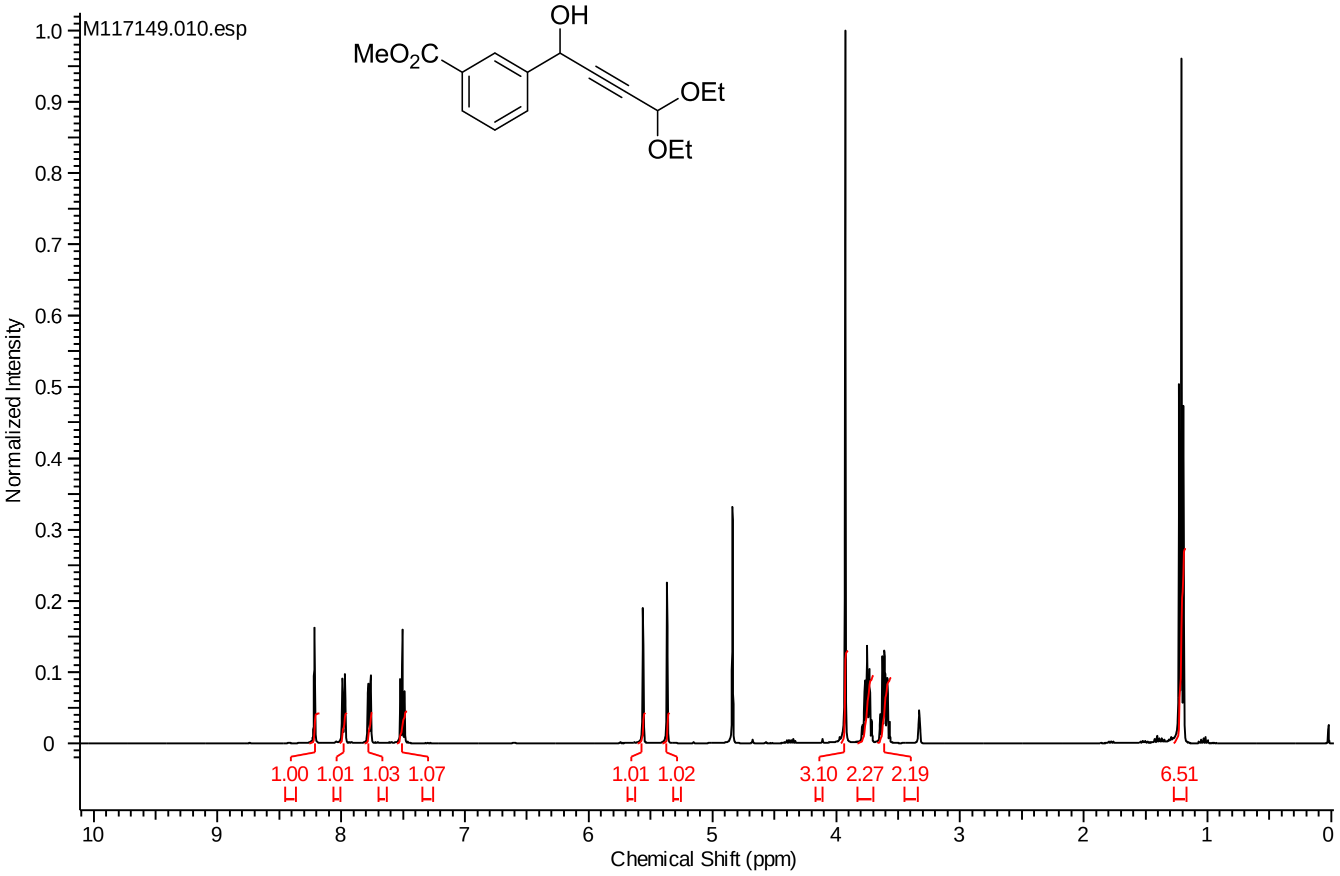

^{13}C NMR (125 MHz, MeOH-d$_4$)

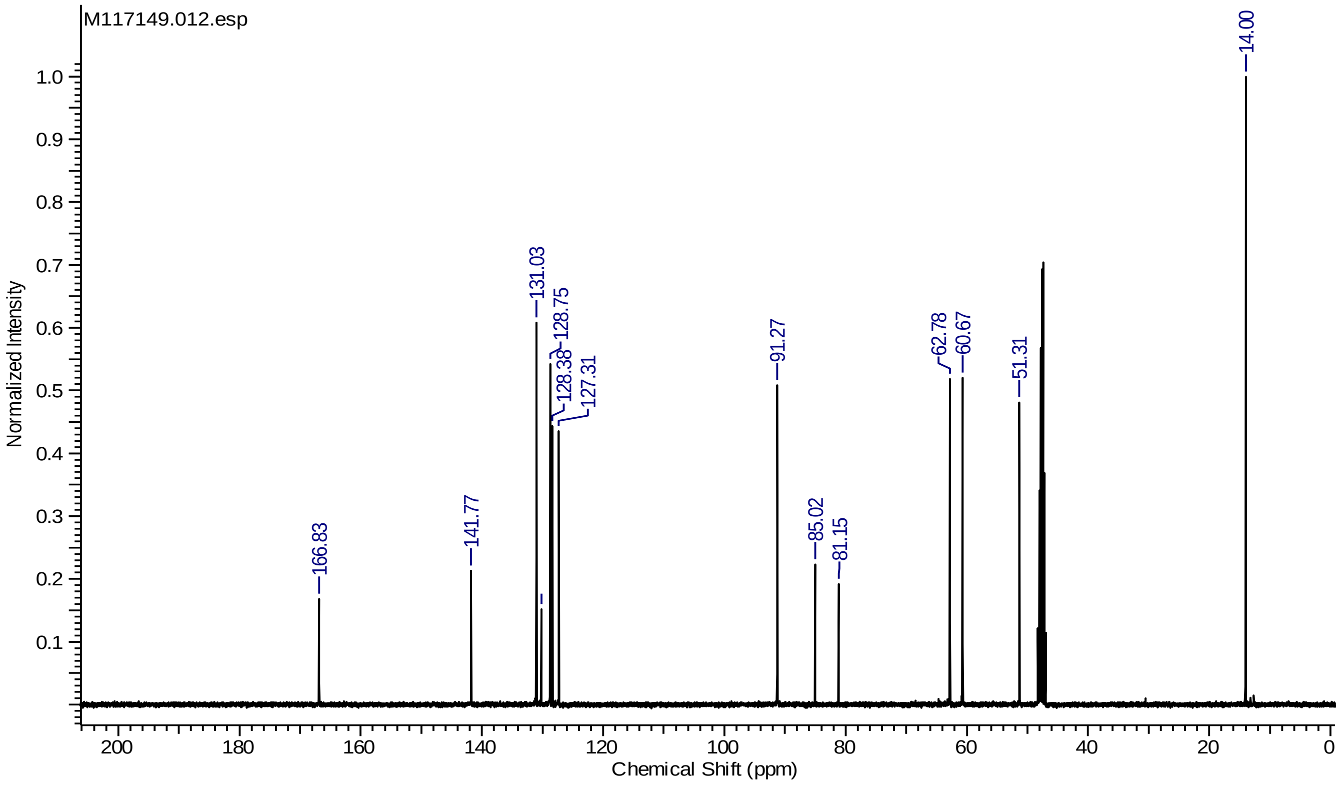

4,4-Diethoxy-1-(*o*-tolyl)but-2-yn-1-ol 1k

^{1}H NMR (500 MHz, $CDCl_3$)

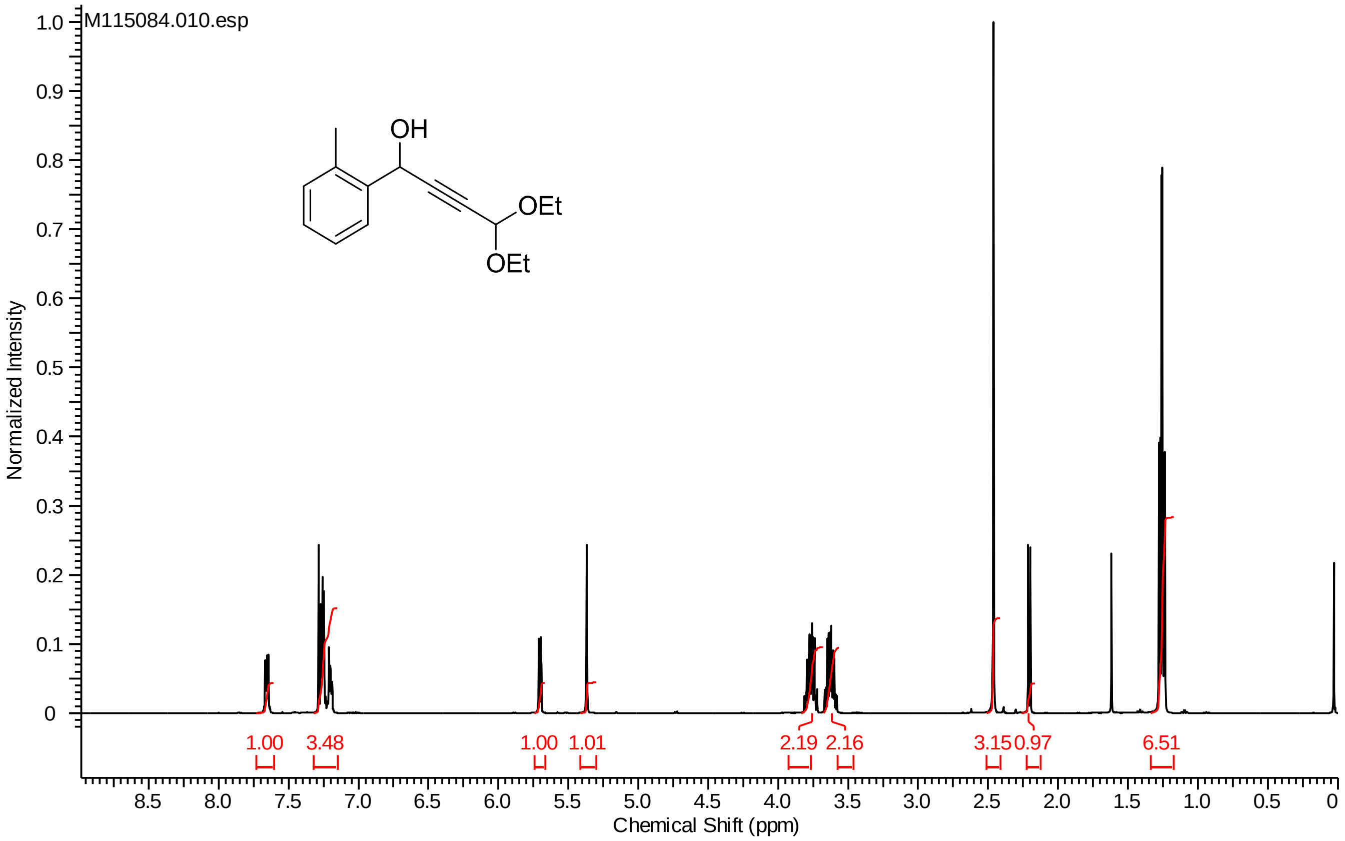

^{13}C NMR (125 MHz, $CDCl_3$)

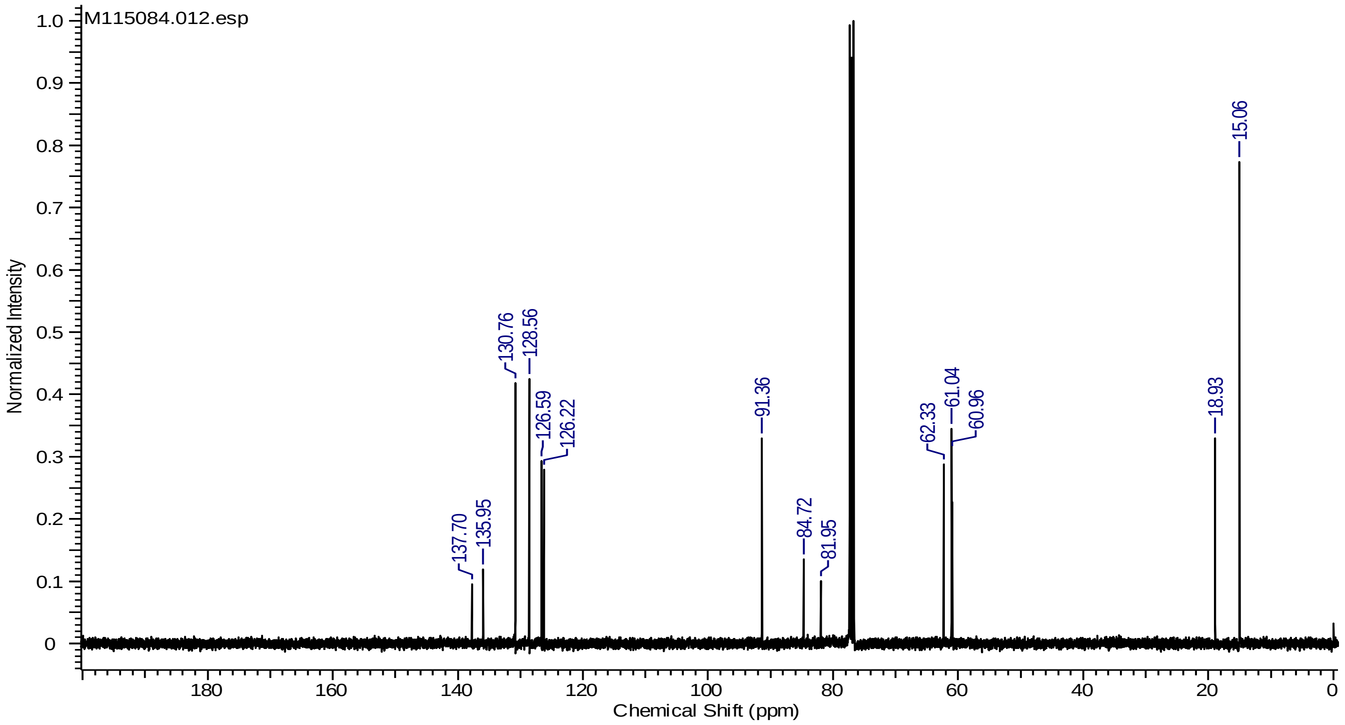

4,4-Diethoxy-1-(furan-2-yl)but-2-yn-1-ol 1l

^{1}H NMR (500 MHz, $CDCl_3$)

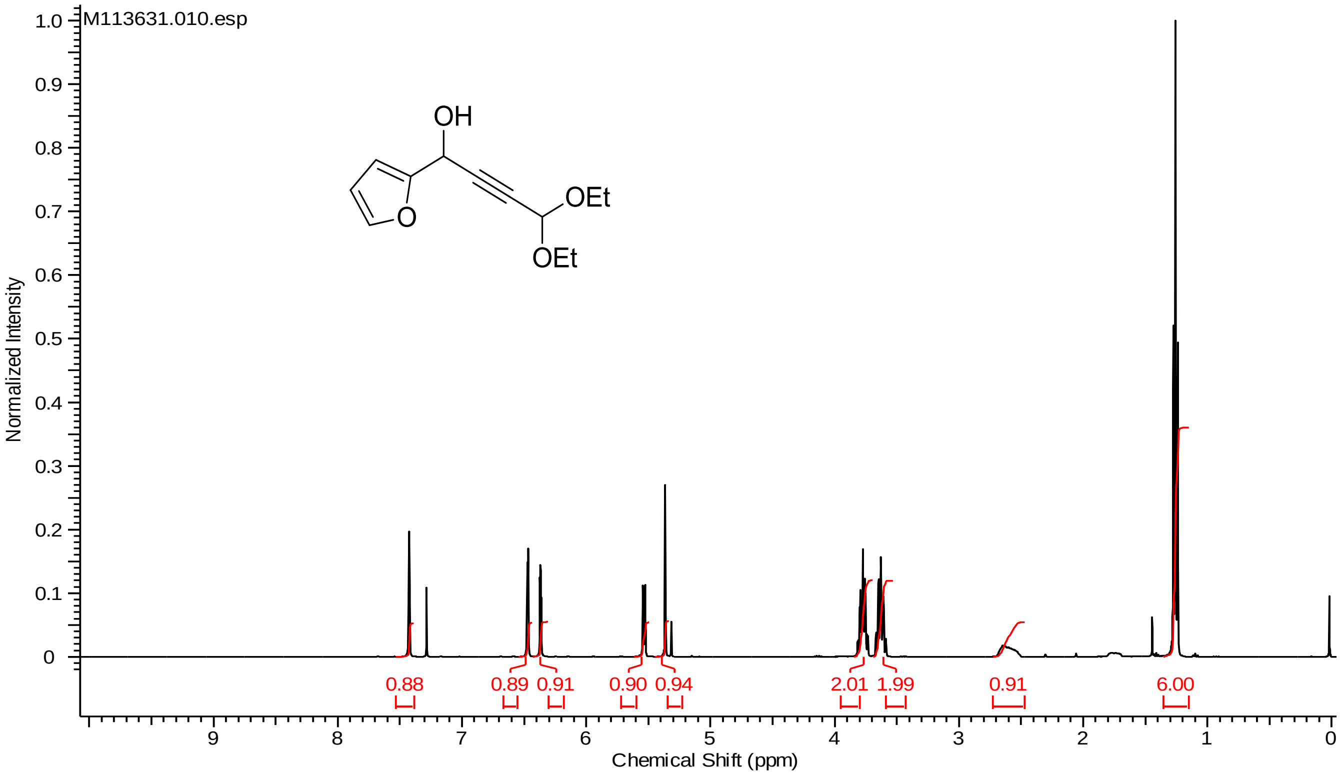

^{13}C NMR (125 MHz, $CDCl_3$)

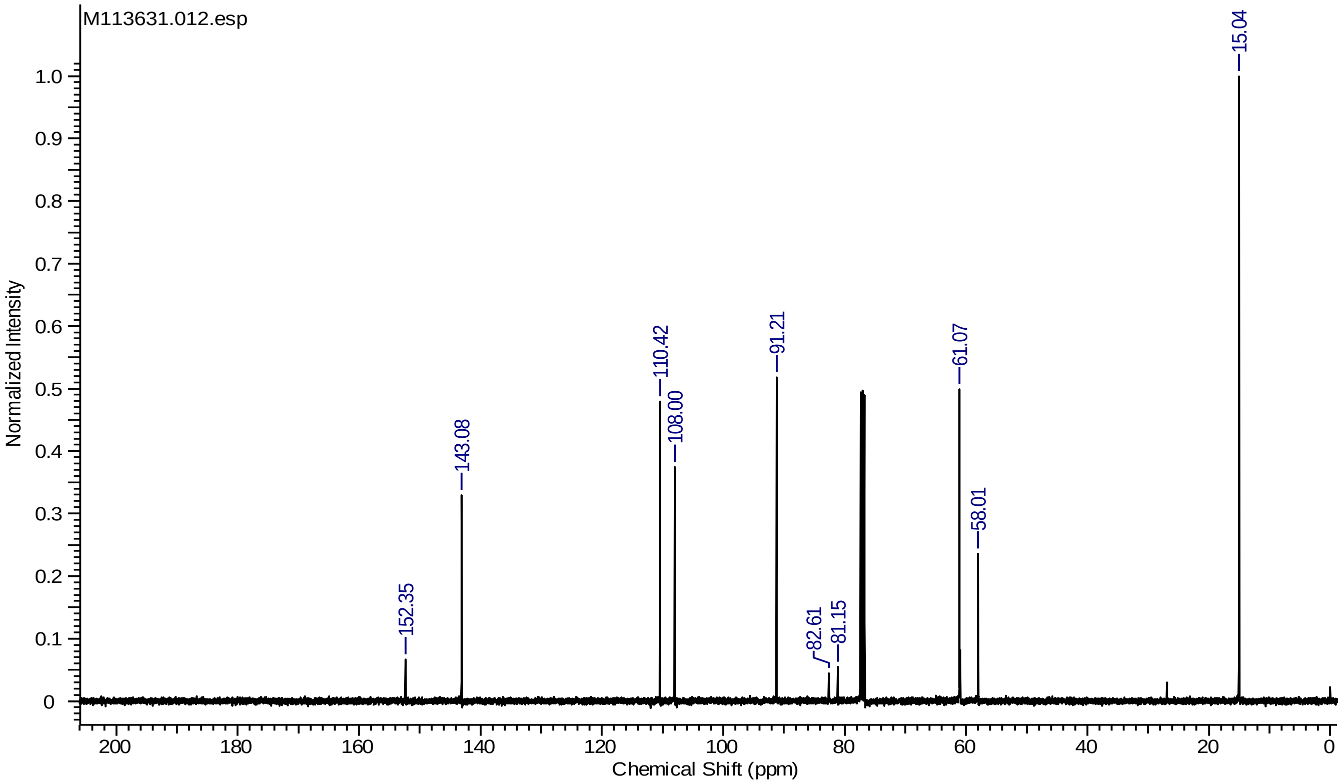

4,4-Diethoxy-1-(thiophen-2-yl)but-2-yn-1-ol 1m

^{1}H NMR (500 MHz, $CDCl_3$)

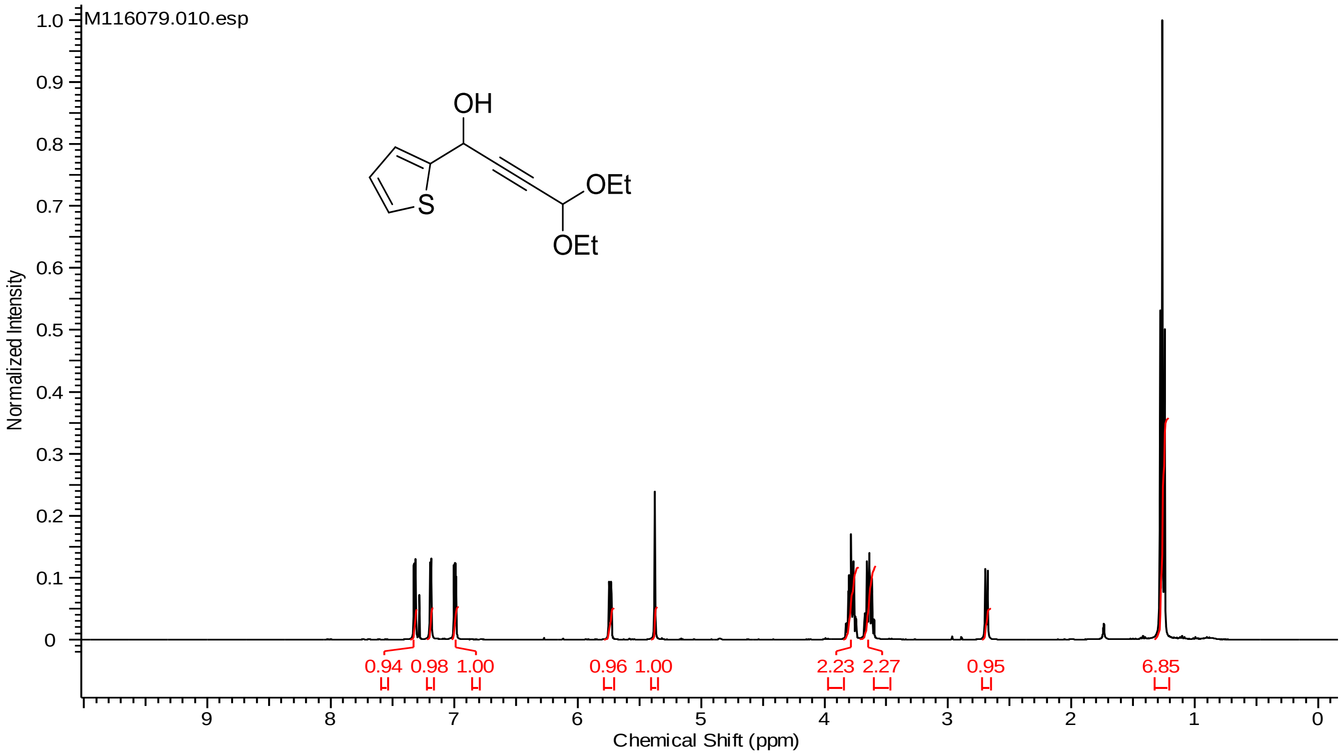

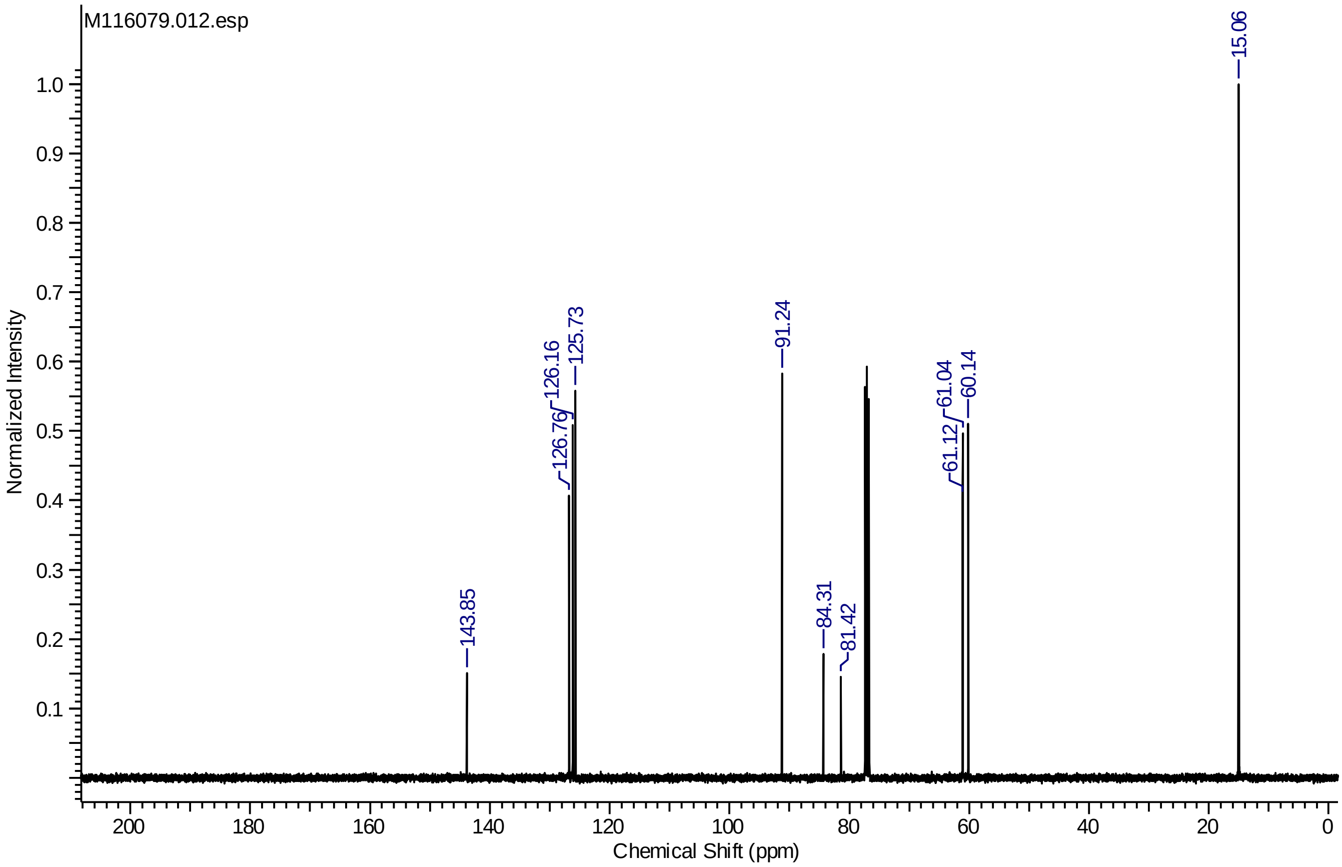

4,4-Diethoxy-1-(pyridin-3-yl)but-2-yn-1-ol 1n

^{1}H NMR (500 MHz, DMSO-d$_6$)

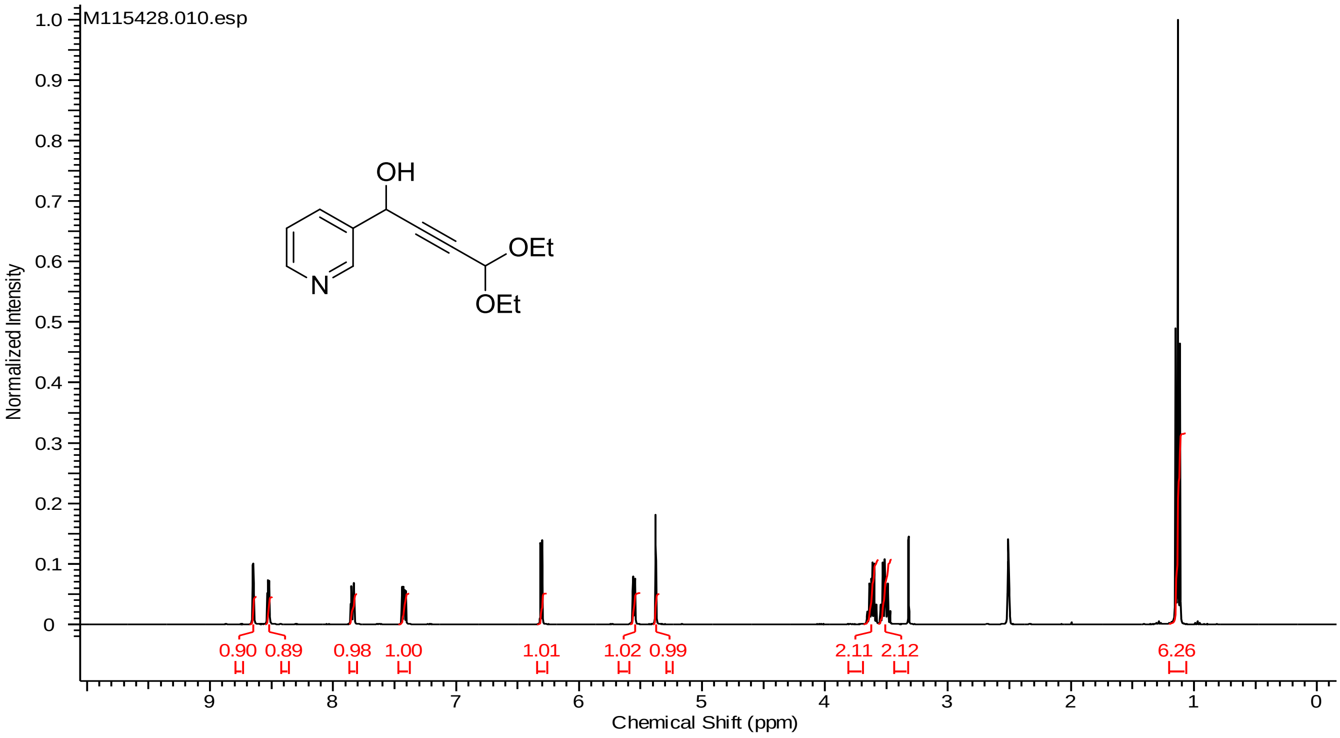

^{13}C NMR (125 MHz, DMSO-d$_6$)

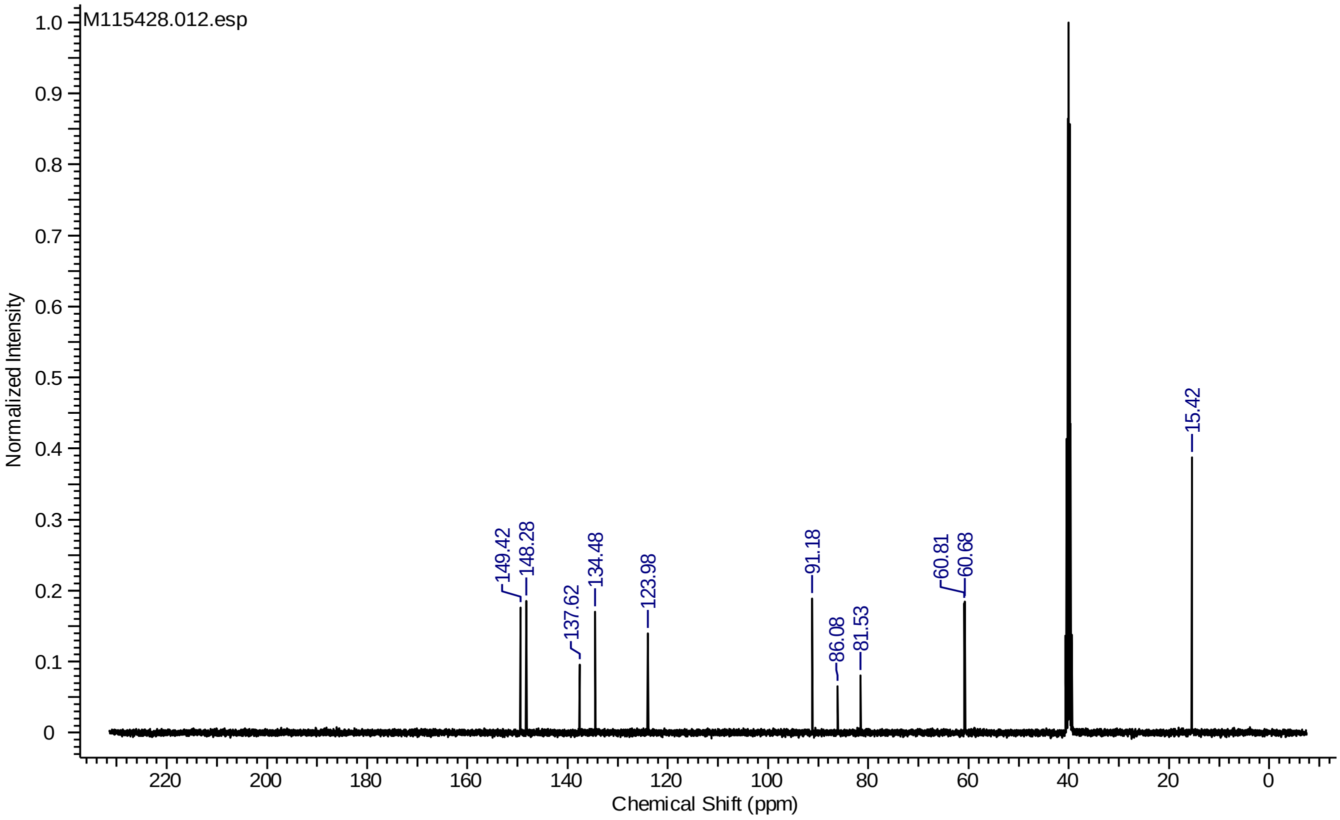

3-Ethoxy-2-phenethylfuran 2a

^{1}H NMR (400 MHz, MeOH-d$_4$)

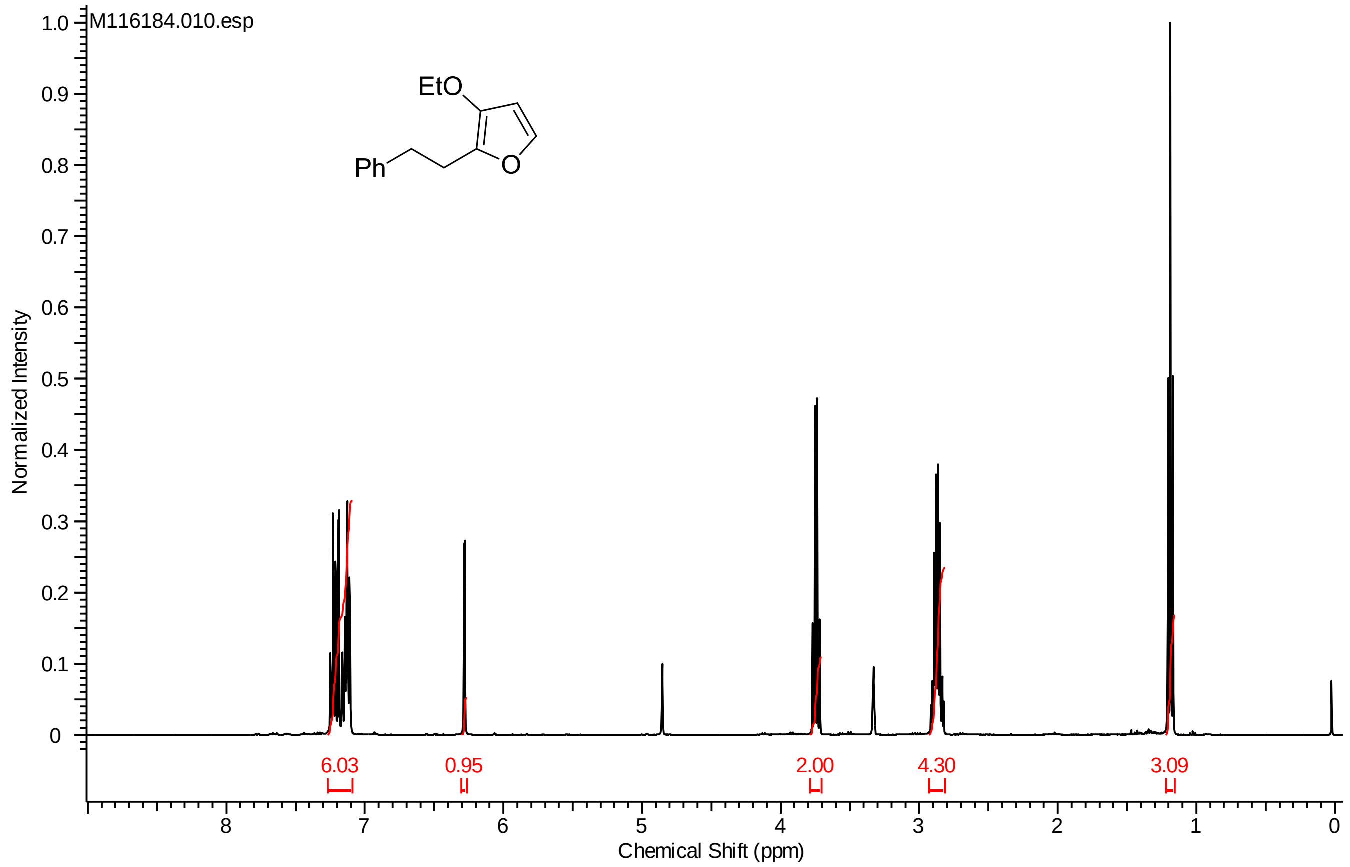

^{13}C NMR (100 MHz, MeOH-d$_4$)

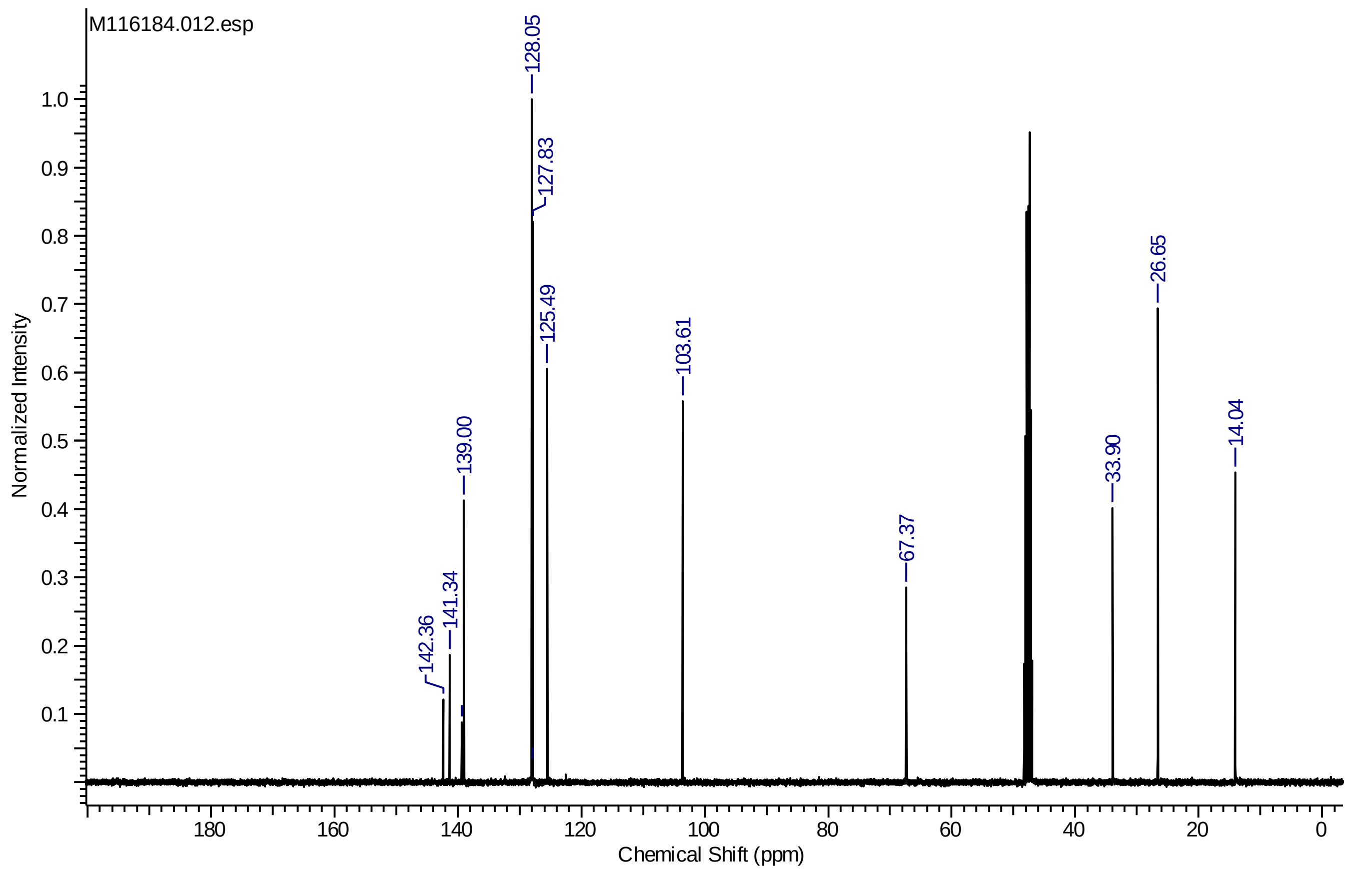

3-Methoxy-2-phenethylfuran 2b

^{1}H NMR (600 MHz, $CDCl_3$)

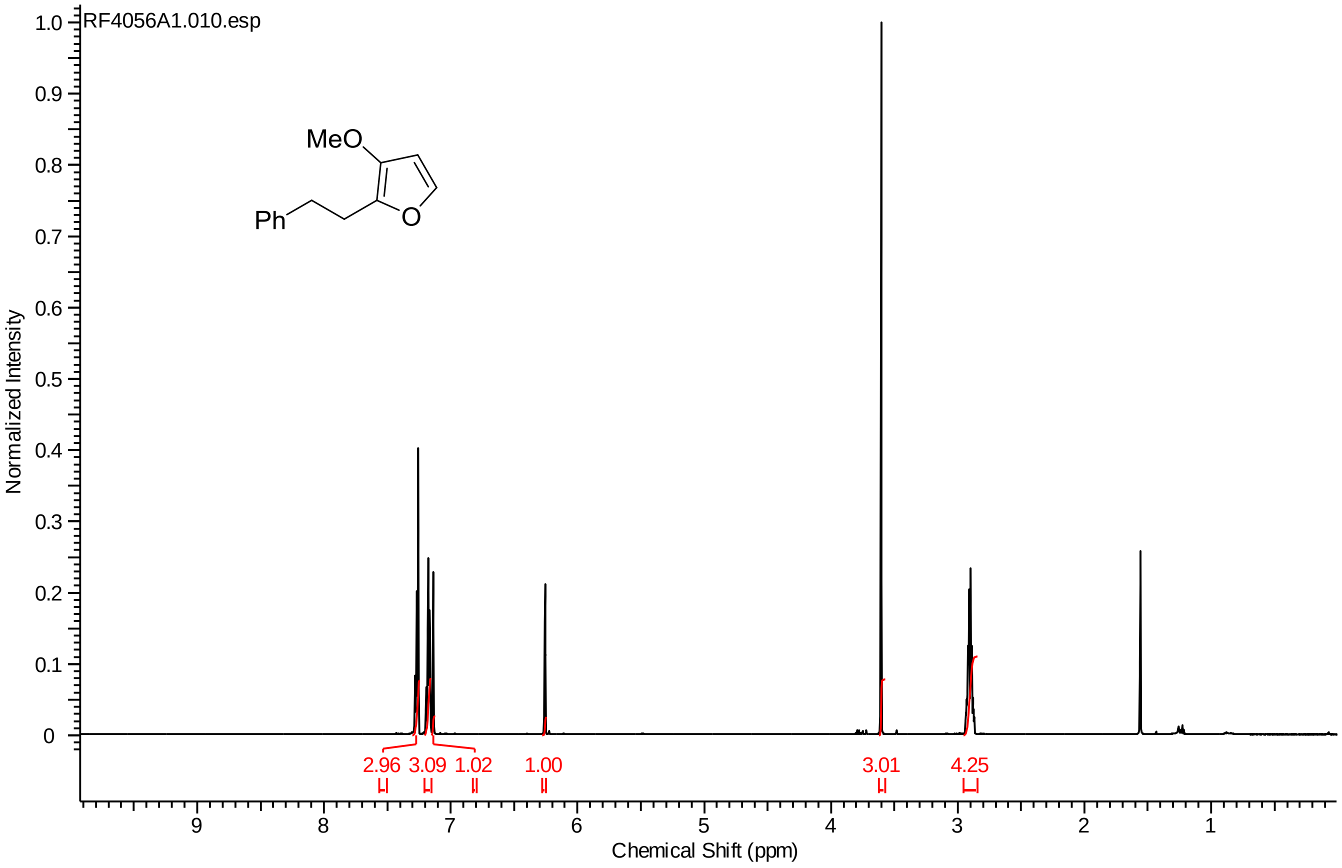

^{13}C NMR (150 MHz, $CDCl_3$)

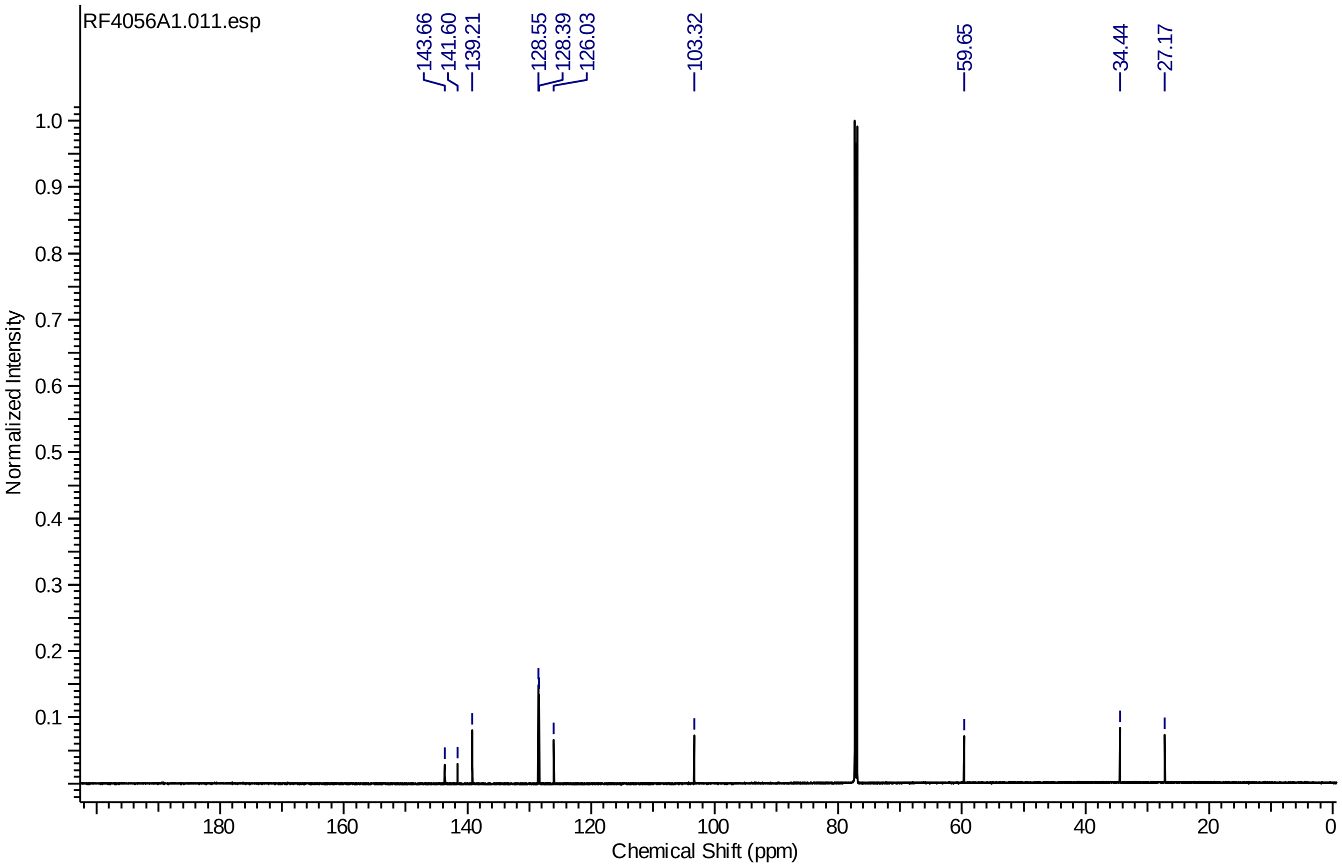

2-Cyclohexyl-3-ethoxyfuran 2c

^{1}H NMR (600 MHz, $CDCl_3$)

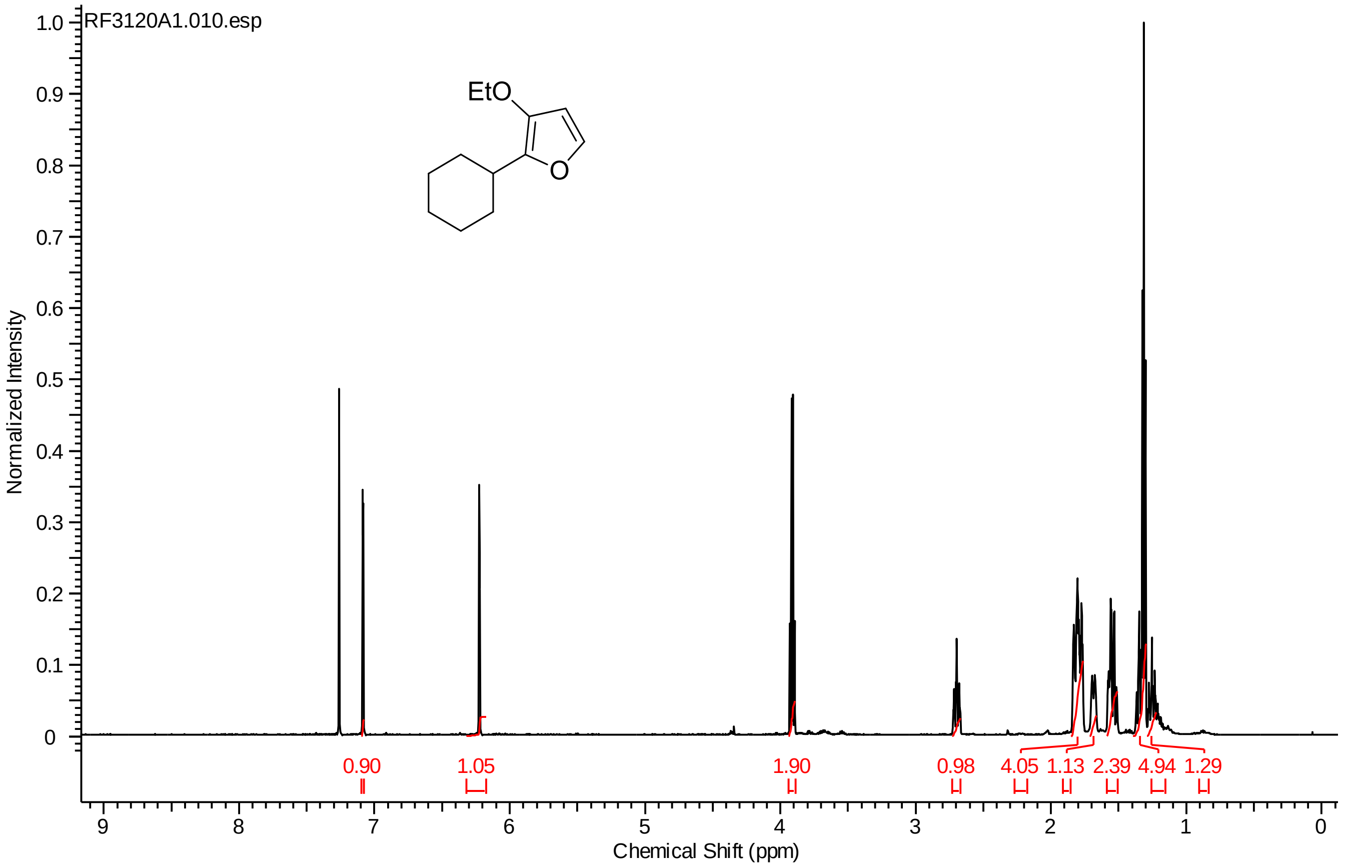

^{13}C NMR (150 MHz, $CDCl_3$)

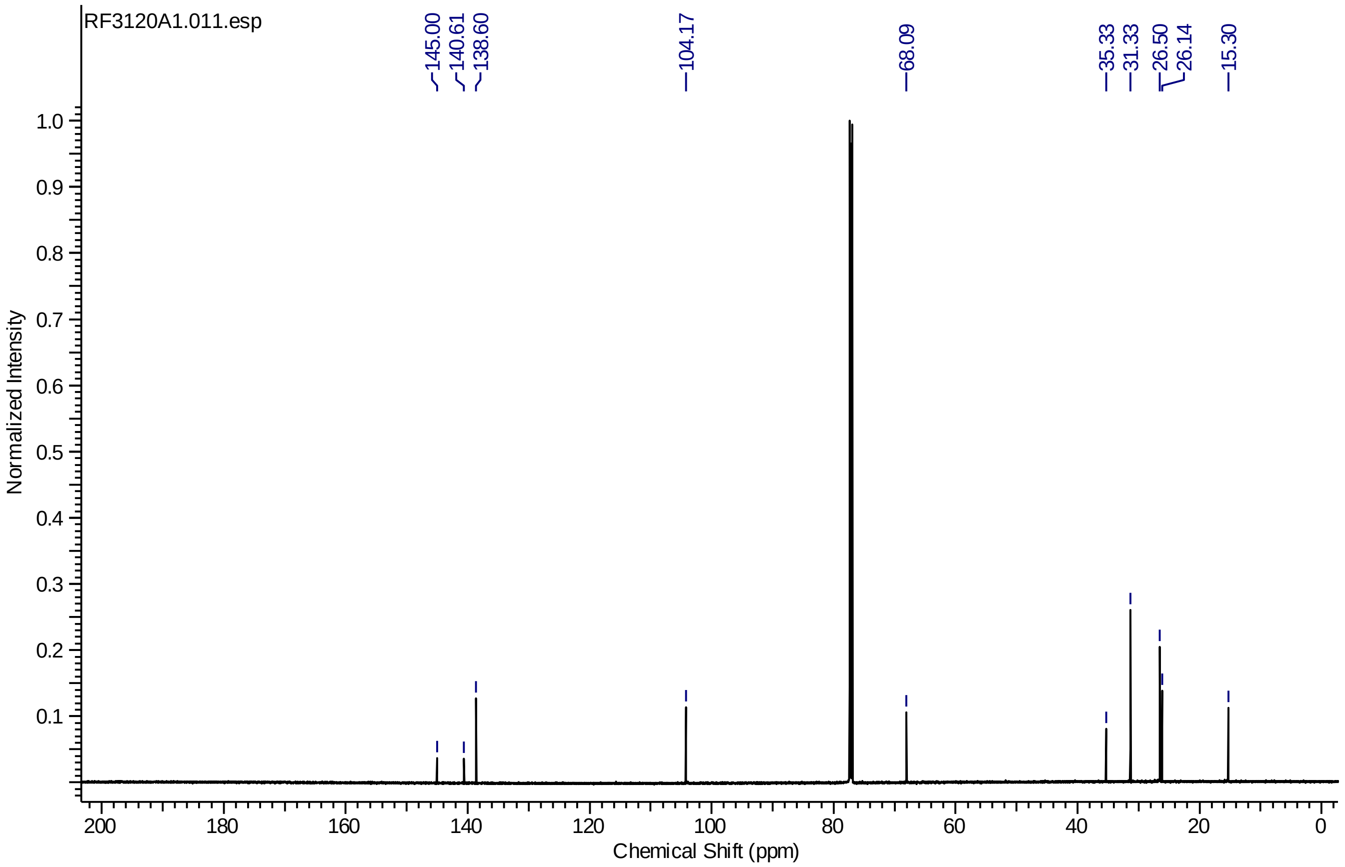

2-Cyclopropyl-3-ethoxyfuran 2d

^{1}H NMR (400 MHz, DMSO-d_6)

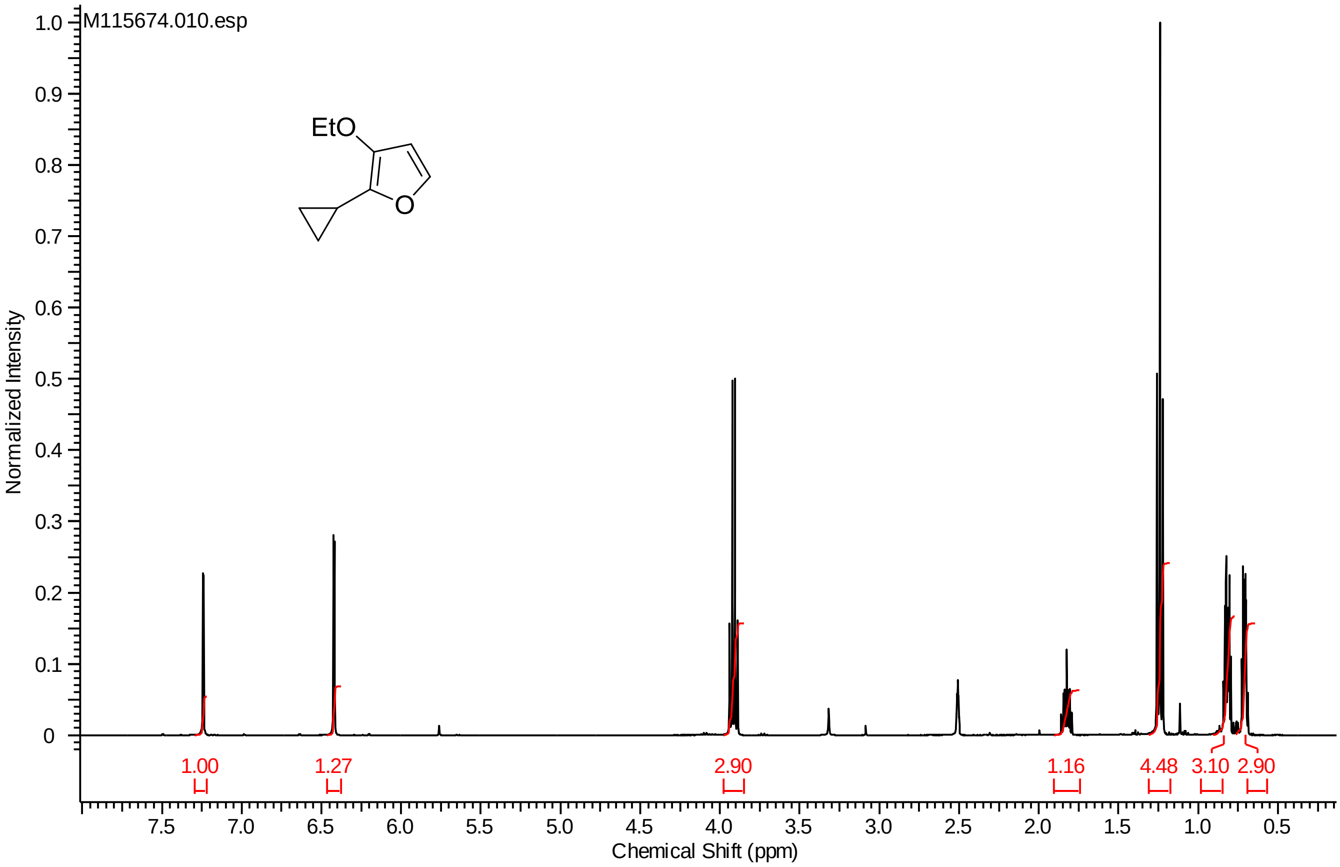

^{13}C NMR (100 MHz, DMSO-d_6)

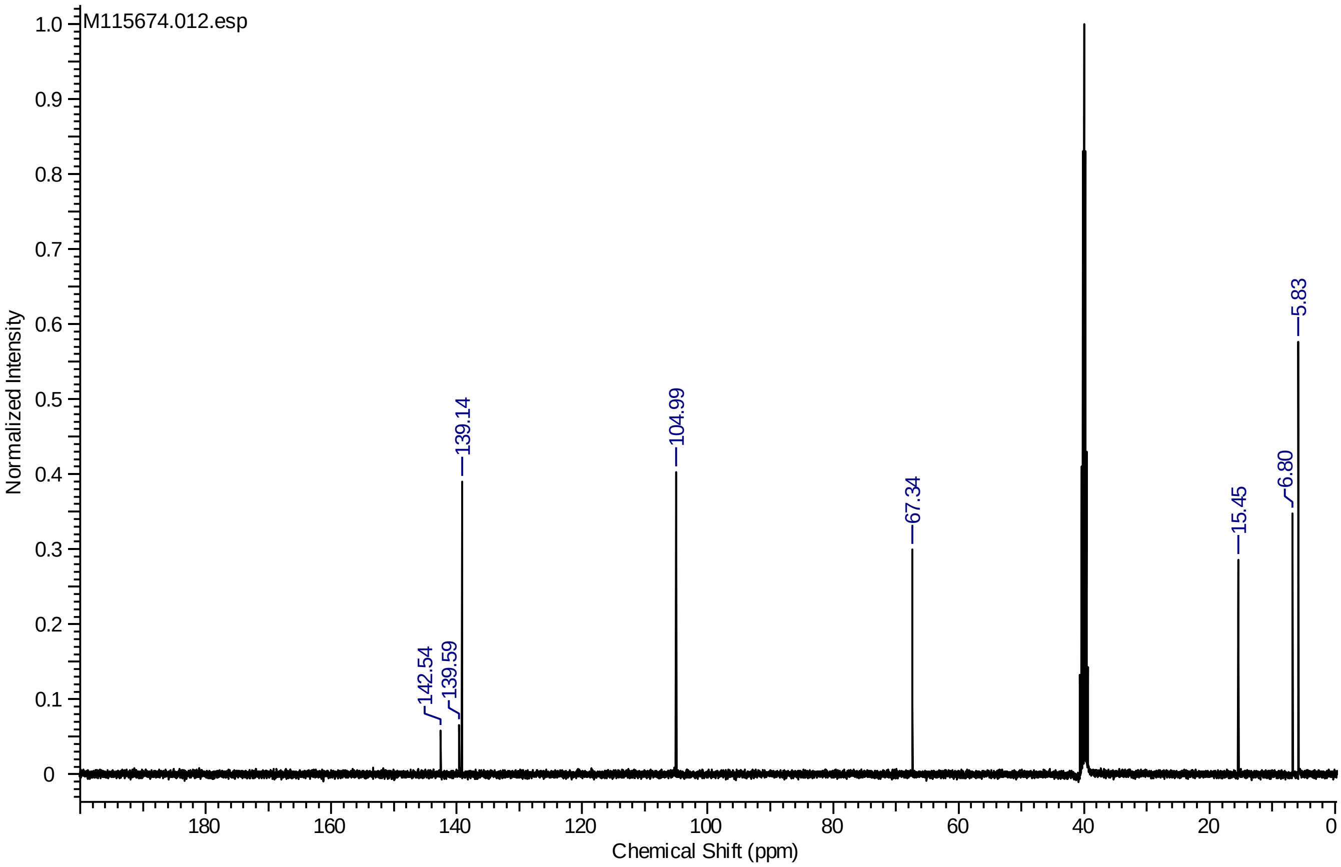

tert-Butyl 4-((3-ethoxyfuran-2-yl)methyl)piperidine-1-carboxylate 2e

^{1}H NMR (400 MHz, MeOH-d$_4$)

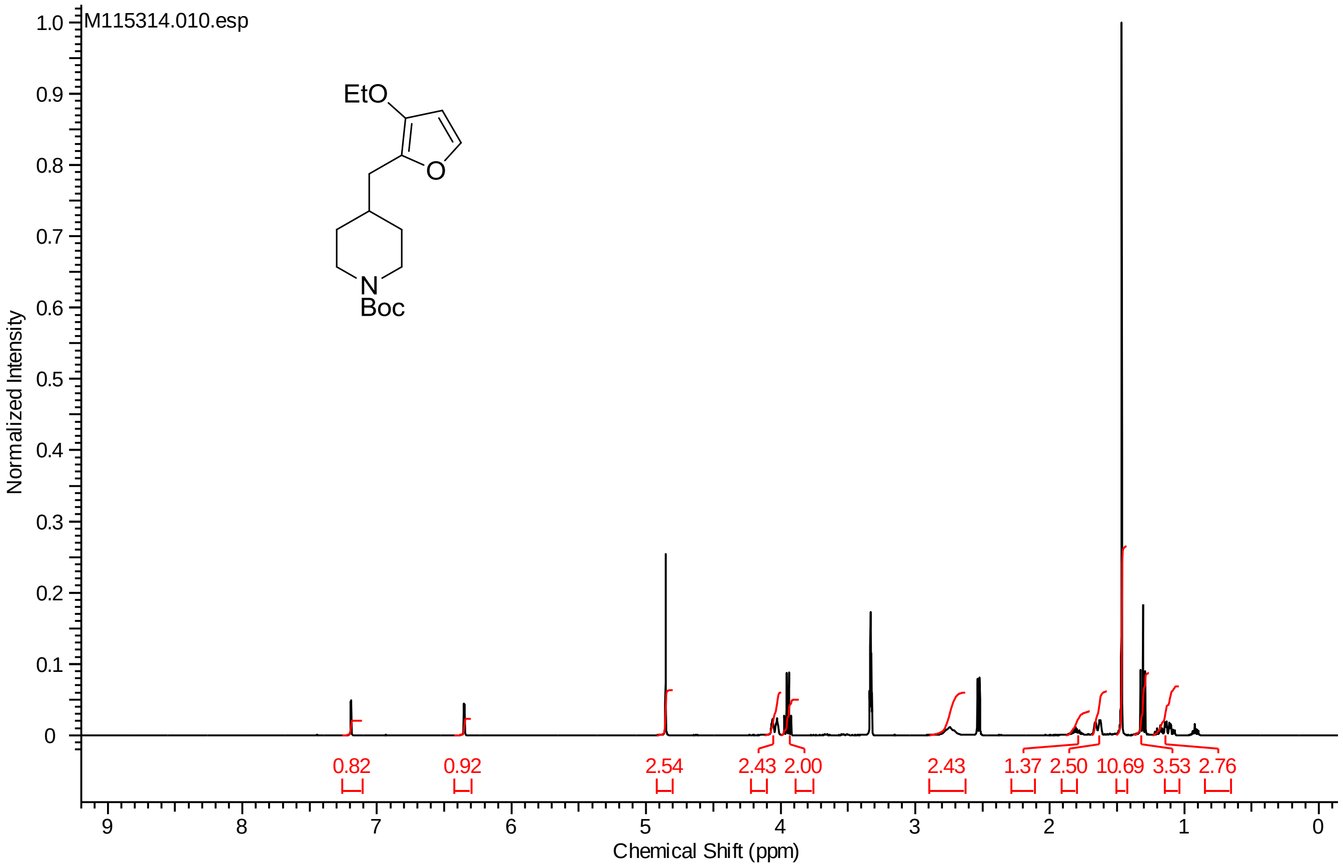

^{13}C NMR (100 MHz, MeOH-d$_4$)

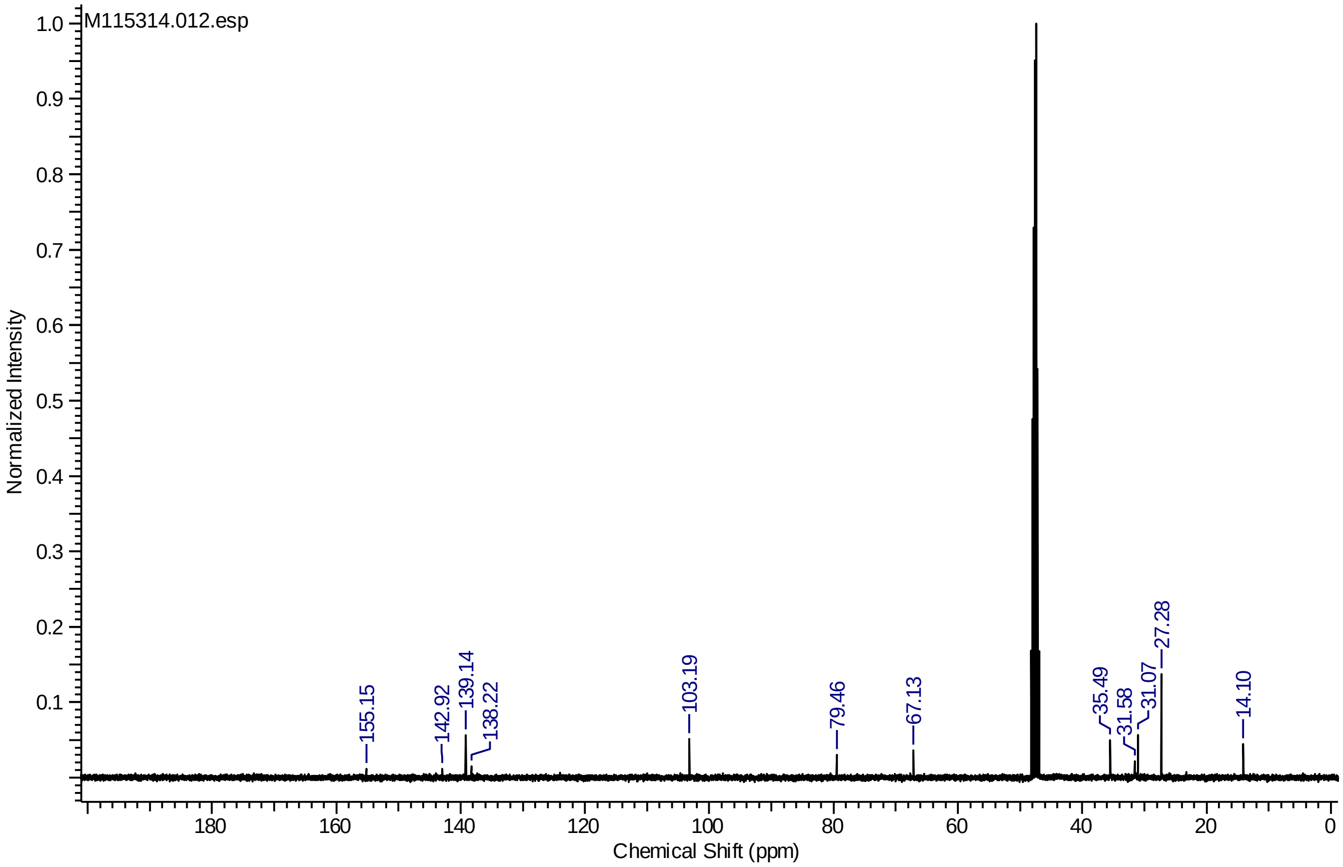

3-Ethoxy-2-phenylfuran 2f

^{1}H NMR (600 MHz, $CDCl_3$)

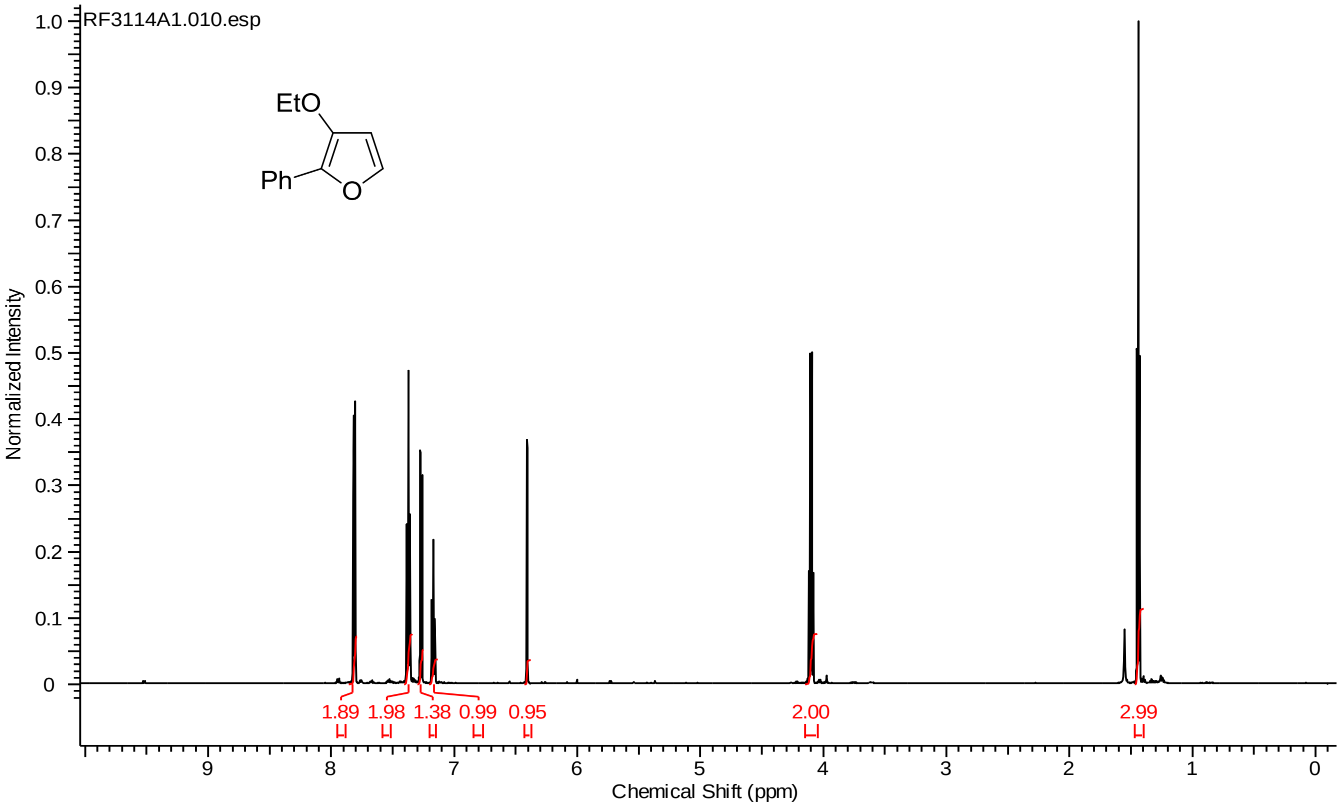

^{13}C NMR (150 MHz, $CDCl_3$)

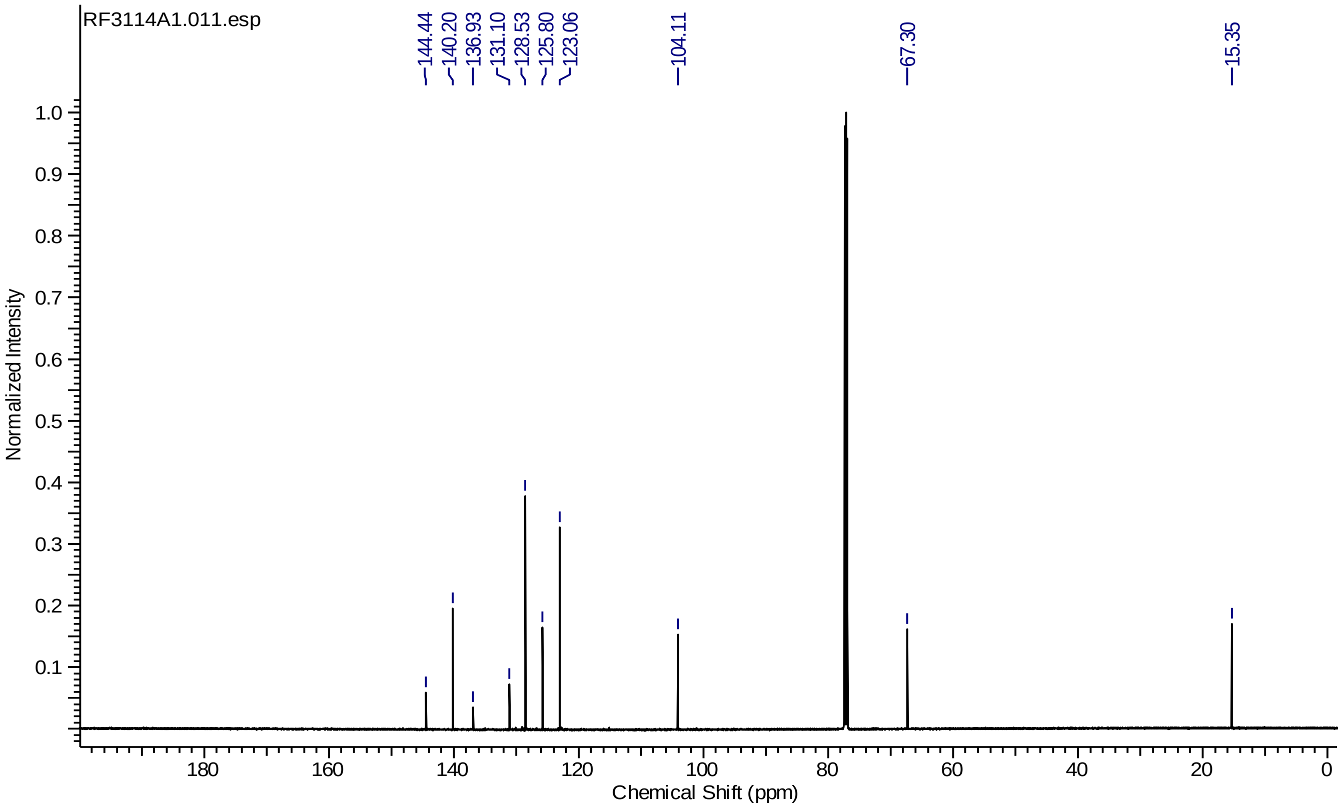

3-Ethoxy-2-(4-(trifluoromethyl)phenyl)furan 2g

^{1}H NMR (600 MHz, MeOH-d$_4$)

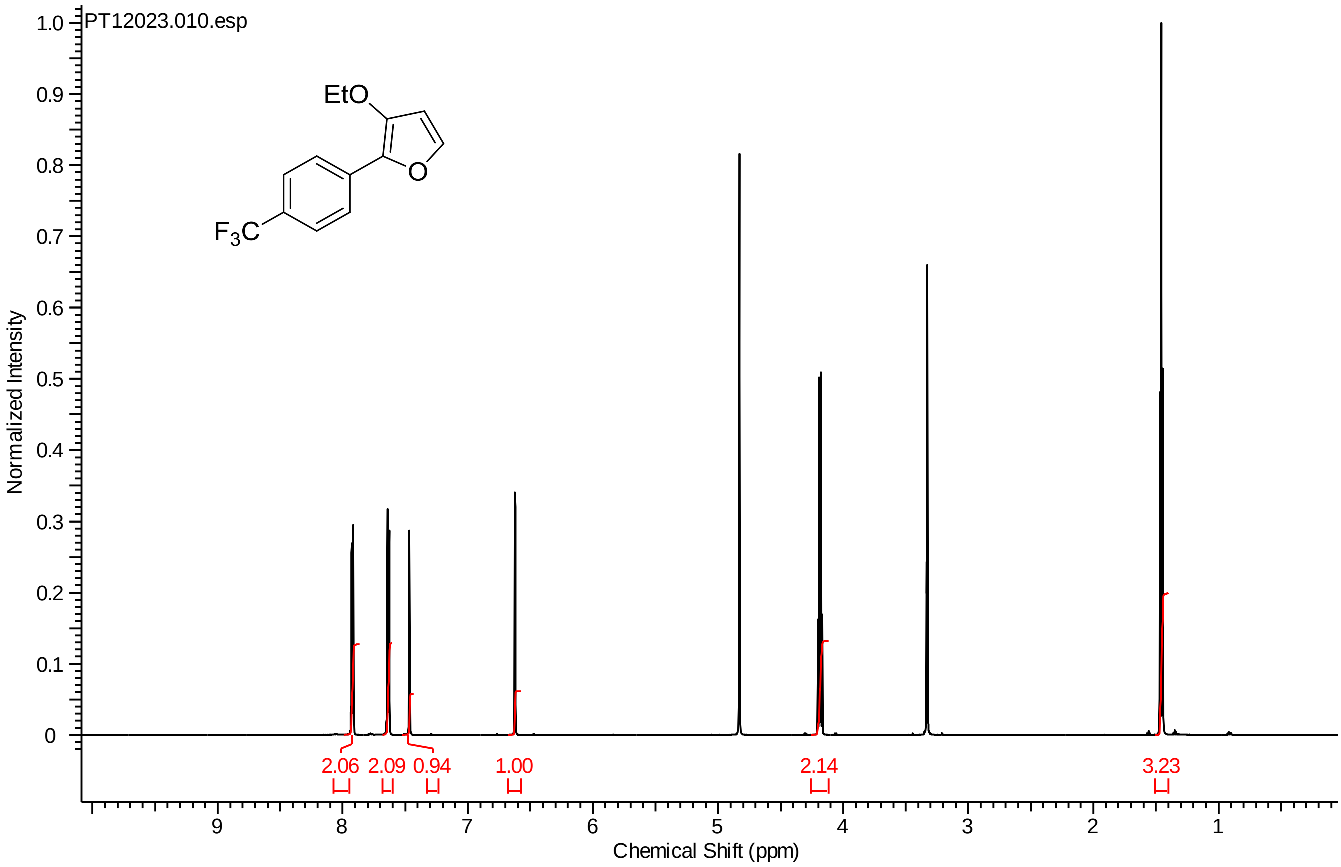

^{13}C NMR (150 MHz, MeOH-d$_4$)

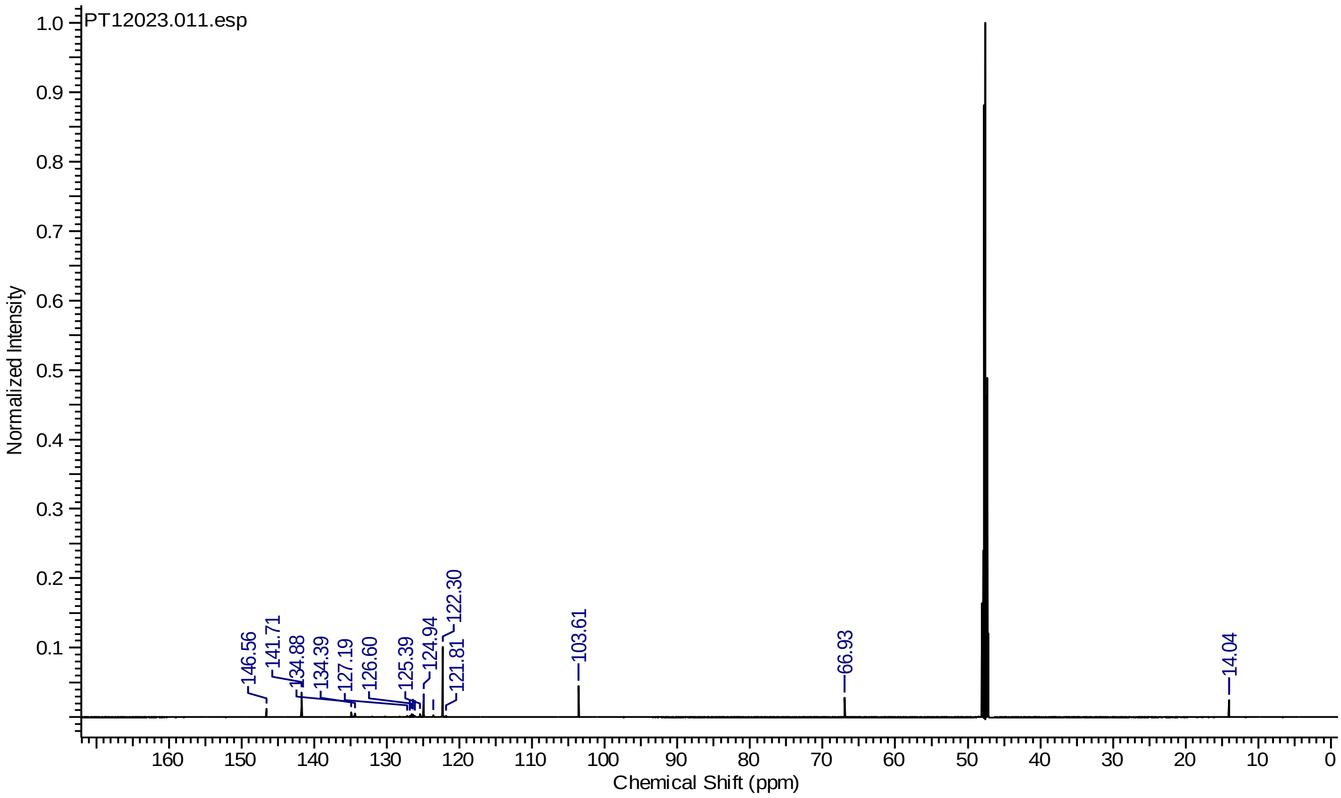

2-(4-Bromophenyl)-3-ethoxyfuran 2h

^{1}H NMR (400 MHz, MeOH-d$_4$)

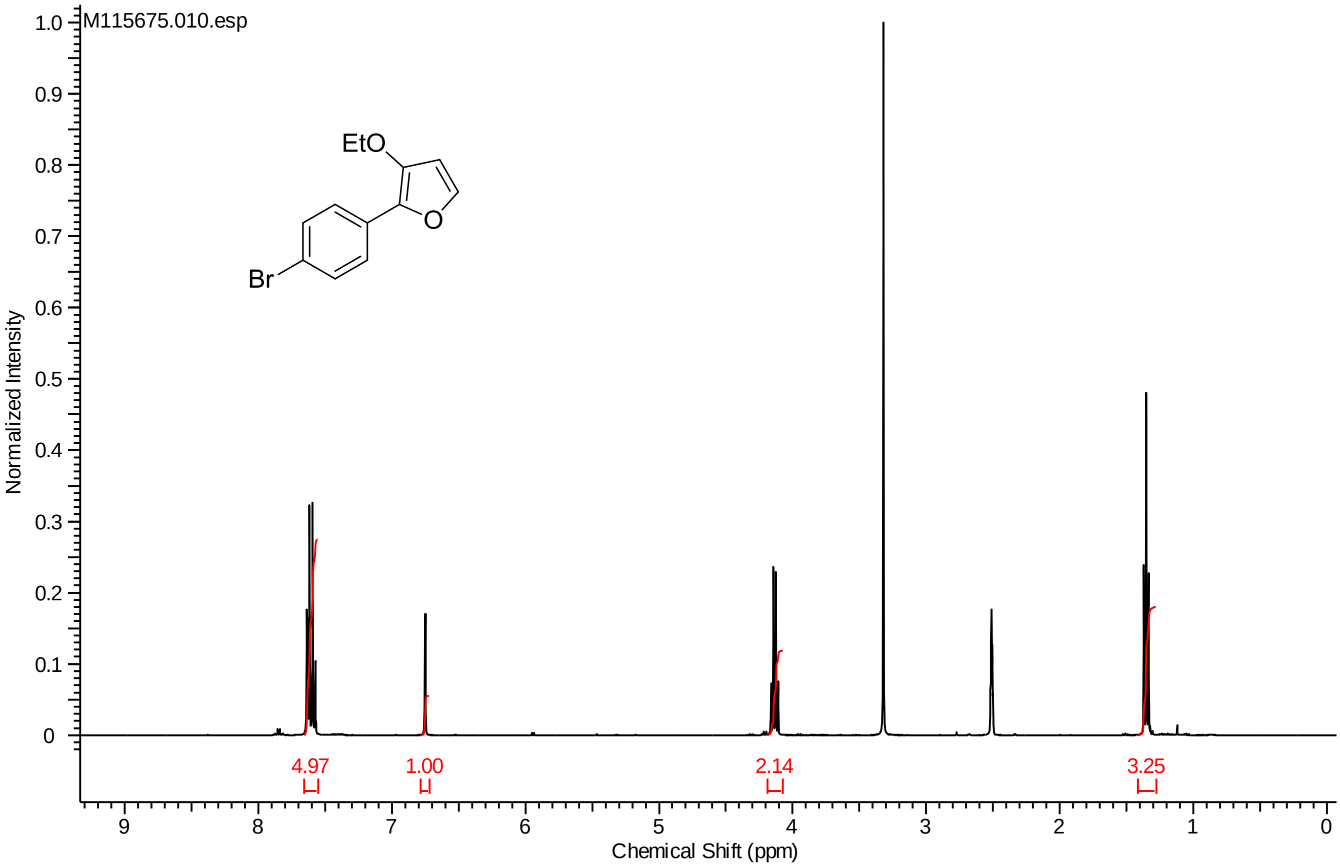

^{13}C NMR (100 MHz, MeOH-d$_4$)

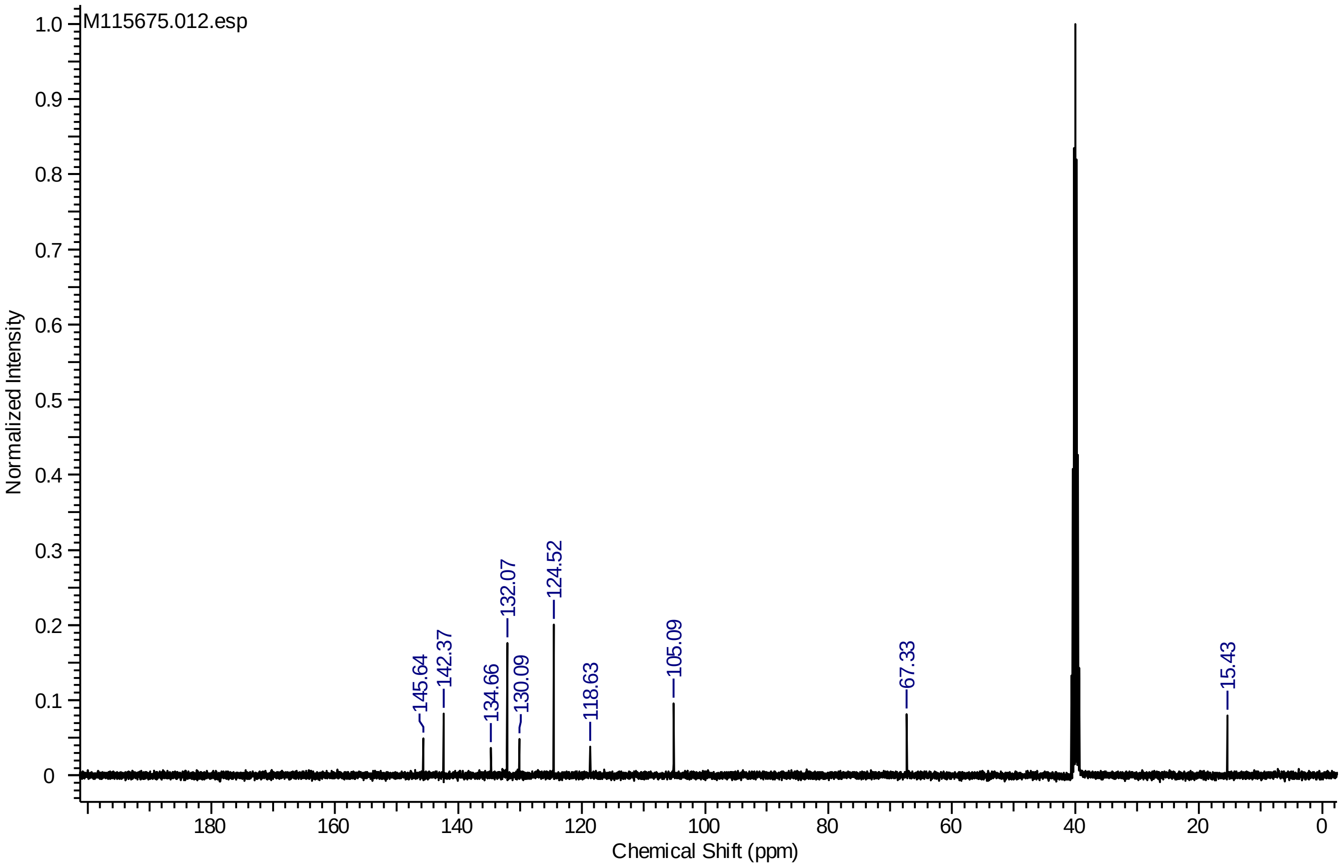

3-Ethoxy-2-(4-methoxyphenyl)furan 2i

^{1}H NMR (400 MHz, MeOH-d_4)

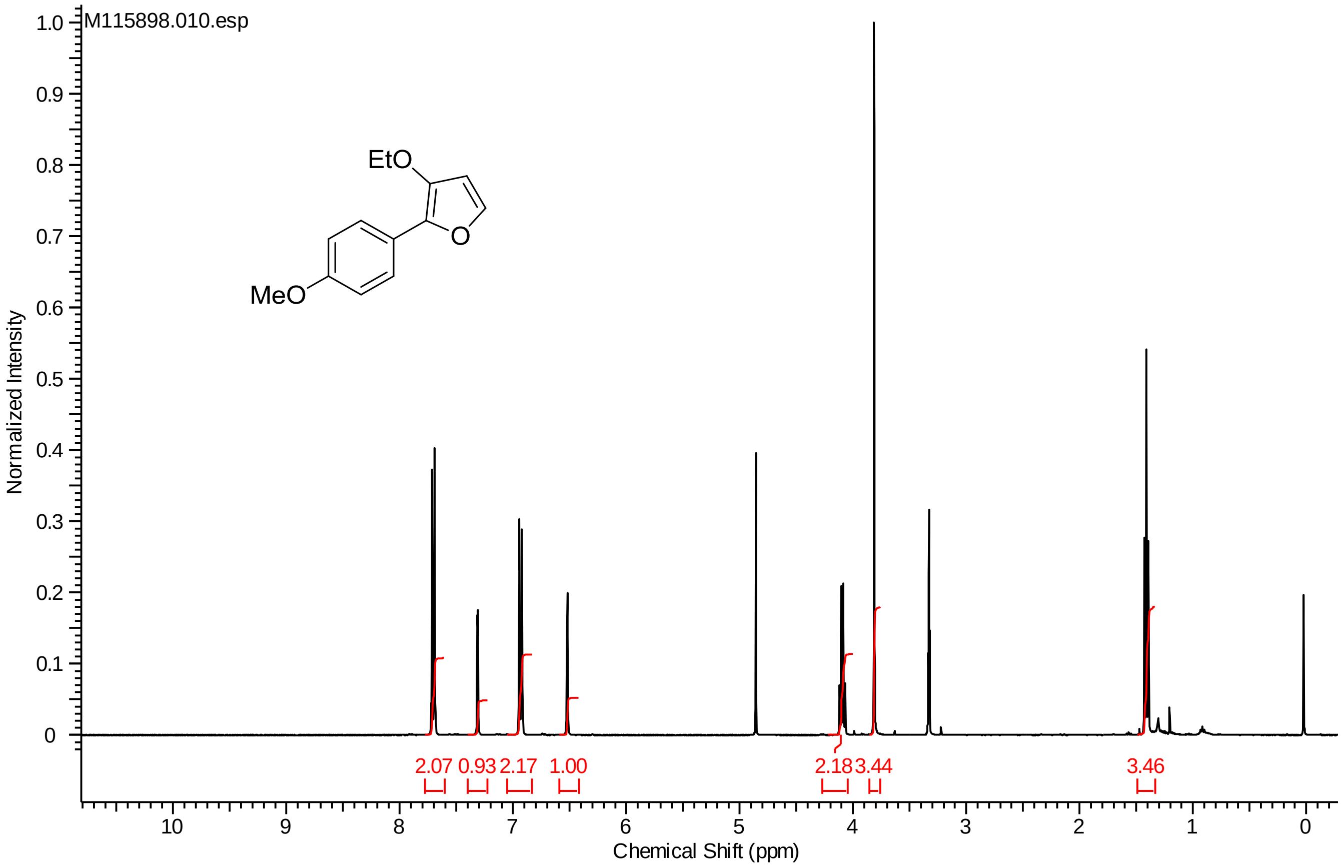

^{13}C NMR (100 MHz, MeOH-d_4)

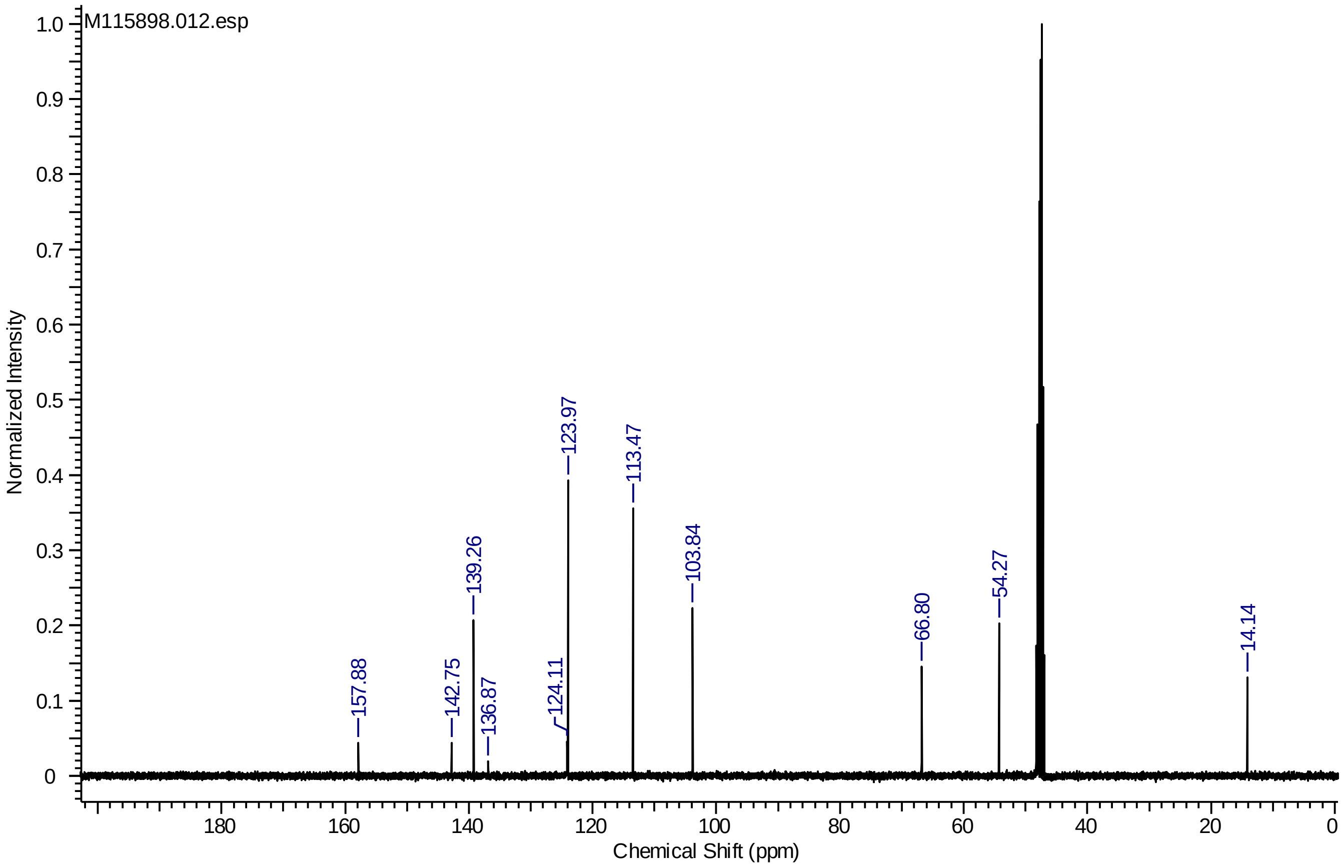

Methyl 3-(3-ethoxyfuran-2-yl)benzoate 3j

^{1}H NMR (400 MHz, MeOH-d$_4$)

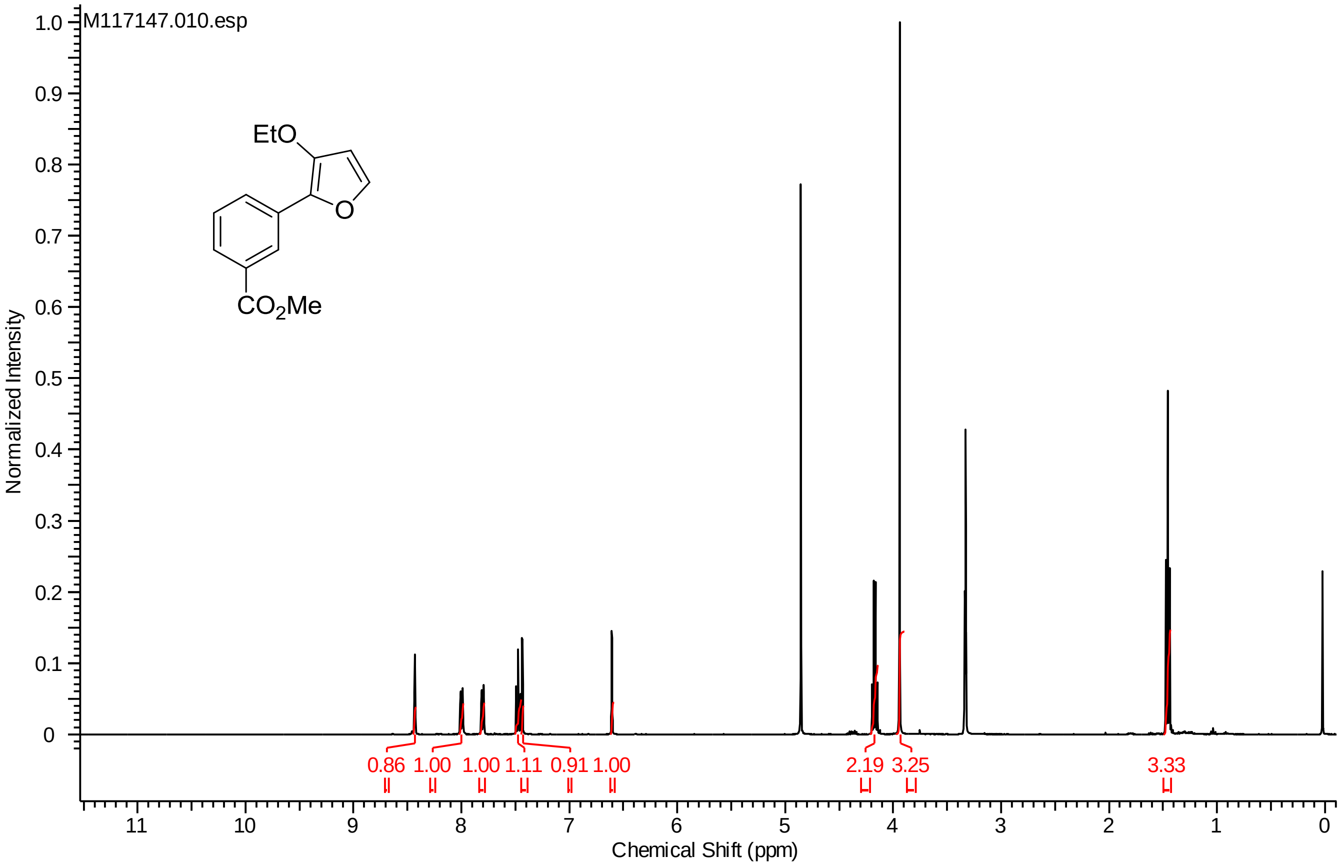

^{1}H NMR (100 MHz, MeOH-d$_4$)

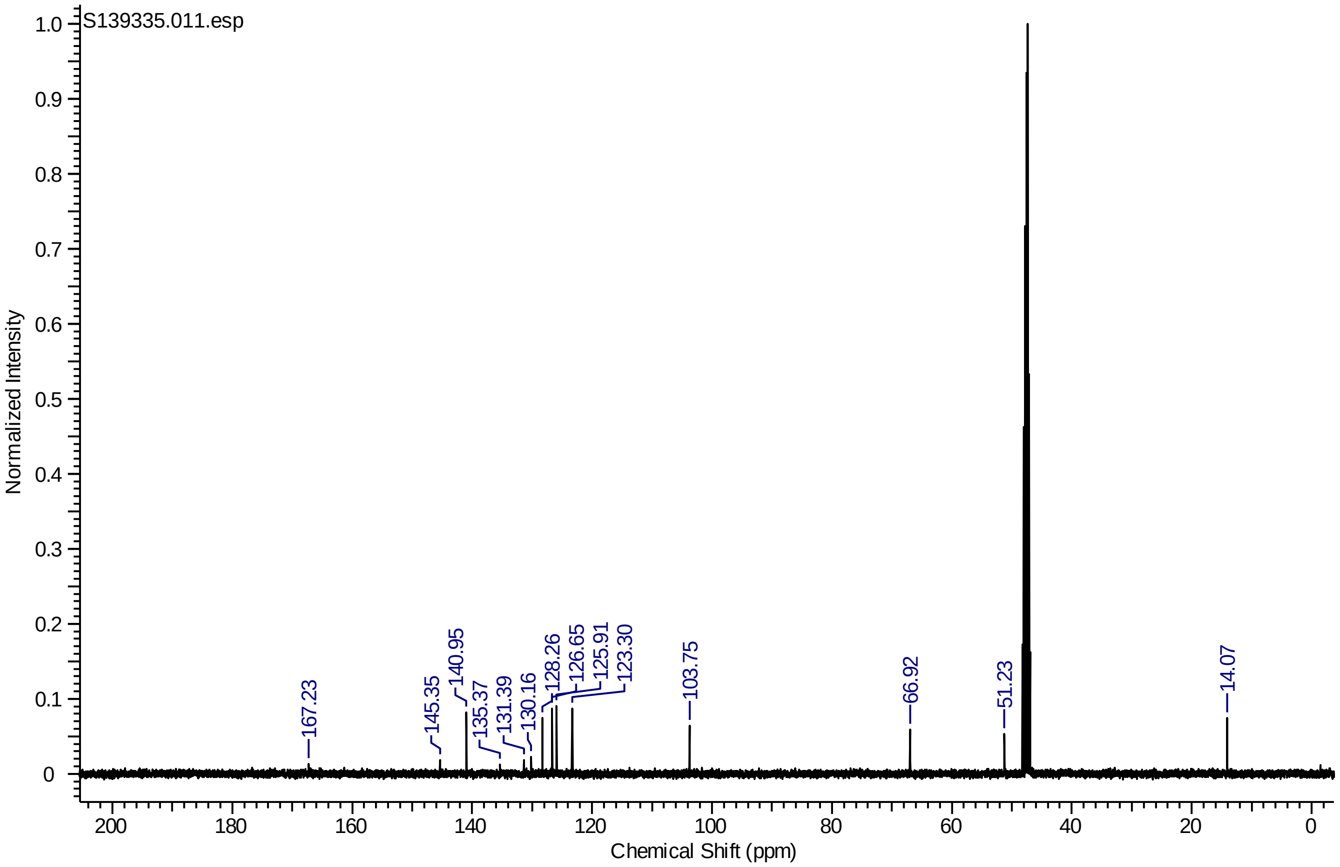

3-Ethoxy-2-(*o*-tolyl)furan 2k

^{1}H NMR (400 MHz, MeOH-d$_4$)

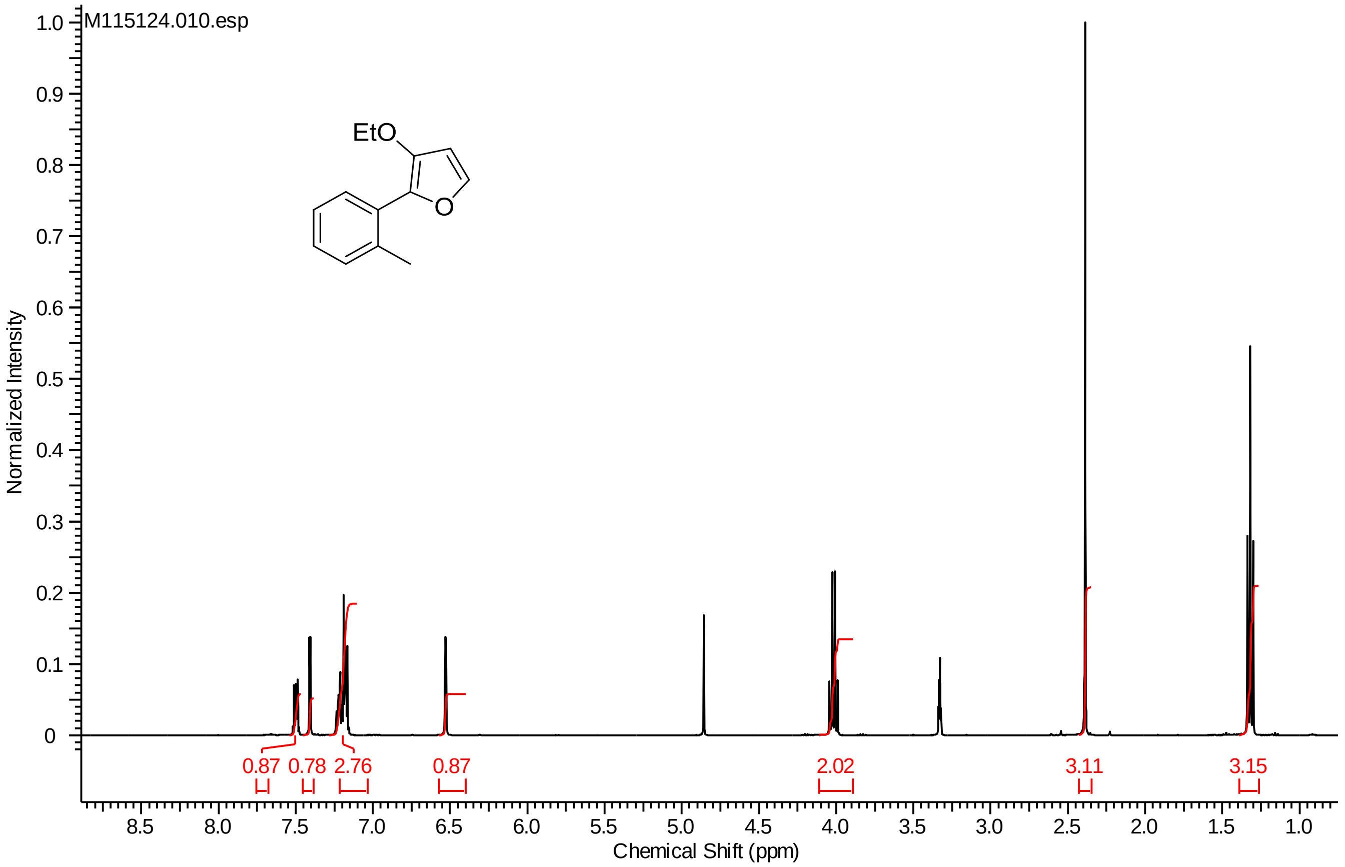

^{13}C NMR (100 MHz, MeOH-d$_4$)

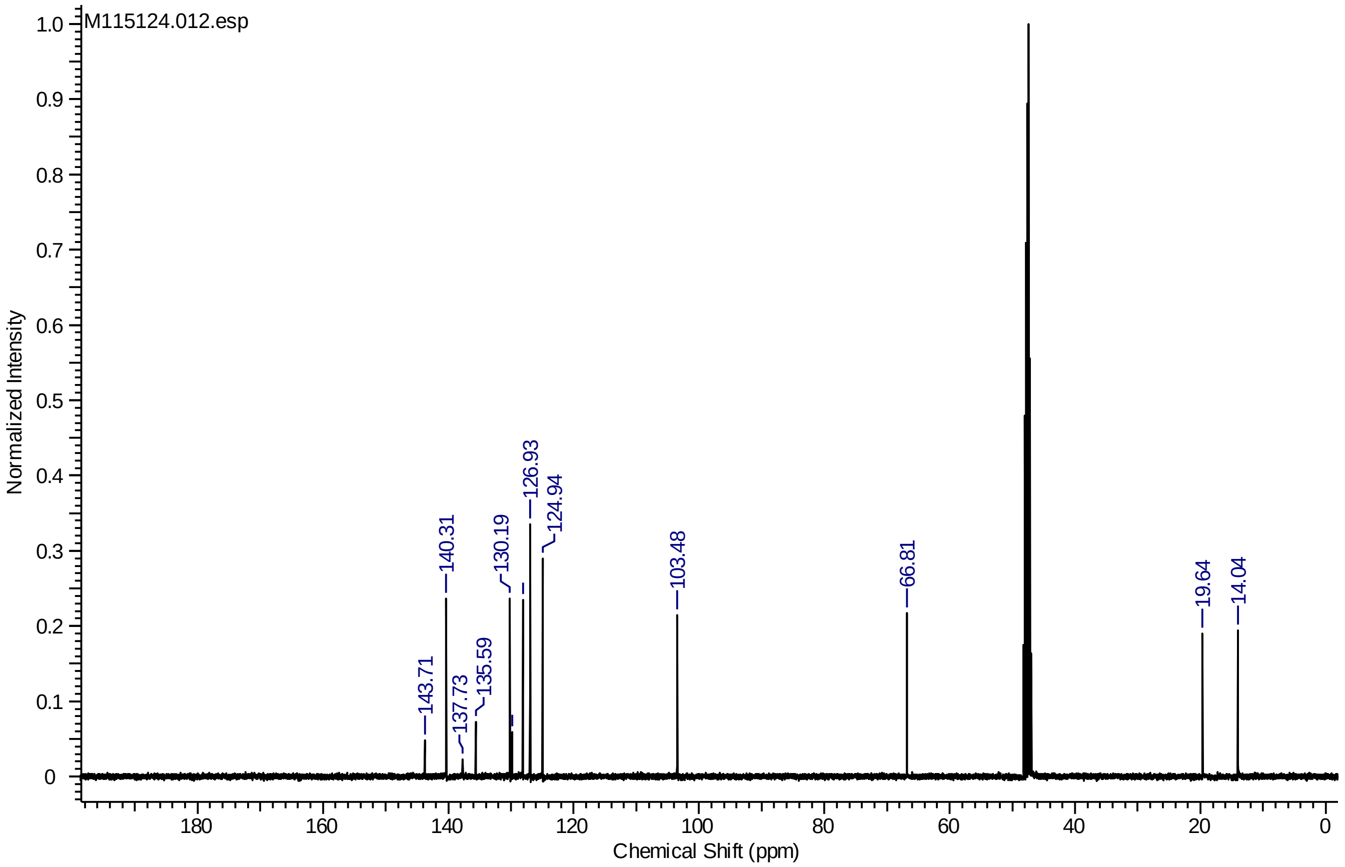

3-Ethoxy-2,2'-bifuran 2l

^{1}H NMR (400 MHz, MeOH-d_4)

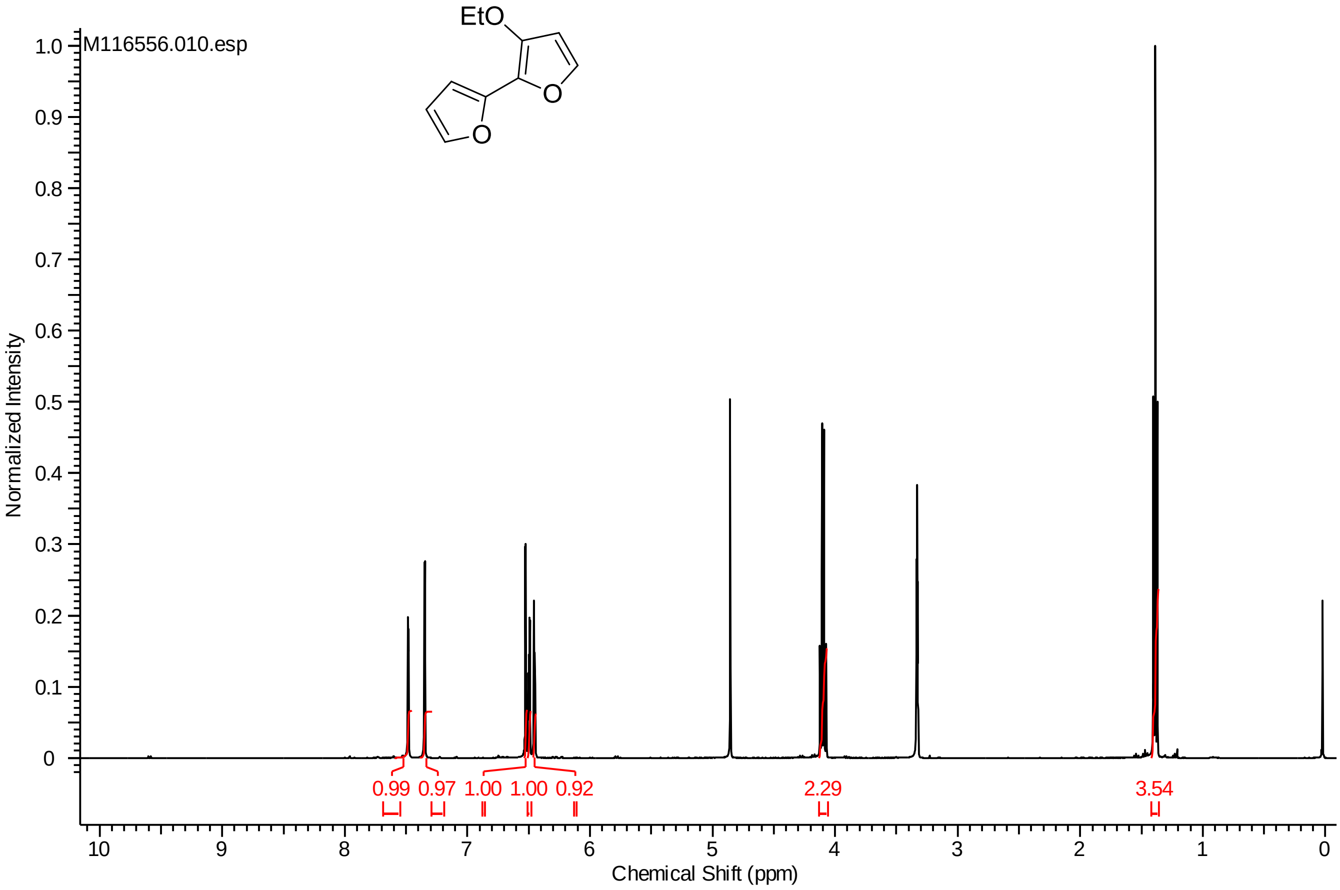

^{13}C NMR (100 MHz, MeOH-d_4)

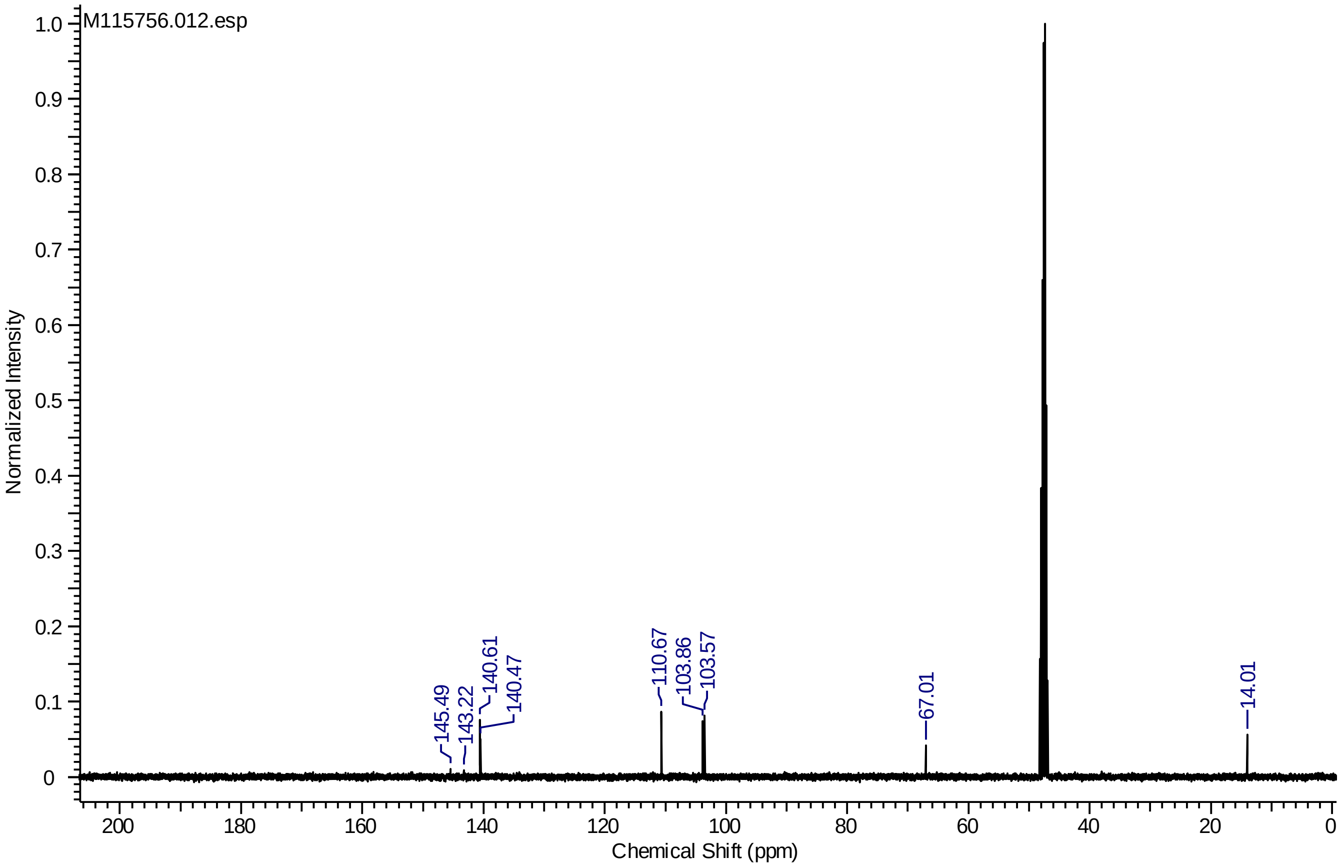

3-Ethoxy-2-(thiophen-2-yl)furan 2m

^{1}H NMR (400 MHz, MeOH-d_4)

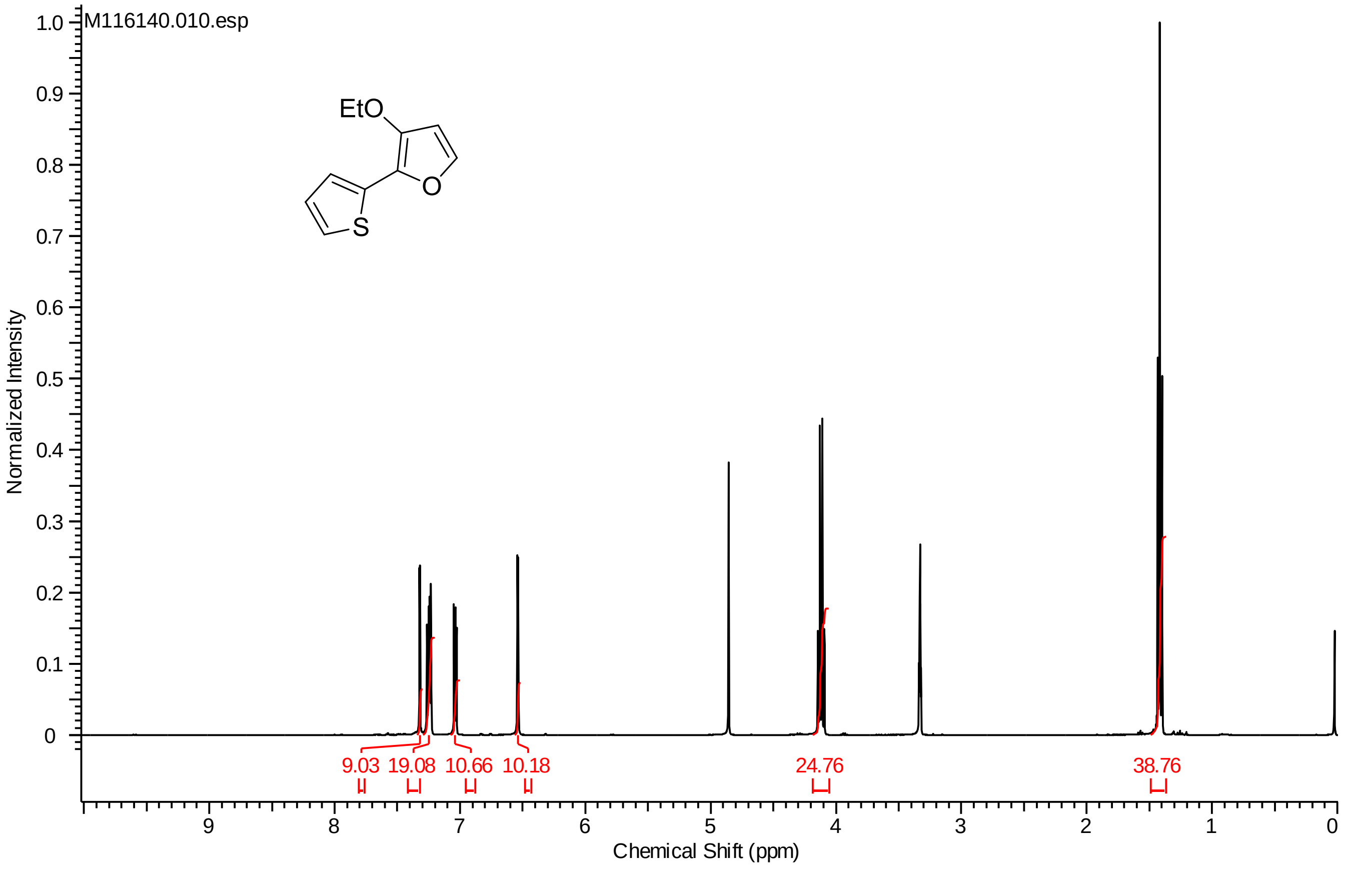

^{13}C NMR (100 MHz, MeOH-d_4)

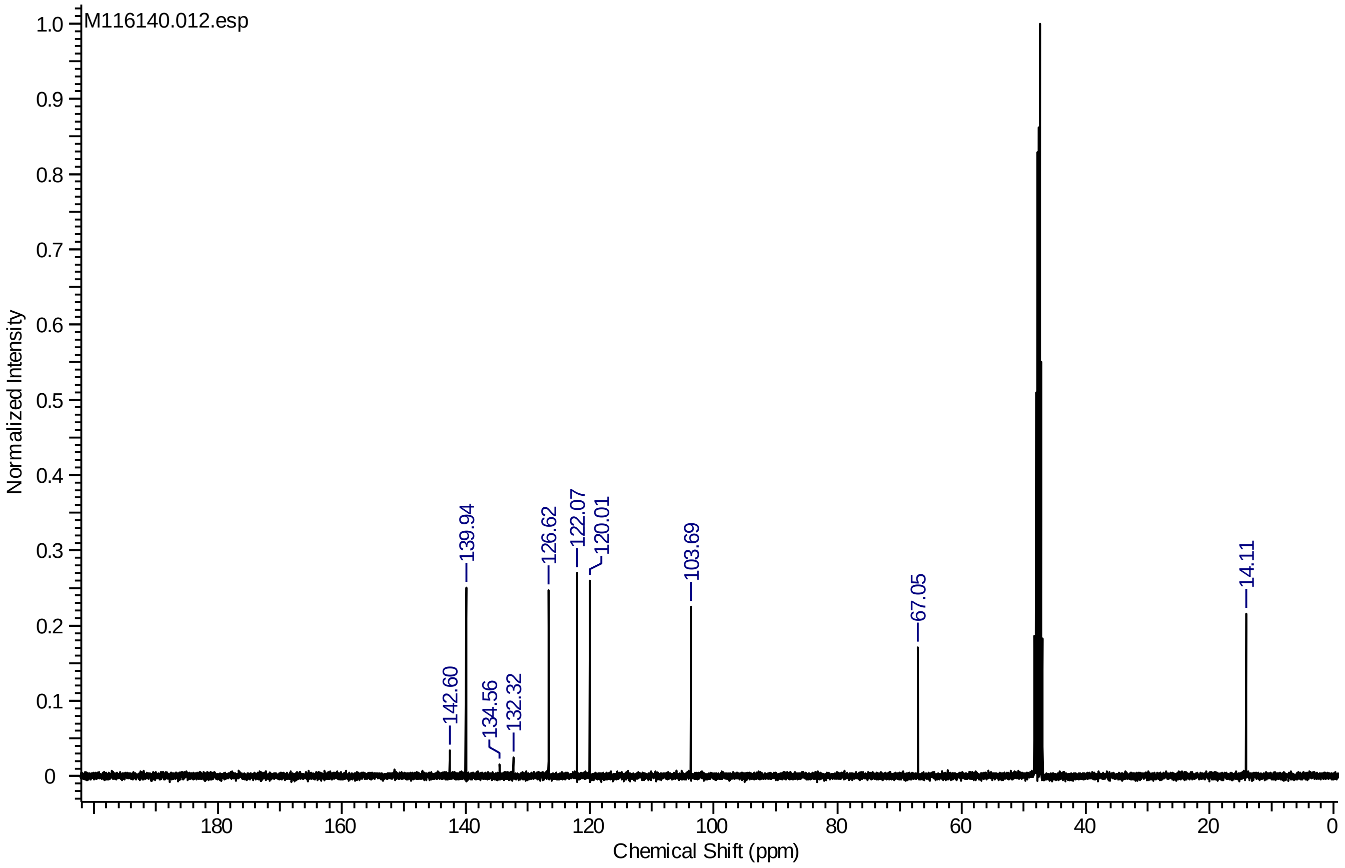

3-(3-Ethoxyfuran-2-yl)pyridine 2n

^{1}H NMR (400 MHz, MeOH-d_4)

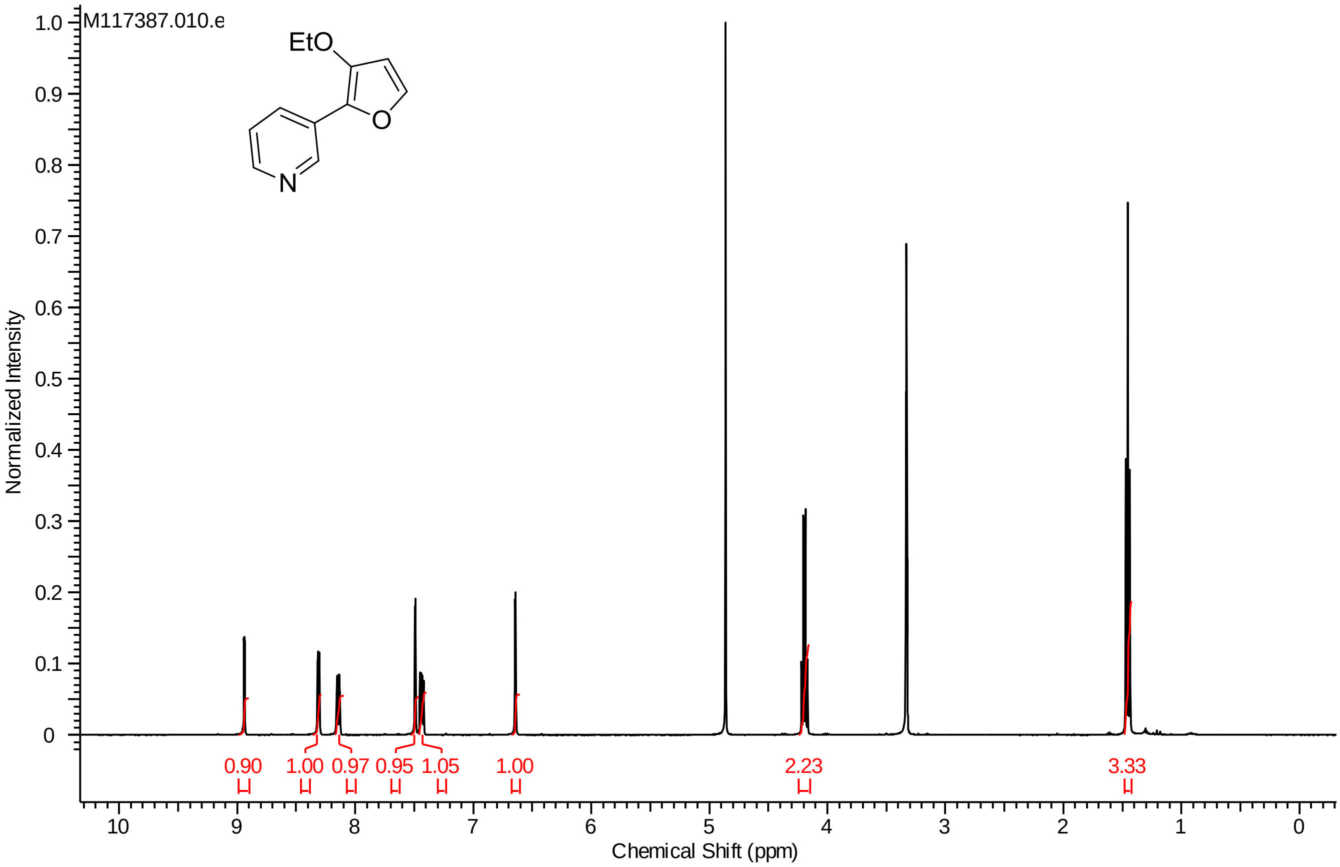

^{13}C NMR (100 MHz, DMSO-d_6)

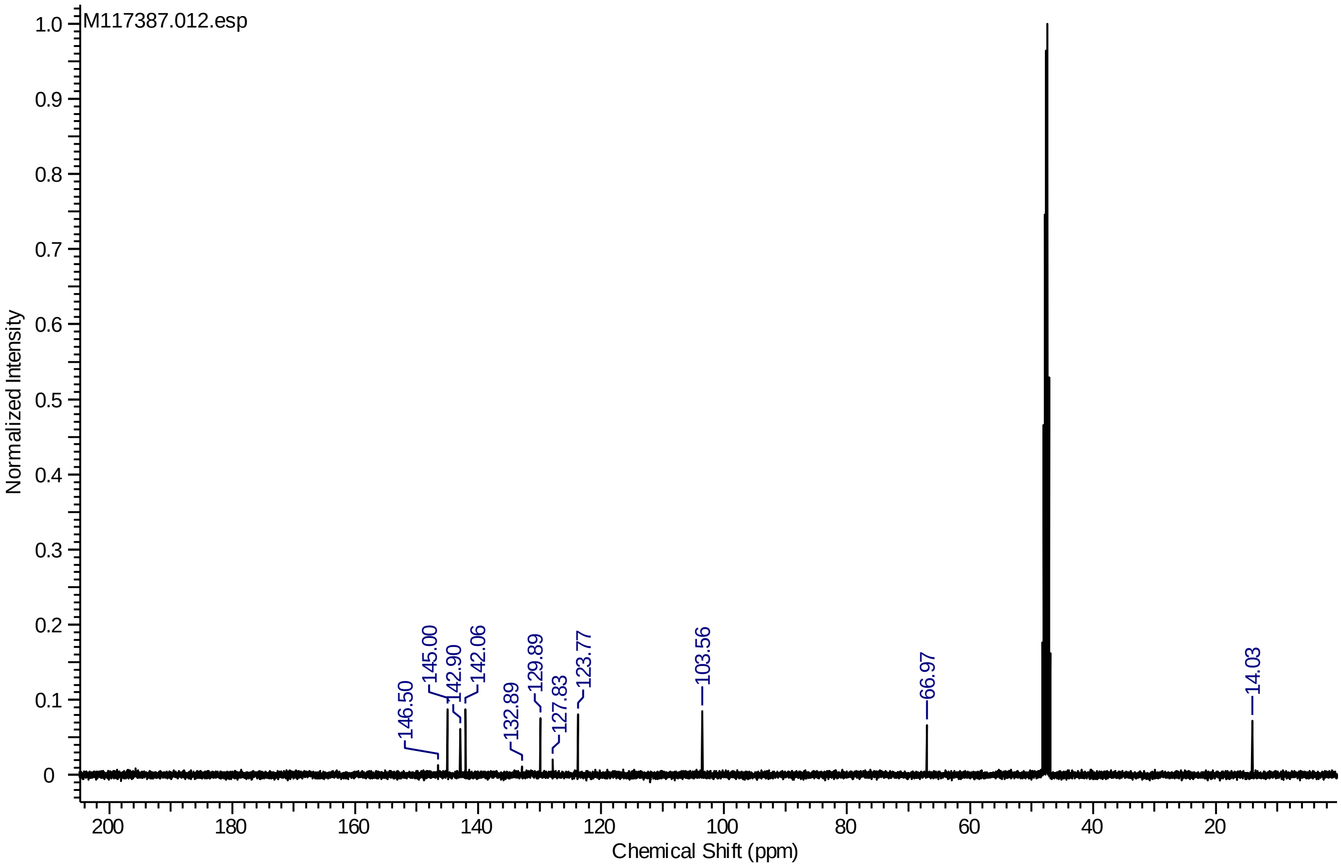

1-(4-Ethoxy-5-phenethylfuran-2-yl)-*N*,*N*-dimethylmethanamine 2o

^{1}H NMR (600 MHz, MeOH-d$_4$)

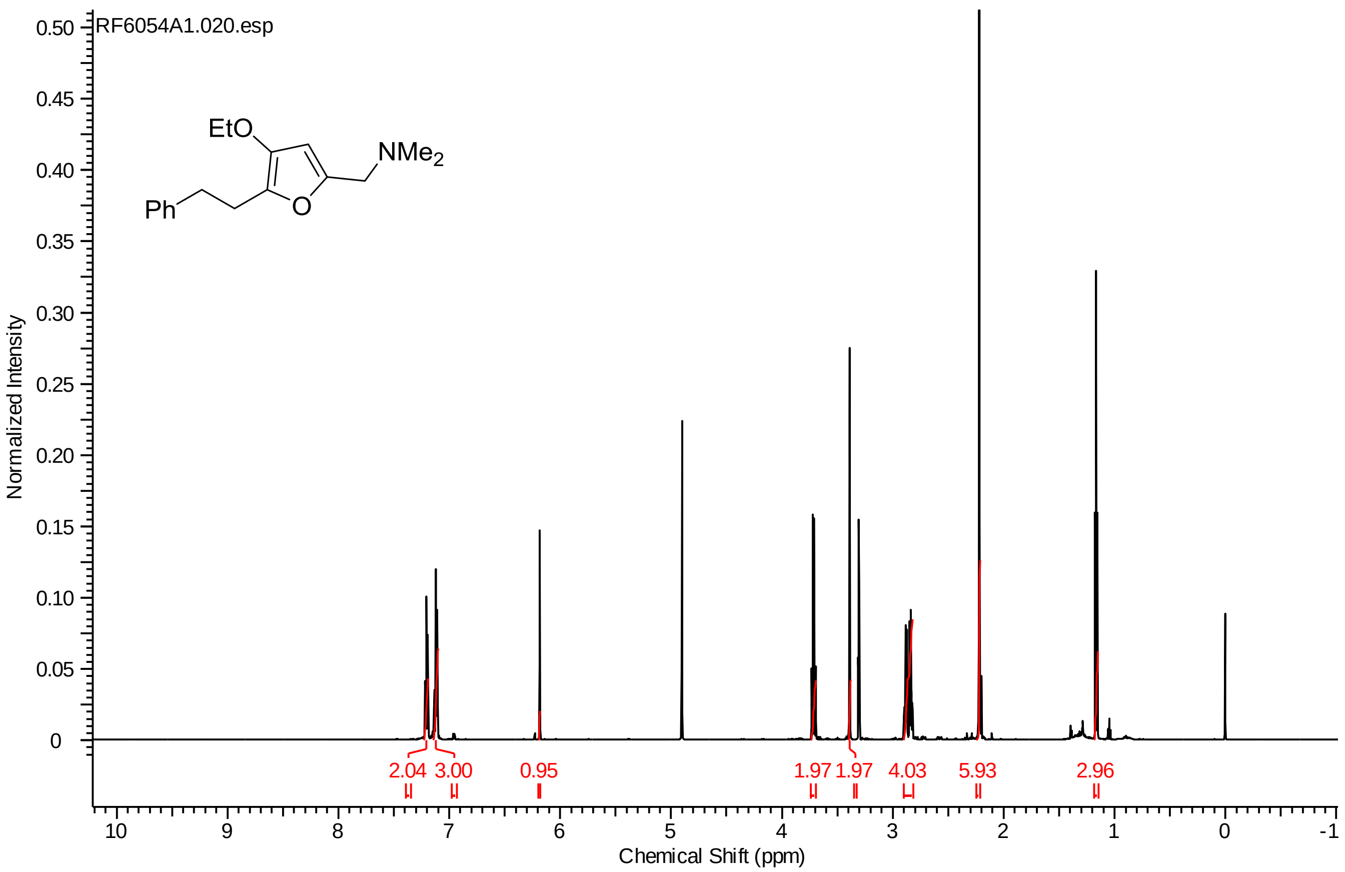

^{13}C NMR (150 MHz, MeOH-d$_4$)

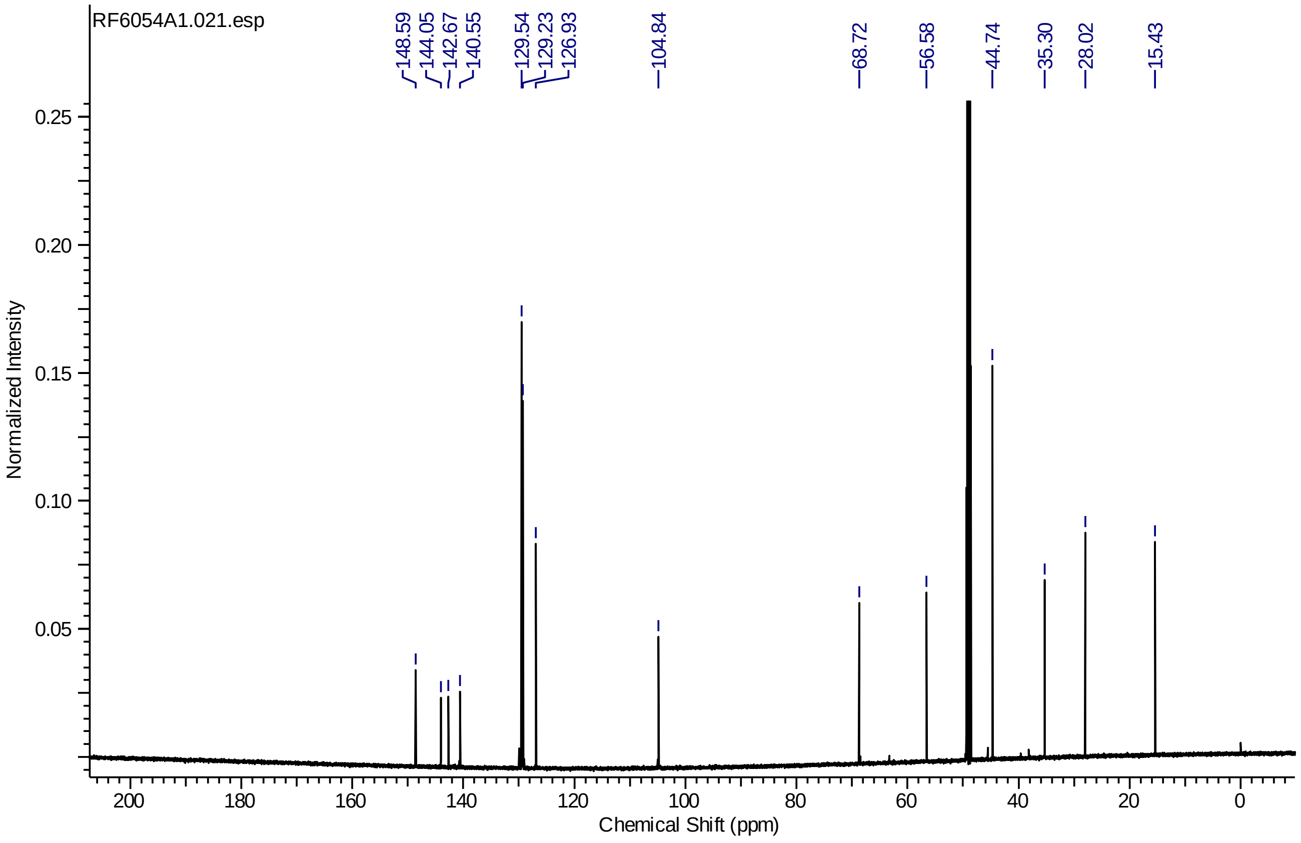

(3a*S*,4*R*,7*R*,7a*R*)-5-Ethoxy-2-methyl-4-phenethyl-3a,4,7,7a-tetrahydro-1*H*-4,7-epoxyisoindole-1,3(2*H*)-dione *endo*-3a

1H NMR (400 MHz, $CDCl_3$)

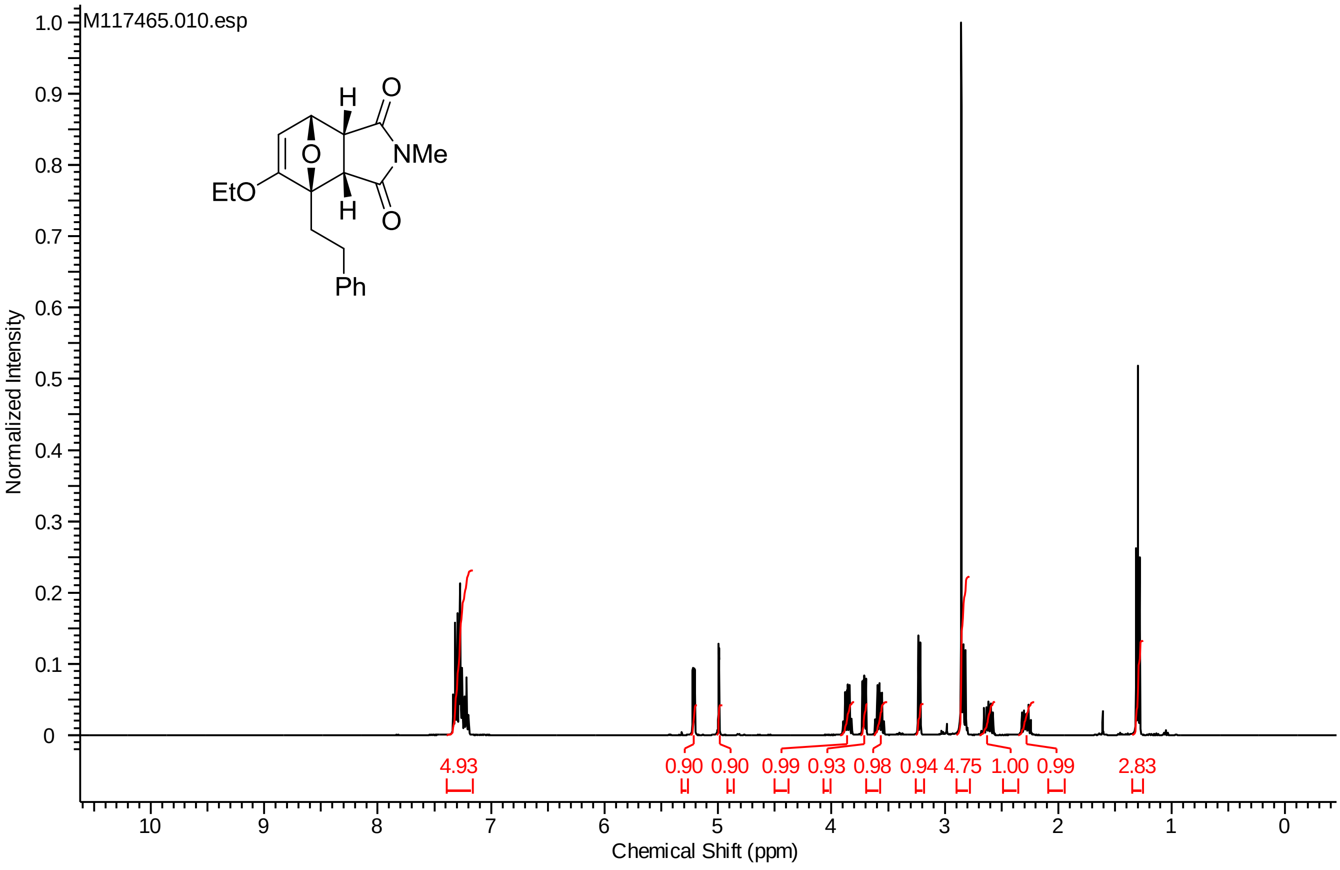

^{13}C NMR (100 MHz, $CDCl_3$)

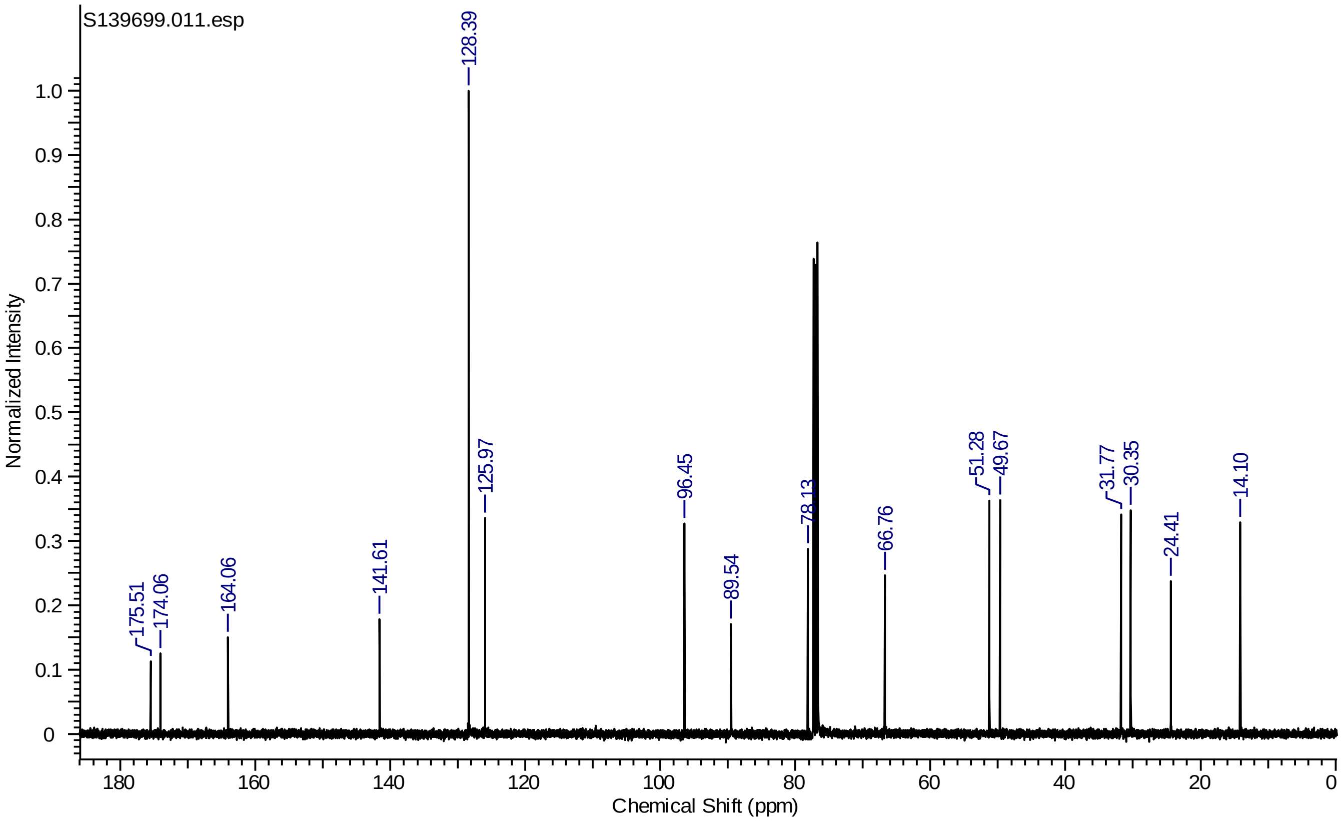

(3a*R*,4*R*,7*R*,7a*S*)-5-Ethoxy-2-methyl-4-phenethyl-3a,4,7,7a-tetrahydro-1H-4,7-epoxyisoindole-1,3(2H)-dione *exo*-3a

^{1}H NMR (400 MHz, MeOH-d$_4$)

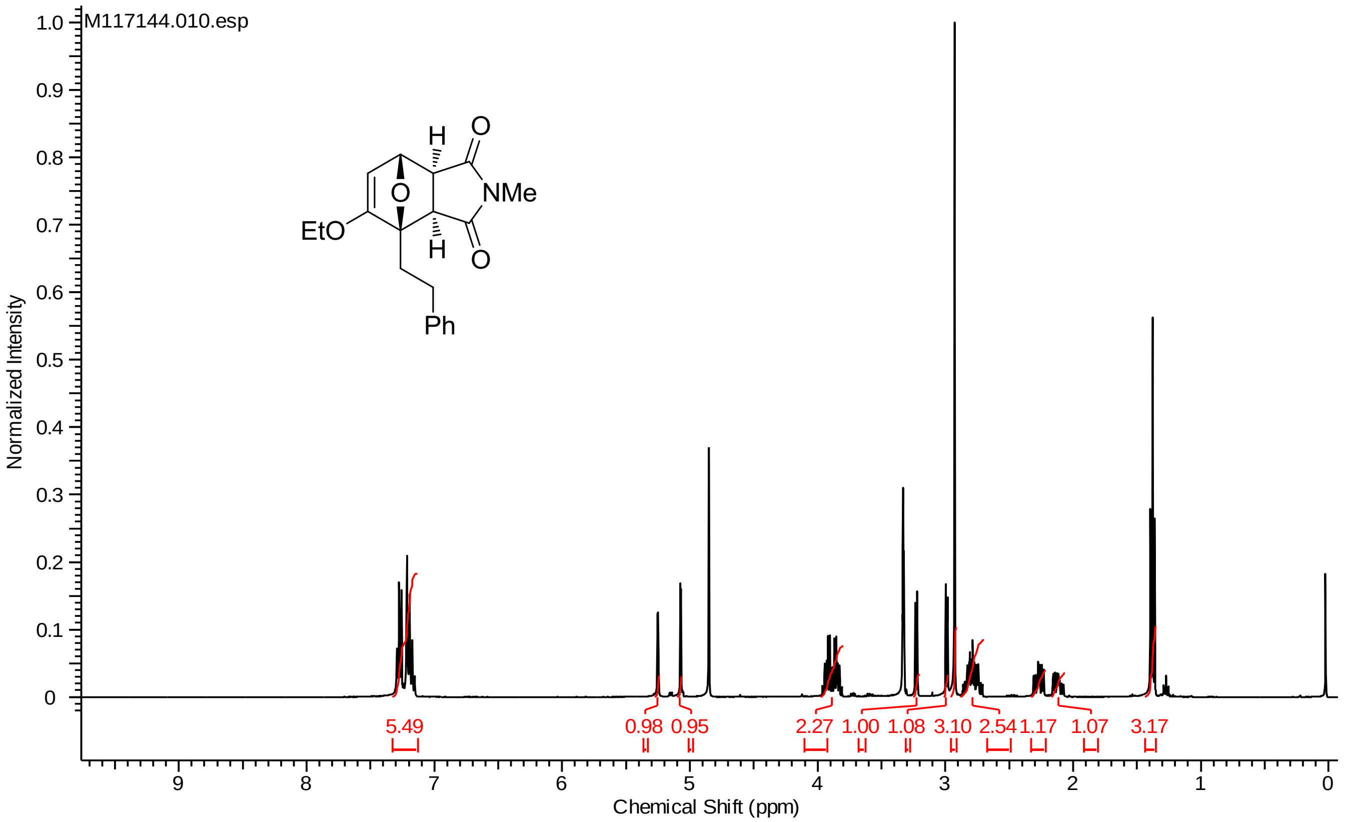

^{13}C NMR (100 MHz, MeOH-d$_4$)

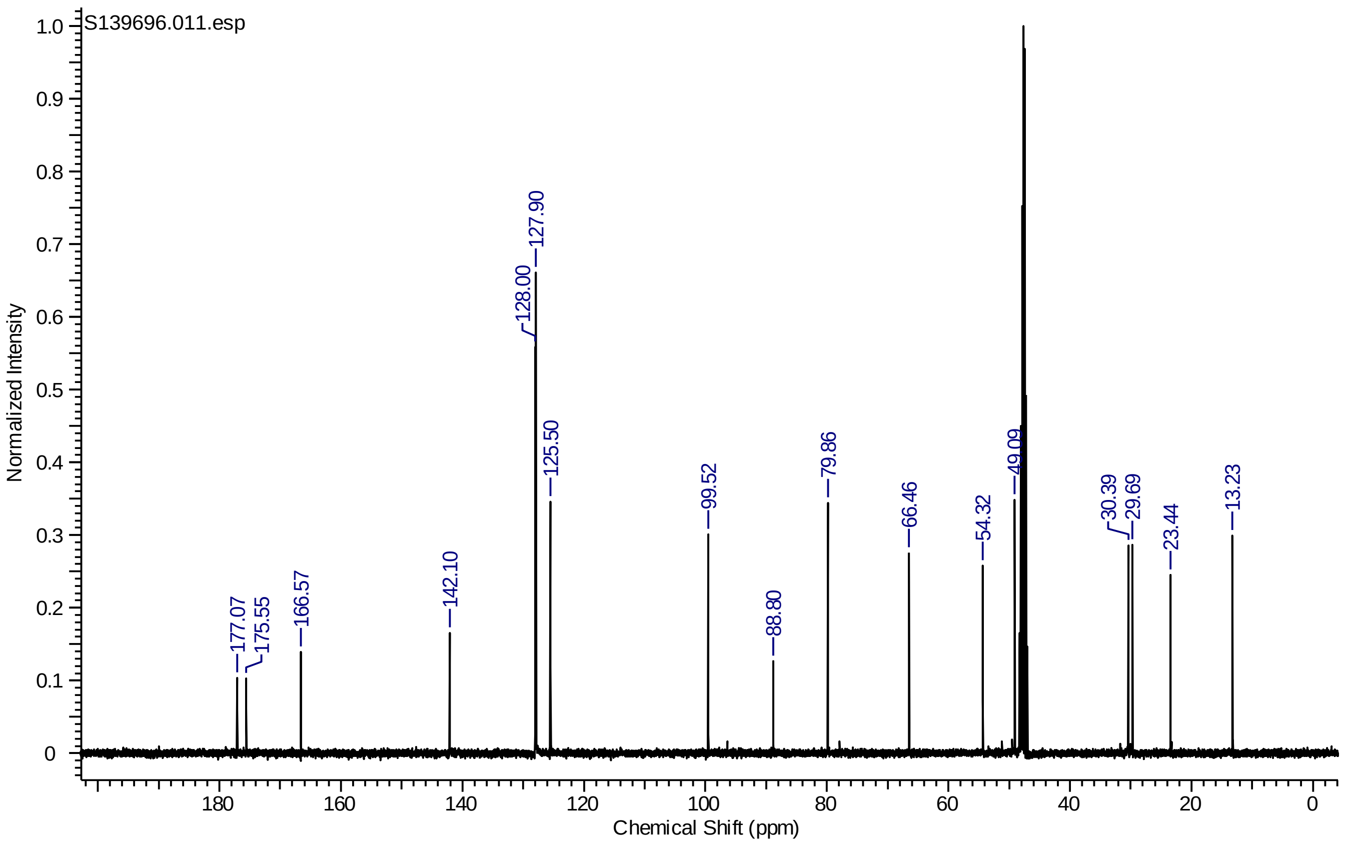

(3a*S*,4*R*,7*R*,7a*R*)-5-Methoxy-2-methyl-4-phenethyl-3a,4,7,7a-tetrahydro-1*H*-4,7-epoxyisoindole-1,3(2*H*)-dione *endo*-3b

^{1}H NMR (400 MHz, $CDCl_3$)

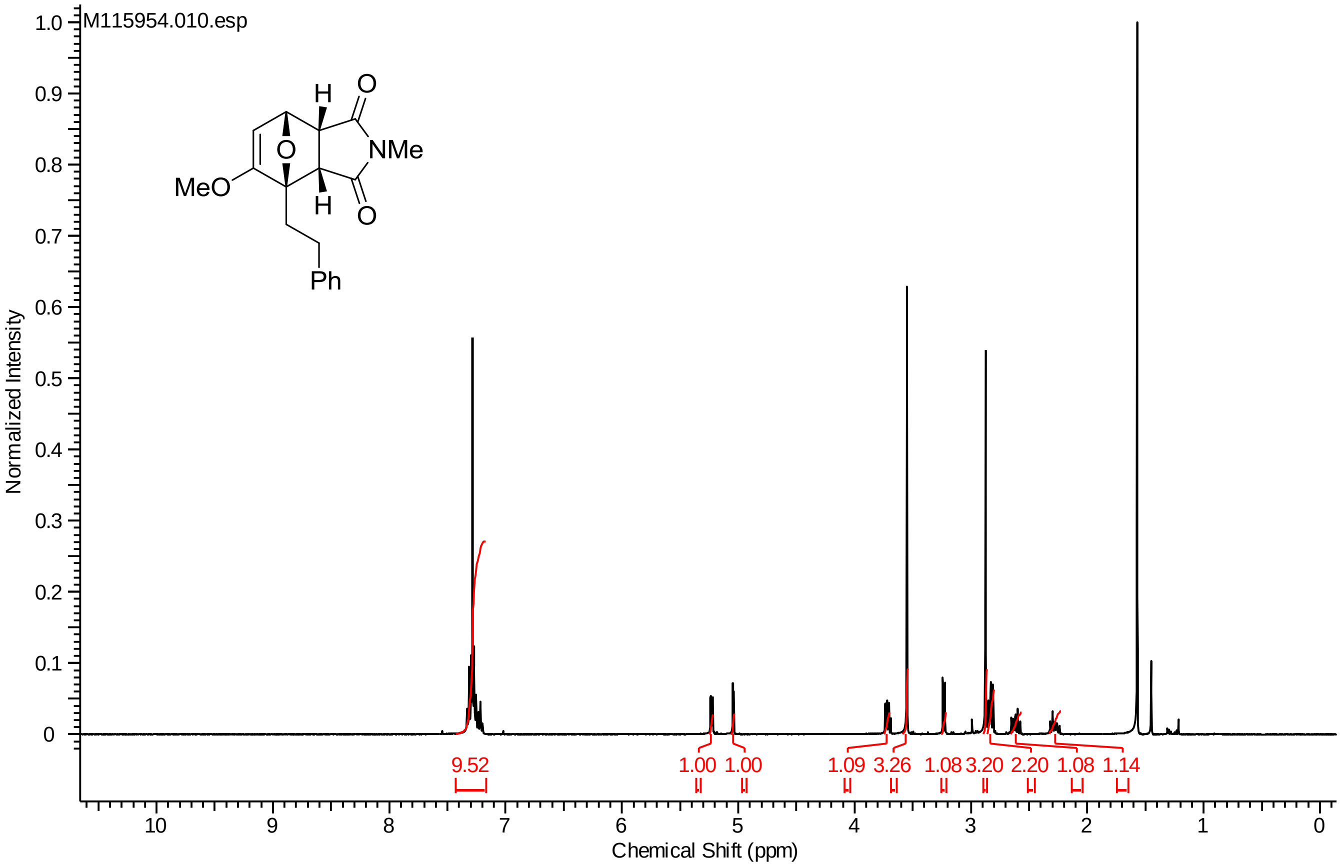

^{13}C NMR (150 MHz, $CDCl_3$)

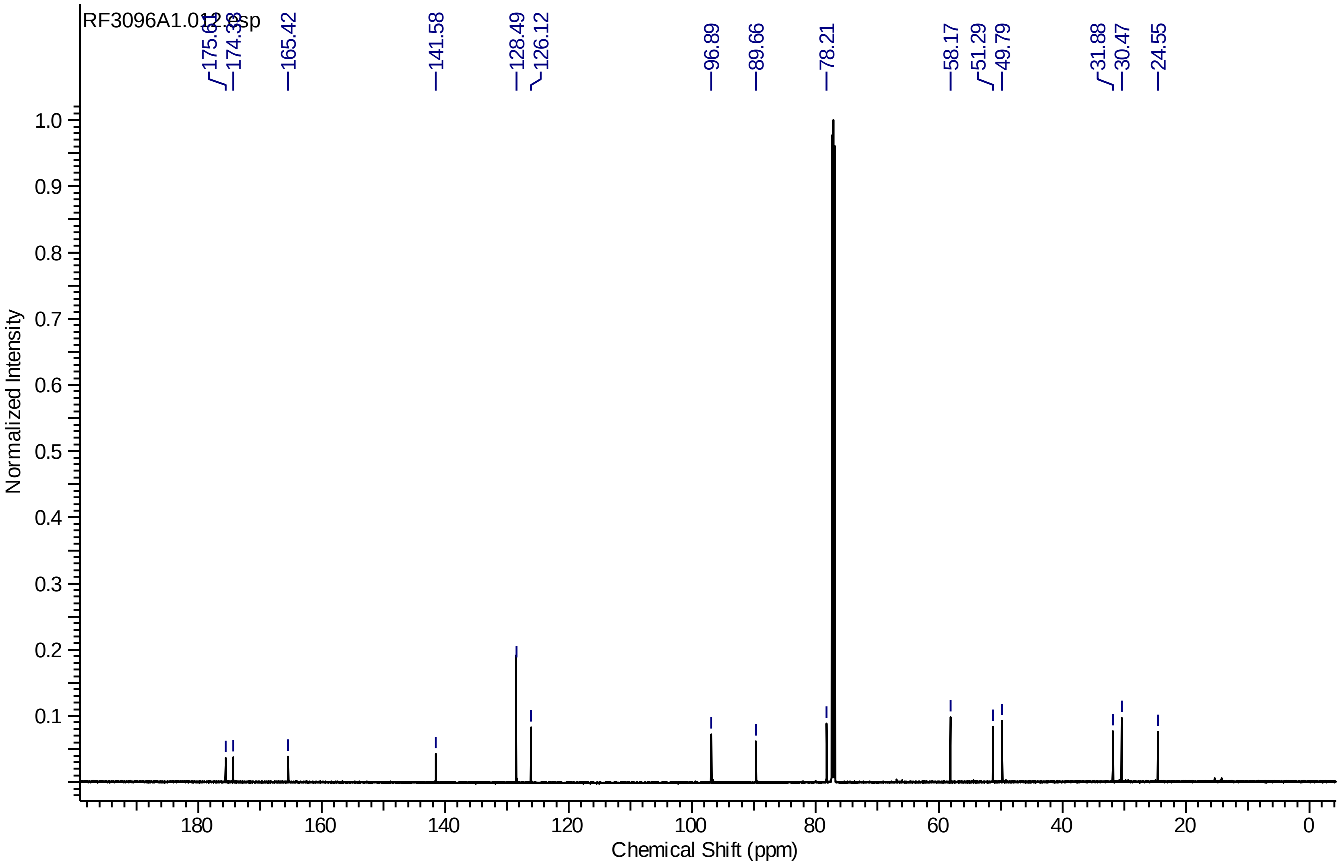

(3a*R*,4*R*,7*R*,7a*S*)-5-Methoxy-2-methyl-4-phenethyl-3a,4,7,7a-tetrahydro-1*H*-4,7-epoxyisoindole-1,3(2*H*)-dione *exo*-3b

^{1}H NMR (400 MHz, $CDCl_3$)

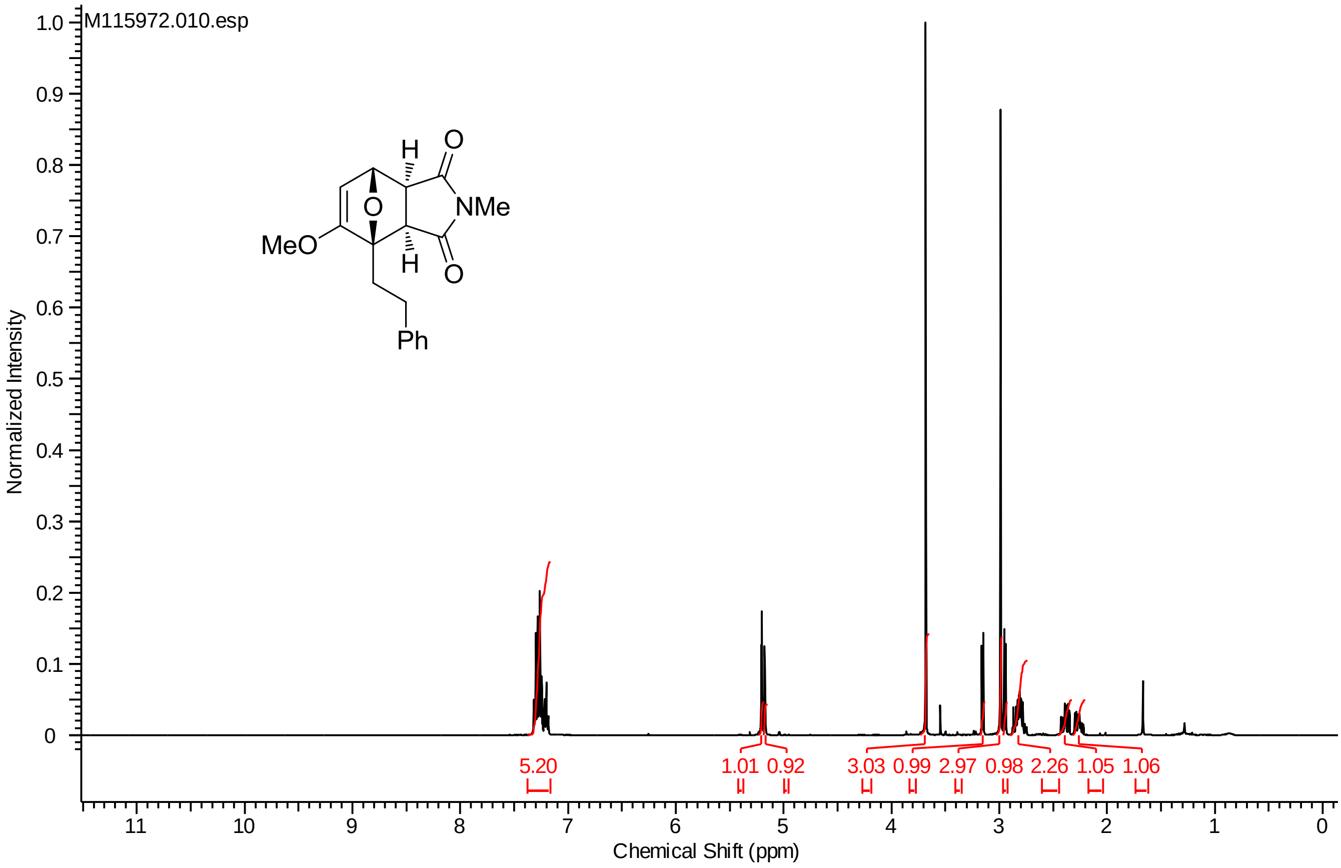

^{13}C NMR (100 MHz, $CDCl_3$)

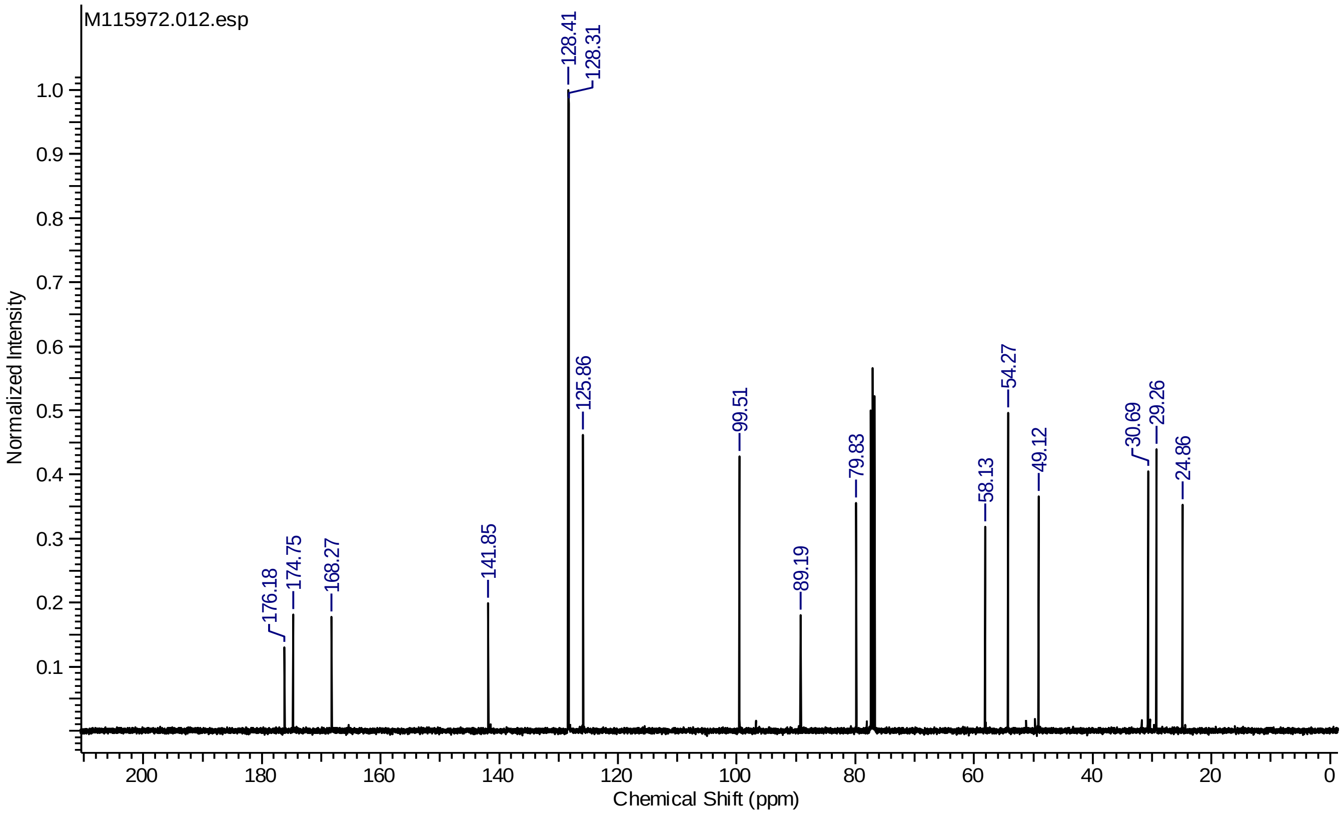

(3a*S*,4*R*,7*R*,7a*R*)-4-Cyclohexyl-5-ethoxy-2-methyl-3a,4,7,7a-tetrahydro-1*H*-4,7-epoxyisoindole-1,3(2*H*)-dione e*ndo*-3c and (3a*R*,4*R*,7*R*,7a*S*)-4-Cyclohexyl-5-ethoxy-2-methyl-3a,4,7,7a-tetrahydro-1*H*-4,7-epoxyisoindole-1,3(2*H*)-dione e*xo*-3c

^{1}H NMR (400 MHz, MeOH-d$_4$)

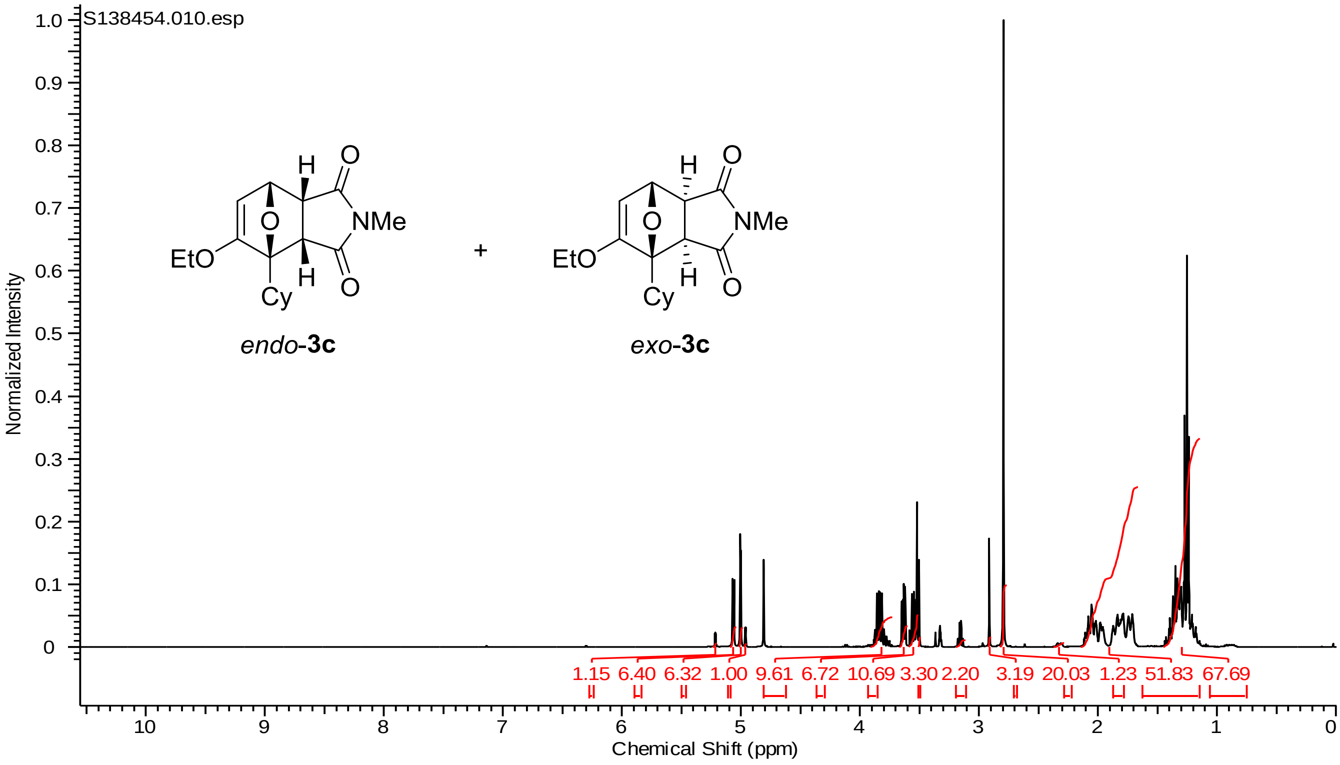

^{13}C NMR (100 MHz, MeOH-d$_4$)

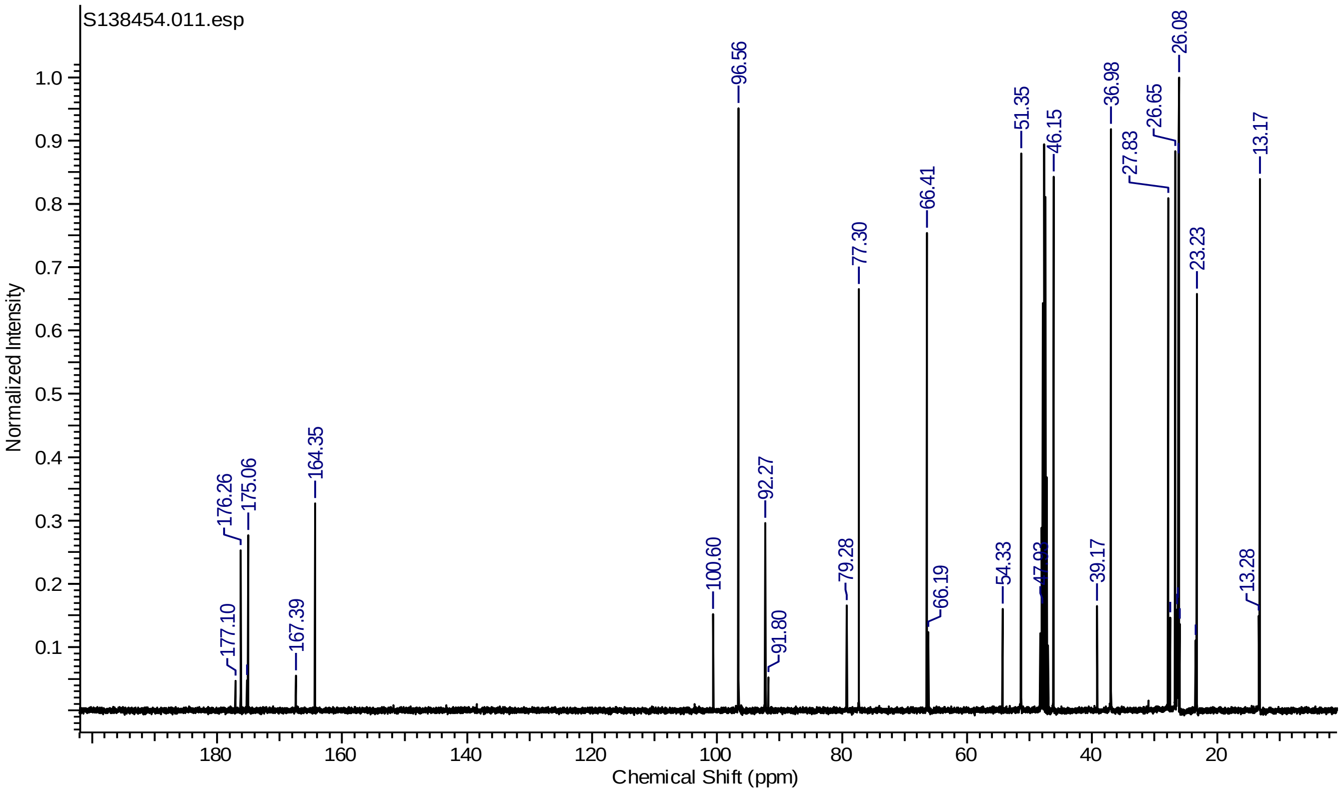

(3a*S*,4*R*,7*R*,7a*R*)-4-Cyclopropyl-5-ethoxy-2-methyl-3a,4,7,7a-tetrahydro-1*H*-4,7-epoxyisoindole-1,3(2*H*)-dione *endo*-3d and (3a*R*,4*R*,7*R*,7a*S*)-4-Cyclopropyl-5-ethoxy-2-methyl-3a,4,7,7a-tetrahydro-1*H*-4,7-epoxyisoindole-1,3(2*H*)-dione *exo*-3d

^{1}H NMR (400 MHz, MeOH-d$_4$)

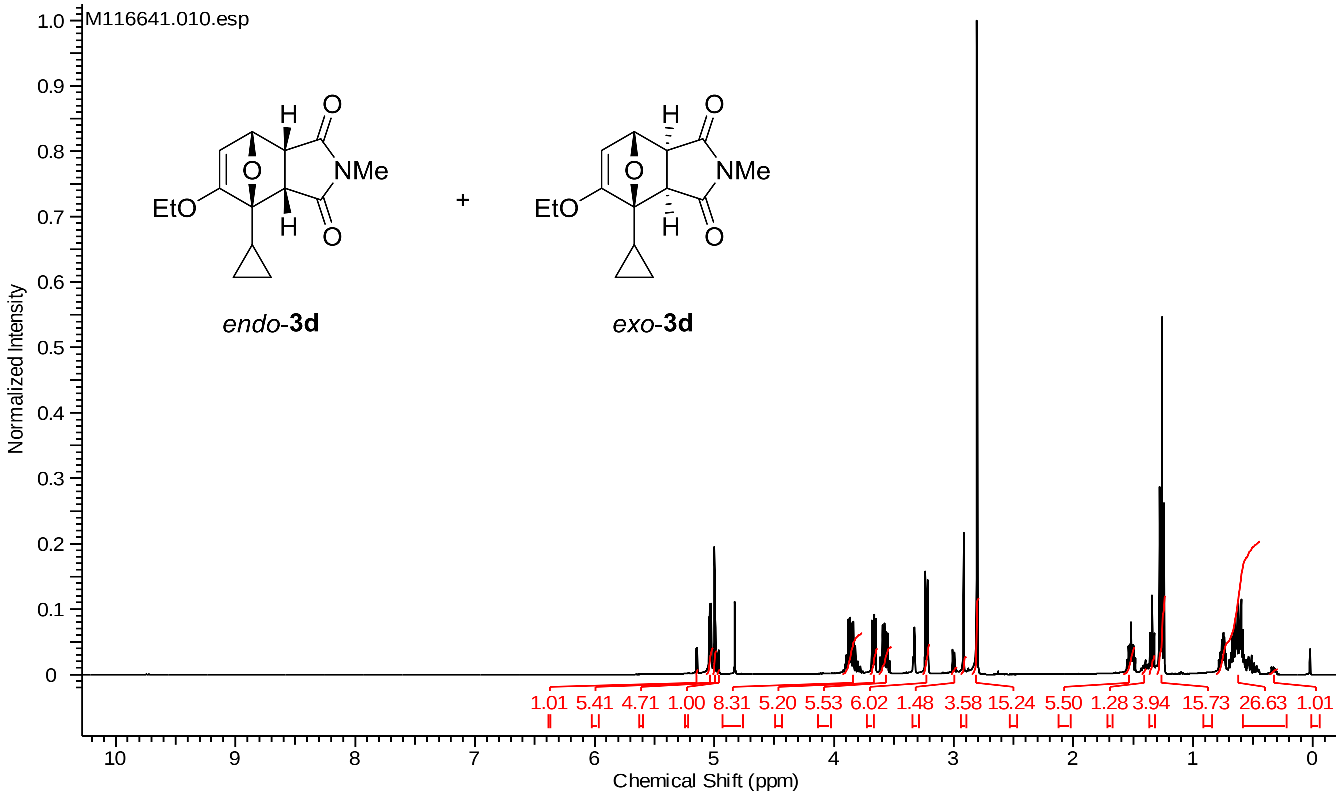

^{1}H NMR (100 MHz, MeOH-d$_4$)

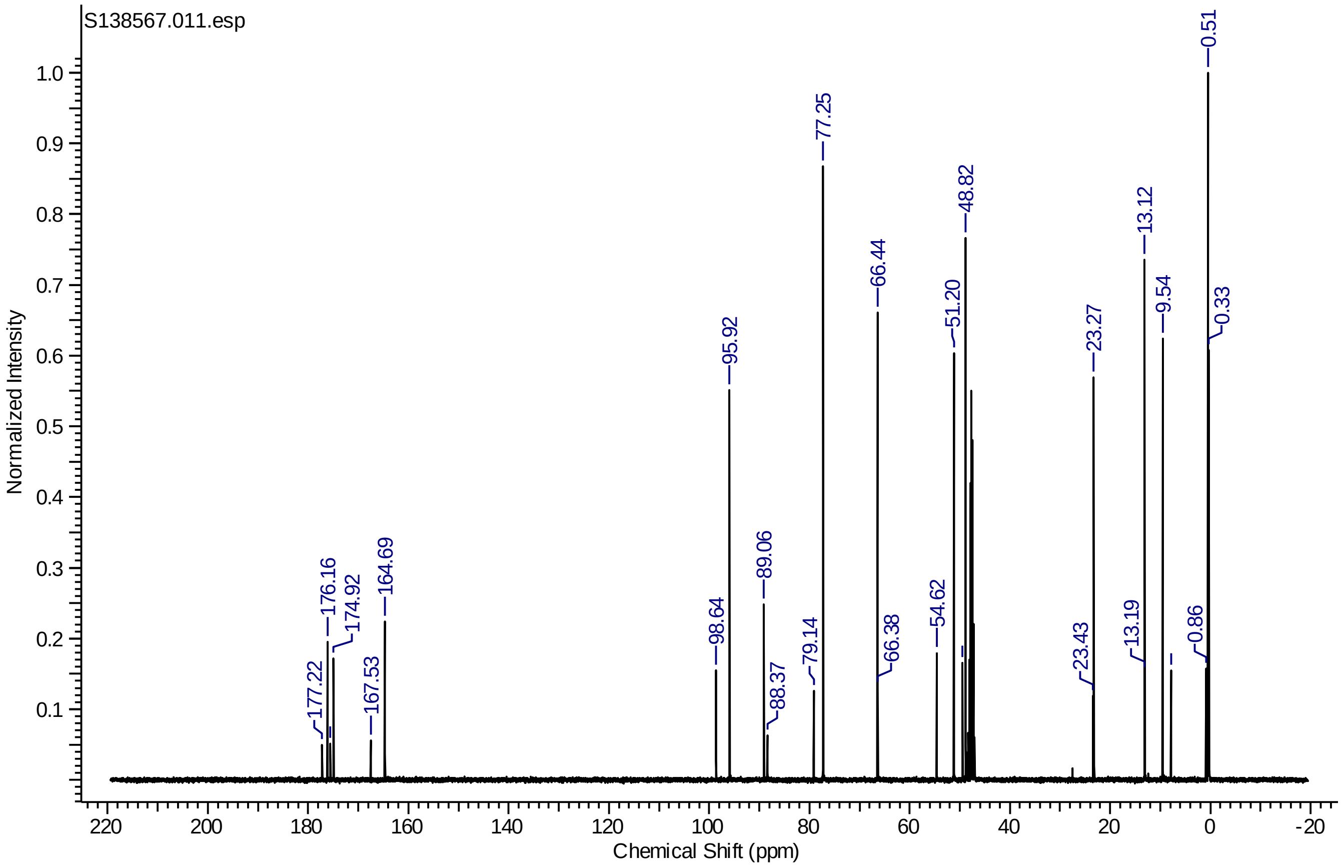

tert-Butyl 4-(((3a*S*,4*R*,7*R*,7a*R*)-5-ethoxy-2-methyl-1,3-dioxo-2,3,3a,4,7,7a-hexahydro-1*H*-4,7-epoxyisoindol-4-yl)methyl)piperidine-1-carboxylate *endo*-3e and *tert*-Butyl 4-(((3a*R*,4*R*,7*R*,7a*S*)-5-ethoxy-2-methyl-1,3-dioxo-2,3,3a,4,7,7a-hexahydro-1*H*-4,7-epoxyisoindol-4-yl)methyl)piperidine-1-carboxylate *exo*-3e

^{1}H NMR (600 MHz, $CDCl_3$)

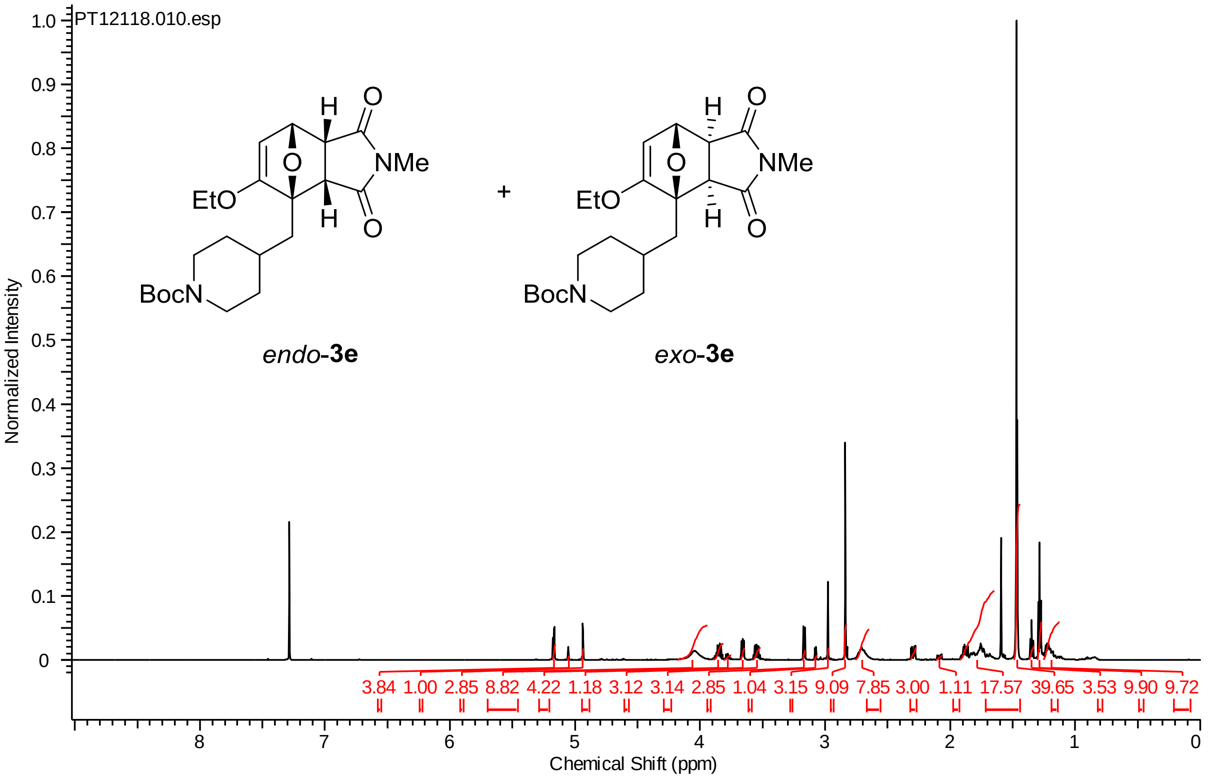

^{13}C NMR (150 MHz, $CDCl_3$)

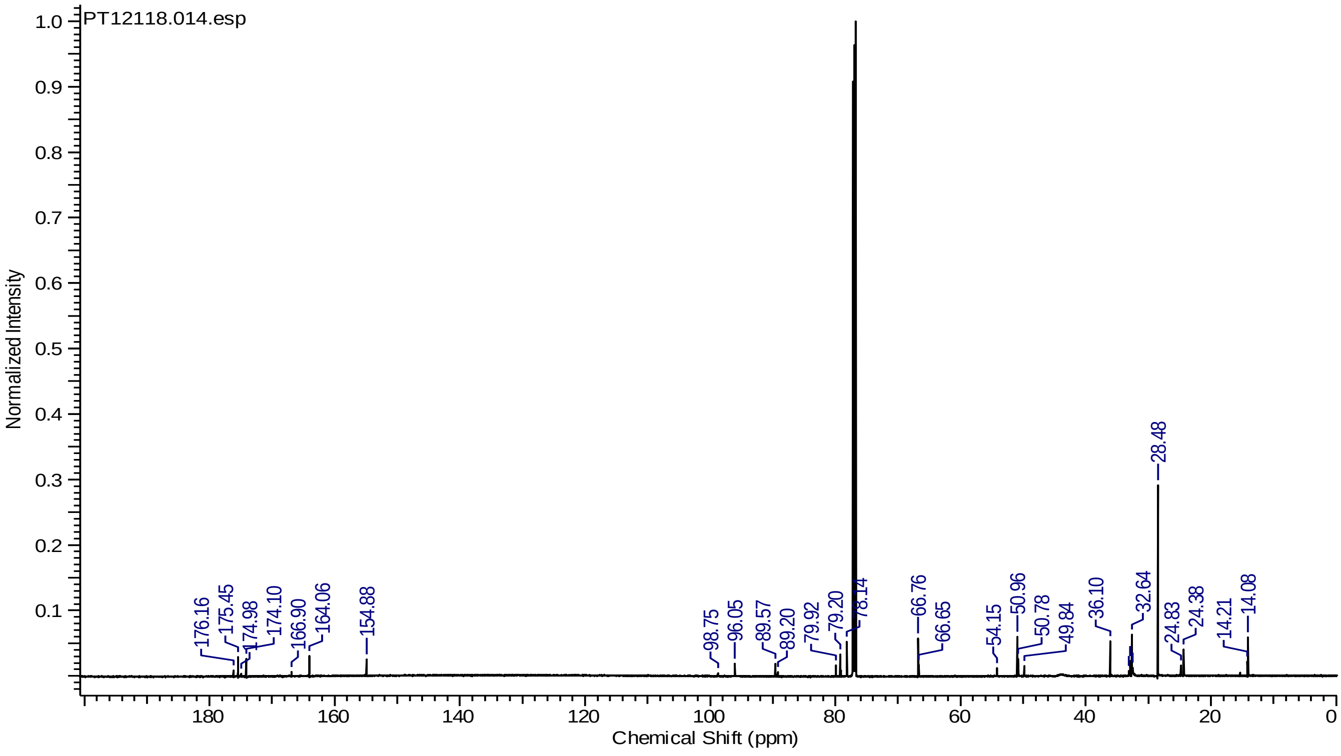

(3a*S*,4*S*,7*R*,7a*R*)-5-Ethoxy-2-methyl-4-phenyl-3a,4,7,7a-tetrahydro-1*H*-4,7-epoxyisoindole-1,3(2*H*)-dione *endo*-3f

^{1}H NMR (400 MHz, $CDCl_3$)

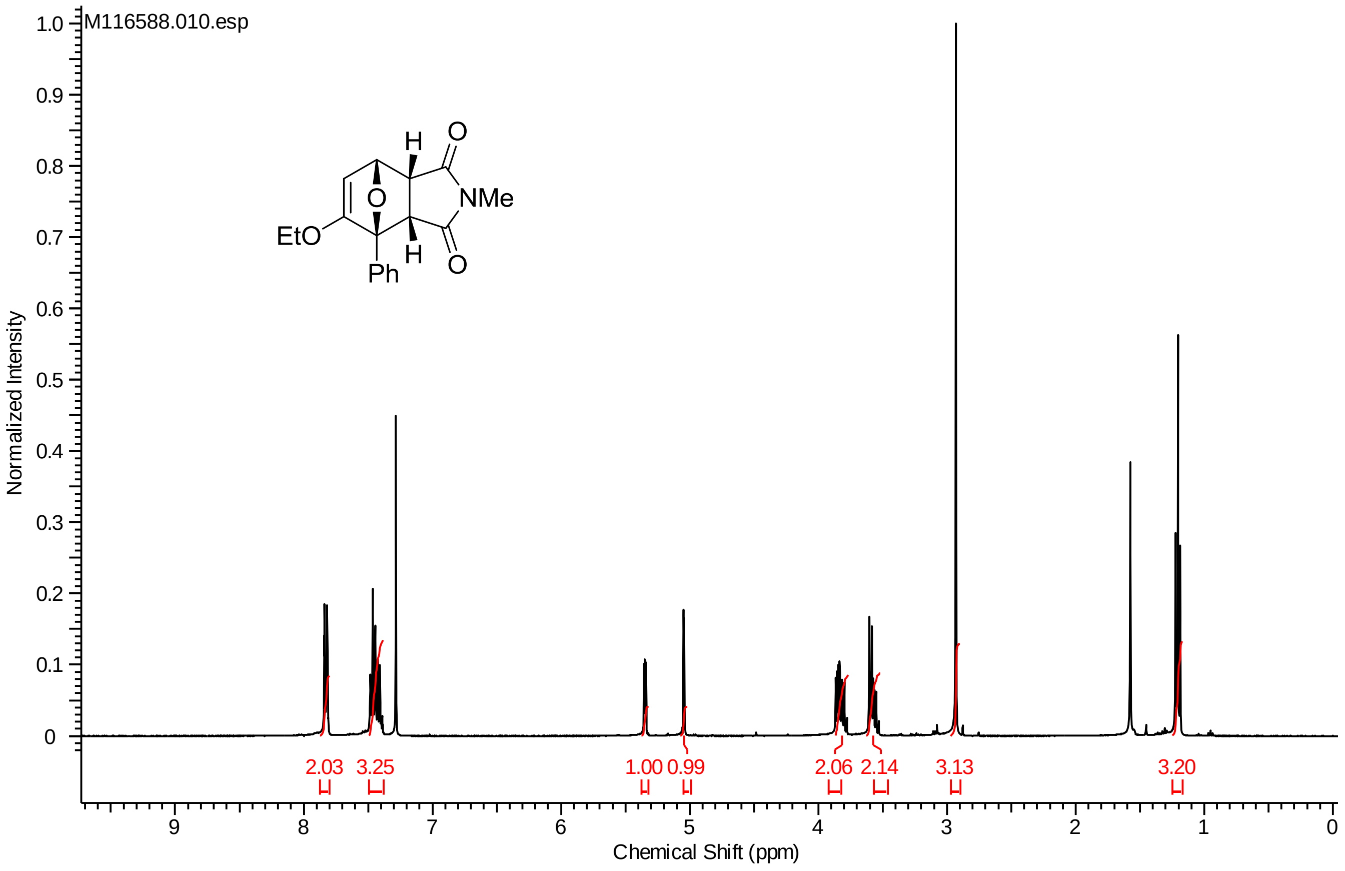

^{13}C NMR (100 MHz, $CDCl_3$)

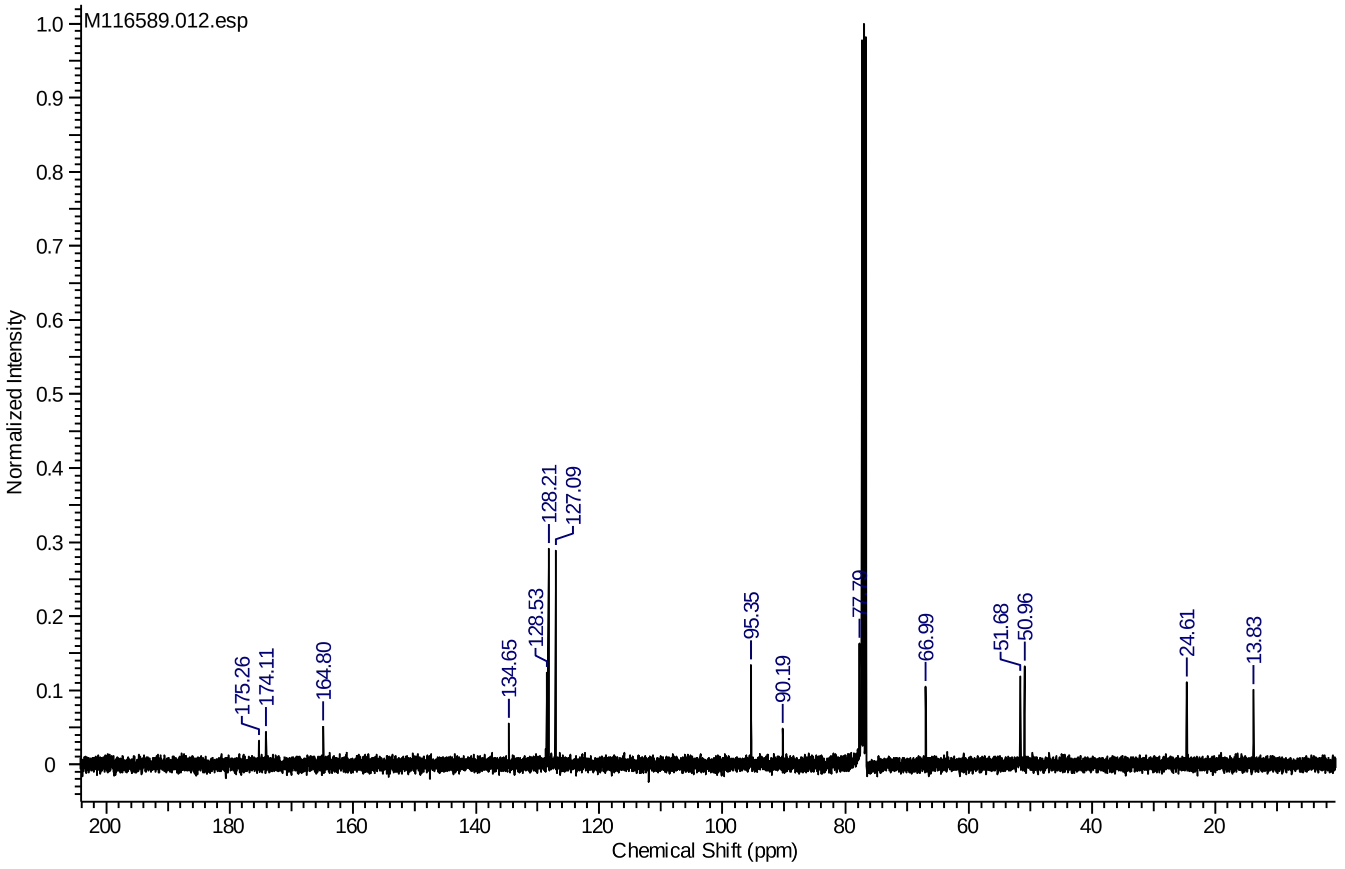

(3a*R*,4*S*,7*R*,7a*S*)-5-Ethoxy-2-methyl-4-phenyl-3a,4,7,7a-tetrahydro-1*H*-4,7-epoxyisoindole-1,3(2*H*)-dione *exo*-3f

^{1}H NMR (400 MHz, $CDCl_3$)

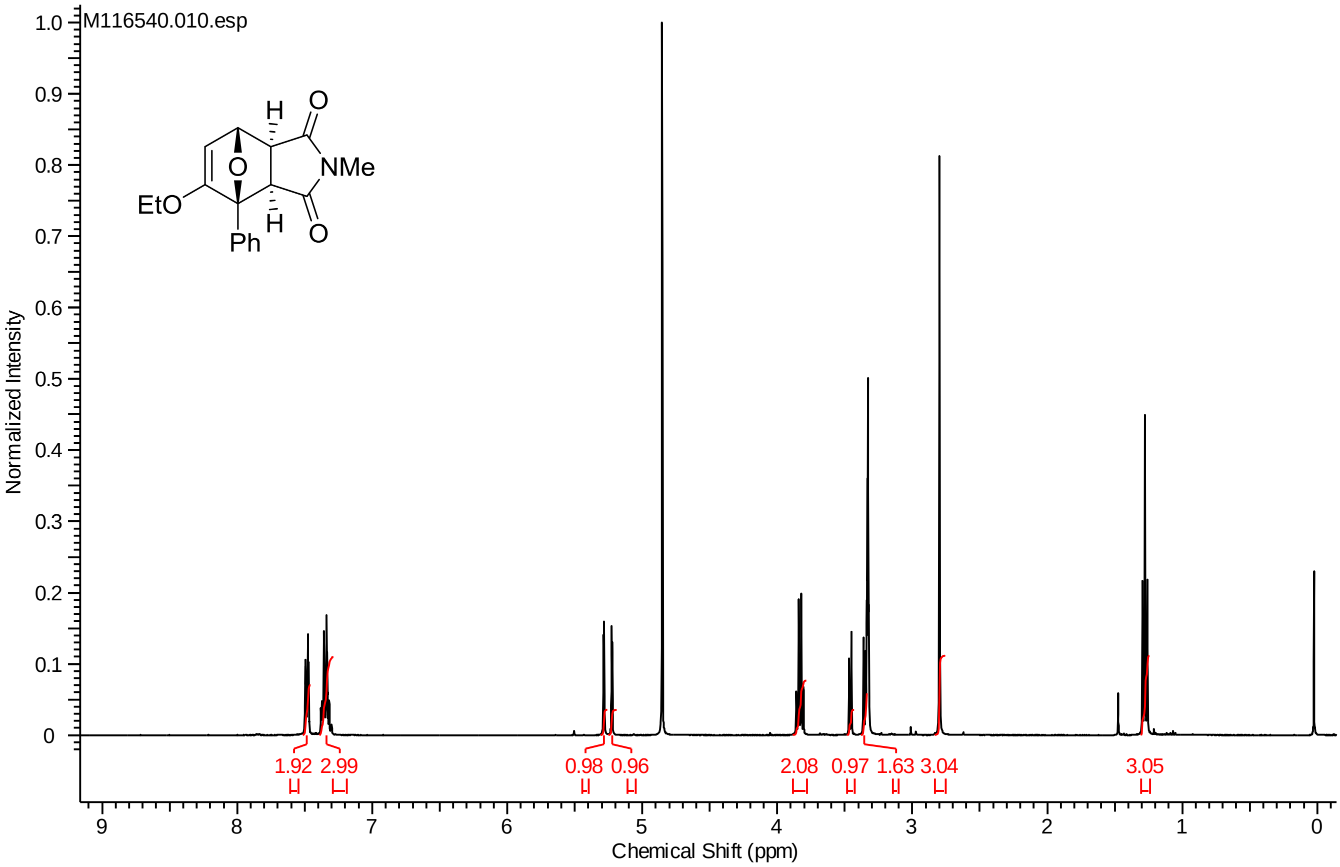

^{13}C NMR (100 MHz, $CDCl_3$)

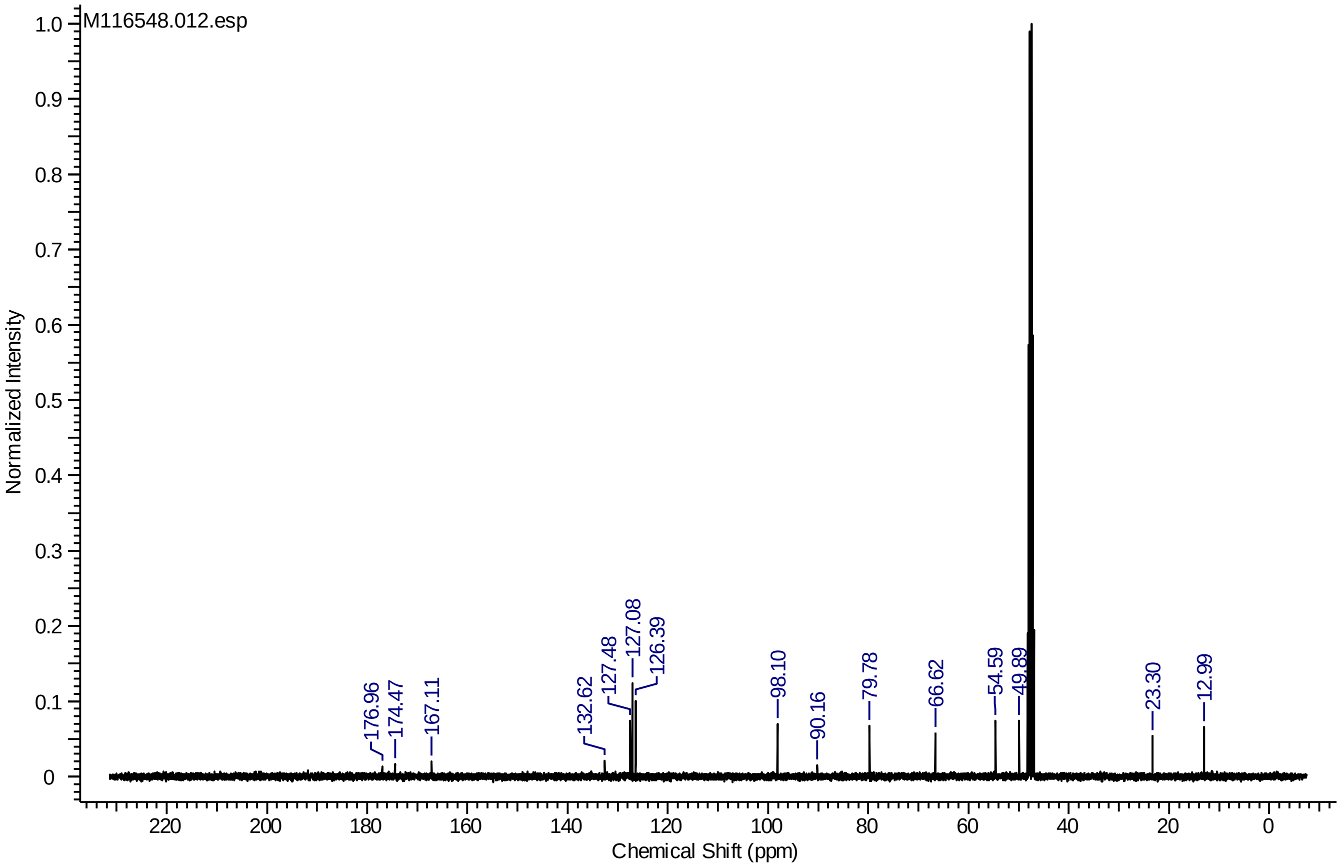

(3a*S*,4*S*,7*R*,7a*R*)-5-Ethoxy-2-methyl-4-(4-(trifluoromethyl)phenyl)-3a,4,7,7a-tetrahydro-1*H*-4,7-epoxyisoindole-1,3(2*H*)-dione *endo*-3g and (3a*R*,4*S*,7*R*,7a*S*)-5-Ethoxy-2-methyl-4-(4-(trifluoromethyl)phenyl)-3a,4,7,7a-tetrahydro-1*H*-4,7-epoxyisoindole-1,3(2*H*)-dione *exo*-3g

^{1}H NMR (400 MHz, MeOH-d$_4$)

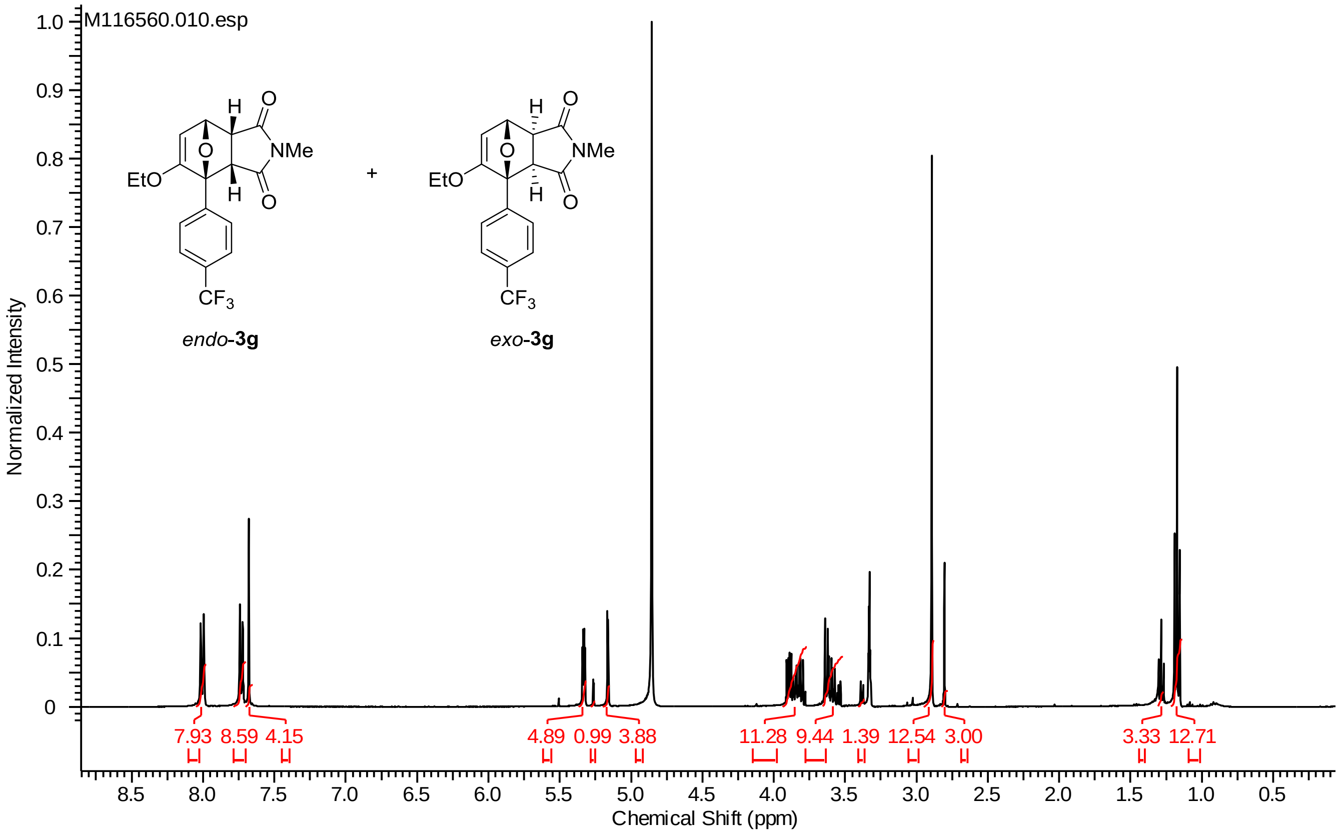

^{13}C NMR (150 MHz, CDCl$_3$)

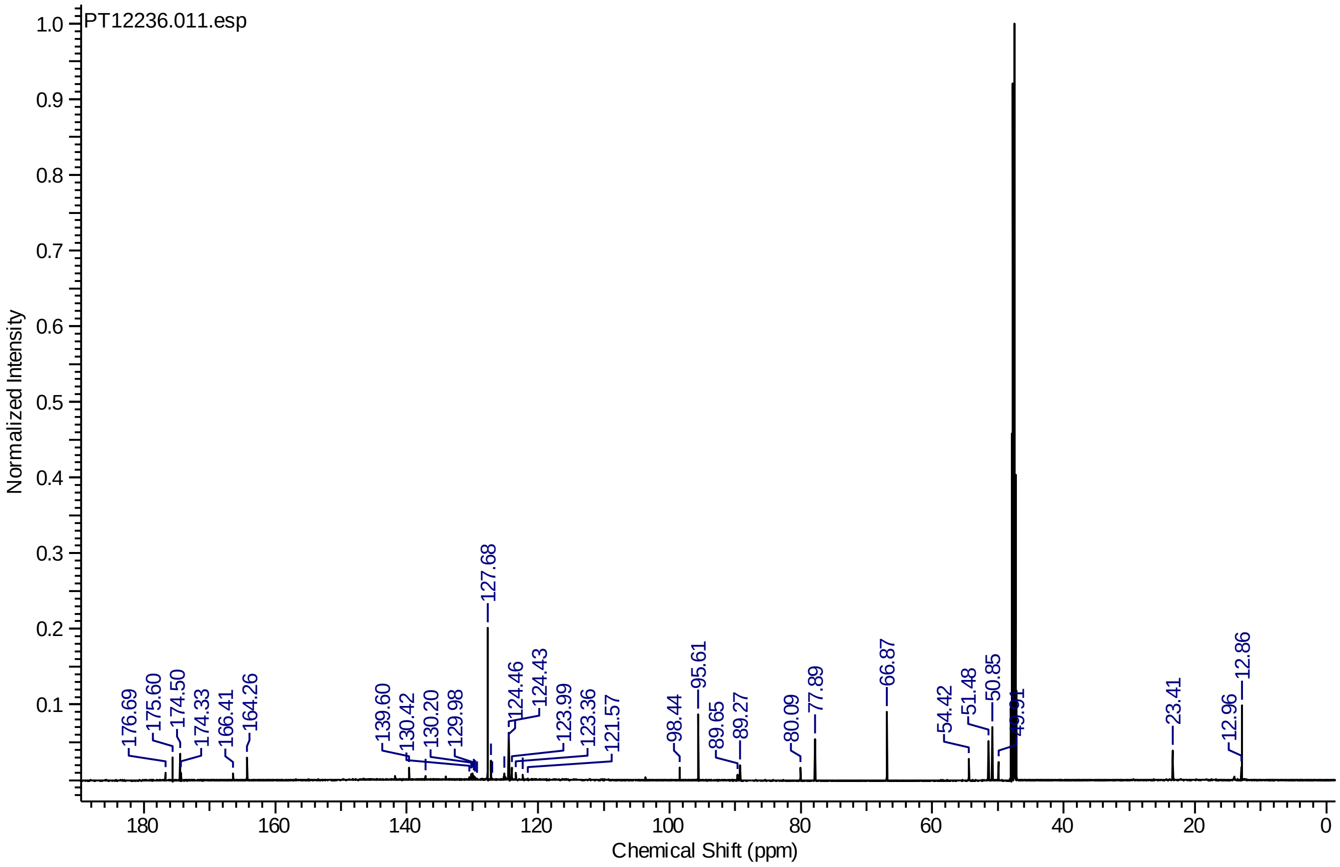

(3a*S*,4*S*,7*R*,7a*R*)-4-(4-Bromophenyl)-5-ethoxy-2-methyl-3a,4,7,7a-tetrahydro-1*H*-4,7-epoxyisoindole-1,3(2*H*)-dione *endo*-3h and (3a*R*,4*S*,7*R*,7a*S*)-4-(4-Bromophenyl)-5-ethoxy-2-methyl-3a,4,7,7a-tetrahydro-1*H*-4,7-epoxyisoindole-1,3(2*H*)-dione *exo*-3h

^{1}H NMR (400 MHz, MeOH-d_4)

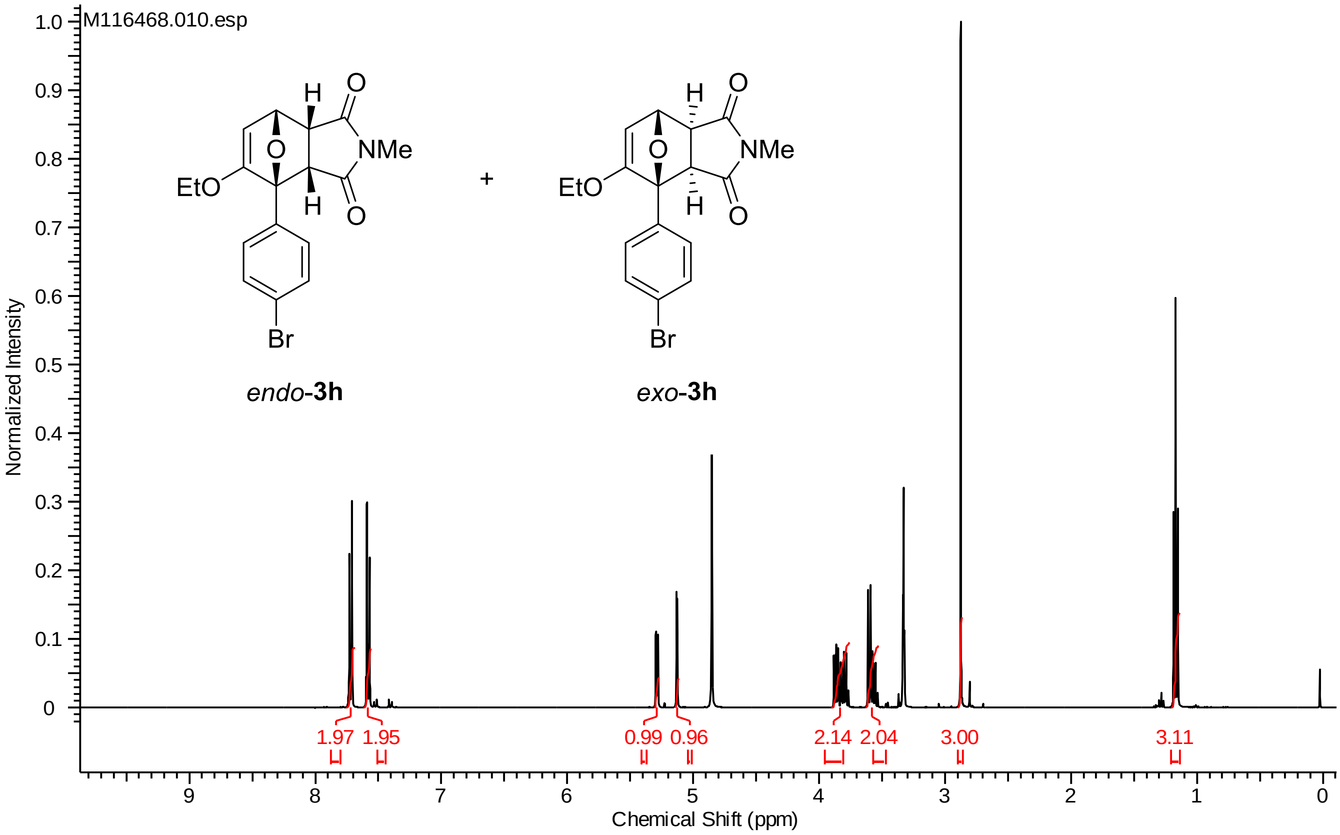

^{13}C NMR (100 MHz, MeOH-d_4)

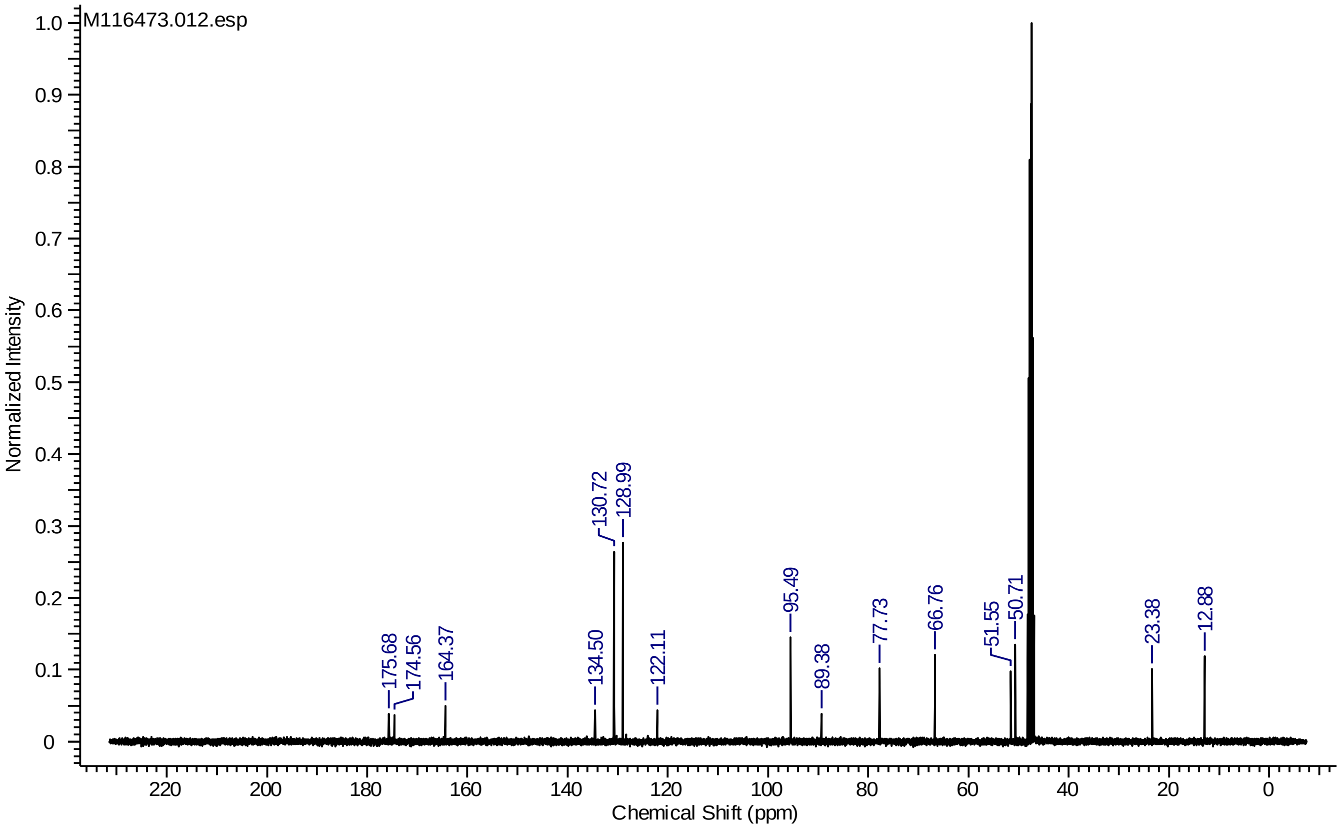

(3a*S*,4*S*,7*R*,7a*R*)-5-Ethoxy-4-(4-methoxyphenyl)-2-methyl-3a,4,7,7a-tetrahydro-1*H*-4,7-epoxyisoindole-1,3(2*H*)-dione *endo*-3i and (3a*R*,4*S*,7*R*,7a*S*)-5-Ethoxy-4-(4-methoxyphenyl)-2-Methyl-3a,4,7,7a-tetrahydro-1*H*-4,7-epoxyisoindole-1,3(2*H*)-dione *exo*-3i

^{1}H NMR (400 MHz, $CDCl_3$)

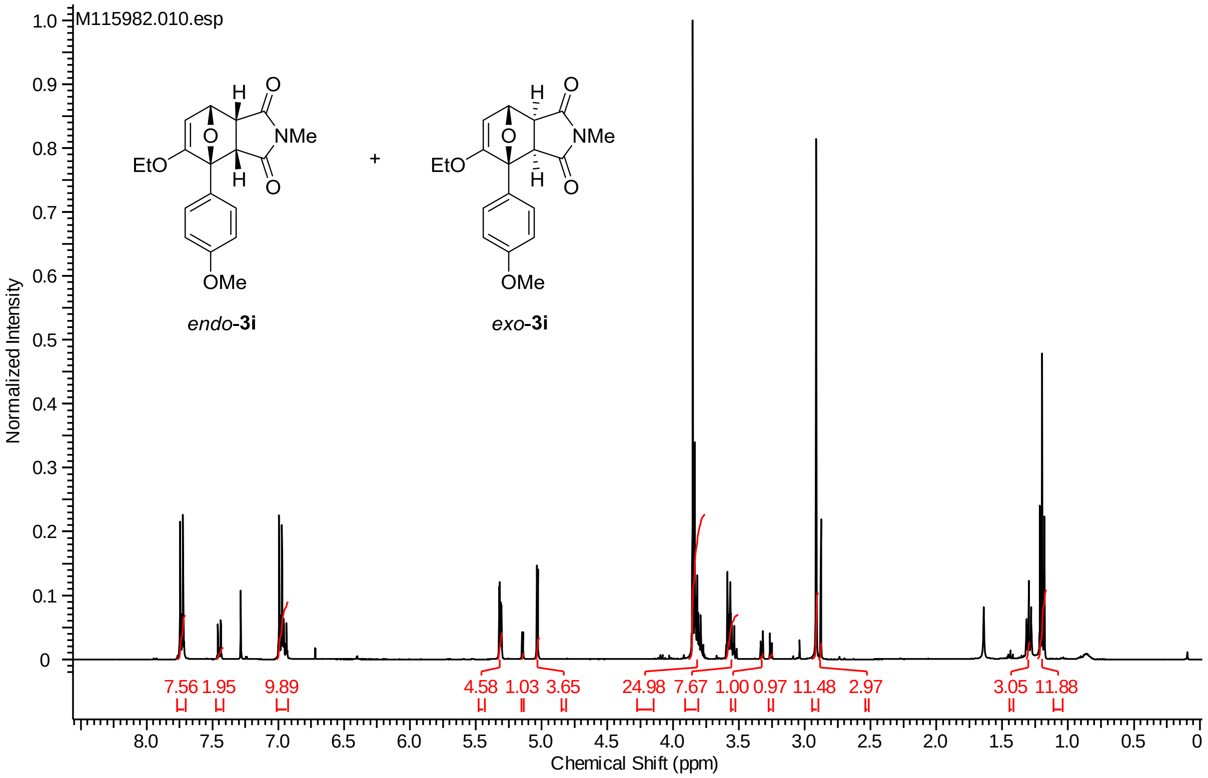

^{13}C NMR (100 MHz, MeOH-d_4)

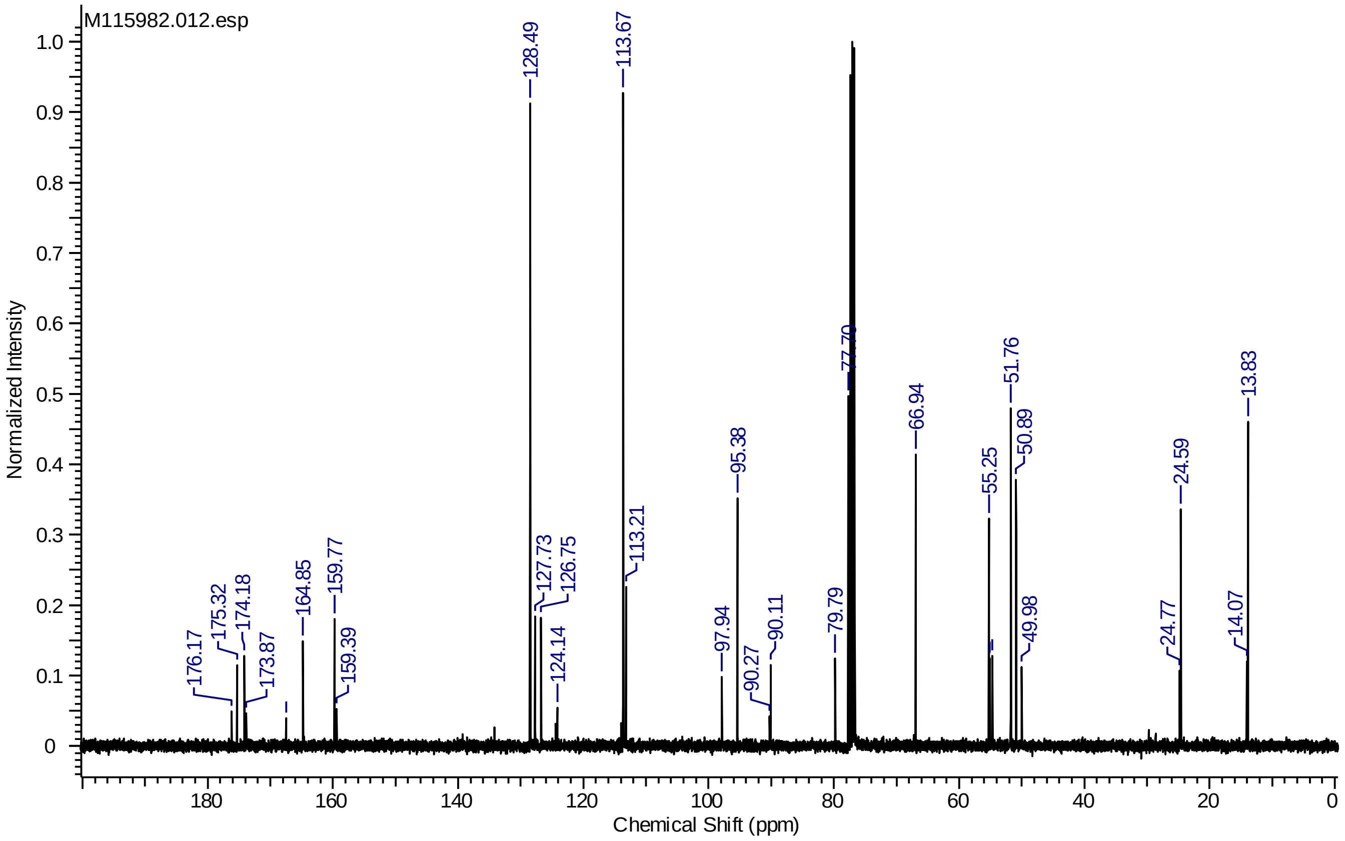

Methyl 3-((3a*S*,4*S*,7*R*,7a*R*)-5-ethoxy-2-methyl-1,3-dioxo-2,3,3a,4,7,7a-hexahydro-1*H*-4,7-epoxyisoindol-4-yl)benzoate *endo*-3j

^{1}H NMR (400 MHz, DMSO-d$_6$)

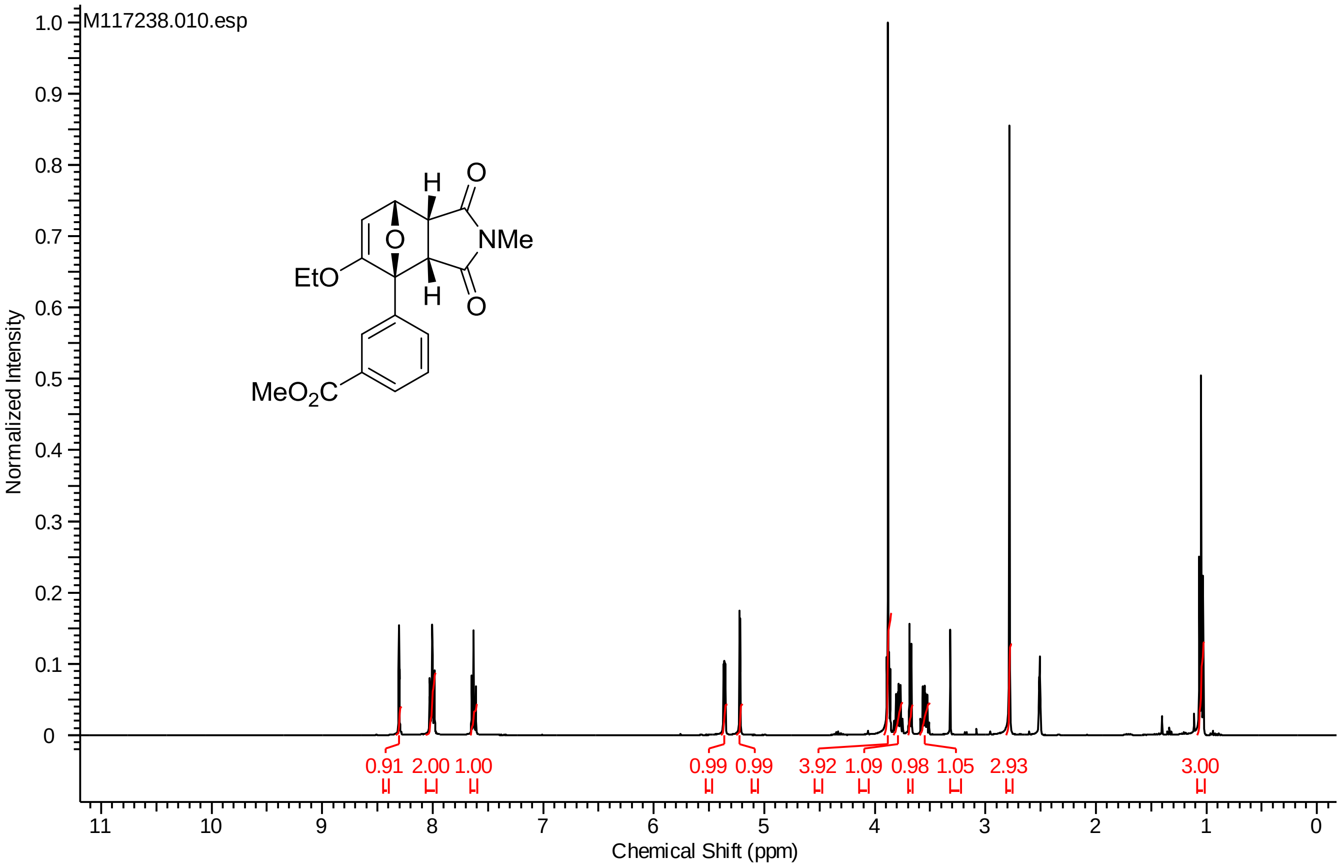

^{13}C NMR (100 MHz, DMSO-d$_6$)

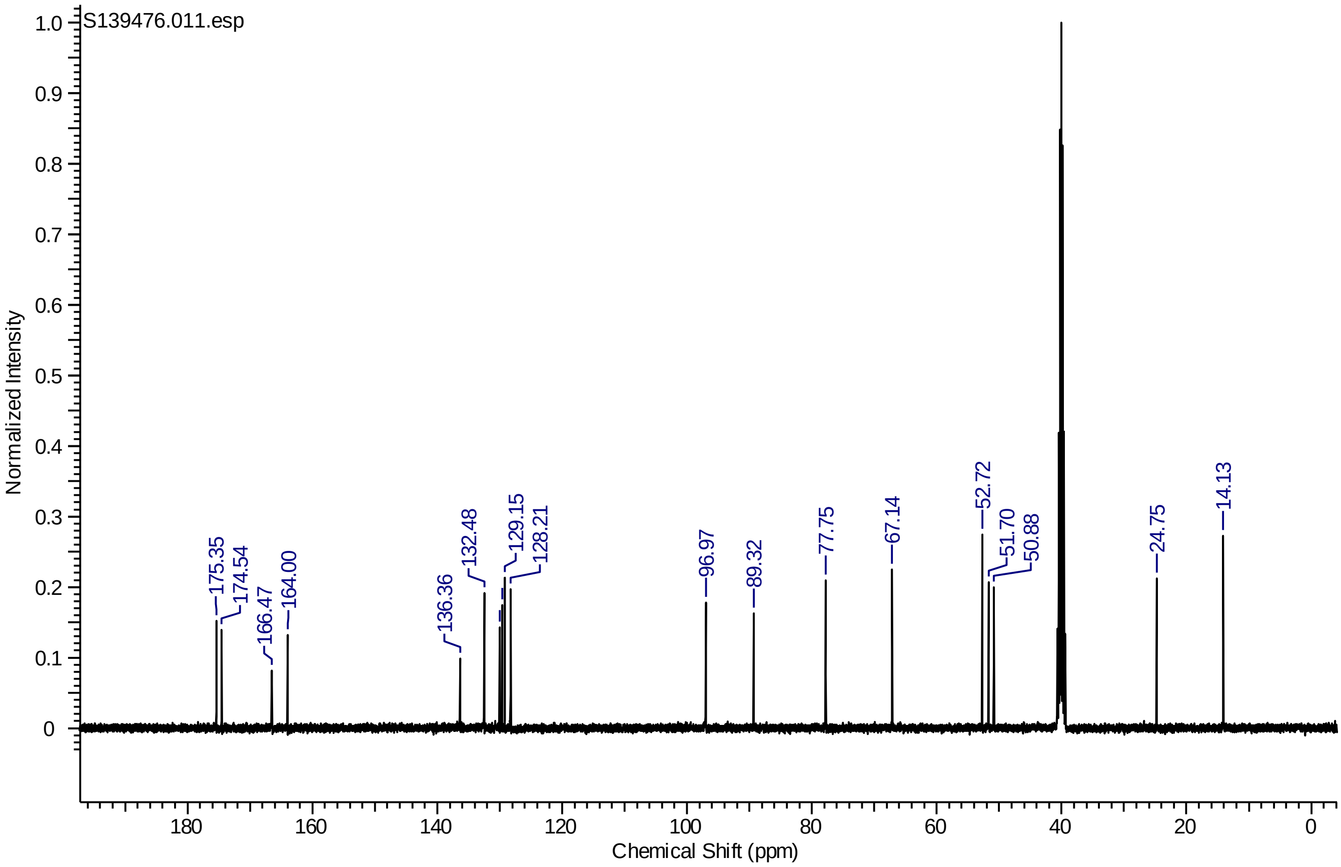

Methyl 3-((3a*R*,4*S*,7*R*,7a*S*)-5-ethoxy-2-methyl-1,3-dioxo-2,3,3a,4,7,7a-hexahydro-1*H*-4,7-epoxyisoindol-4-yl)benzoate *exo*-3j

^{1}H NMR (400 MHz, $CDCl_3$)

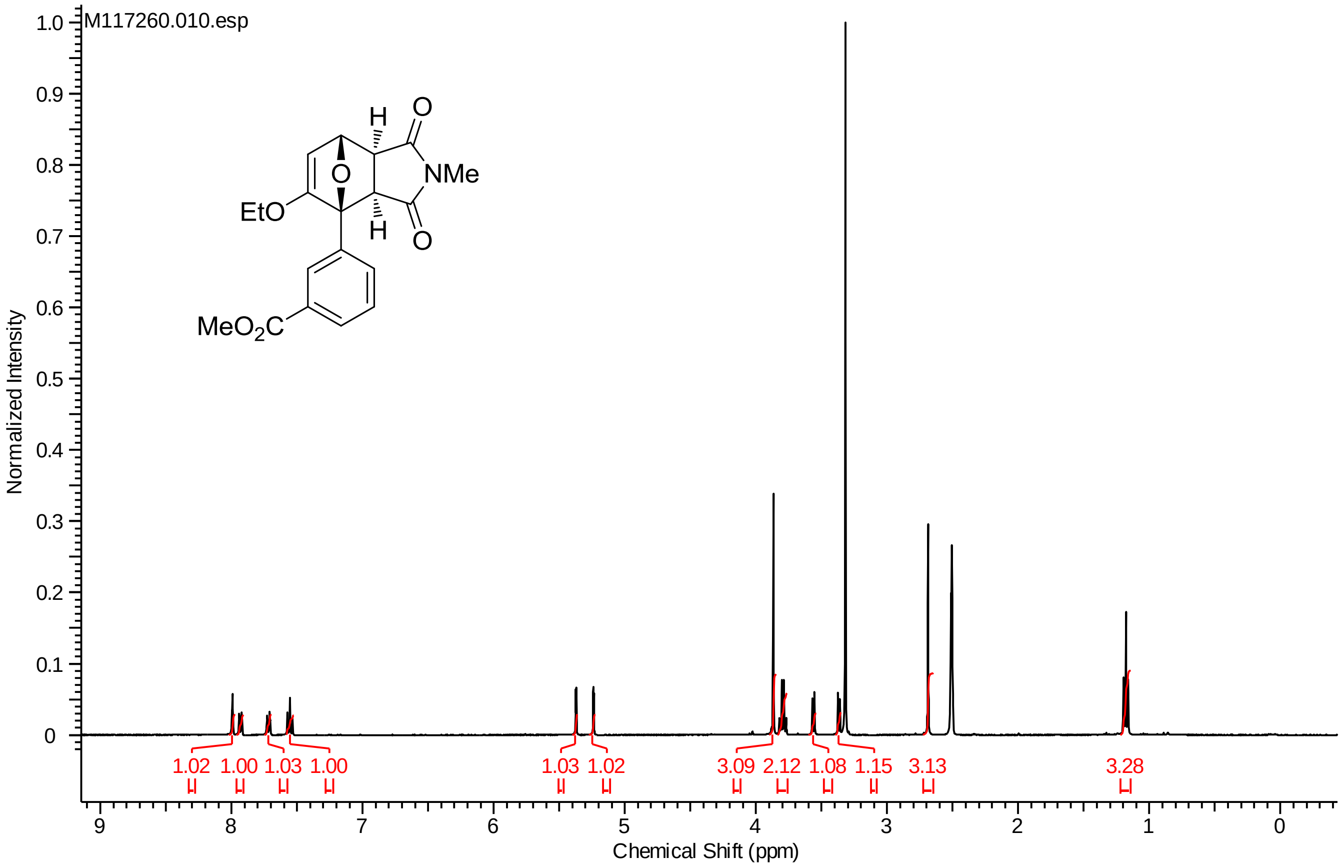

^{13}C NMR (100 MHz, $CDCl_3$)

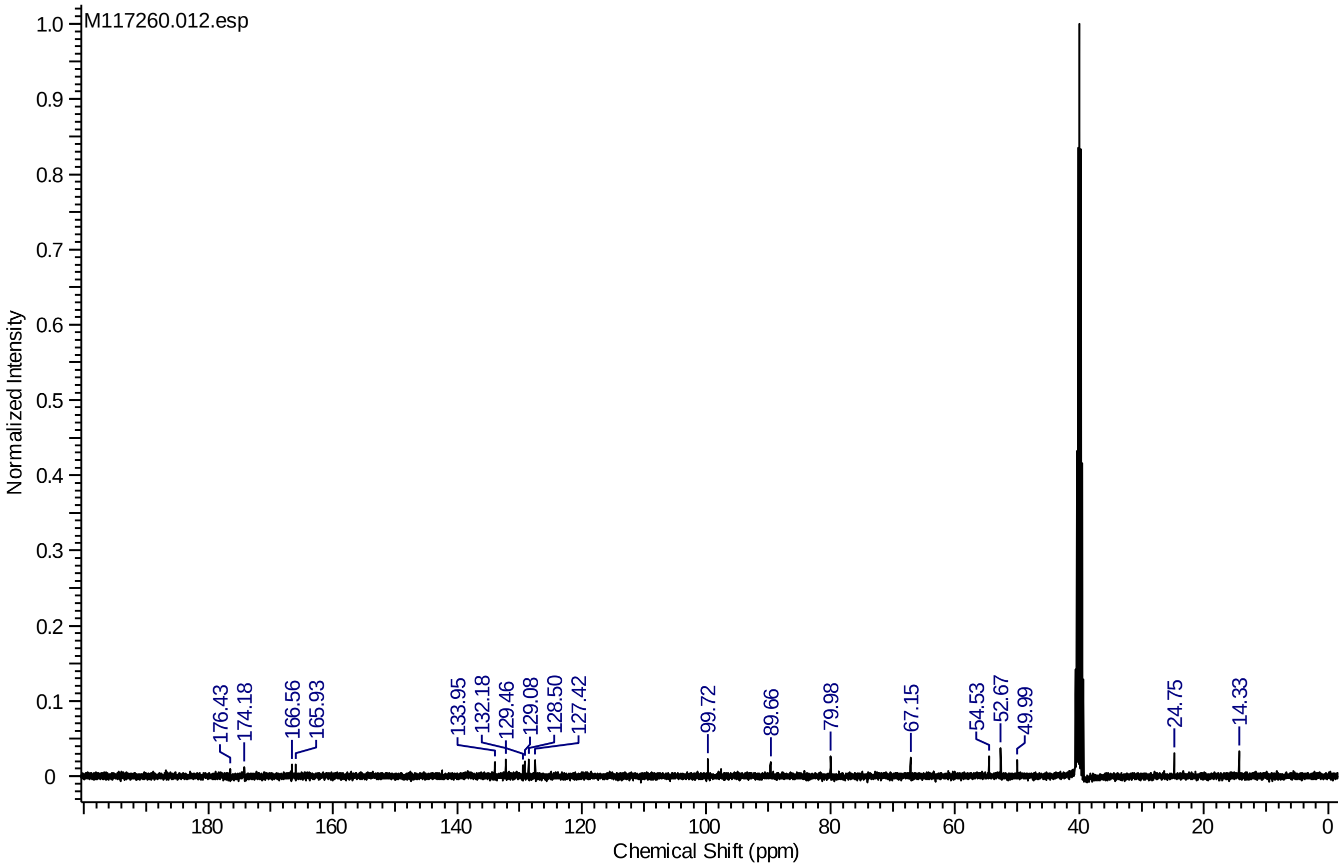

(3a*S*,4*S*,7*R*,7a*R*)-4-(*o*-Tolyl)-5-ethoxy-2-methyl-3a,4,7,7a-tetrahydro-1*H*-4,7-epoxyisoindole-1,3(2*H*)-dione *endo*-3k and (3a*R*,4*S*,7*R*,7a*S*)-4-(*o*-Tolyl)-5-ethoxy-2-methyl-3a,4,7,7a-tetrahydro-1*H*-4,7-epoxyisoindole-1,3(2*H*)-dione *exo*-3k

^{1}H NMR (400 MHz, DMSO-d_6)

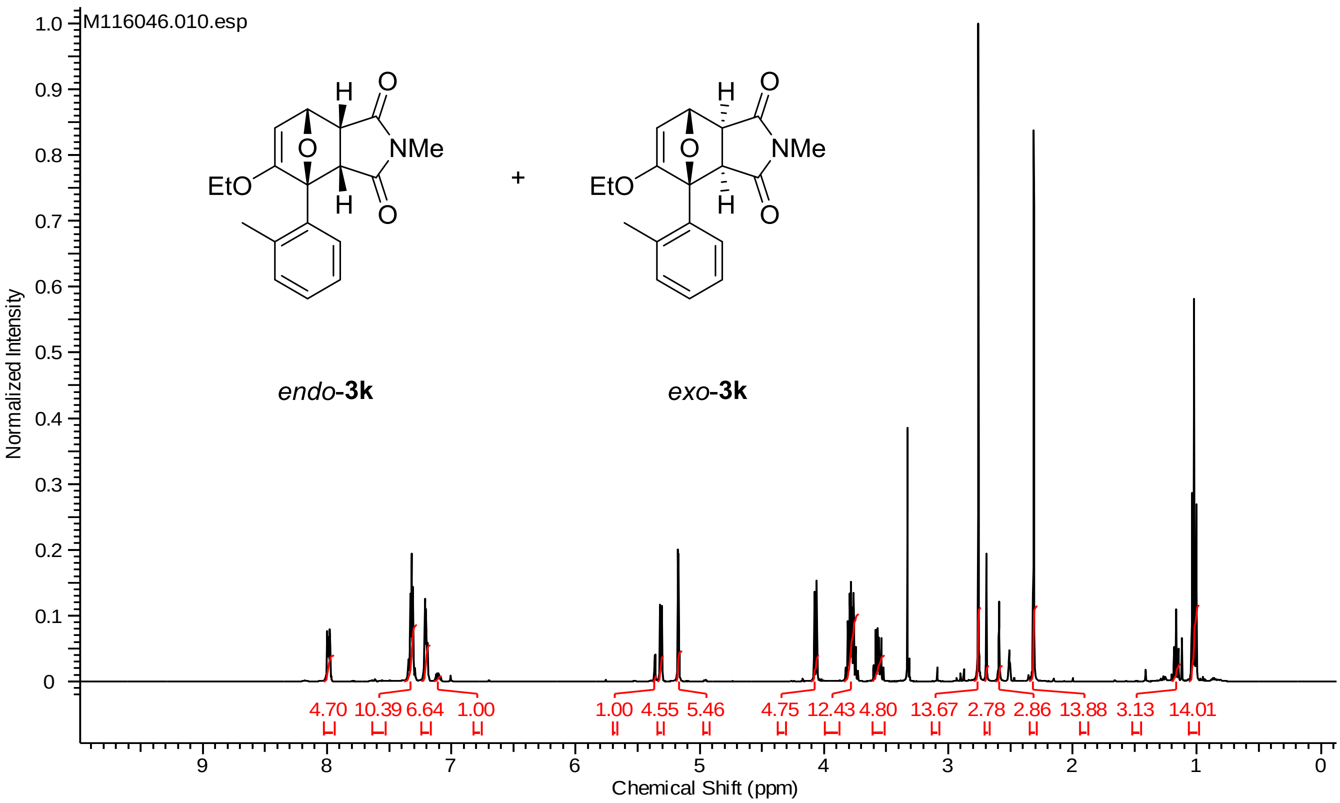

^{13}C NMR (100 MHz, $CDCl_3$)

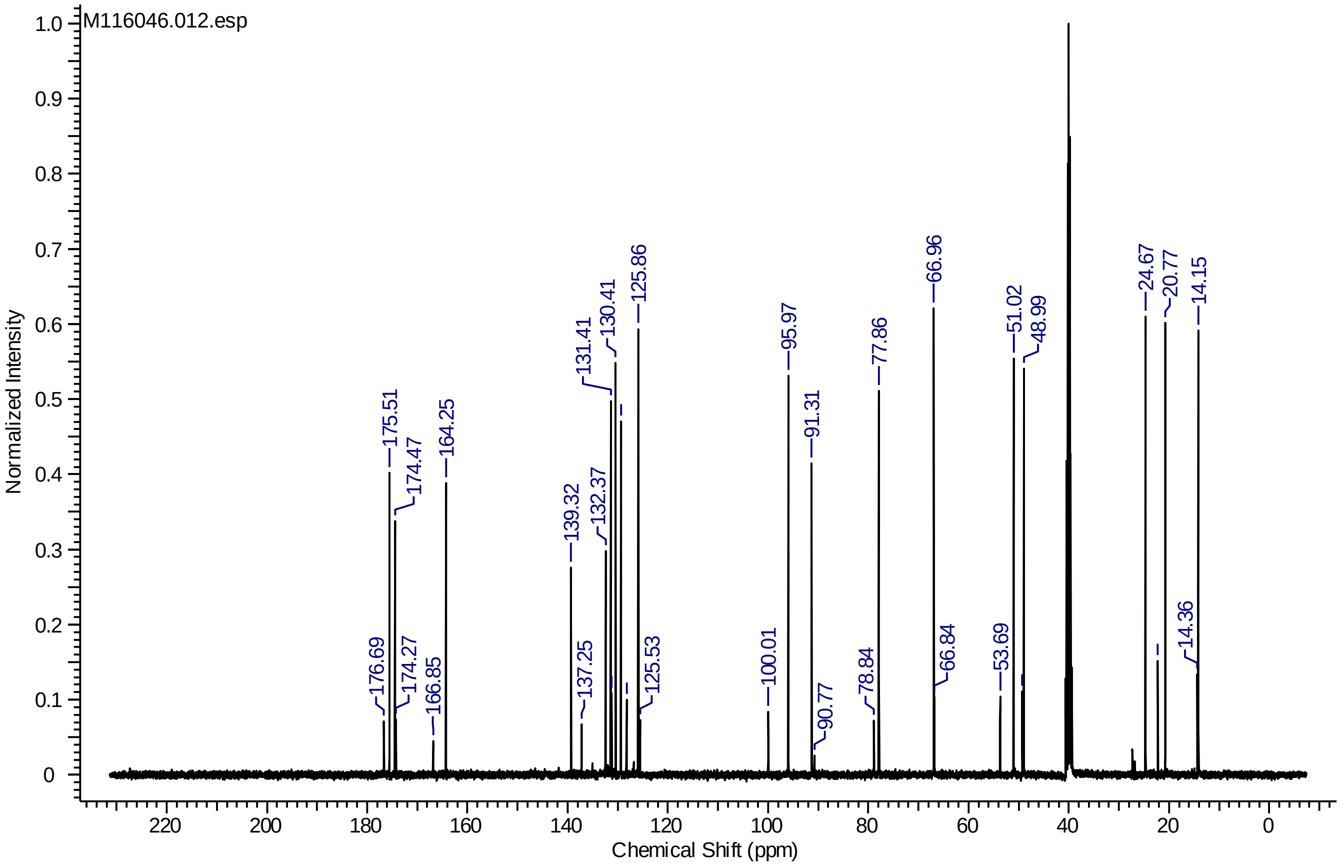

(3a*S*,4*S*,7*R*,7a*R*)-4-(Furan-2-yl)-5-ethoxy-2-methyl-3a,4,7,7a-tetrahydro-1*H*-4,7-epoxyisoindole-1,3(2*H*)-dione *endo*-3l and (3a*R*,4*S*,7*R*,7a*S*)-4-(Furan-2-yl)-5-ethoxy-2-methyl-3a,4,7,7a-tetrahydro-1*H*-4,7-epoxyisoindole-1,3(2*H*)-dione *exo*-3l

^{1}H NMR (400 MHz, DMSO-d$_6$)

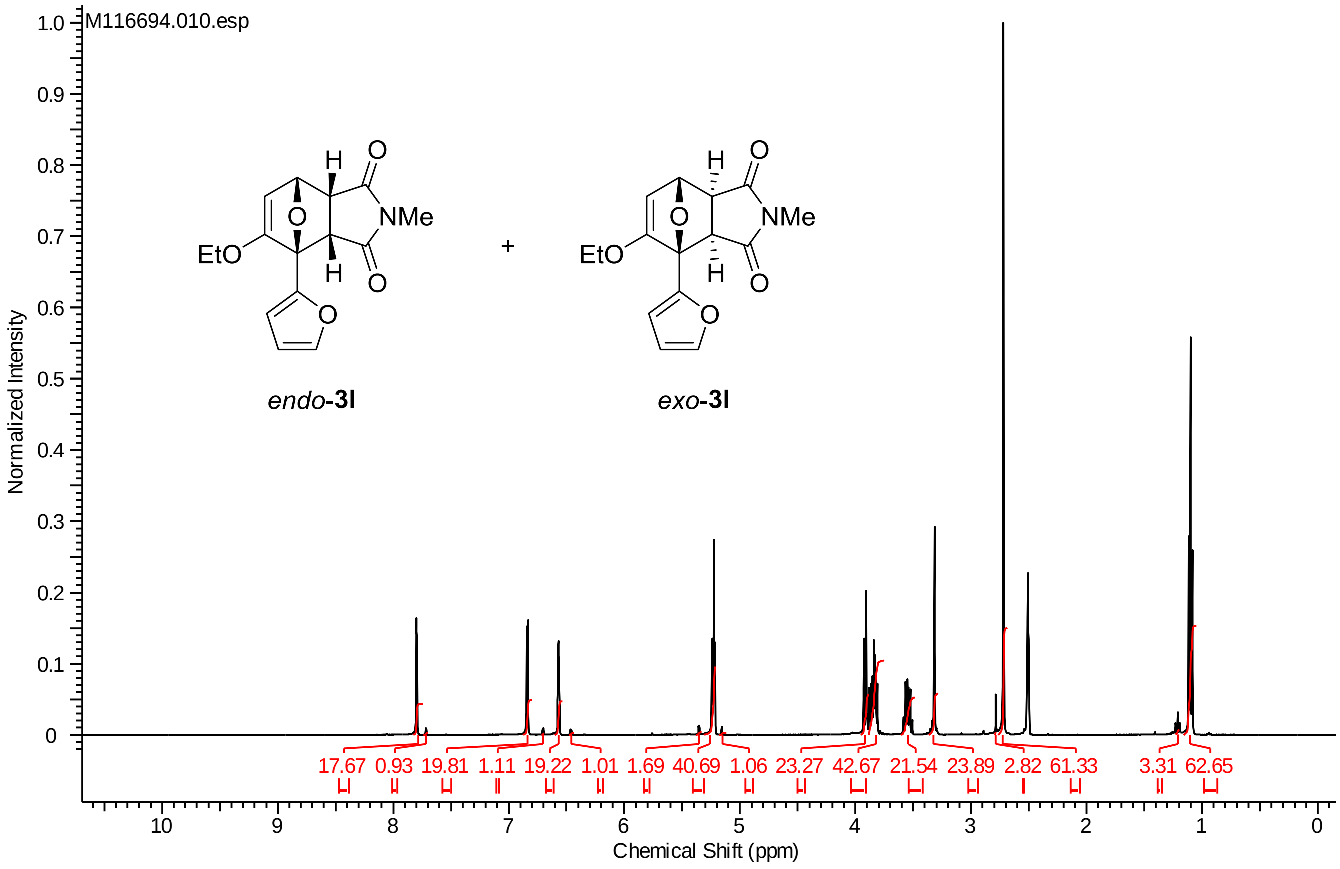

^{13}C NMR (100 MHz, DMSO-d$_6$)

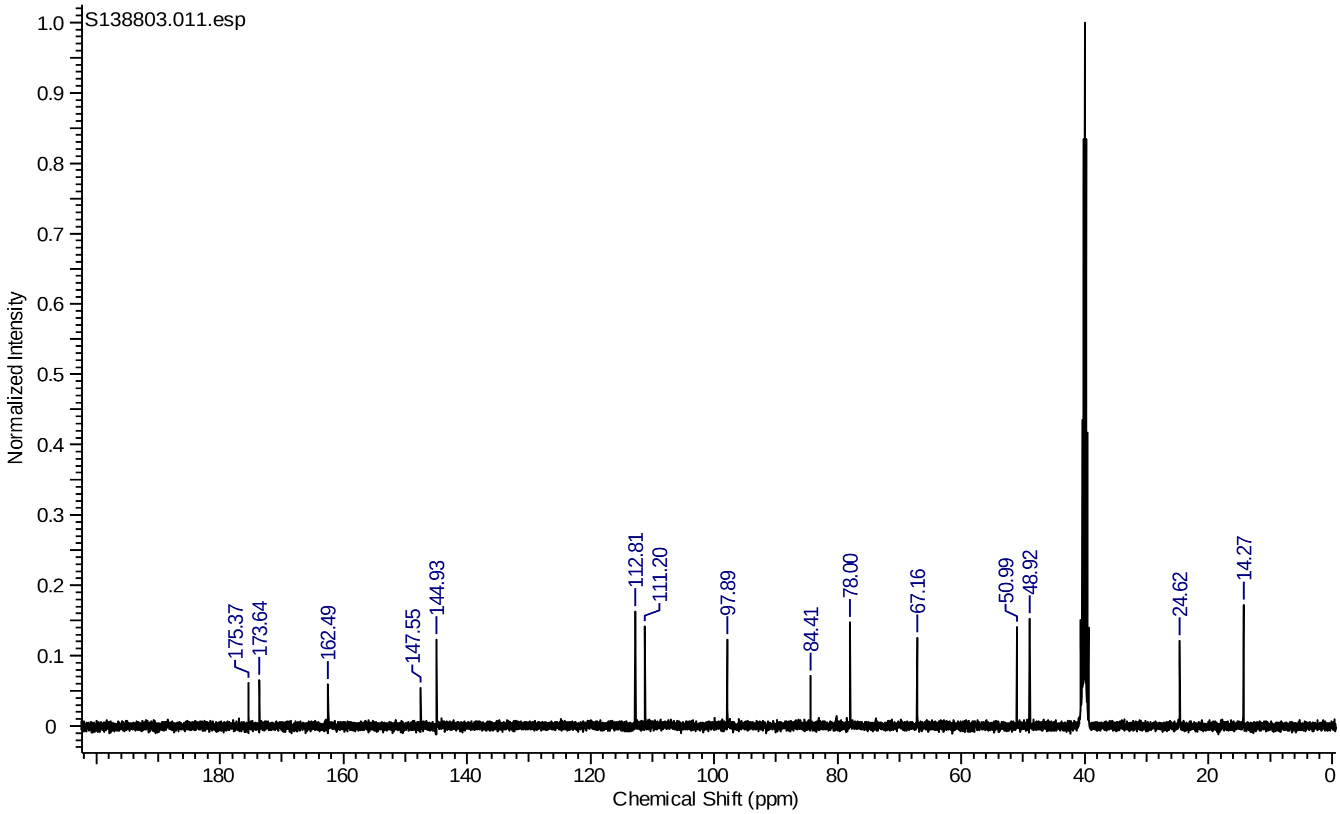

(3a*S*,4*R*,7*R*,7a*R*)-5-Ethoxy-2-methyl-4-(thiophen-2-yl)-3a,4,7,7a-tetrahydro-1*H*-4,7-epoxyisoindole-1,3(2*H*)-dione e*ndo*-3m and (3a*R*,4*R*,7*R*,7a*S*)-5-Ethoxy-2-methyl-4-(thiophen-2-yl)-3a,4,7,7a-tetrahydro-1*H*-4,7-epoxyisoindole-1,3(2*H*)-dione e*xo*-3m

^{1}H NMR (400 MHz, DMSO-d$_6$)

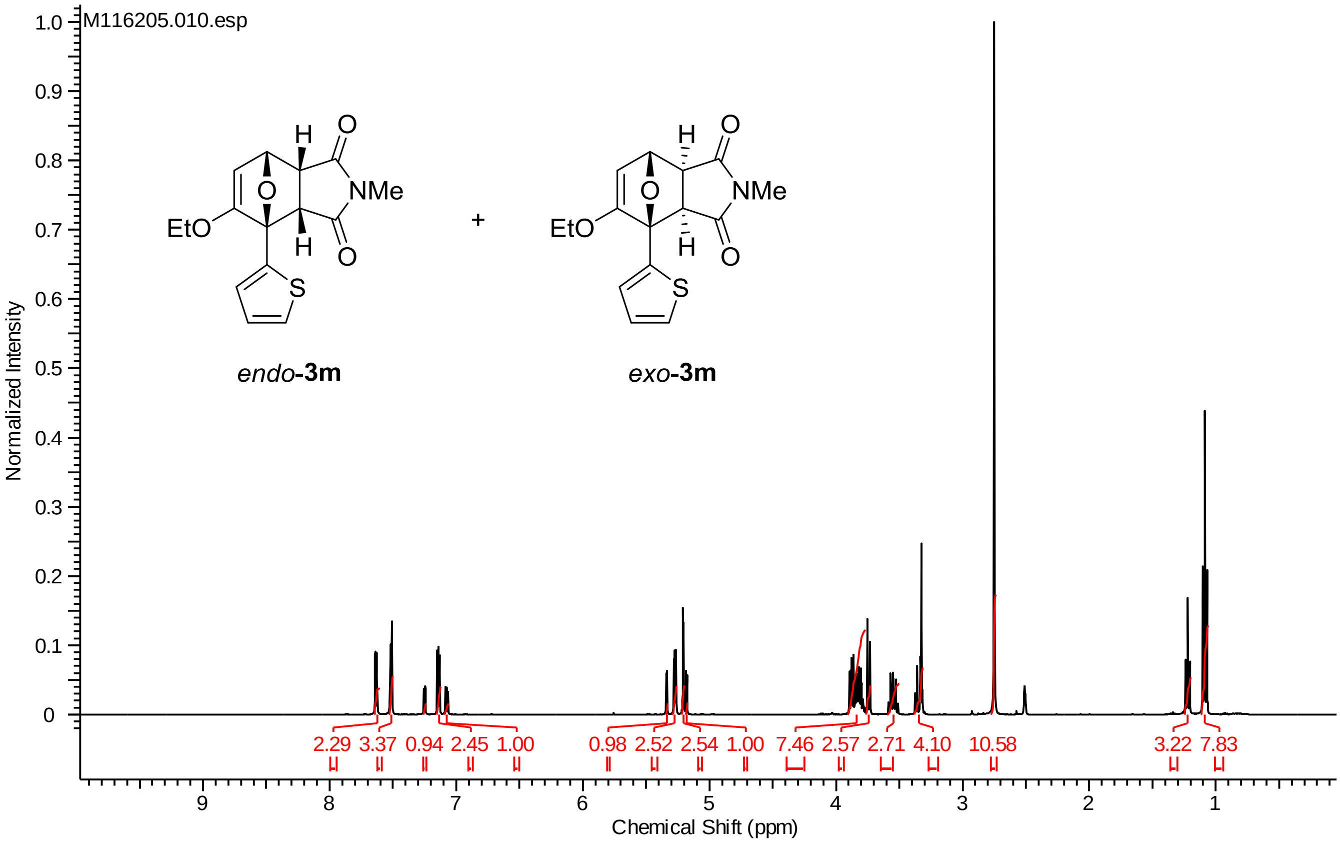

^{13}C NMR (100 MHz, DMSO-d$_6$)

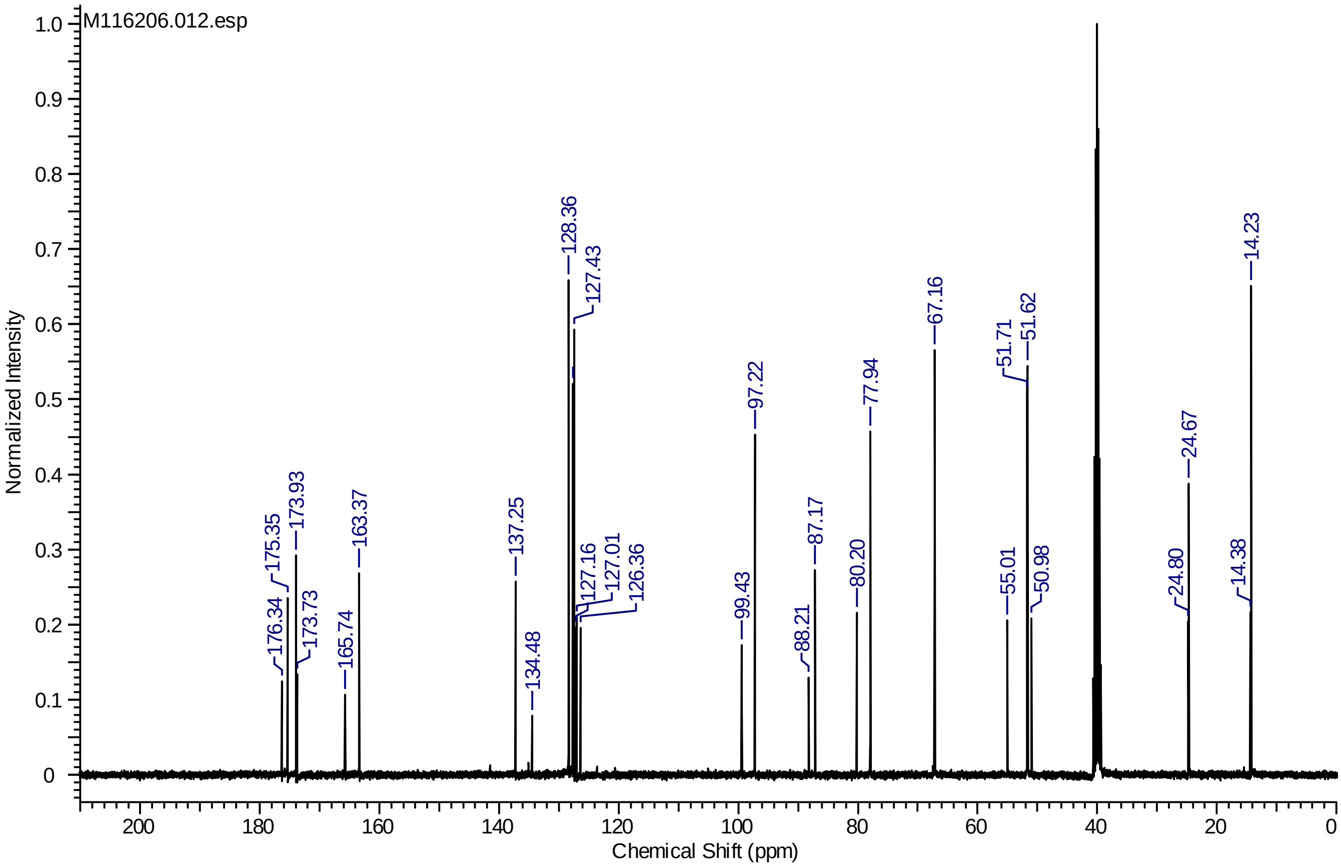

(3a*S*,4*S*,7*R*,7a*R*)-5-Ethoxy-2-methyl-4-(pyridin-3-yl)-3a,4,7,7a-tetrahydro-1*H*-4,7-epoxyisoindole-1,3(2*H*)-dione *endo*-3n and (3a*R*,4*S*,7*R*,7a*S*)-5-Ethoxy-2-methyl-4-(pyridin-3-yl)-3a,4,7,7a-tetrahydro-1*H*-4,7-epoxyisoindole-1,3(2*H*)-dione *exo*-3n

^{1}H NMR (400 MHz, MeOH-d$_4$)

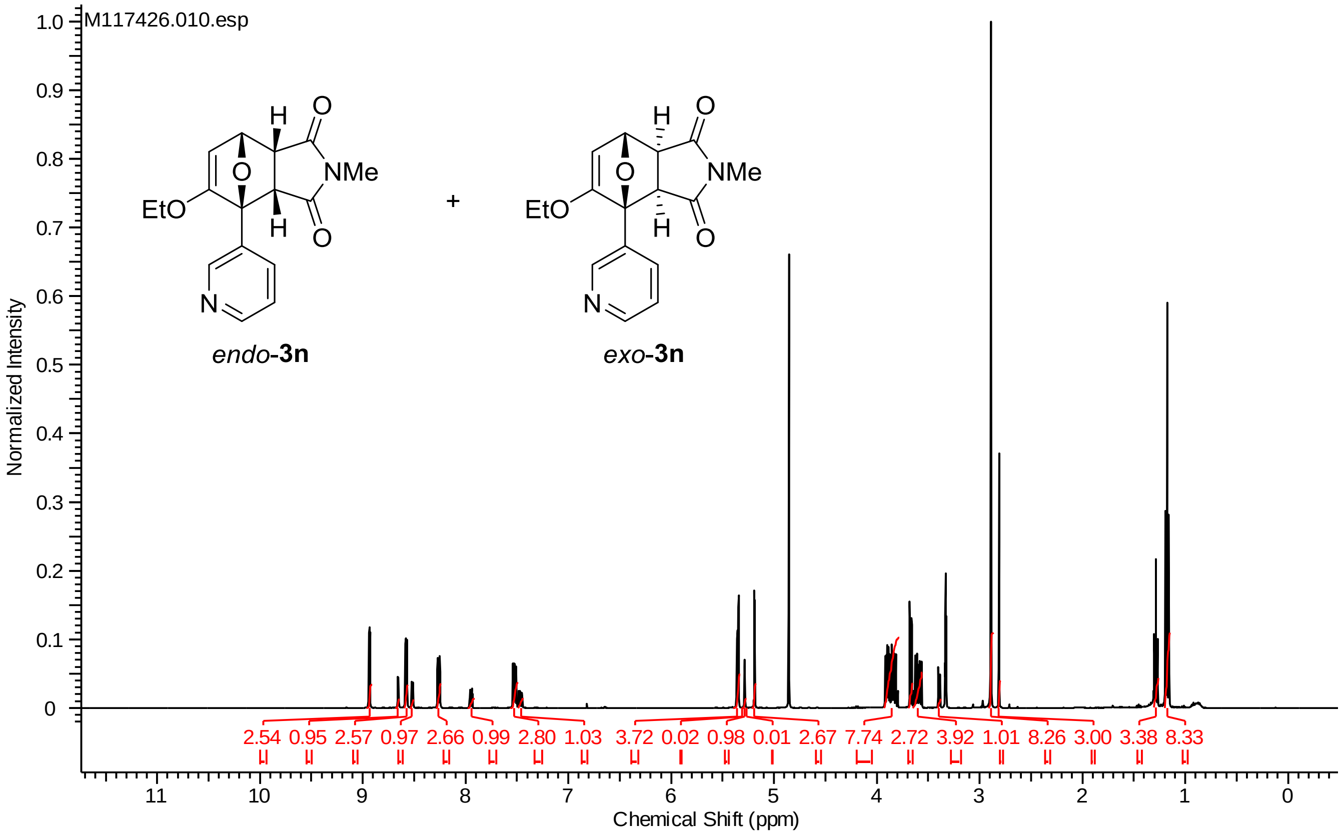

^{13}C NMR (100 MHz, MeOH-d$_4$)

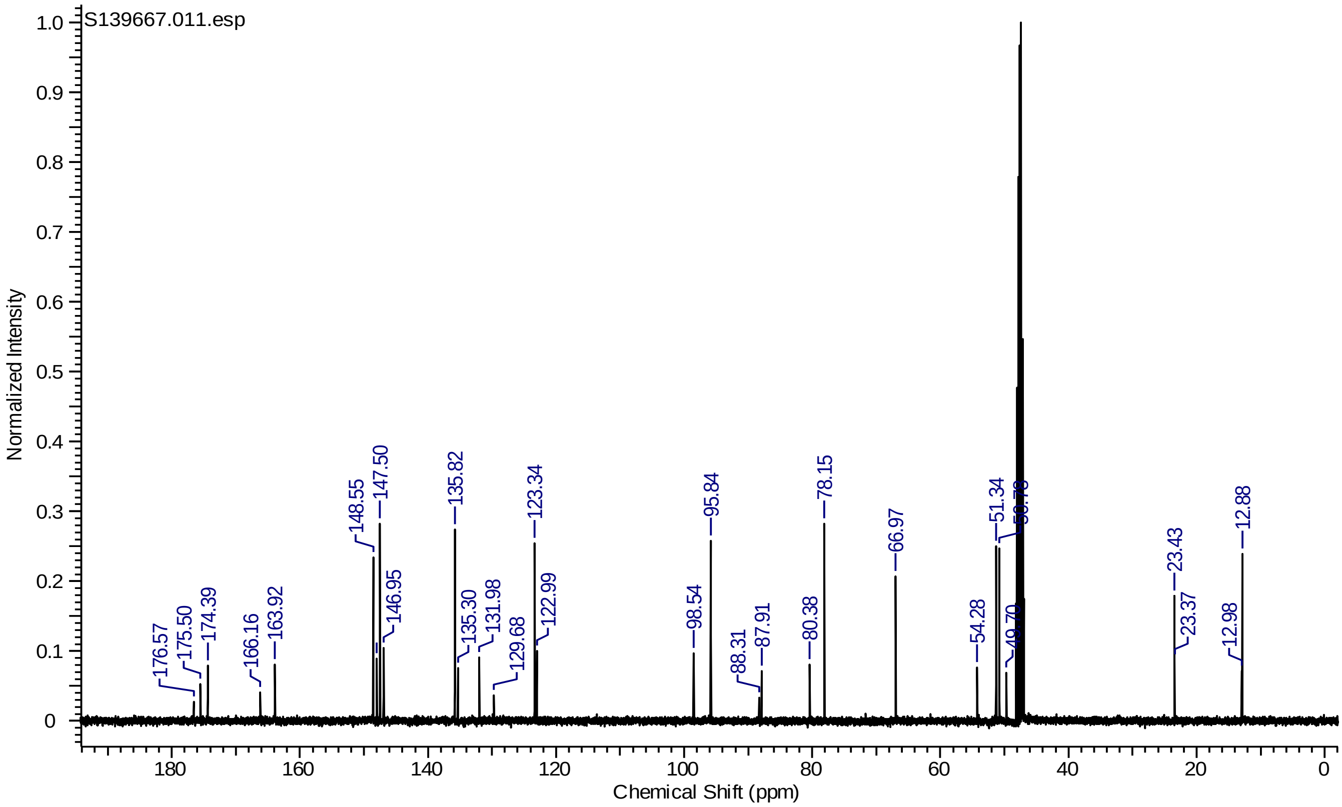

(3a*S*,4*R*,7*R*,7a*R*)-7-((Dimethylamino)methyl)-5-ethoxy-2-methyl-4-phenethyl-3a,4,7,7a-tetrahydro-1*H*-4,7-epoxyisoindole-1,3(2*H*)-dione *endo*-3o and (3a*R*,4*R*,7*R*,7a*S*)-7-((Dimethylamino)methyl)-5-ethoxy-2-methyl-4-phenethyl-3a,4,7,7a-tetrahydro-1*H*-4,7-epoxyisoindole-1,3(2*H*)-dione *exo*-3o

^{1}H NMR (600 MHz, MeOH-d$_4$)

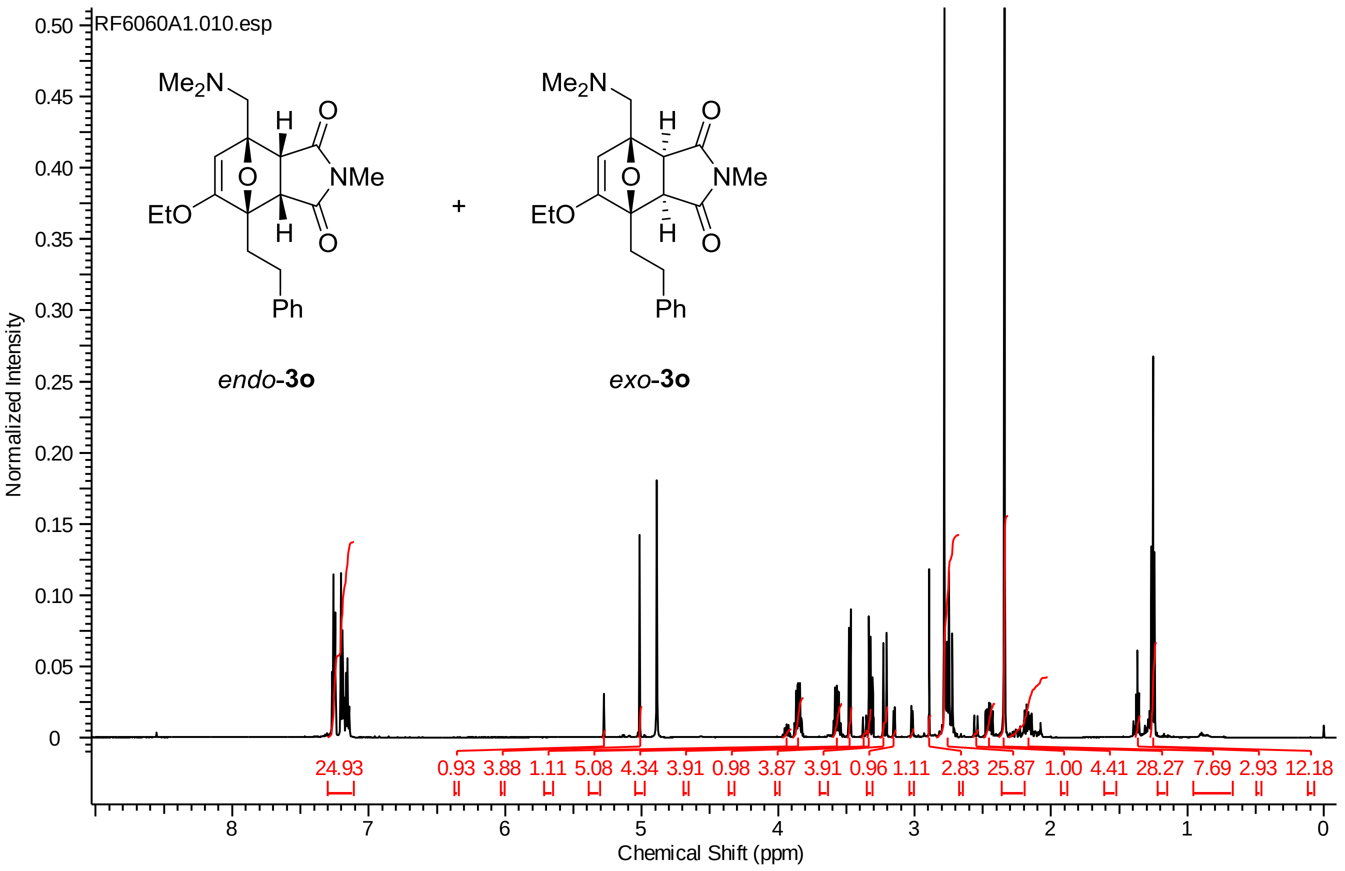

^{13}C NMR (1500 MHz, MeOH-d$_4$)

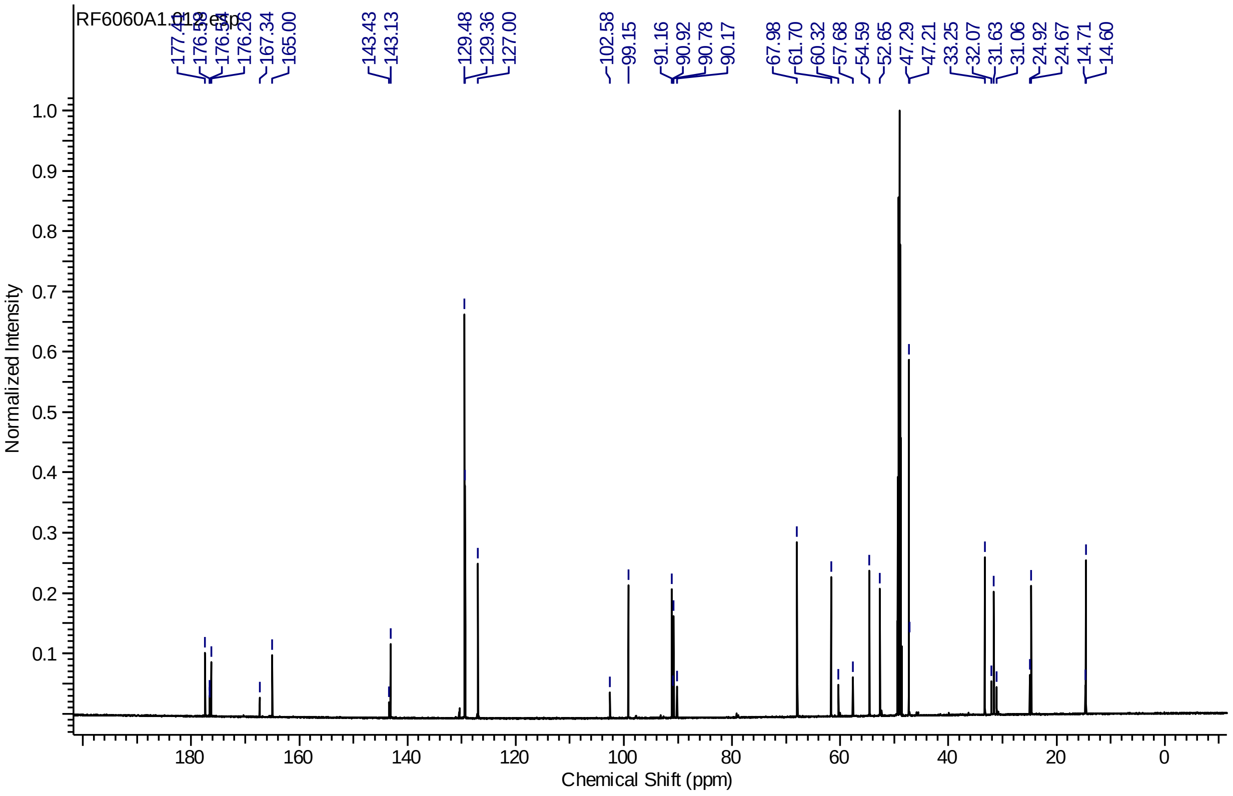

1-(4-Methylbenzyl)-1*H*-pyrrole-2,5-dione 4b

^{1}H NMR (400 MHz, DMSO-d_6)

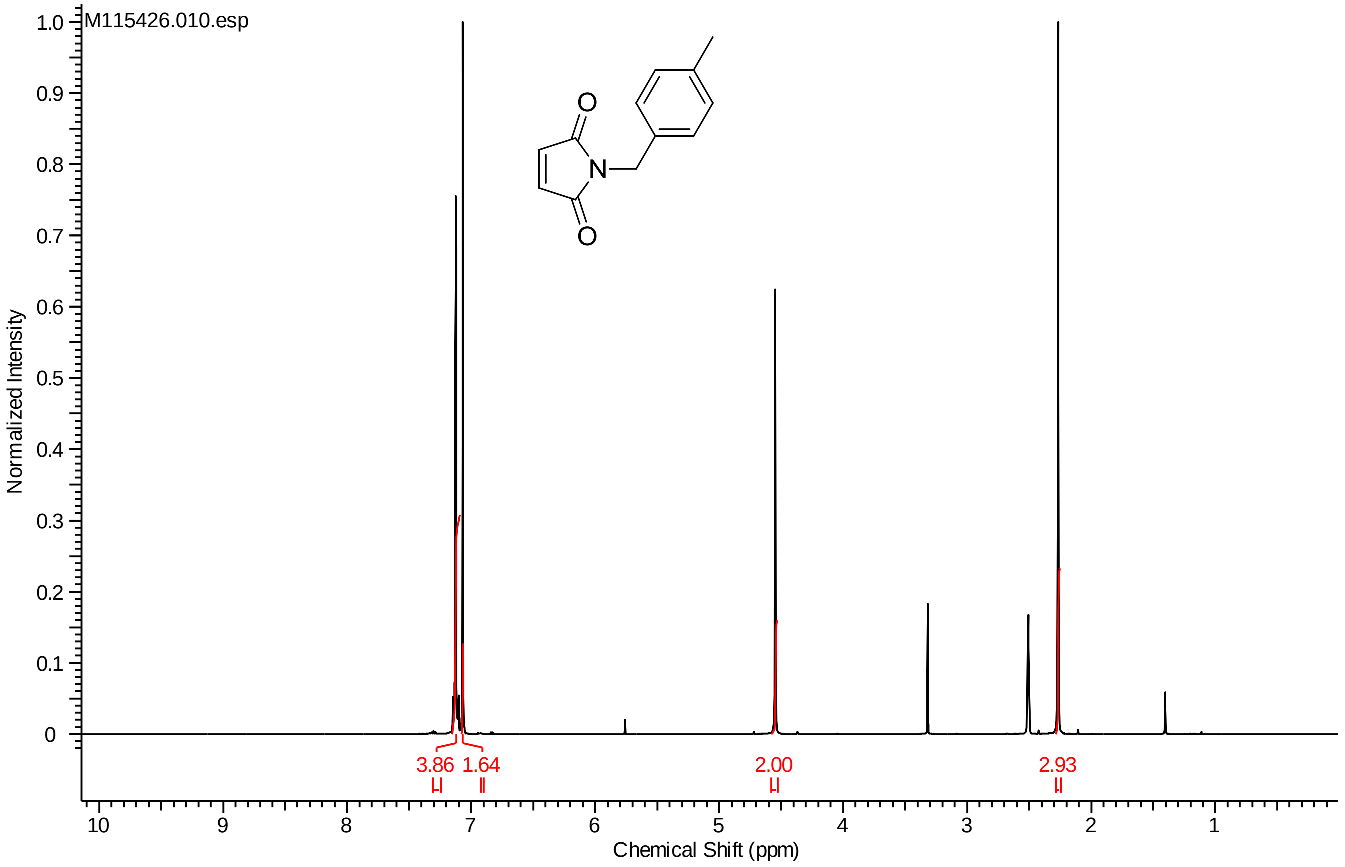

^{13}C NMR (100 MHz, DMSO-d_6)

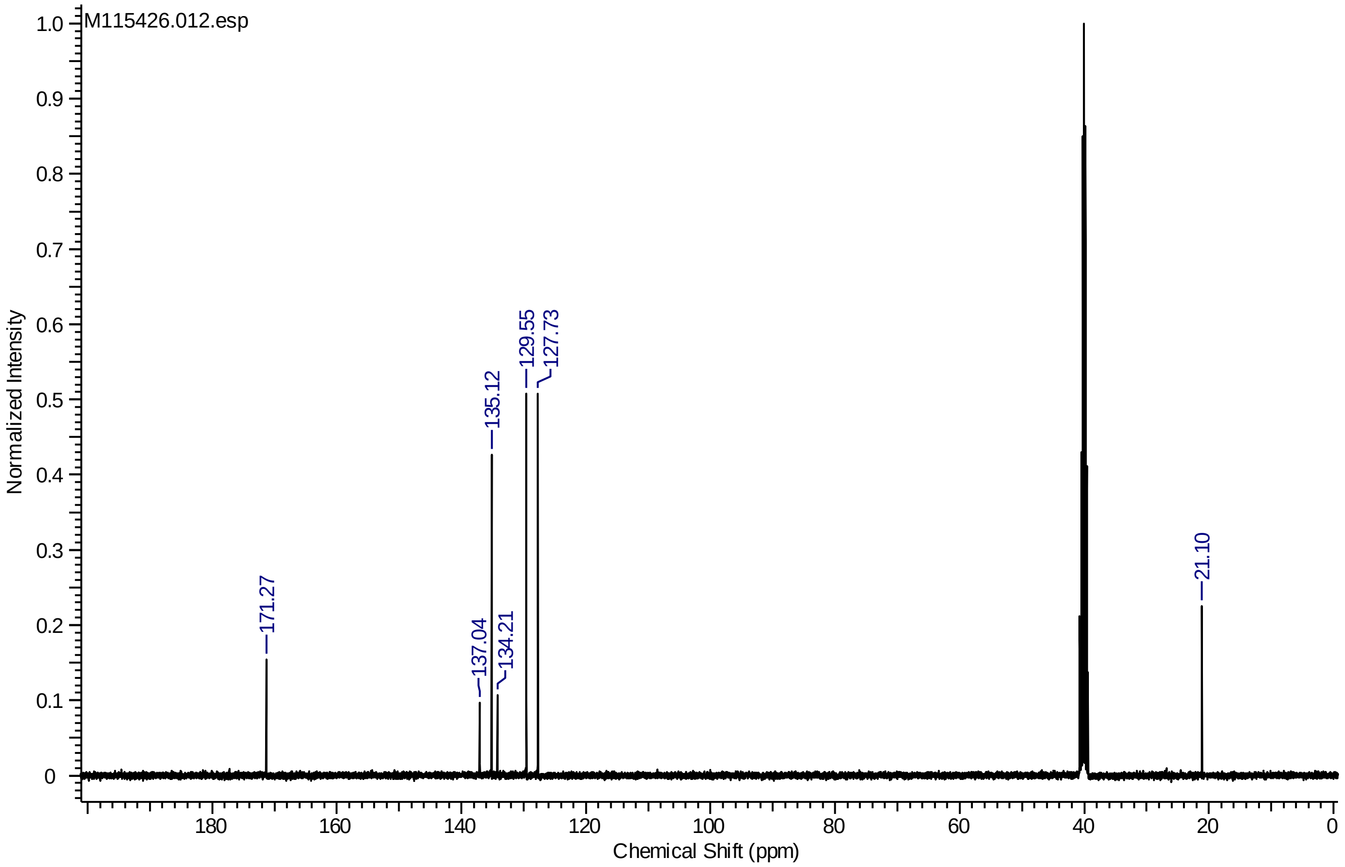

1-Cyclopropyl-1*H*-pyrrole-2,5-dione 4c

^{1}H NMR (400 MHz, DMSO-d_6)

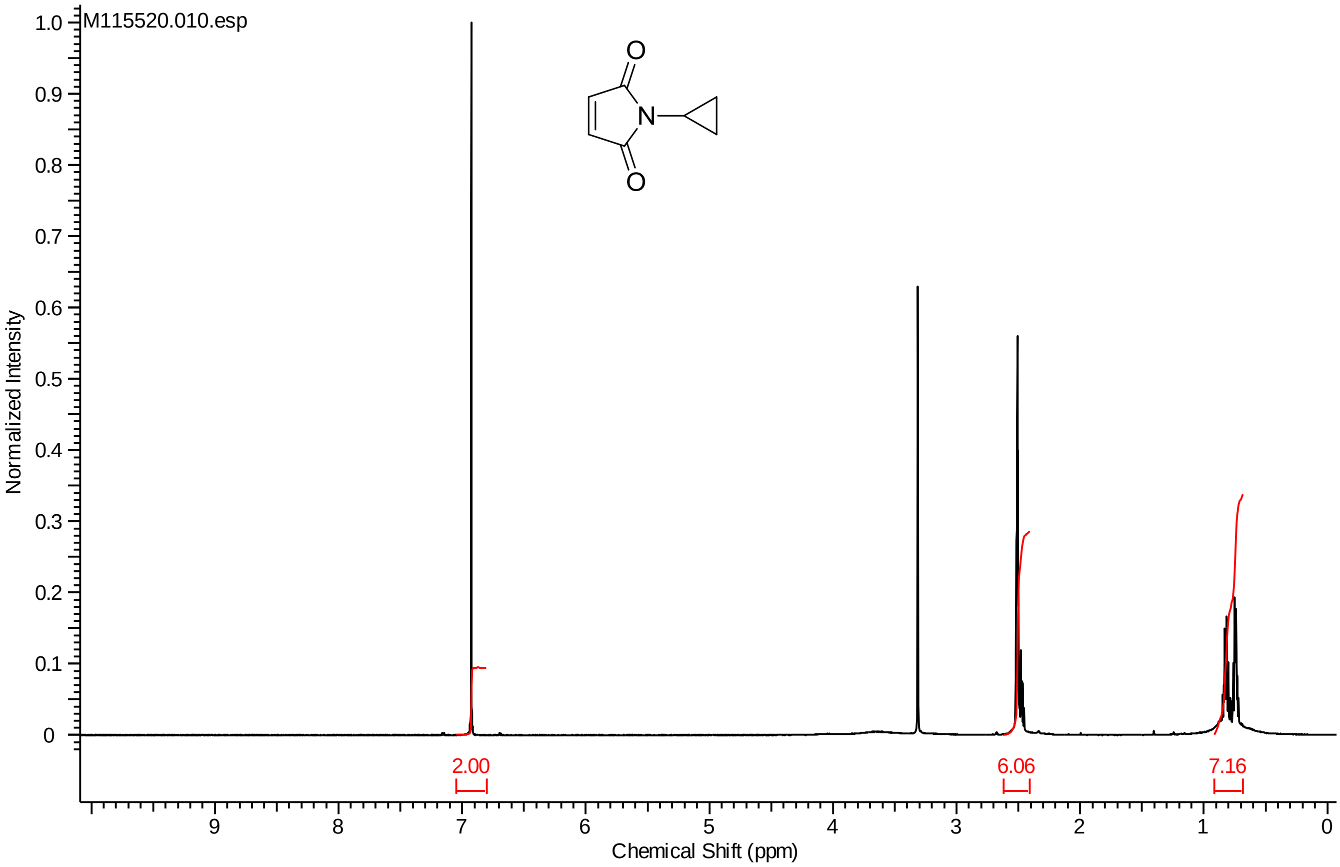

^{13}C NMR (100 MHz, DMSO-d_6)

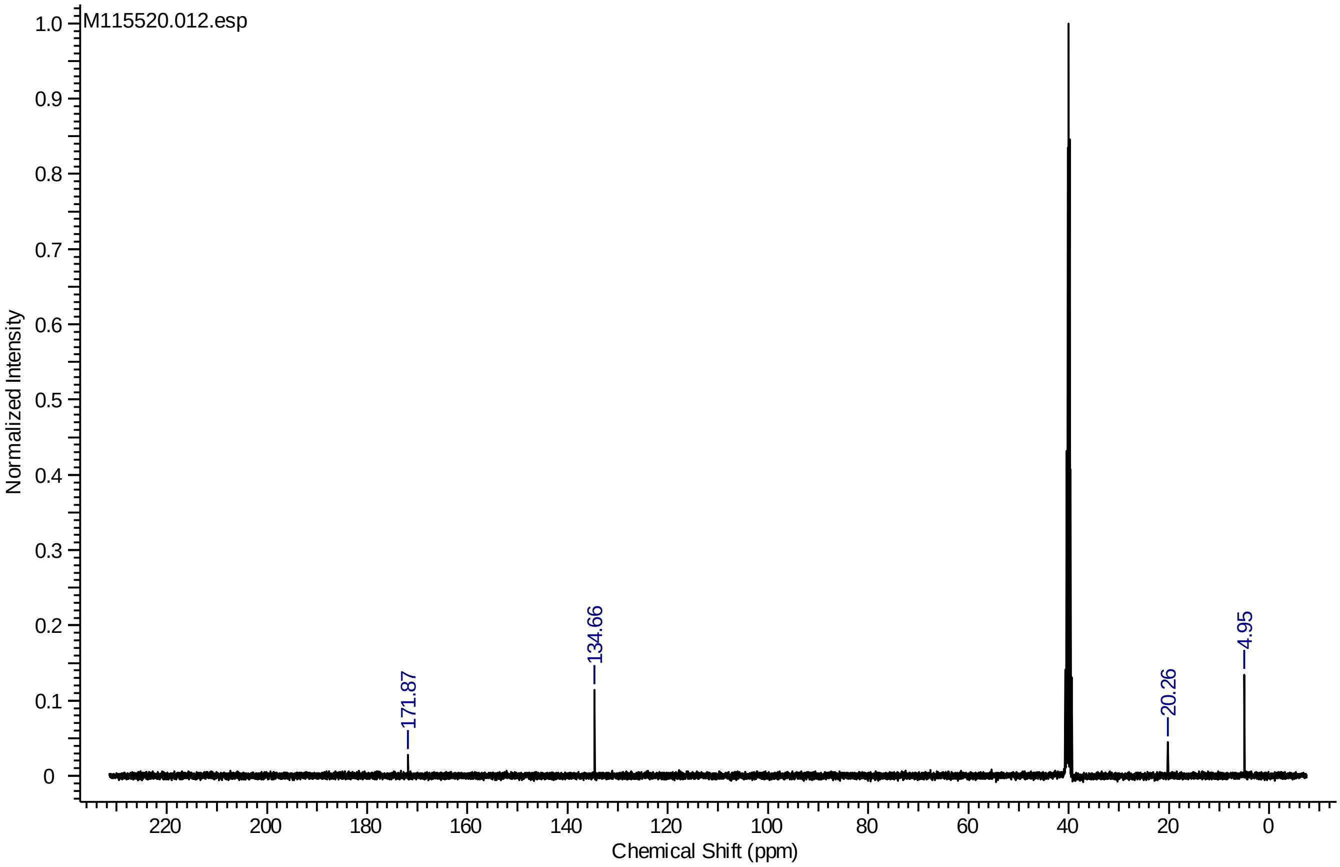

(3a*S*,4*R*,7*R*,7a*R*)-5-Ethoxy-2-phenyl-4-phenethyl-3a,4,7,7a-tetrahydro-1*H*-4,7-epoxyisoindole-1,3(2*H*)-dione *endo*-5a and (3a*R*,4*R*,7*R*,7a*S*)-5-Ethoxy-2-phenyl-4-phenethyl-3a,4,7,7a-tetrahydro-1*H*-4,7-epoxyisoindole-1,3(2*H*)-dione *exo*-5a

^{1}H NMR (400 MHz, $CDCl_3$)

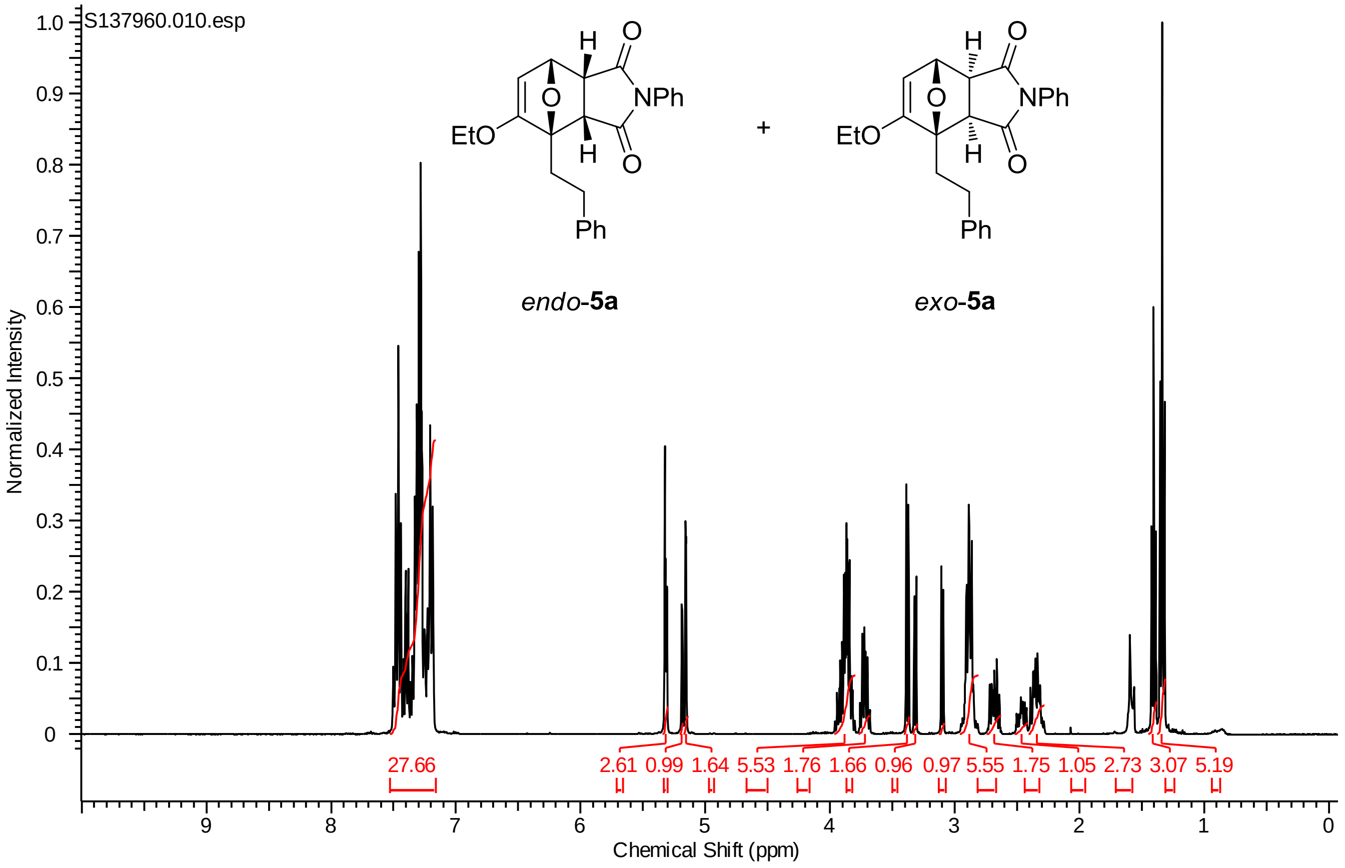

^{13}C NMR (100 MHz, $CDCl_3$)

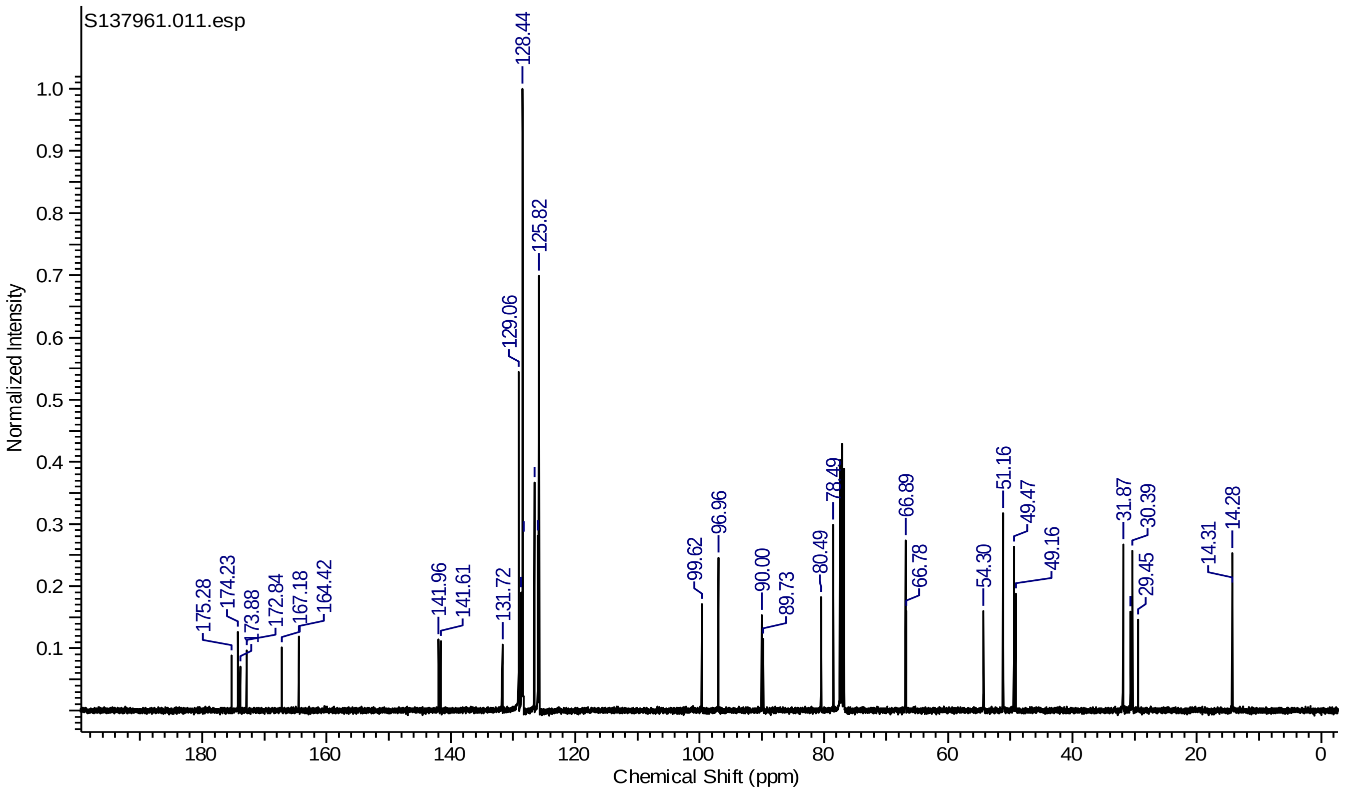

(3a*S*,4*R*,7*R*,7a*R*)-5-Ethoxy-2-(4-methylbenzyl)-4-phenethyl-3a,4,7,7a-tetrahydro-1*H*-4,7-epoxyisoindole-1,3(2*H*)-dione *endo*-5b and (3a*R*,4*R*,7*R*,7a*S*)-5-Ethoxy-2-(4-methylbenzyl)-4-phenethyl-3a,4,7,7a-tetrahydro-1*H*-4,7-epoxyisoindole-1,3(2*H*)-dione *exo*-5b

^{1}H NMR (400 MHz, MeOH-d$_4$)

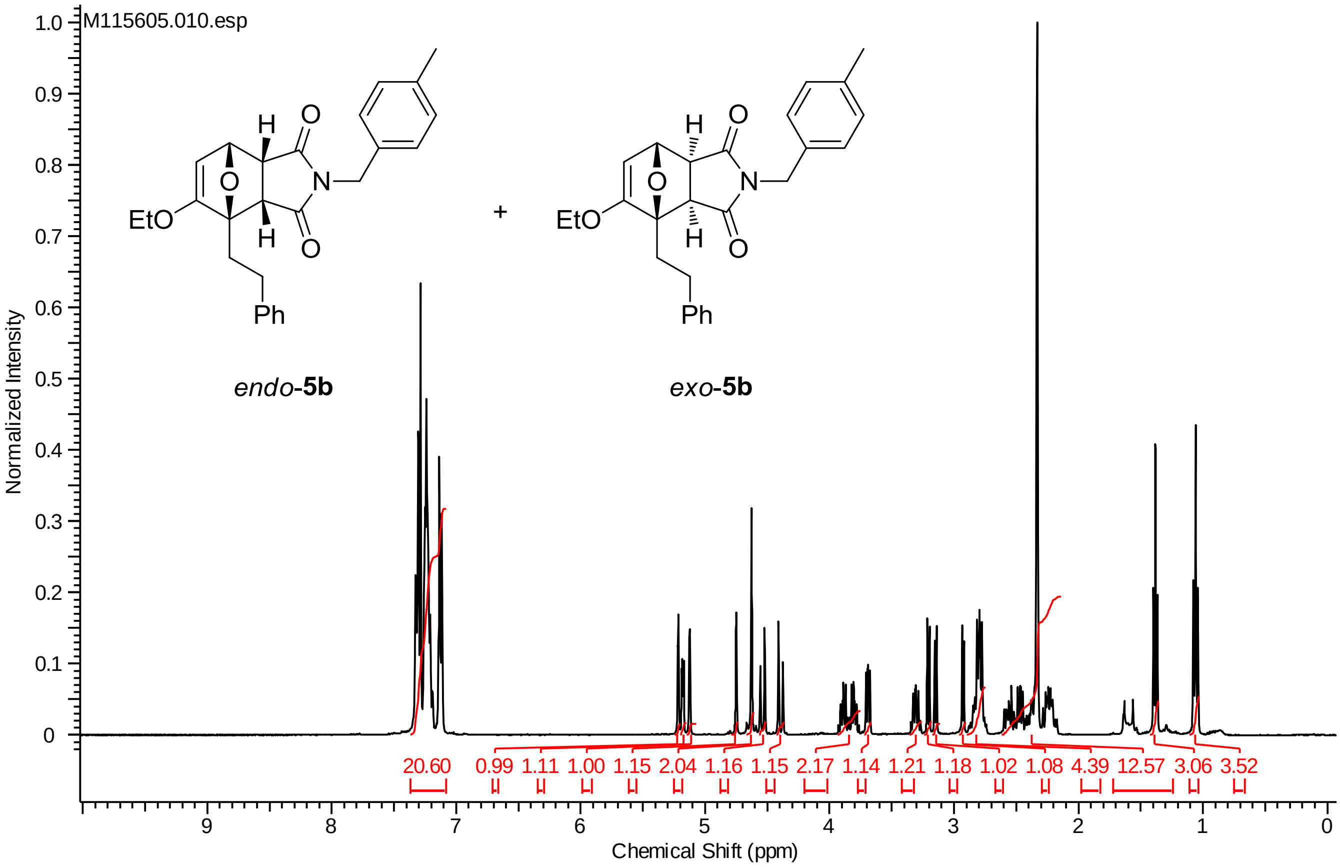

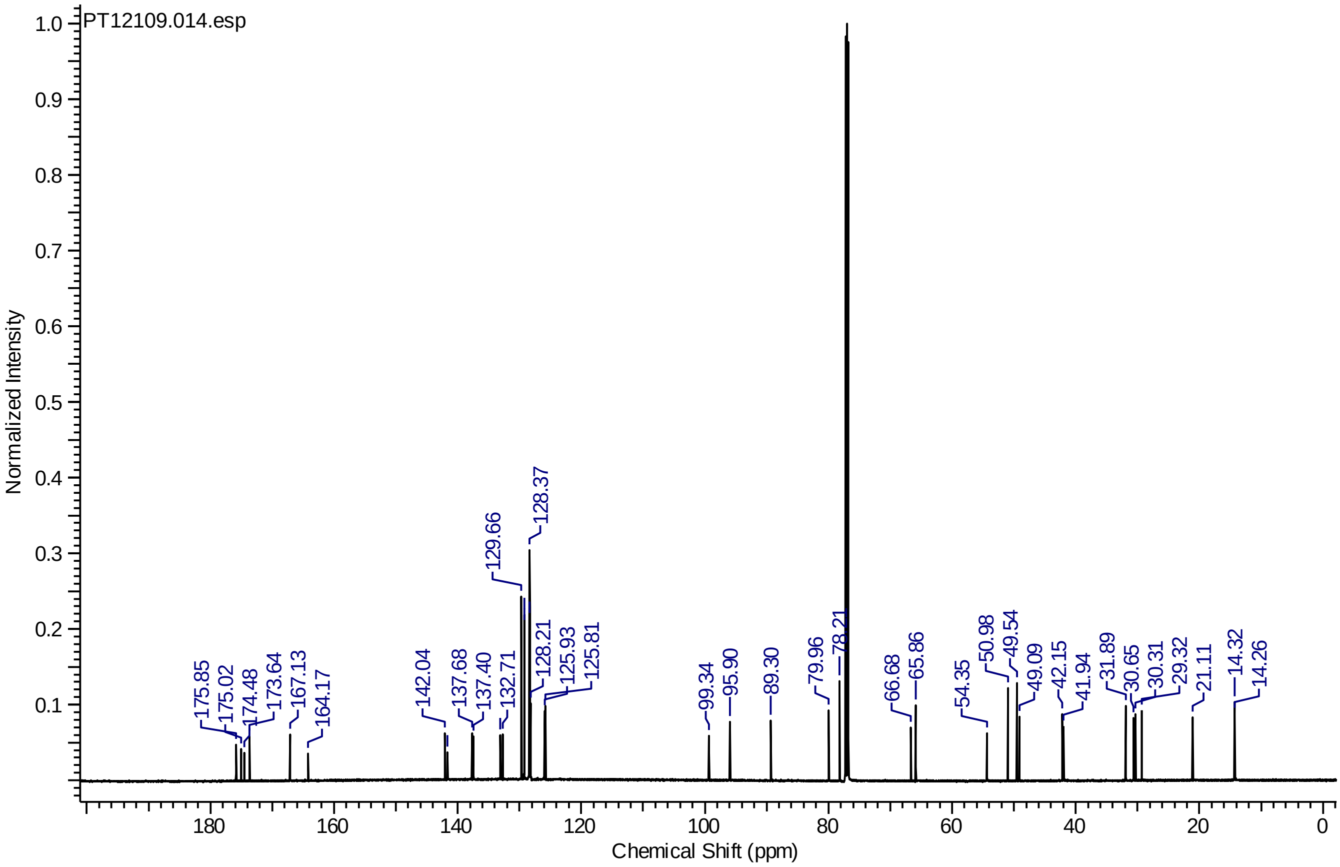

(3a*S*,4*R*,7*R*,7a*R*)-2-Cyclopropyl-5-ethoxy-4-phenethyl-3a,4,7,7a-tetrahydro-1*H*-4,7-epoxyisoindole-1,3(2*H*)-dione *endo*-5c and (3a*R*,4*R*,7*R*,7a*S*)-2-Cyclopropyl-5-ethoxy -4-phenethyl-3a,4,7,7a-tetrahydro-1*H*-4,7-epoxyisoindole-1,3(2*H*)-dione *exo*-5c

^{1}H NMR (400 MHz, $CDCl_3$)

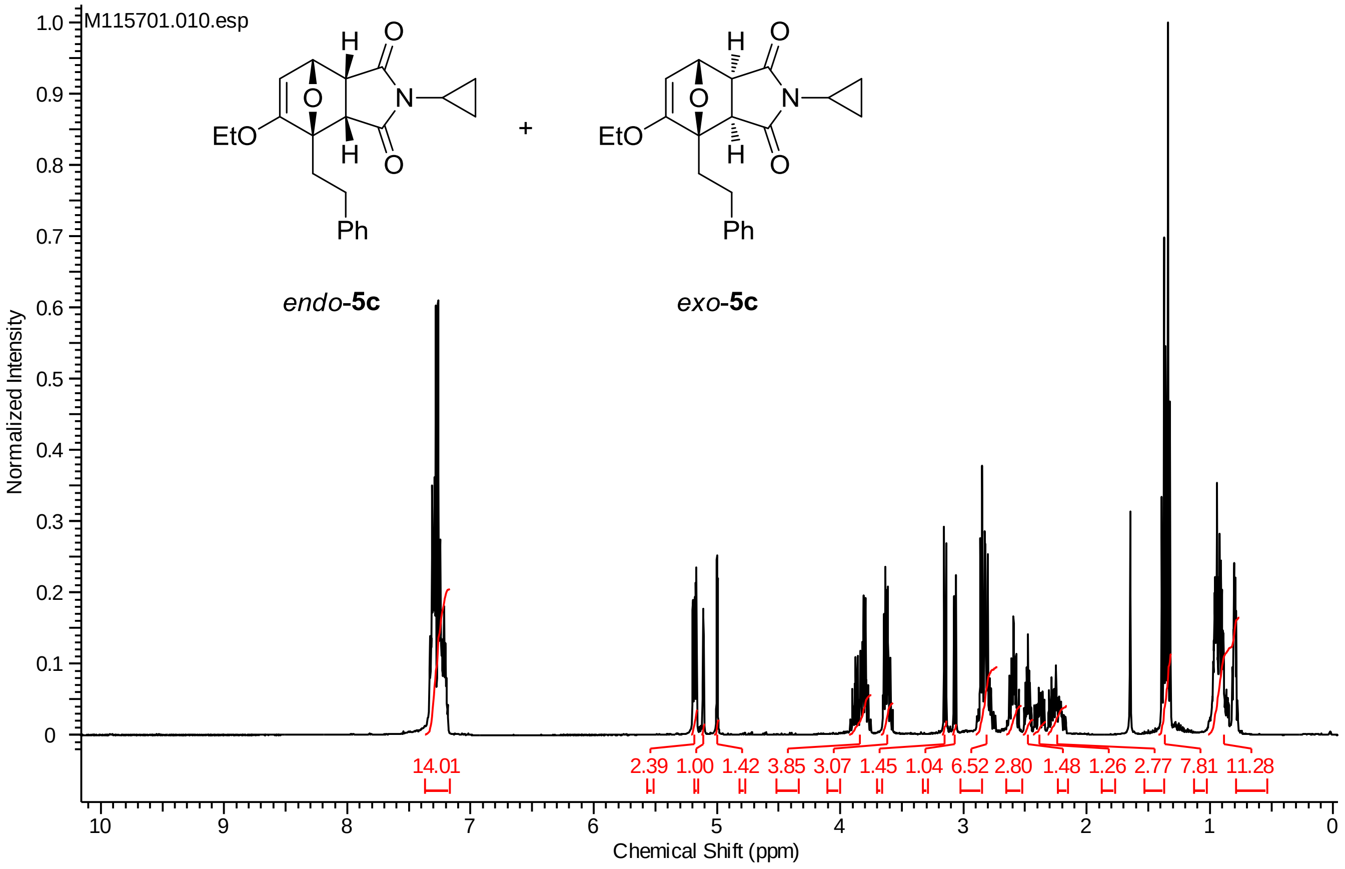

^{13}C NMR (100 MHz, $CDCl_3$)

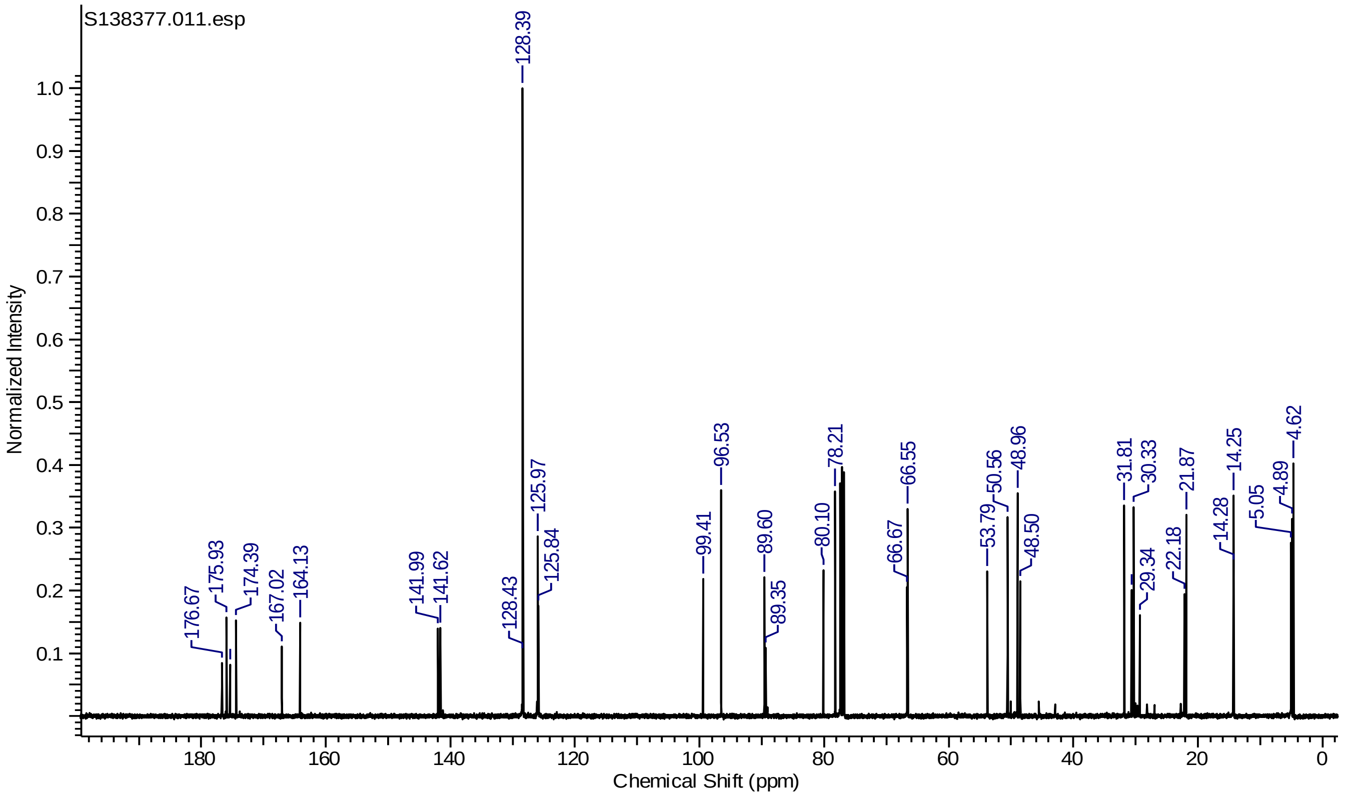

(3a*S*,4*R*,7*R*,7a*R*)-5,5-Diethoxy-2-methyl-4-phenethylhexahydro-1*H*-4,7-epoxyisoindole-1,3(2*H*)-dione *endo*-6a

^{1}H NMR (400 MHz, DMSO-d$_6$)

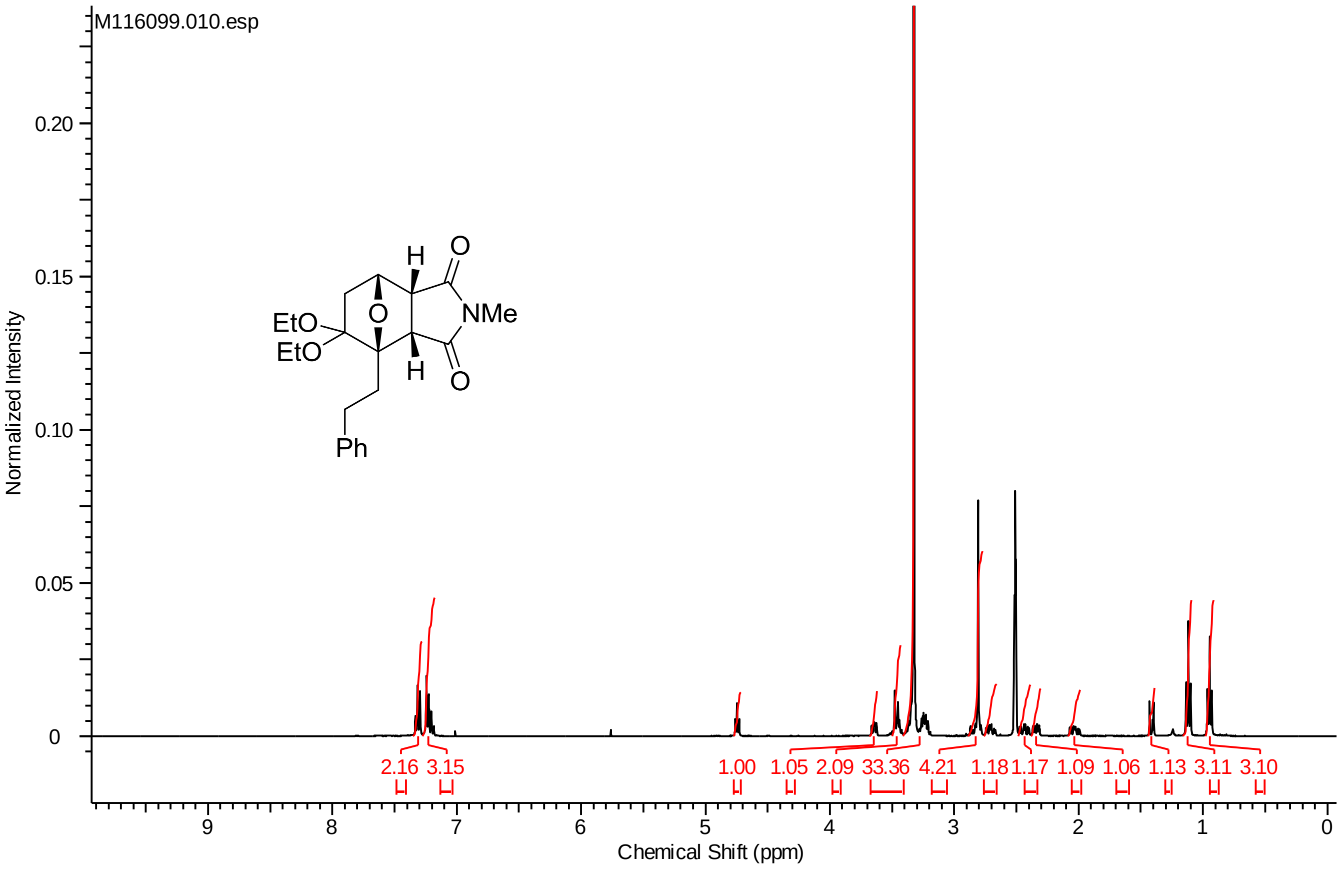

^{13}C NMR (100 MHz, DMSO-d$_6$)

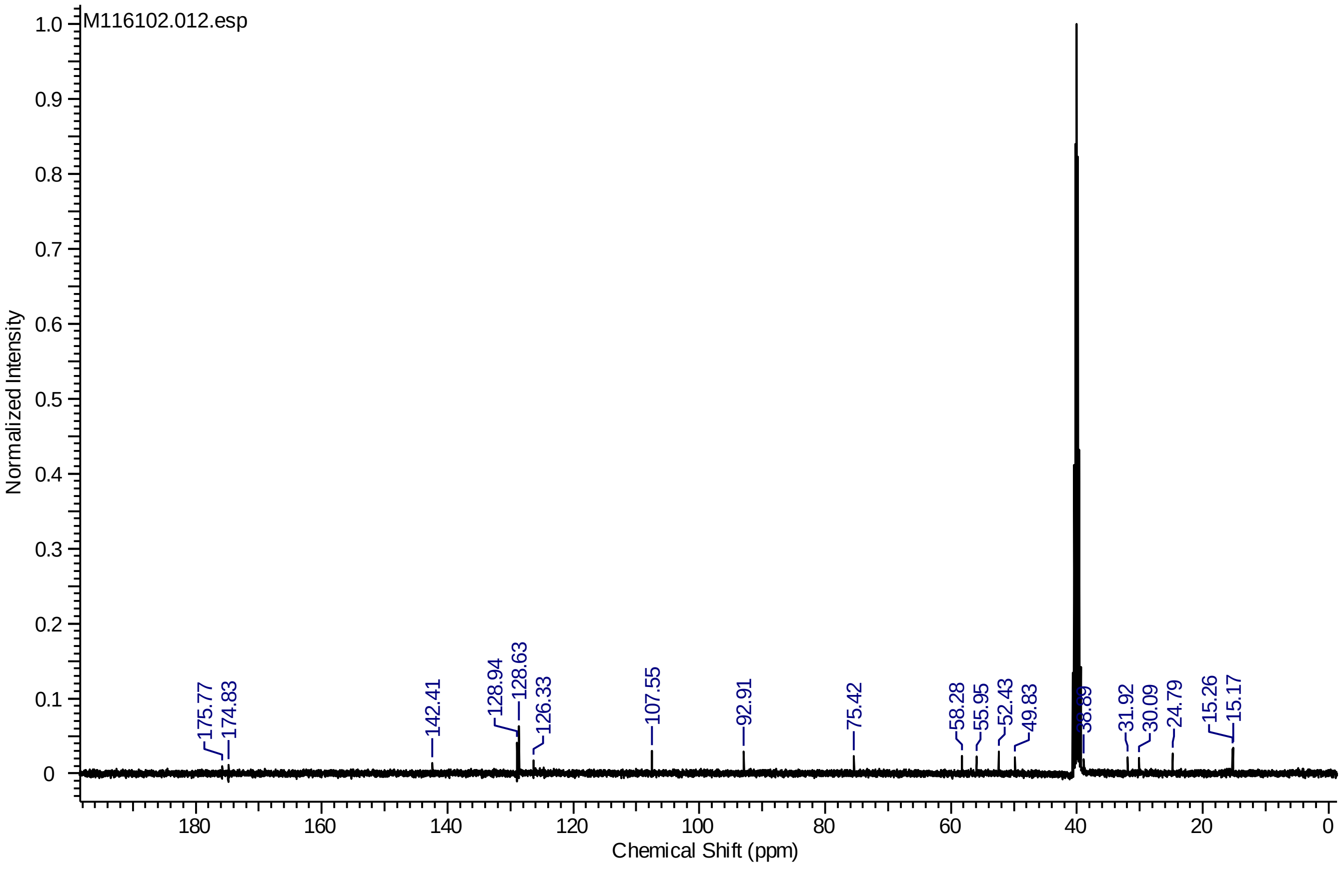

(3a*R*,4*R*,7*R*,7a*S*)-5,5-Diethoxy-2-methyl-4-phenethylhexahydro-1*H*-4,7-epoxyisoindole-1,3(2*H*)-dione *exo*-6b (with a 20% impurity of *endo*-6b)

^{1}H NMR (400 MHz, $CDCl_3$)

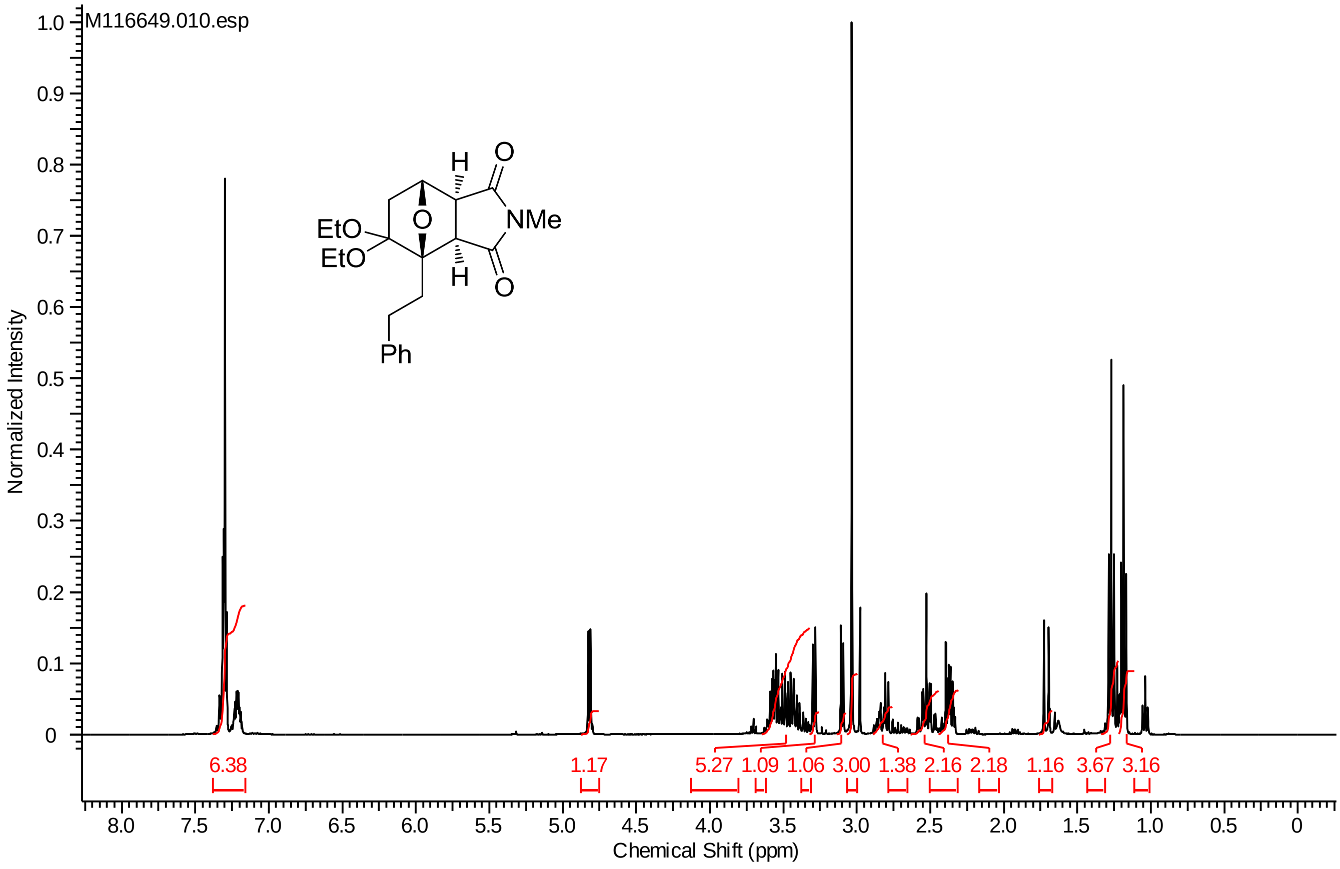

^{13}C NMR (100 MHz, $CDCl_3$)

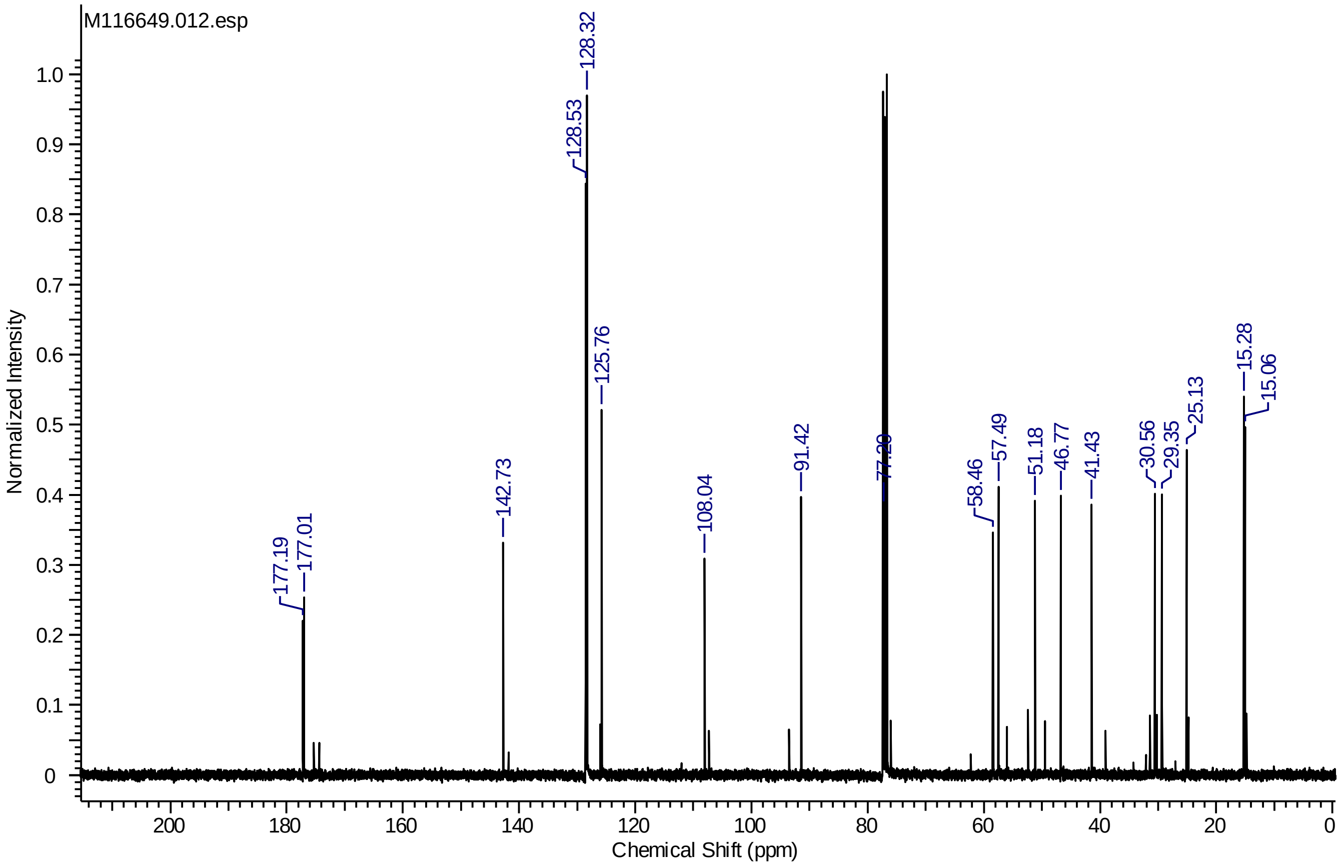

(3a*S*,4*S*,5*R*,7*R*,7a*R*)-5-Ethoxy-2-methyl-4-phenylhexahydro-1*H*-4,7-epoxyisoindole-1,3(2*H*)-dione

7

^{1}H NMR (400 MHz, $CDCl_3$)

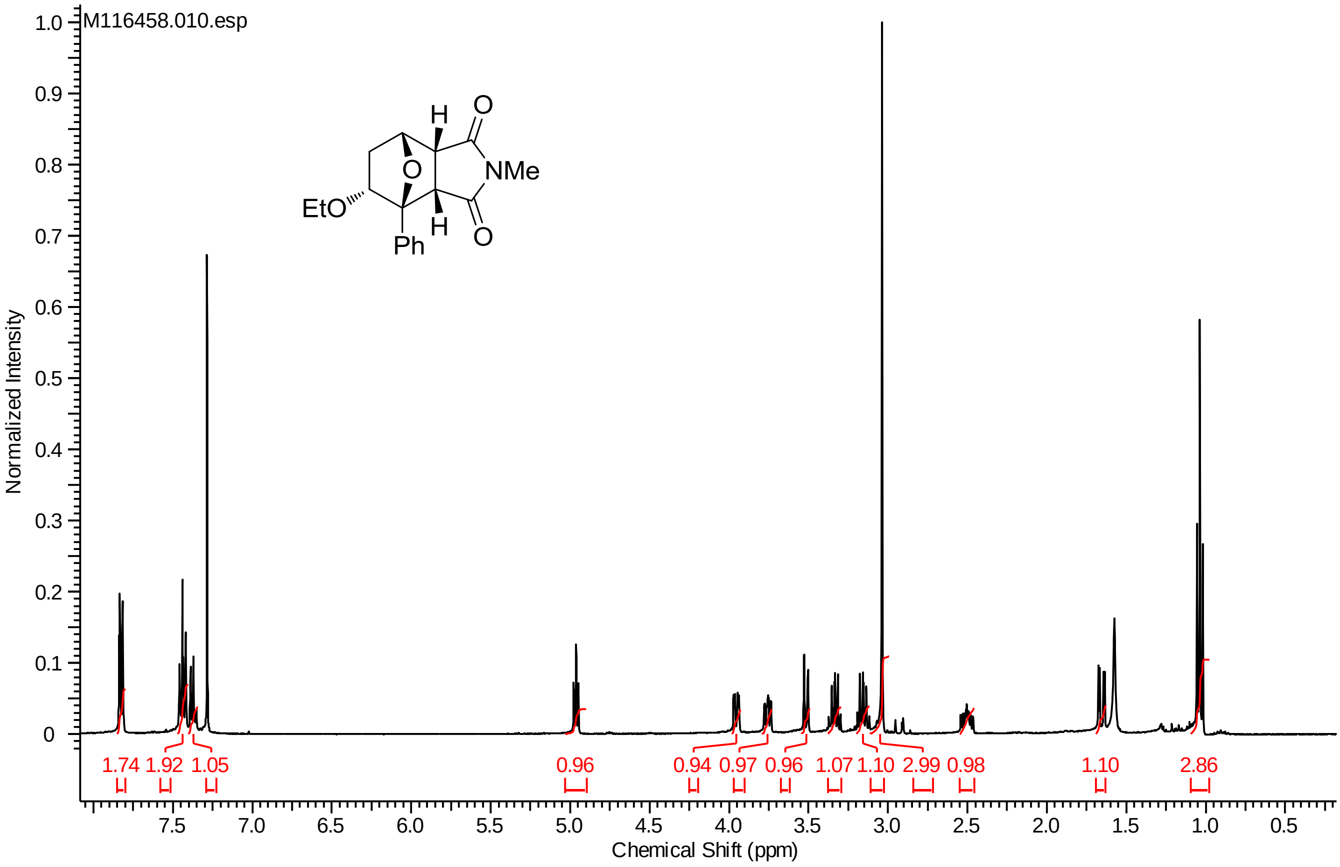

^{13}C NMR (100 MHz, $CDCl_3$)

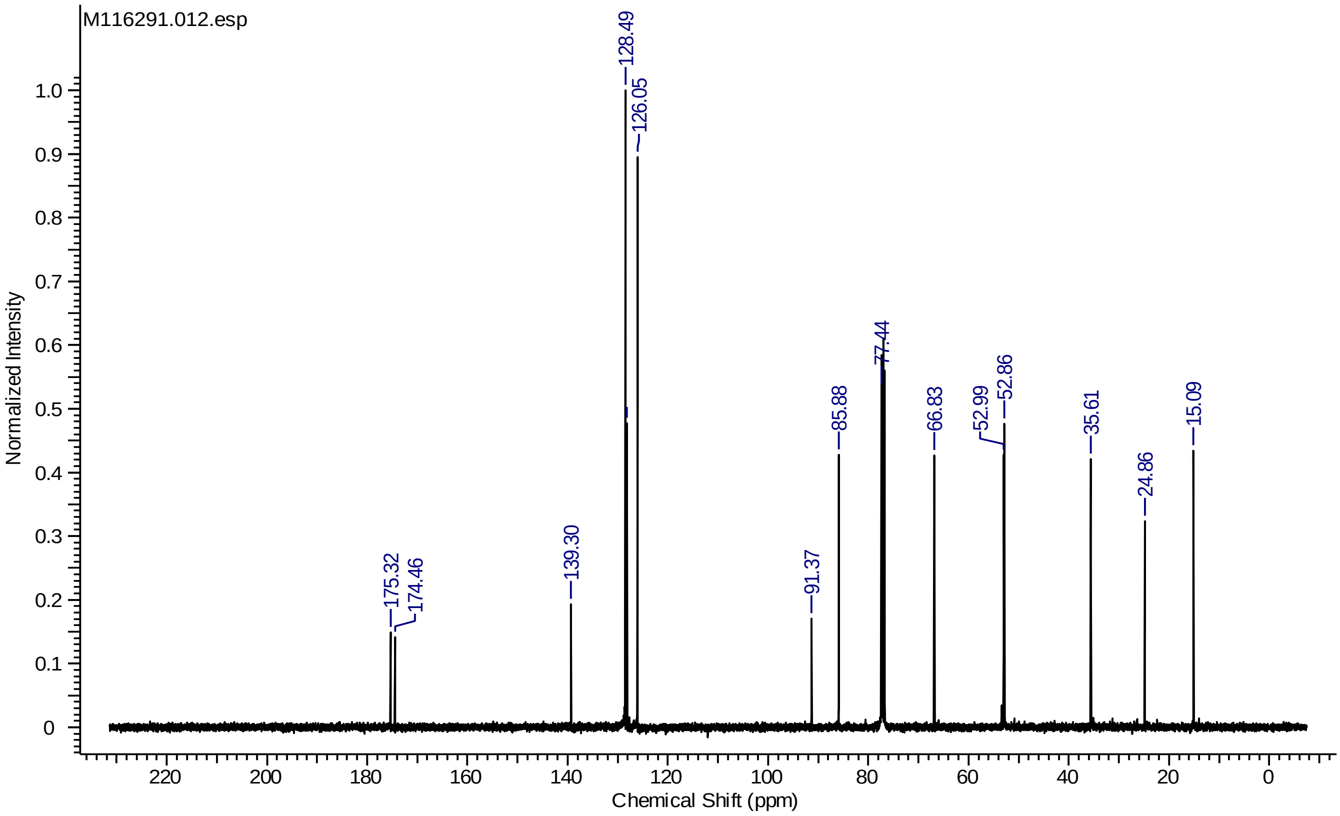

(3a*S*,4*S*,5*R*,6*S*,7*S*,7a*R*)-5-Ethoxy-6-hydroxy-2-methyl-4-phenylhexahydro-1*H*-4,7-epoxyisoindole-1,3(2*H*)-dione 8

^{1}H NMR (400 MHz, DMSO-d_6)

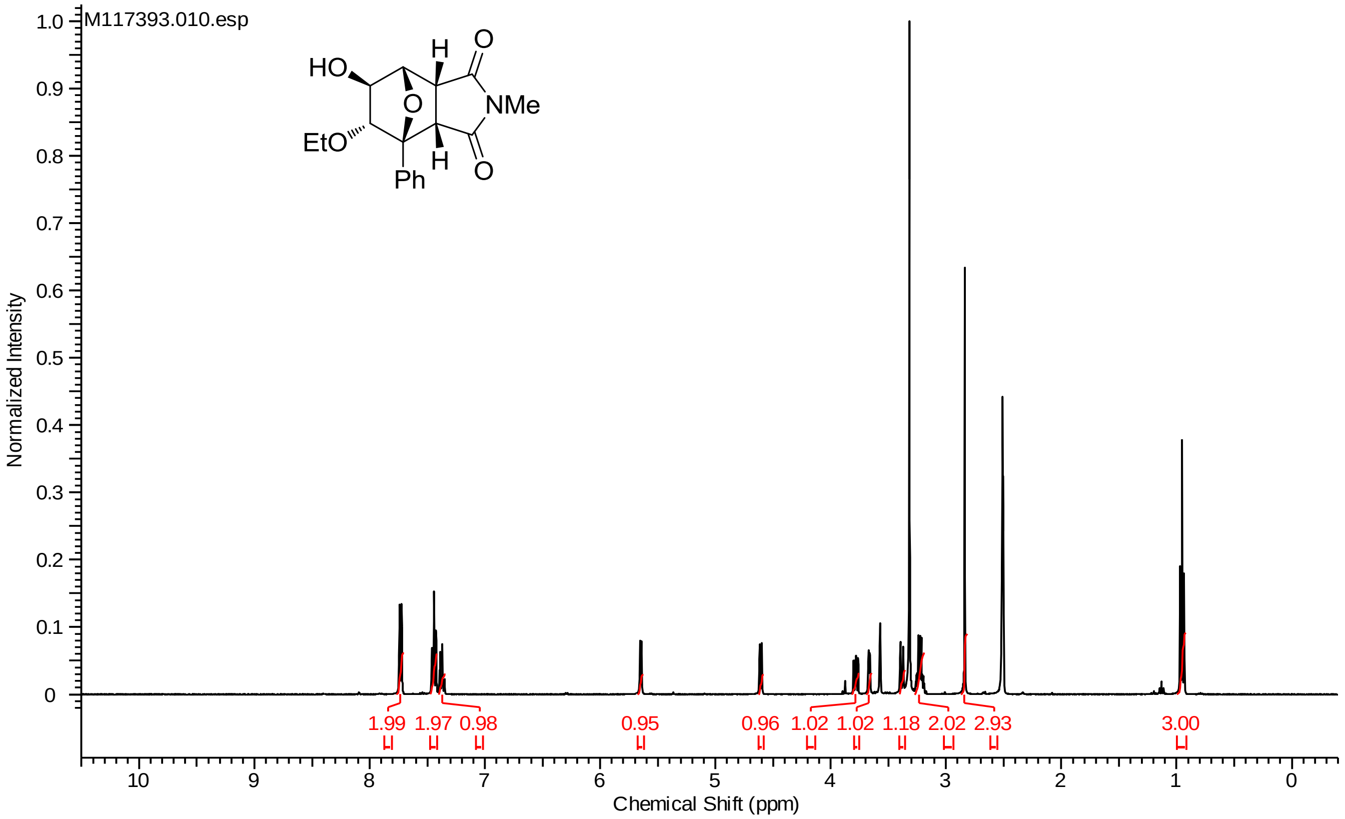

^{13}C NMR (100 MHz, DMSO-d_6)

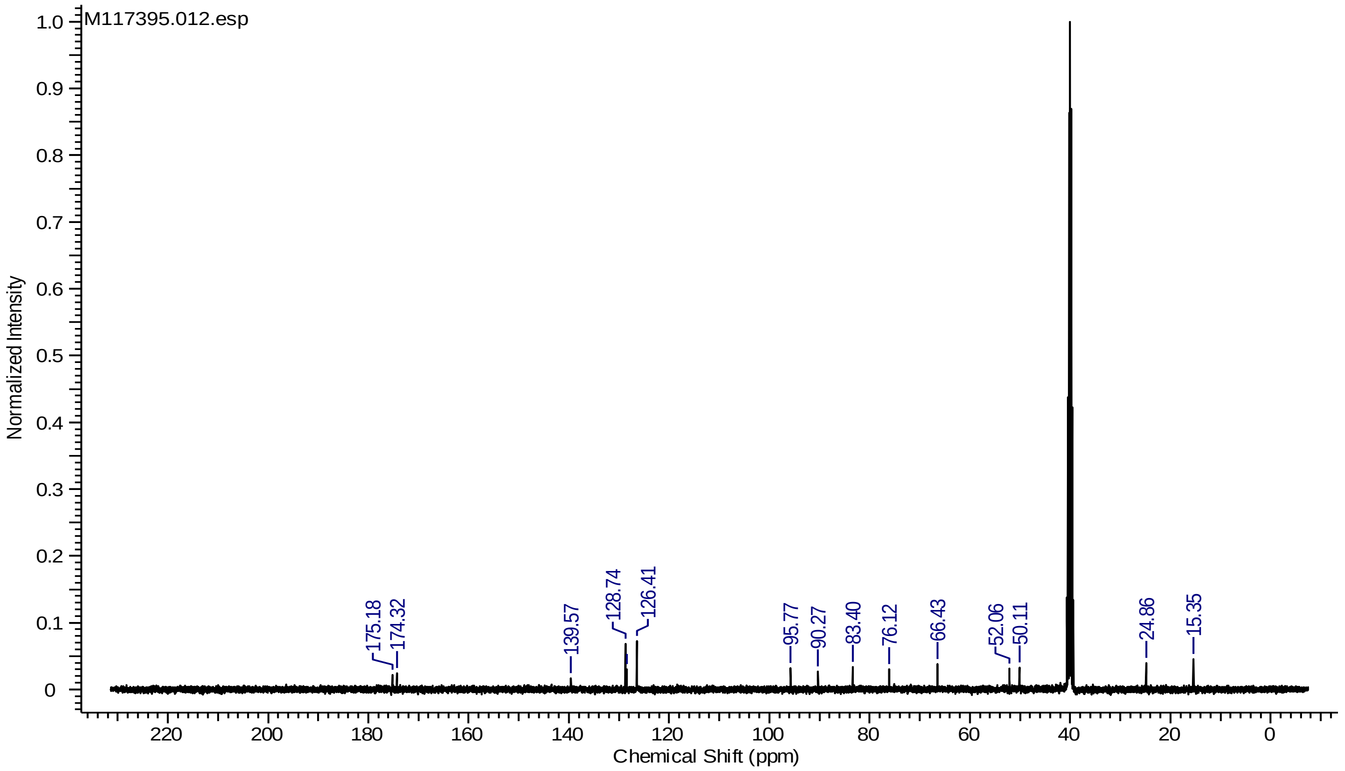

(3a*S*,4*S*,7*R*,7a*R*)-2-Methyl-4-phenyltetrahydro-1H-4,7-epoxyisoindole-1,3,5(2H,6H)-trione 9

^{1}H NMR (400 MHz, $CDCl_3$)

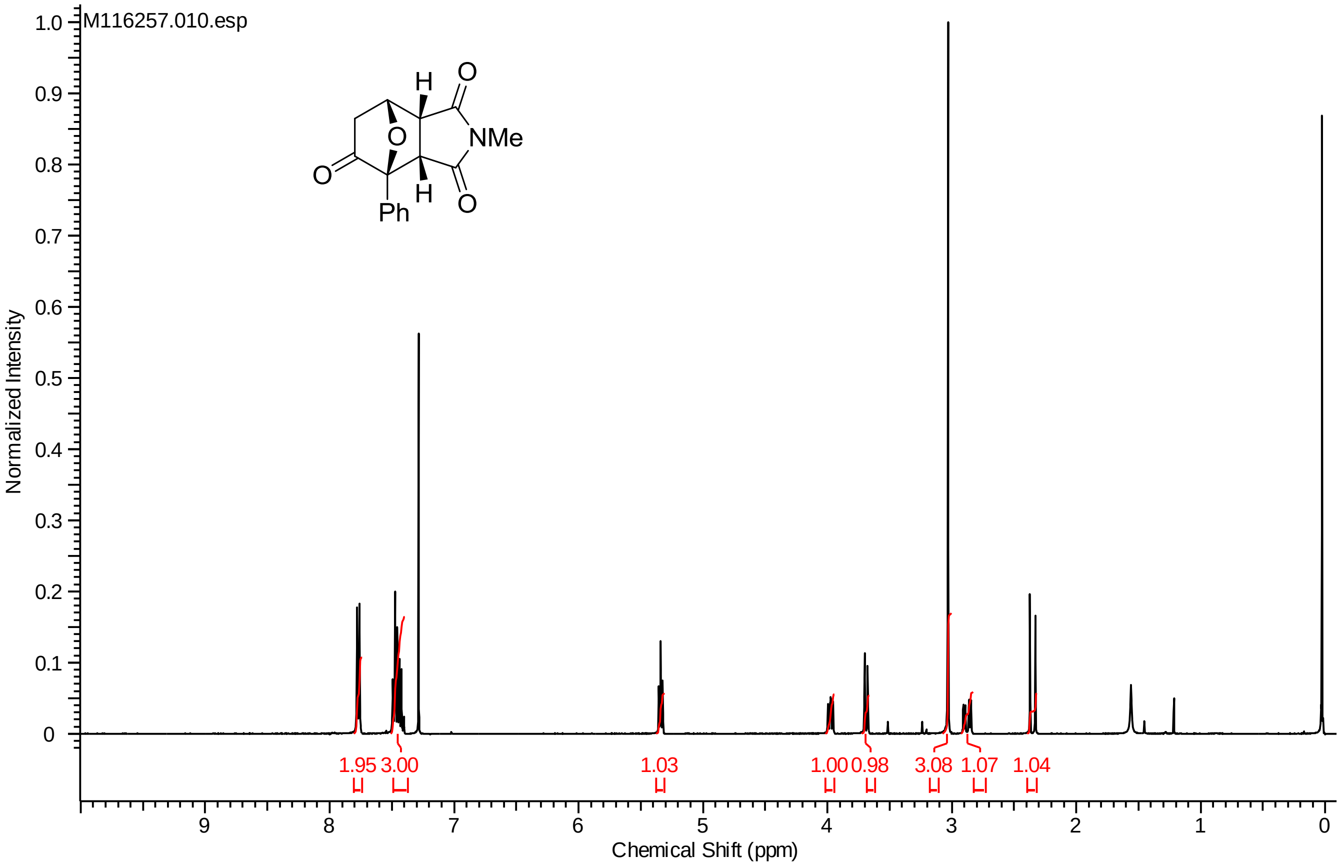

^{13}C NMR (100 MHz, $CDCl_3$)

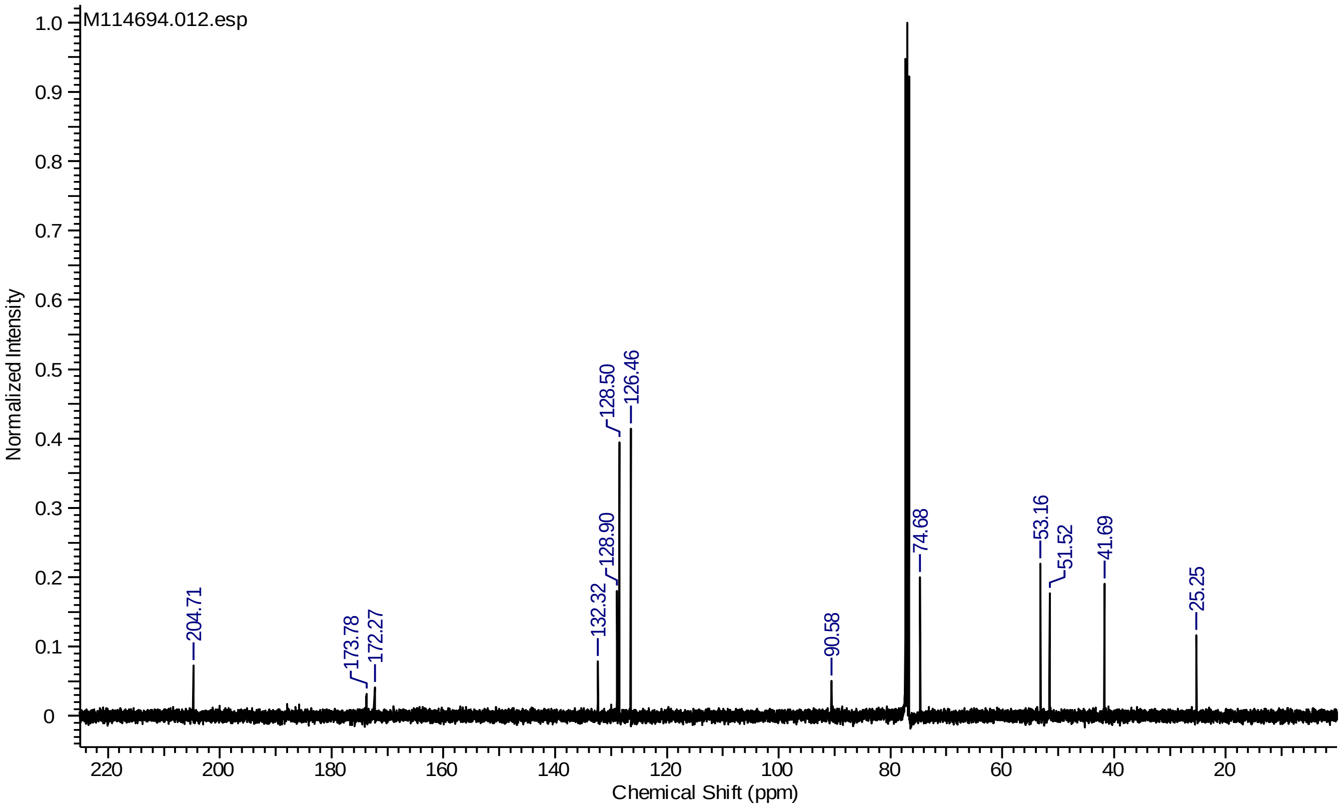

(3a*S*,4*S*,5*R*,7*R*,7a*R*)-5-Hydroxy-2-methyl-4-phenylhexahydro-1*H*-4,7-epoxyisoindole-1,3(2*H*)-dione 10

^{1}H NMR (400 MHz, $CDCl_3$)

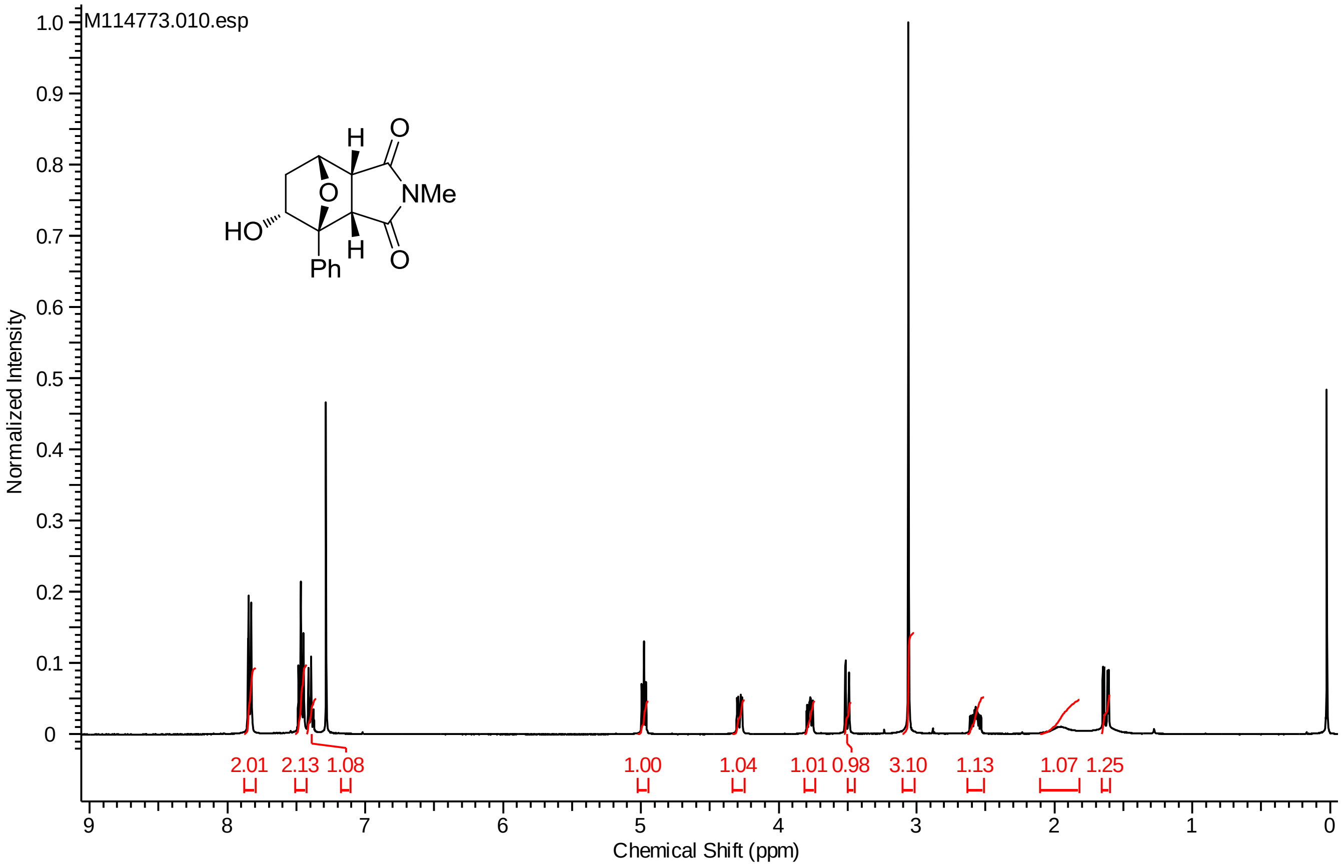

^{13}C NMR (100 MHz, $CDCl_3$)

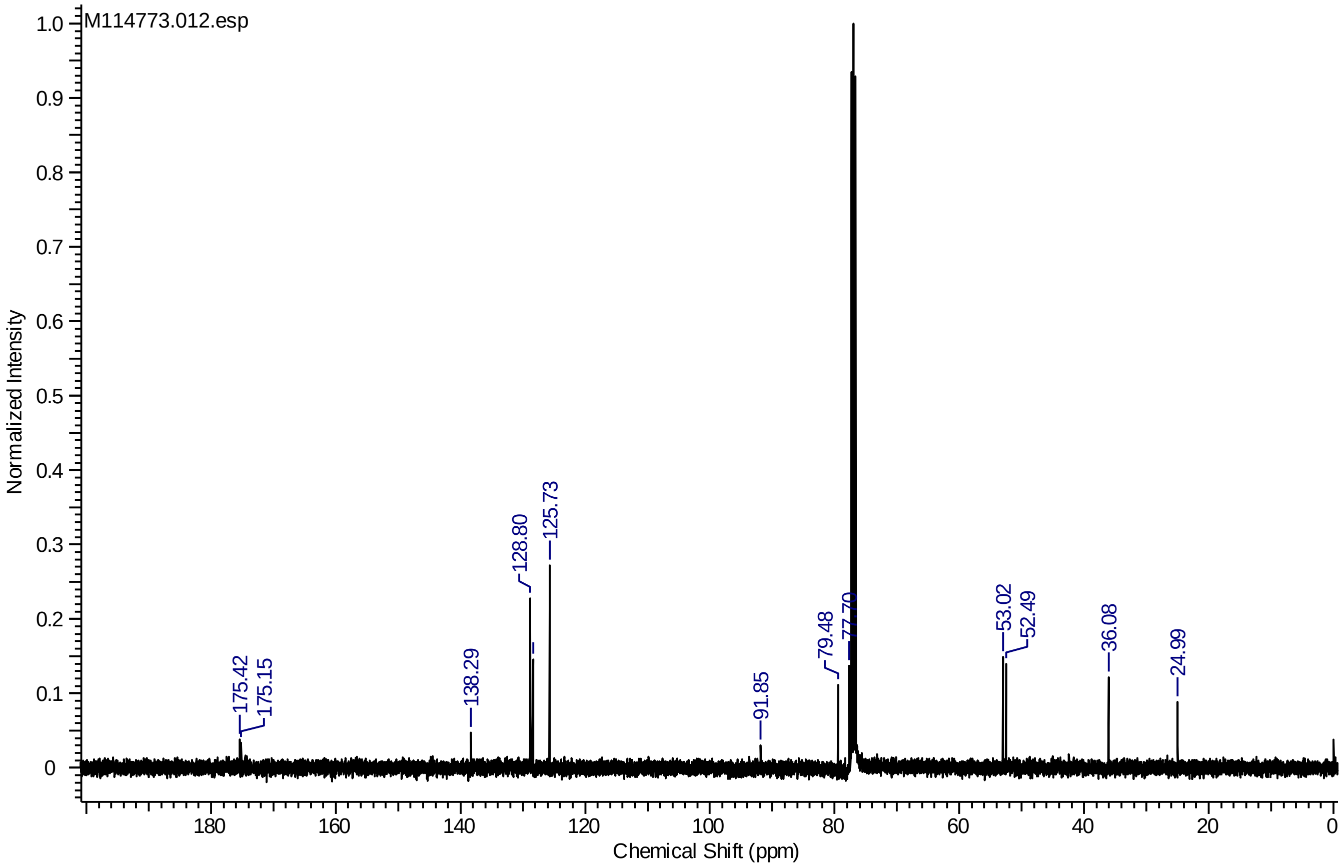

5-Ethoxy-2-methyl-4-phenylisoindoline-1,3-dione 11

^{1}H NMR (400 MHz, $CDCl_3$)

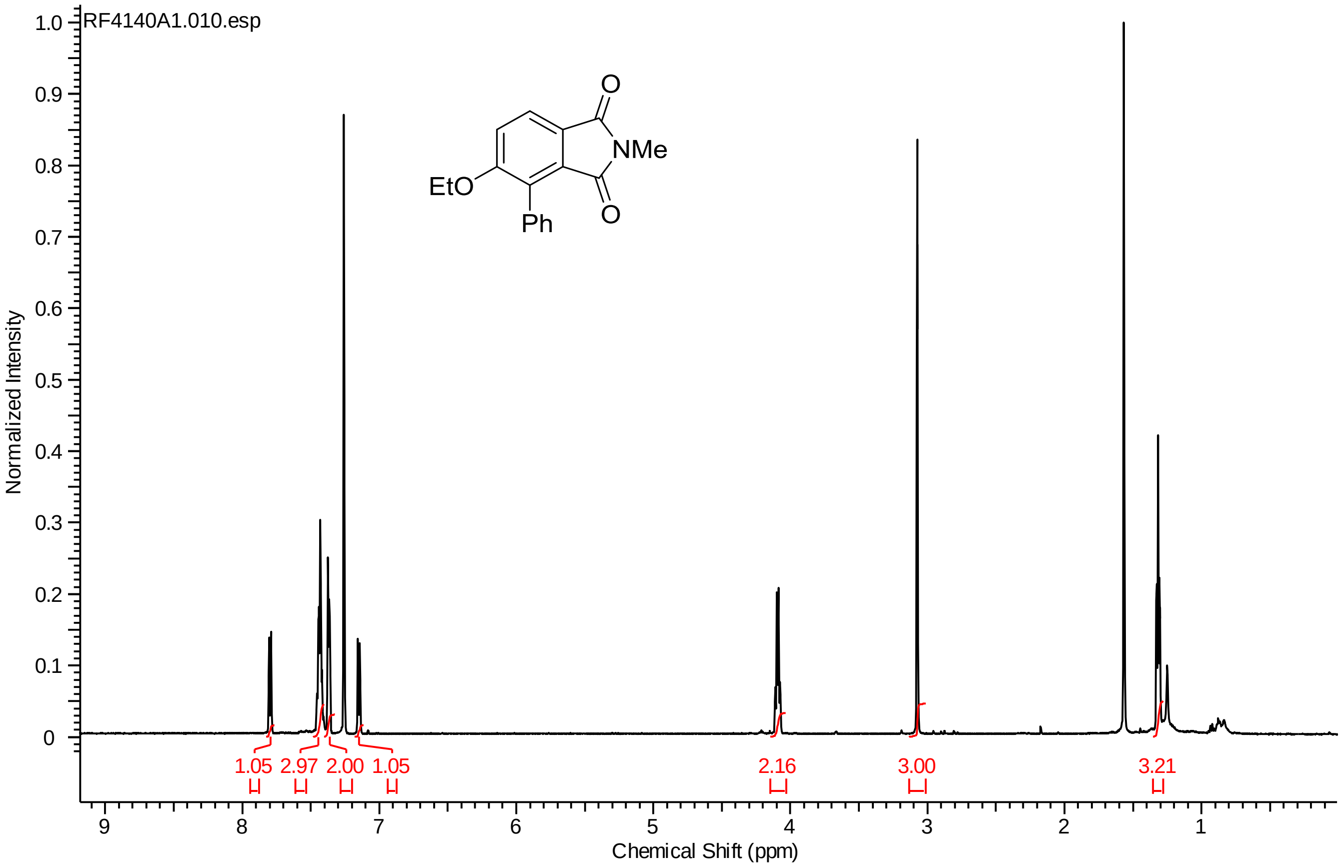

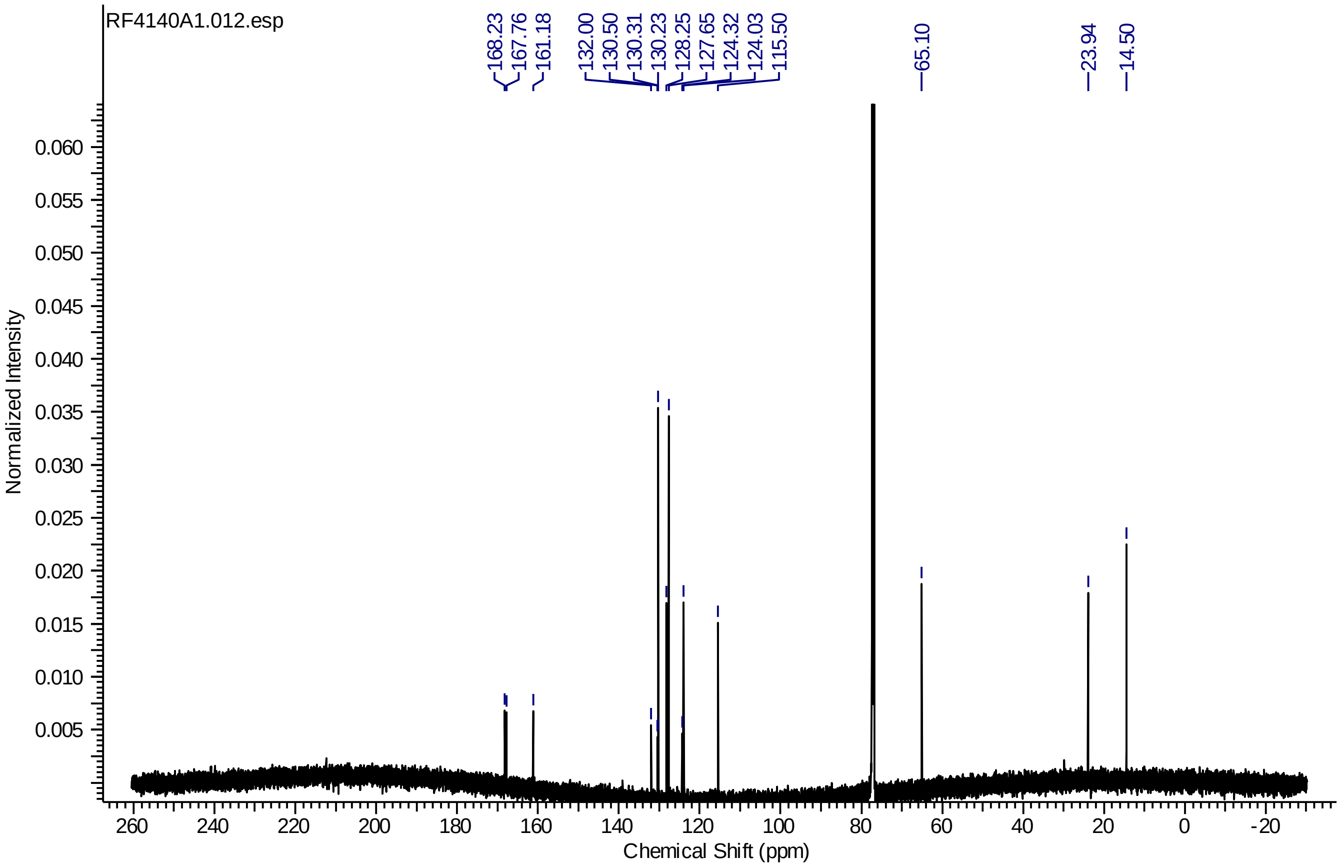

(1*R*,2*S*,3*R*,4*R*)-Dimethyl 6-oxo-1-phenethyl-7-oxabicyclo[2.2.1]heptane-2,3-dicarboxylate *endo*-13a and (1*R*,2*R*,3*S*,4*R*)-Dimethyl 6-oxo-1-phenethyl-7-oxabicyclo[2.2.1]heptane-2,3-dicarboxylate *exo*-13a

^{1}H NMR (400 MHz, $CDCl_3$)

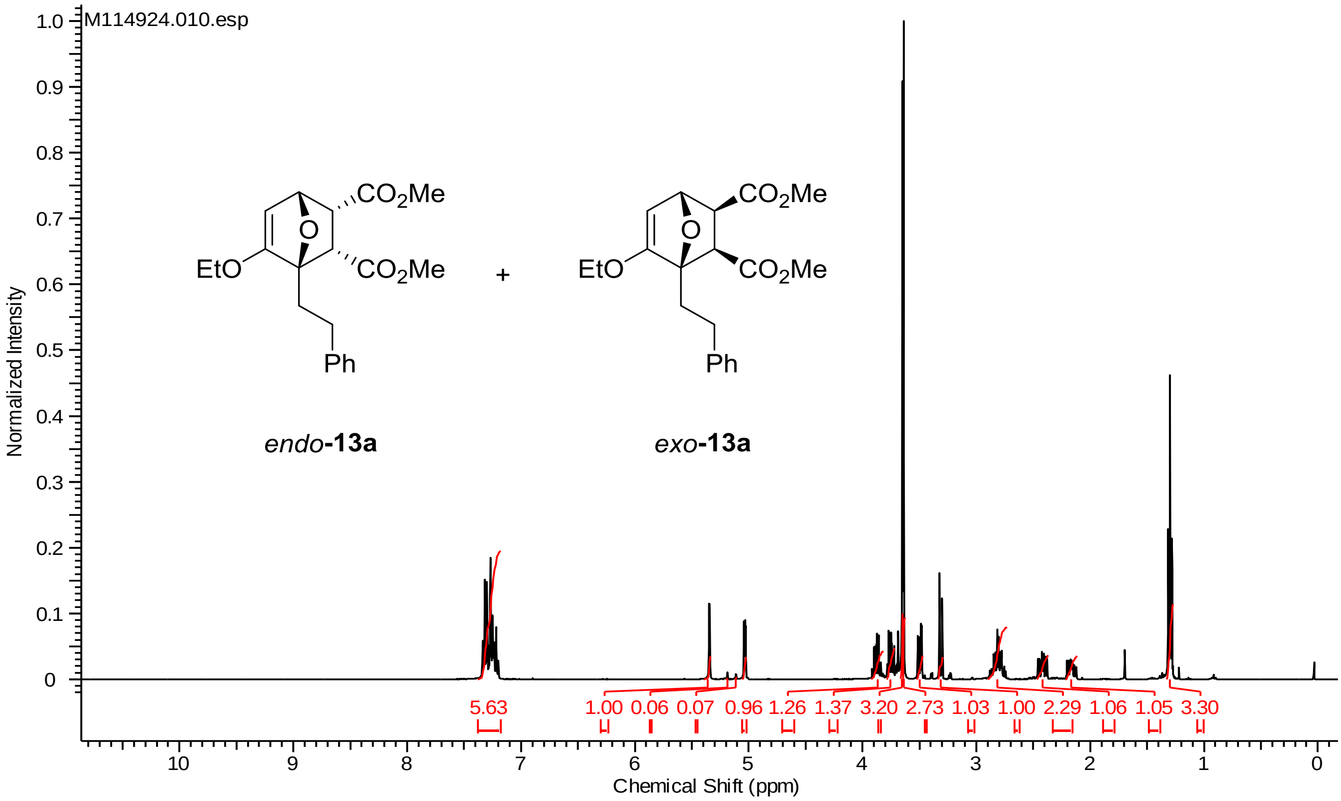

^{13}C NMR (100 MHz, $CDCl_3$)

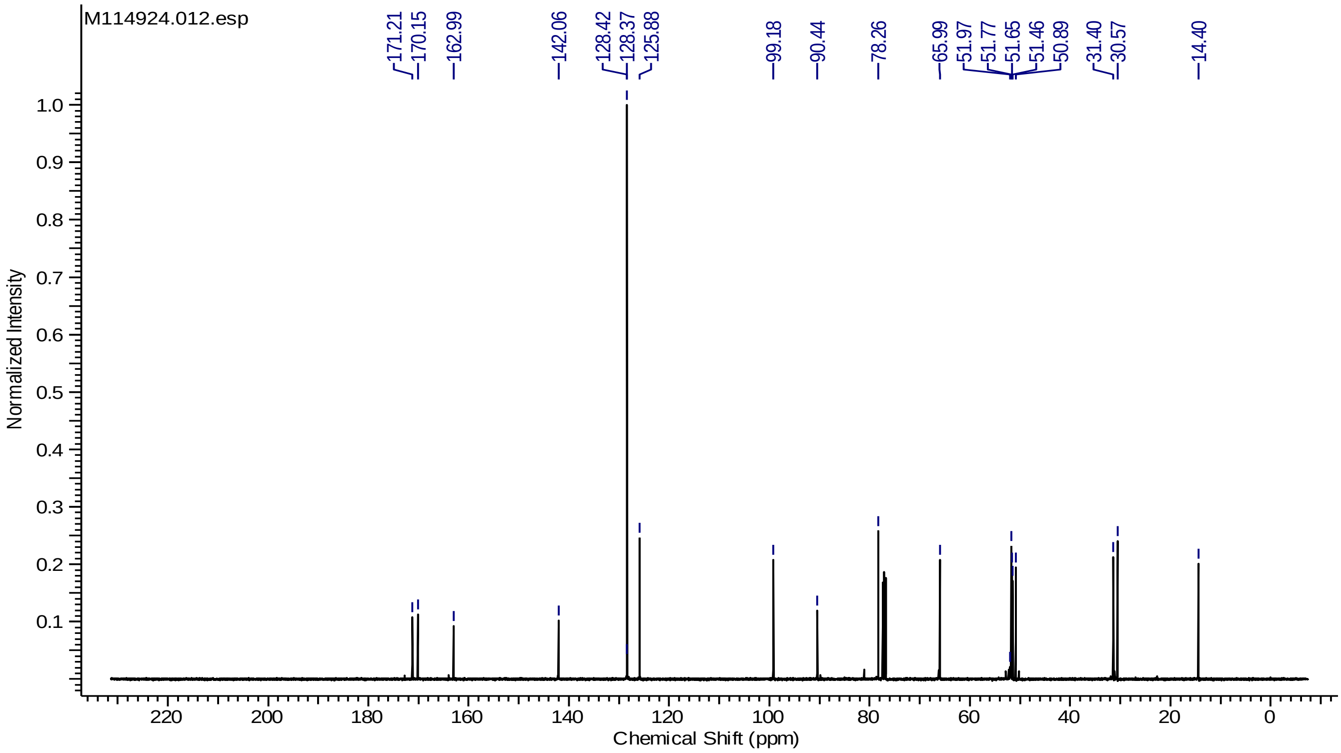

(1*R*,2*R*,3*R*,4*R*)-Dimethyl 6-oxo-1-phenethyl-7-oxabicyclo[2.2.1]heptane-2,3-dicarboxylate 3-*endo*-13b and (1*R*,2*S*,3*S*,4*R*)-Dimethyl 6-oxo-1-phenethyl-7-oxabicyclo[2.2.1]heptane-2,3-dicarboxylate 3-*exo*-13b

^{1}H NMR (400 MHz, $CDCl_3$)

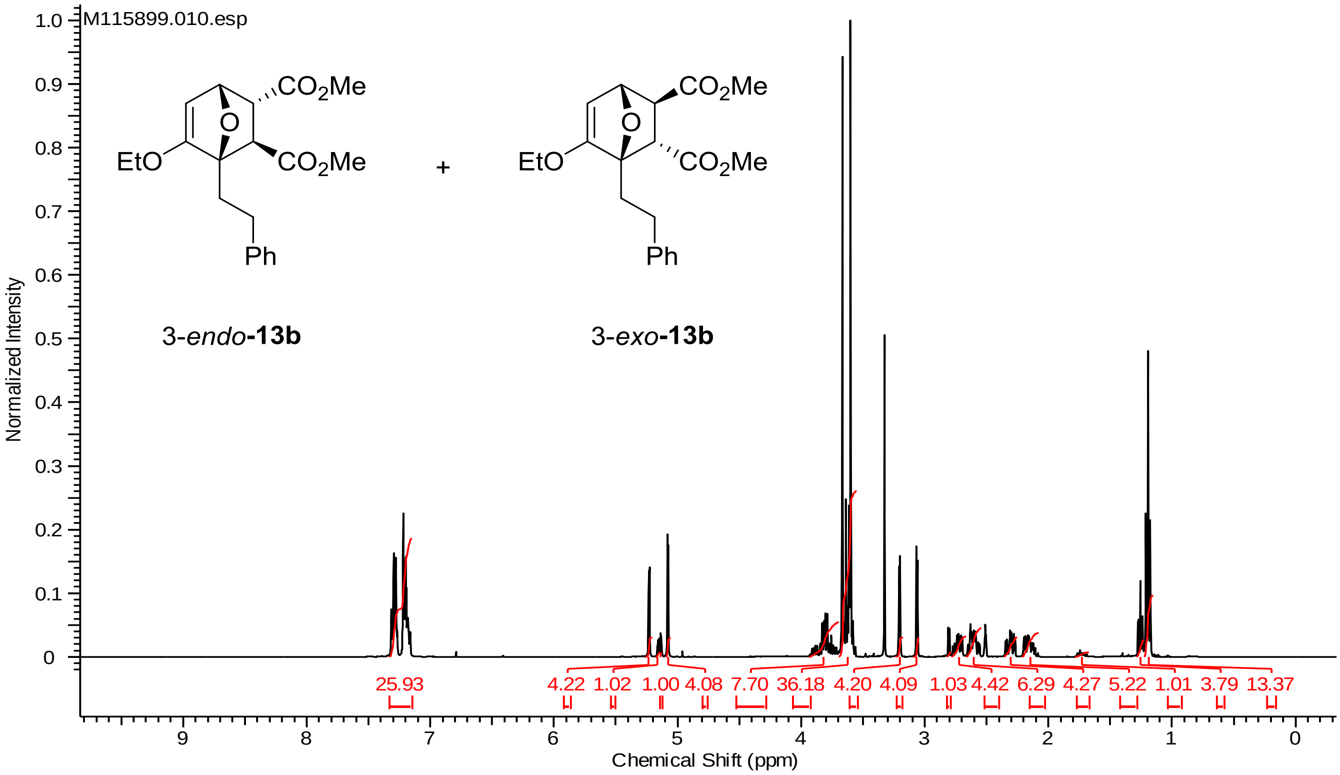

^{13}C NMR (100 MHz, $CDCl_3$)

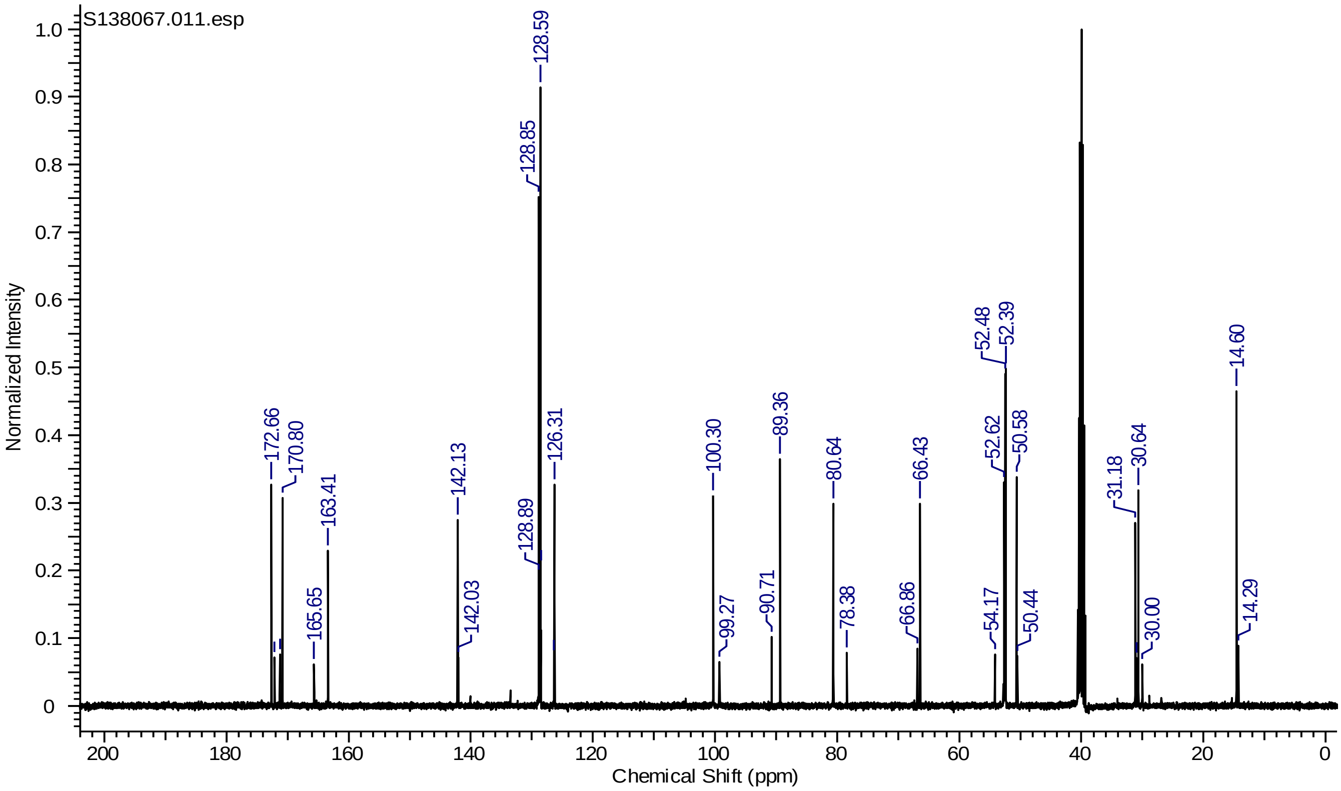

(1*R*,2*R*,3*R*,4*R*)-Diethyl 6-oxo-1-phenethyl-7-oxabicyclo[2.2.1]heptane-2,3-dicarboxylate 3-*endo*-13c and (1*R*,2*S*,3*S*,4*R*)-Diethyl 6-oxo-1-phenethyl-7-oxabicyclo[2.2.1]heptane-2,3-dicarboxylate 3-*exo*-13c

^{1}H NMR (400 MHz, CDCl$_3$)

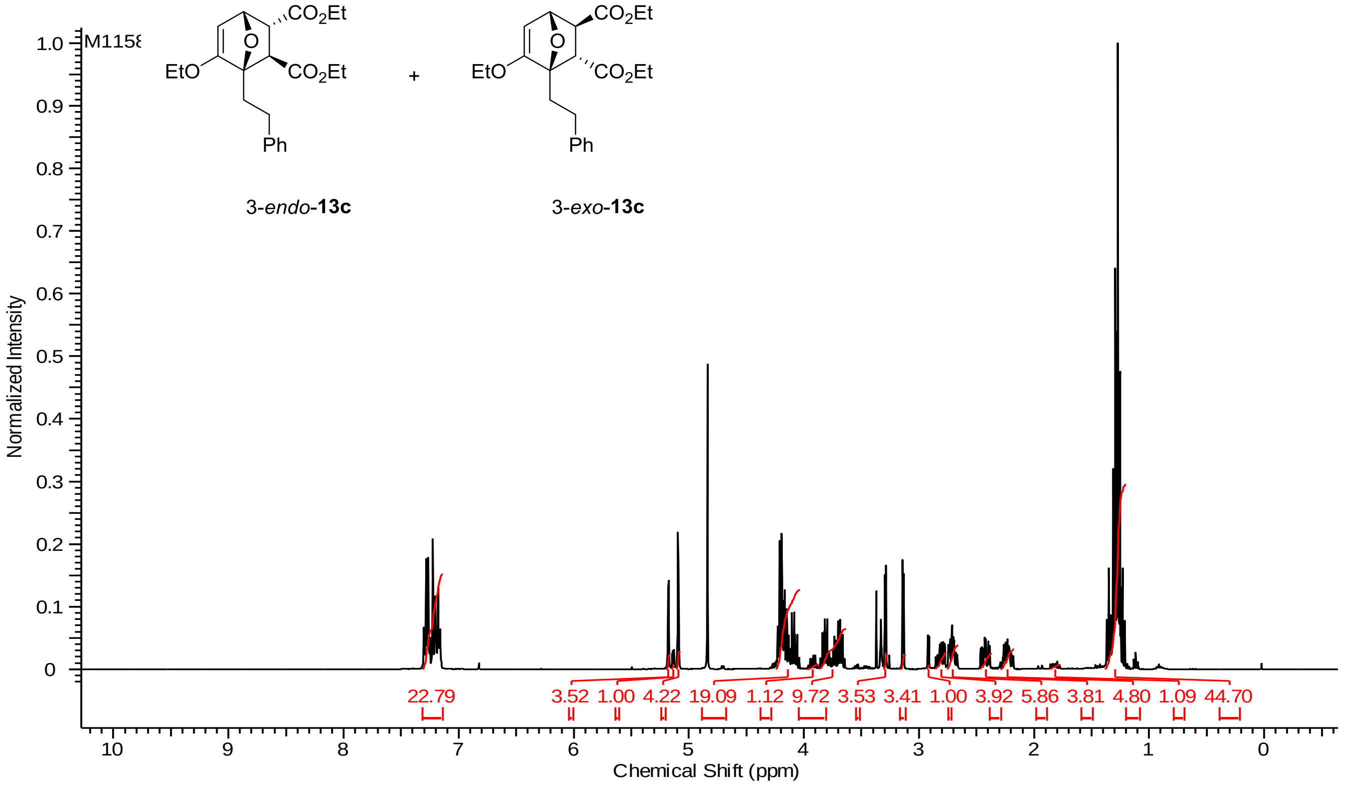

^{13}C NMR (100 MHz, CDCl$_3$)

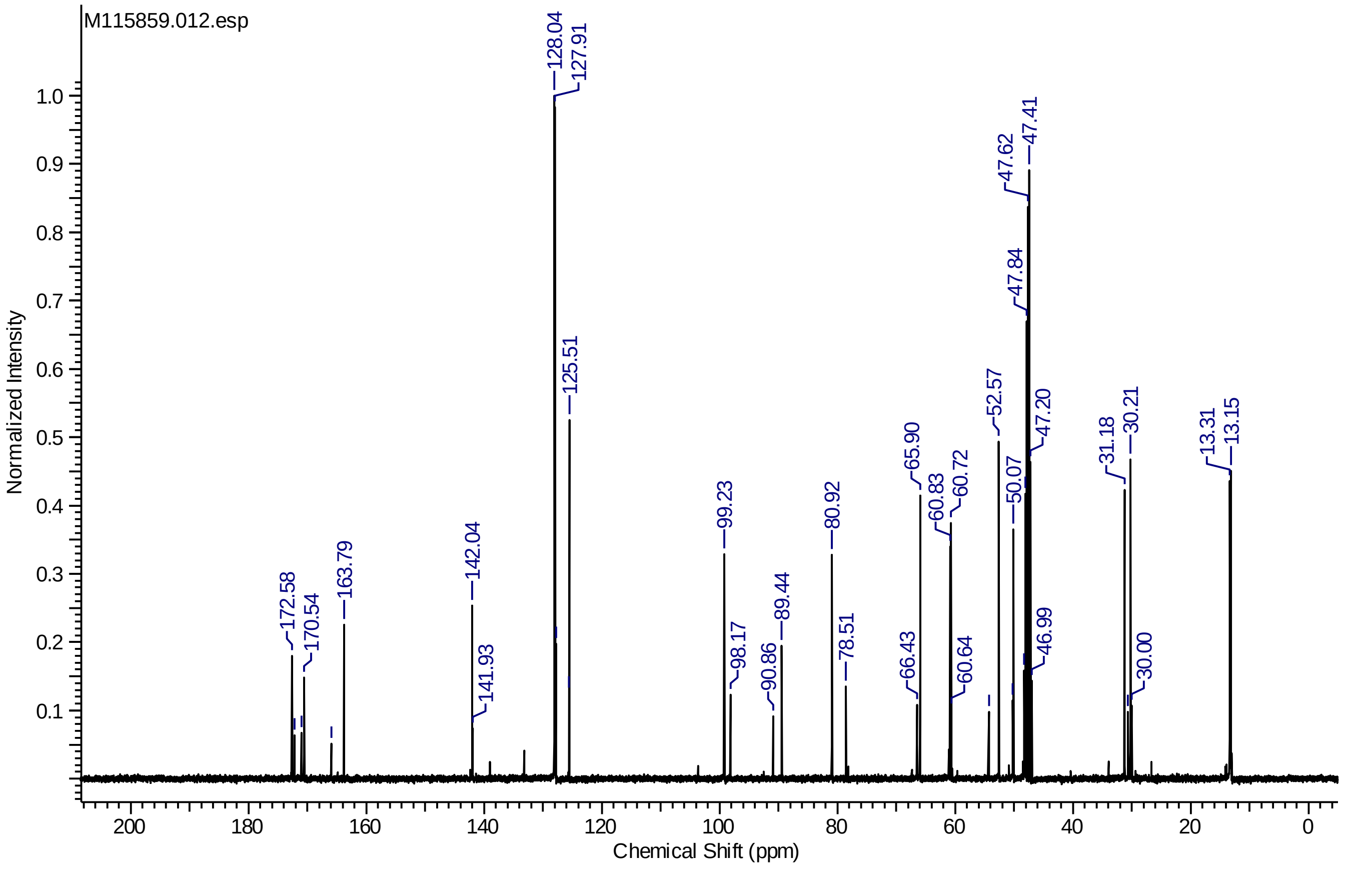

(1*R*,4*R*,5*R*)-1-Phenethyl-5-propionyl-7-oxabicyclo[2.2.1]heptan-2-one *endo*-13d, (1*R*,4*R*,5*S*)-1-Phenethyl-5-propionyl-7-oxabicyclo[2.2.1]heptan-2-one *exo*-13d and (1R,4S)-1-phenethyl-6-propionyl-7-oxabicyclo[2.2.1]heptan-2-one 13d'

^{1}H NMR (600 MHz, DMSO-d$_6$, Fraction A)

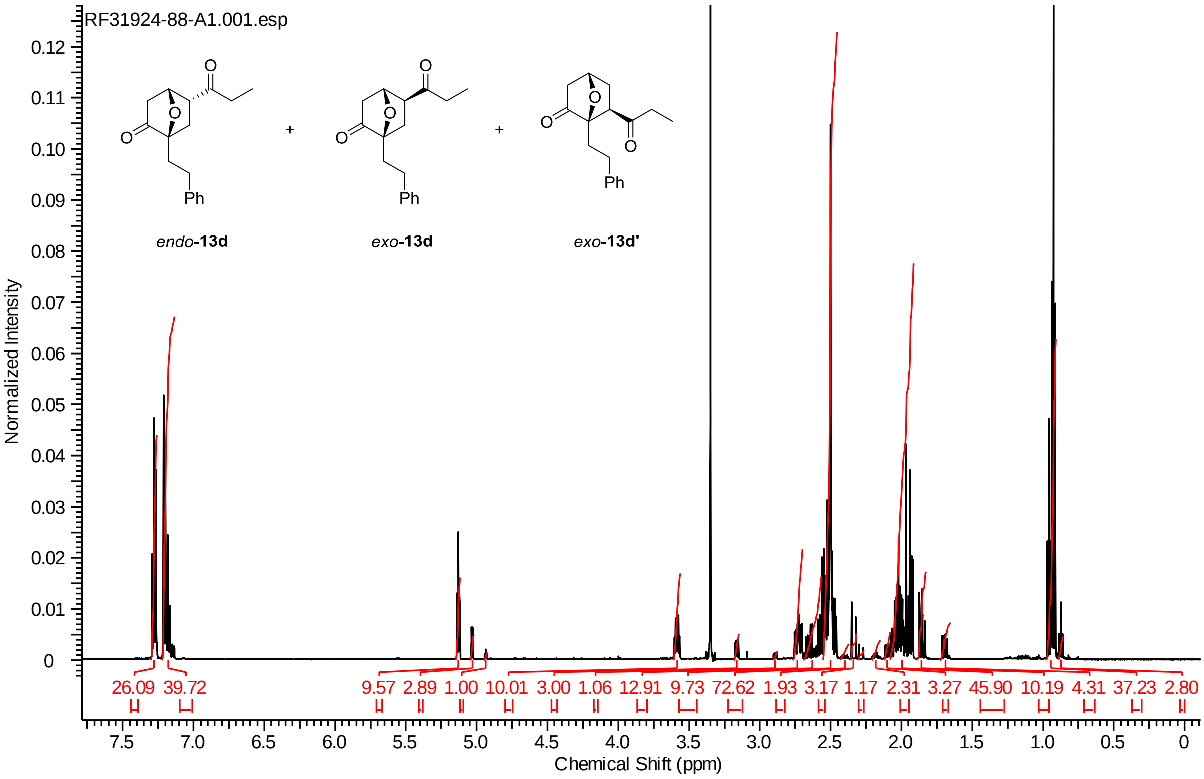

^{13}C NMR (150 MHz, DMSO-d$_6$, Fraction A)

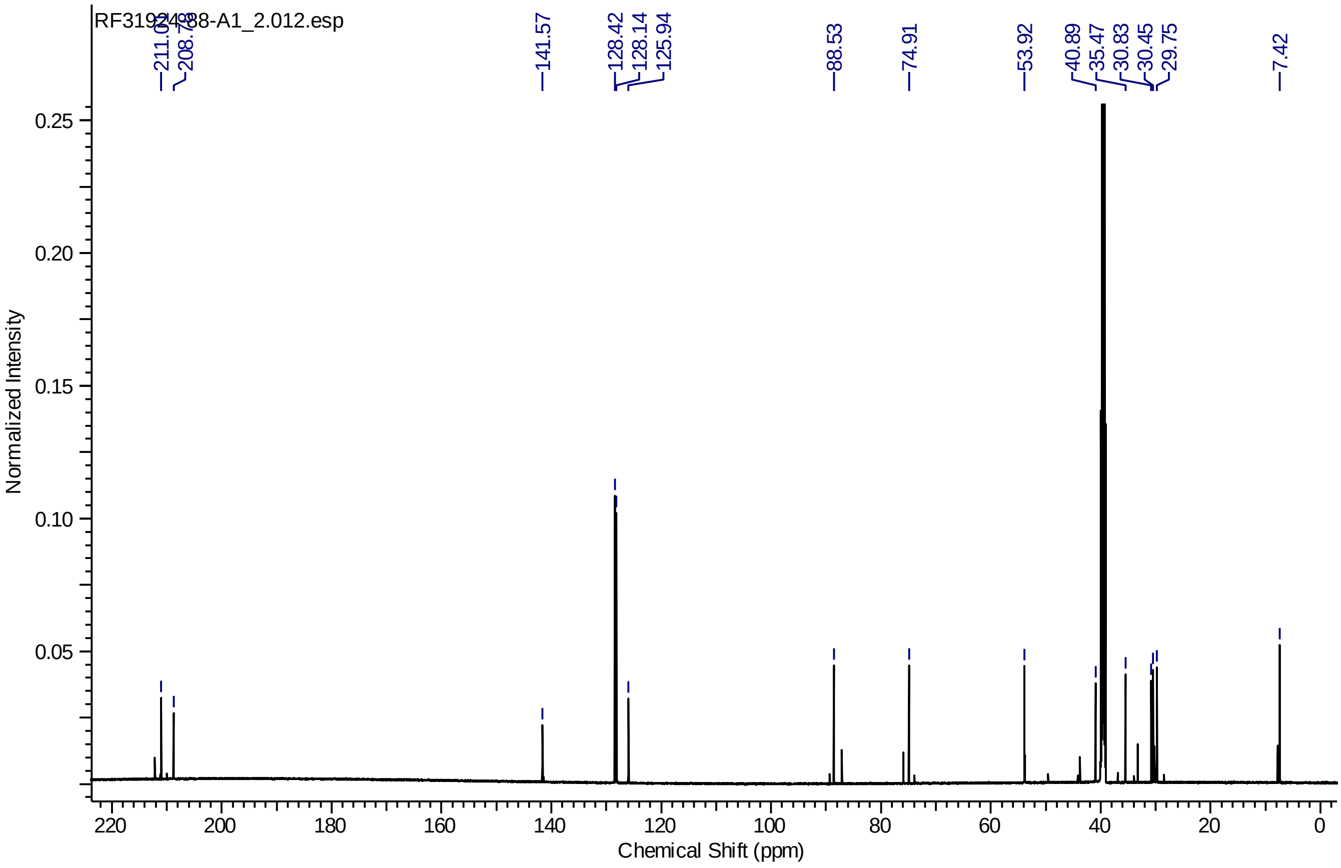

TOCSY NMR (600 MHz, DMSO-d$_6$, *endo*-**13d**)

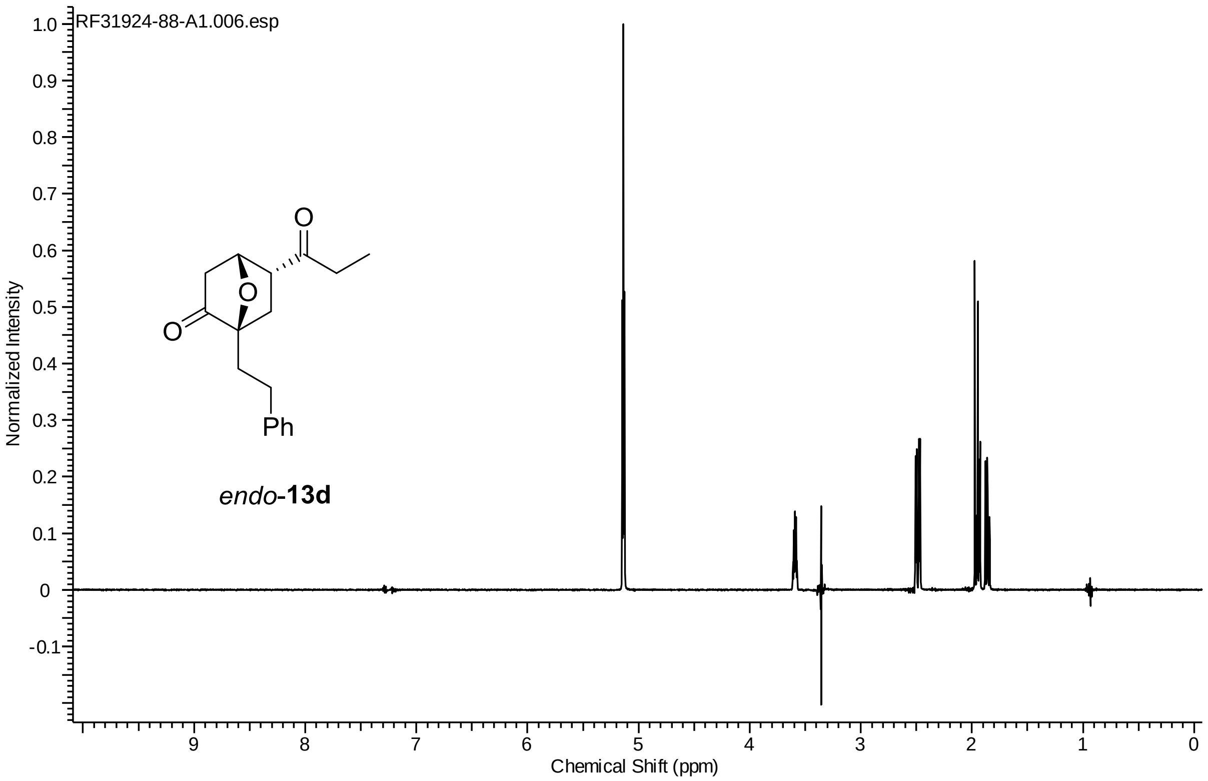

TOCSY NMR (600 MHz, DMSO-d$_6$, *exo*-**13d**)

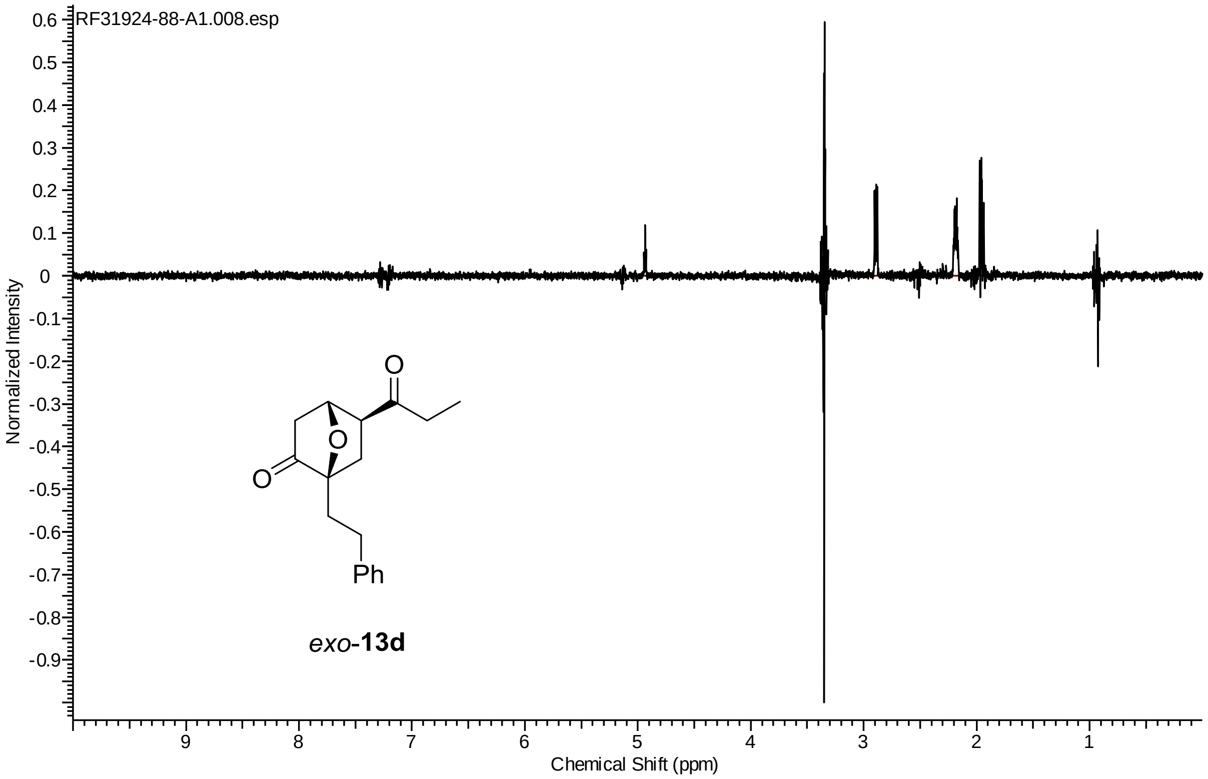

TOCSY NMR (600 MHz, DMSO-d_6 **13d'**)

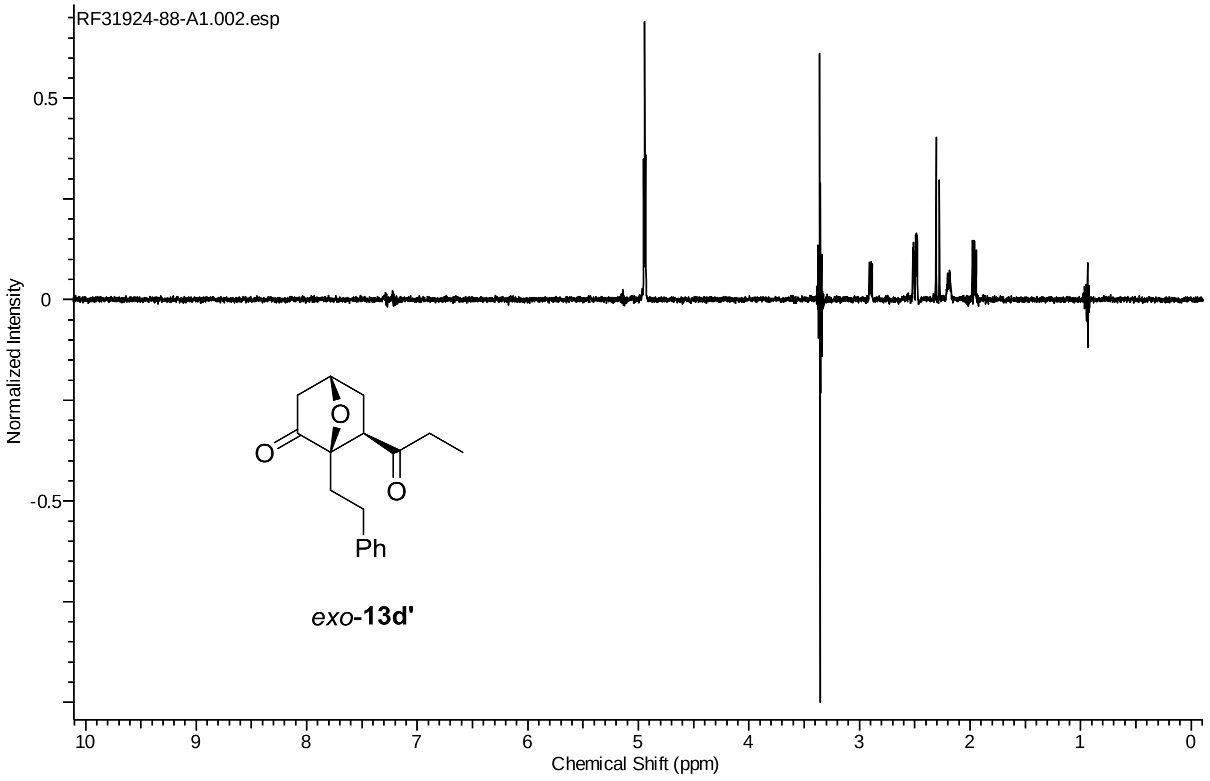

^{1}H NMR (400 MHz, MeOH-d_4, Fraction B)

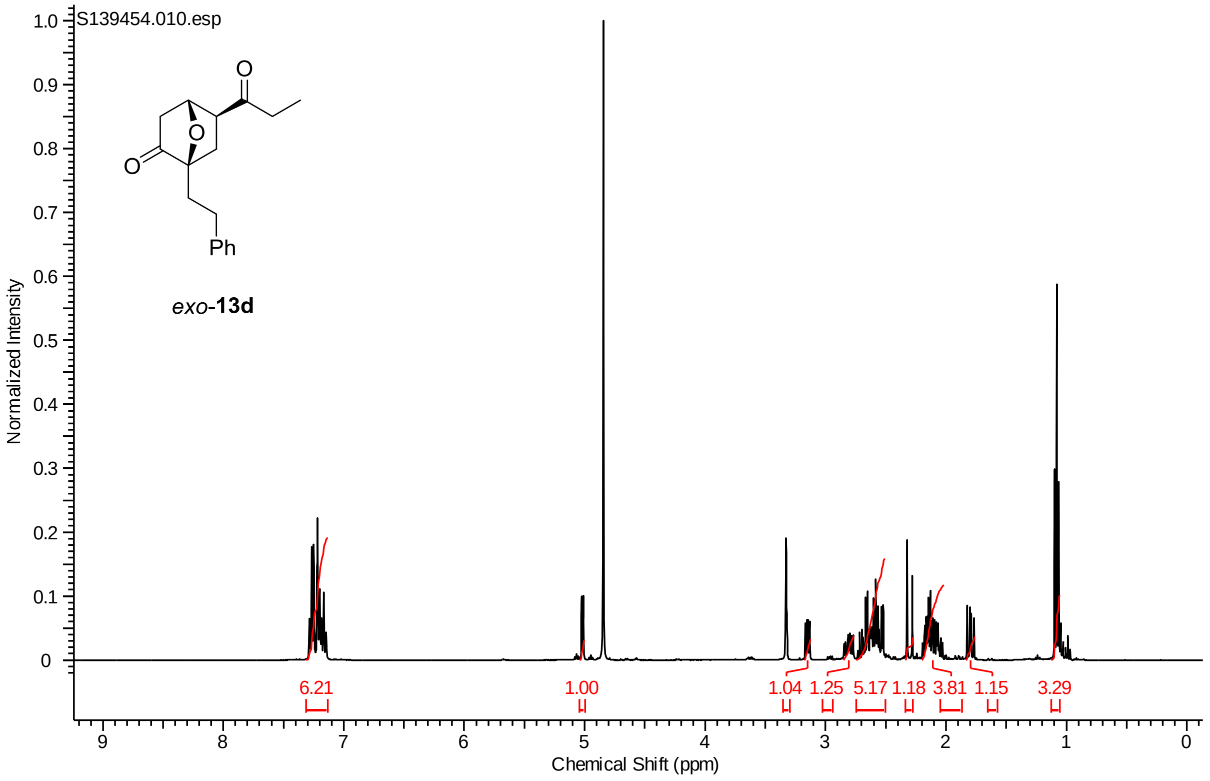

^{13}C NMR (150 MHz, $CDCl_3$, Fraction B)

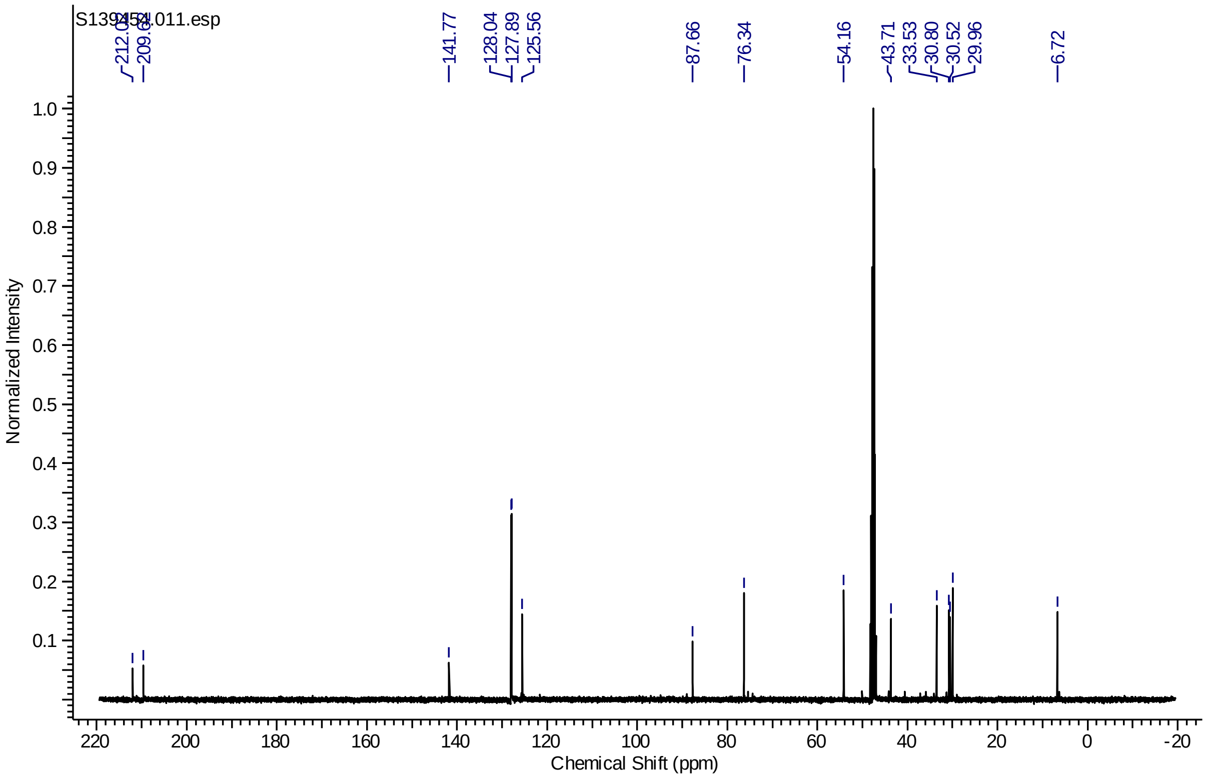

(1*R*,2*R*,4*R*)-Ethyl 5-oxo-4-phenethyl-7-oxabicyclo[2.2.1]heptane-2-carboxylate 13e

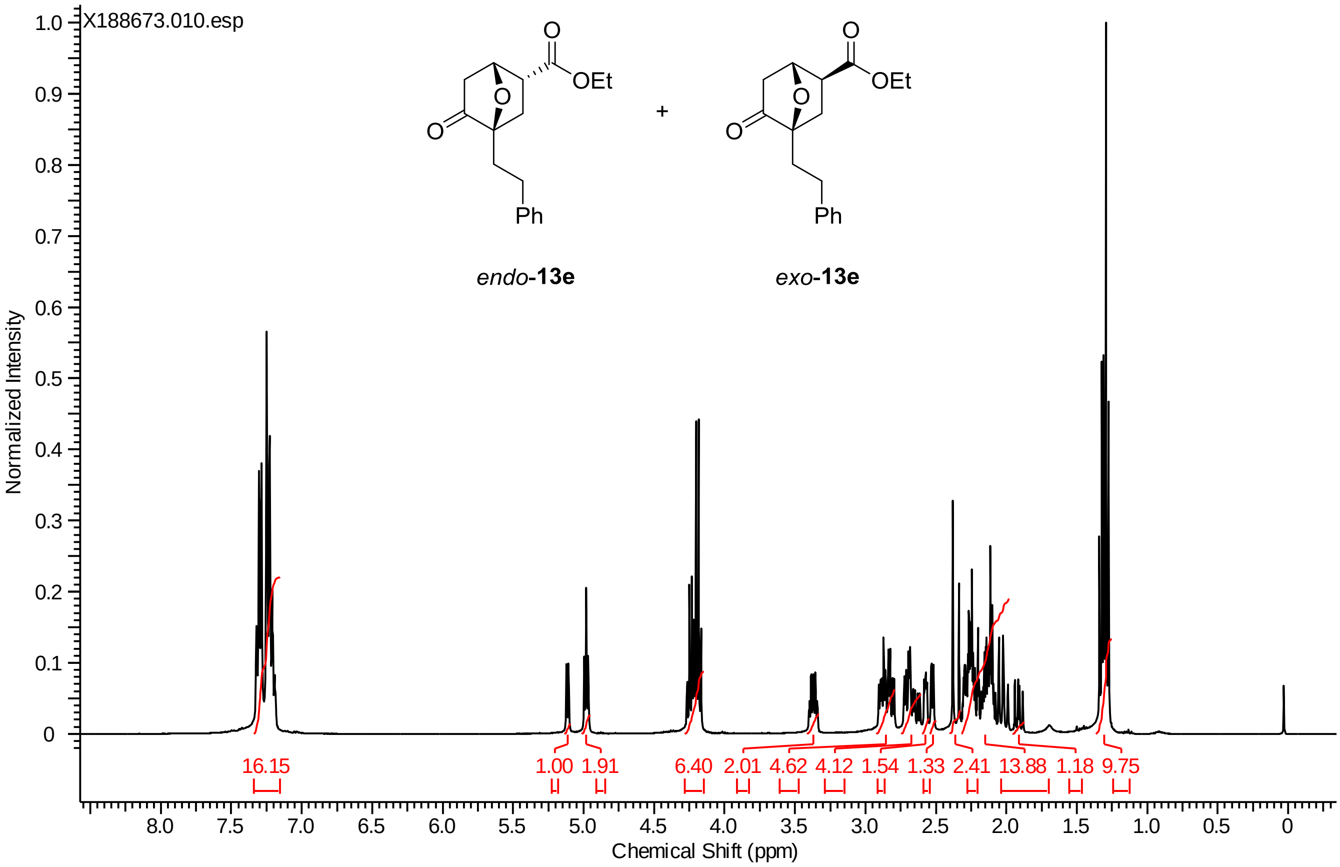

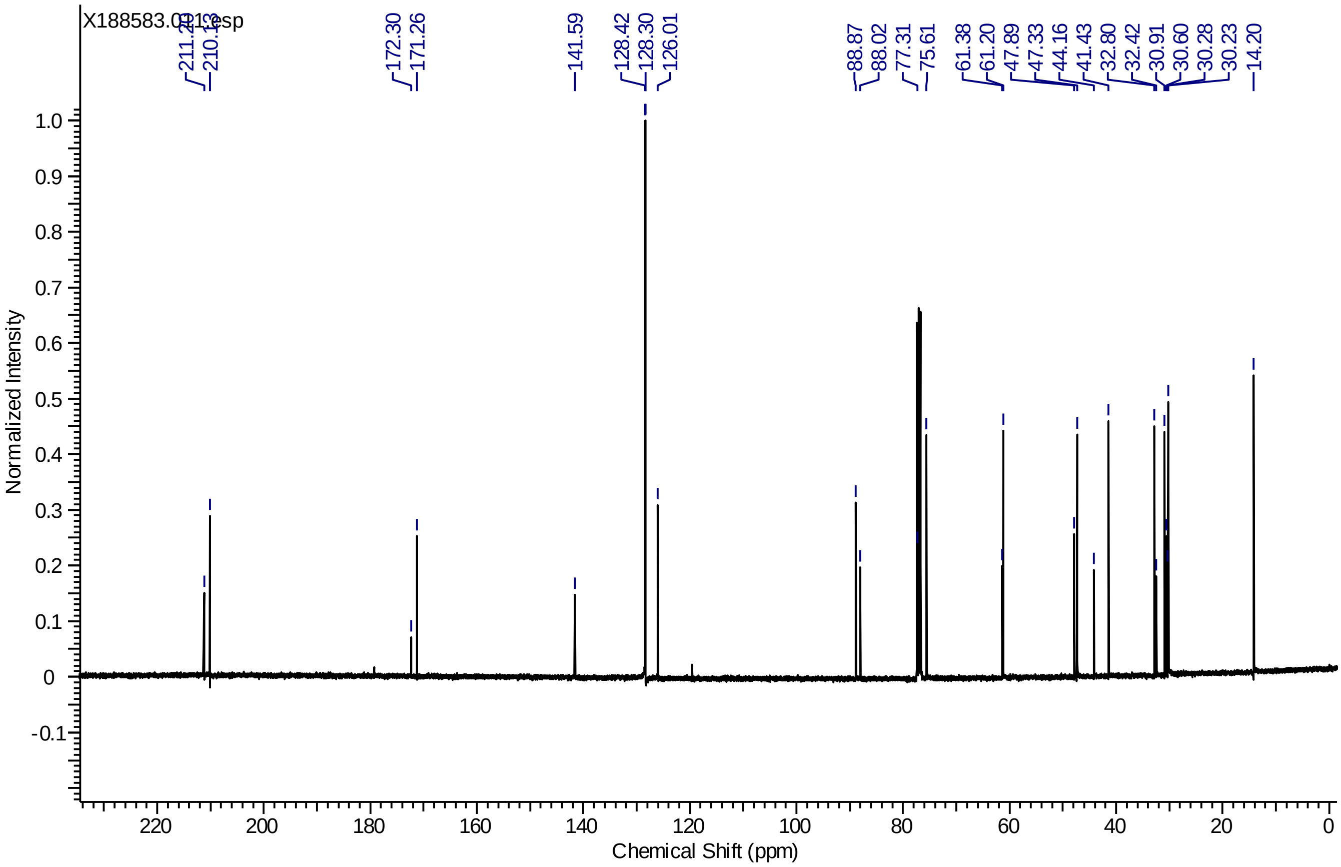

Ethyl (1*R*,2*R*,4*R*)-5-oxo-4-phenethyl-7-oxabicyclo[2.2.1]heptane-2-carboxylate *endo*-13e

^{1}H NMR (600 MHz, $CDCl_3$)

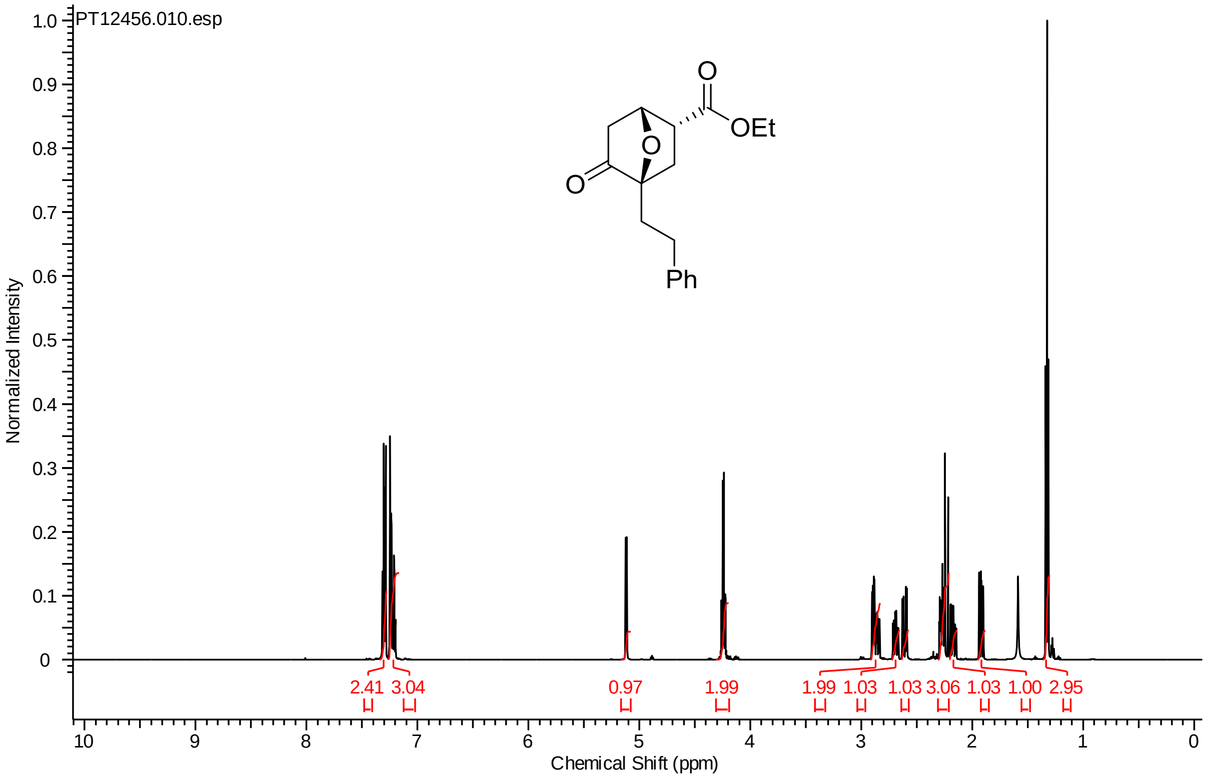

^{13}C NMR (150 MHz, $CDCl_3$)

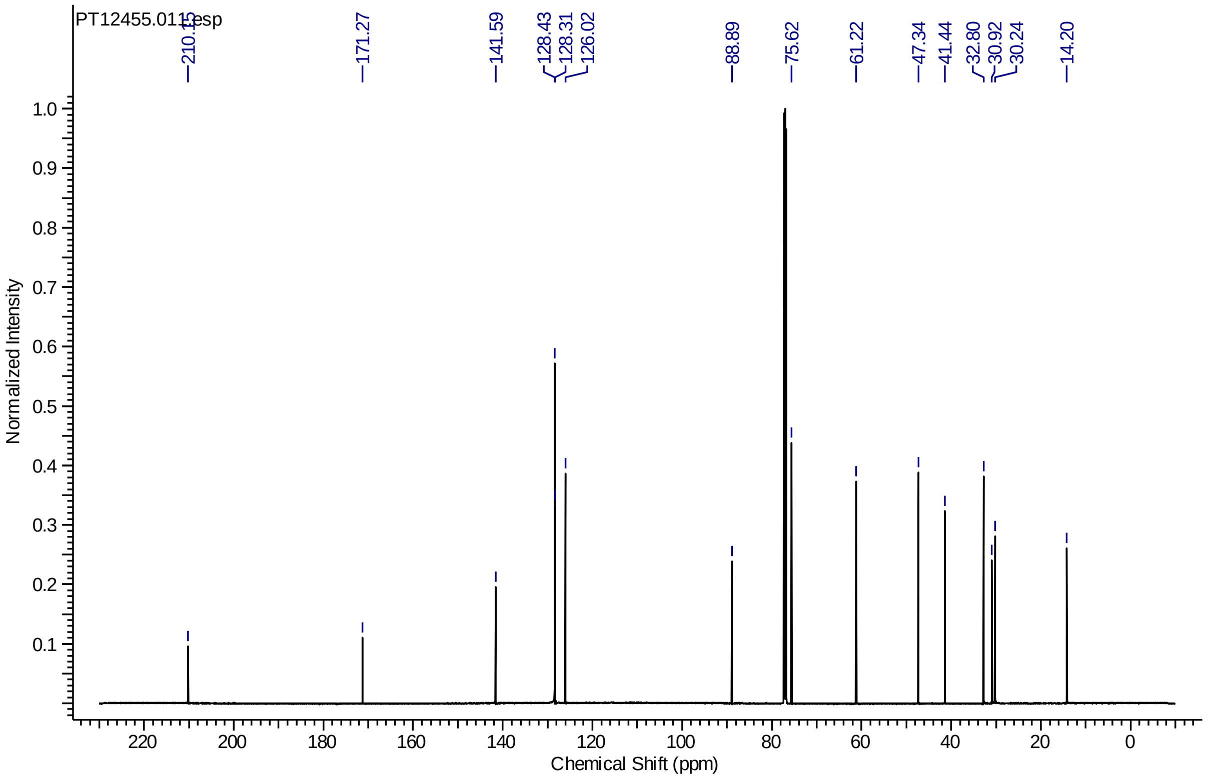

Ethyl (1*R*,2*S*,4*R*)-5-oxo-4-phenethyl-7-oxabicyclo[2.2.1]heptane-2-carboxylate *exo*-13e

^{1}H NMR (600 MHz, $CDCl_3$)

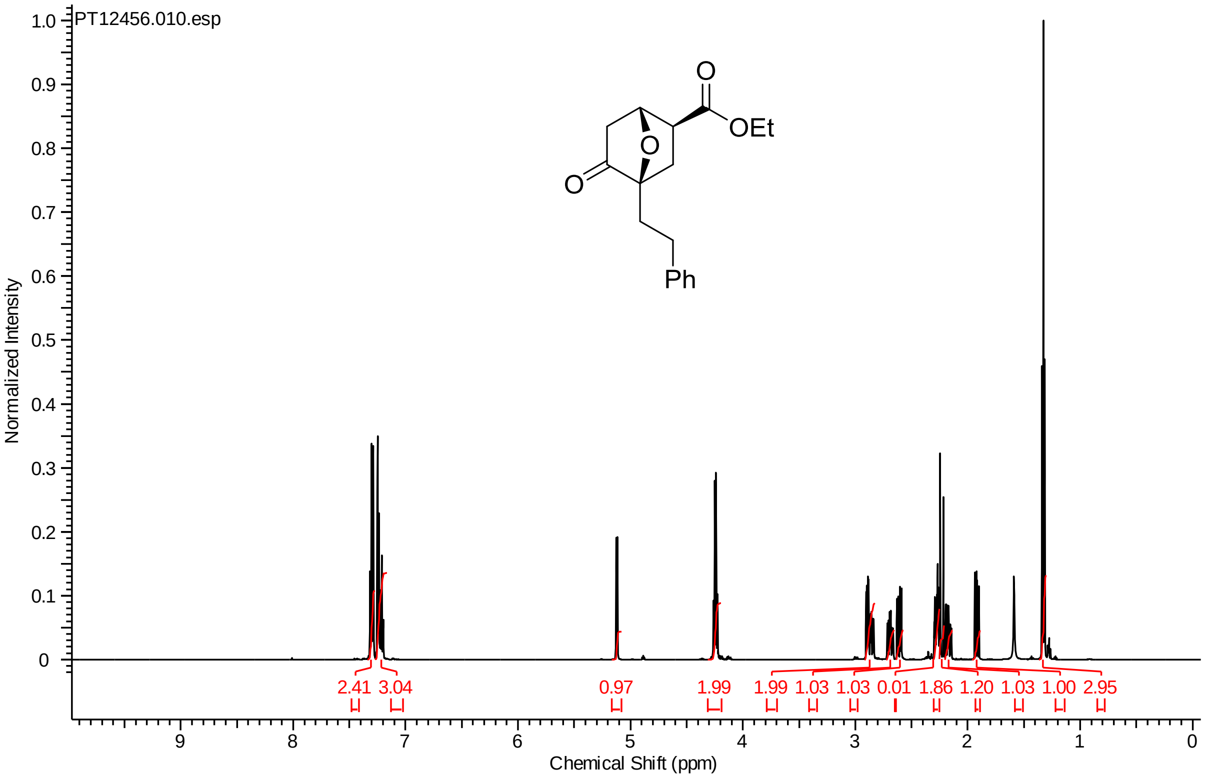

^{13}C NMR (150 MHz, $CDCl_3$)

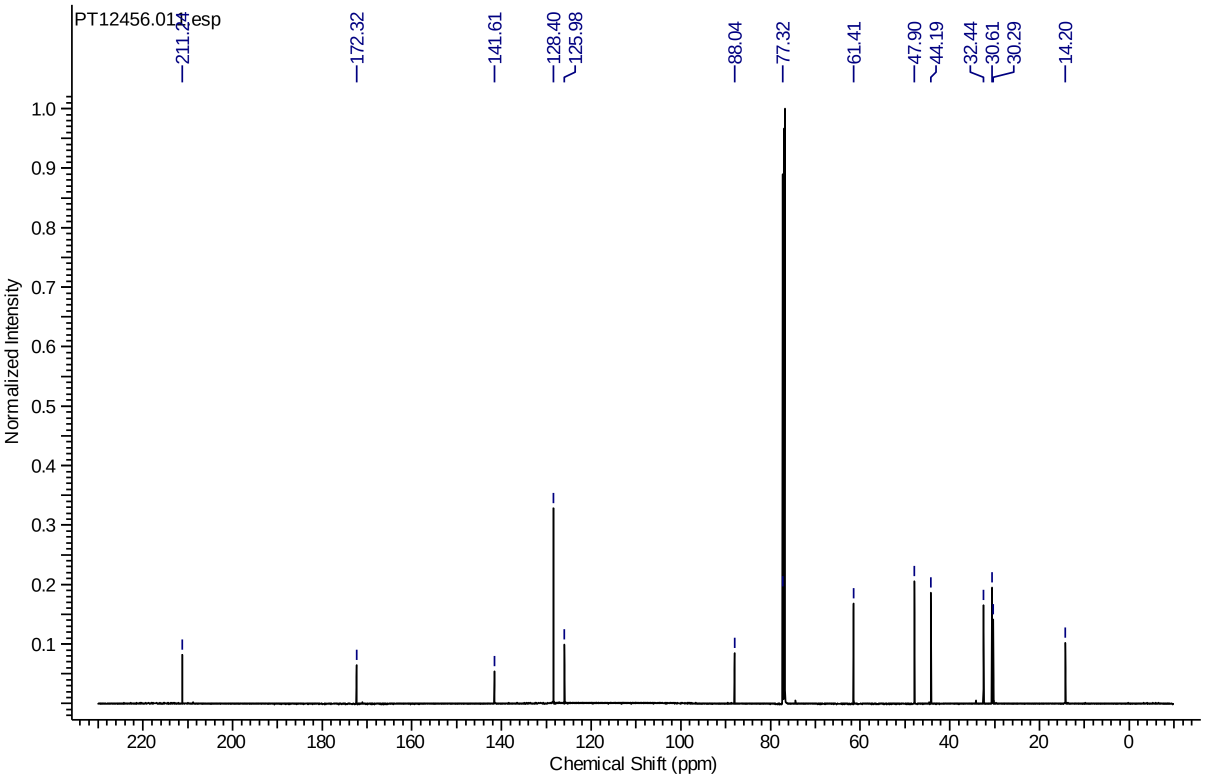

Supplement: Supplementary file 1 [file chem0021-6107-sd1.pdf]
